# Elevation of Cytoplasmic Calcium Suppresses Microtentacle Formation and Function in Breast Tumor Cells

**DOI:** 10.3390/cancers15030884

**Published:** 2023-01-31

**Authors:** Katarina T. Chang, Keyata N. Thompson, Stephen J. P. Pratt, Julia A. Ju, Rachel M. Lee, Trevor J. Mathias, Makenzy L. Mull, David A. Annis, Eleanor C. Ory, Megan B. Stemberger, Michele I. Vitolo, Stuart S. Martin

**Affiliations:** 1 Graduate Program in Molecular Medicine, University of Maryland School of Medicine, 800 W. Baltimore St., Baltimore, MD 21201, USA; 2 Marlene and Stewart Greenebaum NCI Comprehensive Cancer Center, University of Maryland School of Medicine, 655 W. Baltimore St., Baltimore, MD 21201, USA; 3 Graduate Program in Biochemistry & Molecular Biology, University of Maryland School of Medicine, 108 N. Greene St., Baltimore, MD 21201, USA; 4 Graduate Program in Epidemiology and Human Genetics, University of Maryland School of Medicine, 800 W. Baltimore St., Baltimore, MD 21201, USA; 5 Department of Pharmacology and Physiology, University of Maryland School of Medicine, 655 W. Baltimore St., Baltimore, MD 21201, USA; 6 United States Department of Veterans Affairs, VA Maryland Health Care System, Baltimore, MD 21201, USA

**Keywords:** microtentacle (McTN), Ionomycin (Iono), Thapsigargin (Tg), calcium (Ca^2+^), breast cancer, metastasis

## Abstract

**Simple Summary:**

Calcium is a versatile and ubiquitous signaling molecule that long-term dysregulation can increase the spread of cancer to various parts of the body but that short-term effects are understudied. Disseminated cancer cells in circulation have distinct extensions or protrusions, called microtentacles, that enhance their ability to attach to surfaces or other cells. In this study, we show rapidly increasing cellular calcium with the compounds of Ionomycin and Thapsigargin decreases the microtentacle frequency and clustering functions on cancer cells in a detached and suspended environment. Acute calcium-induced signaling events promoted changes to actin contraction and rearrangement responsible for suppressing microtentacles. The results from this study support clinical trial data from Thapsigargin derivatives, suggesting Ca^2+^ modulating therapies can potentially be used to promote cellular shape and structure changes in free-floating tumor cells to reduce metastasis.

**Abstract:**

Cytoskeletal remodeling in circulating tumor cells (CTCs) facilitates metastatic spread. Previous oncology studies examine sustained aberrant calcium (Ca^2+^) signaling and cytoskeletal remodeling scrutinizing long-term phenotypes such as tumorigenesis and metastasis. The significance of acute Ca^2+^ signaling in tumor cells that occur within seconds to minutes is overlooked. This study investigates rapid cytoplasmic Ca^2+^ elevation in suspended cells on actin and tubulin cytoskeletal rearrangements and the metastatic microtentacle (McTN) phenotype. The compounds Ionomycin and Thapsigargin acutely increase cytoplasmic Ca^2+^, suppressing McTNs in the metastatic breast cancer cell lines MDA-MB-231 and MDA-MB-436. Functional decreases in McTN-mediated reattachment and cell clustering during the first 24 h of treatment are not attributed to cytotoxicity. Rapid cytoplasmic Ca^2+^ elevation was correlated to Ca^2+^-induced actin cortex contraction and rearrangement via myosin light chain 2 and cofilin activity, while the inhibition of actin polymerization with Latrunculin A reversed Ca^2+^-mediated McTN suppression. Preclinical and phase 1 and 2 clinical trial data have established Thapsigargin derivatives as cytotoxic anticancer agents. The results from this study suggest an alternative molecular mechanism by which these compounds act, and proof-of-principle Ca^2+^-modulating compounds can rapidly induce morphological changes in free-floating tumor cells to reduce metastatic phenotypes.

## 1. Introduction

Cancer metastasis accounts for up to 90% of all cancer related mortalities [1]. Cancer metastasis is a multi-step process of cancerous cell shedding from the primary tumor and invasion into the vasculature. Detached cancer cells in the vasculature, a nonadherent environment, can travel to distal parts of the body and colonize a secondary site. Cytoskeletal remodeling within cancer cells is an integral component throughout the metastatic cascade, but precise targeting of these dynamic cytoskeletal changes during the metastatic progression remains a challenge [2].

The dynamic components of the cytoskeleton, actin, intermediate filaments, and microtubules, play an important role in cellular architecture and morphology [2,3]. Actin filaments and the microtubule network act in a coordinated manner to maintain cellular structure and facilitate movement within the microenvironment [4]. They also act as counterbalancing forces on the plasma membrane, an idea established by Donald Ingber called tensegrity [2,5,6,7,8]. Cellular tensegrity, based on Ingber’s tensegrity principle, describes a two-part phenomenon where actin filaments at the periphery of the plasma membrane provide an inward force through contraction or polymerization. While the microtubule network, expanding from the microtubule organizing center, provides the counteracting outward force on the plasma membrane. In adherent microenvironments, the inward and outward forces applied to the plasma membrane are balanced. Conversely, in nonadherent microenvironments, these forces become unbalanced, and the outward force from the microtubules overcomes the inward force of actin, leading to the production of microtubule-based protrusions, termed microtentacles (McTNs) [5,6,7,9]. The current working model of McTN suppression is also based on cellular tensegrity. It has been hypothesized the inward force from either increased actin polymerization, decreased actin mesh size, or increased actin cortex stability can overcome the outward force of the microtubules. However, the upstream signaling factors that control this process have yet to be fully elucidated.

The divalent ion calcium (Ca^2+^) is a ubiquitous secondary messenger bridging chemical communication between intracellular and extracellular environments [10,11,12]. Ca^2+^ also acts as an intermediary between the dynamic cytoskeletal components of actin filaments, intermediate filaments, and the microtubule network [13,14]. Additionally, the frequency, amplitude, and magnitude of cytoplasmic Ca^2+^ signals can dictate specific downstream signaling pathways and cellular processes such as cytoskeletal rearrangement, proliferation, migration, invasion, or apoptosis [10,11,12,15]. Historically within the cancer field, studies involving aberrant Ca^2+^ signaling have focused on the activation or expression of calcium channels or receptors and their contribution to the long-term phenotypes of tumor growth, and metastatic progression [10,11,12,16,17,18]. However, recent publications have begun to elucidate the mechanisms mediating short-term Ca^2+^ signaling in breast epithelial and breast cancer cells [19,20,21]. For example, one study demonstrated aggressive metastatic breast cancer cell lines MDA-MB-231 and MDA-MB-436 were less responsive to mechanically stimulated Ca^2+^ signals than nontumorigenic breast epithelial cell counterpart [20]. These studies generate new questions about the role of Ca^2+^ signaling and its contributions to cytoskeletal rearrangement in breast cancer cells in environments where tumor cells are detached and suspended, such as the circulatory and lymphatic systems, during metastasis. The relationship between Ca^2+^ signaling, and McTN formation, a prominent and functionally relevant structure in the metastasis of circulating tumor cells (CTCs), have yet to be explored. Thus, this study aims to elucidate the mechanisms of direct Ca^2+^ signal modulation that affect the cytoskeleton in a free-floating environment.

## 2. Materials and Methods

### 2.1. Cell Lines

MDA-MB-231 and MDA-MB-436 cells were obtained from American Type Culture Collection (ATCC; Manassas, VA, USA) and cultured in Dulbecco’s Modification of Eagle’s Medium (DMEM) (Corning, Manassas, VA, USA) supplemented with 10% Fetal Bovine Serum (Atlanta Biologicals, Flowery Branch, GA, USA), and 1% Penicillin/Streptomycin (Gemini, Sacramento, CA, USA). All cells were cultured at 37 °C with 5% CO_2_ and 95% humidity.

### 2.2. Reagents

Compounds purchased through Sigma Aldrich (St. Louis, MO, USA): Ionomycin (Iono), a Ca^2+^ ionophore [22] (Cat#: 10634), Thapsigargin (Tg), a sarco/endoplasmic Ca^2+^ ATPase (SERCA) pump inhibitor [23] (Cat#:T9033), and Dimethyl sulfoxide (DMSO) (Cat#: 276855).

### 2.3. Kinetic Calcium Assay

A collagen layer was coated rotating overnight at 4 °C on a clear bottom 96-well black plate (Corning 3603) as previously described [21]. Cells were then plated in the prepared plate at 50,000 cells per well for a confluent monolayer. Cells were then loaded with 4 μM Fluo4-AM (Life Technologies F14201) and prepared as previously described [21] prior to the addition of compound. The final volume per well was 100 μL of Hank’s Balanced Salt Solution (HBSS + Ca^2+^; Gibco 14025-092).

Dilutions of compounds were prepared in a separate plate at 5× concentration for the robotic addition of 25 μL to cells by the FLEX Station 3 Multi-Mode plate reader (Molecular Devices). The FLEX Station 3 reads Relative Fluorescence Units (RFUs) measured every 1.28 s for a period of 300 s after compound addition. The results were either plotted as the maximum-minimum for each compound concentration over the time period or ΔF/F_0_ as previously calculated [21] with the initial 30 s reading as a baseline value prior to compound addition. All values shown are mean ± SD of triplicate samples.

### 2.4. Cell Viability

White 96-well tissue culture plates (Greiner Bio-One 655180) were PEM coated with a single bilayer and formaldehyde crosslinkedas previously described [24]. Cells were seeded in triplicate at 5000 cells per well in culture media in the presence of DMSO, Staurosporine (Sigma S6942 (Stauro)) or select concentrations of Iono, or Tg for 0, 6, 24 h. After respective drug treatment times, Promega Cell titer Glo (G7571), was added. Cells were prepared following the manufacturer’s instructions, and luminescence was read on the FLEX Station 3 (Molecular Devices). Viability was measured as a percentage of ATP production at time 0 ([luminescence at hour x ÷ average luminescence at time 0] × 100). All values shown are mean ± SD of triplicate samples.

### 2.5. McTNs Scoring and Analysis

Cells were trypsinized and suspended in DMEM in the presence of DMSO, Iono or Tg for 30 min while in suspension, and then tethered, fixed, and, stained on our TetherChip Technology as previously described [25,26]. For zero Ca^2+^ conditions, cells were initially washed in zero Ca^2+^ Hank’s Balanced Salt Solution (HBSS−Ca^2+^; Gibco 1424-092) then suspended in HBSS−Ca^2+^ supplemented with 100 μM ethylene glycol-bis(β-aminoethyl-ether)-N,N,N’,N’-tetra acetic acid (EGTA) (Sigma E3889) alone or with Iono or Tg. For inhibition of actin polymerization experiments, detached and suspended cells were subjected to an initial 15 min pretreatment of 5 μM Latrunculin A (LA) (Emdmillipore 428021) prior to the addition of DMSO, Iono or Tg. Samples were treated for 30 min in suspension then tethered, fixed, and stained as previously described [25,26]. Blind scoring for the presence of McTNs in a given population of 100 cells per channel was conducted. Cell positivity was defined by a blinded individual observer as to whether the suspended cells are producing at least two membrane protrusions greater than the radius of the cell body [25]. Additionally, single whole cell perimeter analysis was performed, as previously described [25,26].

### 2.6. Cellular Clustering Assay

In the presence of Iono, Tg or DMSO, cells were allowed to aggregate for 6 h in a 96-well low-attached plate (50,000 cells/well) then transferred to our TetherChip Technology [26]. Cells were fixed using 4% formaldehyde and stained with Hoechst 3325 (1:1000). Images were acquired using the Nikon Eclipse Ti2-E inverted microscope with a 4× air objective. Images were auto-contrasted in Nikon Elements software before being analyzed as previously described (https://github.com/ScientistRachel/CellAggregationAnalysis) accessed on 13 December 2021 [27].

### 2.7. Cell-Electrode Impedance Reattachment Assay

Real-time monitoring of cellular reattachment of suspended cells was measured using the xCelligence RTCA-DP real-time sensing device (Agilent Technologies, Santa Clara, CA, USA) to compare attachment rates of cells treated in DMSO, Iono or Tg.

MDA-MB-231 cells and MDA-MB-436 cells were grown to 80% confluency in a 10 cm tissue culture dish or a 6-well tissue culture plate. Both cell lines were then detached and seeded at 40,000 cells per well. Cell impedance was recorded every 5 min over a 24 h time course.

Live cell imaging of precipitation, attachment and spreading of cells in suspension attaching to the bottom of a 12-well tissue culture-treated plate was taken using the Nikon Eclipse Ti2-E inverted microscope with a Tokai-Hit stage top incubation chamber. Population images were collected every hour over a 17 h time course at a 10× air objective with phase contrast.

### 2.8. Immunoblotting

Cells were trypsinized and suspended in DMEM with either DMSO, Iono, or Tg treated for 5 min in a 6-well low-attached plate (Corning). Cells were centrifuged at 1000 rpm for 5 min. Lysates were prepared and immunoblot analysis was conducted as previously described [28] using the following antibodies: Anti-Acetyl-α-tubulin (Lys40) (D20G3) XP (1:1000, Cell Signaling Technologies, CST5335, Danvers, MA, USA), Anti-α-tubulin (1:1000, Sigma T6199), Anti-Detyrosinated alpha tubulin (1:1000, Abcam, ab48389, Cambridge, UK), Anti-GAPDH (1:1000, Santa Cruz sc32233, Santa Cruz, CA, USA), Anti-Myosin Light Chain 2 (D18E2) (1:1000, CST8505), Anti-phospho-myosin light chain 2 (Ser19) (1:1000, CST3675), Anti-myosin phosphatase 1 (D6C1) (1:1000, CST8574), Anti-phospho-myosin phosphatase (Thr853) (1:1000, CST4563), Anti-phospho-cofilin (Ser3) (77G2) (1:1000, CST3313), Anti-cofilin (D3F9) (1:1000, CST5175). Densitometry analysis was conducted across 3 independent experiments using ImageJ software. Original immunoblot images and additional detailed densitometry method and analysis using the iBright Software (ThermoFisher, Waltham, MA, USA) can be found in the supplementary material method S1, Supplementary Figures and File S1.

### 2.9. Confocal Microscopy

All confocal imaging was conducted on tethered and fixed cells using an Olympus IX81 microscope with a FV-1000 confocal laser scanning system with a 60× oil emersion magnification and objective lens numerical aperture of 1.42. Z-stack sliced images were taken at 0.5 μm slice along the entire thickness of each cell.

### 2.10. Statistical Analysis

Statistical analyses were conducted using either a *t*-test, or one-way ANOVA with Bonferroni’s multiple comparison test in GraphPad Prism 9.0 software; *p* < 0.05 was considered significant. Outlier data in the clustering efficiency analysis were identified using the ROUT method with Q = 1% in GraphPad Prism 9.0 software. Biological replicates with an identified outlier were not included in the paired t-test for cellular clustering efficiency analysis.

## 3. Results

### 3.1. Increasing Concentrations of Ionomycin or Thapsigargin Induces an Elevation of Cytoplasmic Calcium in Breast Cancer Cells

To begin to understand how cytoplasmic Ca^2+^ affects McTN production, we initially validated whether the compounds Ionomycin (Iono) and Thapsigargin (Tg), a, induce an elevation in cytoplasmic Ca^2+^ in the metastatic breast cancer cell lines, MDA-MB-231 and MDA-MB-436. Iono facilitates the movement of extracellular Ca^2+^ ions across the plasma membrane into the cytoplasm, while Tg diffuses across the plasma membrane to inhibit the reuptake of Ca^2+^ into the endoplasmic reticulum thereby increasing cytoplasmic Ca^2+^ [22,23,29]. Using both compounds to increase cytoplasmic Ca^2+^ concentrations, we can further test the impact of Ca^2+^ entry from different sources on cancer cell functions and phenotypes in detached and suspended conditions in future assays.

**Figure 1:**
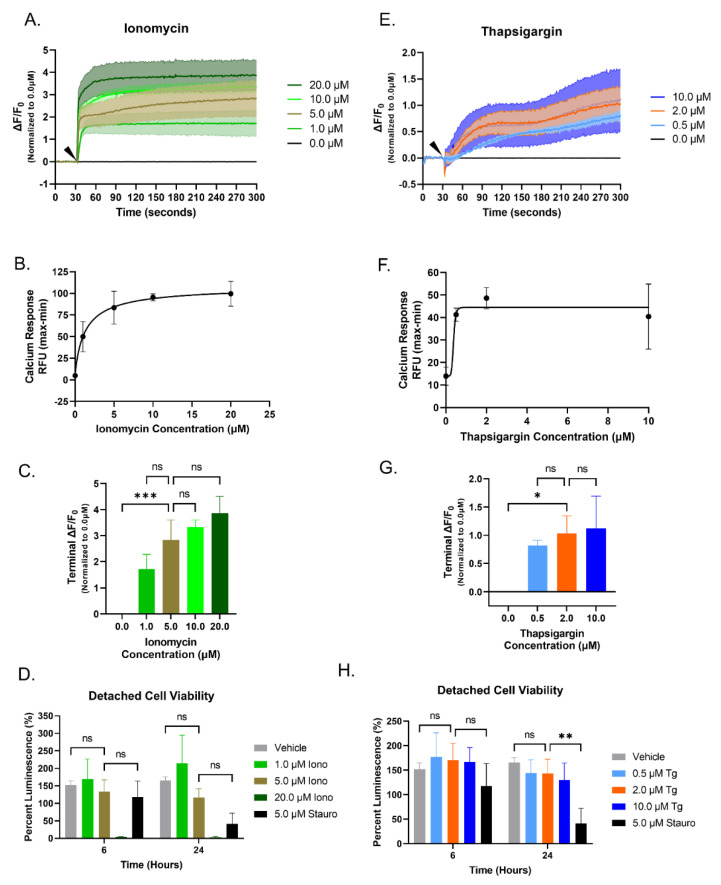
Ionomycin and Thapsigargin cause an increase in cytoplasmic Ca^2+^ in a dose-dependent manner in MDA-MB-231 cells. (**A**–**C**,**E**–**G**) After a 30 s basal recording of fluorescence (Ex494 nm; Em: 516 nm), an arrow indicates the increasing concentrations of Ionomycin or Thapsigargin that were added and continually recorded for an additional 270 s. Relative fluorescence unit (RFU) data was analyzed in GraphPad Prism and presented as change in RFU divided by the initial RFU (ΔF/F_0_). Data shown are mean ± SD and representative of 3 independent experiments performed in triplicate. (**A**) Traces of increasing cytoplasmic Ca^2+^ (ΔF/F_0_) of MDA-MB231 cells in response to the addition of increasing concentrations of Ionomycin. (**B**) EC50 Ca^2+^ response curve (RFU) for increasing concentrations of Ionomycin. (**C**) Terminal ΔF/F_0_ value for each concentration of Ionomycin used. (**D**) MDA-MB-231 cell viability in a suspended state with different concentrations of Ionomycin treatment over a 24 h time course. (**E**) Increasing cytoplasmic Ca^2+^ response (ΔF/F_0_) traces to the addition of increasing Thapsigargin concentrations. (**F**) EC50 Ca^2+^ response curve (RFU) of MDA-MB-231 cells for increasing concentrations of Thapsigargin. (**G**) Terminal ΔF/F_0_ value for each concentration of Thapsigargin. (**H**) Cell viability of MDA-MB-231 cells in suspension with increasing concentrations of Thapsigargin treatment over 6- and 24- hour treatment. (**D**,**H**) Data shown are mean ± SD and representative of 3 independent experiments performed in triplicate. n.s., * *p* < 0.05, ** *p* < 0.01, and *** *p* < 0.001.

A standard Ca^2+^ dye assay was used to measure real-time changes in fluorescence measured as a Relative Fluorescence Unit (RFU). ΔF/F_0_ was calculated from the recorded RFU values and shown as ΔF/F_0_ traces over a 300 s time course. After an initial 30 s baseline reading, increasing concentrations of Iono or Tg were added to an adherent monolayer of cells. Increasing concentrations of Iono added to MDA-MB-231 cells caused a sharp and sustained increase in ΔF/F_0_ in a dose-dependent manner (Figure 1A). The difference between the minimum RFU value recorded and the maximum RFU value was used to create the EC50 Ca^2+^ response curve. In the MDA-MB-231 cells, 5 μM Iono was the lowest concentration to elicit a maximal Ca^2+^ response (Figure 1B). Since the minimal or maximal reading is not necessarily seen at the beginning or end of each reading, the terminal ΔF/F_0_ value was analyzed to determine potential differences between the concentrations. While 20 μM Iono had the greatest terminal ΔF/F, when compared to the 5 μM Iono, there was no statistical difference (Figure 1C).

While McTN extension can be measured in less than an hour, the phenotypic consequences of initial McTN extensions are measured with longer-term assays such as cell clustering (6 h) or cell reattachment (24 h). A cell viability study was conducted to determine the potential toxicity of compounding variables, such as the nonadherent environment and the various compound concentrations capable of triggering a cytoplasmic Ca^2+^ increase. Cell viability was calculated by measuring luminescence at 0, 6, and 24 h time points. MDA-MB-231 cells were seeded into increasing concentrations of 1, 5, and 20 μM Iono. Treatment with 20 μM of Iono was shown to be extremely cytotoxic at 6 and 24 h, while 1 μM Iono treatment had limited cytotoxic effects at the time points of interest (Figure 1D). The concentration of interest, 5 μM Iono, also showed limited cytotoxic effects at 6 and 24 hours (Figure 1D). Therefore, the optimal concentration for Iono used for further investigation was 5 μM.

The Ca^2+^ assay and cytotoxicity experiments were repeated to determine the optimal concentration of Tg needed to trigger an increase in cytoplasmic Ca^2+^ at low cytotoxic levels. Using the same Ca^2+^ dye-based assay, increasing concentrations of Tg were added to MDA-MB-231 cells. Graphical ΔF/F0 traces illustrate a gradual rise in cytoplasmic Ca^2+^ over time. Increasing additions of Tg show that 2 µM and 10 µM Tg have overlapping ΔF/F0 traces that indicate both concentrations achieve a maximal response (Figure 1E). The EC50 Ca^2+^ response curve also shows the addition of 2 μM Tg was sufficient to elicit a maximal Ca^2+^ response similar that of 10 μM Tg (Figure 1F). Additional analysis of the calculated end ΔF/F values found no statistical difference between 10 μM Tg and 2 μM Tg (Figure 1G). Cytotoxicity experiments showed increased concentrations of Tg had limited cytotoxic effects at 6 and 24 hours (Figure 1H). Collectively, these data indicate 2 µM Tg is the optimal concentration to use for subsequent studies.

**Figure 2:**
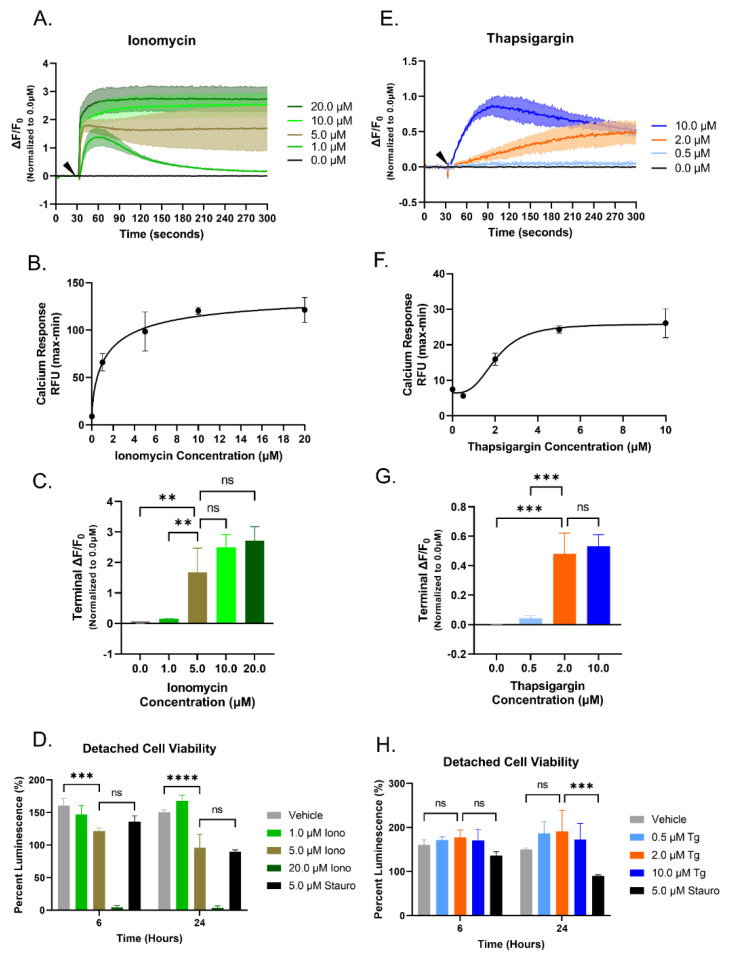
Ionomycin and Thapsigargin induce an elevation of cytoplasmic Ca^2+^ in a dose-dependent manner in MDA-MB-436 cells. (**A**–**C**,**E**–**G**) After a 30 s basal recording of fluorescence (Ex494 nm; Em: 516 nm), an arrow indicates the increasing concentrations of Iono or Tg that were added and continually recorded for an additional 270 s. Relative fluorescence unit (RFU) data was analyzed in GraphPad Prism and presented as change in RFU divided by the initial RFU (ΔF/F_0_). Data shown are mean ± SD and representative of 3 independent experiments performed in triplicate. (**A**) Traces of increasing cytoplasmic Ca^2+^ (ΔF/F_0_) of MDA-MB-436 cells in response to the addition of increasing concentrations of Iono. (**B**) EC50 Ca^2+^ response curve (RFU) for increasing concentrations of Iono. (**C**) Terminal ΔF/F_0_ value for each concentration of Iono used. (**D**) MDA-MB-436 cell viability in a suspended state with different concentrations of Iono treatment over a 24 h time course. (**E**) Increasing cytoplasmic Ca^2+^ response (ΔF/F_0_) traces to the addition of increasing Tg concentrations. (**F**) EC50 Ca^2+^ response curve (RFU) of MDA-MB-436 cells for increasing concentrations of Tg. (**G**) Terminal ΔF/F_0_ value for each concentration of Tg. (**H**) Cell viability of MDA-MB-436 cells in suspension with increasing concentrations of Tg treatment over 6- and 24- hour treatment. (**D**,**H**) Data shown are mean ± SD and representative of 3 independent experiments performed in triplicate. n.s., ** *p* < 0.01, *** *p* < 0.001 and **** *p* < 0.0001.

Breast cancer is a heterogeneous disease with diverse genetic backgrounds, resulting in differing functional and biological effects. Therefore, a second metastatic breast cancer cell line, MDA-MB-436 cells, was tested to determine the minimal doses of Iono and Tg to elicit the maximum cytoplasmic Ca^2+^ increase without affecting cell viability. The MDA-MB-436 cells demonstrated different Ca^2+^ kinetic responses to the addition of Iono and Tg from that of the MDA-MB-231 cell line (Figure 2A–C,E–G). However, similar to the MDA-MB-231 cells, the concentrations of interest, 5 μM Iono and 2 μM Tg were able to elicit an increase in cytoplasmic Ca^2+^ over 300 s time course and with limited cytotoxic effects at 6- and 24 h (Figure 2D,H).

### 3.2. Elevated Cytoplasmic Calcium Suppresses Microtentacle Formation

**Figure 3:**
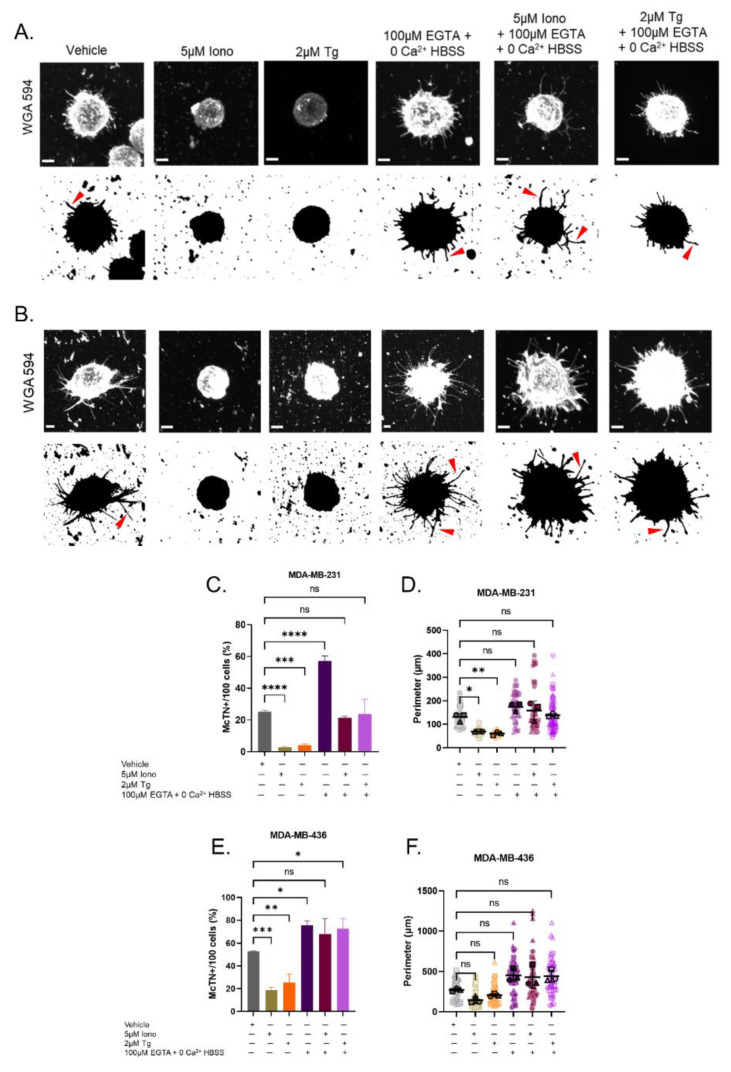
Induce elevation of cytoplasmic Ca^2+^ with Ionomycin or Thapsigargin suppresses McTNs in the presence of extracellular Ca^2+^. (**A**,**B**) Representative single cell maximum intensity confocal images (wheat germ agglutinin (WGA) 594 (1:100)) and their black and white contrast counterparts shown. MDA-MB-231 and MDA-MB-436 cells treated with vehicle (0.5% DMSO), 5 µM Ionomycin, 2 µM Thapsigargin, 100 µM ethylene glycol-bis(β-aminoethyl-ether)-N,N,N’,N’-tetra acetic acid (EGTA) supplemented with Hank’s Balanced Salt Solution without Ca^2+^, or 100 µM EGTA supplemented with Hank’s Balanced Salt Solution without Ca^2+^ and 5 µM Ionomycin or 2 µM Thapsigargin. Arrows indicating microtentacles on the cell. Scale bar = 5 μM. (**A**) MDA-MB-231 cells treated with 5 µM Ionomycin or 2 µM Thapsigargin in full Ca^2+^ shows a suppression of McTN formation. (**B**) 5 µM Ionomycin or 2 µM Thapsigargin treatment in MDA-MB-436 cells displays a suppression of McTN formation. (**C**) MDA-MB-231 cell positivity for microtentacle formation decreases with Ionomycin or Thapsigargin treatment in full Ca^2+^ conditions. (**D**) MDA-MB-231 whole cell perimeter significantly decreases with Ionomycin or Thapsigargin treatment in the presence of extracellular Ca^2+^. (**E**) MDA-MB-436 cell positivity for McTN formation decreases with Ionomycin or Thapsigargin treatment in full Ca^2+^ conditions. (**F**) MDA-MB-436 whole cell perimeter does not significantly decrease with Ionomycin or Thapsigargin treatment in the presence of extracellular Ca^2+^. (**C**,**E**) Data shown as mean ± SD, *n* = 3. (**D**,**F**) Data shown as mean ± SD, *n* = 3, determined from a total of 20 to 30 cells from combined biological replicates. n.s., * *p* < 0.05, ** *p* < 0.01, *** *p* < 0.001, and **** *p* < 0.0001.

Normal and malignant breast epithelial cells have varying frequencies of tubulin-based protrusions termed McTNs when placed in a nonadherent microenvironment. Both genetic and chemical methods can modulate McTN levels, but the effects on Ca^2+^-mediated signaling pathways on McTNs have not yet been investigated. The metastatic breast cancer lines of MDA-MB-231 and MDA-MB-436 produce moderate-to-high levels of McTNs in detached and suspended environments [25,30]. Therefore, using these cell lines, we can establish the direction of change in the McTN phenotype due to the elevation of cytoplasmic Ca^2+^. Cells were treated for 30 min with the vehicle (0.5% DMSO), 5 μM Iono, or 2 μM Tg in suspension, and then transferred to a TetherChip for fixation, nuclear and plasma membrane staining, visualization, and analysis. Representative single cell confocal images from both MDA-MB-231 and MDA-MB-436 cell lines treated with 5 μM Iono or 2 μM Tg show a decrease in McTN formation compared to the vehicle control (Figure 3A,B). To further probe into the role of extracellular Ca^2+^ in McTN suppression, 100 μM EGTA (an extracellular calcium chelator) was supplemented into Hank’s Balanced Salt Solution without Ca^2+^ (HBSS–Ca^2+^) to remove all extracellular Ca^2+^ and create a near-zero Ca^2+^ condition. Images illustrate that the complete ablation extracellular Ca^2+^ does not change the McTN phenotype (Figure 3A,B). Furthermore, simultaneous chelation of extracellular Ca^2+^ while adding either 5 μM Iono or 2 μM Tg maintained the McTN phenotypes in both MDA-MB-231 and MDA-MB-436 cell lines (Figure 3A,B).

Cell positivity for McTN production was used to quantify the differences observed between the various treatment groups. Quantification of MDA-MB-231 cells treated in full Ca^2+^ with either 5 μM Iono or 2 μM Tg significantly decreased McTN positivity compared to the vehicle (Figure 3C). These results were replicated in MDA-MB-436 cells (Figure 3E). -MDA-MB-231 subjected to only zero Ca^2+^ conditions significantly increased in McTN positivity compared to the vehicle in full Ca^2+^ (*p* < 0.0001) (Figure 3C). MDA-MB-436 cells also recapitulated a significant increase in McTN positivity in zero Ca^2+^ HBSS conditions compared to the complete Ca^2+^ condition vehicle control (*p* = 0.0178) and (Figure 3E). The concurrent treatment with 5 μM Iono or 2 μM Tg in HBSS–Ca^2+^ supplemented with 100 μM EGTA did not significantly alter cell positivity in the MDA-MB-231 cells compared to the vehicle in full Ca^2+^ (Figure 3C). Additionally, the change in McTN positivity was not significant when MDA-MB-436 cells were treated with 5 μM Iono in the absence of Ca^2+^ compared to the vehicle. However, when MDA-MB-436 cells were simultaneously treated with 2 μM Tg in HBSS–Ca^2+^, cell positivity for McTNs significantly increased compared to the vehicle control in the presence of Ca^2+^ (*p* = 0.0440) (Figure 3E).

The quantitative measurement of the whole cell perimeter was also examined as an orthogonal approach to using of the investigator’s defined cell McTN positivity. The whole cell perimeter is defined as the perimeter, including the cell body and McTNs. Changes in the whole cell perimeter can be used as a metric of McTN positivity compared to the vehicle but will not reflect the number of McTNs on each cell or the structure of individual McTNs. Analysis of maximum intensity projection images in ImageJ revealed a significant reduction in whole cell perimeter for MDA-MB-231 cells treated with either 5 μM Iono or 2 μM Tg compared to the vehicle (Figure 3D). 5 μM Iono or 2 μM Tg treatment in MDA-MB-436 cells decreased in whole cell perimeter but did not reach statistical significance (Figure 3F). The whole cell perimeter of MDA-MB-231 and MDA-MB-436 cells in the absence of all extracellular Ca^2+^ alone remained unchanged compared to the vehicle in full Ca^2+^ (Figure 3D,F). Furthermore, the treatment of either 5 μM Iono or 2 μM Tg in HBSS–Ca^2+^ did not change the whole cell perimeter in either cell line compared to their corresponding vehicle in full Ca^2+^ (Figure 3D,F).

### 3.3. Distinct Calcium Entry Sources Result in an Inhibited Reattachment Response to Surfaces

**Figure 4:**
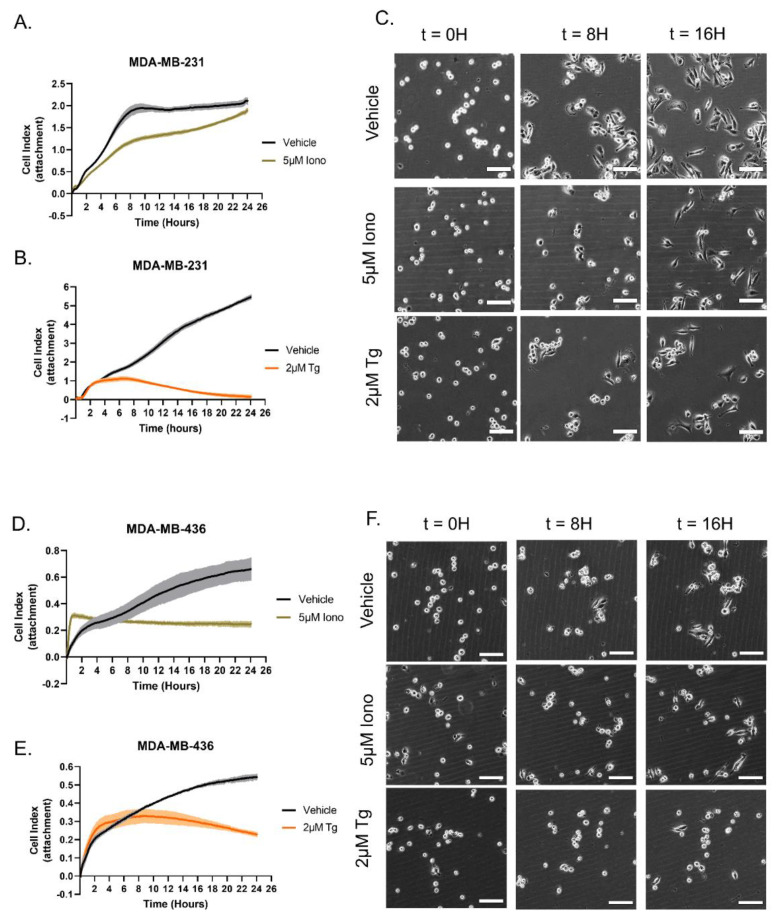
Ionomycin or Thapsigargin treatment inhibits cellular reattachment in MDA-MB-231 and MDA-MB-436 cells. (**A**) Representative graph shows treatment with Ionomycin reduces initial attachment and spreading but recovers over time in MDA-MB-231 cells. (**B**) Representative graph shows Thapsigargin treatment significantly inhibits cellular reattachment over time in MDA-MB-231 cells. (**A**,**B**) Technical quadruplicates with data shown as mean ± SD. (**C**) Enlarged representative cell population images extracted from 17 h time course video. At time points 0, 8, and 16 h, MDA-MB-231 cells are visualized attaching to the bottom of the tissue culture-treated plate in either vehicle control, 5 µM Ionomycin or 2 µM Thapsigargin treatments. (**D**) Representative graphs shows 5 μM Ionomycin-treated MDA-MB-436 cells have a decreased reattachment trend in comparison to the vehicle control. (**E**) Representative graphs show Thapsigargin-treated MDA-MB-436 cells have a decreased reattachment trend in comparison to the vehicle control. (**D**,**E**) Data shown as mean ± SD of technical triplicates. (**F**) Representative enlarged region of interest populations of interest were extracted from a 17 h time course video. At time points 0, 8, and 16 h MDA-MB 436 cells were visualized attaching to the bottom of the tissue culture-treated plate with either vehicle control, 5 μM Iono, or 2 μM Tg. (**C**,**F**) The time course video was taken on the Nikon Eclipse Ti2-E inverted microscope using a 10× phase contrast air objective with the Tokai-Hit incubation stage. Scale bar = 100 μM.

We have previously demonstrated that elevated levels of McTNs in free-floating cells enhance reattachment to surfaces [7,28,30]. Therefore, we hypothesized the suppression of McTNs using either Iono or Tg in detached breast cancer cells would inhibit reattachment. To test this hypothesis, MDA-MB-231 cells or MDA-MB-436 cells were seeded in a cell-electrode impedance reattachment assay plate (E-plate) with either Iono, Tg, or vehicle control to measure attachment after suspension over a 24 h time course. MDA-MB-231 cells treated with either 5 μM Iono or 2 μM Tg showed a general inhibition of reattachment over the 24 h time course (Figure 4A,B). Changes in electrical impedance trends for MBA-MB-231 cells treated with 5 μM Iono displayed inhibition of reattachment compared to the vehicle control that was eventually overcome during the 24 h time course (Figure 4A). However, MDA-MB-231 cells treated with 2 μM Tg showed significant suppression of electrical impedance trend when compared to the vehicle control throughout the 24 h time course (Figure 4B). Complementary phase contrast images at the 8 h and 16 h time points visualize cellular reattachment patterns to the bottom of a tissue culture plate for the different treatment groups. MDA-MB-231 cells treated with either 5 μM Iono or 2 μM Tg showed more cellular rounding at 8 h and 16 h time points compared to the vehicle control (Figure 4C). For the MDA-MB-436 cells treated with either Iono or Tg, the xCelligence reattachment electrical impedance trends decrease in comparison to the vehicle control (Figure 4D,E). Additional live cell time course imaging of reattachment to the bottom of a tissue culture-treated plate demonstrated that Iono and Tg-treated MDA-MB-436 cells exhibit enhanced cellular rounding at 8 h and 16 h time points when compared to the vehicle control-treated cells (Figure 4F).

### 3.4. Increasing Cytoplasmic Calcium Decreases Homotypic Cellular Clustering

**Figure 5:**
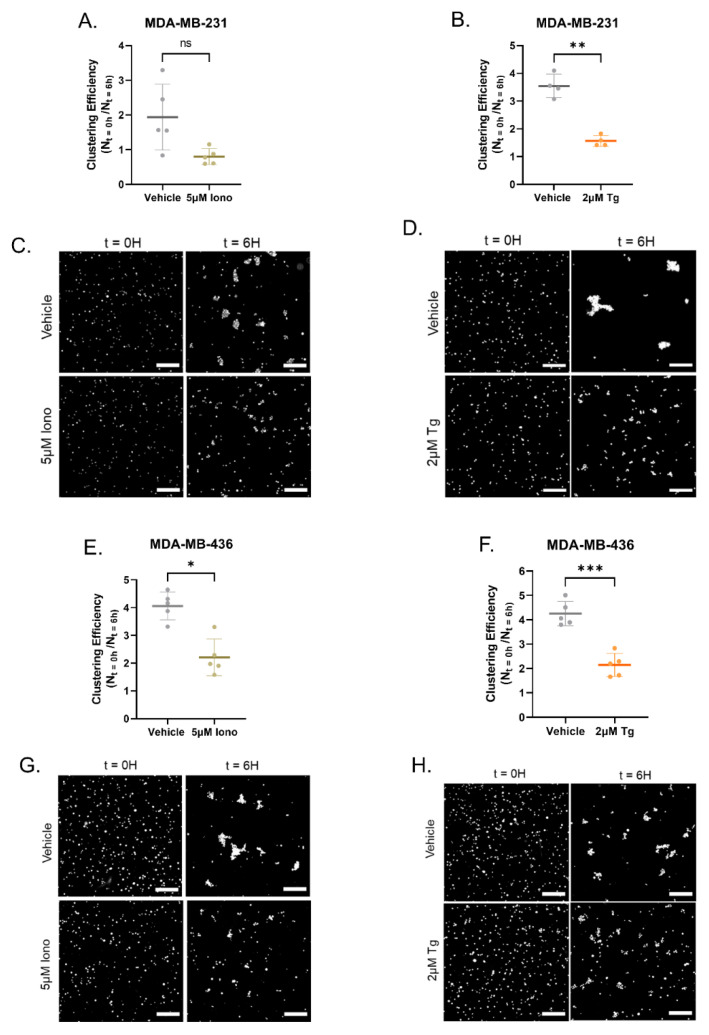
Induced elevation of cytoplasmic Ca^2+^ in MDA-MB-231 and MDA-MB-436 cells decreases homotypic clustering. (**A**) Trending decrease of clustering efficiency with a treatment of 5 μM Ionomycin in MDA-MB-231 cells over 6 h. Data are shown as mean ± SD, *n* = 5. (**B**) MDA-MB-231 cells with 2 μM Thapsigargin 6 h treatment significantly decreases clustering efficiency. Data are shown as mean ± SD, *n* = 4; a fifth biological replicate was removed because it was identified as an outlier with the ROUT test with a Q = 1%. (**C**) Representative zoomed black and white images of nuclear staining show MDA-MB-231 cells with 5 µM Ionomycin and vehicle treatment density at t = 0 and t = 6 h. (**D**) Representative enlarged region of interest black and white images of Hoescht staining show MDA-MB-231 cells with 2 μM Thapsigargin and vehicle treatment density at t = 0 and t = 6 h. (**E**) 5µM Ionomycin treatment in MDA-MB-436 cells decreases clustering efficiency over 6 h. (**F**) Thapsigargin treatment in MDA-MB-436 cells significantly decreased clustering efficiency in comparison to the vehicle control. (**E**,**F**) Data shown as mean ± SD of 5 independently conducted experiments. (**G**) MDA-MB-436 representative enlarged black and white images of nuclear staining shows 5 µM Iono and vehicle treatment density at t = 0 and t = 6 h. (**H**) Representative enlarged black and white MDA-MB-436 images of nuclear staining shows 2 µM Tg and vehicle treatment density at t = 0 and t = 6 h. n.s., * *p* < 0.05, ** *p* < 0.01, and *** *p* < 0.001. Scale bar = 250 μM.

The ability of cancer cells to cluster/aggregate together plays a vital role in metastatic progression. Therefore, to determine whether clustering was impacted by the increase in cytoplasmic Ca^2+^, cells were directly plated in a nonadherent environment with either 5 µM Iono, 2 µM Tg, or vehicle control and allowed to cluster for 6 h. Quantification of homotypic clustering efficiency was determined using a custom MATLAB script optimized to the area of a single nuclei of 75 μM^2^ [27]. Clustering efficiency was determined by the number of nuclei aggregates detected at time 0 divided by the number of nuclei aggregates at 6 h. MDA-MB-231 cells treated with 5 μM Iono showed a reduction in clustering efficiency, but do not reach statistical significance (*p* = 0.0547). However, treatment with 2 μM Tg did significantly reduce clustering efficiency (Figure 5A,B). Representative images of MDA-MB-231 cells stained with nuclear staining visualize the initial and final seeding density and clusters comparing the vehicle control group and the treatment group at the end time points (t = 0H and t = 6H) are shown in Figure 5C,D (Figure S1). In the MDA-MB-436 cells, 5 μM Iono and 2 μM Tg treatment significantly decreased clustering efficiency over the 6 h time course (*p* = 0.0053 and *p* = 0.0009) (Figure 5E,F). Images of MDA-MB-436 cells stained with Hoechst illustrate the initial and final seeding density and clusters of the treatment group and the vehicle control group at the time points t = 0H and t = 6H (Figure 5G,H and Figure S2). As previously shown, a decrease in McTN formation and the ability of cells to reattach to surfaces over time with Iono or Tg treatment, both treatments are effective in reducing the ability of detached MDA-MB-231 and MDA-MB-436 cells to cluster over time.

### 3.5. Elevated Cytoplasmic Calcium Concentration Induces Actin Contraction and Rearrangement

**Figure 6:**
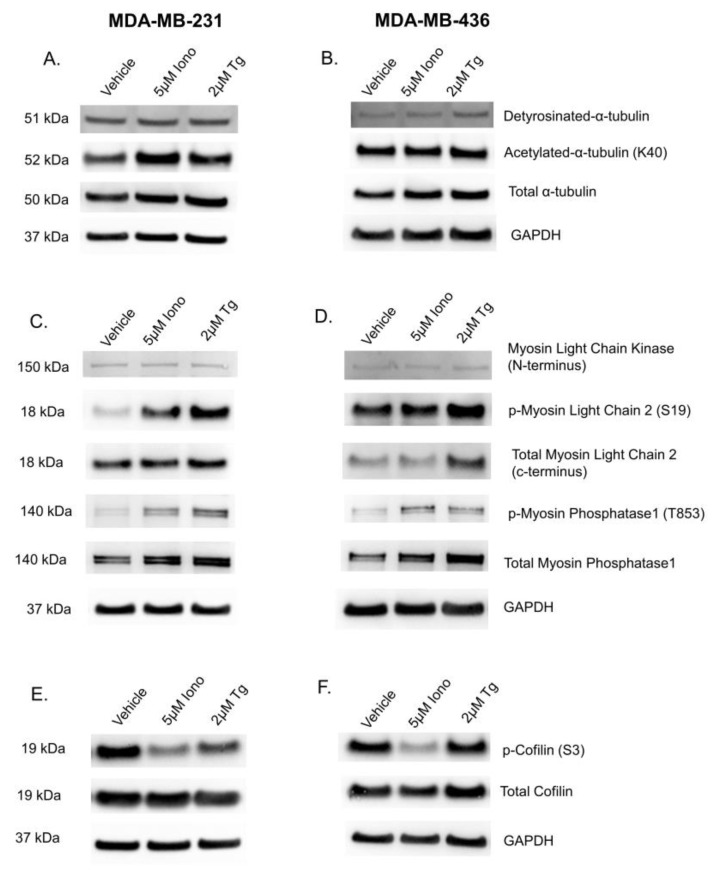
Elevation of cytoplasmic Ca^2+^ in MDA−MB−231 and MDA−MB−436 cells induces actin cortex contraction and rearrangement. (**A**) Ionomycin or Thapsigargin treatment in MDA-MB-231 cells does not affect microtubule PTMs. (**B**) MDA-MB-436 cells treated with Ionomycin or Thapsigargin does not affect microtubule PTMs. (**C**) Ionomycin or Thapsigargin in MDA-MB-231 cell-induced cytoplasmic Ca^2+^ elevation increases the phosphorylation of myosin light chain 2 at serine 19 and of myosin phosphatase1 at threonine 853, indicating cortical actin contraction. (**D**) MDA-MB-436 cells treated with either Ionomycin or Thapsigargin induce cytoplasmic Ca^2+^ elevation, which increases phosphorylation of myosin light chain 2 at serine 19 and of myosin phosphatase1 at threonine 853 indicate cortical actin contraction. (**E**) Ca^2+^ increases in MDA-MB-231 cells causes a dephosphorylation of cofilin on serine 3, resulting in the activation of cofilin. (**F**) Ionomycin treatment in MDA-MB-436 cells increase cofilin activity through an elevation of cytoplasmic Ca^2+^-mediating dephosphorylation of S3 on cofilin. (**A**–**F**) Three independent experiments were conducted and representative immunoblot images shown. All original immunoblot images can be found in the supplementary materials.

The specific mechanisms that underlie cytoplasmic Ca^2+^-mediated McTN suppression was next investigated. McTNs are microtubule-based structures that are stabilized by their post-translational modifications (PTM) of acetylation at lysine 40 and detyrosination on α-tubulin. We initially investigated whether changes in expression of these PTMs contributed to McTN suppression. Protein analysis of acetylation and detyrosination in cells treated with either 5 μM Iono or 2 μM Tg did not reach statistical significance (Figure 6A,B, Figures S3A,B and S4A,B). These results suggest the loss of tubulin PTMs known to support McTNs is not the mechanism of McTN suppression by elevated cytoplasmic Ca^2+^.

Given that the PTMs of tubulin remained unchanged by Iono or Tg treatment, we next investigated the role of the actin network. However, evidence from the literature suggests that calcium-calmodulin dependent myosin light chain kinase (MLCK) activity and expression regulates actin rearrangement. To determine whether this mechanism is conserved in breast cancer cells, we first verified the basal expression of MLCK, myosin light chain 2 (MLC2), and phospho-myosin light chain 2 (p-MLC2). We next probed for p-MLC2 at serine 19 (S19), an indicator of actin cortex contraction, to interrogate if increasing cytoplasmic Ca^2+^ stabilized the actin cortex. An initial time course was performed to determine the maximal effect seen. Five minutes of either compound treatment was sufficient to show the greatest change in expression. Immunoblot images of MDA-MB-231-treated cells with either 5 μM Iono or 2 μM Tg show an increase in phosphorylation at S19 for MLC2 in comparison to the vehicle control, however, densitometry analysis of three biological replicates do not reach statistical significance (Figure 6C and Figure S3C). Immunoblot images of MDA-MB-436 cells samples treated with either 5 μM Iono or 2 μM Tg show an increase in phosphorylation on MLC2 on S19; however, a variation of the total MLC2 between each sample within a biological replicate challenges any conclusion that can be drawn from these results (Figure 6D and Figure S4C). On the other hand, actin cortex contractility is also regulated through myosin phosphatase1 (MYPT1) activity. Phosphorylation at threonine 853 (T853) on MYPT1 is the inactive state of MYPT1 that is an additional indicator of actin cortex contraction. The immunoblot images of three biological replicates of MDA-MB-231 and MDA-MB-436 cells show a trending increase in phosphorylation of MYPT1 with Iono or Tg treatment in comparison to the vehicle, but densitometry analysis does not reach statistical significance (Figure 6C,D, Figures S3D and S4D).

Actin rearrangement was further assessed by probing for phosphorylated cofilin at serine 3 (S3). A 5 min treatment with either 5 μM Iono or 2 μM Tg consistently decreased cofilin phosphorylation in MDA-MB-231 cells (Figure 6E and Figure S3E). In MDA-MB-436 cells, only 5 μM Iono treatment was sufficient to significantly decrease cofilin phosphorylation (Figure 6F and Figure S4E).

### 3.6. Calcium-Induced Microtentacle Suppression Requires Actin Polymerization

**Figure 7:**
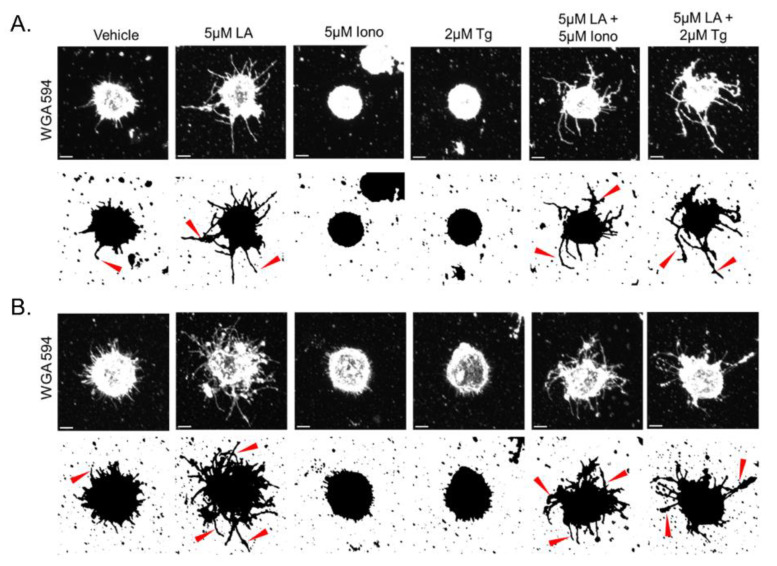

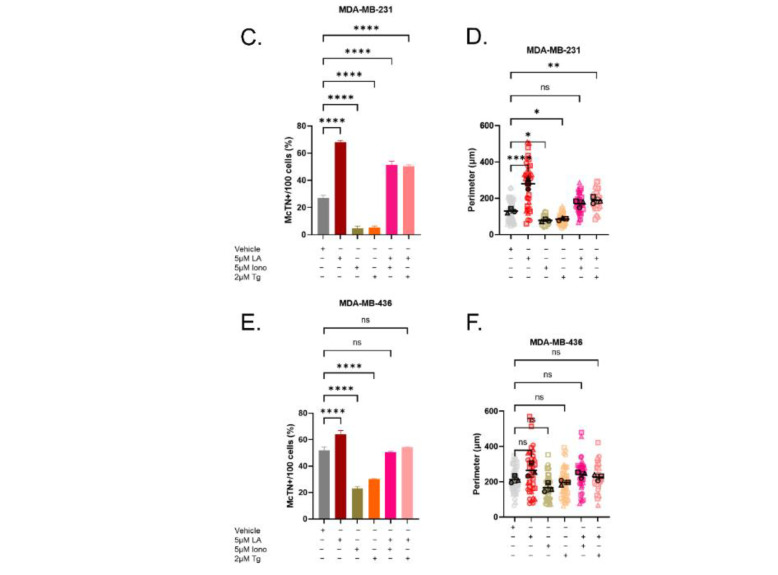
Inhibition of actin polymerization abrogates Ca^2+^-mediated microtentacle suppression in MDA−MB−231 and MDA−MB−436 cells. (**A**,**B**) Representative maximum intensity single cell confocal images (wheat germ agglutinin (WGA) 594 (1:100)) and their black and white contrast counterparts illustrate MDA-MB 231 and MDA-MB-436 cells initially treated with vehicle (0.5% DMSO), 5 µM Latrunculin A, 5 µM Ionomycin, 2 µM Thapsigargin, or the combination of 5 µM Latrunculin A with 5 µM Ionomycin or 2 µM Thapsigargin. Arrows indicating microtentacles on the cell. Scale bar = 5 μM. (**A**) Images of MDA-MB-231 cells illustrate enhanced McTN formation with 5 μM Latrunculin A alone or the combination of 5 µM Latrunculin A with 5 µM Ionomycin or 2 µM Thapsigargin. (**B**) Representative images of MDA-MB-436 cells treated with 5 μM Latrunculin A alone or the combination of 5 µM Latrunculin A with 5 µM Ionomycin or 2 µM Thapsigargin show an elevation of McTN formation. (**C**) Inhibition of actin polymerization alone significantly increases McTN positivity, while the addition Ca^2+^ flux by Ionomycin or Thapsigargin under this condition also impedes McTN suppression in MDA-MB-231 cells. (**D**) Whole cell perimeter of MDA-MB-231 cells increases with 5 µM Latrunculin A treatment alone and with the elevation of cytoplasmic Ca^2+^ from 5 µM Ionomycin and 2 µM Thapsigargin treatment. (**E**) Inhibition of actin polymerization in MDA-MB-436 cells with 5 μM Latrunculin A significantly increases McTN positivity alone and in the presence of Ca^2+^ flux by Ionomycin or Thapsigargin treatment. (**F**) Whole cell perimeter in MDA-MB-436 cells does not significantly change when treated with 5 μM Latrunculin A alone and with the elevation of cytoplasmic Ca^2+^ from 5 µM Ionomycin or 2 µM Thapsigargin treatment. (**C**,**E**) Data shown as mean ± SD, *n* = 3. (**D**,**F**) Data shown as mean ± SD, *n* = 3, determined from a total of 20 to 30 cells from combined biological replicates. n.s., * *p* < 0.05, ** *p* < 0.01, and **** *p* < 0.0001.

Given the rapid phosphorylation events regulating actin turnover observed in the immunoblot analysis, we next assessed the necessity of filamentous actin polymerization for Ca^2+^-induced McTN suppression. Latrunculin A (LA), an actin depolymerizing agent known to increase McTN formation, was used to inhibit actin polymerization before elevating cytoplasmic Ca^2+^ with either 5 μM Iono or 2 μM Tg. As previously described, representative single cell confocal images of 5 μM LA-treated MDA-MB-231 and MDA-MB-436 cells show the McTN phenotype, while cells treated with only 5 μM Iono or 2 μM Tg lack McTNs (Figure 7A,B). However, when detached and suspended MDA-MB-231, and MDA-MB-436 cells were initially pretreated with 5 μM LA for 15 min before the addition of 5 μM Iono or 2 μM Tg, cell retained the McTN phenotype (Figure 7B).

Quantitation of cell positivity for McTNs shows a significant increase in cell positivity for MDA-MB-231 and MDA-MB-436 cells treated with 5 μM LA compared to the vehicle (Figure 7C,E). The results from Figure 1 were independently reproduced to show that an elevation of cytoplasmic Ca^2+^ with 5 μM Iono or 2 μM Tg treatment in both cell lines significantly decreased cell positivity for McTNs in compared to the vehicle. In contrast, inhibition of actin polymerization with the induced elevation of cytoplasmic Ca^2+^ significantly increases cell positivity for McTNs compared to the vehicle in both cell lines (Figure 7C,E). Orthogonal whole cell perimeter measurements from confocal images showed MDA-MB-231 cells treated with 5 μM LA have a significantly larger perimeter than the vehicle (Figure 7D). 5 μM Iono or 2 μM Tg treatment in the MDA-MB-231 cells also duplicated previous results showing a significant decrease in the perimeter compared to the vehicle (Figure 7D). MDA-MB-231 cells initially treated with 5 μM LA before Ca^2+^ flux stimulation by either 5 μM Iono or 2 μM Tg increased whole cell perimeter, but only 2 μM Tg treatment after 5 μM LA pretreatment achieved statistical significance (Figure 7D). The overall results for whole cell perimeter measurements in the MDA-MB-436 cells trended in the same directions under each condition but did not reach statistical significance (Figure 7F).

## 4. Discussion

The impact of acute Ca^2+^-mediated signaling pathways is well established in many organ systems and cell types, however, its role in tumor biology remains a knowledge gap. Early work using two-dimensional adherent cell culture models showed that chemical and mechanical-induced rapid Ca^2+^ signaling differs between breast epithelial and breast cancer cells [19,20,21], suggesting dysregulation of acute Ca^2+^ signaling mechanisms in cancer. These studies generated new questions about the contributions of acute Ca^2+^ to cancer cell morphologies and phenotypes. Of notable interest is the role of Ca^2+^ signaling in the dynamic arrangement of actin and tubulin in the nonadherent environment that can produce the McTN metastatic phenotype.

Currently, little is known about the physiological relevance of Ca^2+^ signaling on the cytoskeleton in nonadherent models. Our lab has previously demonstrated the mechanisms of McTN formation through the examination of tubulin and actin dynamics in breast cancer cells in a nonadherent state. We have shown an increased frequency of McTNs through actin depolymerization with Cytochalasin-D or Latrunculin A, while McTN frequency decreased after treatment with tubulin depolymerizers such as Colchicine or Vinorelbine [7,27]. Additionally, our lab has shown inhibition of the upstream effectors of actomyosin contractility, such as Rho-associated kinase (ROCK), destabilizes the actin cortex, and increases the formation of microtubule based McTNs [28]. This current work shows that treatment of Iono or Tg induces an elevation of cytoplasmic Ca^2+^ (Figure 1 and Figure 2) and suppresses McTN formation in the presence of extracellular Ca^2+^ (Figure 3). Additionally, the ablation of extracellular Ca^2+^ alone with a Ca^2+^ chelator leads to an enhancement of McTN levels, while the co-treatment of compound and zero Ca^2+^ conditions shows no difference (Figure 3). The data may suggest that the presence or absence of McTN relies on a necessary balance of spatiotemporal Ca^2+^ signaling and provides an intriguing avenue for future studies.

Cytoskeletal plasticity and coordinated remodeling play an essential role in metastatic dissemination and progression. Actin filaments are composed of monomeric globular subunits, which make up part of the cellular cytoskeleton, and is responsible for maintaining cellular morphology [2,3]. Maintenance of the actin cytoskeleton is a highly dynamic process modulated by rapid signaling cascades and the rapid recruitment of accessory proteins to mediate the organization, polymerization, and depolymerization of filamentous actin from pools of globular actin and vice versa [2,3].

Research within the muscle field has established a conserved signaling pathway by which Ca^2+^ signaling can induce cellular and actin contraction [11,31,32]. The conserved mechanism of cellular and actin contraction via Ca^2+^ signaling in epithelial cells is through non-muscle myosin II. The role of non-muscle myosin II is primarily regulated via the phosphorylation of S19 MLC2 by MLCK in a calcium-calmodulin-dependent manner [33]. MLCK-dependent phosphorylation of MLC2 leads to an unfolding of non-muscle-myosin II and a 1000-fold increase in ATPase activity that promotes motor activity on actin filaments and contraction of the actin cortex [34]. Recently, Li et al., further elucidated how the actomyosin cortex regulates McTNs by showing dominant negative MLC2 promotes McTNs, and constitutively active MLC2 suppresses McTNs [4]. Furthermore, MYPT1 dephosphorylates MLC2, resulting in the relaxation of the actin cortex [28,35]. Investigation into the regulation of the actomyosin cortex was also examined through cofilin activity. Cofilin plays an essential role in tumor cell motility and has been shown to regulate McTN formation [3,36]. Our immunoblot data show increases in phosphorylated MLC2 at S19 and MYPT1 at T853, indicating contraction of the actin cortex, with simultaneous decreases in phosphorylated cofilin to increase actin severing and rearrangement through cofilin activity (Figure 6). Additionally, inhibition of actin polymerization prior to the elevation of cytoplasmic Ca^2+^ abrogates Ca^2+^-mediated McTN suppression (Figure 7). The summation of these phenotypic observations and immunoblot analysis indicates dynamic actin polymerization and turnover are necessary for Ca^2+^-mediated McTN suppression. These results suggest a concurrent molecular mechanism between actin contraction and dynamic actin polymerization and depolymerization for Ca^2+^-mediated McTN suppression. The dual molecular roles of myosin light chain II and cofilin support the nonlinear mechanical response of individual actin filaments contracting and buckling, which drive cortical actomyosin contractility and polymerization dynamics [37]. Ca^2+^-dependent activation of myosin light chain kinase stimulates actin contraction while concurrently, cofilin activation mediates filamentous actin depolymerization to suppress McTNs over an acute time course [38,39,40,41].

While cytoplasmic Ca^2+^ entry from either internal or external Ca^2+^ sources gives rise to similar phenotypic suppression of McTNs (Figure 3), further scrutiny into reattachment at later time points in the 24 h time frame begins to yield observable differences in cellular spreading (Figure 4). From previous live cell time course imaging observations and xCelligence reattachment data, the initial attachment was estimated to begin immediately after seeding to around 5 h post seeding, while cellular spreading was visualized as early as 4 h over the 24 h time course [27]. While Iono and Tg treatments acutely decrease the McTN phenotype, these observations do not correlate with the hypothesis of a decrease in electrical impedance reattachment trend from initial seeding to post 5 h after seeding compared to the vehicle control (Figure 4). However, visualization of cellular spreading at 8 h and 16 h illustrated Iono-treated cells have similar cellular spreading patterns to the bottom of a tissue culture plate to the vehicle control, while Tg treatment revealed a decrease in cellular spreading at 8 h and 16 h compared to the vehicle control (Figure 4). These observed differences in the cellular spreading are potentially a result of differential signaling cascades based on the entrance of extracellular Ca^2+^ or the release of intracellular Ca^2+^ stores. Different genetic backgrounds between cell lines can account for differential functional responses. Yet, functional differences observed within a cell line suggest the initiation of distinct signaling cascades that are dependent on the location of Ca^2+^ entrance (Figure 4). For example, extracellular Ca^2+^ can increase migration and invasion preferentially to the bone through Ca^2+^-mediated cytoskeletal rearrangement in breast cancer cells [42,43]. Furthermore, sustained extracellular Ca^2+^ signaling can additionally cross talk with other oncogenic signaling pathways to promote cell survival, migration, invasion, and enhanced epithelial–mesenchymal transition (EMT) [33,44,45,46]. In contrast, Ca^2+^ signaling through the release of internal stores into the cytoplasm can trigger different signaling cascades some of which result in apoptosis or autophagy over longer time periods, i.e., 48 to 72 h to days or weeks [23,47,48]. While cell death, tumor growth and migration and invasion are established phenotypes and experimental endpoints in the study of metastatic progression and tumor biology, their measurements are not within the time frame of this study.

Targeting CTCs that have shed from the primary tumor remains challenging. These CTCs that aggregate into homotypic and heterotypic clusters in circulation have increased metastatic potential [49,50,51]. Clustering together allows CTCs to survive the hostile environment of shear forces within the circulatory and lymphatic systems to metastasize to distal regions within the body [50]. A recent study in animal models showed cancer cells treatment with Ouabain or Digitoxin, cardiac glycosides, decreased CTC clustering through increasing intracellular Ca^2+^ levels, and disruption of cell–cell junctions [49]. Furthermore, inhibiting McTNs with the microtubule depolymerizer Vinorelbine was also recently shown to reduce homotypic McTN-mediated clustering and significantly delayed lung metastasis in mouse models [27]. Our results provide additional evidence linking these previous phenomena by demonstrating the utility of increasing cytoplasmic Ca^2+^ to decreases McTN-mediated clustering over a short time course, i.e., 6 h (Figure 5). Drugs that induce this rapid change of free-floating cancer cell morphology could be leveraged as possible adjuvant treatments immediately post-surgery to reduce the metastatic potential of tumor cells that have shed into the blood stream [52].

Current Ca^2+^-mediating therapies, such as Digitoxin, used in the clinic for the treatment of cardiac disease have shown promise in vitro studies to have a synergistic cytotoxic effect when used in combination with chemotherapeutic agents such as Paclitaxel [23,53]. Tg is a known cytotoxic agent that has demonstrated potential as both a single and combination anticancer agent in vitro [23,47,53]. The results from phase 1 and 2 clinical trials of its prodrug derivative, Mipsigargin, for metastatic disease have shown a favorable pharmacokinetic profile, with dosages that are well tolerated by patients and have been shown to prolong disease stabilization [38,39]. It was also observed in patients with advanced hepatocellular carcinoma who had progressed from sorafenib treatment, Mipsigargin treatment reduced blood flow to hepatic lesions [39]. The results of our study in conjunction with the clinical trial results highlight the potential utility of reducing the metastatic potential of circulating tumor cells through an alternative cytoskeletal mechanism of action.

Close examination of the acute effects of treatment are often overlooked due to a lack of a distinct change in phenotype, i.e., change in tumor size. In cancer biology, we often use the reduction of tumor size as a common endpoint measurement in animal and human studies to determine whether experimental treatments are effective. However, this reduction in primary tumor size occurs days to weeks after treatment is administered, leaving an overlooked time frame immediately following the administration of treatment. The primary tumor is estimated to shed 3.2 × 10^6^ cells per gram of tumor tissue per day with the majority of cells quickly dying [31,51]. An increasing number of published studies highlight the elevation of the dissemination of cancer cells from the primary tumor after insults to primary tumors such as tumor biopsies and surgical or pharmaceutical interventions such as neoadjuvant chemotherapy [32,34,37,40,41,52,54]. The emerging data suggest targeting these subpopulations with current chemotherapies, such as Paclitaxel, can impact their metastatic potential. Previous high throughput screening and global gene analysis of selective inhibitors targeting breast cancer stem cells identified HMLER breast cancer cells treated with Paclitaxel have enriched expression of cancer stem cell genes [5,55]. Karaginannis and colleagues later showed patient-derived xenografts treated with Paclitaxel that demonstrated pro-metastatic changes within the tumor microenvironment of metastasis, which increased the dissemination and intravasation of cancer cells [56]. Currently, the monitoring of CTCs serves as a prognostic biomarker in cancer treatments. They are used to determine the efficacy of treatment or disease progression by measuring increases or decreases in CTCs found in the patient’s blood samples over time [57]. However, these enumeration studies rarely examination of the phenotype or morphology of each CTC at the time of collection. The short-term impacts of drug treatment on CTC enumeration and phenotypes highlight the gap in the knowledge of molecular mechanisms in this subpopulation and emphasize the importance of appropriate time frames for the use of pharmaceuticals in both adjuvant and neoadjuvant settings. By identifying and understanding the various phenotypes and molecular targets at specific time points within the metastatic cascade will lead to the development of personalized treatments.

## 5. Conclusions

The complex 3-dimensional environment that CTCs survive encompasses a vast array of factors acting on the cell, including fluid shear stress mechanical signals, other circulating cell types, and various soluble signaling factors. Recent studies have begun to elucidate CTC sensitivity to fluid shear stress and have suggested increased CTC stiffness leads to an increase in cell death [51,58,59]. Our current data supports clinical trial data indicating a rationale for the use of Ca^2+^ modulators as a potential therapeutic strategy for preventing metastasis [38,39]. Our study shows proof-of-principle that viability-independent transient increases in cytoplasmic Ca^2+^ with Iono or Tg yield rapid morphological changes to tumor cells in suspension that reflect a less advantageous phenotype for metastatic behaviors.

## Data Availability

The data will be freely provided upon request from the corresponding author.

## Supplementary Materials

The following supporting information can be downloaded at: https://www.mdpi.com/article/10.3390/cancers15030884/s1: Figure S1: MDA-MB-231 cells treated with Ionomycin or Thapsigargin decreases homotypic cellular aggregation. Figure S2: Ionomycin or Thapsigargin treatment decreases homotypic cellular clustering in MDA-MB-436 cells. Figure S3: Densitometry results of MDA-MB-231 cells treated with the vehicle, Ionomycin, or Thapsigargin. Figure S4: Densitometry results of MDA-MB-436 cells treated with the vehicle, Ionomycin, or Thapsigargin. File S1: All original immunoblot images. Method S1: Chemiluminescence and Molecular Weights: Using the iBright Software.
