# Supplementary material for: Elevation of Cytoplasmic Calcium Suppresses Microtentacle Formation and Function in Breast Tumor Cells
Source: Cancers (Basel). 2023 Jan 31;15(3):884. doi: 10.3390/cancers15030884 (PMC9913253; doi:10.3390/cancers15030884)
Supplement: Supplementary file 1 [file cancers-15-00884-s001.zip › cancers-2080686-Supplementary/Chang etal Supplemental Figures and Figure Legends.pdf]

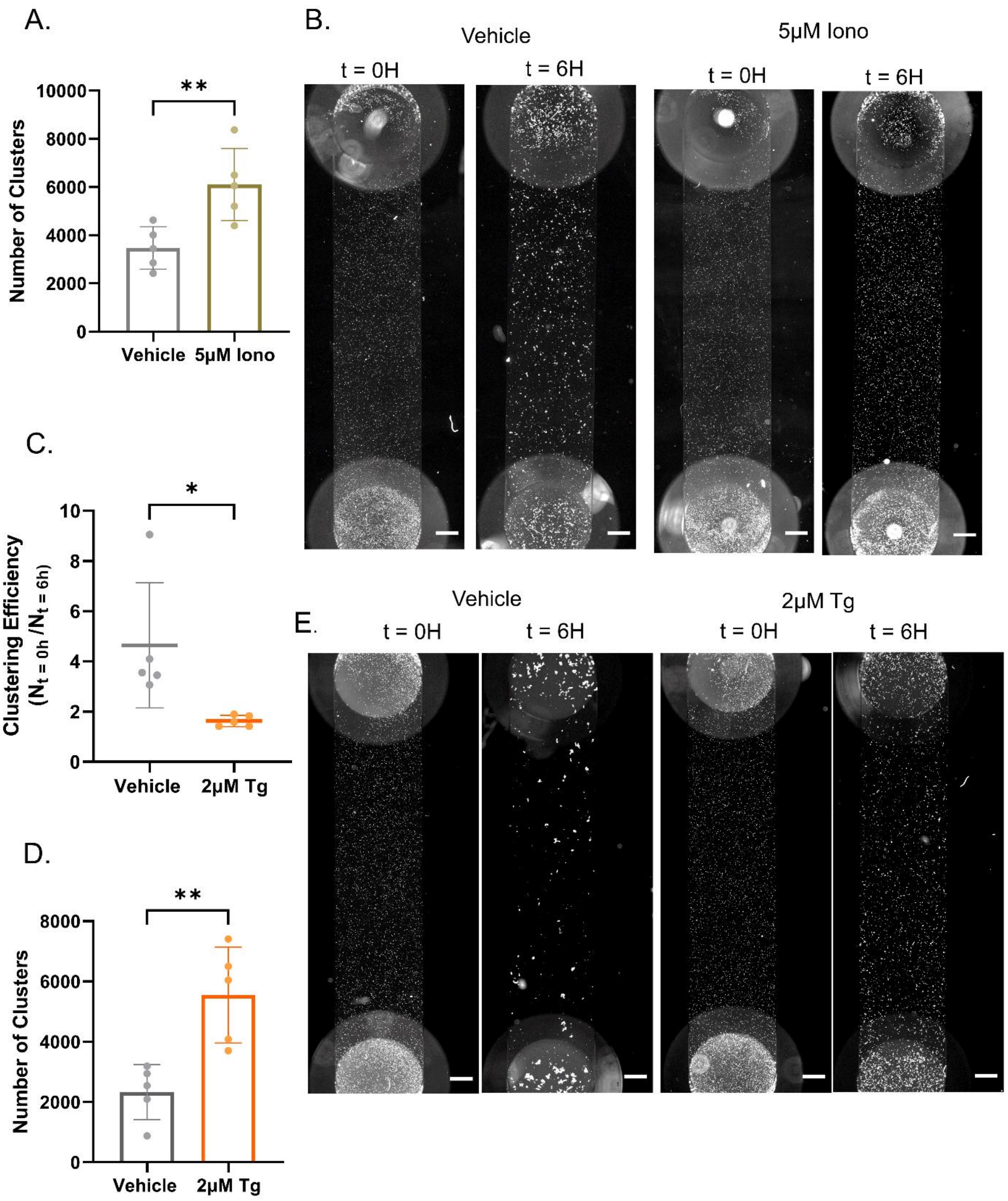

Supplemental Figure 1: MDA-MB-231 cells treated with Ionomycin or Thapsgargin decreases homotypic cellular aggregation. A) At terminal t = 6 hours, Ionomycin

treatment increases the number of clusters detected. **B)** Representative Hoechst stained images show the full region of interest of the Ibidi slide channel from Figure 5 panel C. **C)** 2μM Thapsigargin 6-hour treatment significantly decreases clustering efficiency. Data are shown as mean $\pm$ SD, n = 5; a fifth biological replicate was identified as an outlier with the ROUT test with a Q = 1% was included. **D)** Thapsigargin treatment increases the number of clusters detected at t = 6 hours. **E)** Representative Hoechst stained images show the full region of interest of the Ibidi slide channel of Figure 5 panel D. (A,C,D) Data shown as mean $\pm$ SD of 5 independently conducted experiments. *p<0.05, **p<0.01. (B and E) Scale bar = 1000μm.

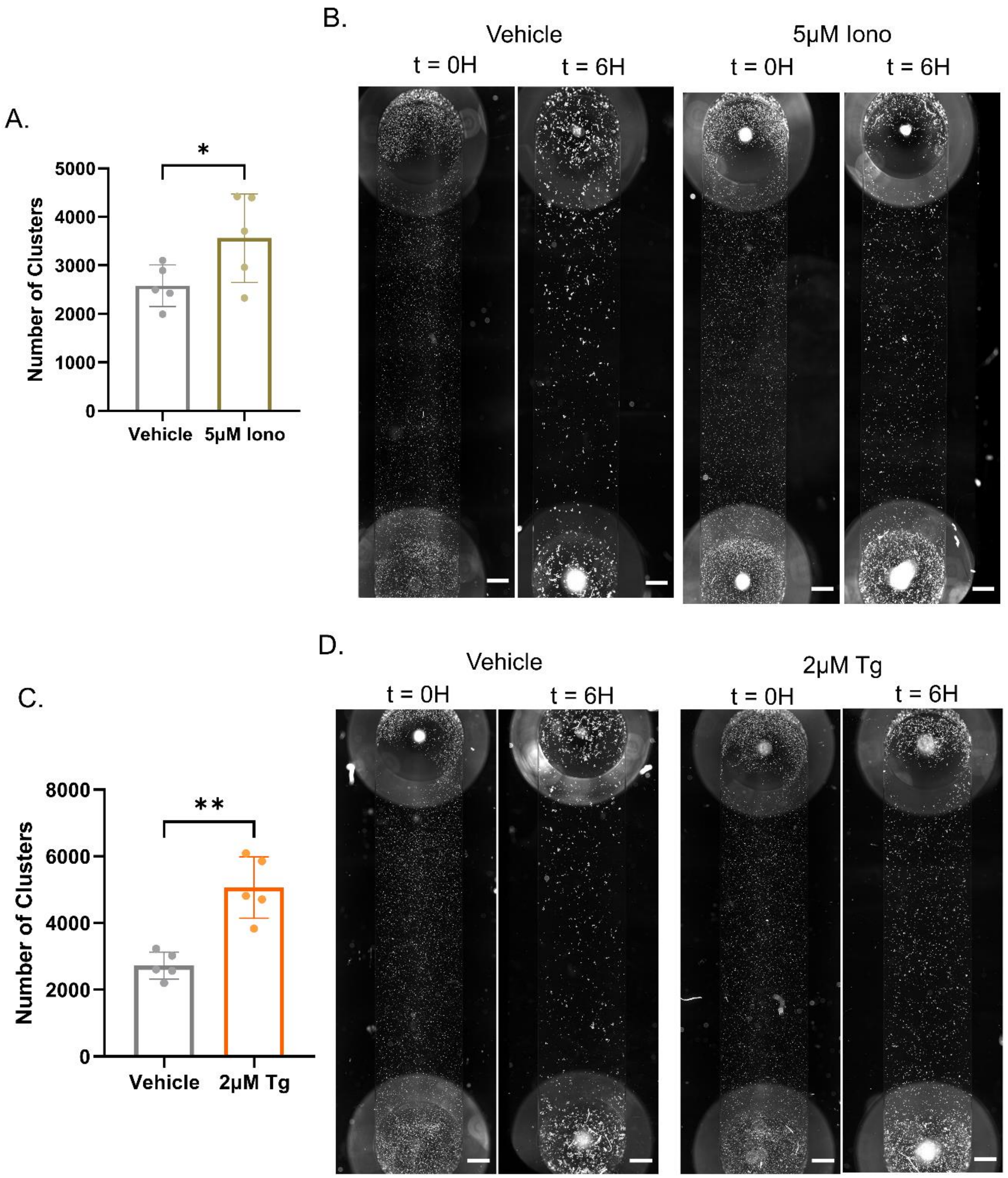

Supplemental Figure 2: Ionomycin or Thapsigargin treatment decreases homotypic cellular clustering in MDA-MB 436 cells. A) Ionomycin treatment

increases the number of clusters detected in comparison to the vehicle. **B)** Representative Hoechst stained images show the full ROI of the Ibidi slide channel of Figure 5 panel G. **C)** Thapsigargin treatment increases the number of clusters detected in comparison to the vehicle control. **D)** Representative Hoechst stained images show the full ROI of the Ibidi slide channel of Figure 5 panel H. (A and C) Data shown as mean $\pm$ SD of 5 independently conducted experiments. *$p<0.05$, **$p<0.01$. (B and D) Scale bar = 1000μm.

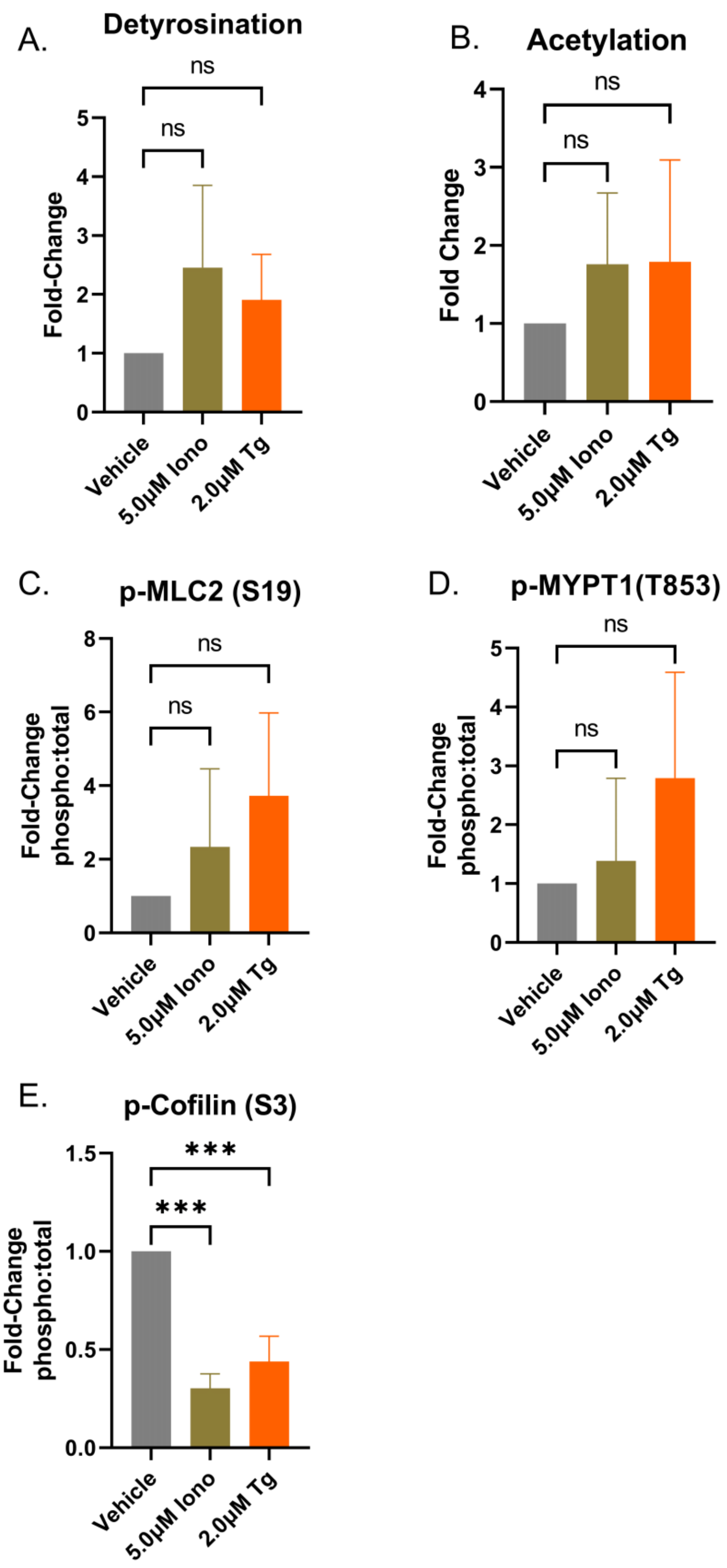
A.
Detyrosination
ns
ns
Fold-Change
0
1
2
3
4
5
Vehicle
5.0μM Iono
2.0μM Tg
B.
Acetylation
ns
ns
Fold Change
0
1
2
3
4
Vehicle
5.0μM Iono
2.0μM Tg
C.
p-MLC2 (S19)
ns
ns
Fold-Change
phospho:total
0
2
4
6
8
Vehicle
5.0μM Iono
2.0μM Tg
D.
p-MYPT1(T853)
ns
ns
Fold-Change
phospho:total
0
1
2
3
4
5
Vehicle
5.0μM Iono
2.0μM Tg
E.
p-Cofilin (S3)
***
***
Fold-Change
phospho:total
0.0
0.5
1.0
1.5
Vehicle
5.0μM Iono
2.0μM Tg

Supplemental Figure 3: Densitometry results of MDA-MB-231 cells treated with vehicle, Ionomycin or Thapsigargin. A) The ratio of detyrosinated (deTyr) tubulin to alpha tubulin was taken and cells treated with vehicle was set to 1. B) The ratio of acetylated-α-tubulin (K40) (Acetyl) to alpha tubulin was taken and cells treated with vehicle was set to 1. C) The ratio of phosphorylated-myosin light chain (S19) to total myosin light chain was taken and cells treated with vehicle was set to 1. D) The ratio of phosphorylated myosin phosphatase1 (T853) to total myosin phosphotase1 was taken and cells treated with vehicle was set to 1. E) The ratio of phosphorylated-cofilin (S3) to total cofilin was taken and cells treated with vehicle was set to 1. (A-E) Data shown as mean $\pm$ SD of 3 independently conducted experiments. Bands were initially normalized to the GAPDH loading control. n.s., ***p<0.001.

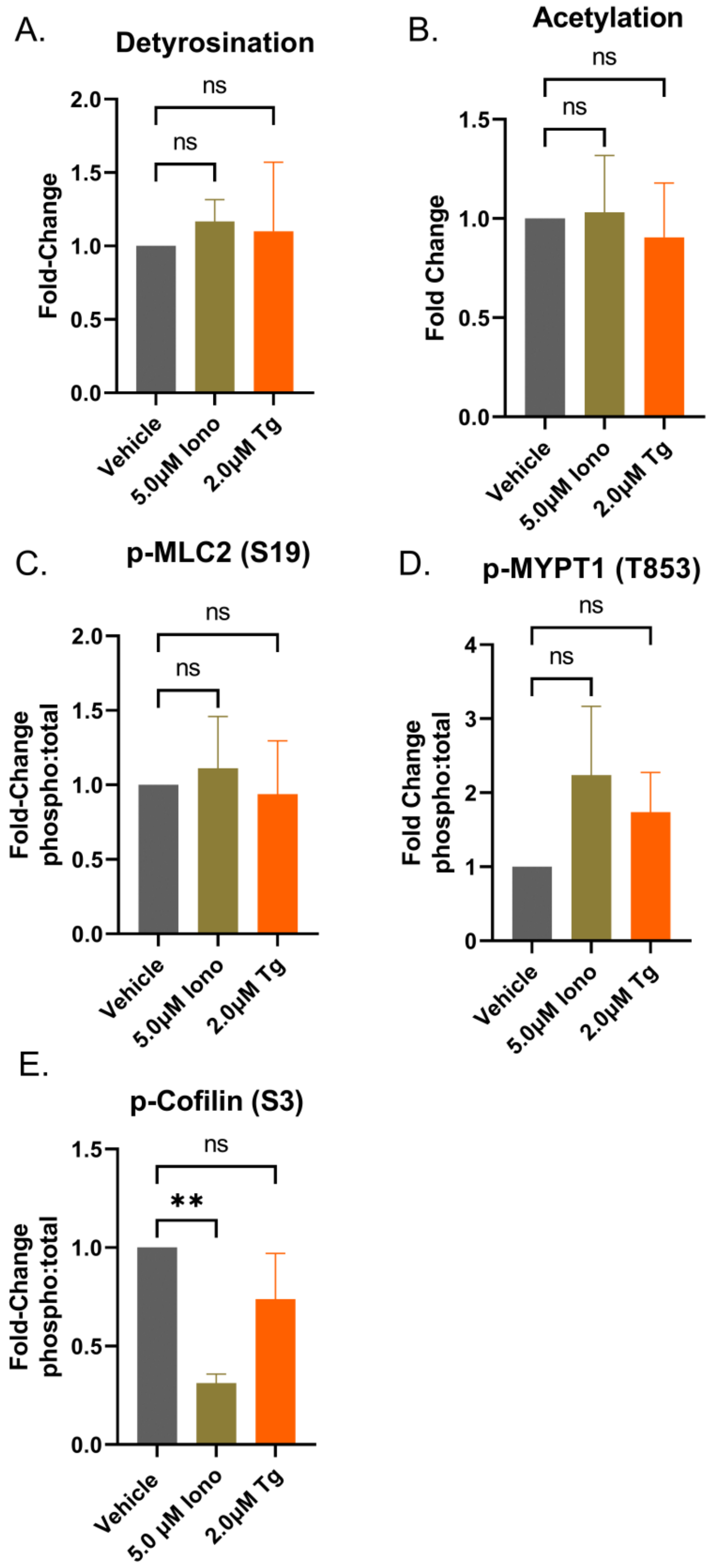
A.
Detyrosination
Fold-Change
2.0
1.5
1.0
0.5
0.0
ns
ns
Vehicle
5.0µM Iono
2.0µM Tg
B.
Acetylation
Fold Change
1.5
1.0
0.5
0.0
ns
ns
Vehicle
5.0µM Iono
2.0µM Tg
C.
p-MLC2 (S19)
Fold-Change
phospho:total
2.0
1.5
1.0
0.5
0.0
ns
ns
Vehicle
5.0µM Iono
2.0µM Tg
D.
p-MYPT1 (T853)
Fold Change
phospho:total
4
3
2
1
0
ns
ns
Vehicle
5.0µM Iono
2.0µM Tg
E.
p-Cofilin (S3)
Fold-Change
phospho:total
1.5
1.0
0.5
0.0
ns
**
Vehicle
5.0 µM Iono
2.0µM Tg

Supplemental Figure 4: Densitometry results of MDA-MB-436 cells treated with vehicle, Ionomycin or Thapsigargin. A) The ratio detyrosinated (detyr) tubulin to alpha tubulin was taken and cells treated with vehicle was set to 1. B) The ratio of acetylated-α-tubulin (K40) (Acetyl) to alpha tubulin was taken and cells treated with vehicle was set to 1. C) The ratio of phosphorylated myosin light chain (S19) to total myosin light chain was taken and cells treated with vehicle was set to 1. D) The ratio of phosphorylated myosin phosphatase1 (T853) to total myosin phosphotase1 was taken and cells treated with vehicle was set to 1. E) The ratio of phosphorylated cofilin (S3) to total cofilin was taken and cells treated with vehicle was set to 1. (A-E) Data shown as mean $\pm$ SD of 3 independently conducted experiments. Bands were initially normalized to the GAPDH loading control. n.s., **$p<0.01$.
