# Supplementary material for: Elevation of Cytoplasmic Calcium Suppresses Microtentacle Formation and Function in Breast Tumor Cells
Source: Cancers (Basel). 2023 Jan 31;15(3):884. doi: 10.3390/cancers15030884 (PMC9913253; doi:10.3390/cancers15030884)
Supplement: Supplementary file 1 [file cancers-15-00884-s001.zip › cancers-2080686-Supplementary/File S1_ Original Blots/Original Immunoblot Images MDAMB231 Biological Replicate 1.pdf]

iBright™ Image Analysis Report

Katarina+ Chang

18 November 2022

GAPDH_CHEMI_10122021_113734

Date:	12 October 2021 11:37:34AM
Mode:	Chemi Blots
Notes:	
Model:	FL1500
Instrument name:	2462619090234
Serial No:	2462619090234
Firmware version:	1.6.0
iBA version:	5.0
Image size:	676px X 540px
Image area:	118.63mm X 94.91mm
Optical Zoom:	1.9x
Digital Zoom:	1x
Focus level:	430
Resolution:	5 x 5
Exposure time:	150000 ms
Exposure mode:	Normal

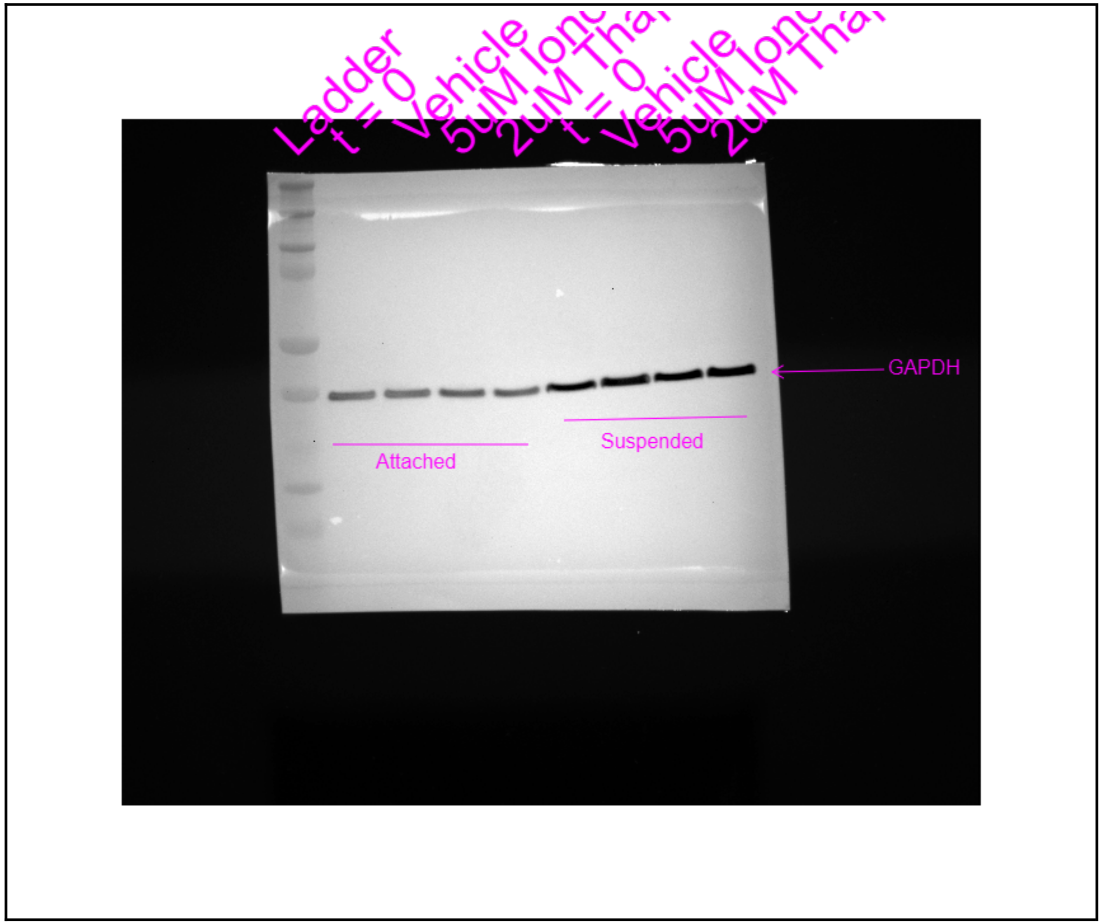

GAPDH_CHEMI_10122021_113734

Date:	12 October 2021 11:37:34AM
Mode:	Chemi Blots
Notes:	
Model:	FL1500
Instrument name:	2462619090234
Serial No:	2462619090234
Firmware version:	1.6.0
iBA version:	5.0
Image size:	676px X 540px
Image area:	118.63mm X 94.91mm
Optical Zoom:	1.9x
Digital Zoom:	1x
Focus level:	430
Resolution:	5 x 5
Exposure time:	150000 ms
Exposure mode:	Normal

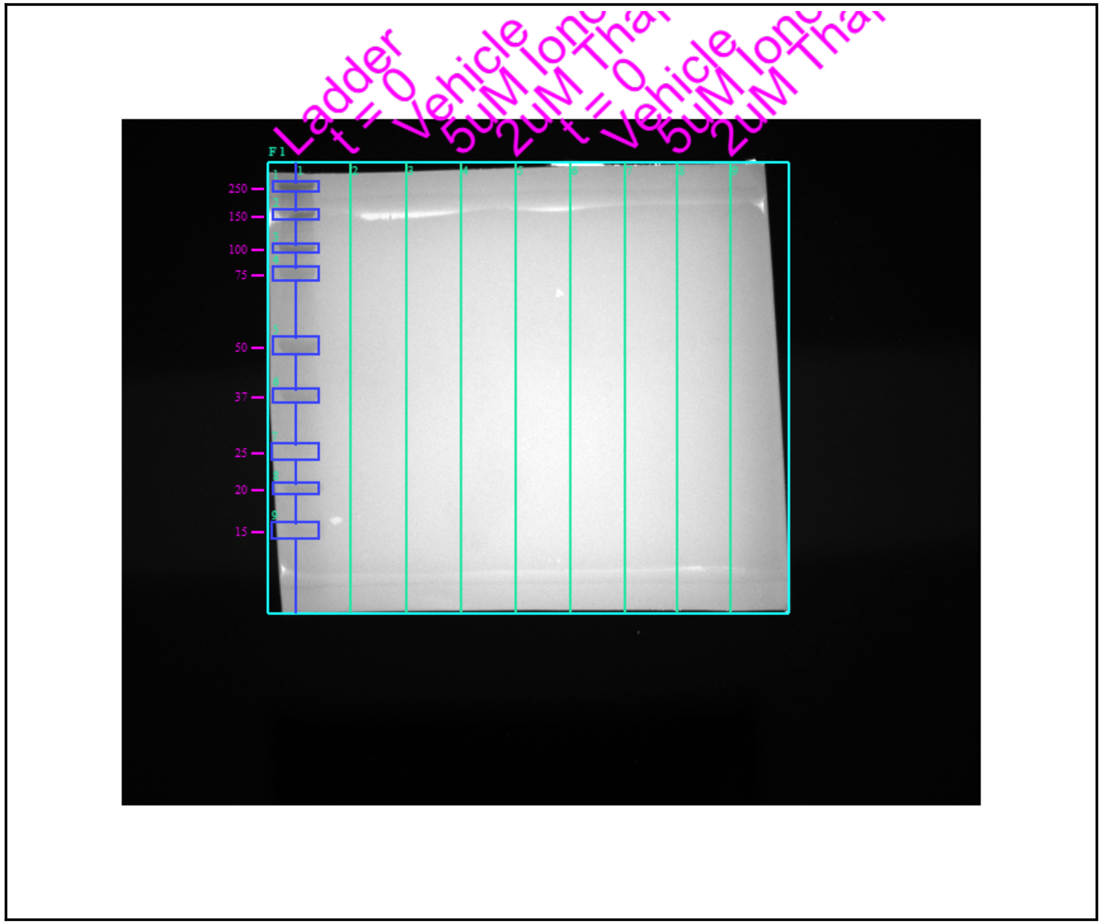

GAPDH_CHEMI_10122021_113734

Date:	12 October 2021 11:37:34AM
Mode:	Chemi Blots
Notes:	
Model:	FL1500
Instrument name:	2462619090234
Serial No:	2462619090234
Firmware version:	1.6.0
iBA version:	5.0
Image size:	676px X 540px
Image area:	118.63mm X 94.91mm
Optical Zoom:	1.9x
Digital Zoom:	1x
Focus level:	430
Resolution:	5 x 5
Exposure time:	150000 ms
Exposure mode:	Normal

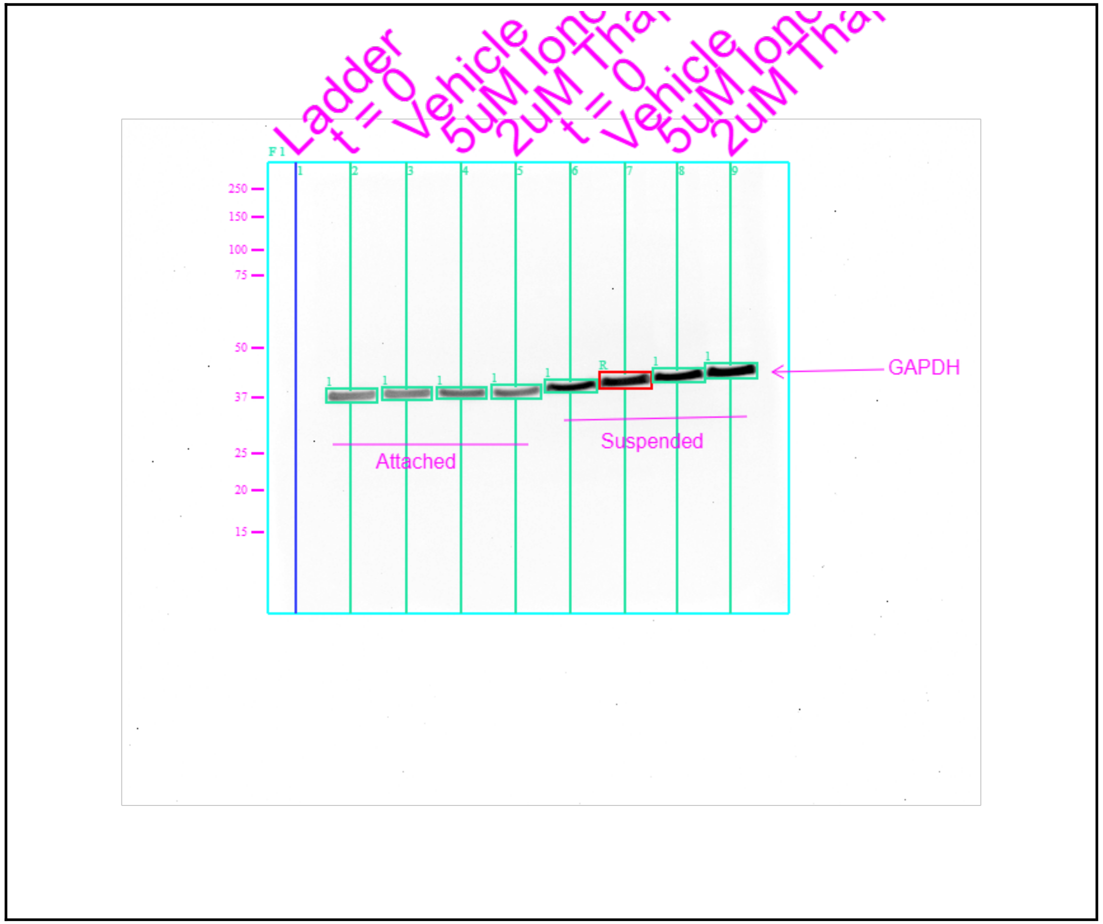

LANE AND BAND ANALYSIS DATA TABLE

GAPDH_CHEMI_10122021_113734

Frame: 1
Channel: Membrane
Sensitivity: 100
Molecular Weight Analysis Regression Method : Point to Point

Lane 1 - Ladder

#	Vol. (Int.)	Local Bg. Corr. Vol.	Area	Rf	Density	Local Bg. Corr. Den.	% band purity	% lane purity	Mol. Wt.
1	13,805,636	1,198,305	333	0.054	41,458	3,598.514	18.734	3.107	250
2	12,131,087	1,159,346	333	0.115	36,429	3,481.522	18.125	2.73	150
3	11,044,395	1,009,679	296	0.189	37,312	3,411.081	15.785	2.486	100
4	15,335,750	551,595	444	0.245	34,539	1,242.333	8.624	3.451	75
5	18,930,499	572,715	555	0.406	34,109	1,031.92	8.954	4.261	50
6	14,842,192	292,520	444	0.515	33,428	658.83	4.573	3.34	37
7	16,651,437	167,575	532	0.639	31,299	314.991	2.62	3.748	25
8	12,450,146	241,306	370	0.721	33,649	652.181	3.773	2.802	20
9	18,614,089	1,203,267	532	0.814	34,988	2,261.781	18.812	4.189	15

Frame: 1
Channel: Chemi
Sensitivity: 100
Molecular Weight Analysis Regression Method : Point to Point

Lane 2 - t = 0

#	Vol. (Int.)	Local Bg. Corr. Vol.	Area	Rf	Density	Local Bg. Corr. Den.	% band purity	% lane purity	Mol. Wt.	Rel. Quant. (w/ LB Corr. Vol.)
1	6,202,236	5,365,774	492	0.515	12,606	10,906	100	40.662	37	0.535

Lane 3 - Vehicle

#	Vol. (Int.)	Local Bg. Corr. Vol.	Area	Rf	Density	Local Bg. Corr. Den.	% band purity	% lane purity	Mol. Wt.	Rel. Quant. (w/ LB Corr. Vol.)
1	5,952,011	5,012,179	440	0.513	13,527	11,391	100	35.788	37.333	0.5

Lane 4 - 5uM Ionomycin

#	Vol. (Int.)	Local Bg. Corr. Vol.	Area	Rf	Density	Local Bg. Corr. Den.	% band purity	% lane purity	Mol. Wt.	Rel. Quant. (w/ LB Corr. Vol.)

#	Vol. (Int.)	Local Bg. Corr. Vol.	Area	Rf	Density	Local Bg. Corr. Den.	% band purity	% lane purity	Mol. Wt.	Rel. Quant. (w/ LB Corr. Vol.)
1	6,267,556	5,311,180	400	0.51	15,668	13,277	100	35.142	37.667	0.53

Lane 5 - 2uM Thapsigargin

#	Vol. (Int.)	Local Bg. Corr. Vol.	Area	Rf	Density	Local Bg. Corr. Den.	% band purity	% lane purity	Mol. Wt.	Rel. Quant. (w/ LB Corr. Vol.)
1	6,272,120	4,988,143	480	0.507	13,066	10,391	100	35.077	38	0.498

Lane 6 - t = 0

#	Vol. (Int.)	Local Bg. Corr. Vol.	Area	Rf	Density	Local Bg. Corr. Den.	% band purity	% lane purity	Mol. Wt.	Rel. Quant. (w/ LB Corr. Vol.)
1	10,553,415	8,835,597	462	0.496	22,842	19,124	100	47.371	39.333	0.882

Lane 7 - Vehicle

#	Vol. (Int.)	Local Bg. Corr. Vol.	Area	Rf	Density	Local Bg. Corr. Den.	% band purity	% lane purity	Mol. Wt.	Rel. Quant. (w/ LB Corr. Vol.)
1	12,688,582	10,021,512	588	0.482	21,579	17,043	100	51.544	41	1

Lane 8 - 5uM Ionomycin

#	Vol. (Int.)	Local Bg. Corr. Vol.	Area	Rf	Density	Local Bg. Corr. Den.	% band purity	% lane purity	Mol. Wt.	Rel. Quant. (w/ LB Corr. Vol.)
1	12,880,860	10,312,009	504	0.47	25,557	20,460	100	52.653	42.333	1.029

Lane 9 - 2uM Thapsigargin

#	Vol. (Int.)	Local Bg. Corr. Vol.	Area	Rf	Density	Local Bg. Corr. Den.	% band purity	% lane purity	Mol. Wt.	Rel. Quant. (w/ LB Corr. Vol.)
1	14,083,812	12,488,132	546	0.462	25,794	22,872	100	59.621	43.333	1.246

iBright™ Image Analysis Report

Katarina+ Chang

18 November 2022

MLCK_CHEMI_10052021_121650

Date:	5 October 2021 12:16:50PM
Mode:	Chemi Blots
Notes:	
Model:	FL1500
Instrument name:	2462619090234
Serial No:	2462619090234
Firmware version:	1.6.0
iBA version:	5.0
Image size:	676px X 540px
Image area:	118.63mm X 94.91mm
Optical Zoom:	1.9x
Digital Zoom:	1x
Focus level:	430
Resolution:	5 x 5
Exposure time:	15000 ms
Exposure mode:	Normal

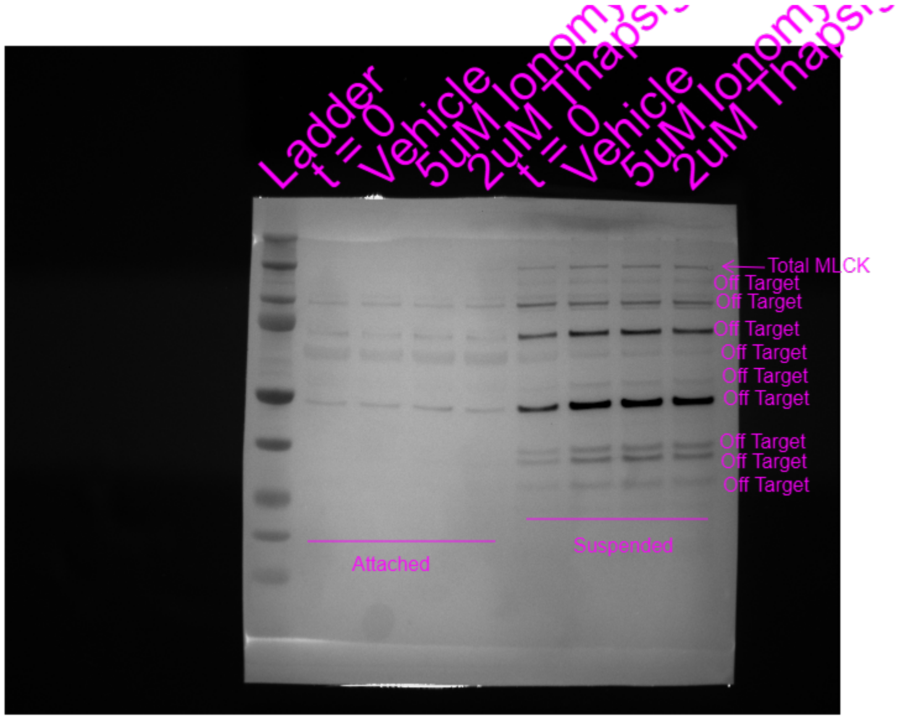

MLCK_CHEMI_10052021_121650

Date:	5 October 2021 12:16:50PM
Mode:	Chemi Blots
Notes:	
Model:	FL1500
Instrument name:	2462619090234
Serial No:	2462619090234
Firmware version:	1.6.0
iBA version:	5.0
Image size:	676px X 540px
Image area:	118.63mm X 94.91mm
Optical Zoom:	1.9x
Digital Zoom:	1x
Focus level:	430
Resolution:	5 x 5
Exposure time:	15000 ms
Exposure mode:	Normal

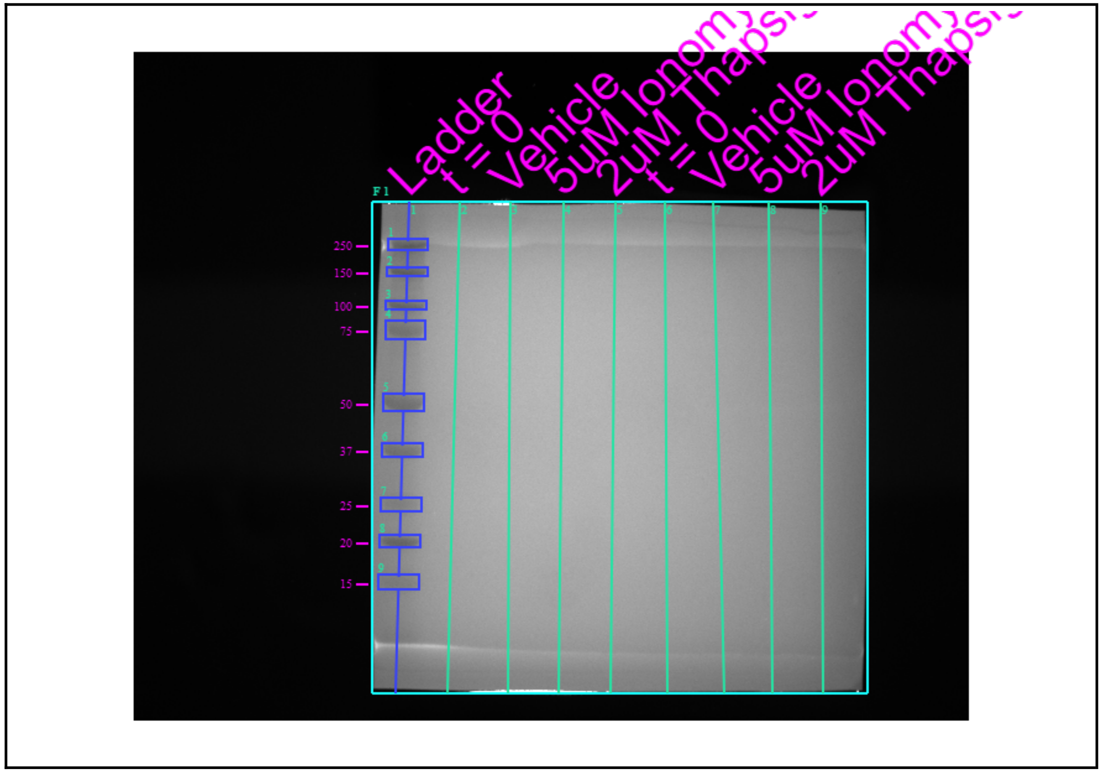

MLCK_CHEMI_10052021_121650

Date:	5 October 2021 12:16:50PM
Mode:	Chemi Blots
Notes:	
Model:	FL1500
Instrument name:	2462619090234
Serial No:	2462619090234
Firmware version:	1.6.0
iBA version:	5.0
Image size:	676px X 540px
Image area:	118.63mm X 94.91mm
Optical Zoom:	1.9x
Digital Zoom:	1x
Focus level:	430
Resolution:	5 x 5
Exposure time:	15000 ms
Exposure mode:	Normal

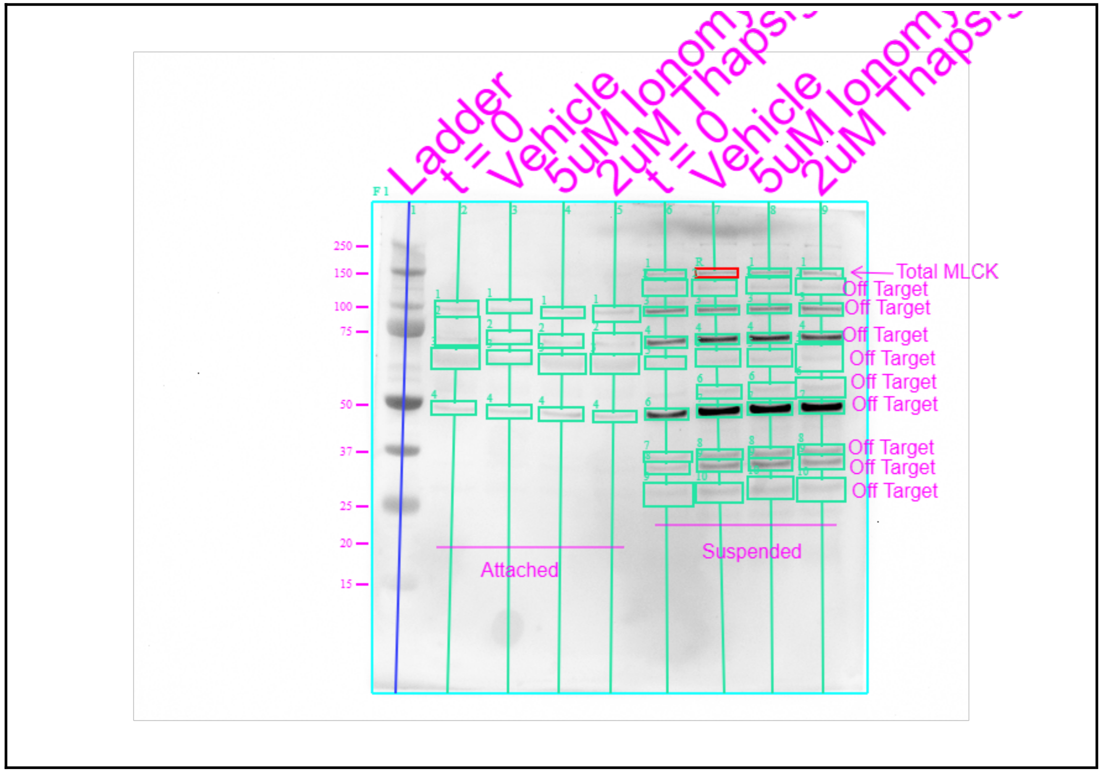

LANE AND BAND ANALYSIS DATA TABLE

MLCK_CHEMI_10052021_121650

Frame: 1
Channel: Membrane
Sensitivity: 100
Molecular Weight Analysis Regression Method : Point to Point

Lane 1 - Ladder

#	Vol. (Int.)	Local Bg. Corr. Vol.	Area	Rf	Density	Local Bg. Corr. Den.	% band purity	% lane purity	Mol. Wt.
1	12,126,482	1,891,448	330	0.086	36,746	5,731.661	12.242	2.864	250
2	9,955,428	1,487,856	272	0.141	36,600	5,470.06	9.63	2.352	150
3	9,593,764	1,459,878	272	0.209	35,271	5,367.201	9.449	2.266	100
4	17,266,004	2,004,708	528	0.259	32,700	3,796.796	12.975	4.078	75
5	17,212,893	2,665,788	510	0.408	33,750	5,227.037	17.254	4.066	50
6	13,615,234	1,958,024	408	0.504	33,370	4,799.079	12.673	3.216	37
7	12,649,913	922,651	408	0.615	31,004	2,261.402	5.972	2.988	25
8	13,133,389	2,079,609	374	0.69	35,116	5,560.453	13.46	3.102	20
9	14,450,729	980,723	442	0.773	32,693	2,218.832	6.347	3.413	15

Frame: 1
Channel: Chemi
Sensitivity: 100
Molecular Weight Analysis Regression Method : Point to Point

Lane 2 - t = 0

#	Vol. (Int.)	Local Bg. Corr. Vol.	Area	Rf	Density	Local Bg. Corr. Den.	% band purity	% lane purity	Mol. Wt.	Rel. Quant. (w/ LB Corr. Vol.)
1	3,767,111	797,757	504	0.217	7,474.427	1,582.852	19.271	6.272	96.25	0.931
2	6,095,571	1,127,291	888	0.262	6,864.382	1,269.473	27.232	10.149	74.576	1.315
3	5,229,145	1,557,828	720	0.317	7,262.701	2,163.651	37.632	8.707	65.254	1.818
4	2,449,106	656,772	444	0.418	5,516.005	1,479.218	15.865	4.078	48.632	0.766

Lane 3 - Vehicle

#	Vol. (Int.)	Local Bg. Corr. Vol.	Area	Rf	Density	Local Bg. Corr. Den.	% band purity	% lane purity	Mol. Wt.	Rel. Quant. (w/ LB Corr. Vol.)
1	2,660,995	735,123	444	0.212	5,993.232	1,655.684	28.472	4.937	98.75	0.858

#	Vol. (Int.)	Local Bg. Corr. Vol.	Area	Rf	Density	Local Bg. Corr. Den.	% band purity	% lane purity	Mol. Wt.	Rel. Quant. (w/ LB Corr. Vol.)
2	2,290,314	410,594	444	0.275	5,158.365	924.763	15.903	4.25	72.458	0.479
3	2,621,462	687,740	444	0.315	5,904.194	1,548.965	26.637	4.864	65.678	0.802
4	2,218,738	748,448	444	0.426	4,997.158	1,685.694	28.988	4.117	47.605	0.873

Lane 4 - 5uM Ionomycin

#	Vol. (Int.)	Local Bg. Corr. Vol.	Area	Rf	Density	Local Bg. Corr. Den.	% band purity	% lane purity	Mol. Wt.	Rel. Quant. (w/ LB Corr. Vol.)
1	2,402,643	495,335	360	0.224	6,674.008	1,375.931	13.947	3.817	92.5	0.578
2	2,749,821	746,693	444	0.282	6,193.291	1,681.741	21.025	4.368	71.186	0.871
3	4,150,692	1,320,514	663	0.33	6,260.471	1,991.726	37.183	6.594	63.136	1.541
4	2,692,773	988,888	444	0.431	6,064.804	2,227.226	27.845	4.278	46.921	1.154

Lane 5 - 2uM Thapsigargin

#	Vol. (Int.)	Local Bg. Corr. Vol.	Area	Rf	Density	Local Bg. Corr. Den.	% band purity	% lane purity	Mol. Wt.	Rel. Quant. (w/ LB Corr. Vol.)
1	4,775,718	669,753	546	0.227	8,746.736	1,226.654	20.738	5.205	91.25	0.781
2	5,090,257	393,292	720	0.287	7,069.801	546.239	12.178	5.547	70.339	0.459
3	5,252,267	1,482,943	680	0.33	7,723.922	2,180.799	45.918	5.724	63.136	1.73
4	2,653,807	683,535	360	0.436	7,371.686	1,898.71	21.165	2.892	46.237	0.798

Lane 6 - t = 0

#	Vol. (Int.)	Local Bg. Corr. Vol.	Area	Rf	Density	Local Bg. Corr. Den.	% band purity	% lane purity	Mol. Wt.	Rel. Quant. (w/ LB Corr. Vol.)
1	3,449,089	518,632	272	0.146	12,680	1,906.739	3.763	2.52	146.296	0.605
2	5,946,873	165,050	518	0.174	11,480	318.629	1.198	4.344	125.926	0.193
3	5,187,434	1,955,354	288	0.222	18,011	6,789.424	14.188	3.79	93.75	2.282
4	6,371,800	3,267,806	350	0.285	18,205	9,336.59	23.711	4.655	70.763	3.813
5	3,476,451	700,775	374	0.327	9,295.324	1,873.732	5.085	2.54	63.559	0.818
6	8,657,330	5,555,130	360	0.431	24,048	15,430	40.308	6.324	46.921	6.482
7	3,804,327	457,414	360	0.519	10,567	1,270.596	3.319	2.779	35.364	0.534
8	4,521,713	579,343	407	0.542	11,109	1,423.449	4.204	3.303	32.909	0.676
9	8,564,670	582,091	820	0.594	10,444	709.868	4.224	6.257	27.182	0.679

Lane 7 - Vehicle

#	Vol. (Int.)	Local Bg. Corr. Vol.	Area	Rf	Density	Local Bg. Corr. Den.	% band purity	% lane purity	Mol. Wt.	Rel. Quant. (w/ LB Corr. Vol.)
1	3,580,458	857,028	280	0.144	12,787	3,060.816	3.559	2.441	148.148	1
2	5,678,878	247,600	518	0.174	10,963	477.993	1.028	3.872	125.926	0.289
3	5,138,507	1,996,670	288	0.219	17,842	6,932.884	8.291	3.503	95	2.33
4	8,477,767	5,136,143	324	0.28	26,165	15,852	21.326	5.78	71.61	5.993
5	5,620,157	474,686	555	0.317	10,126	855.29	1.971	3.832	65.254	0.554
6	4,659,961	461,347	444	0.385	10,495	1,039.07	1.916	3.177	53.814	0.538
7	15,567,771	10,979,321	407	0.426	38,250	26,976	45.589	10.613	47.605	12.811
8	4,942,151	961,080	342	0.514	14,450	2,810.177	3.991	3.369	35.909	1.121
9	6,423,503	1,688,087	407	0.537	15,782	4,147.635	7.009	4.379	33.455	1.97
10	7,478,397	1,281,451	663	0.592	11,279	1,932.808	5.321	5.098	27.455	1.495

Lane 8 - 5uM Ionomycin

#	Vol. (Int.)	Local Bg. Corr. Vol.	Area	Rf	Density	Local Bg. Corr. Den.	% band purity	% lane purity	Mol. Wt.	Rel. Quant. (w/ LB Corr. Vol.)
1	3,168,242	915,955	245	0.144	12,931	3,738.593	3.658	2.321	148.148	1.069
2	5,347,231	178,675	518	0.169	10,322	344.934	0.713	3.918	129.63	0.208
3	5,078,318	2,090,224	288	0.217	17,633	7,257.726	8.347	3.721	96.25	2.439
4	8,711,196	5,381,757	324	0.277	26,886	16,610	21.49	6.383	72.034	6.28
5	5,506,520	551,275	555	0.317	9,921.658	993.29	2.201	4.035	65.254	0.643
6	5,799,536	9,249.84	546	0.383	10,621	16.941	0.037	4.249	54.237	0.011
7	16,204,735	11,333,322	407	0.421	39,815	27,846	45.255	11.873	48.289	13.224
8	5,533,875	972,413	370	0.509	14,956	2,628.144	3.883	4.055	36.455	1.135
9	6,780,530	2,124,119	396	0.534	17,122	5,363.937	8.482	4.968	33.727	2.478
10	7,913,707	1,486,087	722	0.582	10,960	2,058.292	5.934	5.798	28.545	1.734

Lane 9 - 2uM Thapsigargin

#	Vol. (Int.)	Local Bg. Corr. Vol.	Area	Rf	Density	Local Bg. Corr. Den.	% band purity	% lane purity	Mol. Wt.	Rel. Quant. (w/ LB Corr. Vol.)
1	3,200,591	880,336	315	0.146	10,160	2,794.718	3.651	2.799	146.296	1.027
2	4,875,570	291,738	574	0.171	8,494.024	508.255	1.21	4.264	127.778	0.34
3	5,738,591	2,612,304	396	0.219	14,491	6,596.729	10.835	5.018	95	3.048
4	7,391,503	4,752,015	324	0.275	22,813	14,666	19.709	6.464	72.458	5.545
5	7,181,067	43,505	897	0.317	8,005.649	48.501	0.18	6.28	65.254	0.051

#	Vol. (Int.)	Local Bg. Corr. Vol.	Area	Rf	Density	Local Bg. Corr. Den.	% band purity	% lane purity	Mol. Wt.	Rel. Quant. (w/ LB Corr. Vol.)
6	6,592,613	32,527	697	0.378	9,458.555	46.669	0.135	5.765	55.085	0.038
7	15,450,963	11,113,561	407	0.418	37,963	27,306	46.094	13.512	48.632	12.968
8	4,541,675	1,228,583	342	0.504	13,279	3,592.35	5.096	3.972	37	1.434
9	6,514,276	1,881,054	481	0.529	13,543	3,910.717	7.802	5.697	34.273	2.195
10	6,837,256	1,275,156	800	0.584	8,546.57	1,593.945	5.289	5.979	28.273	1.488

iBright™ Image Analysis Report

Katarina+ Chang

18 November 2022

pCofilin_CHEMI_10062021_130159

Date:	6 October 2021 01:01:59PM
Mode:	Chemi Blots
Notes:	
Model:	FL1500
Instrument name:	2462619090234
Serial No:	2462619090234
Firmware version:	1.6.0
iBA version:	5.0
Image size:	676px X 540px
Image area:	118.63mm X 94.91mm
Optical Zoom:	1.9x
Digital Zoom:	1x
Focus level:	430
Resolution:	5 x 5
Exposure time:	115691 ms
Exposure mode:	Normal

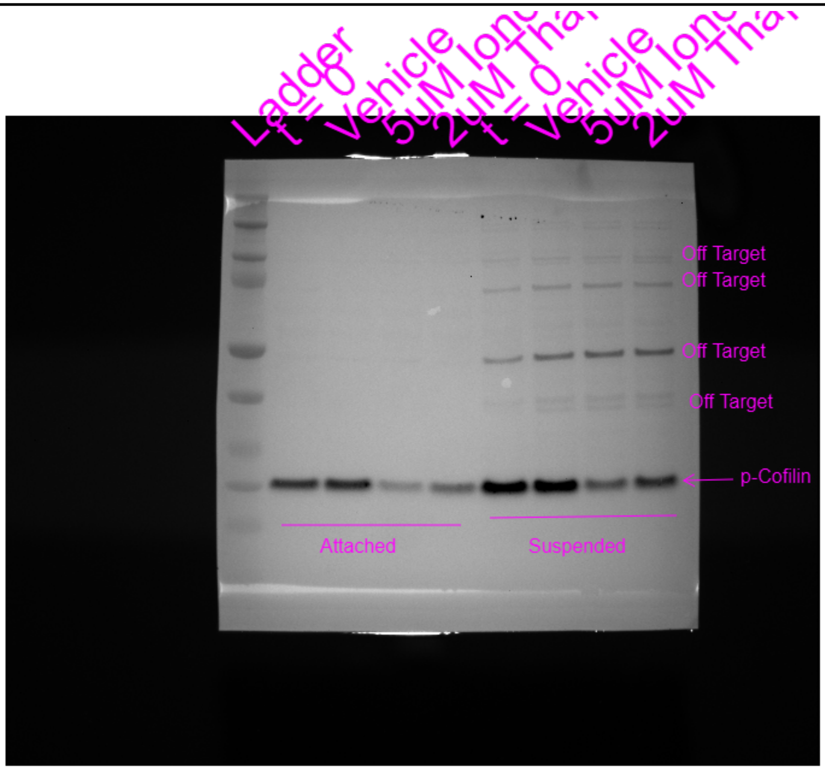

pCofilin_CHEMI_10062021_130159

Date:	6 October 2021 01:01:59PM
Mode:	Chemi Blots
Notes:	
Model:	FL1500
Instrument name:	2462619090234
Serial No:	2462619090234
Firmware version:	1.6.0
iBA version:	5.0
Image size:	676px X 540px
Image area:	118.63mm X 94.91mm
Optical Zoom:	1.9x
Digital Zoom:	1x
Focus level:	430
Resolution:	5 x 5
Exposure time:	115691 ms
Exposure mode:	Normal

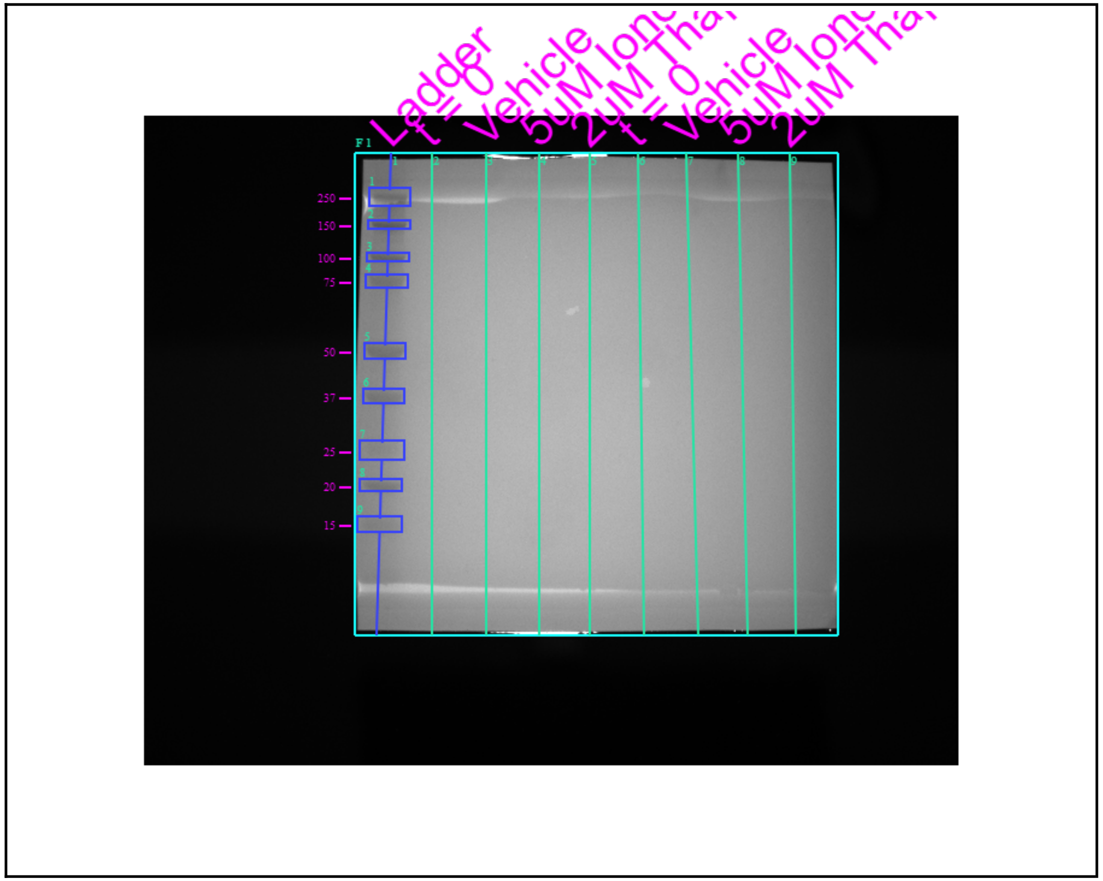

pCofilin_CHEMI_10062021_130159

Date:	6 October 2021 01:01:59PM
Mode:	Chemi Blots
Notes:	
Model:	FL1500
Instrument name:	2462619090234
Serial No:	2462619090234
Firmware version:	1.6.0
iBA version:	5.0
Image size:	676px X 540px
Image area:	118.63mm X 94.91mm
Optical Zoom:	1.9x
Digital Zoom:	1x
Focus level:	430
Resolution:	5 x 5
Exposure time:	115691 ms
Exposure mode:	Normal

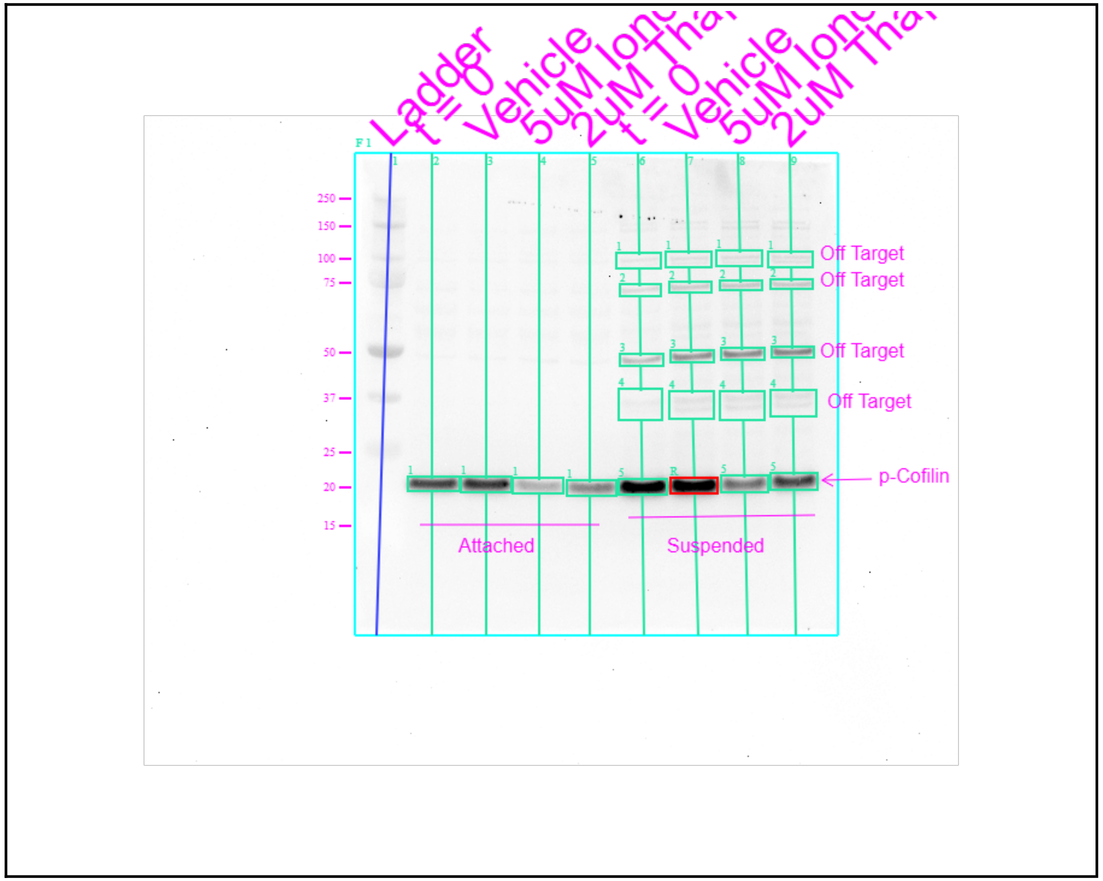

LANE AND BAND ANALYSIS DATA TABLE

pCofilin_CHEMI_10062021_130159

Frame: 1
Channel: Membrane
Sensitivity: 100
Molecular Weight Analysis Regression Method : Point to Point

Lane 1 - Ladder

#	Vol. (Int.)	Local Bg. Corr. Vol.	Area	Rf	Density	Local Bg. Corr. Den.	% band purity	% lane purity	Mol. Wt.
1	19,009,214	1,368,332	560	0.09	33,945	2,443.45	12.517	4.179	250
2	10,904,637	1,476,758	288	0.147	37,863	5,127.635	13.508	2.397	150
3	10,384,949	1,366,274	288	0.214	36,058	4,744.007	12.498	2.283	100
4	14,604,441	1,389,152	432	0.264	33,806	3,215.631	12.707	3.211	75
5	16,467,904	2,126,381	490	0.409	33,607	4,339.553	19.451	3.621	50
6	14,791,651	1,755,827	455	0.504	32,509	3,858.962	16.061	3.252	37
7	19,061,892	480,267	646	0.616	29,507	743.448	4.393	4.191	25
8	12,810,395	961,741	396	0.688	32,349	2,428.64	8.797	2.816	20
9	16,498,615	7,372.297	532	0.768	31,012	13.858	0.067	3.627	15

Frame: 1
Channel: Chemi
Sensitivity: 100
Molecular Weight Analysis Regression Method : Point to Point

Lane 2 - t = 0

#	Vol. (Int.)	Local Bg. Corr. Vol.	Area	Rf	Density	Local Bg. Corr. Den.	% band purity	% lane purity	Mol. Wt.	Rel. Quant. (w/ LB Corr. Vol.)
1	9,906,465	7,308,108	572	0.686	17,318	12,776	100	35.81	20.172	0.571

Lane 3 - Vehicle

#	Vol. (Int.)	Local Bg. Corr. Vol.	Area	Rf	Density	Local Bg. Corr. Den.	% band purity	% lane purity	Mol. Wt.	Rel. Quant. (w/ LB Corr. Vol.)
1	11,112,729	8,273,539	602	0.686	18,459	13,743	100	38.163	20.172	0.646

Lane 4 - 5uM Ionomycin

#	Vol. (Int.)	Local Bg. Corr. Vol.	Area	Rf	Density	Local Bg. Corr. Den.	% band purity	% lane purity	Mol. Wt.	Rel. Quant. (w/ LB Corr. Vol.)

#	Vol. (Int.)	Local Bg. Corr. Vol.	Area	Rf	Density	Local Bg. Corr. Den.	% band purity	% lane purity	Mol. Wt.	Rel. Quant. (w/ LB Corr. Vol.)
1	4,522,491	2,152,194	602	0.688	7,512.444	3,575.074	100	19.111	20	0.168

Lane 5 - 2uM Thapsigargin

#	Vol. (Int.)	Local Bg. Corr. Vol.	Area	Rf	Density	Local Bg. Corr. Den.	% band purity	% lane purity	Mol. Wt.	Rel. Quant. (w/ LB Corr. Vol.)
1	6,810,590	3,879,419	588	0.693	11,582	6,597.651	100	24.539	19.688	0.303

Lane 6 - t = 0

#	Vol. (Int.)	Local Bg. Corr. Vol.	Area	Rf	Density	Local Bg. Corr. Den.	% band purity	% lane purity	Mol. Wt.	Rel. Quant. (w/ LB Corr. Vol.)
1	1,317,236	335,175	532	0.222	2,476.008	630.03	2.219	2.895	96.25	0.026
2	1,190,224	496,578	385	0.284	3,091.491	1,289.814	3.288	2.616	71.552	0.039
3	2,006,590	1,223,848	407	0.429	4,930.197	3,006.999	8.103	4.411	47.263	0.096
4	2,206,131	455,721	999	0.521	2,208.339	456.178	3.017	4.849	35.133	0.036
5	16,843,372	12,592,854	574	0.691	29,343	21,938	83.373	37.024	19.844	0.984

Lane 7 - Vehicle

#	Vol. (Int.)	Local Bg. Corr. Vol.	Area	Rf	Density	Local Bg. Corr. Den.	% band purity	% lane purity	Mol. Wt.	Rel. Quant. (w/ LB Corr. Vol.)
1	1,632,142	452,405	546	0.219	2,989.271	828.582	2.603	3.208	97.5	0.035
2	1,604,798	807,343	360	0.277	4,457.772	2,242.622	4.645	3.154	72.845	0.063
3	3,450,946	2,432,211	407	0.421	8,478.983	5,975.949	13.994	6.783	48.289	0.19
4	2,752,801	890,716	912	0.521	3,018.422	976.663	5.125	5.411	35.133	0.07
5	16,895,439	12,798,077	560	0.688	30,170	22,853	73.634	33.21	20	1

Lane 8 - 5uM Ionomycin

#	Vol. (Int.)	Local Bg. Corr. Vol.	Area	Rf	Density	Local Bg. Corr. Den.	% band purity	% lane purity	Mol. Wt.	Rel. Quant. (w/ LB Corr. Vol.)
1	1,805,932	491,418	546	0.217	3,307.568	900.035	5.32	4.195	98.75	0.038
2	1,637,200	830,257	324	0.274	5,053.086	2,562.522	8.989	3.803	73.276	0.065
3	3,677,879	2,556,147	407	0.416	9,036.558	6,280.462	27.674	8.543	48.974	0.2
4	2,942,640	932,068	950	0.524	3,097.516	981.125	10.091	6.835	34.867	0.073
5	7,632,206	4,426,591	600	0.686	12,720	7,377.652	47.925	17.727	20.172	0.346

Lane 9 - 2uM Thapsigargin

#	Vol. (Int.)	Local Bg. Corr. Vol.	Area	Rf	Density	Local Bg. Corr. Den.	% band purity	% lane purity	Mol. Wt.	Rel. Quant. (w/ LB Corr. Vol.)
1	1,799,317	545,750	532	0.219	3,382.175	1,025.846	4.365	4.171	97.5	0.043
2	1,605,091	819,176	324	0.272	4,953.985	2,528.324	6.552	3.72	73.707	0.064
3	3,627,242	2,600,414	360	0.411	10,075	7,223.373	20.8	8.408	49.658	0.203
4	2,834,010	982,266	897	0.519	3,159.431	1,095.057	7.857	6.569	35.4	0.077
5	9,940,570	7,554,435	600	0.681	16,567	12,590	60.426	23.042	20.517	0.59

iBright™ Image Analysis Report

Katarina+ Chang

18 November 2022

Alpha Tubulin_CHEMI_10072021_123408

Date:	7 October 2021 12:34:08PM
Mode:	Chemi Blots
Notes:	
Model:	FL1500
Instrument name:	2462619090234
Serial No:	2462619090234
Firmware version:	1.6.0
iBA version:	5.0
Image size:	615px X 491px
Image area:	112.7mm X 90.16mm
Optical Zoom:	2x
Digital Zoom:	1.1x
Focus level:	455
Resolution:	5 x 5
Exposure time:	520 ms
Exposure mode:	Normal

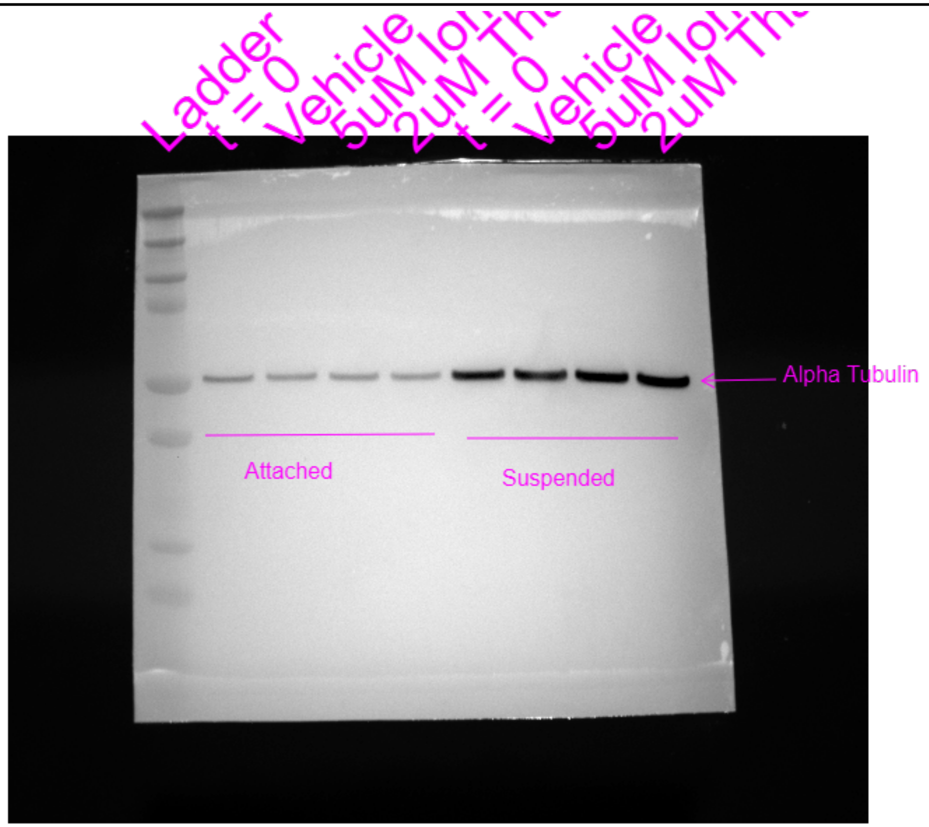

Alpha Tubulin_CHEMI_10072021_123408

Date:	7 October 2021 12:34:08PM
Mode:	Chemi Blots
Notes:	
Model:	FL1500
Instrument name:	2462619090234
Serial No:	2462619090234
Firmware version:	1.6.0
iBA version:	5.0
Image size:	615px X 491px
Image area:	112.7mm X 90.16mm
Optical Zoom:	2x
Digital Zoom:	1.1x
Focus level:	455
Resolution:	5 x 5
Exposure time:	520 ms
Exposure mode:	Normal

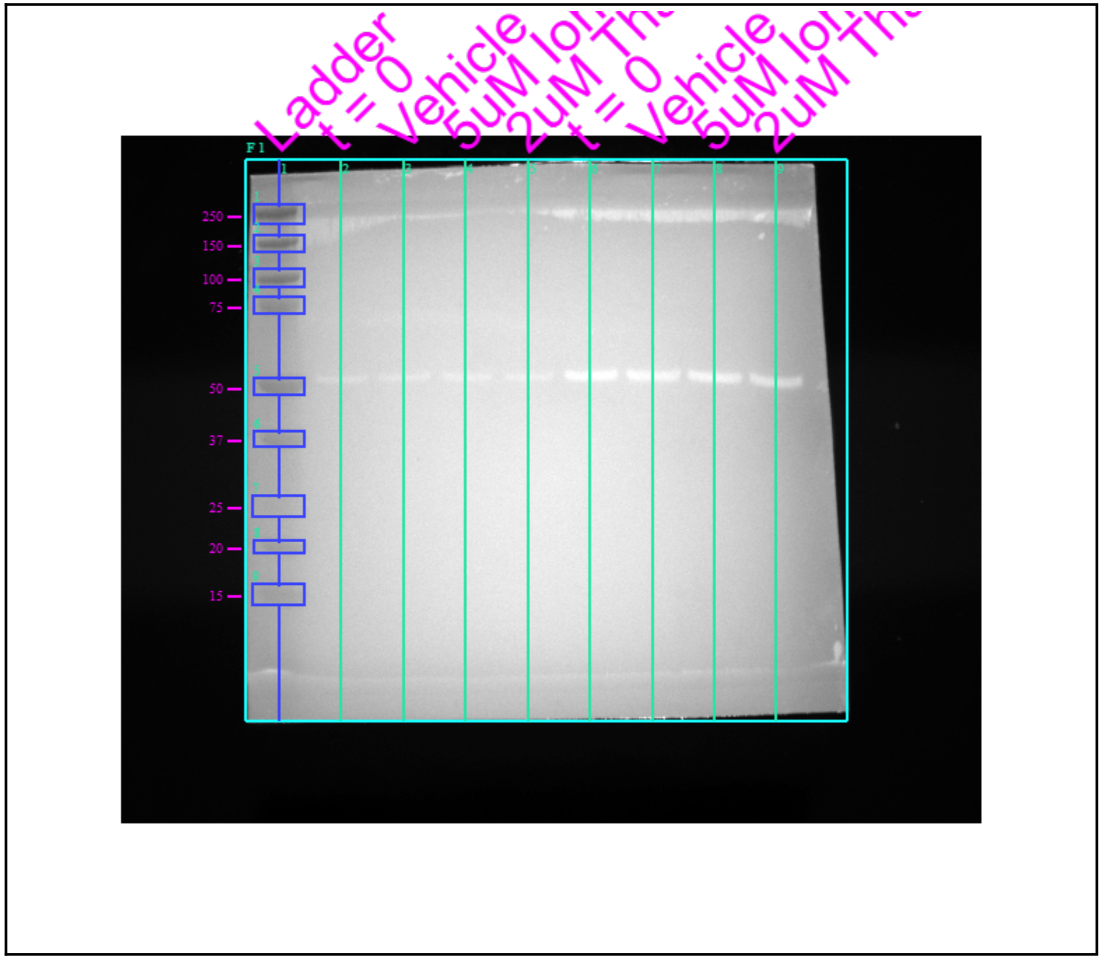

Alpha Tubulin_CHEMI_10072021_123408

Date:	7 October 2021 12:34:08PM
Mode:	Chemi Blots
Notes:	
Model:	FL1500
Instrument name:	2462619090234
Serial No:	2462619090234
Firmware version:	1.6.0
iBA version:	5.0
Image size:	615px X 491px
Image area:	112.7mm X 90.16mm
Optical Zoom:	2x
Digital Zoom:	1.1x
Focus level:	455
Resolution:	5 x 5
Exposure time:	520 ms
Exposure mode:	Normal

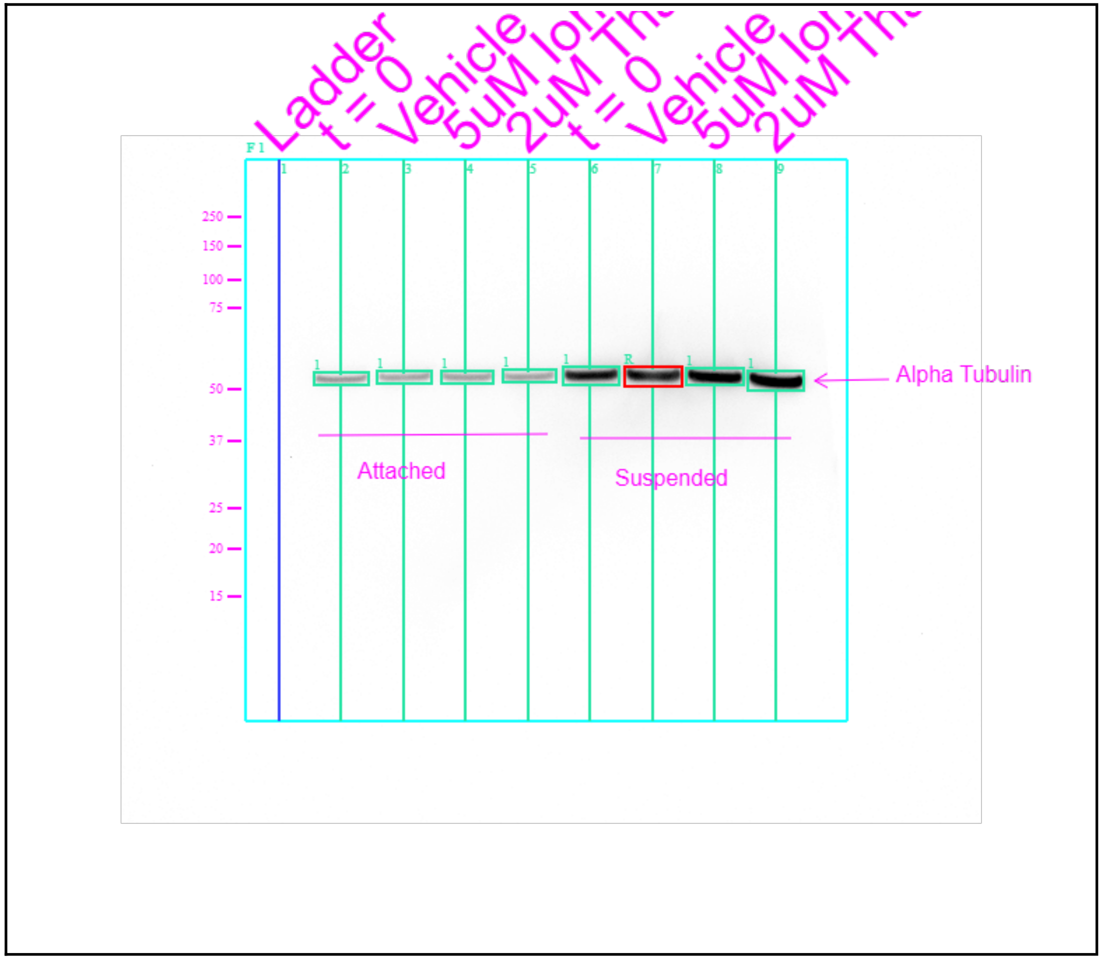

LANE AND BAND ANALYSIS DATA TABLE

Alpha Tubulin_CHEMI_10072021_123408

Frame: 1
Channel: Membrane
Sensitivity: 100
Molecular Weight Analysis Regression Method : Point to Point

Lane 1 - Ladder

#	Vol. (Int.)	Local Bg. Corr. Vol.	Area	Rf	Density	Local Bg. Corr. Den.	% band purity	% lane purity	Mol. Wt.
1	21,446,835	1,757,628	555	0.097	38,642	3,166.899	16.925	4.263	250
2	17,636,709	1,441,710	481	0.15	36,666	2,997.32	13.883	3.505	150
3	18,691,683	1,421,867	518	0.209	36,084	2,744.919	13.692	3.715	100
4	16,744,031	1,252,998	481	0.259	34,810	2,604.987	12.065	3.328	75
5	16,733,474	1,608,974	481	0.404	34,788	3,345.062	15.493	3.326	50
6	14,776,550	1,115,713	444	0.496	33,280	2,512.869	10.743	2.937	37
7	18,805,903	361,576	608	0.616	30,930	594.698	3.482	3.738	25
8	12,065,225	711,568	370	0.688	32,608	1,923.159	6.852	2.398	20
9	19,799,040	712,986	608	0.773	32,564	1,172.675	6.866	3.935	15

Frame: 1
Channel: Chemi
Sensitivity: 100
Molecular Weight Analysis Regression Method : Point to Point

Lane 2 - t = 0

#	Vol. (Int.)	Local Bg. Corr. Vol.	Area	Rf	Density	Local Bg. Corr. Den.	% band purity	% lane purity	Mol. Wt.	Rel. Quant. (w/ LB Corr. Vol.)
1	2,950,539	2,515,465	400	0.389	7,376.348	6,288.663	100	53.191	52.586	0.318

Lane 3 - Vehicle

#	Vol. (Int.)	Local Bg. Corr. Vol.	Area	Rf	Density	Local Bg. Corr. Den.	% band purity	% lane purity	Mol. Wt.	Rel. Quant. (w/ LB Corr. Vol.)
1	2,731,543	2,218,779	400	0.387	6,828.858	5,546.948	100	45.765	53.017	0.281

Lane 4 - 5uM Ionomycin

#	Vol. (Int.)	Local Bg. Corr. Vol.	Area	Rf	Density	Local Bg. Corr. Den.	% band purity	% lane purity	Mol. Wt.	Rel. Quant. (w/ LB Corr. Vol.)

#	Vol. (Int.)	Local Bg. Corr. Vol.	Area	Rf	Density	Local Bg. Corr. Den.	% band purity	% lane purity	Mol. Wt.	Rel. Quant. (w/ LB Corr. Vol.)
1	2,797,954	2,263,025	380	0.387	7,363.037	5,955.331	100	42.332	53.017	0.286

Lane 5 - 2uM Thapsigargin

#	Vol. (Int.)	Local Bg. Corr. Vol.	Area	Rf	Density	Local Bg. Corr. Den.	% band purity	% lane purity	Mol. Wt.	Rel. Quant. (w/ LB Corr. Vol.)
1	2,919,121	2,180,254	390	0.384	7,484.926	5,590.395	100	38.156	53.448	0.276

Lane 6 - t = 0

#	Vol. (Int.)	Local Bg. Corr. Vol.	Area	Rf	Density	Local Bg. Corr. Den.	% band purity	% lane purity	Mol. Wt.	Rel. Quant. (w/ LB Corr. Vol.)
1	10,126,660	8,227,473	574	0.384	17,642	14,333	100	61.58	53.448	1.041

Lane 7 - Vehicle

#	Vol. (Int.)	Local Bg. Corr. Vol.	Area	Rf	Density	Local Bg. Corr. Den.	% band purity	% lane purity	Mol. Wt.	Rel. Quant. (w/ LB Corr. Vol.)
1	10,959,614	7,903,954	630	0.387	17,396	12,545	100	58.44	53.017	1

Lane 8 - 5uM Ionomycin

#	Vol. (Int.)	Local Bg. Corr. Vol.	Area	Rf	Density	Local Bg. Corr. Den.	% band purity	% lane purity	Mol. Wt.	Rel. Quant. (w/ LB Corr. Vol.)
1	13,077,443	9,884,715	546	0.387	23,951	18,103	100	62.985	53.017	1.251

Lane 9 - 2uM Thapsigargin

#	Vol. (Int.)	Local Bg. Corr. Vol.	Area	Rf	Density	Local Bg. Corr. Den.	% band purity	% lane purity	Mol. Wt.	Rel. Quant. (w/ LB Corr. Vol.)
1	14,518,753	12,410,042	615	0.394	23,607	20,178	100	74.63	51.724	1.57

iBright™ Image Analysis Report

Katarina+ Chang

18 November 2022

GAPDH_CHEMI_10082021_125607

Date:	8 October 2021 12:56:07PM
Mode:	Chemi Blots
Notes:	
Model:	FL1500
Instrument name:	2462619090234
Serial No:	2462619090234
Firmware version:	1.6.0
iBA version:	5.0
Image size:	563px X 450px
Image area:	112.7mm X 90.16mm
Optical Zoom:	2x
Digital Zoom:	1.2x
Focus level:	455
Resolution:	5 x 5
Exposure time:	1949 ms
Exposure mode:	Normal

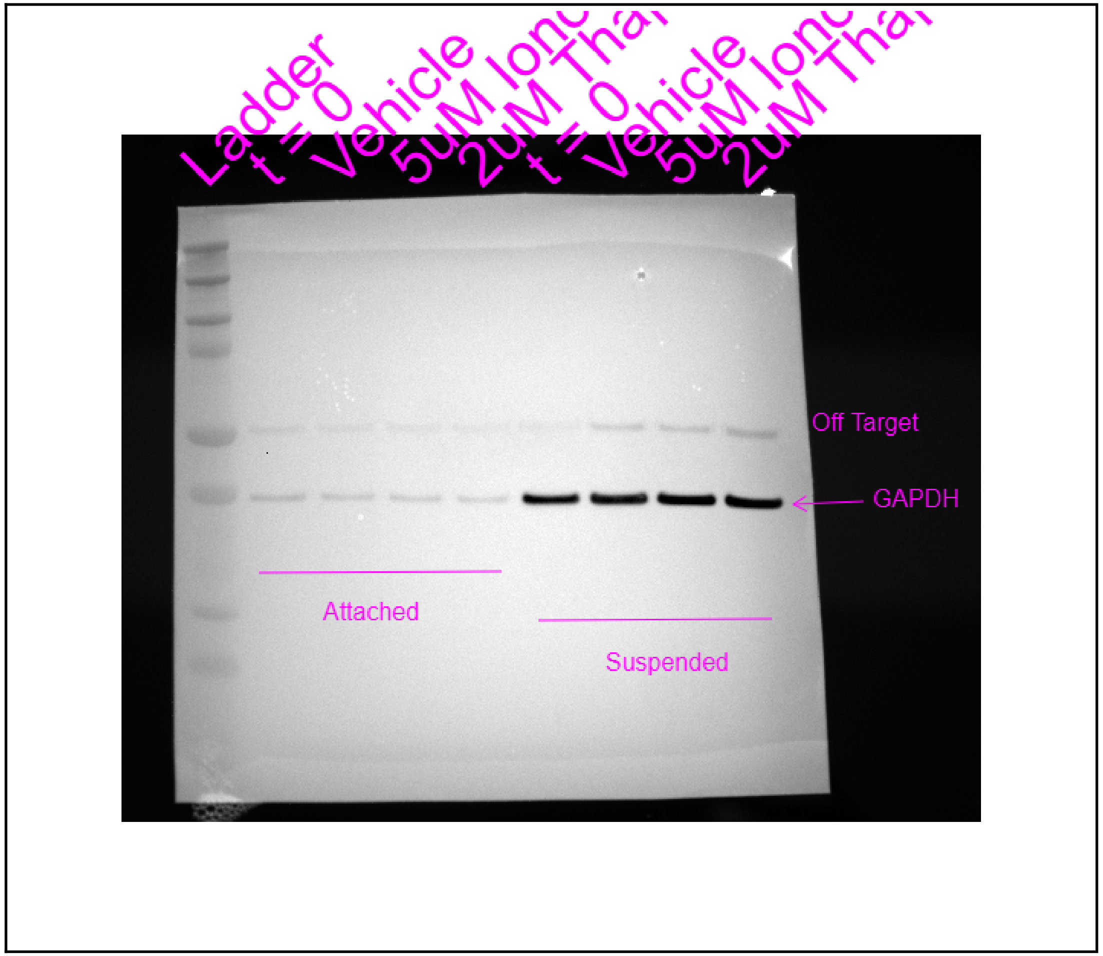

GAPDH_CHEMI_10082021_125607

Date:	8 October 2021 12:56:07PM
Mode:	Chemi Blots
Notes:	
Model:	FL1500
Instrument name:	2462619090234
Serial No:	2462619090234
Firmware version:	1.6.0
iBA version:	5.0
Image size:	563px X 450px
Image area:	112.7mm X 90.16mm
Optical Zoom:	2x
Digital Zoom:	1.2x
Focus level:	455
Resolution:	5 x 5
Exposure time:	1949 ms
Exposure mode:	Normal

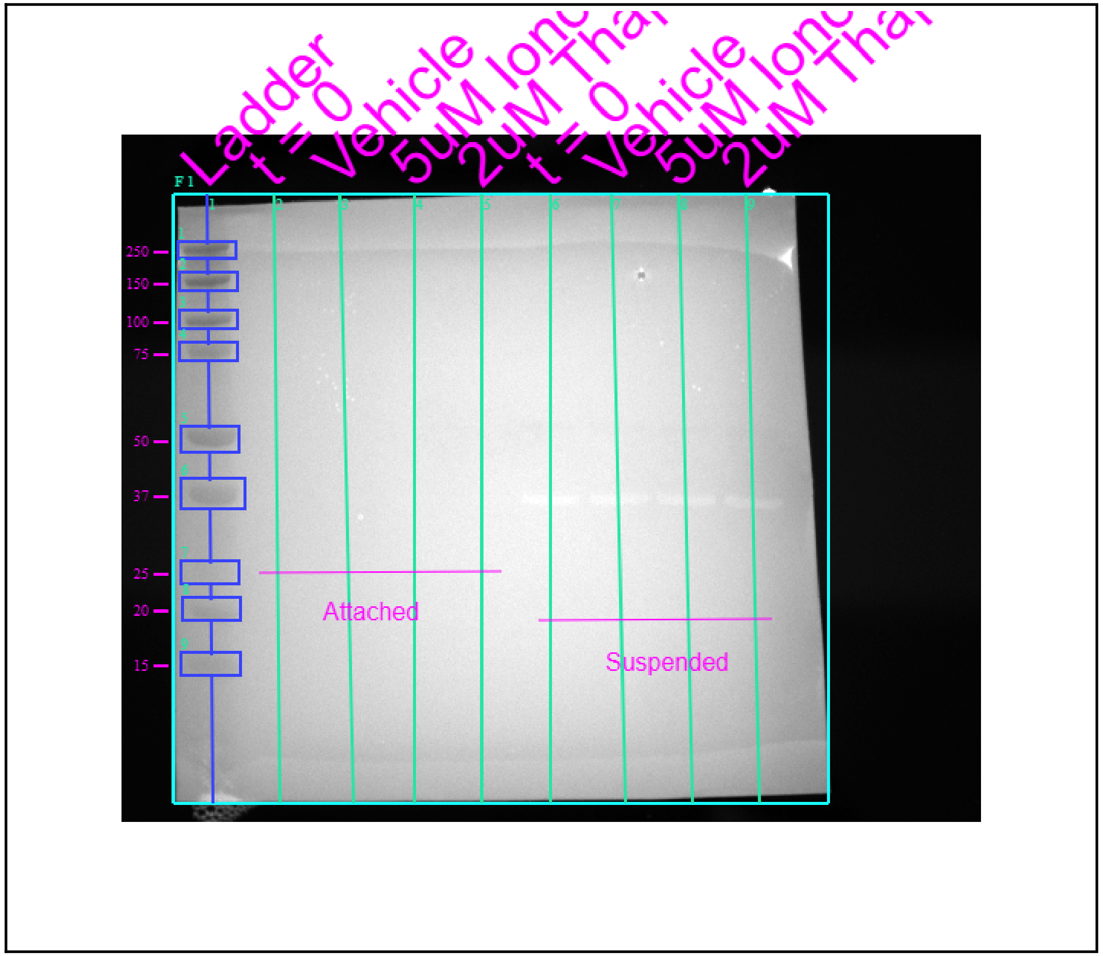

GAPDH_CHEMI_10082021_125607

Date:	8 October 2021 12:56:07PM
Mode:	Chemi Blots
Notes:	
Model:	FL1500
Instrument name:	2462619090234
Serial No:	2462619090234
Firmware version:	1.6.0
iBA version:	5.0
Image size:	563px X 450px
Image area:	112.7mm X 90.16mm
Optical Zoom:	2x
Digital Zoom:	1.2x
Focus level:	455
Resolution:	5 x 5
Exposure time:	1949 ms
Exposure mode:	Normal

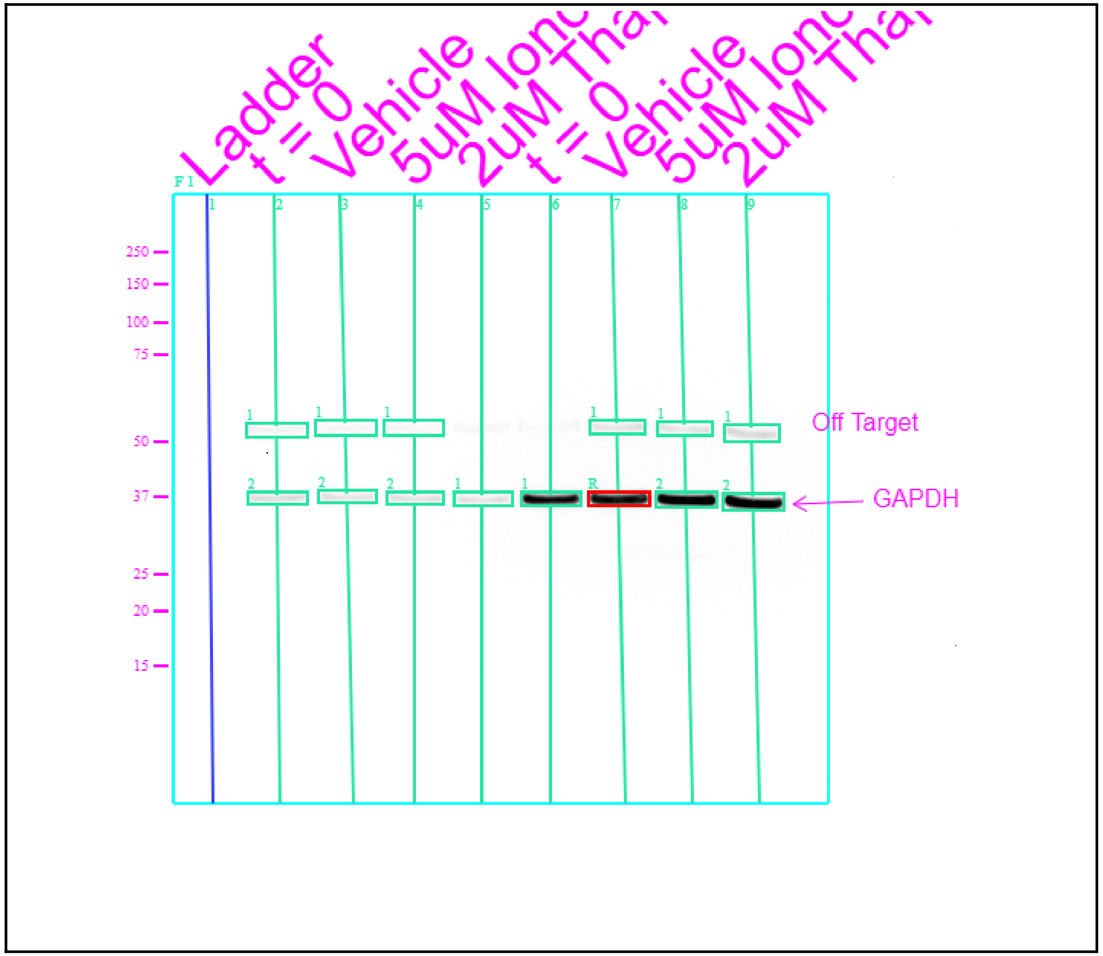

LANE AND BAND ANALYSIS DATA TABLE

GAPDH_CHEMI_10082021_125607

Frame: 1
Channel: Membrane
Sensitivity: 100
Molecular Weight Analysis Regression Method : Point to Point

Lane 1 - Ladder

#	Vol. (Int.)	Local Bg. Corr. Vol.	Area	Rf	Density	Local Bg. Corr. Den.	% band purity	% lane purity	Mol. Wt.
1	18,391,089	446,862	468	0.09	39,297	954.834	5.748	NA	250
2	19,456,200	817,164	507	0.143	38,375	1,611.764	10.51	NA	150
3	18,527,760	757,996	507	0.206	36,543	1,495.062	9.749	NA	100
4	17,807,297	539,621	507	0.258	35,122	1,064.342	6.941	NA	75
5	24,147,996	1,606,514	702	0.401	34,398	2,288.482	20.663	NA	50
6	29,488,300	1,484,593	903	0.491	32,655	1,644.068	19.095	NA	37
7	19,969,529	377,234	624	0.619	32,002	604.543	4.852	NA	25
8	20,648,116	975,775	624	0.679	33,089	1,563.744	12.55	NA	20
9	21,663,182	769,092	640	0.769	33,848	1,201.707	9.892	NA	15

Frame: 1
Channel: Chemi
Sensitivity: 100
Molecular Weight Analysis Regression Method : Point to Point

Lane 2 - t = 0

#	Vol. (Int.)	Local Bg. Corr. Vol.	Area	Rf	Density	Local Bg. Corr. Den.	% band purity	% lane purity	Mol. Wt.	Rel. Quant. (w/ LB Corr. Vol.)
1	458,181	408,757	410	0.386	1,117.515	996.97	30.669	0.017	52.632	0.046
2	1,010,480	924,067	360	0.499	2,806.889	2,566.855	69.331	0.038	36.294	0.103

Lane 3 - Vehicle

#	Vol. (Int.)	Local Bg. Corr. Vol.	Area	Rf	Density	Local Bg. Corr. Den.	% band purity	% lane purity	Mol. Wt.	Rel. Quant. (w/ LB Corr. Vol.)
1	394,476	336,759	451	0.383	874.67	746.695	31.351	0.017	53.07	0.038
2	816,103	737,395	351	0.496	2,325.08	2,100.842	68.649	0.036	36.529	0.082

Lane 4 - 5uM Ionomycin

#	Vol. (Int.)	Local Bg. Corr. Vol.	Area	Rf	Density	Local Bg. Corr. Den.	% band purity	% lane purity	Mol. Wt.	Rel. Quant. (w/ LB Corr. Vol.)
1	384,797	320,212	440	0.383	874.539	727.756	29.375	0.02	53.07	0.036
2	855,513	769,869	342	0.499	2,501.5	2,251.081	70.625	0.045	36.294	0.086

Lane 5 - 2uM Thapsigargin

#	Vol. (Int.)	Local Bg. Corr. Vol.	Area	Rf	Density	Local Bg. Corr. Den.	% band purity	% lane purity	Mol. Wt.	Rel. Quant. (w/ LB Corr. Vol.)
1	890,394	710,465	390	0.499	2,283.062	1,821.707	100	0.057	36.294	0.079

Lane 6 - t = 0

#	Vol. (Int.)	Local Bg. Corr. Vol.	Area	Rf	Density	Local Bg. Corr. Den.	% band purity	% lane purity	Mol. Wt.	Rel. Quant. (w/ LB Corr. Vol.)
1	8,868,097	8,294,621	400	0.499	22,170	20,736	100	0.72	36.294	0.927

Lane 7 - Vehicle

#	Vol. (Int.)	Local Bg. Corr. Vol.	Area	Rf	Density	Local Bg. Corr. Den.	% band purity	% lane purity	Mol. Wt.	Rel. Quant. (w/ LB Corr. Vol.)
1	916,181	690,828	370	0.381	2,476.165	1,867.103	7.168	0.103	53.509	0.077
2	9,775,702	8,946,448	410	0.499	23,843	21,820	92.832	1.1	36.294	1

Lane 8 - 5uM Ionomycin

#	Vol. (Int.)	Local Bg. Corr. Vol.	Area	Rf	Density	Local Bg. Corr. Den.	% band purity	% lane purity	Mol. Wt.	Rel. Quant. (w/ LB Corr. Vol.)
1	910,764	673,645	370	0.383	2,461.524	1,820.664	5.822	0.178	53.07	0.075
2	11,587,312	10,896,437	451	0.501	25,692	24,160	94.178	2.264	36.059	1.218

Lane 9 - 2uM Thapsigargin

#	Vol. (Int.)	Local Bg. Corr. Vol.	Area	Rf	Density	Local Bg. Corr. Den.	% band purity	% lane purity	Mol. Wt.	Rel. Quant. (w/ LB Corr. Vol.)
1	1,222,556	998,018	444	0.391	2,753.505	2,247.789	8.486	1.844	51.754	0.112
2	11,166,920	10,763,186	492	0.504	22,696	21,876	91.514	16.845	35.824	1.203

iBright™ Image Analysis Report

Katarina+ Chang

18 November 2022

Total MLC_CHEMI_10052021_122334

Date:	5 October 2021 12:23:34PM
Mode:	Chemi Blots
Notes:	
Model:	FL1500
Instrument name:	2462619090234
Serial No:	2462619090234
Firmware version:	1.6.0
iBA version:	5.0
Image size:	563px X 450px
Image area:	112.7mm X 90.16mm
Optical Zoom:	2x
Digital Zoom:	1.2x
Focus level:	455
Resolution:	5 x 5
Exposure time:	10000 ms
Exposure mode:	Normal

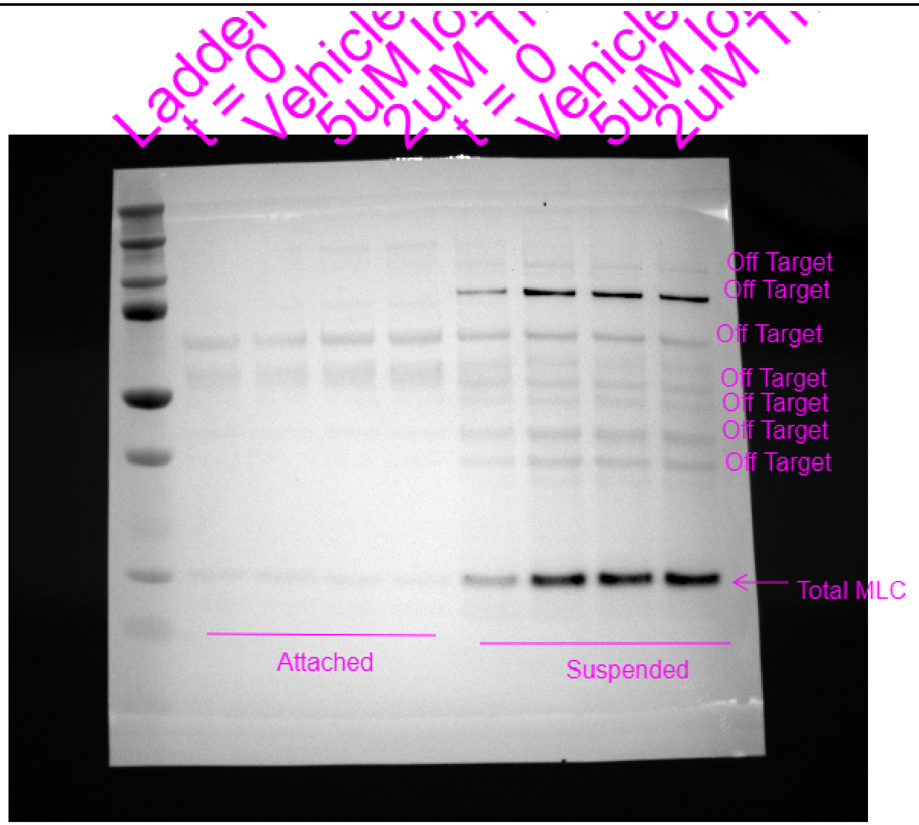

Total MLC_CHEMI_10052021_122334

Date:	5 October 2021 12:23:34PM
Mode:	Chemi Blots
Notes:	
Model:	FL1500
Instrument name:	2462619090234
Serial No:	2462619090234
Firmware version:	1.6.0
iBA version:	5.0
Image size:	563px X 450px
Image area:	112.7mm X 90.16mm
Optical Zoom:	2x
Digital Zoom:	1.2x
Focus level:	455
Resolution:	5 x 5
Exposure time:	10000 ms
Exposure mode:	Normal

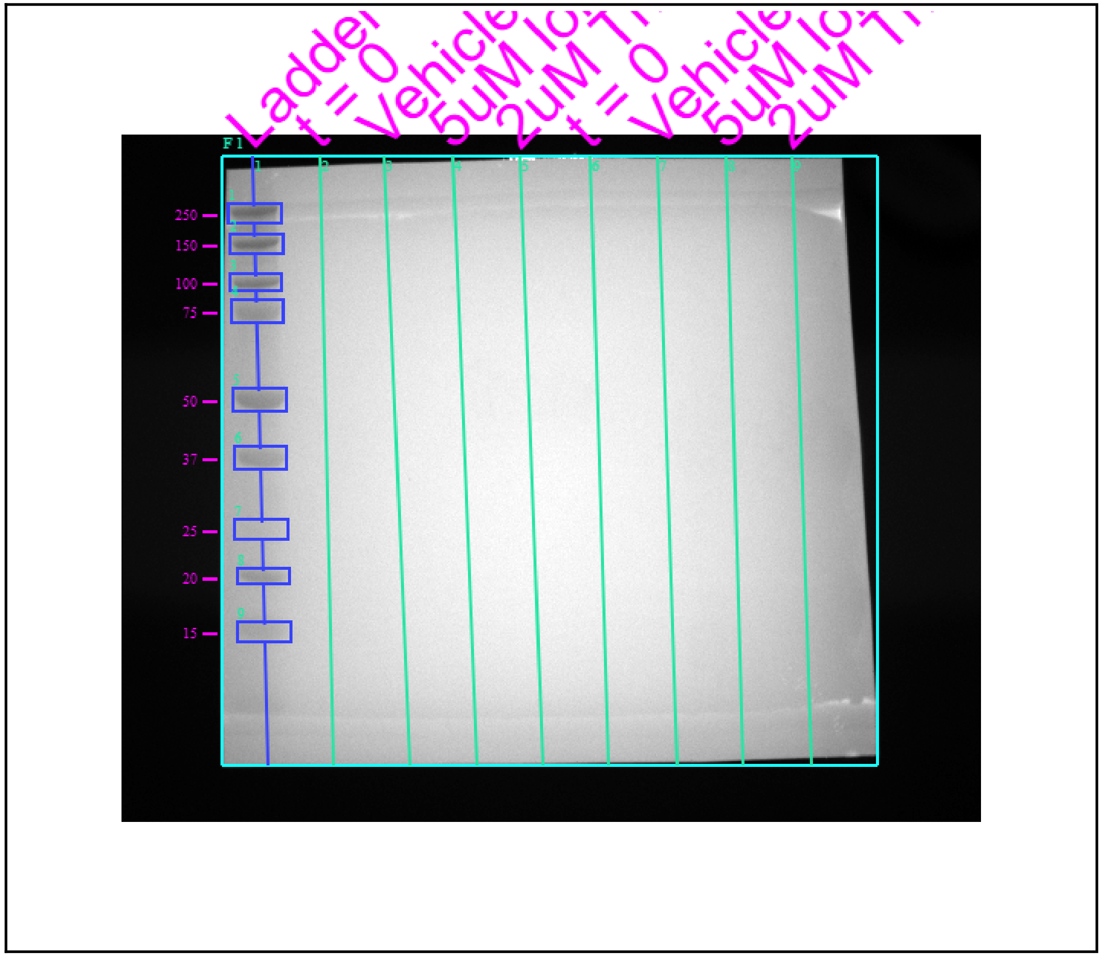

Total MLC_CHEMI_10052021_122334

Date:	5 October 2021 12:23:34PM
Mode:	Chemi Blots
Notes:	
Model:	FL1500
Instrument name:	2462619090234
Serial No:	2462619090234
Firmware version:	1.6.0
iBA version:	5.0
Image size:	563px X 450px
Image area:	112.7mm X 90.16mm
Optical Zoom:	2x
Digital Zoom:	1.2x
Focus level:	455
Resolution:	5 x 5
Exposure time:	10000 ms
Exposure mode:	Normal

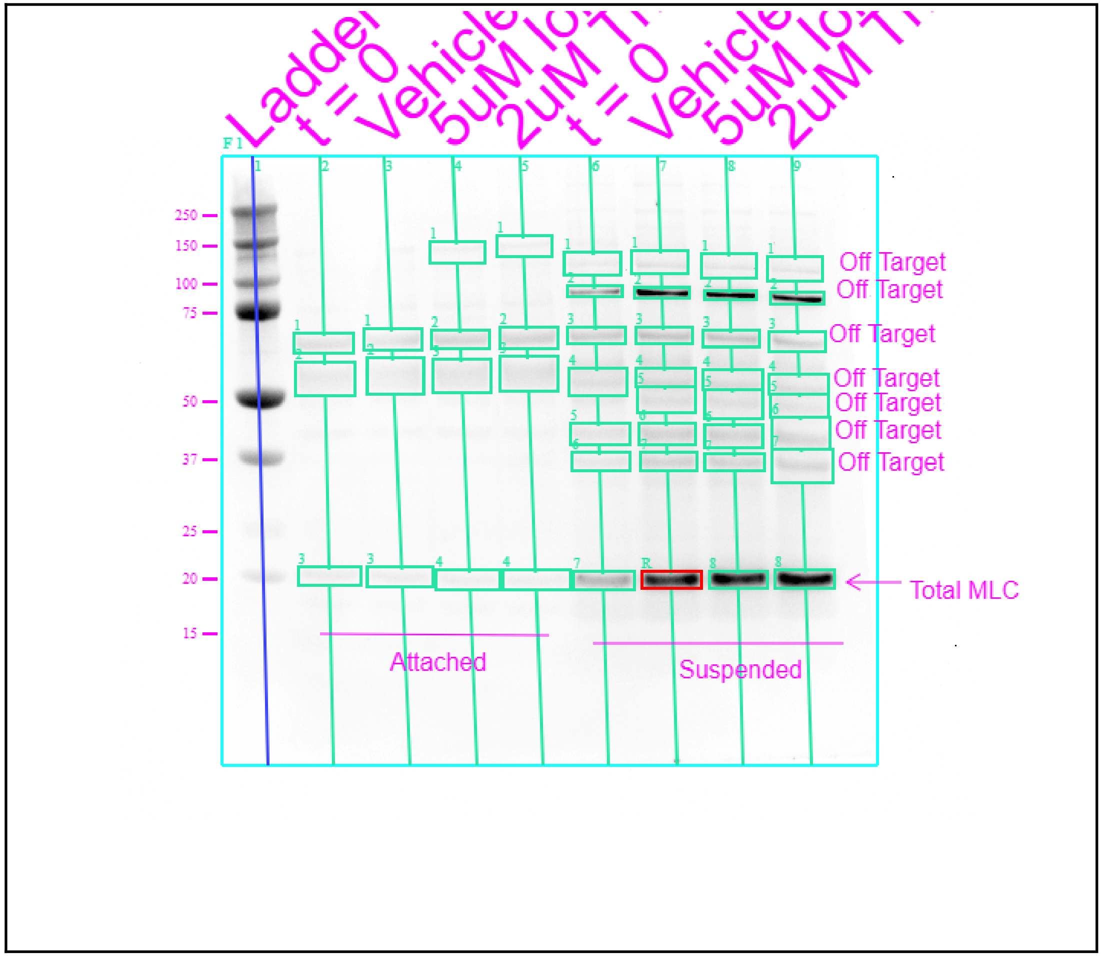

LANE AND BAND ANALYSIS DATA TABLE

Total MLC_CHEMI_10052021_122334

Frame: 1
Channel: Membrane
Sensitivity: 100
Molecular Weight Analysis Regression Method : Point to Point

Lane 1 - Ladder

#	Vol. (Int.)	Local Bg. Corr. Vol.	Area	Rf	Density	Local Bg. Corr. Den.	% band purity	% lane purity	Mol. Wt.
1	19,940,780	1,021,683	504	0.093	39,565	2,027.15	8.072	4.452	250
2	19,250,867	1,399,935	504	0.143	38,196	2,777.649	11.06	4.298	150
3	15,537,478	1,336,340	420	0.206	36,993	3,181.762	10.557	3.469	100
4	19,524,649	1,630,166	560	0.253	34,865	2,911.012	12.879	4.359	75
5	20,128,095	2,541,271	576	0.398	34,944	4,411.929	20.077	4.494	50
6	18,626,059	1,878,352	560	0.494	33,260	3,354.201	14.839	4.159	37
7	15,495,804	539,585	504	0.612	30,745	1,070.605	4.263	3.46	25
8	12,871,094	1,289,542	385	0.689	33,431	3,349.461	10.188	2.874	20
9	16,399,586	1,020,966	504	0.779	32,538	2,025.728	8.066	3.662	15

Frame: 1
Channel: Chemi
Sensitivity: 100
Molecular Weight Analysis Regression Method : Point to Point

Lane 2 - t = 0

#	Vol. (Int.)	Local Bg. Corr. Vol.	Area	Rf	Density	Local Bg. Corr. Den.	% band purity	% lane purity	Mol. Wt.	Rel. Quant. (w/ LB Corr. Vol.)
1	2,112,374	1,280,845	560	0.306	3,772.096	2,287.223	38.688	10.973	65.948	0.128
2	2,850,049	1,432,116	920	0.366	3,097.879	1,556.649	43.257	14.805	55.603	0.143
3	1,421,295	597,739	546	0.689	2,603.104	1,094.762	18.055	7.383	20	0.06

Lane 3 - Vehicle

#	Vol. (Int.)	Local Bg. Corr. Vol.	Area	Rf	Density	Local Bg. Corr. Den.	% band purity	% lane purity	Mol. Wt.	Rel. Quant. (w/ LB Corr. Vol.)
1	1,942,345	1,156,498	585	0.301	3,320.248	1,976.921	37.168	11.272	66.81	0.115
2	2,739,809	1,377,365	950	0.361	2,884.009	1,449.858	44.267	15.9	56.466	0.137

#	Vol. (Int.)	Local Bg. Corr. Vol.	Area	Rf	Density	Local Bg. Corr. Den.	% band purity	% lane purity	Mol. Wt.	Rel. Quant. (w/ LB Corr. Vol.)
3	1,476,294	577,660	616	0.689	2,396.581	937.76	18.565	8.567	20	0.058

Lane 4 - 5uM Ionomycin

#	Vol. (Int.)	Local Bg. Corr. Vol.	Area	Rf	Density	Local Bg. Corr. Den.	% band purity	% lane purity	Mol. Wt.	Rel. Quant. (w/ LB Corr. Vol.)
1	1,053,569	338,551	555	0.158	1,898.323	610.002	7.741	4.821	138	0.034
2	2,605,161	1,694,272	507	0.301	5,138.385	3,341.76	38.739	11.92	66.81	0.169
3	3,440,854	1,842,754	897	0.361	3,835.958	2,054.353	42.134	15.743	56.466	0.184
4	1,433,175	497,949	602	0.694	2,380.689	827.159	11.386	6.557	19.722	0.05

Lane 5 - 2uM Thapsigargin

#	Vol. (Int.)	Local Bg. Corr. Vol.	Area	Rf	Density	Local Bg. Corr. Den.	% band purity	% lane purity	Mol. Wt.	Rel. Quant. (w/ LB Corr. Vol.)
1	1,169,375	345,003	555	0.148	2,106.982	621.629	7.575	4.741	146	0.034
2	3,095,458	1,871,059	585	0.298	5,291.381	3,198.392	41.082	12.551	67.241	0.187
3	4,050,714	2,060,108	936	0.356	4,327.686	2,200.971	45.233	16.424	57.328	0.205
4	1,545,651	278,299	630	0.694	2,453.414	441.745	6.11	6.267	19.722	0.028

Lane 6 - t = 0

#	Vol. (Int.)	Local Bg. Corr. Vol.	Area	Rf	Density	Local Bg. Corr. Den.	% band purity	% lane purity	Mol. Wt.	Rel. Quant. (w/ LB Corr. Vol.)
1	1,745,217	502,307	608	0.175	2,870.423	826.164	5.213	4.295	124	0.05
2	2,738,267	1,991,820	296	0.221	9,250.902	6,729.122	20.67	6.739	92.105	0.199
3	2,946,130	1,621,267	468	0.293	6,295.15	3,464.247	16.824	7.25	68.103	0.162
4	3,817,111	1,029,219	741	0.371	5,151.297	1,388.961	10.681	9.394	54.741	0.103
5	2,513,573	932,975	600	0.456	4,189.288	1,554.959	9.682	6.186	42.132	0.093
6	1,871,521	576,984	507	0.501	3,691.363	1,138.036	5.988	4.606	36.234	0.058
7	4,944,904	2,981,817	533	0.697	9,277.493	5,594.404	30.943	12.169	19.583	0.297

Lane 7 - Vehicle

#	Vol. (Int.)	Local Bg. Corr. Vol.	Area	Rf	Density	Local Bg. Corr. Den.	% band purity	% lane purity	Mol. Wt.	Rel. Quant. (w/ LB Corr. Vol.)
1	2,271,350	792,688	608	0.173	3,735.773	1,303.764	3.315	3.534	126	0.079
2	7,714,184	6,672,781	296	0.223	26,061	22,543	27.905	12.002	90.789	0.666
3	3,400,095	1,797,173	456	0.293	7,456.349	3,941.171	7.516	5.29	68.103	0.179

#	Vol. (Int.)	Local Bg. Corr. Vol.	Area	Rf	Density	Local Bg. Corr. Den.	% band purity	% lane purity	Mol. Wt.	Rel. Quant. (w/ LB Corr. Vol.)
4	3,306,749	897,453	520	0.363	6,359.133	1,725.872	3.753	5.145	56.034	0.09
5	3,858,801	825,582	702	0.398	5,496.868	1,176.043	3.453	6.003	50	0.082
6	3,707,429	1,664,875	570	0.456	6,504.261	2,920.834	6.962	5.768	42.132	0.166
7	3,082,228	1,235,804	494	0.501	6,239.328	2,501.629	5.168	4.795	36.234	0.123
8	13,130,724	10,025,711	480	0.694	27,355	20,886	41.927	20.429	19.722	1

Lane 8 - 5uM Ionomycin

#	Vol. (Int.)	Local Bg. Corr. Vol.	Area	Rf	Density	Local Bg. Corr. Den.	% band purity	% lane purity	Mol. Wt.	Rel. Quant. (w/ LB Corr. Vol.)
1	2,148,224	580,596	629	0.18	3,415.3	923.047	2.529	3.485	120	0.058
2	7,039,208	5,938,584	245	0.228	28,731	24,239	25.868	11.418	88.158	0.592
3	3,174,609	1,586,331	456	0.298	6,961.862	3,478.796	6.91	5.149	67.241	0.158
4	3,271,459	862,417	560	0.366	5,841.891	1,540.031	3.757	5.307	55.603	0.086
5	3,970,573	732,529	780	0.406	5,090.478	939.141	3.191	6.441	48.974	0.073
6	3,933,127	1,685,548	624	0.459	6,303.088	2,701.2	7.342	6.38	41.789	0.168
7	2,946,291	1,179,796	480	0.501	6,138.106	2,457.909	5.139	4.779	36.234	0.118
8	13,703,870	10,391,574	468	0.694	29,281	22,204	45.265	22.228	19.722	1.036

Lane 9 - 2uM Thapsigargin

#	Vol. (Int.)	Local Bg. Corr. Vol.	Area	Rf	Density	Local Bg. Corr. Den.	% band purity	% lane purity	Mol. Wt.	Rel. Quant. (w/ LB Corr. Vol.)
1	1,926,106	635,340	629	0.185	3,062.172	1,010.081	2.509	3.474	116	0.063
2	7,073,611	6,122,114	324	0.233	21,832	18,895	24.178	12.759	85.526	0.611
3	2,759,885	1,409,189	532	0.303	5,187.754	2,648.852	5.565	4.978	66.379	0.141
4	2,714,454	834,765	560	0.373	4,847.239	1,490.653	3.297	4.896	54.31	0.083
5	2,747,998	644,923	624	0.409	4,403.843	1,033.532	2.547	4.957	48.632	0.064
6	4,196,928	1,682,181	858	0.454	4,891.524	1,960.584	6.643	7.57	42.474	0.168
7	4,369,172	1,377,683	943	0.509	4,633.268	1,460.958	5.441	7.881	35.468	0.137
8	15,326,781	12,614,988	520	0.694	29,474	24,259	49.82	27.646	19.722	1.258

iBright™ Image Analysis Report

Katarina+ Chang

18 November 2022

Total MYPT1_CHEMI_10062021_131104

Date:	6 October 2021 01:11:04PM
Mode:	Chemi Blots
Notes:	
Model:	FL1500
Instrument name:	2462619090234
Serial No:	2462619090234
Firmware version:	1.6.0
iBA version:	5.0
Image size:	676px X 540px
Image area:	118.63mm X 94.91mm
Optical Zoom:	1.9x
Digital Zoom:	1x
Focus level:	430
Resolution:	5 x 5
Exposure time:	7859 ms
Exposure mode:	Normal

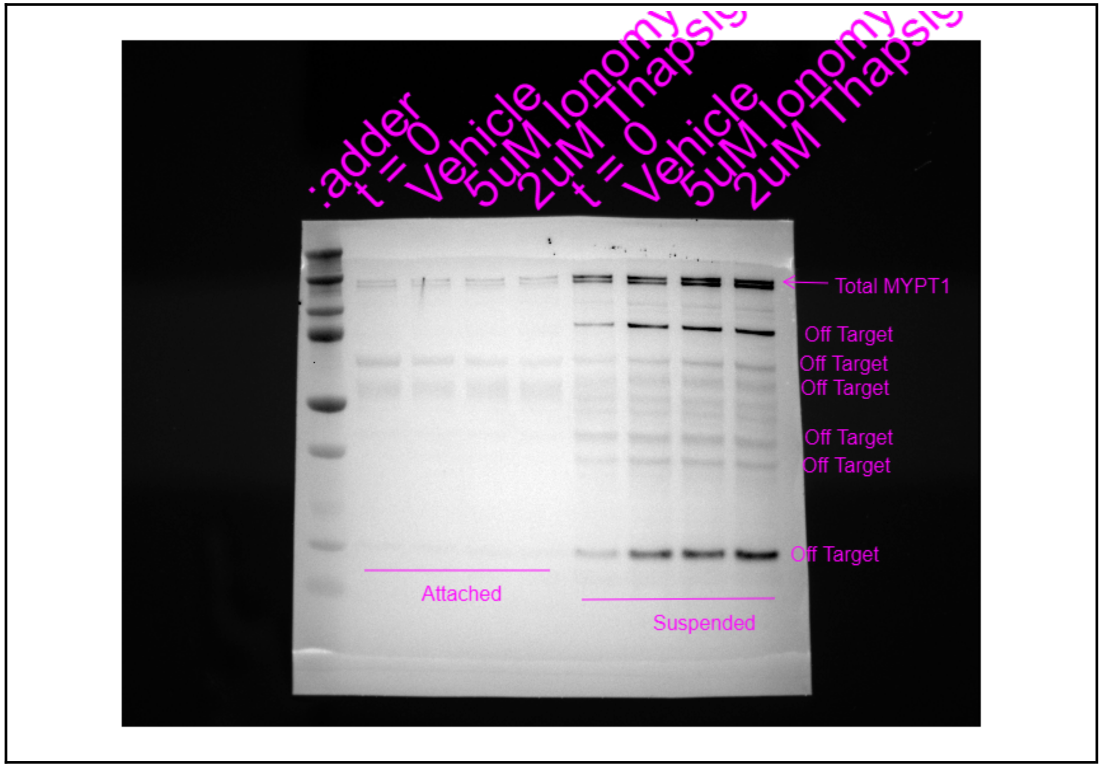

Total MYPT1_CHEMI_10062021_131104

Date:	6 October 2021 01:11:04PM
Mode:	Chemi Blots
Notes:	
Model:	FL1500
Instrument name:	2462619090234
Serial No:	2462619090234
Firmware version:	1.6.0
iBA version:	5.0
Image size:	676px X 540px
Image area:	118.63mm X 94.91mm
Optical Zoom:	1.9x
Digital Zoom:	1x
Focus level:	430
Resolution:	5 x 5
Exposure time:	7859 ms
Exposure mode:	Normal

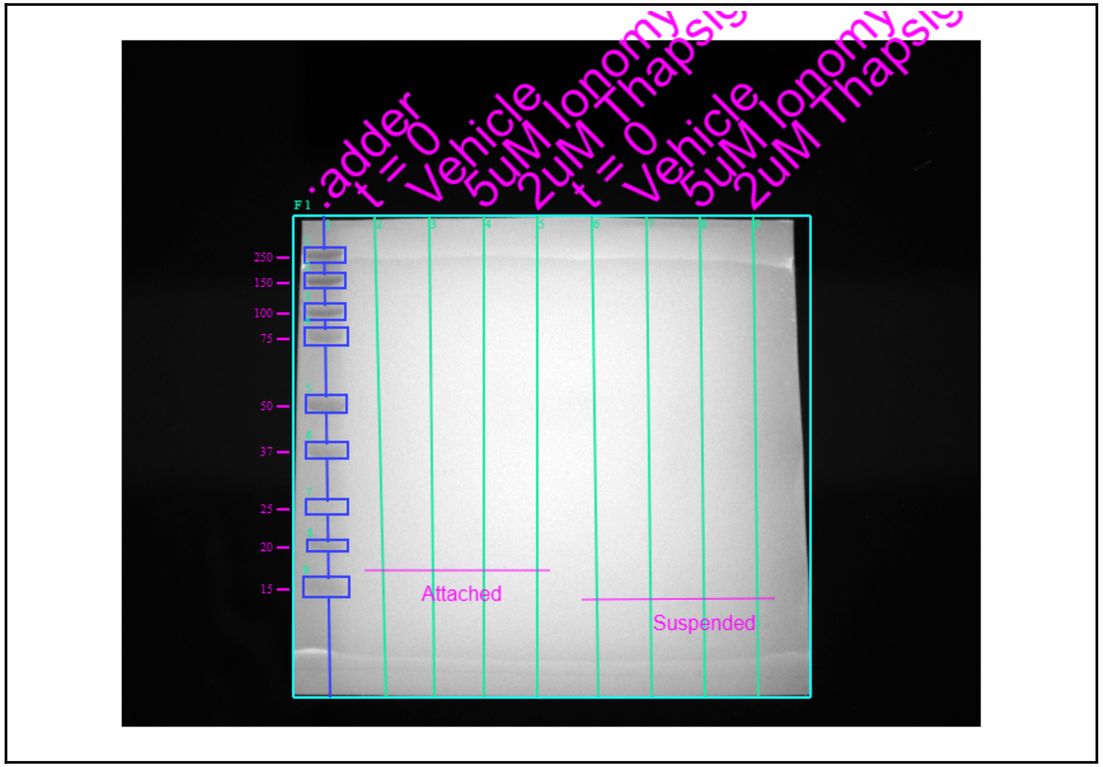

Total MYPT1_CHEMI_10062021_131104

Date:	6 October 2021 01:11:04PM
Mode:	Chemi Blots
Notes:	
Model:	FL1500
Instrument name:	2462619090234
Serial No:	2462619090234
Firmware version:	1.6.0
iBA version:	5.0
Image size:	676px X 540px
Image area:	118.63mm X 94.91mm
Optical Zoom:	1.9x
Digital Zoom:	1x
Focus level:	430
Resolution:	5 x 5
Exposure time:	7859 ms
Exposure mode:	Normal

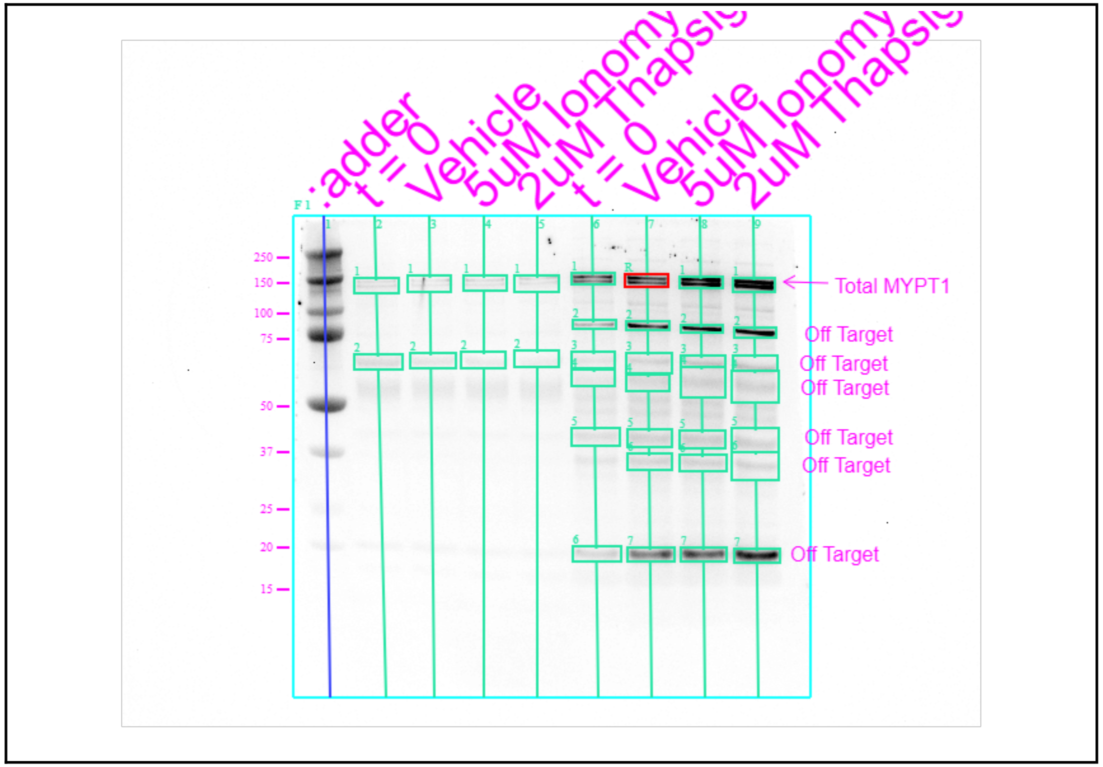

LANE AND BAND ANALYSIS DATA TABLE

Total MYPT1_CHEMI_10062021_131104

Frame: 1
Channel: Membrane
Sensitivity: 100
Molecular Weight Analysis Regression Method : Point to Point

Lane 1 - :adder

#	Vol. (Int.)	Local Bg. Corr. Vol.	Area	Rf	Density	Local Bg. Corr. Den.	% band purity	% lane purity	Mol. Wt.
1	15,794,085	941,194	429	0.082	36,816	2,193.927	8.24	3.951	250
2	15,715,335	1,306,364	429	0.135	36,632	3,045.138	11.437	3.932	150
3	16,019,106	1,407,167	462	0.198	34,673	3,045.818	12.32	4.008	100
4	17,206,584	1,437,478	525	0.251	32,774	2,738.054	12.585	4.305	75
5	16,671,529	2,076,661	495	0.391	33,679	4,195.276	18.181	4.171	50
6	15,378,980	1,584,016	476	0.485	32,308	3,327.765	13.868	3.847	37
7	13,344,815	478,693	442	0.604	30,191	1,083.016	4.191	3.339	25
8	10,746,693	864,606	330	0.683	32,565	2,620.019	7.57	2.689	20
9	20,436,436	1,325,690	629	0.77	32,490	2,107.615	11.607	5.113	15

Frame: 1
Channel: Chemi
Sensitivity: 100
Molecular Weight Analysis Regression Method : Point to Point

Lane 2 - t = 0

#	Vol. (Int.)	Local Bg. Corr. Vol.	Area	Rf	Density	Local Bg. Corr. Den.	% band purity	% lane purity	Mol. Wt.	Rel. Quant. (w/ LB Corr. Vol.)
1	1,144,332	563,104	468	0.145	2,445.154	1,203.215	45.269	11.625	141.667	0.159
2	1,242,143	680,815	507	0.303	2,449.986	1,342.832	54.731	12.619	65.566	0.192

Lane 3 - Vehicle

#	Vol. (Int.)	Local Bg. Corr. Vol.	Area	Rf	Density	Local Bg. Corr. Den.	% band purity	% lane purity	Mol. Wt.	Rel. Quant. (w/ LB Corr. Vol.)
1	1,106,438	624,385	490	0.14	2,258.037	1,274.257	59.287	12.566	145.833	0.176
2	867,594	428,780	481	0.301	1,803.73	891.435	40.713	9.853	66.038	0.121

Lane 4 - 5uM Ionomycin

#	Vol. (Int.)	Local Bg. Corr. Vol.	Area	Rf	Density	Local Bg. Corr. Den.	% band purity	% lane purity	Mol. Wt.	Rel. Quant. (w/ LB Corr. Vol.)
1	992,784	558,963	455	0.14	2,181.943	1,228.492	64.639	10.905	145.833	0.157
2	743,341	305,784	504	0.298	1,474.883	606.716	35.361	8.165	66.509	0.086

Lane 5 - 2uM Thapsigargin

#	Vol. (Int.)	Local Bg. Corr. Vol.	Area	Rf	Density	Local Bg. Corr. Den.	% band purity	% lane purity	Mol. Wt.	Rel. Quant. (w/ LB Corr. Vol.)
1	1,150,808	558,929	504	0.14	2,283.349	1,108.988	70.951	10.491	145.833	0.157
2	751,858	228,843	504	0.296	1,491.782	454.055	29.049	6.854	66.981	0.064

Lane 6 - t = 0

#	Vol. (Int.)	Local Bg. Corr. Vol.	Area	Rf	Density	Local Bg. Corr. Den.	% band purity	% lane purity	Mol. Wt.	Rel. Quant. (w/ LB Corr. Vol.)
1	3,557,264	2,711,267	350	0.129	10,163	7,746.478	51.14	16.85	160	0.764
2	1,177,679	662,131	280	0.224	4,205.996	2,364.757	12.489	5.578	87.5	0.187
3	1,074,994	306,051	490	0.298	2,193.865	624.594	5.773	5.092	66.509	0.086
4	1,169,778	279,191	490	0.335	2,387.302	569.778	5.266	5.541	59.906	0.079
5	1,397,752	580,312	585	0.459	2,389.32	991.987	10.946	6.621	40.611	0.163
6	1,325,202	762,717	507	0.702	2,613.811	1,504.375	14.386	6.277	18.939	0.215

Lane 7 - Vehicle

#	Vol. (Int.)	Local Bg. Corr. Vol.	Area	Rf	Density	Local Bg. Corr. Den.	% band purity	% lane purity	Mol. Wt.	Rel. Quant. (w/ LB Corr. Vol.)
1	4,703,203	3,549,402	385	0.135	12,216	9,219.228	31.613	14.197	150	1
2	3,088,984	2,281,199	280	0.227	11,032	8,147.142	20.317	9.324	86.25	0.643
3	2,013,599	522,183	629	0.306	3,201.27	830.181	4.651	6.078	65.094	0.147
4	1,780,770	526,674	490	0.346	3,634.224	1,074.845	4.691	5.375	58.019	0.148
5	1,890,176	831,811	540	0.462	3,500.326	1,540.391	7.408	5.706	40.25	0.234
6	1,570,368	621,323	504	0.509	3,115.81	1,232.786	5.534	4.74	34.6	0.175
7	3,826,272	2,895,229	456	0.702	8,390.947	6,349.188	25.786	11.55	18.939	0.816

Lane 8 - 5uM Ionomycin

#	Vol. (Int.)	Local Bg. Corr. Vol.	Area	Rf	Density	Local Bg. Corr. Den.	% band purity	% lane purity	Mol. Wt.	Rel. Quant. (w/ LB Corr. Vol.)
1	7,180,565	5,735,373	350	0.137	20,515	16,386	38.944	18.04	147.917	1.616
2	3,538,203	2,706,580	238	0.235	14,866	11,372	18.378	8.889	82.5	0.763

#	Vol. (Int.)	Local Bg. Corr. Vol.	Area	Rf	Density	Local Bg. Corr. Den.	% band purity	% lane purity	Mol. Wt.	Rel. Quant. (w/ LB Corr. Vol.)
3	1,572,495	672,227	360	0.301	4,368.042	1,867.298	4.565	3.951	66.038	0.189
4	3,366,781	570,362	900	0.346	3,740.868	633.736	3.873	8.458	58.019	0.161
5	2,133,897	974,205	555	0.464	3,844.859	1,755.326	6.615	5.361	39.889	0.274
6	1,708,917	674,876	532	0.512	3,212.25	1,268.565	4.583	4.293	34.333	0.19
7	4,462,934	3,393,592	444	0.702	10,051	7,643.227	23.043	11.212	18.939	0.956

Lane 9 - 2uM Thapsigargin

#	Vol. (Int.)	Local Bg. Corr. Vol.	Area	Rf	Density	Local Bg. Corr. Den.	% band purity	% lane purity	Mol. Wt.	Rel. Quant. (w/ LB Corr. Vol.)
1	8,167,349	6,878,036	408	0.142	20,018	16,857	36.742	20.5	143.75	1.938
2	4,551,929	3,744,632	306	0.243	14,875	12,237	20.004	11.425	78.75	1.055
3	1,793,817	796,584	468	0.306	3,832.942	1,702.104	4.255	4.502	65.094	0.224
4	3,342,393	734,008	988	0.354	3,382.989	742.924	3.921	8.389	56.604	0.207
5	2,237,393	959,340	760	0.464	2,943.938	1,262.291	5.125	5.616	39.889	0.27
6	2,177,944	703,072	874	0.52	2,491.927	804.43	3.756	5.467	33.533	0.198
7	5,855,605	4,904,119	444	0.704	13,188	11,045	26.198	14.697	18.788	1.382

iBright™ Image Analysis Report

Katarina+ Chang
18 November 2022

Acetyl_tubulin_CHEMI_10072021_123705

Date:	7 October 2021 12:37:05PM
Mode:	Chemi Blots
Notes:	
Model:	FL1500
Instrument name:	2462619090234
Serial No:	2462619090234
Firmware version:	1.6.0
iBA version:	5.0
Image size:	563px X 450px
Image area:	112.7mm X 90.16mm
Optical Zoom:	2x
Digital Zoom:	1.2x
Focus level:	455
Resolution:	5 x 5
Exposure time:	1795 ms
Exposure mode:	Normal

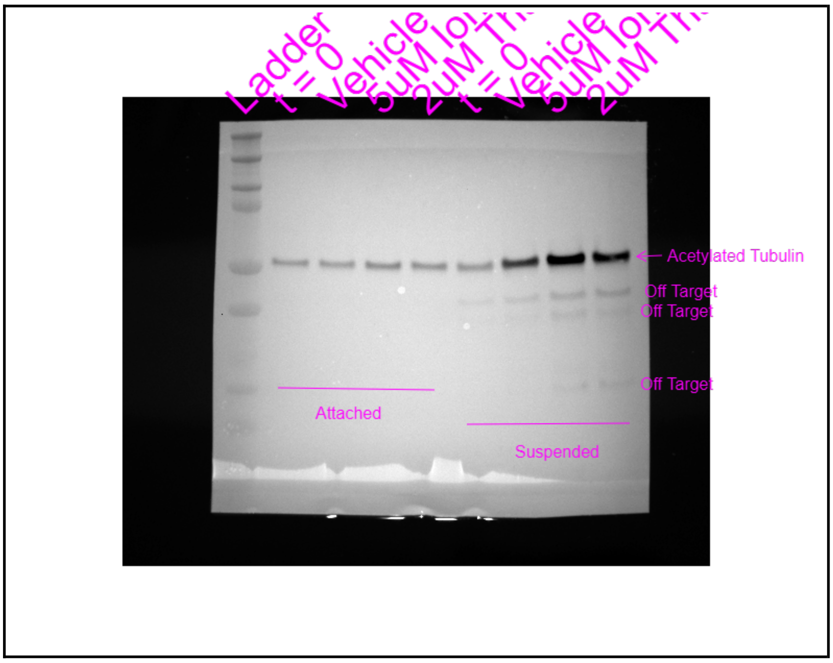

Acetyl_tubulin_CHEMI_10072021_123705

Date:	7 October 2021 12:37:05PM
Mode:	Chemi Blots
Notes:	
Model:	FL1500
Instrument name:	2462619090234
Serial No:	2462619090234
Firmware version:	1.6.0
iBA version:	5.0
Image size:	563px X 450px
Image area:	112.7mm X 90.16mm
Optical Zoom:	2x
Digital Zoom:	1.2x
Focus level:	455
Resolution:	5 x 5
Exposure time:	1795 ms
Exposure mode:	Normal

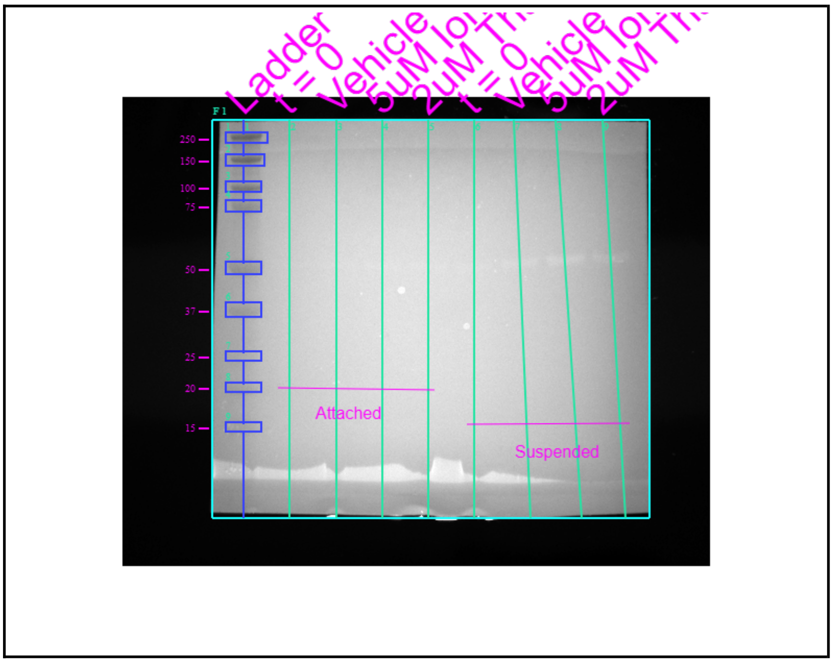

Acetyl_tubulin_CHEMI_10072021_123705

Date:	7 October 2021 12:37:05PM
Mode:	Chemi Blots
Notes:	
Model:	FL1500
Instrument name:	2462619090234
Serial No:	2462619090234
Firmware version:	1.6.0
iBA version:	5.0
Image size:	563px X 450px
Image area:	112.7mm X 90.16mm
Optical Zoom:	2x
Digital Zoom:	1.2x
Focus level:	455
Resolution:	5 x 5
Exposure time:	1795 ms
Exposure mode:	Normal

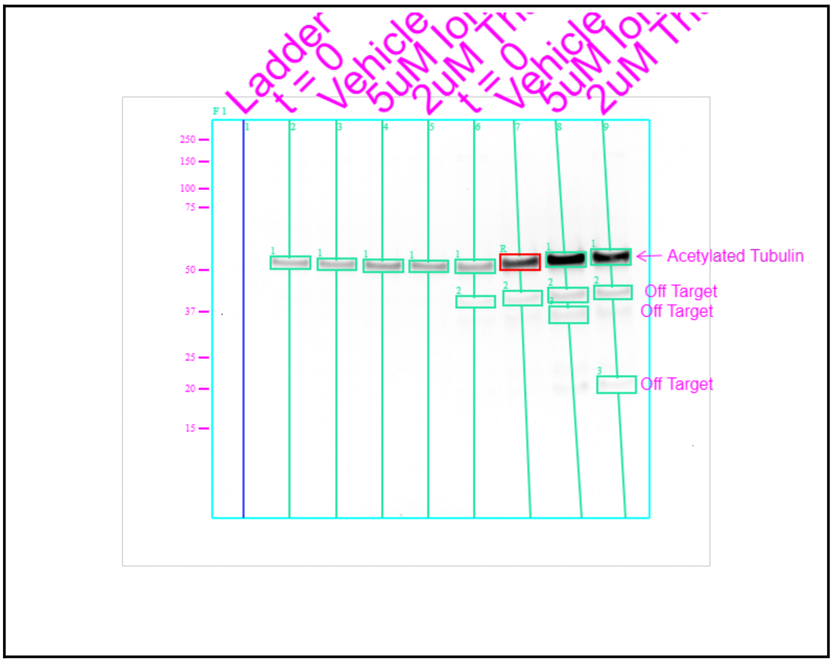

LANE AND BAND ANALYSIS DATA TABLE

Acetyl_tubulin_CHEMI_10072021_123705

Frame: 1
Channel: Membrane
Sensitivity: 100
Molecular Weight Analysis Regression Method : Point to Point

Lane 1 - Ladder

#	Vol. (Int.)	Local Bg. Corr. Vol.	Area	Rf	Density
1	17,371,087	2,017,379	451	0.045	38,516
2	16,947,283	1,958,756	456	0.099	37,165
3	13,860,499	1,548,884	385	0.168	36,001
4	14,406,123	1,302,986	420	0.215	34,300
5	15,097,641	1,522,438	455	0.372	33,181
6	16,676,770	1,411,328	525	0.476	31,765
7	10,507,806	374,626	350	0.592	30,022
8	10,933,808	575,491	350	0.67	31,239
9	10,993,416	419,258	350	0.77	31,409

#	Local Bg. Corr. Den.	% band purity	% lane purity	Mol. Wt.
1	4,473.125	18.124	3.976	250
2	4,295.518	17.597	3.879	150
3	4,023.078	13.915	3.172	100
4	3,102.349	11.706	3.297	75
5	3,346.018	13.677	3.456	50
6	2,688.245	12.679	3.817	37
7	1,070.361	3.366	2.405	25
8	1,644.262	5.17	2.503	20
9	1,197.881	3.767	2.516	15

Frame: 1
Channel: Chemi
Sensitivity: 100
Molecular Weight Analysis Regression Method : Point to Point

Lane 2 - t = 0

#	Vol. (Int.)	Local Bg. Corr. Vol.	Area	Rf	Density
1	2,227,099	1,986,144	507	0.359	4,392.7

#	Local Bg. Corr. Den.	% band purity	% lane purity	Mol. Wt.	Rel. Quant. (w/ LB Corr. Vol.)
1	3,917.445	100	54.784	52.083	0.274

Lane 3 - Vehicle

#	Vol. (Int.)	Local Bg. Corr. Vol.	Area	Rf	Density
1	2,361,212	2,044,706	456	0.361	5,178.096

#	Local Bg. Corr. Den.	% band purity	% lane purity	Mol. Wt.	Rel. Quant. (w/ LB Corr. Vol.)
1	4,484.004	100	50.095	51.667	0.282

Lane 4 - 5uM Ionomycin

#	Vol. (Int.)	Local Bg. Corr. Vol.	Area	Rf	Density
1	3,282,375	2,877,802	507	0.366	6,474.112

#	Local Bg. Corr. Den.	% band purity	% lane purity	Mol. Wt.	Rel. Quant. (w/ LB Corr. Vol.)
1	5,676.139	100	54.301	50.833	0.397

Lane 5 - 2uM Thapsigargin

#	Vol. (Int.)	Local Bg. Corr. Vol.	Area	Rf	Density
1	3,017,786	2,571,962	456	0.366	6,617.952

#	Local Bg. Corr. Den.	% band purity	% lane purity	Mol. Wt.	Rel. Quant. (w/ LB Corr. Vol.)
1	5,640.268	100	47.876	50.833	0.355

Lane 6 - t = 0

#	Vol. (Int.)	Local Bg. Corr. Vol.	Area	Rf	Density
1	3,231,195	2,585,212	546	0.366	5,917.94
2	578,059	307,872	456	0.455	1,267.673

#	Local Bg. Corr. Den.	% band purity	% lane purity	Mol. Wt.	Rel. Quant. (w/ LB Corr. Vol.)
1	4,734.821	89.358	41.357	50.833	0.357
2	675.159	10.642	7.399	39.6	0.042

Lane 7 - Vehicle

#	Vol. (Int.)	Local Bg. Corr. Vol.	Area	Rf	Density
1	8,459,189	7,249,197	624	0.356	13,556
2	1,071,688	545,345	570	0.448	1,880.154

#	Local Bg. Corr. Den.	% band purity	% lane purity	Mol. Wt.	Rel. Quant. (w/ LB Corr. Vol.)
1	11,617	93.003	56.618	52.5	1
2	956.746	6.996	7.173	40.575	0.075

Lane 8 - 5uM Ionomycin

#	Vol. (Int.)	Local Bg. Corr. Vol.	Area	Rf	Density
1	15,411,937	13,454,831	585	0.351	26,345
2	1,732,759	1,046,594	585	0.44	2,961.981
3	1,314,919	666,217	646	0.49	2,035.478

#	Local Bg. Corr. Den.	% band purity	% lane purity	Mol. Wt.	Rel. Quant. (w/ LB Corr. Vol.)
1	22,999	88.707	61.935	53.333	1.856
2	1,789.05	6.9	6.963	41.55	0.144
3	1,031.296	4.392	5.284	35.636	0.092

Lane 9 - 2uM Thapsigargin

#	Vol. (Int.)	Local Bg. Corr. Vol.	Area	Rf	Density
1	11,557,801	10,170,948	624	0.343	18,522
2	1,610,461	1,095,873	518	0.432	3,108.998
3	620,506	443,252	646	0.665	960.536

#	Local Bg. Corr. Den.	% band purity	% lane purity	Mol. Wt.	Rel. Quant. (w/ LB Corr. Vol.)
1	16,299	86.856	58.36	54.583	1.403
2	2,115.586	9.358	8.132	42.525	0.151
3	686.15	3.785	3.133	20.333	0.061

iBright™ Image Analysis Report

Katarina+ Chang

18 November 2022

GAPDH_CHEMI_10082021_124202

Date:	8 October 2021 12:42:02PM
Mode:	Chemi Blots
Notes:	
Model:	FL1500
Instrument name:	2462619090234
Serial No:	2462619090234
Firmware version:	1.6.0
iBA version:	5.0
Image size:	563px X 450px
Image area:	112.7mm X 90.16mm
Optical Zoom:	2x
Digital Zoom:	1.2x
Focus level:	455
Resolution:	5 x 5
Exposure time:	1058 ms
Exposure mode:	Normal

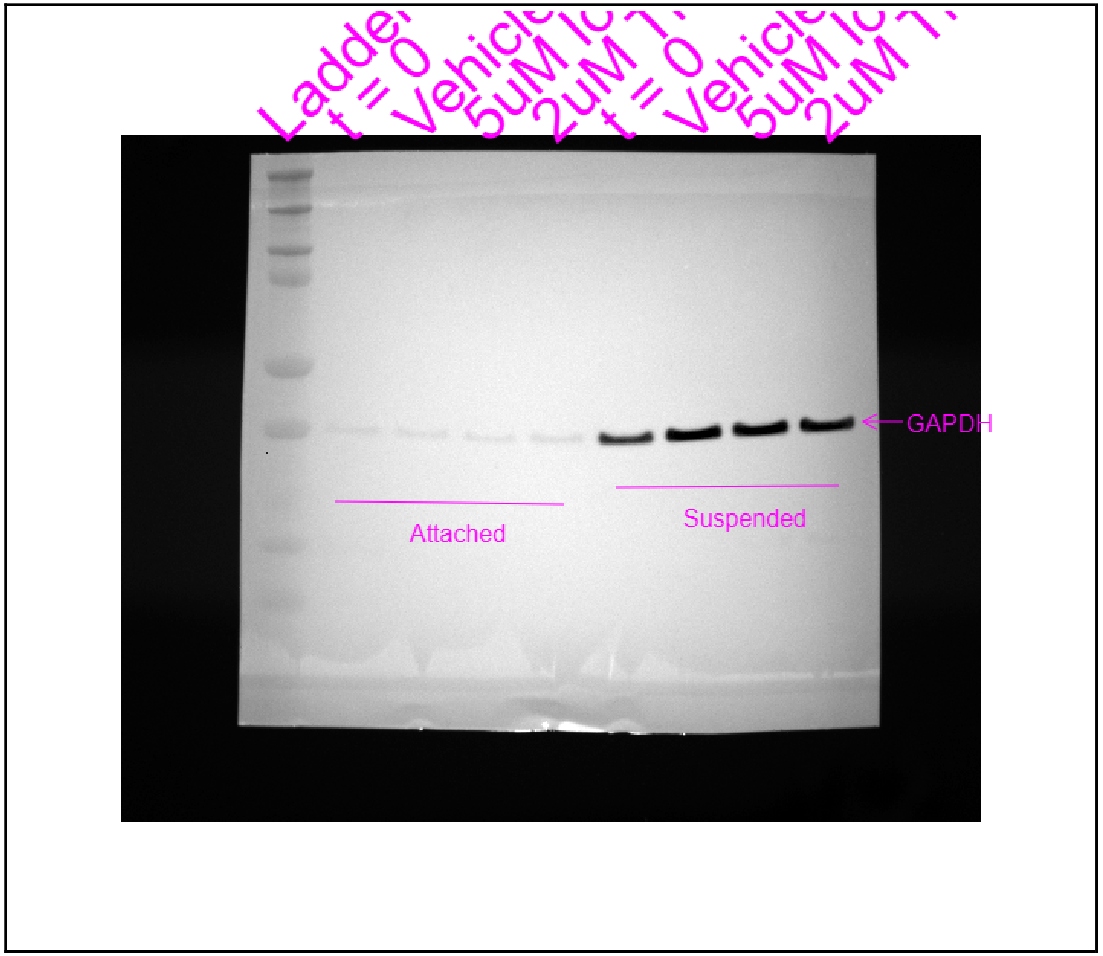

GAPDH_CHEMI_10082021_124202

Date:	8 October 2021 12:42:02PM
Mode:	Chemi Blots
Notes:	
Model:	FL1500
Instrument name:	2462619090234
Serial No:	2462619090234
Firmware version:	1.6.0
iBA version:	5.0
Image size:	563px X 450px
Image area:	112.7mm X 90.16mm
Optical Zoom:	2x
Digital Zoom:	1.2x
Focus level:	455
Resolution:	5 x 5
Exposure time:	1058 ms
Exposure mode:	Normal

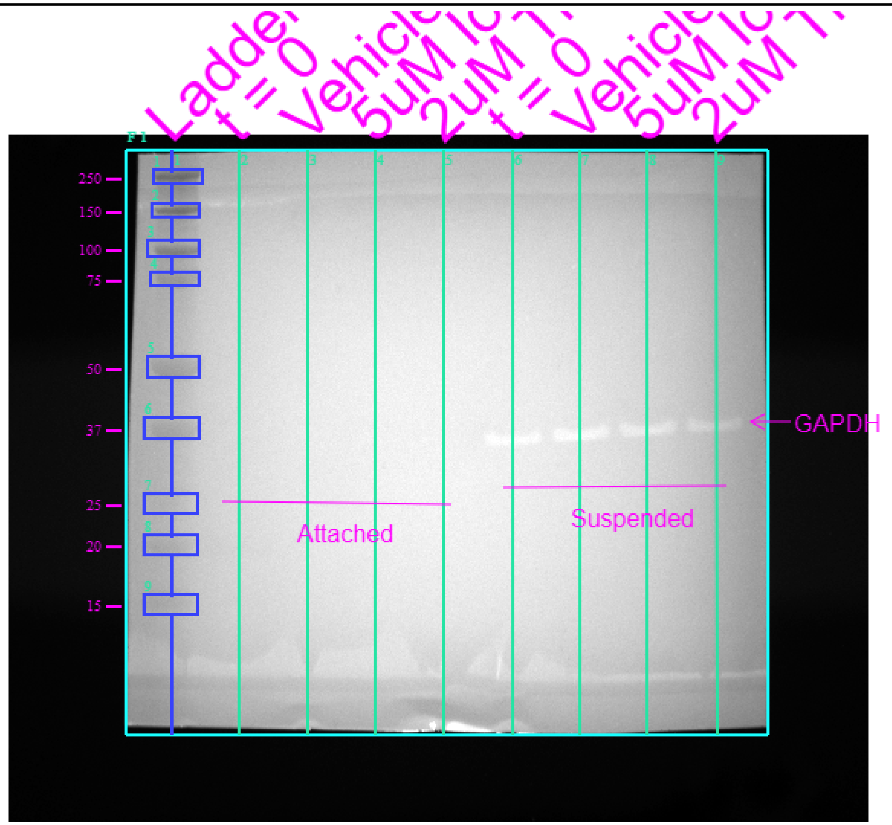

GAPDH_CHEMI_10082021_124202

Date:	8 October 2021 12:42:02PM
Mode:	Chemi Blots
Notes:	
Model:	FL1500
Instrument name:	2462619090234
Serial No:	2462619090234
Firmware version:	1.6.0
iBA version:	5.0
Image size:	563px X 450px
Image area:	112.7mm X 90.16mm
Optical Zoom:	2x
Digital Zoom:	1.2x
Focus level:	455
Resolution:	5 x 5
Exposure time:	1058 ms
Exposure mode:	Normal

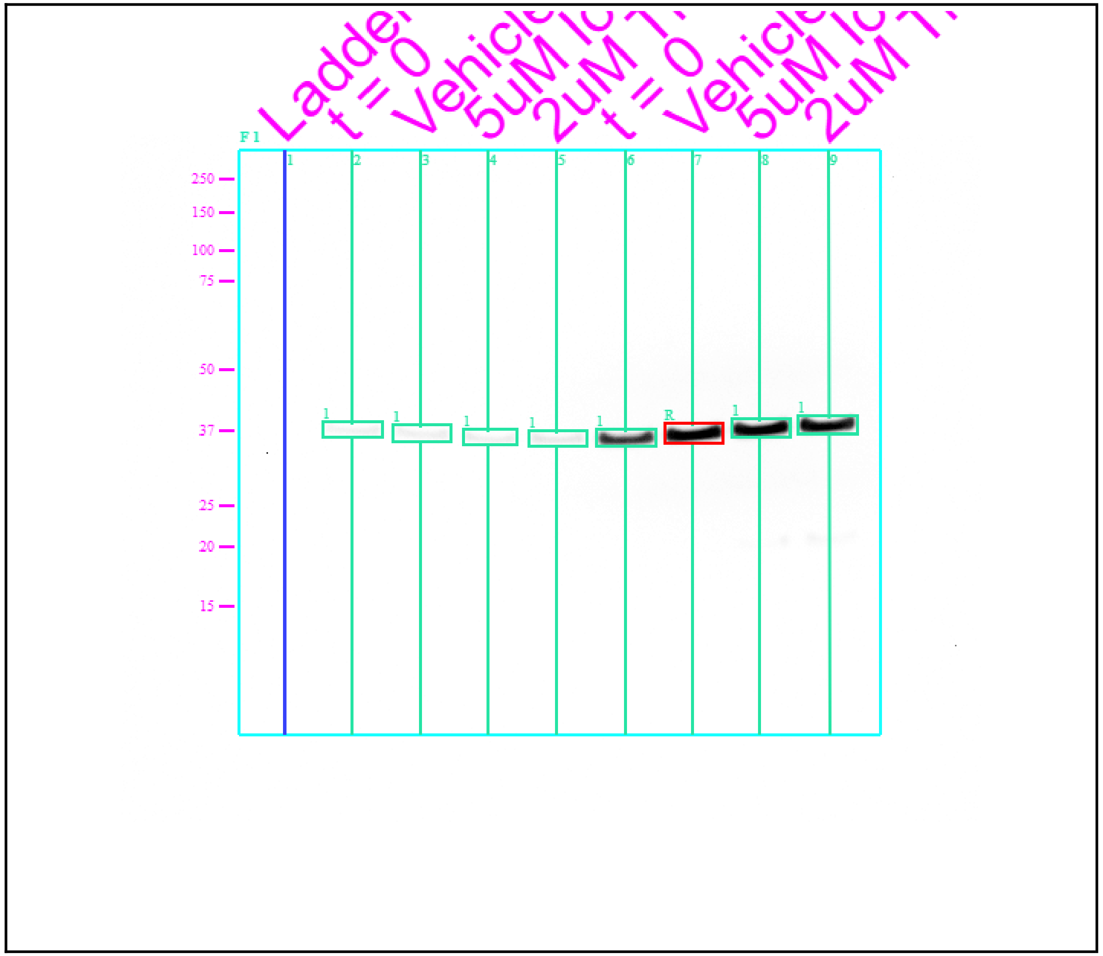

LANE AND BAND ANALYSIS DATA TABLE

GAPDH_CHEMI_10082021_124202

Frame: 1
Channel: Membrane
Sensitivity: 100
Molecular Weight Analysis Regression Method : Point to Point

Lane 1 - Ladder

#	Vol. (Int.)	Local Bg. Corr. Vol.	Area	Rf	Density	Local Bg. Corr. Den.	% band purity	% lane purity	Mol. Wt.
1	13,207,434	1,674,359	330	0.044	40,022	5,073.815	15.48	-0	250
2	12,125,851	1,460,554	320	0.102	37,893	4,564.232	13.503	-0	150
3	14,974,651	1,564,453	420	0.167	35,653	3,724.888	14.463	-0	100
4	11,274,197	1,005,480	330	0.219	34,164	3,046.911	9.296	-0	75
5	17,149,811	1,624,101	525	0.371	32,666	3,093.526	15.015	-0	50
6	17,548,050	1,408,408	555	0.475	31,618	2,537.673	13.021	-0	37
7	15,064,375	531,283	504	0.603	29,889	1,054.133	4.912	-0	25
8	15,623,193	834,824	504	0.674	30,998	1,656.398	7.718	-0	20
9	15,913,735	713,132	504	0.775	31,574	1,414.944	6.593	-0	15

Frame: 1
Channel: Chemi
Sensitivity: 100
Molecular Weight Analysis Regression Method : Point to Point

Lane 2 - t = 0

#	Vol. (Int.)	Local Bg. Corr. Vol.	Area	Rf	Density	Local Bg. Corr. Den.	% band purity	% lane purity	Mol. Wt.	Rel. Quant. (w/ LB Corr. Vol.)
1	432,972	387,858	440	0.478	984.027	881.497	100	-0	36.755	0.036

Lane 3 - Vehicle

#	Vol. (Int.)	Local Bg. Corr. Vol.	Area	Rf	Density	Local Bg. Corr. Den.	% band purity	% lane purity	Mol. Wt.	Rel. Quant. (w/ LB Corr. Vol.)
1	487,384	438,955	468	0.483	1,041.419	937.939	100	-0	36.265	0.04

Lane 4 - 5uM Ionomycin

#	Vol. (Int.)	Local Bg. Corr. Vol.	Area	Rf	Density	Local Bg. Corr. Den.	% band purity	% lane purity	Mol. Wt.	Rel. Quant. (w/ LB Corr. Vol.)

#	Vol. (Int.)	Local Bg. Corr. Vol.	Area	Rf	Density	Local Bg. Corr. Den.	% band purity	% lane purity	Mol. Wt.	Rel. Quant. (w/ LB Corr. Vol.)
1	467,226	416,072	396	0.491	1,179.864	1,050.687	100	-0	35.531	0.038

Lane 5 - 2uM Thapsigargin

#	Vol. (Int.)	Local Bg. Corr. Vol.	Area	Rf	Density	Local Bg. Corr. Den.	% band purity	% lane purity	Mol. Wt.	Rel. Quant. (w/ LB Corr. Vol.)
1	712,364	579,158	429	0.493	1,660.522	1,350.021	100	-0	35.286	0.053

Lane 6 - t = 0

#	Vol. (Int.)	Local Bg. Corr. Vol.	Area	Rf	Density	Local Bg. Corr. Den.	% band purity	% lane purity	Mol. Wt.	Rel. Quant. (w/ LB Corr. Vol.)
1	7,387,436	7,008,230	480	0.491	15,390	14,600	100	-0	35.531	0.642

Lane 7 - Vehicle

#	Vol. (Int.)	Local Bg. Corr. Vol.	Area	Rf	Density	Local Bg. Corr. Den.	% band purity	% lane purity	Mol. Wt.	Rel. Quant. (w/ LB Corr. Vol.)
1	11,470,852	10,916,309	546	0.483	21,008	19,993	100	-0	36.265	1

Lane 8 - 5uM Ionomycin

#	Vol. (Int.)	Local Bg. Corr. Vol.	Area	Rf	Density	Local Bg. Corr. Den.	% band purity	% lane purity	Mol. Wt.	Rel. Quant. (w/ LB Corr. Vol.)
1	11,644,217	11,037,848	507	0.475	22,966	21,770	100	-0	37	1.011

Lane 9 - 2uM Thapsigargin

#	Vol. (Int.)	Local Bg. Corr. Vol.	Area	Rf	Density	Local Bg. Corr. Den.	% band purity	% lane purity	Mol. Wt.	Rel. Quant. (w/ LB Corr. Vol.)
1	10,304,514	9,943,596	520	0.47	19,816	19,122	100	-0	37.65	0.911

iBright™ Image Analysis Report

Katarina+ Chang

18 November 2022

pMLC_CHEMI_10062021_131628

Date:	6 October 2021 01:16:28PM
Mode:	Chemi Blots
Notes:	
Model:	FL1500
Instrument name:	2462619090234
Serial No:	2462619090234
Firmware version:	1.6.0
iBA version:	5.0
Image size:	520px X 415px
Image area:	112.7mm X 90.16mm
Optical Zoom:	2x
Digital Zoom:	1.3x
Focus level:	455
Resolution:	5 x 5
Exposure time:	6773 ms
Exposure mode:	Normal

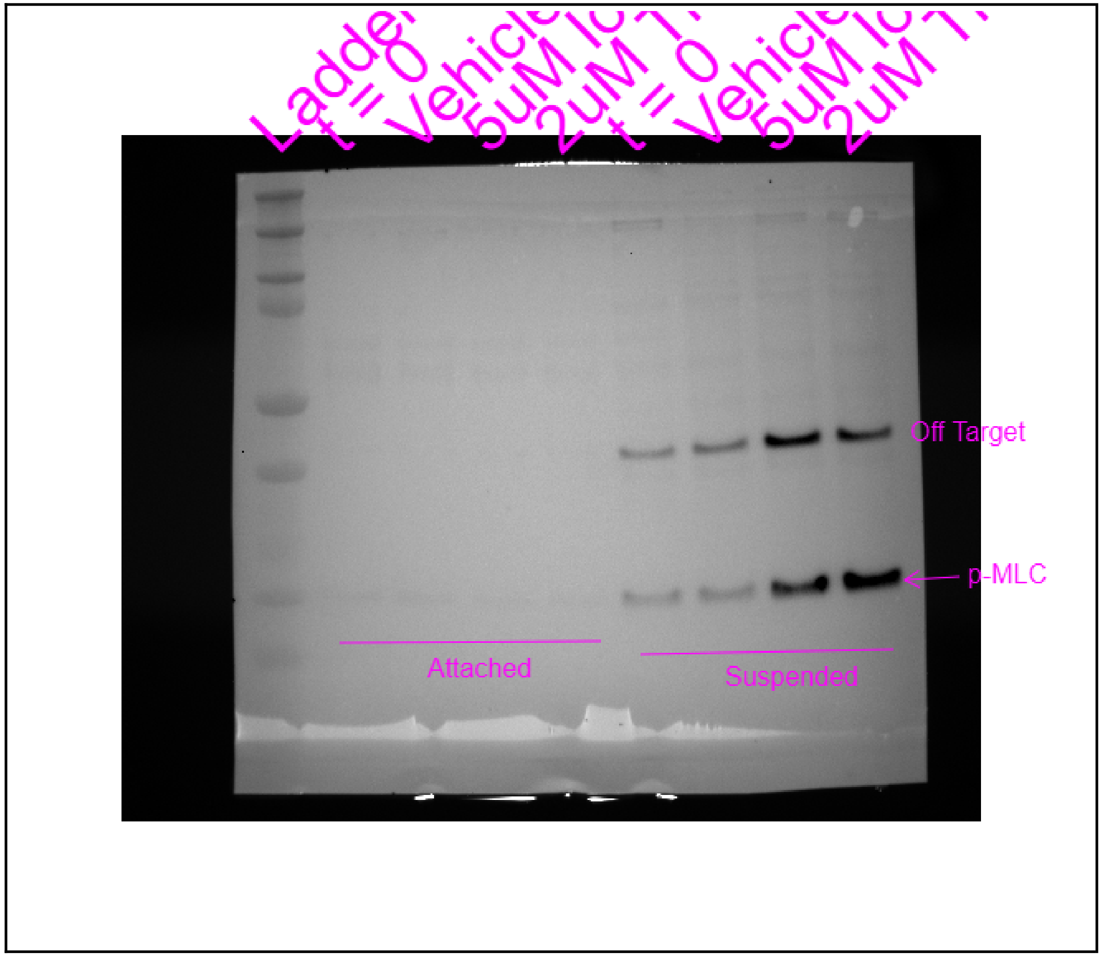

pMLC_CHEMI_10062021_131628

Date:	6 October 2021 01:16:28PM
Mode:	Chemi Blots
Notes:	
Model:	FL1500
Instrument name:	2462619090234
Serial No:	2462619090234
Firmware version:	1.6.0
iBA version:	5.0
Image size:	520px X 415px
Image area:	112.7mm X 90.16mm
Optical Zoom:	2x
Digital Zoom:	1.3x
Focus level:	455
Resolution:	5 x 5
Exposure time:	6773 ms
Exposure mode:	Normal

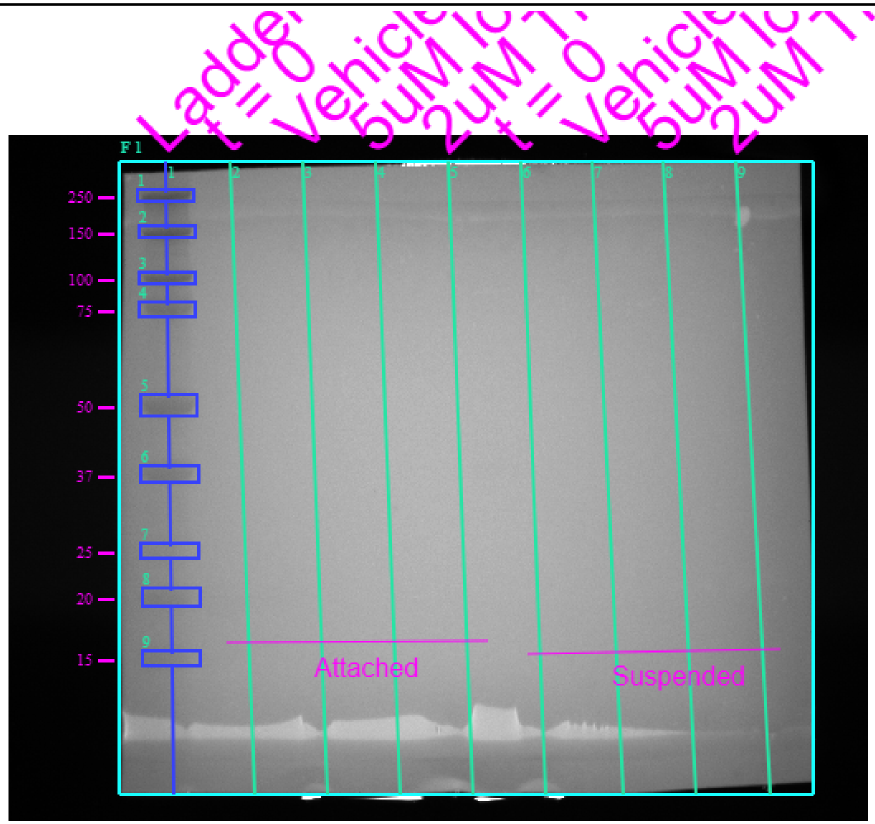

pMLC_CHEMI_10062021_131628

Date:	6 October 2021 01:16:28PM
Mode:	Chemi Blots
Notes:	
Model:	FL1500
Instrument name:	2462619090234
Serial No:	2462619090234
Firmware version:	1.6.0
iBA version:	5.0
Image size:	520px X 415px
Image area:	112.7mm X 90.16mm
Optical Zoom:	2x
Digital Zoom:	1.3x
Focus level:	455
Resolution:	5 x 5
Exposure time:	6773 ms
Exposure mode:	Normal

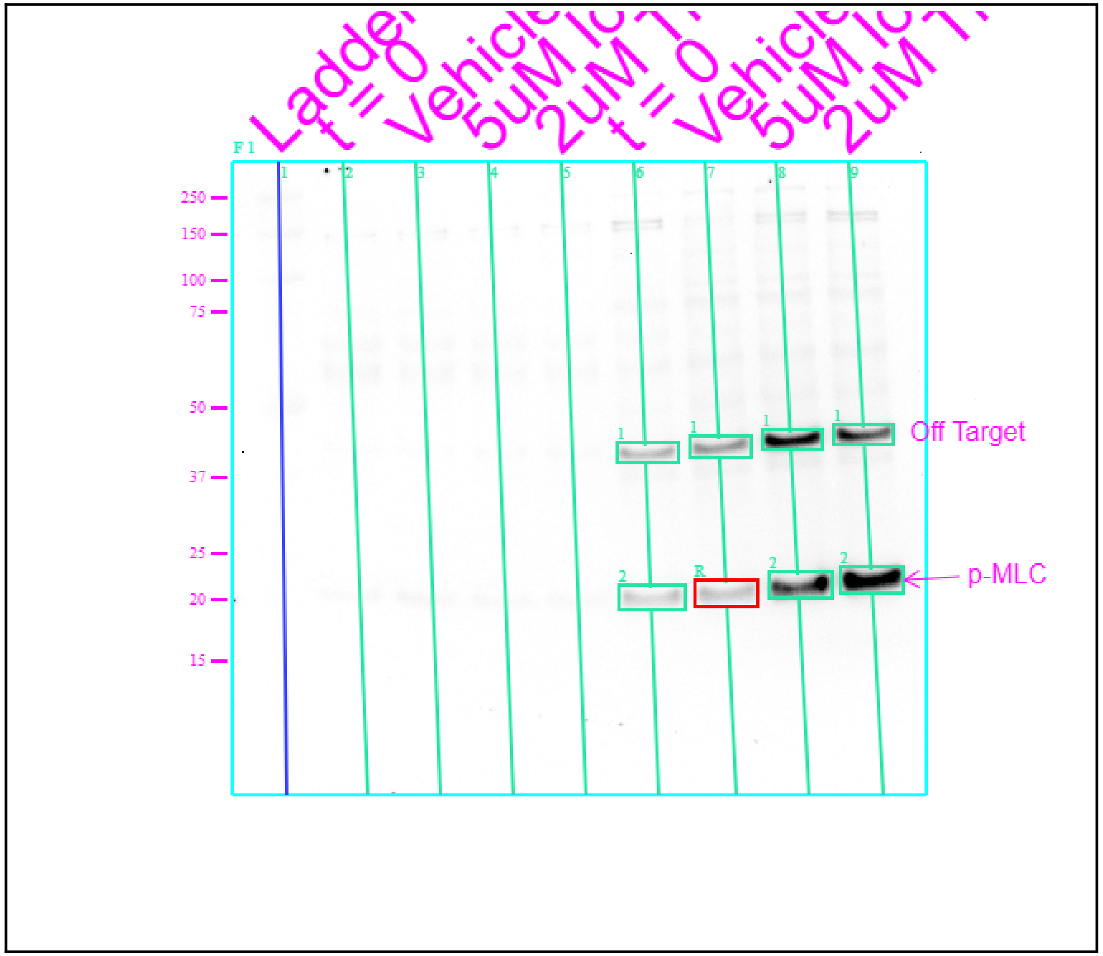

LANE AND BAND ANALYSIS DATA TABLE

pMLC_CHEMI_10062021_131628

Frame: 1
Channel: Membrane
Sensitivity: 100
Molecular Weight Analysis Regression Method : Point to Point

Lane 1 - Ladder

#	Vol. (Int.)	Local Bg. Corr. Vol.	Area	Rf	Density	Local Bg. Corr. Den.	% band purity	% lane purity	Mol. Wt.
1	11,292,904	1,442,791	280	0.052	40,331	5,152.827	14.057	2.636	250
2	10,889,245	1,499,983	280	0.11	38,890	5,357.085	14.614	2.541	150
3	10,273,422	1,357,319	280	0.183	36,690	4,847.569	13.224	2.398	100
4	11,973,450	1,132,731	350	0.232	34,209	3,236.377	11.036	2.795	75
5	16,272,461	1,820,063	490	0.384	33,209	3,714.415	17.733	3.798	50
6	12,737,235	1,181,113	396	0.493	32,164	2,982.609	11.507	2.973	37
7	10,841,214	411,088	360	0.614	30,114	1,141.913	4.005	2.53	25
8	13,643,708	844,511	432	0.687	31,582	1,954.887	8.228	3.184	20
9	11,538,431	574,321	360	0.783	32,051	1,595.339	5.596	2.693	15

Frame: 1
Channel: Chemi
Sensitivity: 100
Molecular Weight Analysis Regression Method : Point to Point

Lane 6 - t = 0

#	Vol. (Int.)	Local Bg. Corr. Vol.	Area	Rf	Density	Local Bg. Corr. Den.	% band purity	% lane purity	Mol. Wt.	Rel. Quant. (w/ LB Corr. Vol.)
1	2,215,157	1,722,696	494	0.46	4,484.123	3,487.24	52.53	22.257	41.024	0.926
2	2,066,741	1,556,784	656	0.687	3,150.52	2,373.147	47.47	20.766	20	0.837

Lane 7 - Vehicle

#	Vol. (Int.)	Local Bg. Corr. Vol.	Area	Rf	Density	Local Bg. Corr. Den.	% band purity	% lane purity	Mol. Wt.	Rel. Quant. (w/ LB Corr. Vol.)
1	3,405,730	2,590,154	532	0.449	6,401.748	4,868.712	58.2	23.477	42.262	1.392
2	2,651,432	1,860,299	663	0.681	3,999.143	2,805.881	41.8	18.278	20.357	1

Lane 8 - 5uM Ionomycin

#	Vol. (Int.)	Local Bg. Corr. Vol.	Area	Rf	Density	Local Bg. Corr. Den.	% band purity	% lane purity	Mol. Wt.	Rel. Quant. (w/ LB Corr. Vol.)
1	8,257,491	7,021,423	481	0.439	17,167	14,597	46.342	29.054	43.5	3.774
2	9,857,746	8,129,872	663	0.668	14,868	12,262	53.658	34.684	21.25	4.37

Lane 9 - 2uM Thapsigargin

#	Vol. (Int.)	Local Bg. Corr. Vol.	Area	Rf	Density	Local Bg. Corr. Den.	% band purity	% lane purity	Mol. Wt.	Rel. Quant. (w/ LB Corr. Vol.)
1	6,082,489	5,217,343	481	0.431	12,645	10,846	30.385	20.837	44.429	2.805
2	13,586,820	11,953,605	663	0.661	20,492	18,029	69.615	46.544	21.786	6.426

iBright™ Image Analysis Report

Katarina+ Chang

18 November 2022

CHEMI_10052021_123018

Date:	5 October 2021 12:30:18PM
Mode:	Chemi Blots
Notes:	
Model:	FL1500
Instrument name:	2462619090234
Serial No:	2462619090234
Firmware version:	1.6.0
iBA version:	5.0
Image size:	563px X 450px
Image area:	112.7mm X 90.16mm
Optical Zoom:	2x
Digital Zoom:	1.2x
Focus level:	455
Resolution:	5 x 5
Exposure time:	27558 ms
Exposure mode:	Normal

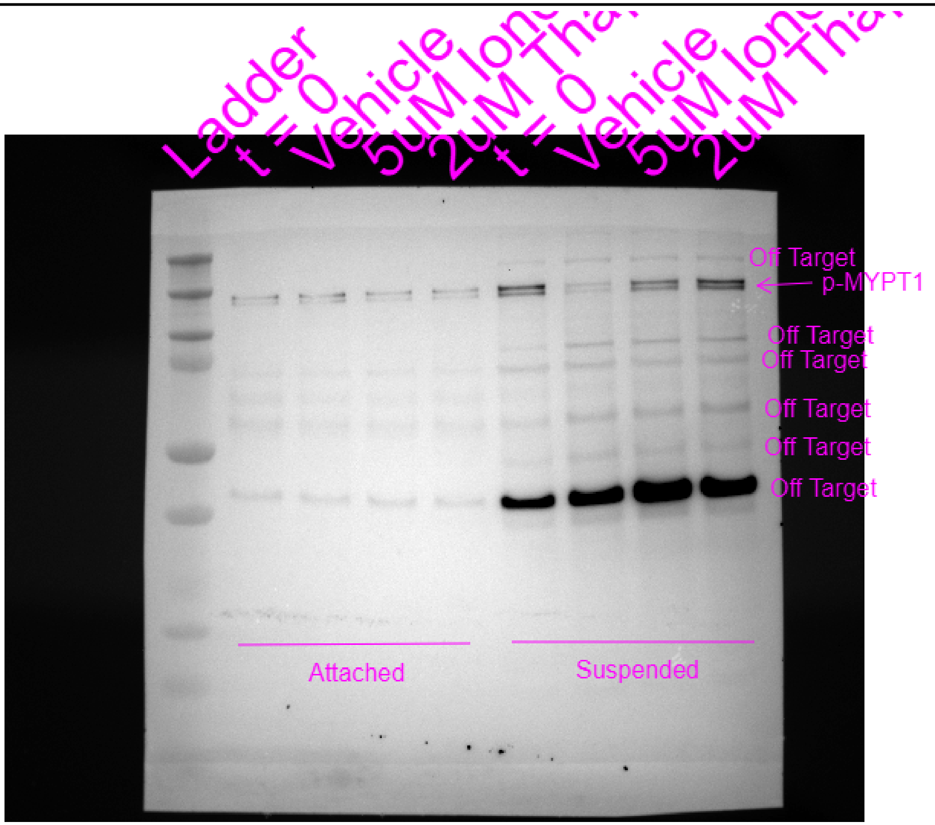

CHEMI_10052021_123018

Date:	5 October 2021 12:30:18PM
Mode:	Chemi Blots
Notes:	
Model:	FL1500
Instrument name:	2462619090234
Serial No:	2462619090234
Firmware version:	1.6.0
iBA version:	5.0
Image size:	563px X 450px
Image area:	112.7mm X 90.16mm
Optical Zoom:	2x
Digital Zoom:	1.2x
Focus level:	455
Resolution:	5 x 5
Exposure time:	27558 ms
Exposure mode:	Normal

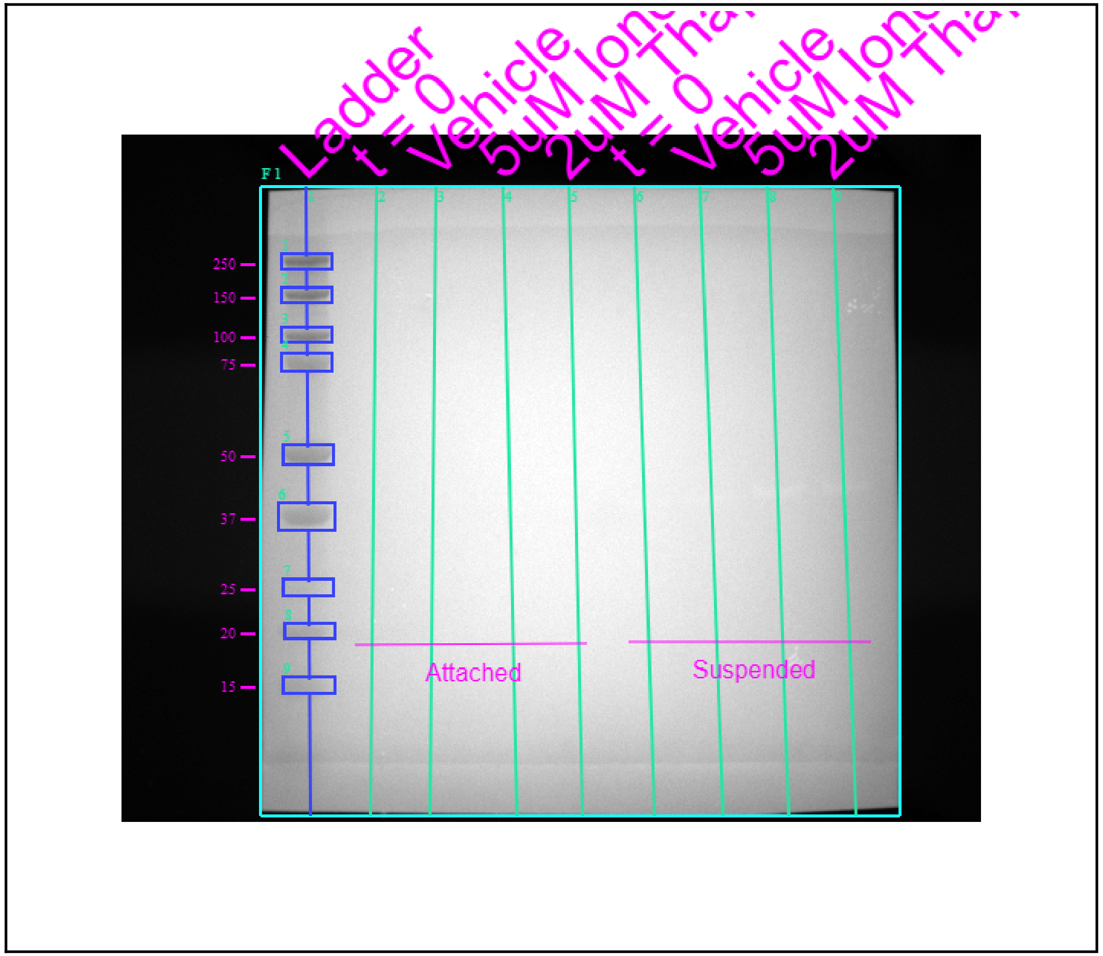

CHEMI_10052021_123018

Date:	5 October 2021 12:30:18PM
Mode:	Chemi Blots
Notes:	
Model:	FL1500
Instrument name:	2462619090234
Serial No:	2462619090234
Firmware version:	1.6.0
iBA version:	5.0
Image size:	563px X 450px
Image area:	112.7mm X 90.16mm
Optical Zoom:	2x
Digital Zoom:	1.2x
Focus level:	455
Resolution:	5 x 5
Exposure time:	27558 ms
Exposure mode:	Normal

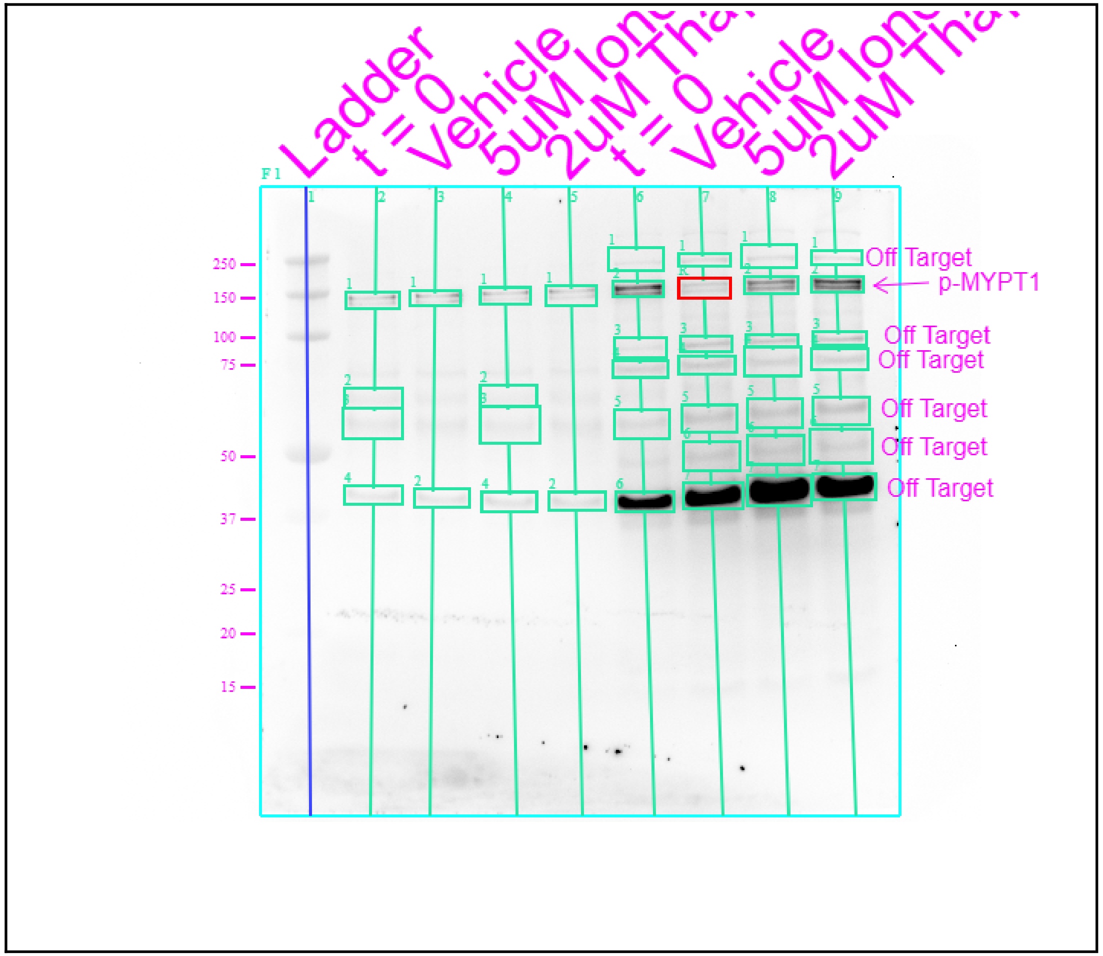

LANE AND BAND ANALYSIS DATA TABLE

CHEMI_10052021_123018

Frame: 1
Channel: Membrane
Sensitivity: 100
Molecular Weight Analysis Regression Method : Point to Point

Lane 1 - Ladder

#	Vol. (Int.)	Local Bg. Corr. Vol.	Area	Rf	Density	Local Bg. Corr. Den.	% band purity	% lane purity	Mol. Wt.
1	14,144,983	2,057,773	374	0.119	37,820	5,502.067	14.848	3.195	250
2	13,579,256	1,910,891	374	0.172	36,308	5,109.336	13.788	3.067	150
3	13,148,325	1,823,919	374	0.235	35,155	4,876.79	13.16	2.97	100
4	14,851,189	1,676,479	442	0.279	33,599	3,792.94	12.097	3.354	75
5	15,875,667	2,177,040	476	0.425	33,352	4,573.614	15.708	3.586	50
6	22,653,942	2,145,061	722	0.524	31,376	2,971	15.478	5.116	37
7	12,270,284	489,568	408	0.636	30,074	1,199.923	3.532	2.771	25
8	11,973,090	872,323	374	0.706	32,013	2,332.415	6.294	2.704	20
9	13,621,063	706,056	420	0.791	32,431	1,681.086	5.095	3.076	15

Frame: 1
Channel: Chemi
Sensitivity: 100
Molecular Weight Analysis Regression Method : Point to Point

Lane 2 - t = 0

#	Vol. (Int.)	Local Bg. Corr. Vol.	Area	Rf	Density	Local Bg. Corr. Den.	% band purity	% lane purity	Mol. Wt.	Rel. Quant. (w/ LB Corr. Vol.)
1	3,039,995	1,536,669	432	0.18	7,037.025	3,557.106	36.845	7.288	144.231	0.985
2	2,468,113	652,345	546	0.335	4,520.353	1,194.771	15.642	5.917	65.417	0.418
3	3,656,900	974,233	840	0.376	4,353.452	1,159.801	23.36	8.767	58.333	0.624
4	1,886,634	1,007,350	507	0.49	3,721.172	1,986.884	24.154	4.523	41.439	0.646

Lane 3 - Vehicle

#	Vol. (Int.)	Local Bg. Corr. Vol.	Area	Rf	Density	Local Bg. Corr. Den.	% band purity	% lane purity	Mol. Wt.	Rel. Quant. (w/ LB Corr. Vol.)
1	3,071,417	1,621,550	374	0.177	8,212.345	4,335.696	62.368	6.764	146.154	1.039

#	Vol. (Int.)	Local Bg. Corr. Vol.	Area	Rf	Density	Local Bg. Corr. Den.	% band purity	% lane purity	Mol. Wt.	Rel. Quant. (w/ LB Corr. Vol.)
2	1,912,351	978,434	481	0.495	3,975.782	2,034.168	37.632	4.211	40.805	0.627

Lane 4 - 5uM Ionomycin

#	Vol. (Int.)	Local Bg. Corr. Vol.	Area	Rf	Density	Local Bg. Corr. Den.	% band purity	% lane purity	Mol. Wt.	Rel. Quant. (w/ LB Corr. Vol.)
1	2,864,059	1,313,499	408	0.172	7,019.752	3,219.362	35.869	6.213	150	0.842
2	2,553,605	451,631	570	0.333	4,480.009	792.336	12.333	5.54	65.833	0.289
3	4,313,842	749,660	1,000	0.379	4,313.842	749.66	20.472	9.358	57.917	0.48
4	2,246,067	1,147,178	518	0.5	4,336.037	2,214.63	31.327	4.872	40.171	0.735

Lane 5 - 2uM Thapsigargin

#	Vol. (Int.)	Local Bg. Corr. Vol.	Area	Rf	Density	Local Bg. Corr. Den.	% band purity	% lane purity	Mol. Wt.	Rel. Quant. (w/ LB Corr. Vol.)
1	3,501,689	1,385,853	476	0.172	7,356.489	2,911.456	59.009	6.687	150	0.888
2	2,711,233	962,674	494	0.5	5,488.326	1,948.735	40.991	5.178	40.171	0.617

Lane 6 - t = 0

#	Vol. (Int.)	Local Bg. Corr. Vol.	Area	Rf	Density	Local Bg. Corr. Den.	% band purity	% lane purity	Mol. Wt.	Rel. Quant. (w/ LB Corr. Vol.)
1	3,120,033	323,211	592	0.114	5,270.326	545.965	1.26	3.319	NA	0.207
2	8,269,014	5,839,986	374	0.163	22,109	15,614	22.771	8.797	168.182	3.743
3	3,106,365	318,826	490	0.255	6,339.52	650.666	1.243	3.305	88.889	0.204
4	3,614,386	1,176,532	444	0.289	8,140.509	2,649.847	4.587	3.845	73.333	0.754
5	5,573,761	1,259,680	740	0.376	7,532.109	1,702.271	4.912	5.93	58.333	0.807
6	22,121,140	16,728,374	546	0.5	40,514	30,638	65.226	23.534	40.171	10.722

Lane 7 - Vehicle

#	Vol. (Int.)	Local Bg. Corr. Vol.	Area	Rf	Density	Local Bg. Corr. Den.	% band purity	% lane purity	Mol. Wt.	Rel. Quant. (w/ LB Corr. Vol.)
1	2,054,897	461,871	315	0.117	6,523.483	1,466.26	1.696	1.827	NA	0.296
2	4,371,571	1,560,185	490	0.16	8,921.573	3,184.052	5.728	3.886	172.727	1
3	3,617,136	981,340	385	0.25	9,395.158	2,548.936	3.603	3.215	91.667	0.629
4	4,604,829	1,308,565	494	0.284	9,321.516	2,648.919	4.804	4.093	74.167	0.839
5	7,600,195	1,958,221	703	0.369	10,811	2,785.522	7.189	6.756	59.583	1.255
6	8,882,268	1,270,515	760	0.427	11,687	1,671.731	4.664	7.896	49.683	0.814

#	Vol. (Int.)	Local Bg. Corr. Vol.	Area	Rf	Density	Local Bg. Corr. Den.	% band purity	% lane purity	Mol. Wt.	Rel. Quant. (w/ LB Corr. Vol.)
7	31,495,682	19,698,480	720	0.49	43,744	27,359	72.317	27.998	41.439	12.626

Lane 8 - 5uM Ionomycin

#	Vol. (Int.)	Local Bg. Corr. Vol.	Area	Rf	Density	Local Bg. Corr. Den.	% band purity	% lane purity	Mol. Wt.	Rel. Quant. (w/ LB Corr. Vol.)
1	3,053,637	485,679	592	0.109	5,158.171	820.405	1.332	2.504	NA	0.311
2	7,102,498	4,715,772	432	0.155	16,440	10,916	12.929	5.823	181.818	3.023
3	2,925,250	1,004,799	315	0.245	9,286.508	3,189.84	2.755	2.398	94.444	0.644
4	5,969,097	831,458	760	0.277	7,854.075	1,094.025	2.28	4.894	76.389	0.533
5	8,182,743	2,049,037	740	0.359	11,057	2,768.97	5.618	6.709	61.25	1.313
6	9,776,198	1,058,551	760	0.417	12,863	1,392.831	2.902	8.015	51.25	0.678
7	44,576,162	26,329,170	903	0.483	49,364	29,157	72.185	36.547	42.39	16.876

Lane 9 - 2uM Thapsigargin

#	Vol. (Int.)	Local Bg. Corr. Vol.	Area	Rf	Density	Local Bg. Corr. Den.	% band purity	% lane purity	Mol. Wt.	Rel. Quant. (w/ LB Corr. Vol.)
1	1,397,343	458,952	340	0.112	4,109.832	1,349.86	1.204	1.33	NA	0.294
2	9,290,776	7,574,966	385	0.155	24,131	19,675	19.875	8.842	181.818	4.855
3	2,905,414	1,181,334	360	0.24	8,070.594	3,281.483	3.1	2.765	97.222	0.757
4	4,029,280	1,177,796	570	0.274	7,068.912	2,066.31	3.09	3.835	77.778	0.755
5	7,208,297	2,580,824	722	0.357	9,983.791	3,574.549	6.772	6.86	61.667	1.654
6	9,291,356	1,041,542	966	0.413	9,618.381	1,078.201	2.733	8.843	52.083	0.668
7	35,525,003	24,097,028	756	0.476	46,990	31,874	63.226	33.811	43.341	15.445

iBright™ Image Analysis Report

Katarina+ Chang

18 November 2022

GAPDH_CHEMI_10082021_124627

Date:	8 October 2021 12:46:27PM
Mode:	Chemi Blots
Notes:	
Model:	FL1500
Instrument name:	2462619090234
Serial No:	2462619090234
Firmware version:	1.6.0
iBA version:	5.0
Image size:	563px X 450px
Image area:	112.7mm X 90.16mm
Optical Zoom:	2x
Digital Zoom:	1.2x
Focus level:	455
Resolution:	5 x 5
Exposure time:	1310 ms
Exposure mode:	Normal

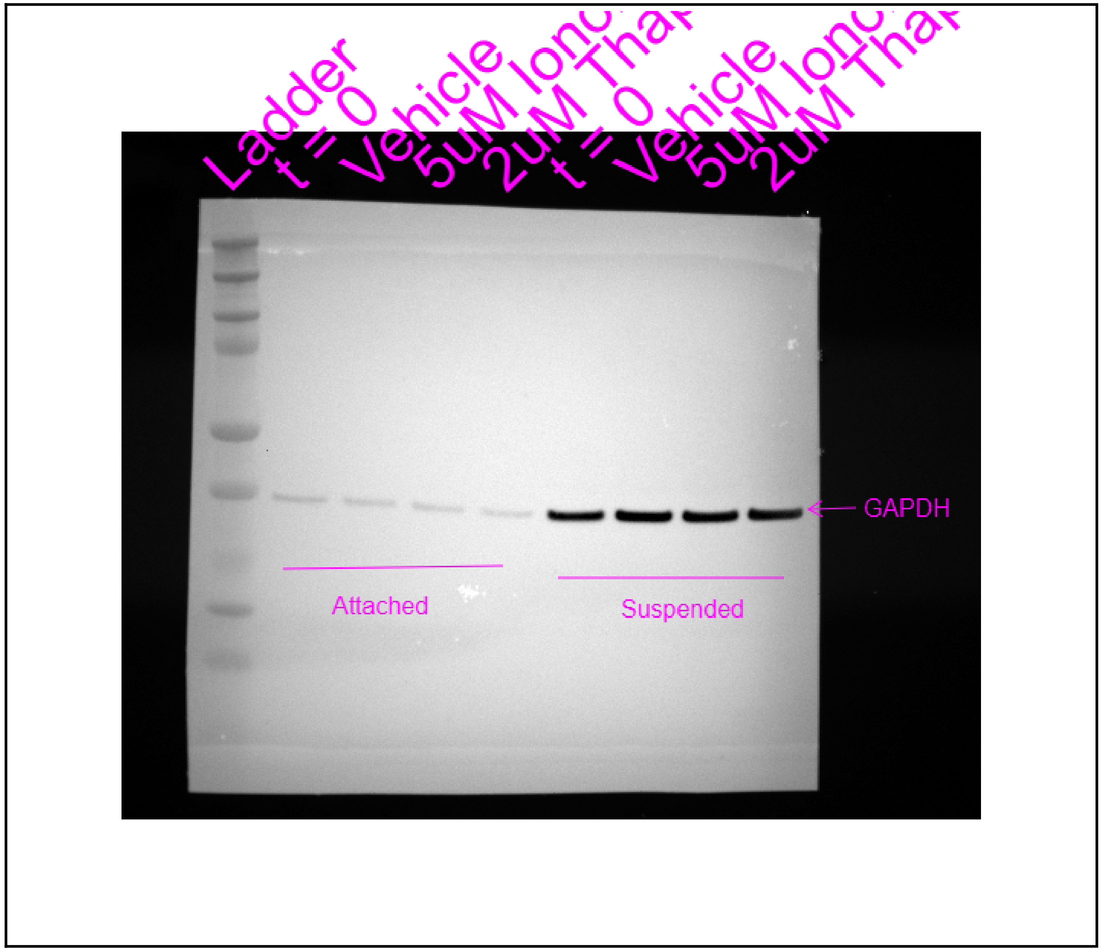

GAPDH_CHEMI_10082021_124627

Date:	8 October 2021 12:46:27PM
Mode:	Chemi Blots
Notes:	
Model:	FL1500
Instrument name:	2462619090234
Serial No:	2462619090234
Firmware version:	1.6.0
iBA version:	5.0
Image size:	563px X 450px
Image area:	112.7mm X 90.16mm
Optical Zoom:	2x
Digital Zoom:	1.2x
Focus level:	455
Resolution:	5 x 5
Exposure time:	1310 ms
Exposure mode:	Normal

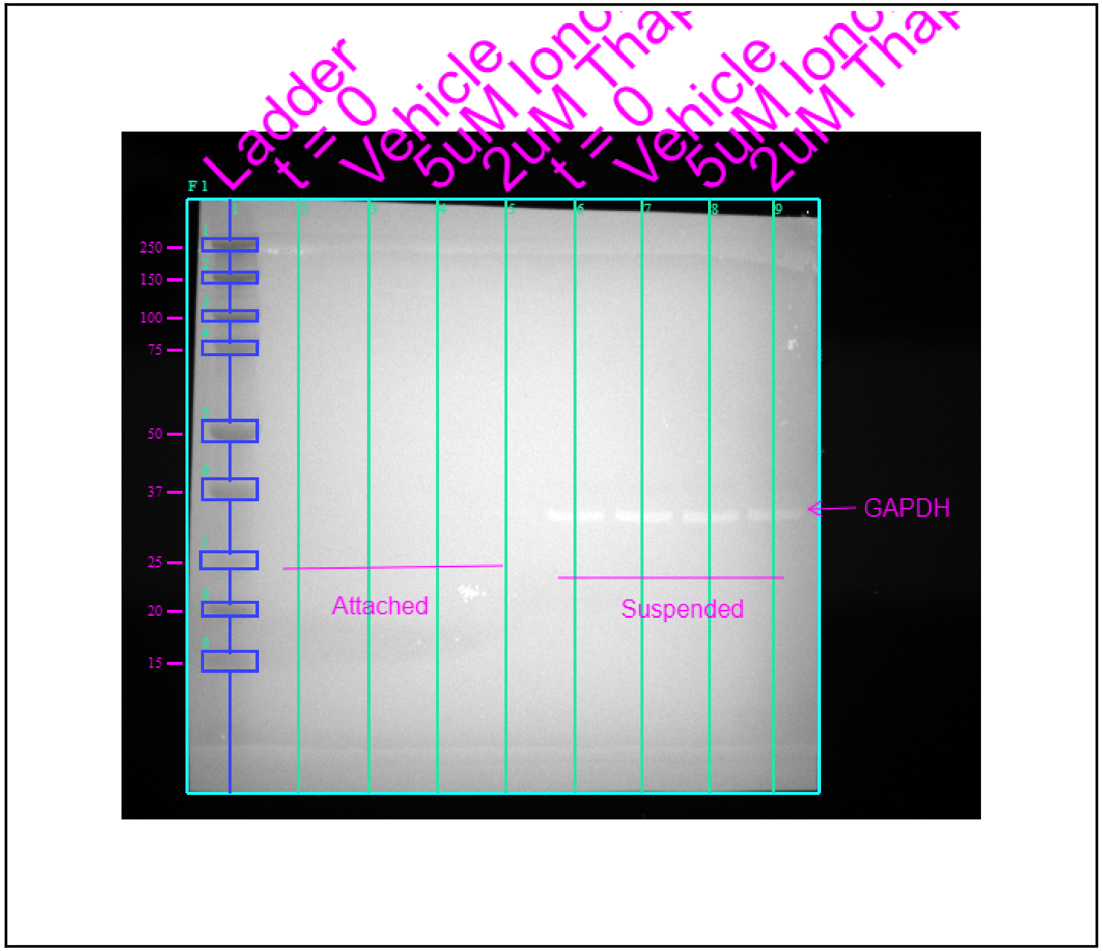

GAPDH_CHEMI_10082021_124627

Date:	8 October 2021 12:46:27PM
Mode:	Chemi Blots
Notes:	
Model:	FL1500
Instrument name:	2462619090234
Serial No:	2462619090234
Firmware version:	1.6.0
iBA version:	5.0
Image size:	563px X 450px
Image area:	112.7mm X 90.16mm
Optical Zoom:	2x
Digital Zoom:	1.2x
Focus level:	455
Resolution:	5 x 5
Exposure time:	1310 ms
Exposure mode:	Normal

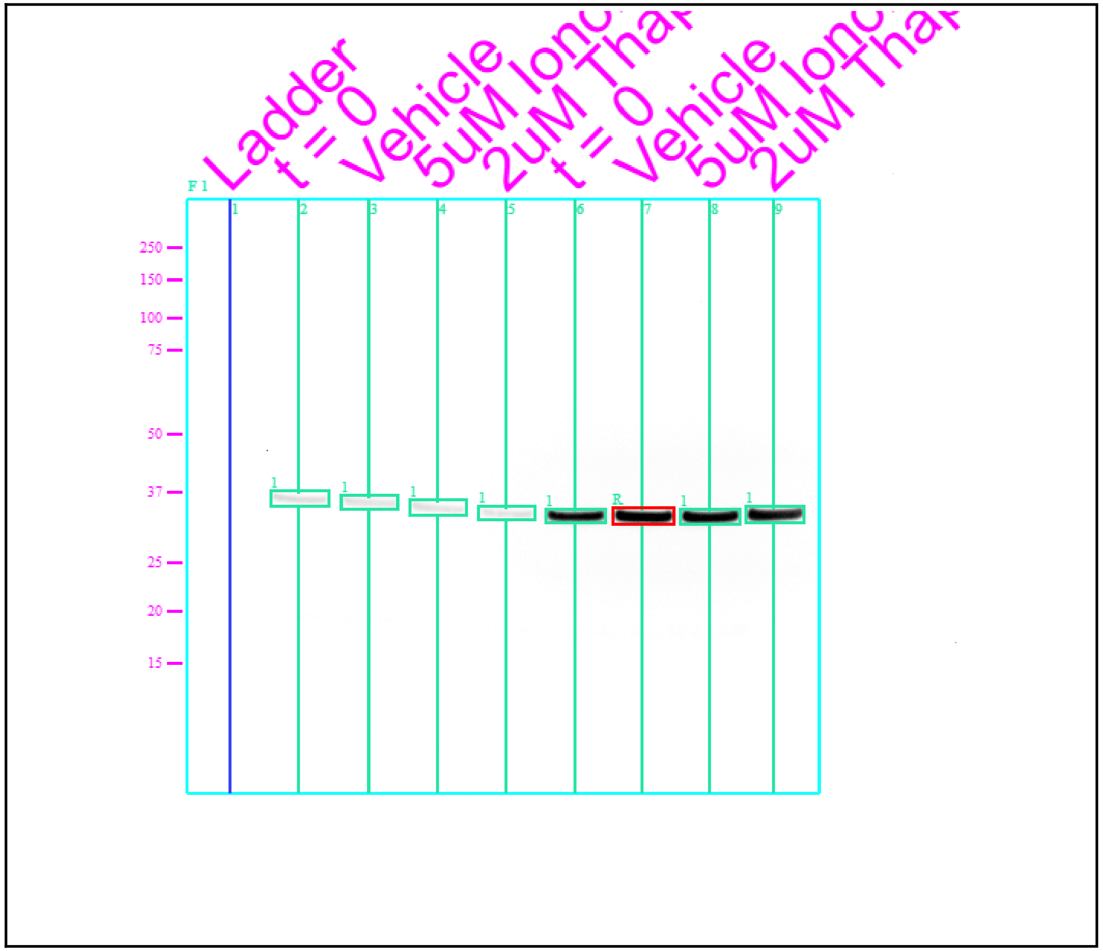

LANE AND BAND ANALYSIS DATA TABLE

GAPDH_CHEMI_10082021_124627

Frame: 1
Channel: Membrane
Sensitivity: 100
Molecular Weight Analysis Regression Method : Point to Point

Lane 1 - Ladder

#	Vol. (Int.)	Local Bg. Corr. Vol.	Area	Rf	Density	Local Bg. Corr. Den.	% band purity	% lane purity	Mol. Wt.
1	13,645,729	906,543	333	0.077	40,978	2,722.354	7.843	NA	250
2	11,962,468	1,121,152	296	0.131	40,413	3,787.676	9.7	NA	150
3	11,531,052	1,235,197	296	0.195	38,956	4,172.964	10.686	NA	100
4	13,488,949	1,096,912	370	0.249	36,456	2,964.628	9.49	NA	75
5	19,802,315	2,384,686	555	0.391	35,679	4,296.733	20.631	NA	50
6	19,007,035	1,815,889	555	0.488	34,246	3,271.873	15.71	NA	37
7	14,856,022	662,111	456	0.607	32,578	1,451.998	5.728	NA	25
8	13,324,672	1,244,782	370	0.689	36,012	3,364.276	10.769	NA	20
9	18,338,531	1,091,555	518	0.776	35,402	2,107.25	9.443	NA	15

Frame: 1
Channel: Chemi
Sensitivity: 100
Molecular Weight Analysis Regression Method : Point to Point

Lane 2 - t = 0

#	Vol. (Int.)	Local Bg. Corr. Vol.	Area	Rf	Density	Local Bg. Corr. Den.	% band purity	% lane purity	Mol. Wt.	Rel. Quant. (w/ LB Corr. Vol.)
1	1,020,364	969,661	429	0.504	2,378.471	2,260.283	100	NA	35.435	0.085

Lane 3 - Vehicle

#	Vol. (Int.)	Local Bg. Corr. Vol.	Area	Rf	Density	Local Bg. Corr. Den.	% band purity	% lane purity	Mol. Wt.	Rel. Quant. (w/ LB Corr. Vol.)
1	861,842	802,771	380	0.509	2,268.005	2,112.557	100	NA	34.913	0.071

Lane 4 - 5uM Ionomycin

#	Vol. (Int.)	Local Bg. Corr. Vol.	Area	Rf	Density	Local Bg. Corr. Den.	% band purity	% lane purity	Mol. Wt.	Rel. Quant. (w/ LB Corr. Vol.)

#	Vol. (Int.)	Local Bg. Corr. Vol.	Area	Rf	Density	Local Bg. Corr. Den.	% band purity	% lane purity	Mol. Wt.	Rel. Quant. (w/ LB Corr. Vol.)
1	872,223	805,806	418	0.519	2,086.658	1,927.767	100	NA	33.87	0.071

Lane 5 - 2uM Thapsigargin

#	Vol. (Int.)	Local Bg. Corr. Vol.	Area	Rf	Density	Local Bg. Corr. Den.	% band purity	% lane purity	Mol. Wt.	Rel. Quant. (w/ LB Corr. Vol.)
1	834,547	714,248	380	0.527	2,196.176	1,879.601	100	NA	33.087	0.063

Lane 6 - t = 0

#	Vol. (Int.)	Local Bg. Corr. Vol.	Area	Rf	Density	Local Bg. Corr. Den.	% band purity	% lane purity	Mol. Wt.	Rel. Quant. (w/ LB Corr. Vol.)
1	9,266,575	8,791,806	400	0.532	23,166	21,979	100	NA	32.565	0.772

Lane 7 - Vehicle

#	Vol. (Int.)	Local Bg. Corr. Vol.	Area	Rf	Density	Local Bg. Corr. Den.	% band purity	% lane purity	Mol. Wt.	Rel. Quant. (w/ LB Corr. Vol.)
1	12,093,710	11,382,304	492	0.532	24,580	23,134	100	NA	32.565	1

Lane 8 - 5uM Ionomycin

#	Vol. (Int.)	Local Bg. Corr. Vol.	Area	Rf	Density	Local Bg. Corr. Den.	% band purity	% lane purity	Mol. Wt.	Rel. Quant. (w/ LB Corr. Vol.)
1	10,964,871	10,281,462	440	0.535	24,920	23,366	100	NA	32.304	0.903

Lane 9 - 2uM Thapsigargin

#	Vol. (Int.)	Local Bg. Corr. Vol.	Area	Rf	Density	Local Bg. Corr. Den.	% band purity	% lane purity	Mol. Wt.	Rel. Quant. (w/ LB Corr. Vol.)
1	9,770,324	9,453,915	468	0.53	20,876	20,200	100	NA	32.826	0.831

iBright™ Image Analysis Report

Katarina+ Chang

18 November 2022

total cofilin_CHEMI_10062021_125536

Date:	6 October 2021 12:55:36PM
Mode:	Chemi Blots
Notes:	
Model:	FL1500
Instrument name:	2462619090234
Serial No:	2462619090234
Firmware version:	1.6.0
iBA version:	5.0
Image size:	615px X 491px
Image area:	112.7mm X 90.16mm
Optical Zoom:	2x
Digital Zoom:	1.1x
Focus level:	455
Resolution:	5 x 5
Exposure time:	2444 ms
Exposure mode:	Normal

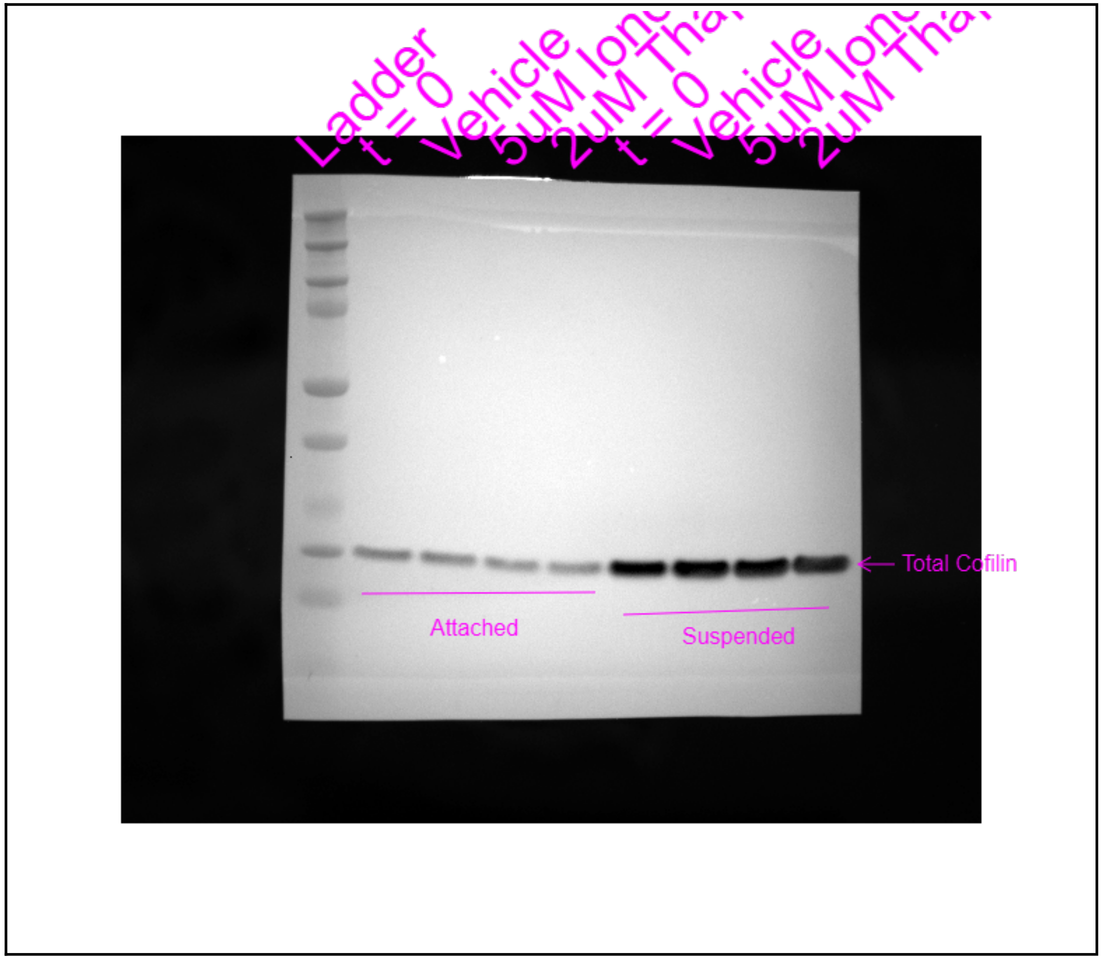

total cofilin_CHEMI_10062021_125536

Date:	6 October 2021 12:55:36PM
Mode:	Chemi Blots
Notes:	
Model:	FL1500
Instrument name:	2462619090234
Serial No:	2462619090234
Firmware version:	1.6.0
iBA version:	5.0
Image size:	615px X 491px
Image area:	112.7mm X 90.16mm
Optical Zoom:	2x
Digital Zoom:	1.1x
Focus level:	455
Resolution:	5 x 5
Exposure time:	2444 ms
Exposure mode:	Normal

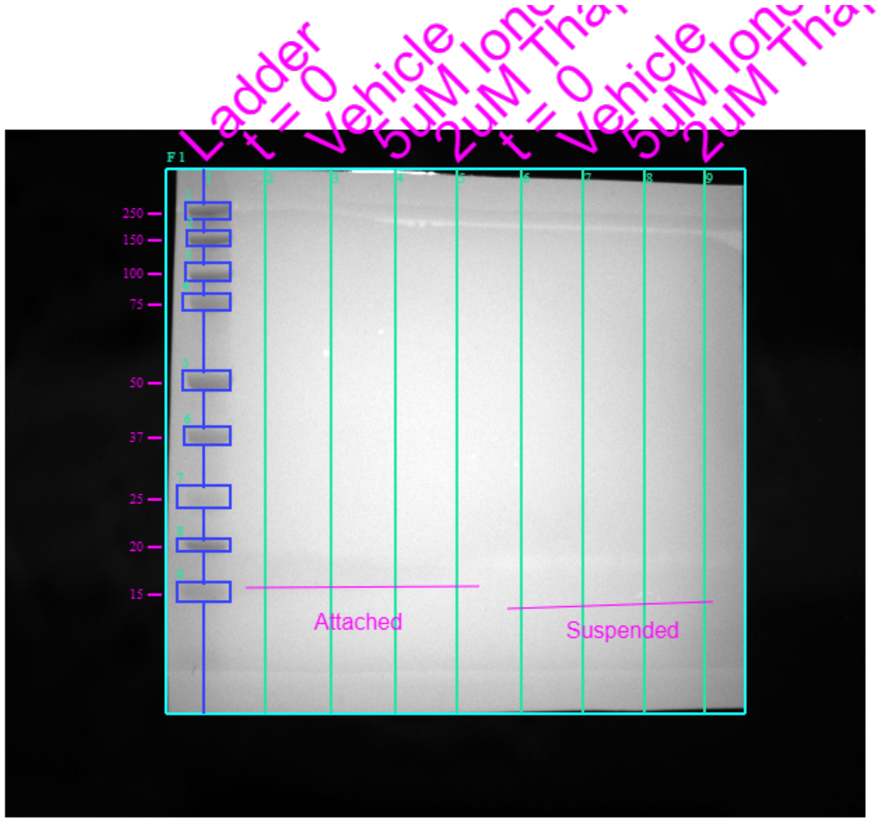

total cofilin_CHEMI_10062021_125536

Date:	6 October 2021 12:55:36PM
Mode:	Chemi Blots
Notes:	
Model:	FL1500
Instrument name:	2462619090234
Serial No:	2462619090234
Firmware version:	1.6.0
iBA version:	5.0
Image size:	615px X 491px
Image area:	112.7mm X 90.16mm
Optical Zoom:	2x
Digital Zoom:	1.1x
Focus level:	455
Resolution:	5 x 5
Exposure time:	2444 ms
Exposure mode:	Normal

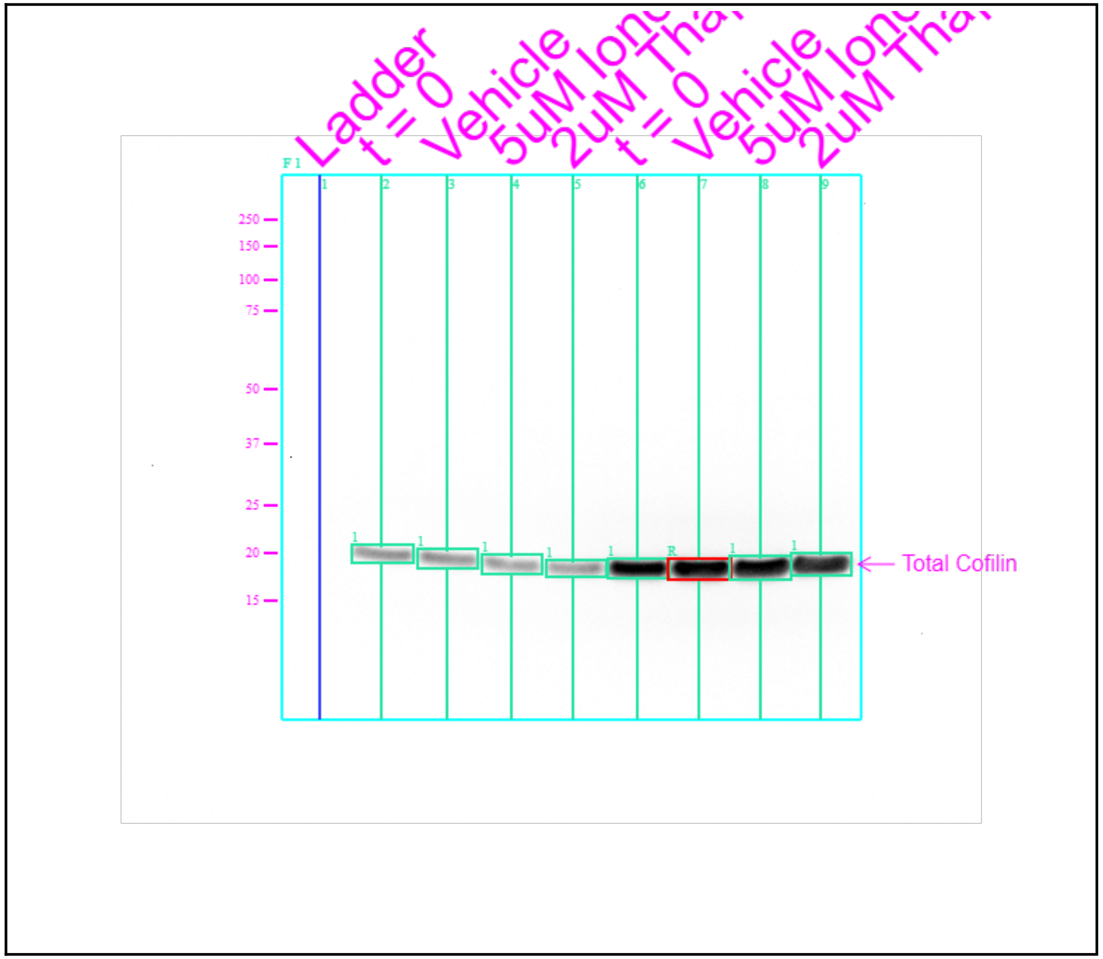

LANE AND BAND ANALYSIS DATA TABLE

total cofilin_CHEMI_10062021_125536

Frame: 1
Channel: Membrane
Sensitivity: 100
Molecular Weight Analysis Regression Method : Point to Point

Lane 1 - Ladder

#	Vol. (Int.)	Local Bg. Corr. Vol.	Area	Rf	Density	Local Bg. Corr. Den.	% band purity	% lane purity	Mol. Wt.
1	17,210,338	2,143,628	429	0.077	40,117	4,996.803	12.121	3.417	250
2	15,093,218	1,850,094	384	0.126	39,305	4,817.956	10.462	2.997	150
3	17,038,398	1,867,926	462	0.188	36,879	4,043.13	10.562	3.383	100
4	16,287,165	1,807,938	455	0.244	35,795	3,973.491	10.223	3.234	75
5	18,664,674	3,029,698	525	0.388	35,551	5,770.854	17.132	3.706	50
6	16,082,982	2,272,512	476	0.488	33,787	4,774.185	12.85	3.194	37
7	20,370,169	1,139,311	663	0.602	30,724	1,718.418	6.442	4.045	25
8	13,987,987	2,010,186	390	0.689	35,866	5,154.324	11.367	2.778	20
9	19,755,438	1,563,478	585	0.776	33,769	2,672.612	8.841	3.923	15

Frame: 1
Channel: Chemi
Sensitivity: 100
Molecular Weight Analysis Regression Method : Point to Point

Lane 2 - t = 0

#	Vol. (Int.)	Local Bg. Corr. Vol.	Area	Rf	Density	Local Bg. Corr. Den.	% band purity	% lane purity	Mol. Wt.	Rel. Quant. (w/ LB Corr. Vol.)
1	4,103,646	3,691,287	630	0.694	6,513.724	5,859.186	100	73.261	19.706	0.332

Lane 3 - Vehicle

#	Vol. (Int.)	Local Bg. Corr. Vol.	Area	Rf	Density	Local Bg. Corr. Den.	% band purity	% lane purity	Mol. Wt.	Rel. Quant. (w/ LB Corr. Vol.)
1	4,157,145	3,571,291	660	0.704	6,298.705	5,411.048	100	69.113	19.118	0.322

Lane 4 - 5uM Ionomycin

#	Vol. (Int.)	Local Bg. Corr. Vol.	Area	Rf	Density	Local Bg. Corr. Den.	% band purity	% lane purity	Mol. Wt.	Rel. Quant. (w/ LB Corr. Vol.)

#	Vol. (Int.)	Local Bg. Corr. Vol.	Area	Rf	Density	Local Bg. Corr. Den.	% band purity	% lane purity	Mol. Wt.	Rel. Quant. (w/ LB Corr. Vol.)
1	3,572,520	2,919,608	660	0.715	5,412.909	4,423.65	100	62.626	18.529	0.263

Lane 5 - 2uM Thapsigargin

#	Vol. (Int.)	Local Bg. Corr. Vol.	Area	Rf	Density	Local Bg. Corr. Den.	% band purity	% lane purity	Mol. Wt.	Rel. Quant. (w/ LB Corr. Vol.)
1	4,043,324	2,559,274	572	0.722	7,068.748	4,474.255	100	59.087	18.088	0.231

Lane 6 - t = 0

#	Vol. (Int.)	Local Bg. Corr. Vol.	Area	Rf	Density	Local Bg. Corr. Den.	% band purity	% lane purity	Mol. Wt.	Rel. Quant. (w/ LB Corr. Vol.)
1	13,329,118	10,667,582	675	0.722	19,746	15,803	100	78.729	18.088	0.961

Lane 7 - Vehicle

#	Vol. (Int.)	Local Bg. Corr. Vol.	Area	Rf	Density	Local Bg. Corr. Den.	% band purity	% lane purity	Mol. Wt.	Rel. Quant. (w/ LB Corr. Vol.)
1	14,993,520	11,102,687	736	0.722	20,371	15,085	100	79.104	18.088	1

Lane 8 - 5uM Ionomycin

#	Vol. (Int.)	Local Bg. Corr. Vol.	Area	Rf	Density	Local Bg. Corr. Den.	% band purity	% lane purity	Mol. Wt.	Rel. Quant. (w/ LB Corr. Vol.)
1	14,621,109	10,608,131	810	0.72	18,050	13,096	100	80.418	18.235	0.955

Lane 9 - 2uM Thapsigargin

#	Vol. (Int.)	Local Bg. Corr. Vol.	Area	Rf	Density	Local Bg. Corr. Den.	% band purity	% lane purity	Mol. Wt.	Rel. Quant. (w/ LB Corr. Vol.)
1	12,302,380	10,359,589	748	0.715	16,447	13,849	100	81.396	18.529	0.933

iBright™ Image Analysis Report

Katarina+ Chang

18 November 2022

detyr tubulin CHEMI_10052021_123652

Date:	5 October 2021 12:36:52PM
Mode:	Chemi Blots
Notes:	
Model:	FL1500
Instrument name:	2462619090234
Serial No:	2462619090234
Firmware version:	1.6.0
iBA version:	5.0
Image size:	563px X 450px
Image area:	112.7mm X 90.16mm
Optical Zoom:	2x
Digital Zoom:	1.2x
Focus level:	455
Resolution:	5 x 5
Exposure time:	20489 ms
Exposure mode:	Normal

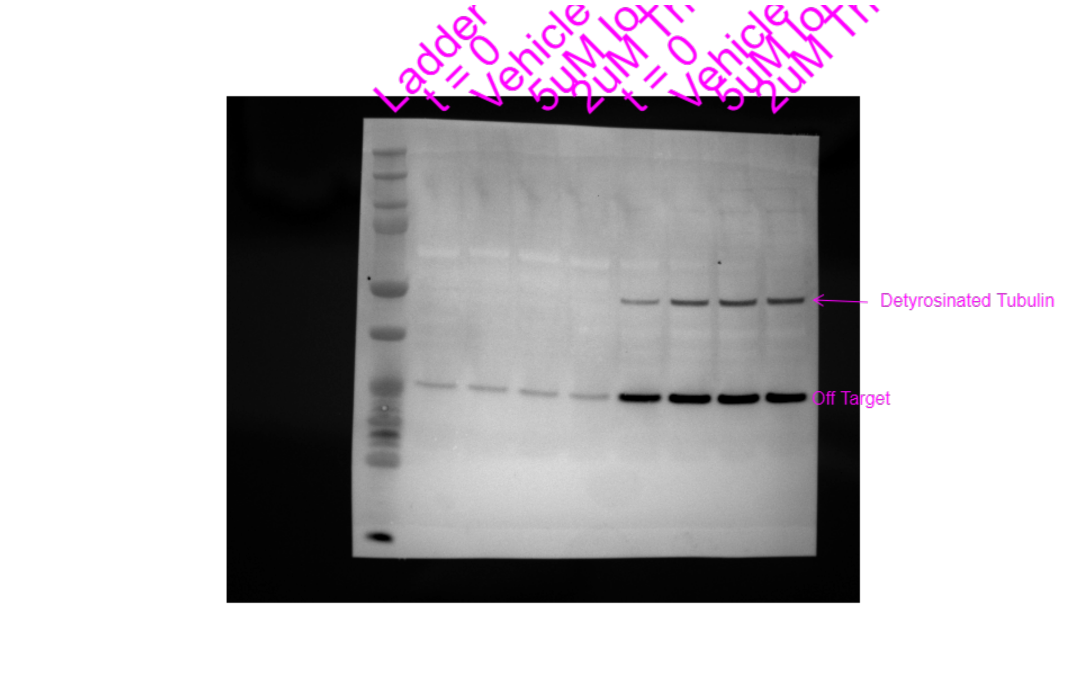

detyr tubulin CHEMI_10052021_123652

Date:	5 October 2021 12:36:52PM
Mode:	Chemi Blots
Notes:	
Model:	FL1500
Instrument name:	2462619090234
Serial No:	2462619090234
Firmware version:	1.6.0
iBA version:	5.0
Image size:	563px X 450px
Image area:	112.7mm X 90.16mm
Optical Zoom:	2x
Digital Zoom:	1.2x
Focus level:	455
Resolution:	5 x 5
Exposure time:	20489 ms
Exposure mode:	Normal

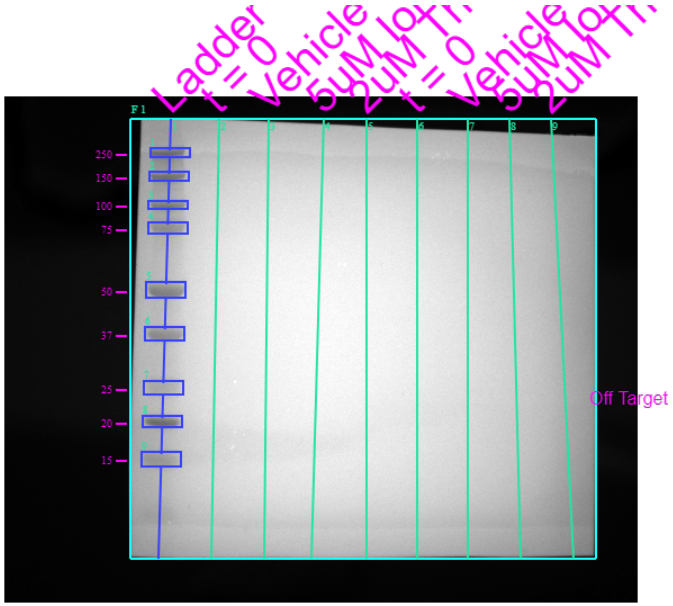

detyr tubulin CHEMI_10052021_123652

Date:	5 October 2021 12:36:52PM
Mode:	Chemi Blots
Notes:	
Model:	FL1500
Instrument name:	2462619090234
Serial No:	2462619090234
Firmware version:	1.6.0
iBA version:	5.0
Image size:	563px X 450px
Image area:	112.7mm X 90.16mm
Optical Zoom:	2x
Digital Zoom:	1.2x
Focus level:	455
Resolution:	5 x 5
Exposure time:	20489 ms
Exposure mode:	Normal

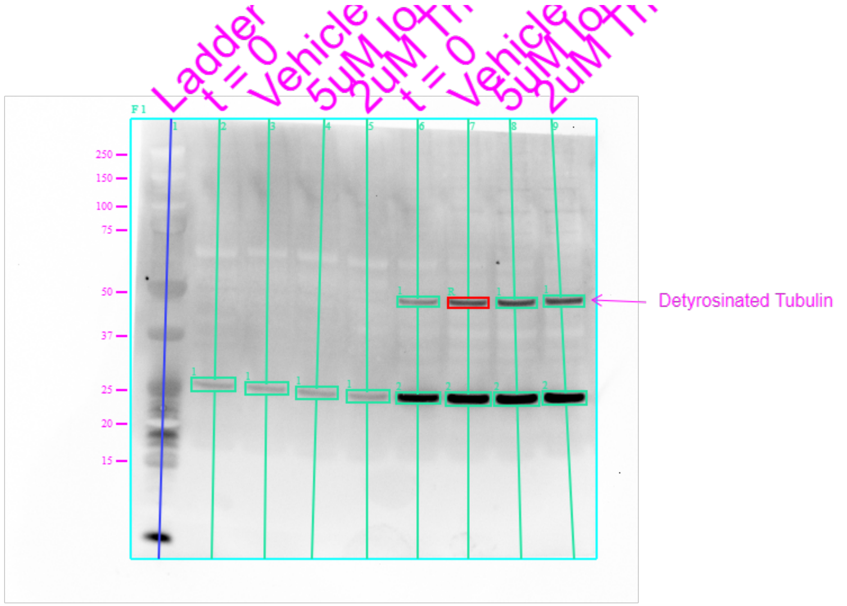

LANE AND BAND ANALYSIS DATA TABLE

detyr tubulin CHEMI_10052021_123652

Frame: 1
Channel: Membrane
Sensitivity: 100
Molecular Weight Analysis Regression Method : Point to Point

Lane 1 - Ladder

#	Vol. (Int.)	Local Bg. Corr. Vol.	Area	Rf	Density	Local Bg. Corr. Den.	% band purity	% lane purity	Mol. Wt.
1	12,720,380	1,939,956	324	0.077	39,260	5,987.519	9.629	2.893	250
2	12,355,404	1,977,039	324	0.13	38,133	6,101.972	9.813	2.81	150
3	10,625,239	1,748,553	288	0.194	36,893	6,071.367	8.679	2.417	100
4	13,527,151	1,815,628	396	0.248	34,159	4,584.922	9.012	3.077	75
5	18,760,303	3,788,776	540	0.389	34,741	7,016.253	18.805	4.267	50
6	15,704,151	2,859,653	468	0.488	33,555	6,110.371	14.194	3.572	37
7	14,188,282	1,229,588	468	0.611	30,316	2,627.326	6.103	3.227	25
8	14,673,237	3,079,305	396	0.688	37,053	7,776.024	15.284	3.337	20
9	16,860,992	1,708,681	504	0.772	33,454	3,390.241	8.481	3.835	15

Frame: 1
Channel: Chemi
Sensitivity: 100
Molecular Weight Analysis Regression Method : Point to Point

Lane 2 - t = 0

#	Vol. (Int.)	Local Bg. Corr. Vol.	Area	Rf	Density	Local Bg. Corr. Den.	% band purity	% lane purity	Mol. Wt.	Rel. Quant. (w/ LB Corr. Vol.)
1	9,531,685	2,064,816	520	0.604	18,330	3,970.801	100	5.819	25.75	0.431

Lane 3 - Vehicle

#	Vol. (Int.)	Local Bg. Corr. Vol.	Area	Rf	Density	Local Bg. Corr. Den.	% band purity	% lane purity	Mol. Wt.	Rel. Quant. (w/ LB Corr. Vol.)
1	8,328,672	2,079,205	468	0.611	17,796	4,442.747	100	4.746	25	0.434

Lane 4 - 5uM Ionomycin

#	Vol. (Int.)	Local Bg. Corr. Vol.	Area	Rf	Density	Local Bg. Corr. Den.	% band purity	% lane purity	Mol. Wt.	Rel. Quant. (w/ LB Corr. Vol.)

#	Vol. (Int.)	Local Bg. Corr. Vol.	Area	Rf	Density	Local Bg. Corr. Den.	% band purity	% lane purity	Mol. Wt.	Rel. Quant. (w/ LB Corr. Vol.)
1	8,603,772	2,199,635	456	0.621	18,867	4,823.763	100	5.026	24.333	0.459

Lane 5 - 2uM Thapsigargin

#	Vol. (Int.)	Local Bg. Corr. Vol.	Area	Rf	Density	Local Bg. Corr. Den.	% band purity	% lane purity	Mol. Wt.	Rel. Quant. (w/ LB Corr. Vol.)
1	9,422,636	2,120,670	468	0.629	20,133	4,531.347	100	5.478	23.833	0.443

Lane 6 - t = 0

#	Vol. (Int.)	Local Bg. Corr. Vol.	Area	Rf	Density	Local Bg. Corr. Den.	% band purity	% lane purity	Mol. Wt.	Rel. Quant. (w/ LB Corr. Vol.)
1	8,681,446	2,344,128	380	0.414	22,845	6,168.759	16.337	4.627	46.667	0.489
2	20,696,333	12,004,029	440	0.634	47,037	27,281	83.663	11.031	23.5	2.506

Lane 7 - Vehicle

#	Vol. (Int.)	Local Bg. Corr. Vol.	Area	Rf	Density	Local Bg. Corr. Den.	% band purity	% lane purity	Mol. Wt.	Rel. Quant. (w/ LB Corr. Vol.)
1	11,050,480	4,790,483	370	0.417	29,866	12,947	24.848	6.122	46.333	1
2	24,930,091	14,488,606	533	0.637	46,773	27,183	75.152	13.812	23.333	3.024

Lane 8 - 5uM Ionomycin

#	Vol. (Int.)	Local Bg. Corr. Vol.	Area	Rf	Density	Local Bg. Corr. Den.	% band purity	% lane purity	Mol. Wt.	Rel. Quant. (w/ LB Corr. Vol.)
1	11,838,395	5,272,883	370	0.417	31,995	14,251	26.638	6.43	46.333	1.101
2	25,137,630	14,521,672	533	0.637	47,162	27,245	73.362	13.654	23.333	3.031

Lane 9 - 2uM Thapsigargin

#	Vol. (Int.)	Local Bg. Corr. Vol.	Area	Rf	Density	Local Bg. Corr. Den.	% band purity	% lane purity	Mol. Wt.	Rel. Quant. (w/ LB Corr. Vol.)
1	12,213,279	5,530,266	407	0.414	30,008	13,587	26.289	7.305	46.667	1.154
2	23,653,976	15,506,376	520	0.634	45,488	29,819	73.711	14.148	23.5	3.237
