# Supplementary material for: Elevation of Cytoplasmic Calcium Suppresses Microtentacle Formation and Function in Breast Tumor Cells
Source: Cancers (Basel). 2023 Jan 31;15(3):884. doi: 10.3390/cancers15030884 (PMC9913253; doi:10.3390/cancers15030884)
Supplement: Supplementary file 1 [file cancers-15-00884-s001.zip › cancers-2080686-Supplementary/File S1_ Original Blots/Original Immunoblot Images MDAMB231 Biological Replicate 2.pdf]

iBright™ Image Analysis Report

Katarina+ Chang

18 November 2022

Acetyl tubulin CHEMI_10152021_130328

Date:	15 October 2021 01:03:28PM
Mode:	Chemi Blots
Notes:	
Model:	FL1500
Instrument name:	2462619090234
Serial No:	2462619090234
Firmware version:	1.6.0
iBA version:	5.0
Image size:	676px X 540px
Image area:	132.59mm X 106.07mm
Optical Zoom:	1.7x
Digital Zoom:	1x
Focus level:	380
Resolution:	5 x 5
Exposure time:	5000 ms
Exposure mode:	Normal

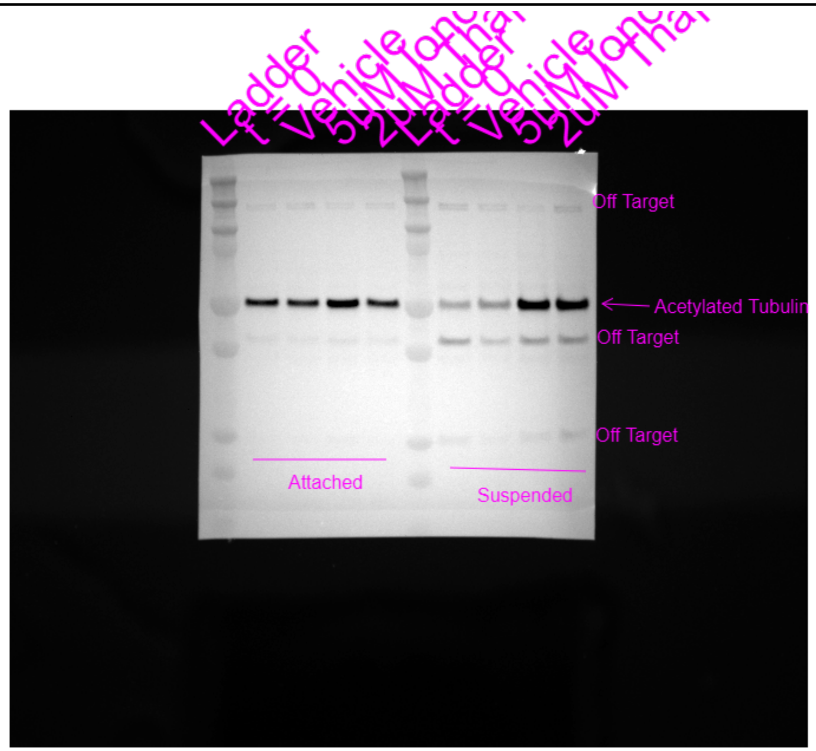

Acetyl tubulin CHEMI_10152021_13032 8

Date:	15 October 2021 01:03:28PM
Mode:	Chemi Blots
Notes:	
Model:	FL1500
Instrument name:	2462619090234
Serial No:	2462619090234
Firmware version:	1.6.0
iBA version:	5.0
Image size:	676px X 540px
Image area:	132.59mm X 106.07mm
Optical Zoom:	1.7x
Digital Zoom:	1x
Focus level:	380
Resolution:	5 x 5
Exposure time:	5000 ms
Exposure mode:	Normal

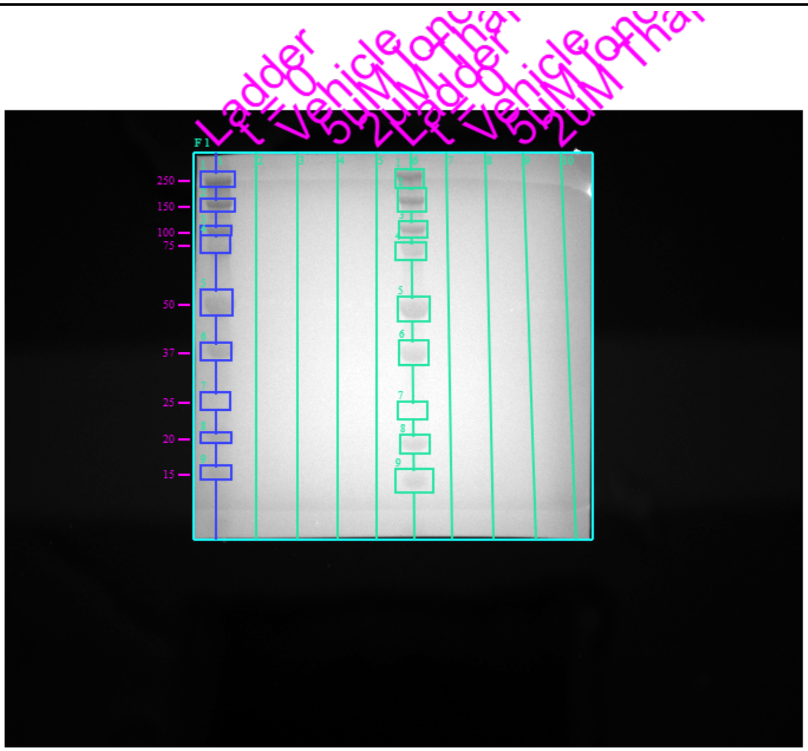

Acetyl tubulin CHEMI_10152021_13032 8

Date:	15 October 2021 01:03:28PM
Mode:	Chemi Blots
Notes:	
Model:	FL1500
Instrument name:	2462619090234
Serial No:	2462619090234
Firmware version:	1.6.0
iBA version:	5.0
Image size:	676px X 540px
Image area:	132.59mm X 106.07mm
Optical Zoom:	1.7x
Digital Zoom:	1x
Focus level:	380
Resolution:	5 x 5
Exposure time:	5000 ms
Exposure mode:	Normal

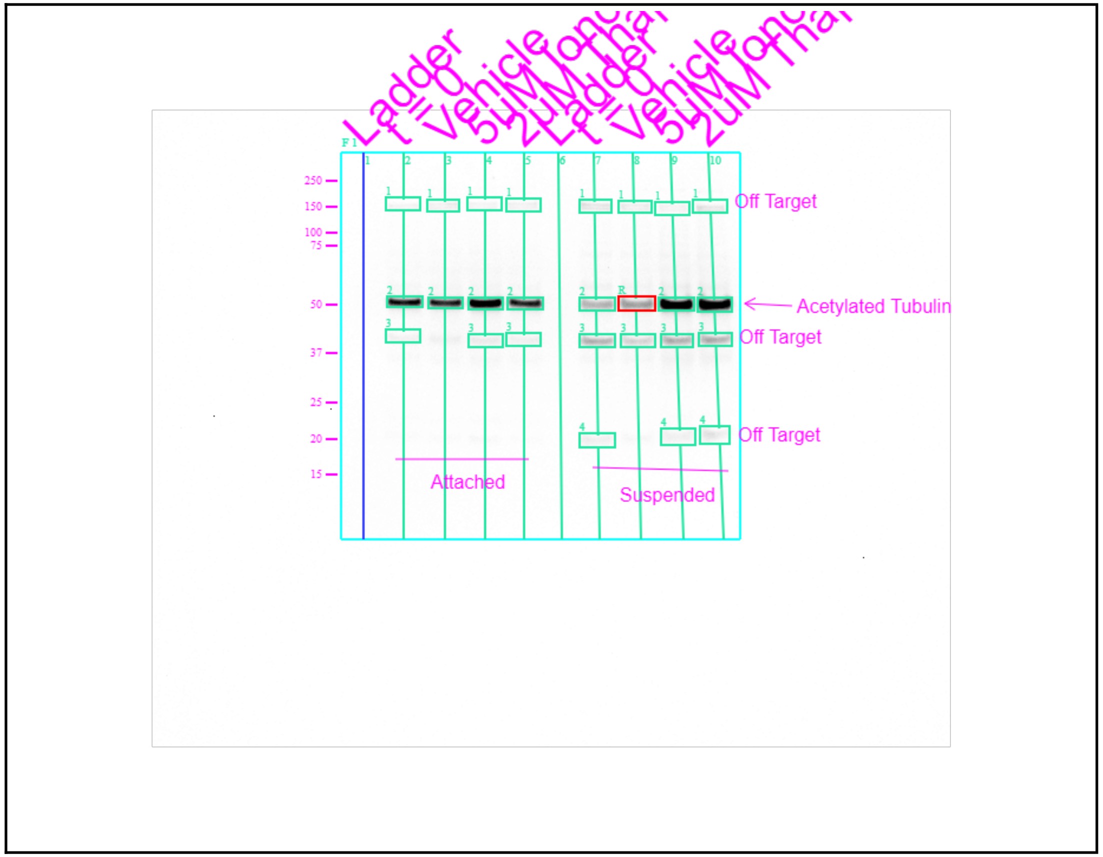

LANE AND BAND ANALYSIS DATA TABLE

Acetyl tubulin CHEMI_10152021_130328

Frame: 1
Channel: Membrane
Sensitivity: 100
Molecular Weight Analysis Regression Method : Point to Point

Lane 1 - Ladder

#	Vol. (Int.)	Local Bg. Corr. Vol.	Area	Rf	Density	Local Bg. Corr. Den.	% band purity	% lane purity	Mol. Wt.
1	15,849,920	824,650	420	0.067	37,737	1,963.454	14.045	-0.072	250
2	13,141,595	868,221	360	0.134	36,504	2,411.726	14.787	-0.059	150
3	8,806,005	705,485	243	0.201	36,238	2,903.233	12.016	-0.04	100
4	13,888,214	75,176	416	0.235	33,385	180.713	1.28	-0.063	75
5	20,407,430	1,370,429	644	0.387	31,688	2,127.996	23.341	-0.092	50
6	13,324,164	745,184	432	0.512	30,842	1,724.964	12.692	-0.06	37
7	12,010,099	203,321	416	0.64	28,870	488.753	3.463	-0.054	25
8	8,433,833	527,540	270	0.735	31,236	1,953.855	8.985	-0.038	20
9	11,109,241	551,325	351	0.826	31,650	1,570.728	9.39	-0.05	15

Lane 6 - Ladder

#	Vol. (Int.)	Local Bg. Corr. Vol.	Area	Rf	Density	Local Bg. Corr. Den.	% band purity	% lane purity	Mol. Wt.
1	14,093,088	1,839,989	425	0.067	33,160	4,329.386	17.004	0.964	250
2	15,638,031	1,664,388	525	0.122	29,786	3,170.264	15.382	1.07	168.182
3	10,261,398	1,363,892	375	0.198	27,363	3,637.047	12.605	0.702	102.273
4	10,385,669	836,063	432	0.253	24,040	1,935.331	7.727	0.71	72
5	13,845,566	1,831,220	616	0.402	22,476	2,972.761	16.923	0.947	48.415
6	11,826,888	1,374,642	572	0.515	20,676	2,403.221	12.704	0.809	36.714
7	7,574,836	168,184	416	0.665	18,208	404.289	1.554	0.518	23.71
8	9,241,792	935,550	442	0.753	20,909	2,116.629	8.646	0.632	19
9	15,166,568	806,751	693	0.848	21,885	1,164.143	7.456	1.037	NA

Frame: 1
Channel: Chemi
Sensitivity: 100
Molecular Weight Analysis Regression Method : Point to Point

Lane 2 - t = 0

#	Vol. (Int.)	Local Bg. Corr. Vol.	Area	Rf	Density	Local Bg. Corr. Den.	% band purity	% lane purity	Mol. Wt.	Rel. Quant. (w/ LB Corr. Vol.)
1	271,243	180,357	360	0.131	753.453	500.992	3.883	0.015	154.545	0.114
2	4,727,852	4,274,914	310	0.384	15,251	13,790	92.042	0.254	50.5	2.703
3	379,607	189,233	360	0.473	1,054.464	525.649	4.074	0.02	41.122	0.12

Lane 3 - Vehicle

#	Vol. (Int.)	Local Bg. Corr. Vol.	Area	Rf	Density	Local Bg. Corr. Den.	% band purity	% lane purity	Mol. Wt.	Rel. Quant. (w/ LB Corr. Vol.)
1	325,302	240,681	308	0.137	1,056.175	781.435	6.08	0.02	147.727	0.152
2	4,250,870	3,717,579	300	0.387	14,169	12,391	93.92	0.256	50	2.35

Lane 4 - 5uM Ionomycin

#	Vol. (Int.)	Local Bg. Corr. Vol.	Area	Rf	Density	Local Bg. Corr. Den.	% band purity	% lane purity	Mol. Wt.	Rel. Quant. (w/ LB Corr. Vol.)
1	254,446	153,299	360	0.131	706.794	425.832	2.316	0.017	154.545	0.097
2	6,867,429	6,196,373	330	0.39	20,810	18,776	93.597	0.466	49.683	3.917
3	547,635	270,626	360	0.485	1,521.208	751.74	4.088	0.037	39.854	0.171

Lane 5 - 2uM Thapsigargin

#	Vol. (Int.)	Local Bg. Corr. Vol.	Area	Rf	Density	Local Bg. Corr. Den.	% band purity	% lane purity	Mol. Wt.	Rel. Quant. (w/ LB Corr. Vol.)
1	307,442	211,409	360	0.134	854.006	587.249	4.794	0.024	150	0.134
2	4,497,399	3,993,943	341	0.39	13,188	11,712	90.564	0.349	49.683	2.525
3	446,557	204,732	360	0.482	1,240.436	568.7	4.642	0.035	40.171	0.129

Lane 7 - t = 0

#	Vol. (Int.)	Local Bg. Corr. Vol.	Area	Rf	Density	Local Bg. Corr. Den.	% band purity	% lane purity	Mol. Wt.	Rel. Quant. (w/ LB Corr. Vol.)
1	520,242	417,514	336	0.137	1,548.339	1,242.603	12.11	0.058	147.727	0.264
2	1,860,077	1,414,259	372	0.39	5,000.207	3,801.774	41.022	0.208	49.683	0.894
3	1,566,959	1,297,400	372	0.485	4,212.255	3,487.635	37.632	0.175	39.854	0.82

#	Vol. (Int.)	Local Bg. Corr. Vol.	Area	Rf	Density	Local Bg. Corr. Den.	% band purity	% lane purity	Mol. Wt.	Rel. Quant. (w/ LB Corr. Vol.)
4	450,069	318,422	403	0.744	1,116.797	790.129	9.236	0.05	19.5	0.201

Lane 8 - Vehicle

#	Vol. (Int.)	Local Bg. Corr. Vol.	Area	Rf	Density	Local Bg. Corr. Den.	% band purity	% lane purity	Mol. Wt.	Rel. Quant. (w/ LB Corr. Vol.)
1	371,617	267,709	319	0.14	1,164.944	839.215	10.78	0.053	145.455	0.169
2	2,439,774	1,581,816	416	0.39	5,864.841	3,802.444	63.695	0.351	49.683	1
3	958,675	633,900	360	0.485	2,662.986	1,760.834	25.525	0.138	39.854	0.401

Lane 9 - 5uM Ionomycin

#	Vol. (Int.)	Local Bg. Corr. Vol.	Area	Rf	Density	Local Bg. Corr. Den.	% band purity	% lane purity	Mol. Wt.	Rel. Quant. (w/ LB Corr. Vol.)
1	303,217	157,233	360	0.143	842.269	436.76	1.872	0.063	143.182	0.099
2	8,034,797	6,716,777	372	0.39	21,598	18,055	79.949	1.665	49.683	4.246
3	1,619,762	1,224,049	336	0.485	4,820.72	3,643.005	14.57	0.336	39.854	0.774
4	480,172	303,277	450	0.735	1,067.049	673.949	3.61	0.1	20	0.192

Lane 10 - 2uM Thapsigargin

#	Vol. (Int.)	Local Bg. Corr. Vol.	Area	Rf	Density	Local Bg. Corr. Den.	% band purity	% lane purity	Mol. Wt.	Rel. Quant. (w/ LB Corr. Vol.)
1	522,017	383,510	360	0.137	1,450.047	1,065.307	4.115	0.234	147.727	0.242
2	8,165,200	7,232,408	390	0.393	20,936	18,544	77.605	3.661	49.366	4.572
3	1,609,488	1,269,869	348	0.482	4,624.966	3,649.05	13.626	0.722	40.171	0.803
4	558,535	433,728	416	0.729	1,342.632	1,042.615	4.654	0.25	20.323	0.274

iBright™ Image Analysis Report

Katarina+ Chang

18 November 2022

GAPDH_CHEMI_10162021_142115

Date:	16 October 2021 02:21:15PM
Mode:	Chemi Blots
Notes:	
Model:	FL1500
Instrument name:	2462619090234
Serial No:	2462619090234
Firmware version:	1.6.0
iBA version:	5.0
Image size:	563px X 450px
Image area:	112.7mm X 90.16mm
Optical Zoom:	2x
Digital Zoom:	1.2x
Focus level:	455
Resolution:	5 x 5
Exposure time:	28182 ms
Exposure mode:	Normal

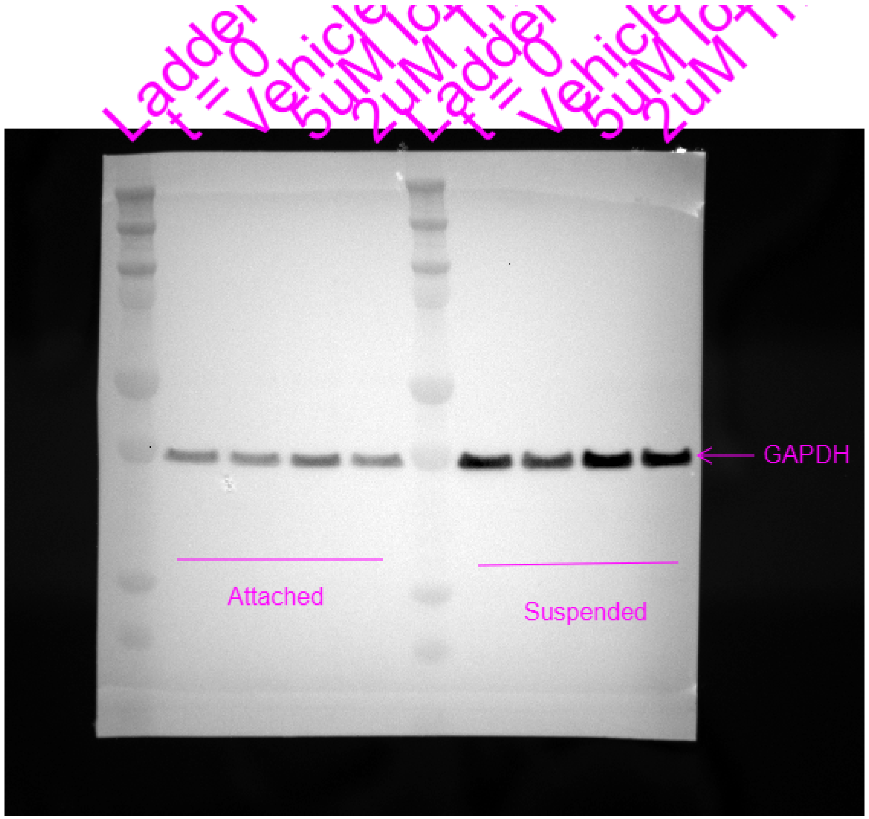

GAPDH_CHEMI_10162021_142115

Date:	16 October 2021 02:21:15PM
Mode:	Chemi Blots
Notes:	
Model:	FL1500
Instrument name:	2462619090234
Serial No:	2462619090234
Firmware version:	1.6.0
iBA version:	5.0
Image size:	563px X 450px
Image area:	112.7mm X 90.16mm
Optical Zoom:	2x
Digital Zoom:	1.2x
Focus level:	455
Resolution:	5 x 5
Exposure time:	28182 ms
Exposure mode:	Normal

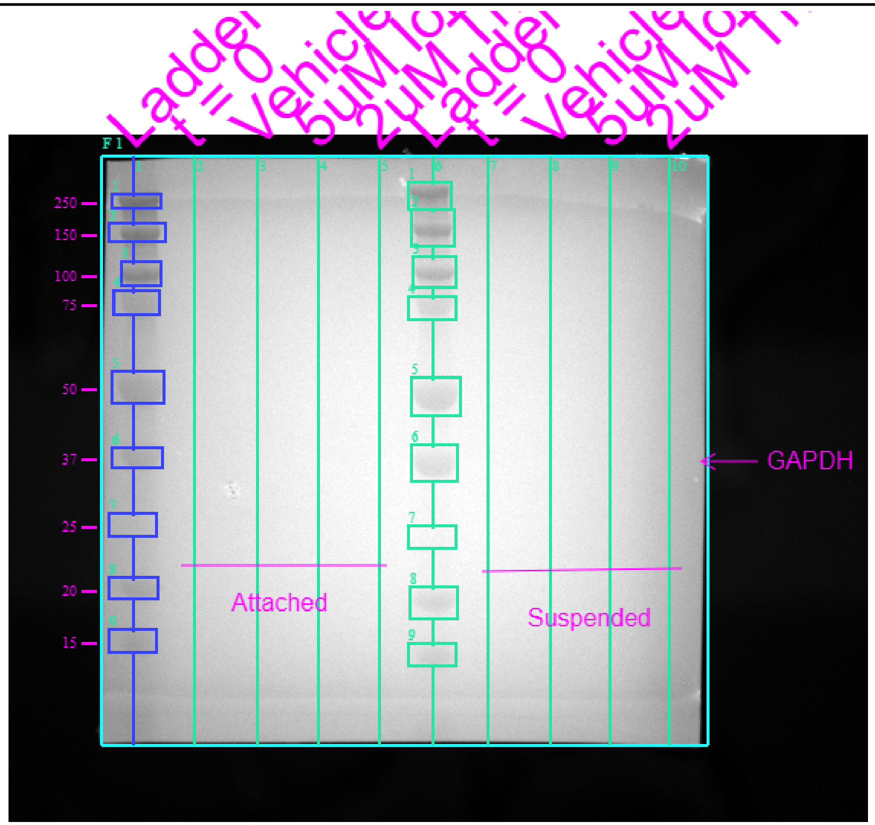

GAPDH_CHEMI_10162021_142115

Date:	16 October 2021 02:21:15PM
Mode:	Chemi Blots
Notes:	
Model:	FL1500
Instrument name:	2462619090234
Serial No:	2462619090234
Firmware version:	1.6.0
iBA version:	5.0
Image size:	563px X 450px
Image area:	112.7mm X 90.16mm
Optical Zoom:	2x
Digital Zoom:	1.2x
Focus level:	455
Resolution:	5 x 5
Exposure time:	28182 ms
Exposure mode:	Normal

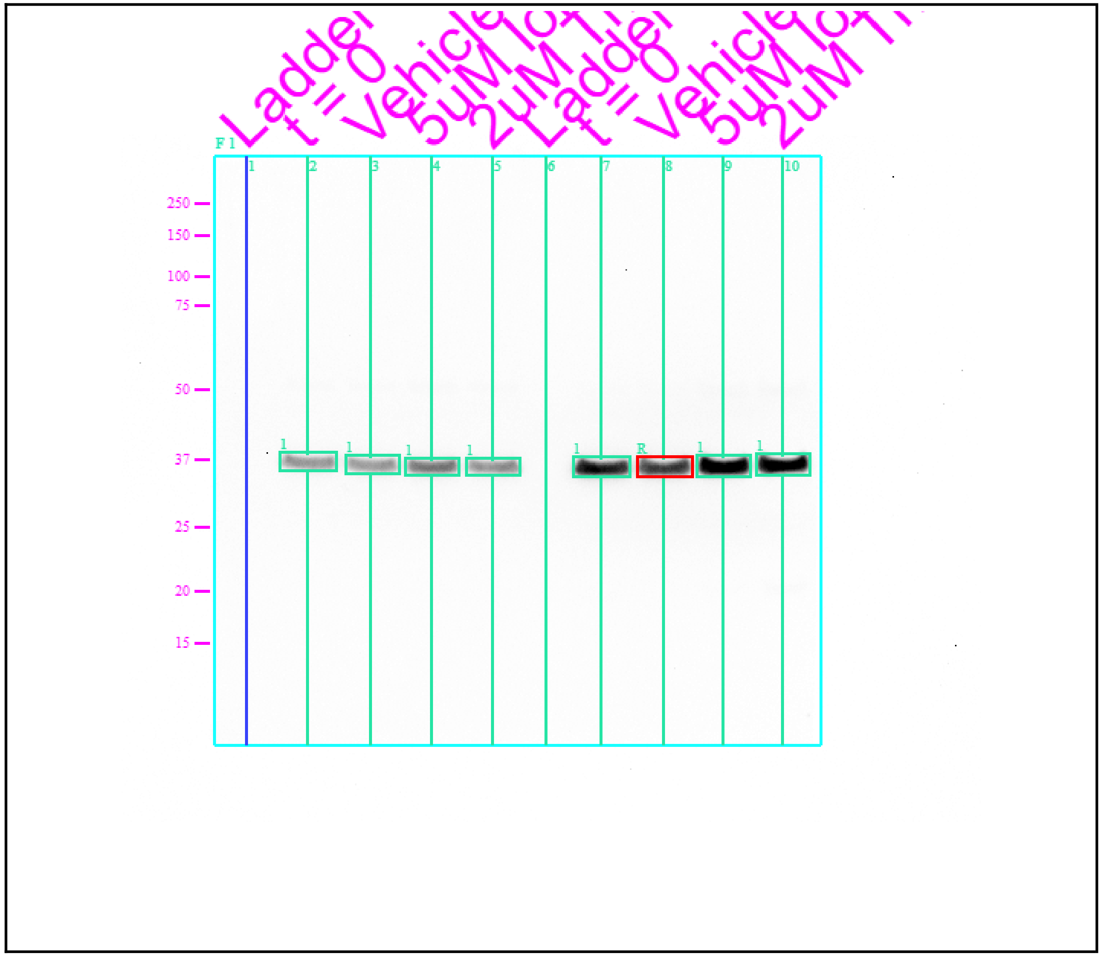

LANE AND BAND ANALYSIS DATA TABLE

GAPDH_CHEMI_10162021_142115

Frame: 1
Channel: Membrane
Sensitivity: 100
Molecular Weight Analysis Regression Method : Point to Point

Lane 1 - Ladder

#	Vol. (Int.)	Local Bg. Corr. Vol.	Area	Rf	Density	Local Bg. Corr. Den.	% band purity	% lane purity	Mol. Wt.
1	13,231,481	1,158,319	330	0.075	40,095	3,510.059	13.51	NA	250
2	18,381,190	676,136	494	0.13	37,208	1,368.697	7.886	NA	150
3	16,575,975	1,722,638	459	0.199	36,113	3,753.025	20.092	NA	100
4	17,756,692	814,782	527	0.249	33,693	1,546.077	9.503	NA	75
5	25,239,479	1,845,358	770	0.391	32,778	2,396.57	21.524	NA	50
6	15,367,495	926,200	476	0.51	32,284	1,945.8	10.803	NA	37
7	15,832,980	111,016	512	0.624	30,923	216.83	1.295	NA	25
8	16,457,762	711,882	495	0.733	33,248	1,438.147	8.303	NA	20
9	17,472,754	607,291	512	0.821	34,126	1,186.116	7.083	NA	15

Lane 6 - Ladder

#	Vol. (Int.)	Local Bg. Corr. Vol.	Area	Rf	Density	Local Bg. Corr. Den.	% band purity	% lane purity	Mol. Wt.
1	18,201,263	2,543,407	551	0.067	33,033	4,615.984	18.931	NA	NA
2	21,406,590	2,174,203	725	0.122	29,526	2,998.902	16.183	NA	164.286
3	16,125,184	1,796,593	609	0.197	26,478	2,950.071	13.372	NA	101.852
4	11,982,897	847,906	512	0.256	23,404	1,656.067	6.311	NA	73.636
5	19,215,650	2,235,908	858	0.407	22,395	2,605.954	16.642	NA	48.304
6	16,554,580	1,712,990	775	0.521	21,360	2,210.311	12.75	NA	35.909
7	10,085,310	198,062	512	0.645	19,697	386.84	1.474	NA	24.048
8	15,996,535	1,196,793	704	0.756	22,722	1,699.99	8.908	NA	18.676
9	12,789,908	729,144	512	0.845	24,980	1,424.111	5.427	NA	NA

Frame: 1
Channel: Chemi
Sensitivity: 100
Molecular Weight Analysis Regression Method : Point to Point

Lane 2 - t = 0

#	Vol. (Int.)	Local Bg. Corr. Vol.	Area	Rf	Density	Local Bg. Corr. Den.	% band purity	% lane purity	Mol. Wt.	Rel. Quant. (w/ LB Corr. Vol.)
1	4,745,840	3,989,484	494	0.518	9,606.964	8,075.88	100	NA	36.182	0.522

Lane 3 - Vehicle

#	Vol. (Int.)	Local Bg. Corr. Vol.	Area	Rf	Density	Local Bg. Corr. Den.	% band purity	% lane purity	Mol. Wt.	Rel. Quant. (w/ LB Corr. Vol.)
1	4,412,639	3,522,651	468	0.523	9,428.716	7,527.033	100	NA	35.636	0.461

Lane 4 - 5uM Ionomycin

#	Vol. (Int.)	Local Bg. Corr. Vol.	Area	Rf	Density	Local Bg. Corr. Den.	% band purity	% lane purity	Mol. Wt.	Rel. Quant. (w/ LB Corr. Vol.)
1	5,775,788	4,866,061	432	0.526	13,369	11,264	100	NA	35.364	0.637

Lane 5 - 2uM Thapsigargin

#	Vol. (Int.)	Local Bg. Corr. Vol.	Area	Rf	Density	Local Bg. Corr. Den.	% band purity	% lane purity	Mol. Wt.	Rel. Quant. (w/ LB Corr. Vol.)
1	4,694,665	3,927,169	432	0.526	10,867	9,090.67	100	NA	35.364	0.514

Lane 7 - t = 0

#	Vol. (Int.)	Local Bg. Corr. Vol.	Area	Rf	Density	Local Bg. Corr. Den.	% band purity	% lane purity	Mol. Wt.	Rel. Quant. (w/ LB Corr. Vol.)
1	11,652,713	10,154,568	532	0.526	21,903	19,087	100	NA	35.364	1.329

Lane 8 - Vehicle

#	Vol. (Int.)	Local Bg. Corr. Vol.	Area	Rf	Density	Local Bg. Corr. Den.	% band purity	% lane purity	Mol. Wt.	Rel. Quant. (w/ LB Corr. Vol.)
1	9,585,416	7,642,077	518	0.526	18,504	14,753	100	NA	35.364	1

Lane 9 - 5uM Ionomycin

#	Vol. (Int.)	Local Bg. Corr. Vol.	Area	Rf	Density	Local Bg. Corr. Den.	% band purity	% lane purity	Mol. Wt.	Rel. Quant. (w/ LB Corr. Vol.)
1	14,280,131	12,619,798	540	0.526	26,444	23,369	100	NA	35.364	1.651

Lane 10 - 2uM Thapsigargin

#	Vol. (Int.)	Local Bg. Corr. Vol.	Area	Rf	Density	Local Bg. Corr. Den.	% band purity	% lane purity	Mol. Wt.	Rel. Quant. (w/ LB Corr. Vol.)
1	13,112,019	12,193,168	540	0.523	24,281	22,579	100	NA	35.636	1.596

iBright™ Image Analysis Report

Katarina+ Chang

18 November 2022

pMLC_CHEMI_10142021_151106

Date:	14 October 2021 03:11:06PM
Mode:	Chemi Blots
Notes:	
Model:	FL1500
Instrument name:	2462619090234
Serial No:	2462619090234
Firmware version:	1.6.0
iBA version:	5.0
Image size:	676px X 540px
Image area:	112.7mm X 90.16mm
Optical Zoom:	2x
Digital Zoom:	1x
Focus level:	455
Resolution:	5 x 5
Exposure time:	16271 ms
Exposure mode:	Normal

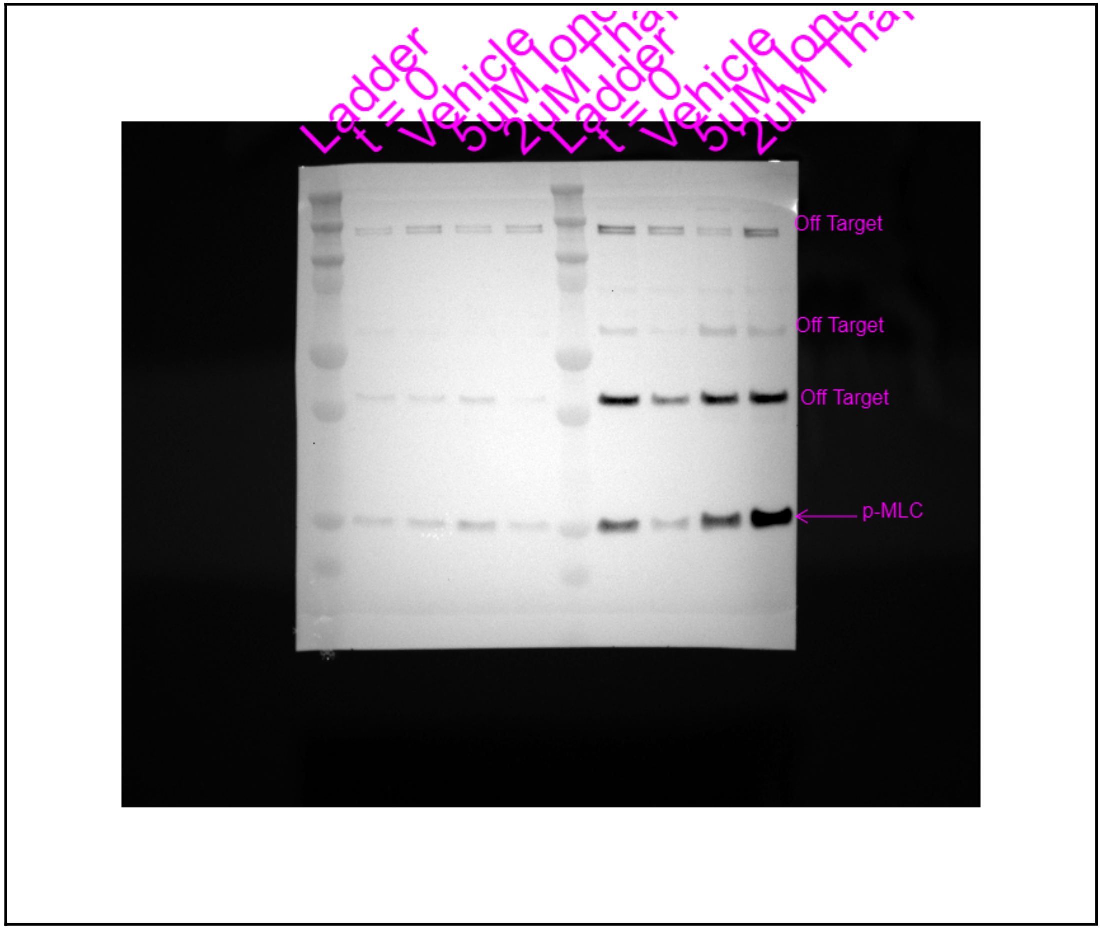

pMLC_CHEMI_10142021_151106

Date:	14 October 2021 03:11:06PM
Mode:	Chemi Blots
Notes:	
Model:	FL1500
Instrument name:	2462619090234
Serial No:	2462619090234
Firmware version:	1.6.0
iBA version:	5.0
Image size:	676px X 540px
Image area:	112.7mm X 90.16mm
Optical Zoom:	2x
Digital Zoom:	1x
Focus level:	455
Resolution:	5 x 5
Exposure time:	16271 ms
Exposure mode:	Normal

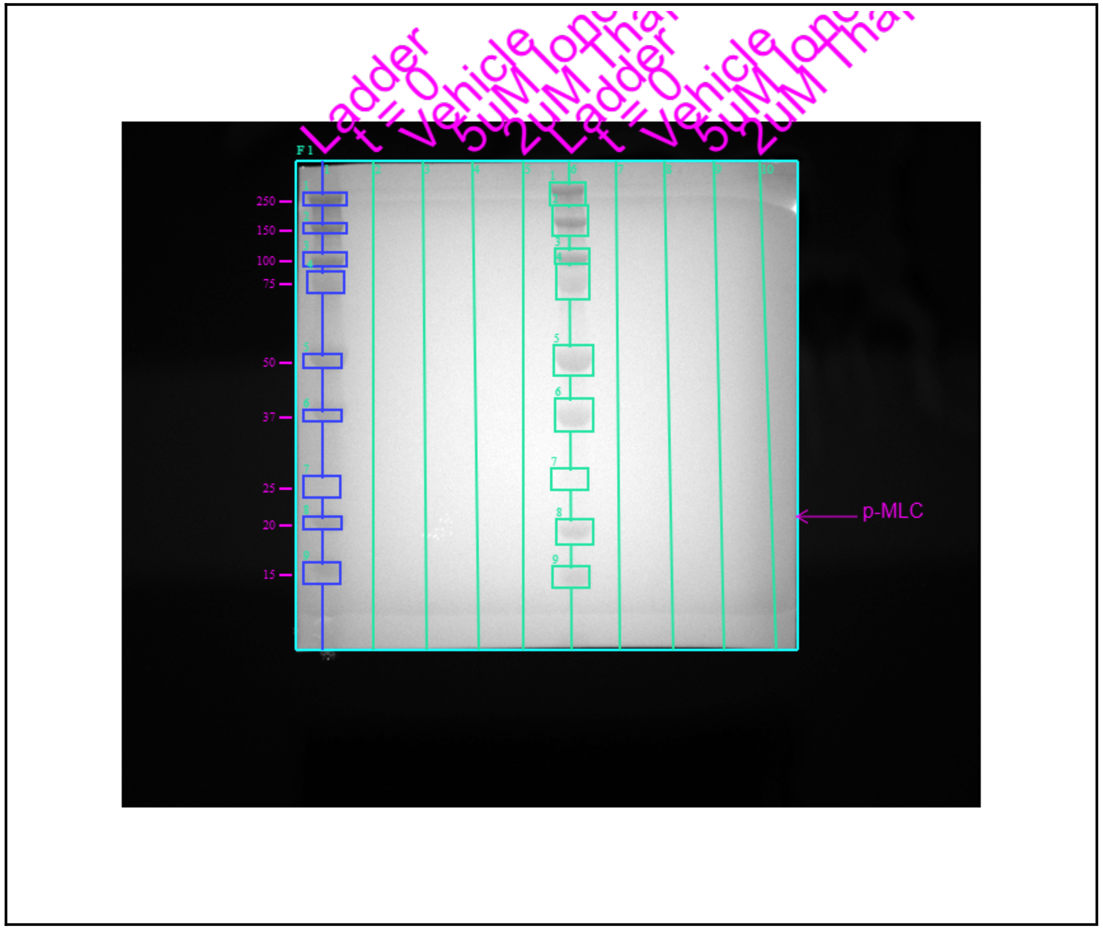

pMLC_CHEMI_10142021_151106

Date:	14 October 2021 03:11:06PM
Mode:	Chemi Blots
Notes:	
Model:	FL1500
Instrument name:	2462619090234
Serial No:	2462619090234
Firmware version:	1.6.0
iBA version:	5.0
Image size:	676px X 540px
Image area:	112.7mm X 90.16mm
Optical Zoom:	2x
Digital Zoom:	1x
Focus level:	455
Resolution:	5 x 5
Exposure time:	16271 ms
Exposure mode:	Normal

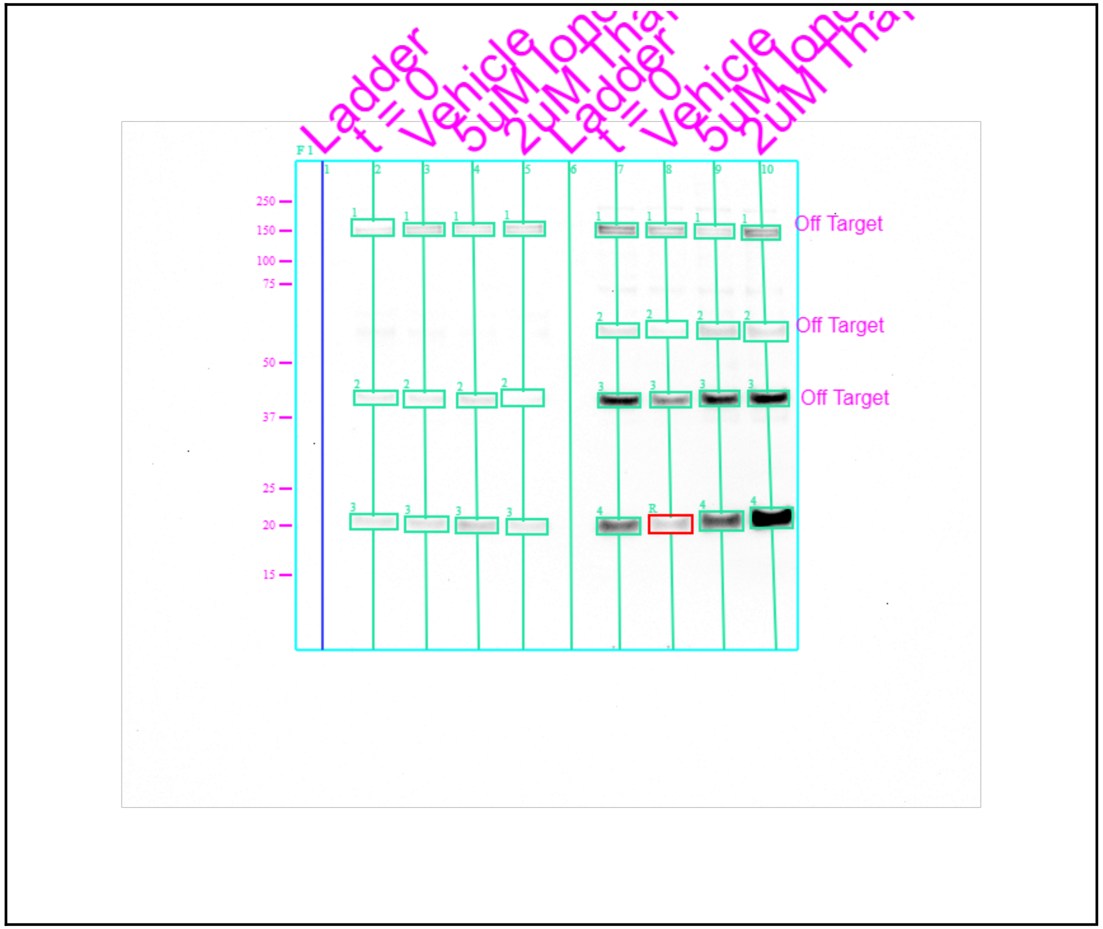

LANE AND BAND ANALYSIS DATA TABLE

pMLC_CHEMI_10142021_151106

Frame: 1
Channel: Membrane
Sensitivity: 100
Molecular Weight Analysis Regression Method : Point to Point

Lane 1 - Ladder

#	Vol. (Int.)	Local Bg. Corr. Vol.	Area	Rf	Density	Local Bg. Corr. Den.	% band purity	% lane purity	Mol. Wt.
1	15,304,990	1,258,122	385	0.078	39,753	3,267.85	17.819	3.793	250
2	12,114,999	1,117,518	315	0.138	38,460	3,547.678	15.827	3.003	150
3	15,226,844	1,220,863	420	0.2	36,254	2,906.818	17.291	3.774	100
4	18,336,158	907,839	540	0.247	33,955	1,681.185	12.858	4.545	75
5	12,562,399	784,331	372	0.408	33,769	2,108.419	11.108	3.114	50
6	10,094,865	460,798	310	0.519	32,564	1,486.446	6.526	2.502	37
7	16,459,255	171,554	540	0.665	30,480	317.694	2.43	4.079	25
8	11,334,276	644,175	341	0.74	33,238	1,889.077	9.123	2.809	20
9	18,116,994	495,488	540	0.842	33,549	917.571	7.018	4.49	15

Lane 6 - Ladder

#	Vol. (Int.)	Local Bg. Corr. Vol.	Area	Rf	Density	Local Bg. Corr. Den.	% band purity	% lane purity	Mol. Wt.
1	18,687,845	2,356,715	551	0.068	33,916	4,277.161	16.599	6.323	NA
2	22,144,539	2,317,818	725	0.122	30,544	3,196.991	16.325	7.493	176.087
3	10,571,311	1,582,773	364	0.195	29,042	4,348.28	11.148	3.577	104.167
4	19,771,277	1,139,828	783	0.247	25,250	1,455.72	8.028	6.69	75
5	18,709,611	2,471,136	800	0.408	23,387	3,088.921	17.405	6.331	50
6	18,175,940	1,917,941	837	0.519	21,715	2,291.447	13.509	6.15	37
7	10,651,447	276,606	540	0.649	19,724	512.234	1.948	3.604	26.286
8	14,389,582	1,320,236	630	0.758	22,840	2,095.614	9.299	4.869	19.103
9	13,320,023	814,811	540	0.849	24,666	1,508.91	5.739	4.507	NA

Frame: 1
Channel: Chemi
Sensitivity: 100
Molecular Weight Analysis Regression Method : Point to Point

Lane 2 - t = 0

#	Vol. (Int.)	Local Bg. Corr. Vol.	Area	Rf	Density	Local Bg. Corr. Den.	% band purity	% lane purity	Mol. Wt.	Rel. Quant. (w/ LB Corr. Vol.)
1	358,421	318,952	476	0.135	752.985	670.068	29.692	13.916	154.348	0.342
2	325,230	261,619	420	0.483	774.357	622.903	24.355	12.627	41.233	0.28
3	587,223	493,623	494	0.738	1,188.711	999.238	45.953	22.799	20.172	0.529

Lane 3 - Vehicle

#	Vol. (Int.)	Local Bg. Corr. Vol.	Area	Rf	Density	Local Bg. Corr. Den.	% band purity	% lane purity	Mol. Wt.	Rel. Quant. (w/ LB Corr. Vol.)
1	645,913	608,197	363	0.14	1,779.375	1,675.476	42.215	20.886	147.917	0.651
2	345,014	261,314	462	0.486	746.784	565.617	18.138	11.156	40.93	0.28
3	698,556	571,190	455	0.743	1,535.288	1,255.364	39.647	22.588	19.872	0.612

Lane 4 - 5uM Ionomycin

#	Vol. (Int.)	Local Bg. Corr. Vol.	Area	Rf	Density	Local Bg. Corr. Den.	% band purity	% lane purity	Mol. Wt.	Rel. Quant. (w/ LB Corr. Vol.)
1	399,126	364,721	363	0.14	1,099.521	1,004.743	21.389	11.953	147.917	0.391
2	469,576	399,028	384	0.488	1,222.854	1,039.138	23.401	14.063	40.628	0.427
3	1,097,198	941,452	442	0.745	2,482.348	2,129.982	55.211	32.859	19.744	1.008

Lane 5 - 2uM Thapsigargin

#	Vol. (Int.)	Local Bg. Corr. Vol.	Area	Rf	Density	Local Bg. Corr. Den.	% band purity	% lane purity	Mol. Wt.	Rel. Quant. (w/ LB Corr. Vol.)
1	598,488	560,027	396	0.138	1,511.333	1,414.212	52.72	23.14	150	0.6
2	172,216	96,822	476	0.483	361.798	203.408	9.115	6.659	41.233	0.104
3	540,181	405,418	429	0.748	1,259.163	945.03	38.165	20.886	19.615	0.434

Lane 7 - t = 0

#	Vol. (Int.)	Local Bg. Corr. Vol.	Area	Rf	Density	Local Bg. Corr. Den.	% band purity	% lane purity	Mol. Wt.	Rel. Quant. (w/ LB Corr. Vol.)
1	1,806,392	1,717,467	408	0.14	4,427.431	4,209.479	16.808	12.999	147.917	1.839
2	519,463	427,846	408	0.345	1,273.194	1,048.644	4.187	3.738	59.677	0.458

#	Vol. (Int.)	Local Bg. Corr. Vol.	Area	Rf	Density	Local Bg. Corr. Den.	% band purity	% lane purity	Mol. Wt.	Rel. Quant. (w/ LB Corr. Vol.)
3	4,935,697	4,553,249	408	0.488	12,097	11,159	44.561	35.517	40.628	4.876
4	3,961,259	3,519,425	490	0.745	8,084.202	7,182.501	34.443	28.505	19.744	3.769

Lane 8 - Vehicle

#	Vol. (Int.)	Local Bg. Corr. Vol.	Area	Rf	Density	Local Bg. Corr. Den.	% band purity	% lane purity	Mol. Wt.	Rel. Quant. (w/ LB Corr. Vol.)
1	967,973	904,623	384	0.14	2,520.763	2,355.789	22.76	12.427	147.917	0.969
2	265,203	147,020	462	0.343	574.032	318.226	3.699	3.405	60.081	0.157
3	2,318,829	1,989,056	429	0.488	5,405.196	4,636.496	50.045	29.769	40.628	2.13
4	1,305,286	933,858	525	0.743	2,486.259	1,778.778	23.496	16.757	19.872	1

Lane 9 - 5uM Ionomycin

#	Vol. (Int.)	Local Bg. Corr. Vol.	Area	Rf	Density	Local Bg. Corr. Den.	% band purity	% lane purity	Mol. Wt.	Rel. Quant. (w/ LB Corr. Vol.)
1	535,968	437,880	384	0.143	1,395.75	1,140.314	4.426	3.337	145.833	0.469
2	946,769	708,676	476	0.345	1,989.011	1,488.817	7.163	5.895	59.677	0.759
3	4,775,677	4,233,117	416	0.486	11,479	10,175	42.786	29.733	40.93	4.533
4	5,389,811	4,514,100	560	0.735	9,624.663	8,060.894	45.626	33.557	20.345	4.834

Lane 10 - 2uM Thapsigargin

#	Vol. (Int.)	Local Bg. Corr. Vol.	Area	Rf	Density	Local Bg. Corr. Den.	% band purity	% lane purity	Mol. Wt.	Rel. Quant. (w/ LB Corr. Vol.)
1	1,658,165	1,531,185	372	0.145	4,457.433	4,116.09	7.42	6.239	143.75	1.64
2	755,572	539,715	560	0.348	1,349.236	963.778	2.615	2.843	59.274	0.578
3	6,037,936	5,502,253	429	0.486	14,074	12,825	26.663	22.719	40.93	5.892
4	14,107,593	13,063,209	578	0.73	24,407	22,600	63.302	53.082	20.69	13.988

iBright™ Image Analysis Report

Katarina+ Chang
18 November 2022

pMYTP1_CHEMI_10132021_120935

Date:	13 October 2021 12:09:35PM
Mode:	Chemi Blots
Notes:	
Model:	FL1500
Instrument name:	2462619090234
Serial No:	2462619090234
Firmware version:	1.6.0
iBA version:	5.0
Image size:	676px X 540px
Image area:	112.7mm X 90.16mm
Optical Zoom:	2x
Digital Zoom:	1x
Focus level:	455
Resolution:	5 x 5
Exposure time:	9085 ms
Exposure mode:	Normal

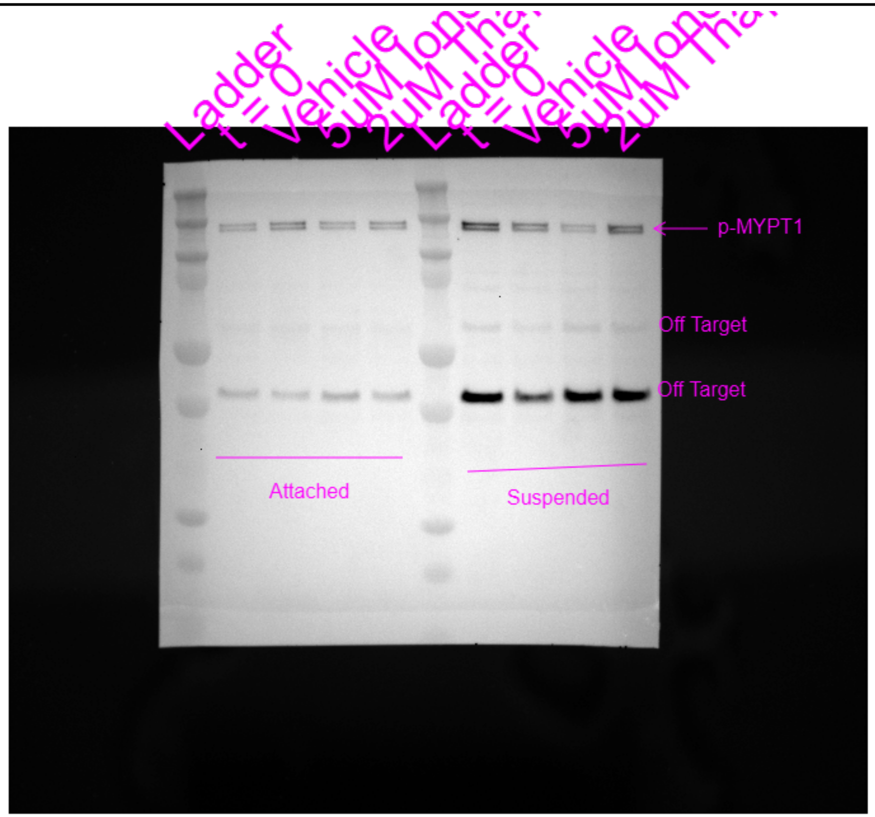

pMYTP1_CHEMI_10132021_120935

Date:	13 October 2021 12:09:35PM
Mode:	Chemi Blots
Notes:	
Model:	FL1500
Instrument name:	2462619090234
Serial No:	2462619090234
Firmware version:	1.6.0
iBA version:	5.0
Image size:	676px X 540px
Image area:	112.7mm X 90.16mm
Optical Zoom:	2x
Digital Zoom:	1x
Focus level:	455
Resolution:	5 x 5
Exposure time:	9085 ms
Exposure mode:	Normal

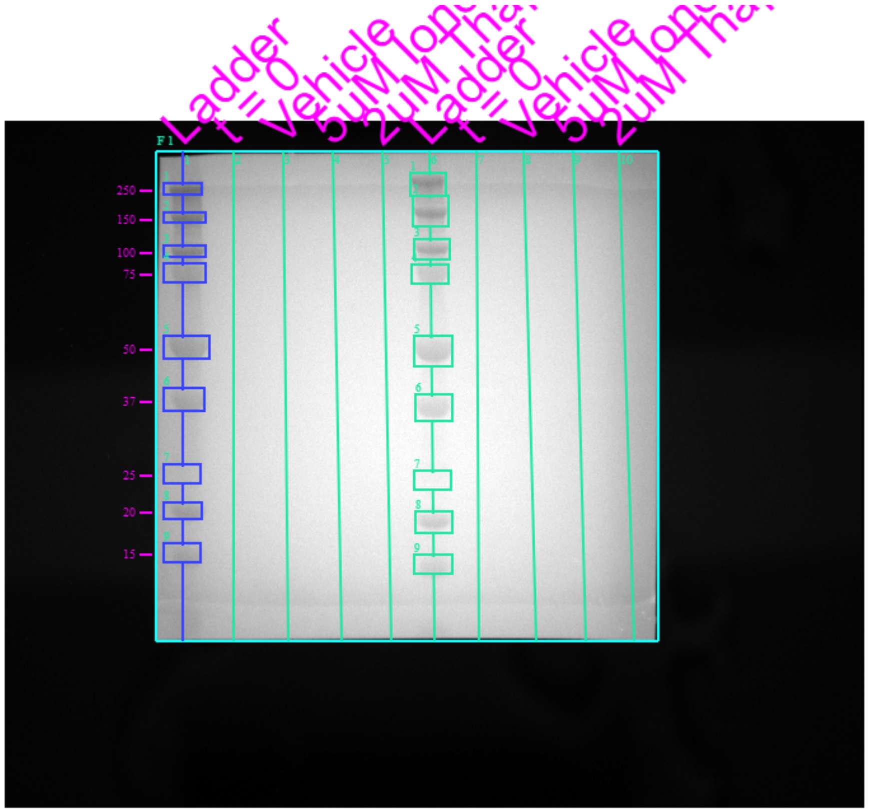

pMYTP1_CHEMI_10132021_120935

Date:	13 October 2021 12:09:35PM
Mode:	Chemi Blots
Notes:	
Model:	FL1500
Instrument name:	2462619090234
Serial No:	2462619090234
Firmware version:	1.6.0
iBA version:	5.0
Image size:	676px X 540px
Image area:	112.7mm X 90.16mm
Optical Zoom:	2x
Digital Zoom:	1x
Focus level:	455
Resolution:	5 x 5
Exposure time:	9085 ms
Exposure mode:	Normal

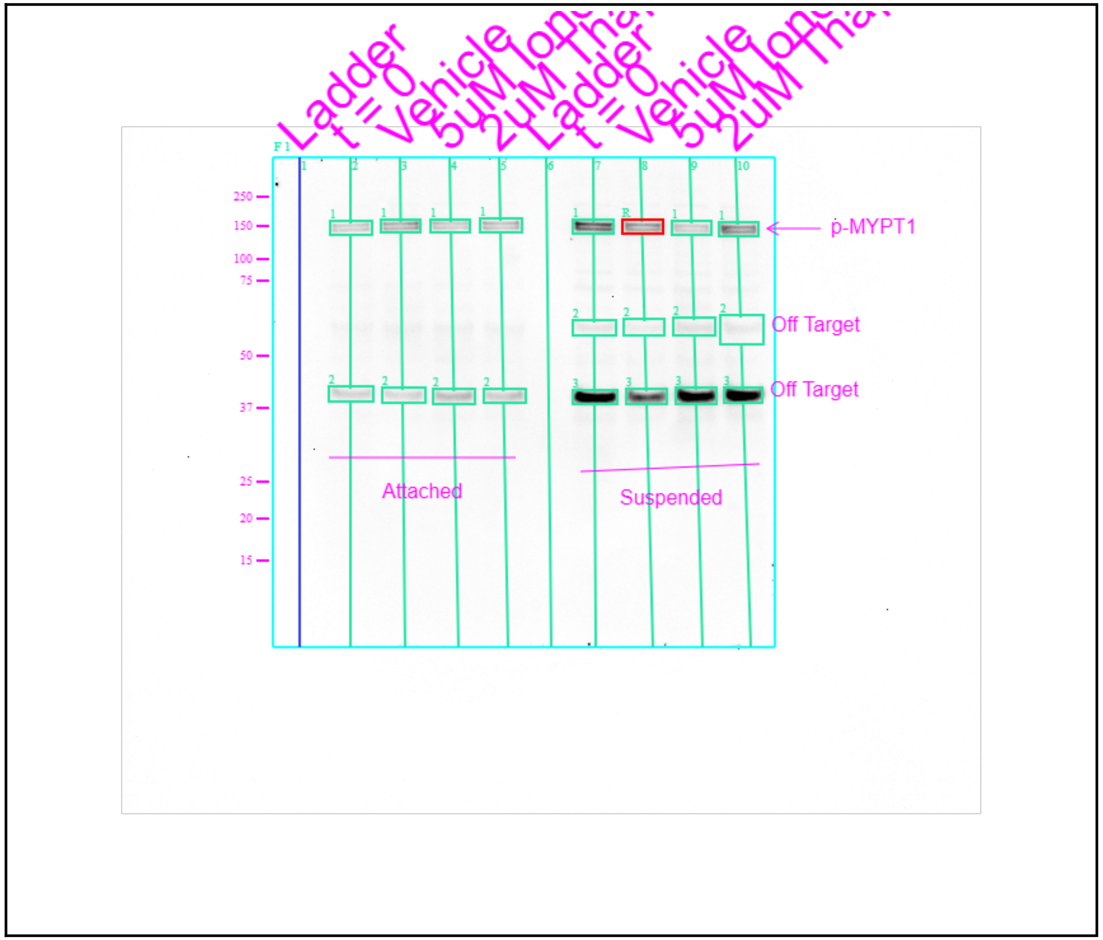

LANE AND BAND ANALYSIS DATA TABLE

pMYTP1_CHEMI_10132021_120935

Frame: 1
Channel: Membrane
Sensitivity: 100
Molecular Weight Analysis Regression Method : Point to Point

Lane 1 - Ladder

#	Vol. (Int.)	Local Bg. Corr. Vol.	Area	Rf	Density	Local Bg. Corr. Den.	% band purity	% lane purity	Mol. Wt.
1	12,951,724	917,848	310	0.075	41,779	2,960.801	9.627	3.242	250
2	12,108,329	968,370	306	0.135	39,569	3,164.609	10.157	3.031	150
3	12,796,375	1,098,426	340	0.203	37,636	3,230.666	11.521	3.203	100
4	18,943,816	775,534	544	0.247	34,823	1,425.615	8.134	4.742	75
5	23,673,993	2,184,499	703	0.4	33,675	3,107.396	22.912	5.926	50
6	20,483,587	1,383,673	627	0.506	32,669	2,206.816	14.513	5.127	37
7	14,855,477	302,198	480	0.657	30,948	629.579	3.17	3.718	25
8	14,713,000	1,198,373	434	0.732	33,900	2,761.23	12.569	3.683	20
9	16,176,799	705,191	480	0.818	33,701	1,469.149	7.397	4.049	15

Lane 6 - Ladder

#	Vol. (Int.)	Local Bg. Corr. Vol.	Area	Rf	Density	Local Bg. Corr. Den.	% band purity	% lane purity	Mol. Wt.
1	18,935,045	2,276,381	551	0.068	34,364	4,131.364	15.382	6.555	NA
2	22,416,555	2,219,395	725	0.122	30,919	3,061.235	14.997	7.761	171.739
3	14,062,710	1,832,656	493	0.2	28,524	3,717.357	12.384	4.869	101.923
4	12,426,001	1,219,826	480	0.249	25,887	2,541.305	8.243	4.302	74.576
5	18,595,663	2,739,443	775	0.408	23,994	3,534.765	18.511	6.438	49.049
6	14,774,974	1,927,158	660	0.522	22,386	2,919.937	13.022	5.115	35.759
7	9,523,507	197,669	480	0.67	19,840	411.812	1.336	3.297	24.138
8	12,651,008	1,525,127	540	0.756	23,427	2,824.311	10.306	4.38	18.636
9	12,105,657	861,303	496	0.842	24,406	1,736.499	5.82	4.191	NA

Frame: 1
Channel: Chemi
Sensitivity: 100
Molecular Weight Analysis Regression Method : Point to Point

Lane 2 - t = 0

#	Vol. (Int.)	Local Bg. Corr. Vol.	Area	Rf	Density	Local Bg. Corr. Den.	% band purity	% lane purity	Mol. Wt.	Rel. Quant. (w/ LB Corr. Vol.)
1	1,423,061	1,061,504	408	0.143	3,487.895	2,601.726	50.745	13.81	144.231	0.509
2	1,461,417	1,030,330	468	0.483	3,122.686	2,201.561	49.255	14.182	39.854	0.494

Lane 3 - Vehicle

#	Vol. (Int.)	Local Bg. Corr. Vol.	Area	Rf	Density	Local Bg. Corr. Den.	% band purity	% lane purity	Mol. Wt.	Rel. Quant. (w/ LB Corr. Vol.)
1	2,066,603	1,659,834	352	0.14	5,871.031	4,715.438	66.769	17.583	146.154	0.797
2	1,281,779	826,084	455	0.486	2,817.097	1,815.57	33.231	10.906	39.537	0.396

Lane 4 - 5uM Ionomycin

#	Vol. (Int.)	Local Bg. Corr. Vol.	Area	Rf	Density	Local Bg. Corr. Den.	% band purity	% lane purity	Mol. Wt.	Rel. Quant. (w/ LB Corr. Vol.)
1	1,401,959	1,074,678	352	0.14	3,982.838	3,053.063	42.009	11.59	146.154	0.516
2	2,004,065	1,483,513	442	0.488	4,534.084	3,356.366	57.991	16.568	39.22	0.712

Lane 5 - 2uM Thapsigargin

#	Vol. (Int.)	Local Bg. Corr. Vol.	Area	Rf	Density	Local Bg. Corr. Den.	% band purity	% lane purity	Mol. Wt.	Rel. Quant. (w/ LB Corr. Vol.)
1	1,714,086	1,388,548	408	0.138	4,201.191	3,403.306	54.23	14.956	148.077	0.666
2	1,651,668	1,171,918	408	0.486	4,048.206	2,872.35	45.77	14.412	39.537	0.562

Lane 7 - t = 0

#	Vol. (Int.)	Local Bg. Corr. Vol.	Area	Rf	Density	Local Bg. Corr. Den.	% band purity	% lane purity	Mol. Wt.	Rel. Quant. (w/ LB Corr. Vol.)
1	4,308,659	3,661,617	396	0.14	10,880	9,246.51	27.696	17.94	146.154	1.757
2	885,163	492,953	455	0.348	1,945.413	1,083.414	3.729	3.686	58.475	0.237
3	9,892,948	9,066,355	432	0.488	22,900	20,986	68.576	41.192	39.22	4.351

Lane 8 - Vehicle

#	Vol. (Int.)	Local Bg. Corr. Vol.	Area	Rf	Density	Local Bg. Corr. Den.	% band purity	% lane purity	Mol. Wt.	Rel. Quant. (w/ LB Corr. Vol.)

#	Vol. (Int.)	Local Bg. Corr. Vol.	Area	Rf	Density	Local Bg. Corr. Den.	% band purity	% lane purity	Mol. Wt.	Rel. Quant. (w/ LB Corr. Vol.)
1	2,652,121	2,083,655	396	0.14	6,697.275	5,261.758	31.301	15.779	146.154	1
2	778,116	329,203	462	0.345	1,684.234	712.562	4.945	4.629	58.898	0.158
3	5,061,419	4,243,987	429	0.488	11,798	9,892.746	63.754	30.113	39.22	2.037

Lane 9 - 5uM Ionomcyin

#	Vol. (Int.)	Local Bg. Corr. Vol.	Area	Rf	Density	Local Bg. Corr. Den.	% band purity	% lane purity	Mol. Wt.	Rel. Quant. (w/ LB Corr. Vol.)
1	1,484,604	989,723	384	0.143	3,866.156	2,577.406	10.448	6.81	144.231	0.475
2	1,218,499	581,305	510	0.345	2,389.214	1,139.814	6.137	5.589	58.898	0.279
3	9,115,198	7,901,785	462	0.486	19,729	17,103	83.415	41.811	39.537	3.792

Lane 10 - 2uM Thapsigargin

#	Vol. (Int.)	Local Bg. Corr. Vol.	Area	Rf	Density	Local Bg. Corr. Den.	% band purity	% lane purity	Mol. Wt.	Rel. Quant. (w/ LB Corr. Vol.)
1	3,022,682	2,537,945	384	0.145	7,871.568	6,609.232	22.422	13.375	142.308	1.218
2	1,324,384	522,534	840	0.351	1,576.648	622.065	4.617	5.86	58.051	0.251
3	9,248,206	8,258,299	403	0.486	22,948	20,492	72.961	40.924	39.537	3.963

iBright™ Image Analysis Report

Katarina+ Chang

18 November 2022

alpha tubulin CHEMI_10152021_130742

Date:	15 October 2021 01:07:42PM
Mode:	Chemi Blots
Notes:	
Model:	FL1500
Instrument name:	2462619090234
Serial No:	2462619090234
Firmware version:	1.6.0
iBA version:	5.0
Image size:	676px X 540px
Image area:	125.22mm X 100.18mm
Optical Zoom:	1.8x
Digital Zoom:	1x
Focus level:	405
Resolution:	5 x 5
Exposure time:	1800 ms
Exposure mode:	Normal

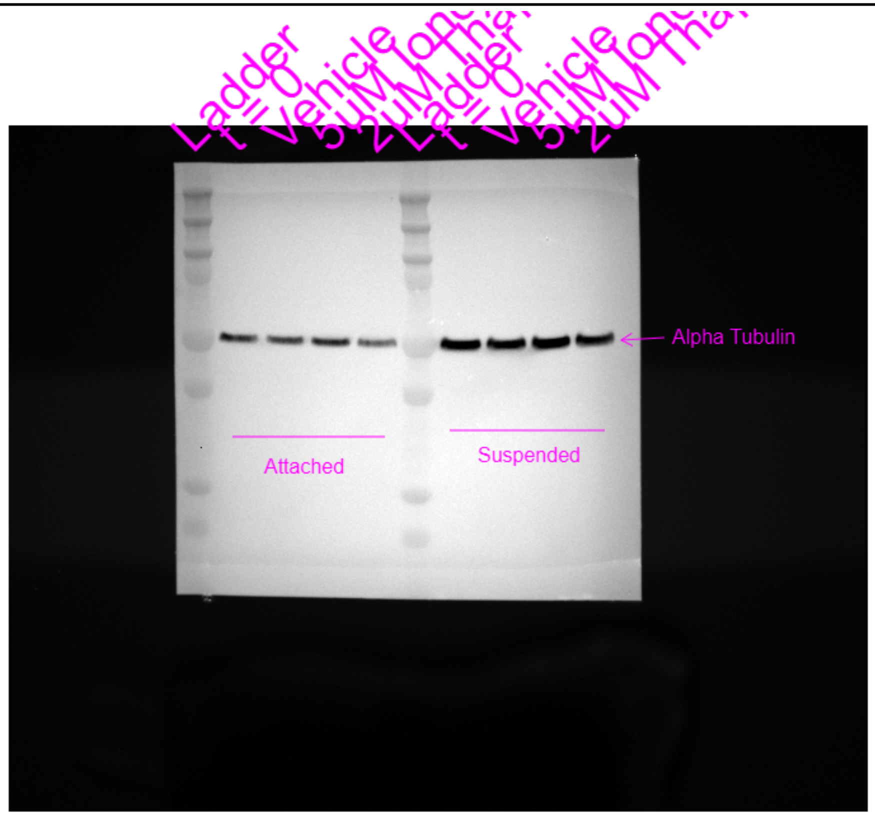

alpha tubulin CHEMI_10152021_130742

Date:	15 October 2021 01:07:42PM
Mode:	Chemi Blots
Notes:	
Model:	FL1500
Instrument name:	2462619090234
Serial No:	2462619090234
Firmware version:	1.6.0
iBA version:	5.0
Image size:	676px X 540px
Image area:	125.22mm X 100.18mm
Optical Zoom:	1.8x
Digital Zoom:	1x
Focus level:	405
Resolution:	5 x 5
Exposure time:	1800 ms
Exposure mode:	Normal

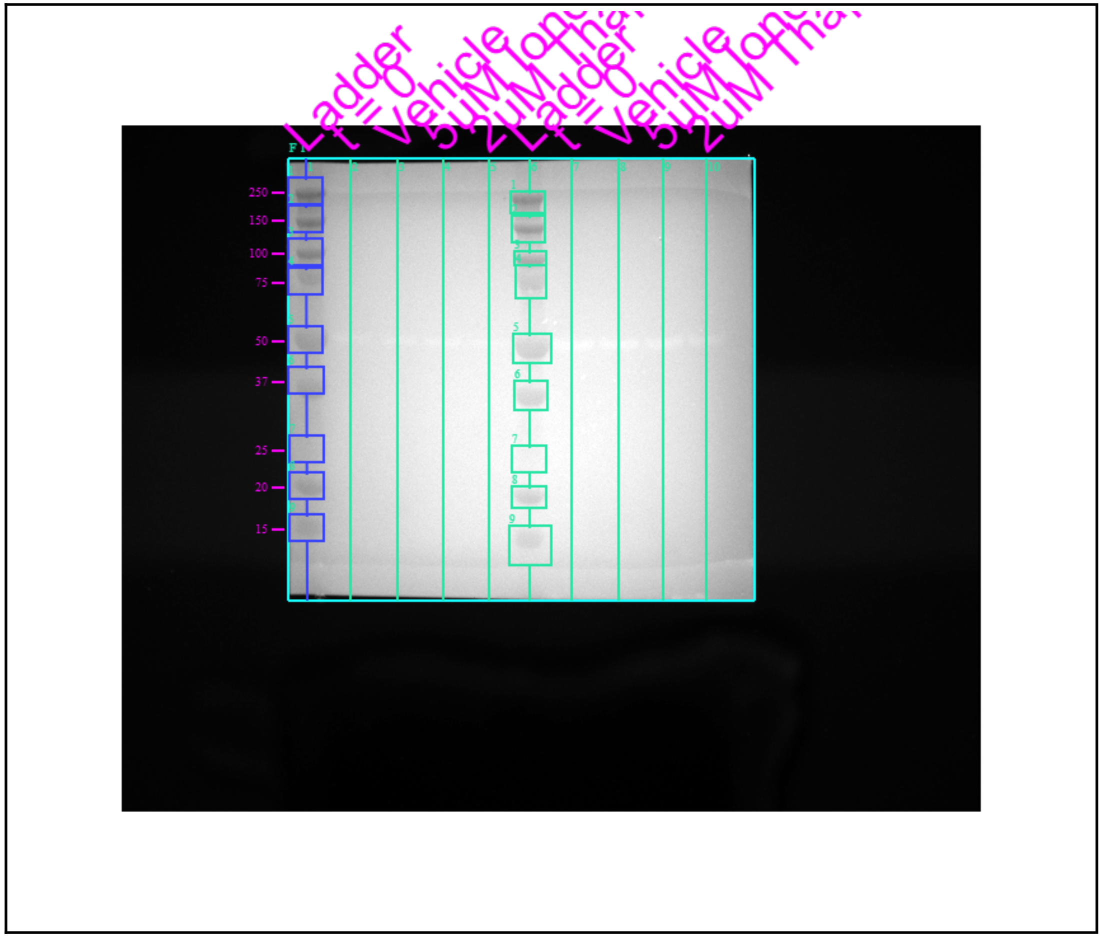

alpha tubulin CHEMI_10152021_130742

Date:	15 October 2021 01:07:42PM
Mode:	Chemi Blots
Notes:	
Model:	FL1500
Instrument name:	2462619090234
Serial No:	2462619090234
Firmware version:	1.6.0
iBA version:	5.0
Image size:	676px X 540px
Image area:	125.22mm X 100.18mm
Optical Zoom:	1.8x
Digital Zoom:	1x
Focus level:	405
Resolution:	5 x 5
Exposure time:	1800 ms
Exposure mode:	Normal

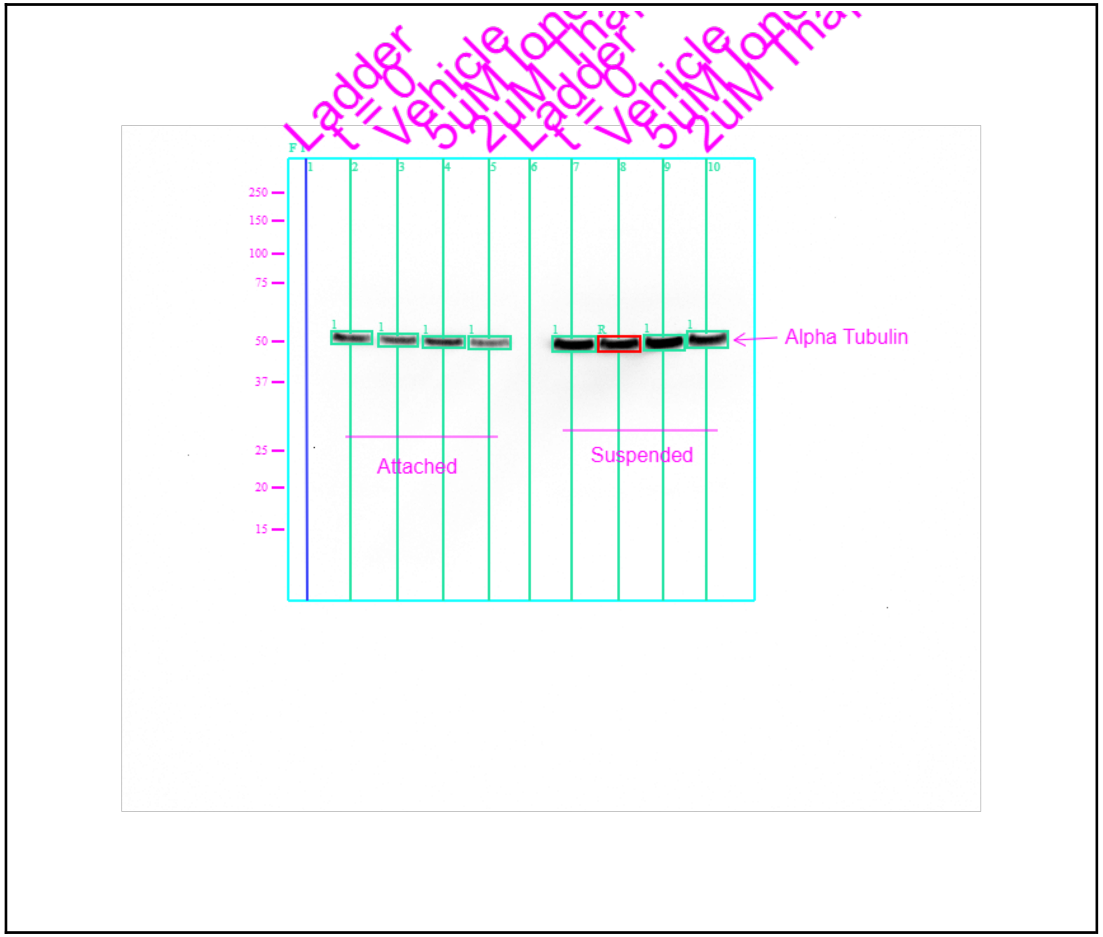

LANE AND BAND ANALYSIS DATA TABLE

alpha tubulin CHEMI_10152021_130742

Frame: 1
Channel: Membrane
Sensitivity: 100
Molecular Weight Analysis Regression Method : Point to Point

Lane 1 - Ladder

#	Vol. (Int.)	Local Bg. Corr. Vol.	Area	Rf	Density	Local Bg. Corr. Den.	% band purity	% lane purity	Mol. Wt.
1	24,462,039	113,920	616	0.072	39,711	184.935	68.697	6.629	250
2	23,654,236	32,605	616	0.135	38,399	52.931	19.662	6.41	150
3	22,222,529	12,521	616	0.21	36,075	20.328	7.551	6.022	100
4	21,169,888	NA	616	0.276	34,366	NA	NA	5.737	75
5	20,842,346	NA	616	0.408	33,834	NA	NA	5.648	50
6	20,865,601	233.93	638	0.5	32,704	0.367	0.141	5.655	37
7	19,395,269	90.32	616	0.655	31,485	0.147	0.054	5.256	25
8	20,553,522	1,921.094	616	0.739	33,366	3.119	1.158	5.57	20
9	20,679,375	4,537.734	616	0.833	33,570	7.366	2.736	5.604	15

Lane 6 - Ladder

#	Vol. (Int.)	Local Bg. Corr. Vol.	Area	Rf	Density	Local Bg. Corr. Den.	% band purity	% lane purity	Mol. Wt.
1	16,684,564	2,465,995	504	0.098	33,104	4,892.848	17.284	6.619	209.091
2	17,551,571	2,270,035	594	0.158	29,548	3,821.609	15.911	6.962	134.615
3	8,954,844	1,458,725	312	0.224	28,701	4,675.401	10.224	3.552	94.565
4	16,706,020	1,097,943	675	0.279	24,749	1,626.583	7.696	6.627	74.457
5	16,718,420	2,177,930	744	0.428	22,470	2,927.326	15.265	6.632	47.156
6	13,835,209	1,698,970	648	0.534	21,350	2,621.868	11.908	5.488	34.333
7	11,736,118	365,113	616	0.678	19,052	592.717	2.559	4.656	23.621
8	11,163,312	1,400,791	504	0.764	22,149	2,779.348	9.818	4.428	18.636
9	24,585,358	1,331,807	1,088	0.874	22,596	1,224.087	9.335	9.753	NA

Frame: 1
Channel: Chemi
Sensitivity: 100
Molecular Weight Analysis Regression Method : Point to Point

Lane 2 - t = 0

#	Vol. (Int.)	Local Bg. Corr. Vol.	Area	Rf	Density	Local Bg. Corr. Den.	% band purity	% lane purity	Mol. Wt.	Rel. Quant. (w/ LB Corr. Vol.)
1	4,686,734	4,342,246	363	0.405	12,911	11,962	100	69.95	50.543	0.642

Lane 3 - Vehicle

#	Vol. (Int.)	Local Bg. Corr. Vol.	Area	Rf	Density	Local Bg. Corr. Den.	% band purity	% lane purity	Mol. Wt.	Rel. Quant. (w/ LB Corr. Vol.)
1	4,205,660	3,676,899	352	0.411	11,947	10,445	100	60.658	49.594	0.543

Lane 4 - 5uM Ionomycin

#	Vol. (Int.)	Local Bg. Corr. Vol.	Area	Rf	Density	Local Bg. Corr. Den.	% band purity	% lane purity	Mol. Wt.	Rel. Quant. (w/ LB Corr. Vol.)
1	4,910,955	4,254,928	340	0.414	14,443	12,514	100	62.544	49.187	0.629

Lane 5 - 2uM Thapsigargin

#	Vol. (Int.)	Local Bg. Corr. Vol.	Area	Rf	Density	Local Bg. Corr. Den.	% band purity	% lane purity	Mol. Wt.	Rel. Quant. (w/ LB Corr. Vol.)
1	3,616,888	3,156,394	374	0.417	9,670.824	8,439.557	100	56.624	48.781	0.466

Lane 7 - t = 0

#	Vol. (Int.)	Local Bg. Corr. Vol.	Area	Rf	Density	Local Bg. Corr. Den.	% band purity	% lane purity	Mol. Wt.	Rel. Quant. (w/ LB Corr. Vol.)
1	9,676,789	8,107,711	455	0.42	21,267	17,819	100	74.78	48.375	1.198

Lane 8 - Vehicle

#	Vol. (Int.)	Local Bg. Corr. Vol.	Area	Rf	Density	Local Bg. Corr. Den.	% band purity	% lane purity	Mol. Wt.	Rel. Quant. (w/ LB Corr. Vol.)
1	8,810,825	6,768,507	442	0.42	19,933	15,313	100	68.466	48.375	1

Lane 9 - 5uM Ionomycin

#	Vol. (Int.)	Local Bg. Corr. Vol.	Area	Rf	Density	Local Bg. Corr. Den.	% band purity	% lane purity	Mol. Wt.	Rel. Quant. (w/ LB Corr. Vol.)
1	10,224,076	8,525,005	429	0.417	23,832	19,871	100	76.166	48.781	1.26

Lane 10 - 2uM Thapsigargin

#	Vol. (Int.)	Local Bg. Corr. Vol.	Area	Rf	Density	Local Bg. Corr. Den.	% band purity	% lane purity	Mol. Wt.	Rel. Quant. (w/ LB Corr. Vol.)
1	7,465,784	6,678,586	462	0.408	16,159	14,455	100	78.27	50	0.987

iBright™ Image Analysis Report

Katarina+ Chang

18 November 2022

GAPDH_CHEMI_10162021_142805

Date:	16 October 2021 02:28:05PM
Mode:	Chemi Blots
Notes:	
Model:	FL1500
Instrument name:	2462619090234
Serial No:	2462619090234
Firmware version:	1.6.0
iBA version:	5.0
Image size:	676px X 540px
Image area:	112.7mm X 90.16mm
Optical Zoom:	2x
Digital Zoom:	1x
Focus level:	455
Resolution:	5 x 5
Exposure time:	25722 ms
Exposure mode:	Normal

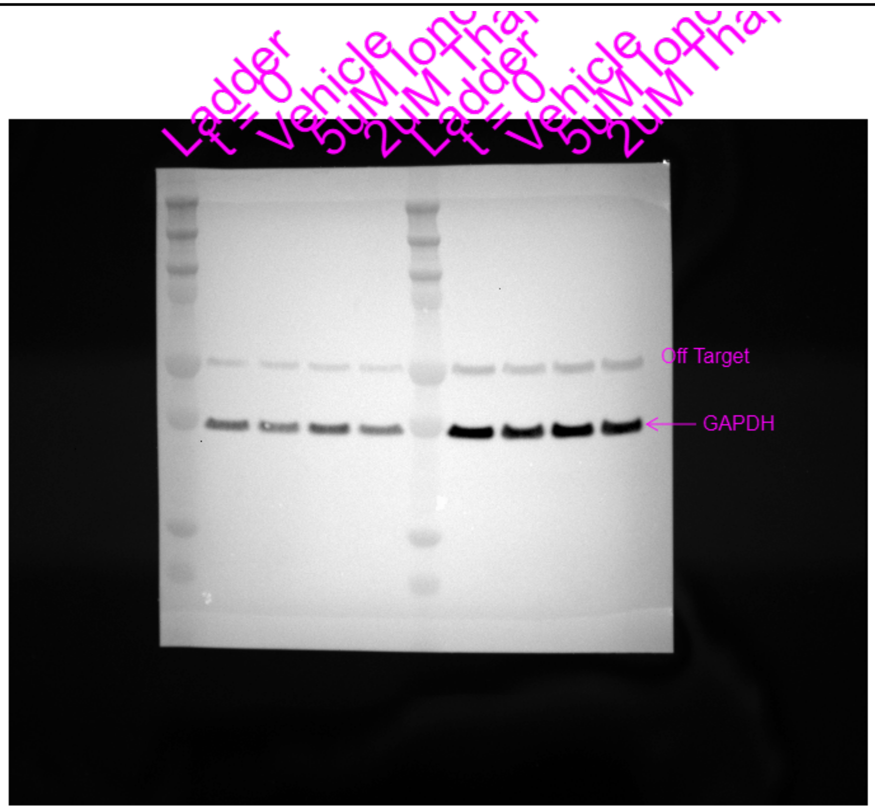

GAPDH_CHEMI_10162021_142805

Date:	16 October 2021 02:28:05PM
Mode:	Chemi Blots
Notes:	
Model:	FL1500
Instrument name:	2462619090234
Serial No:	2462619090234
Firmware version:	1.6.0
iBA version:	5.0
Image size:	676px X 540px
Image area:	112.7mm X 90.16mm
Optical Zoom:	2x
Digital Zoom:	1x
Focus level:	455
Resolution:	5 x 5
Exposure time:	25722 ms
Exposure mode:	Normal

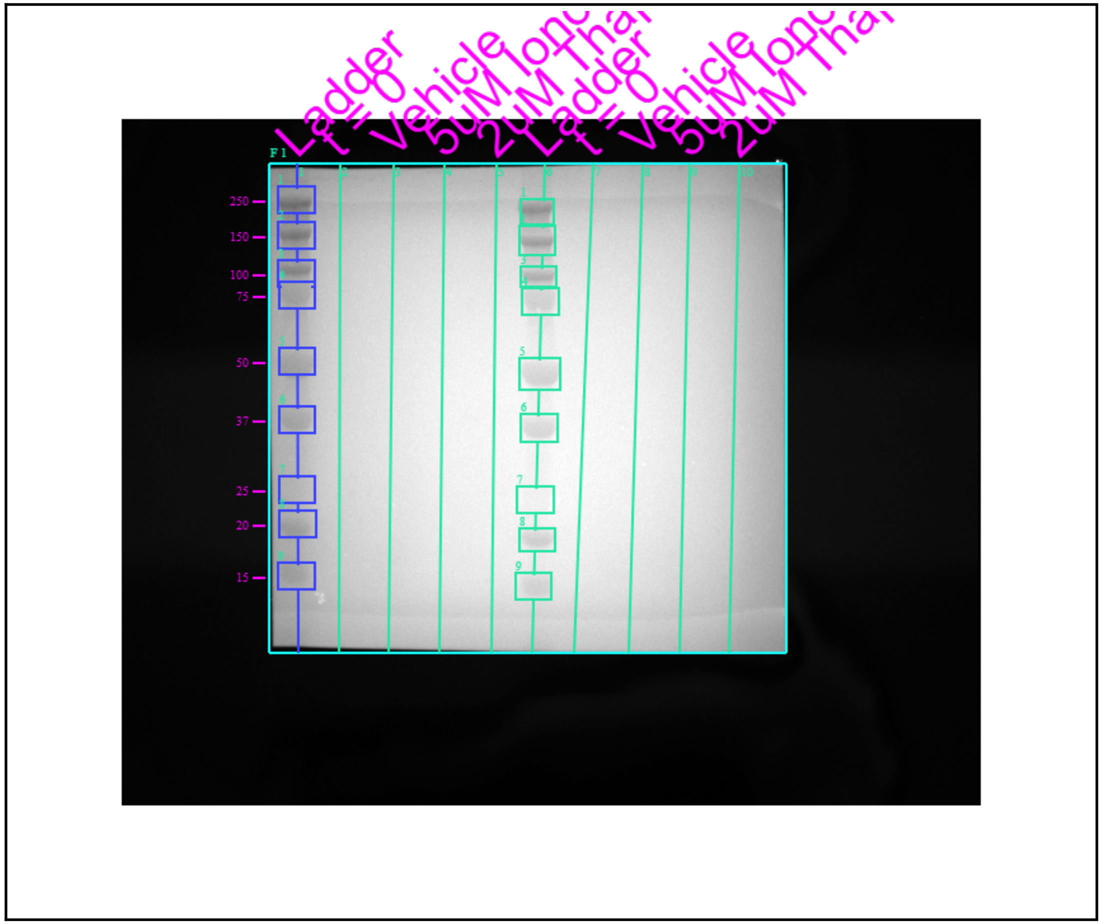

GAPDH_CHEMI_10162021_142805

Date:	16 October 2021 02:28:05PM
Mode:	Chemi Blots
Notes:	
Model:	FL1500
Instrument name:	2462619090234
Serial No:	2462619090234
Firmware version:	1.6.0
iBA version:	5.0
Image size:	676px X 540px
Image area:	112.7mm X 90.16mm
Optical Zoom:	2x
Digital Zoom:	1x
Focus level:	455
Resolution:	5 x 5
Exposure time:	25722 ms
Exposure mode:	Normal

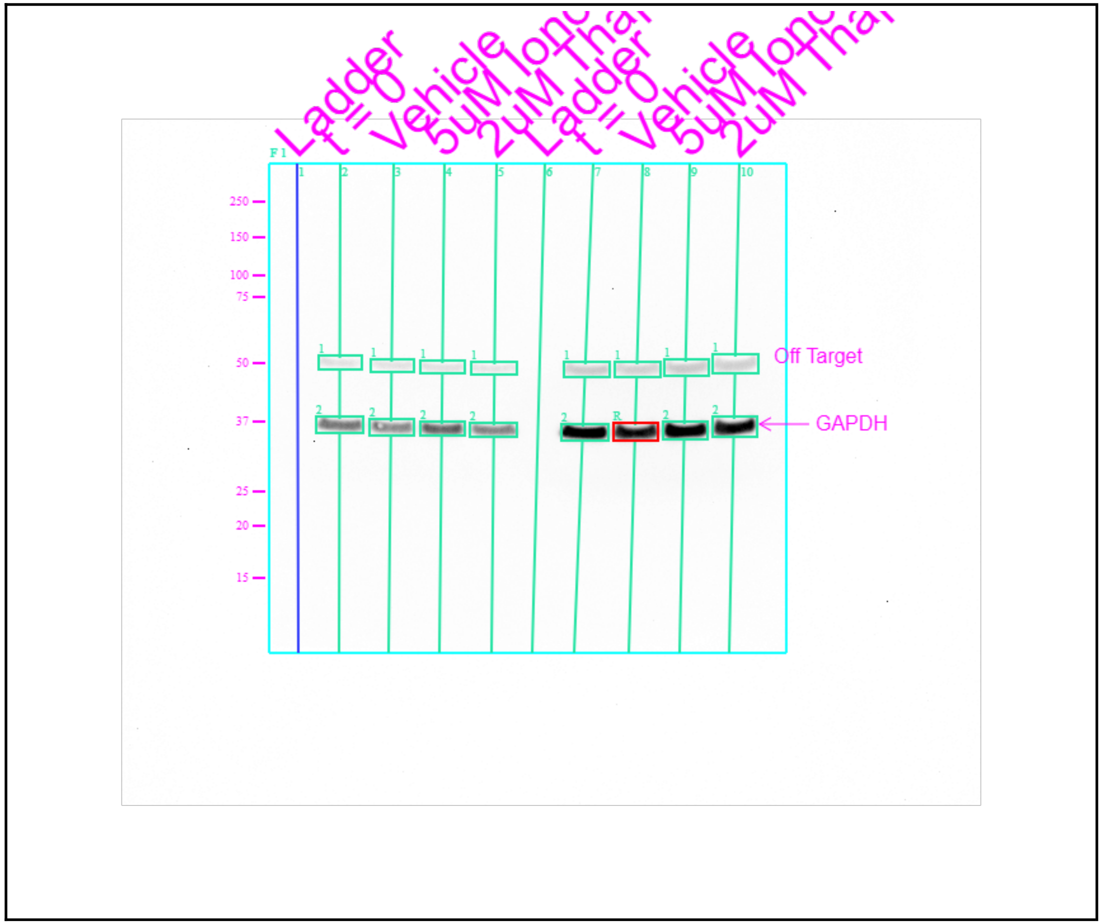

LANE AND BAND ANALYSIS DATA TABLE

GAPDH_CHEMI_10162021_142805

Frame: 1
Channel: Membrane
Sensitivity: 100
Molecular Weight Analysis Regression Method : Point to Point

Lane 1 - Ladder

#	Vol. (Int.)	Local Bg. Corr. Vol.	Area	Rf	Density	Local Bg. Corr. Den.	% band purity	% lane purity	Mol. Wt.
1	25,812,879	2,028,517	660	0.073	39,110	3,073.512	15.666	5.929	250
2	24,705,866	2,164,537	660	0.145	37,433	3,279.602	16.716	5.675	150
3	23,396,872	1,932,525	660	0.223	35,449	2,928.07	14.924	5.374	100
4	21,416,737	1,221,706	638	0.268	33,568	1,914.9	9.435	4.92	75
5	21,231,769	1,625,070	638	0.403	33,278	2,547.133	12.55	4.877	50
6	20,689,300	1,515,569	638	0.522	32,428	2,375.501	11.704	4.752	37
7	19,874,606	513,920	638	0.665	31,151	805.517	3.969	4.565	25
8	21,920,458	1,317,936	660	0.735	33,212	1,996.874	10.178	5.035	20
9	22,400,484	629,054	660	0.842	33,940	953.113	4.858	5.146	15

Lane 6 - Ladder

#	Vol. (Int.)	Local Bg. Corr. Vol.	Area	Rf	Density	Local Bg. Corr. Den.	% band purity	% lane purity	Mol. Wt.
1	18,897,817	2,800,610	567	0.099	33,329	4,939.349	17.199	6.069	214.286
2	20,696,557	2,654,236	696	0.156	29,736	3,813.558	16.3	6.646	143.333
3	13,679,766	2,029,969	493	0.231	27,748	4,117.584	12.466	4.393	95.588
4	15,968,789	1,279,079	660	0.281	24,195	1,938	7.855	5.128	72.596
5	19,540,822	2,537,624	858	0.429	22,774	2,957.605	15.584	6.275	47.174
6	15,288,586	1,947,816	690	0.54	22,157	2,822.922	11.962	4.91	35.473
7	12,976,301	425,282	660	0.686	19,661	644.367	2.612	4.167	23.519
8	12,900,825	1,558,918	551	0.769	23,413	2,829.253	9.573	4.143	18.415
9	15,496,222	1,050,231	638	0.862	24,288	1,646.131	6.45	4.976	NA

Frame: 1
Channel: Chemi
Sensitivity: 100
Molecular Weight Analysis Regression Method : Point to Point

Lane 2 - t = 0

#	Vol. (Int.)	Local Bg. Corr. Vol.	Area	Rf	Density	Local Bg. Corr. Den.	% band purity	% lane purity	Mol. Wt.	Rel. Quant. (w/ LB Corr. Vol.)
1	990,138	732,869	420	0.405	2,357.471	1,744.928	12.096	8.537	49.717	0.074
2	5,801,243	5,325,703	532	0.532	10,904	10,010	87.904	50.016	36.127	0.538

Lane 3 - Vehicle

#	Vol. (Int.)	Local Bg. Corr. Vol.	Area	Rf	Density	Local Bg. Corr. Den.	% band purity	% lane purity	Mol. Wt.	Rel. Quant. (w/ LB Corr. Vol.)
1	949,339	669,257	385	0.413	2,465.816	1,738.331	14.083	8.569	48.87	0.068
2	4,707,090	4,083,089	504	0.538	9,339.464	8,101.367	85.917	42.488	35.691	0.412

Lane 4 - 5uM Ionomycin

#	Vol. (Int.)	Local Bg. Corr. Vol.	Area	Rf	Density	Local Bg. Corr. Den.	% band purity	% lane purity	Mol. Wt.	Rel. Quant. (w/ LB Corr. Vol.)
1	1,059,530	766,602	396	0.416	2,675.581	1,935.866	12.222	8.238	48.587	0.077
2	6,144,474	5,505,471	432	0.54	14,223	12,744	87.778	47.775	35.473	0.556

Lane 5 - 2uM Thapsigargin

#	Vol. (Int.)	Local Bg. Corr. Vol.	Area	Rf	Density	Local Bg. Corr. Den.	% band purity	% lane purity	Mol. Wt.	Rel. Quant. (w/ LB Corr. Vol.)
1	939,280	652,292	407	0.418	2,307.813	1,602.685	12.998	8.245	48.304	0.066
2	4,914,934	4,366,133	456	0.543	10,778	9,574.853	87.002	43.142	35.255	0.441

Lane 7 - t = 0

#	Vol. (Int.)	Local Bg. Corr. Vol.	Area	Rf	Density	Local Bg. Corr. Den.	% band purity	% lane purity	Mol. Wt.	Rel. Quant. (w/ LB Corr. Vol.)
1	2,269,910	1,724,484	481	0.421	4,719.148	3,585.207	11.886	10.103	48.022	0.174
2	14,159,100	12,784,357	532	0.548	26,614	24,030	88.114	63.021	34.818	1.291

Lane 8 - Vehicle

#	Vol. (Int.)	Local Bg. Corr. Vol.	Area	Rf	Density	Local Bg. Corr. Den.	% band purity	% lane purity	Mol. Wt.	Rel. Quant. (w/ LB Corr. Vol.)
1	2,050,614	1,415,626	481	0.421	4,263.231	2,943.091	12.511	10.059	48.022	0.143

#	Vol. (Int.)	Local Bg. Corr. Vol.	Area	Rf	Density	Local Bg. Corr. Den.	% band purity	% lane purity	Mol. Wt.	Rel. Quant. (w/ LB Corr. Vol.)
2	11,371,900	9,899,754	540	0.548	21,059	18,332	87.489	55.782	34.818	1

Lane 9 - 5uM Ionomycin

#	Vol. (Int.)	Local Bg. Corr. Vol.	Area	Rf	Density	Local Bg. Corr. Den.	% band purity	% lane purity	Mol. Wt.	Rel. Quant. (w/ LB Corr. Vol.)
1	2,376,961	1,735,100	504	0.416	4,716.192	3,442.66	12.313	10.307	48.587	0.175
2	13,806,830	12,356,833	540	0.545	25,568	22,883	87.687	59.867	35.036	1.248

Lane 10 - 2uM Thapsigargin

#	Vol. (Int.)	Local Bg. Corr. Vol.	Area	Rf	Density	Local Bg. Corr. Den.	% band purity	% lane purity	Mol. Wt.	Rel. Quant. (w/ LB Corr. Vol.)
1	2,251,825	1,706,329	592	0.408	3,803.758	2,882.314	13.316	11.041	49.435	0.172
2	12,092,735	11,107,365	612	0.538	19,759	18,149	86.684	59.291	35.691	1.122

iBright™ Image Analysis Report

Katarina+ Chang

18 November 2022

total MLC_CHEMI_10132021_123220

Date:	13 October 2021 12:32:20PM
Mode:	Chemi Blots
Notes:	
Model:	FL1500
Instrument name:	2462619090234
Serial No:	2462619090234
Firmware version:	1.6.0
iBA version:	5.0
Image size:	615px X 491px
Image area:	112.7mm X 90.16mm
Optical Zoom:	2x
Digital Zoom:	1.1x
Focus level:	455
Resolution:	5 x 5
Exposure time:	4798 ms
Exposure mode:	Normal

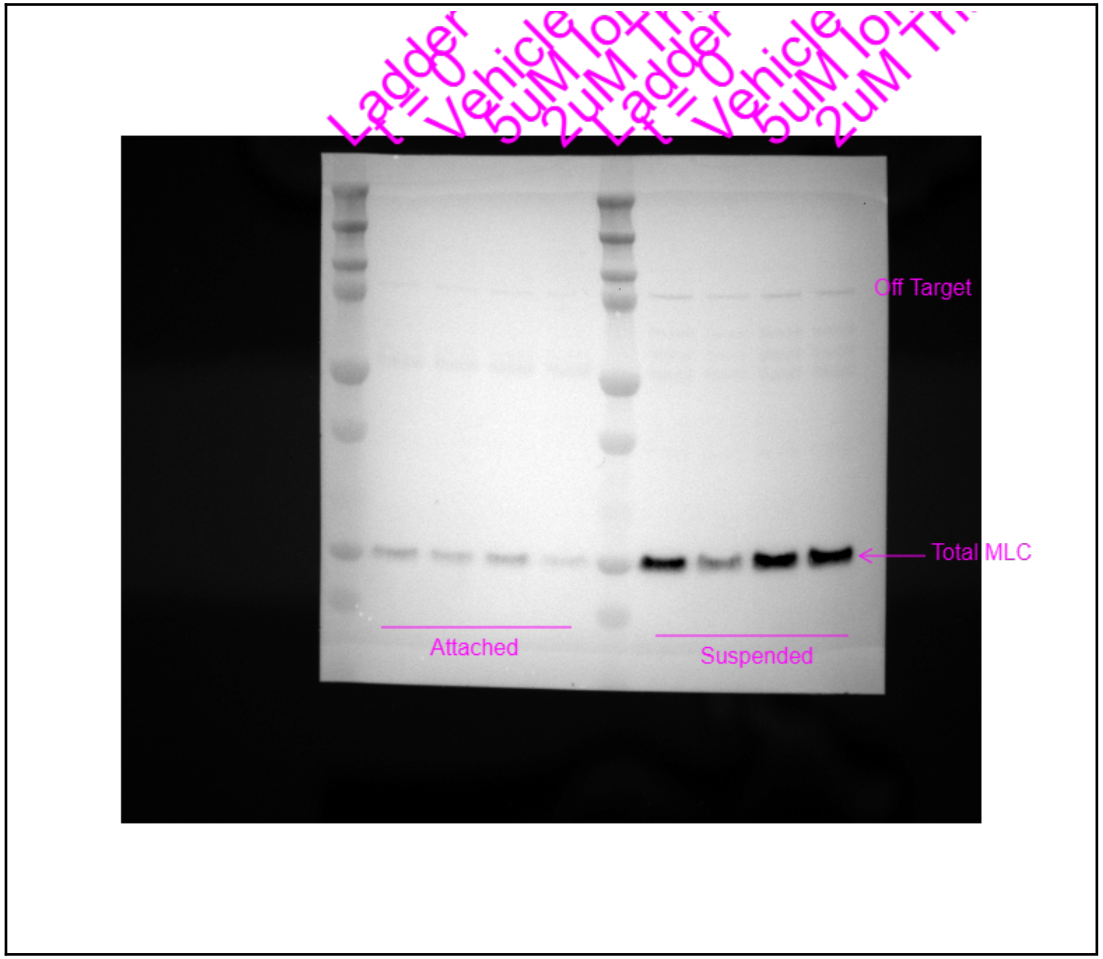

total MLC_CHEMI_10132021_123220

Date:	13 October 2021 12:32:20PM
Mode:	Chemi Blots
Notes:	
Model:	FL1500
Instrument name:	2462619090234
Serial No:	2462619090234
Firmware version:	1.6.0
iBA version:	5.0
Image size:	615px X 491px
Image area:	112.7mm X 90.16mm
Optical Zoom:	2x
Digital Zoom:	1.1x
Focus level:	455
Resolution:	5 x 5
Exposure time:	4798 ms
Exposure mode:	Normal

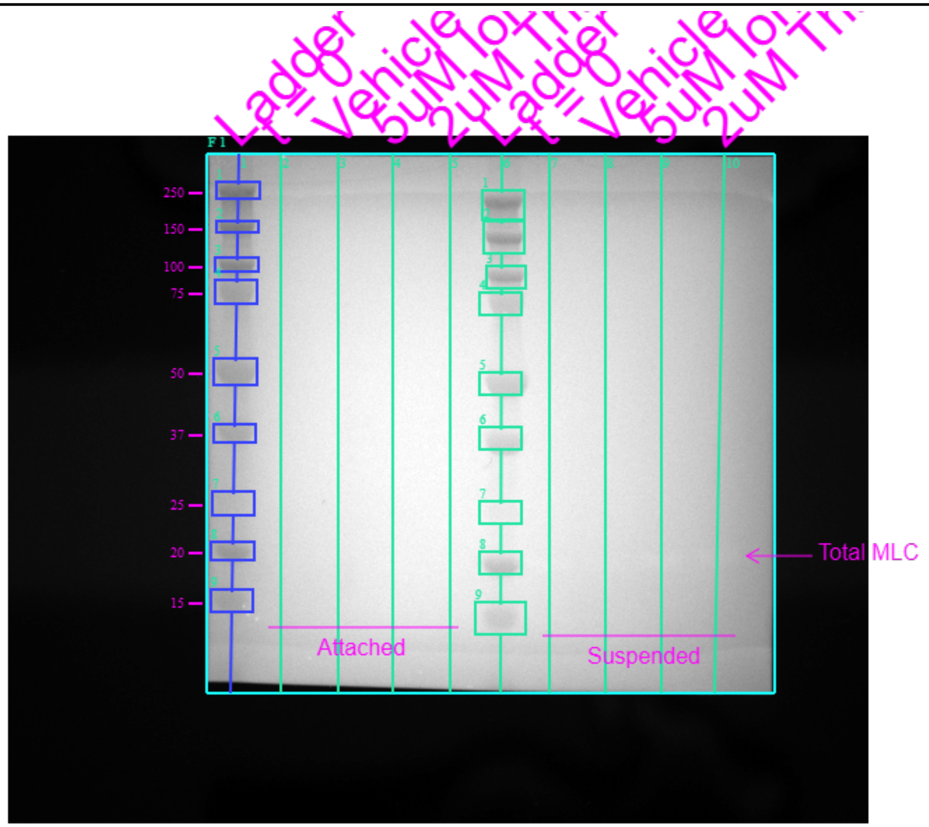

total MLC_CHEMI_10132021_123220

Date:	13 October 2021 12:32:20PM
Mode:	Chemi Blots
Notes:	
Model:	FL1500
Instrument name:	2462619090234
Serial No:	2462619090234
Firmware version:	1.6.0
iBA version:	5.0
Image size:	615px X 491px
Image area:	112.7mm X 90.16mm
Optical Zoom:	2x
Digital Zoom:	1.1x
Focus level:	455
Resolution:	5 x 5
Exposure time:	4798 ms
Exposure mode:	Normal

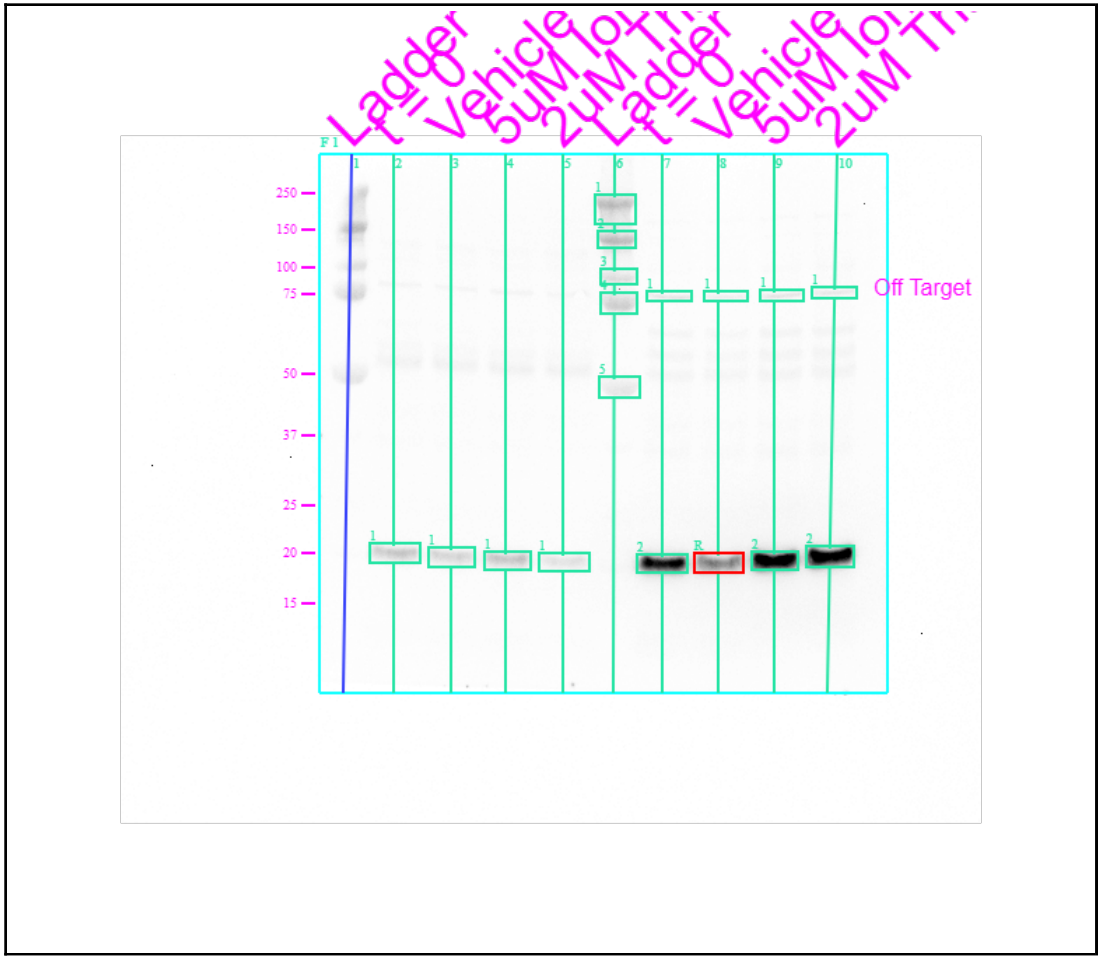

LANE AND BAND ANALYSIS DATA TABLE

total MLC_CHEMI_10132021_123220

Frame: 1
Channel: Membrane
Sensitivity: 100
Molecular Weight Analysis Regression Method : Point to Point

Lane 1 - Ladder

#	Vol. (Int.)	Local Bg. Corr. Vol.	Area	Rf	Density	Local Bg. Corr. Den.	% band purity	% lane purity	Mol. Wt.
1	16,382,779	1,722,622	416	0.068	39,381	4,140.92	20.423	4.082	250
2	10,705,340	1,371,596	279	0.135	38,370	4,916.117	16.261	2.667	150
3	12,691,864	1,463,602	352	0.205	36,056	4,157.962	17.352	3.162	100
4	18,516,926	1,203,042	558	0.255	33,184	2,155.991	14.263	4.614	75
5	20,951,358	1,522,063	640	0.403	32,736	2,378.224	18.045	5.22	50
6	13,881,902	798,071	434	0.517	31,985	1,838.875	9.462	3.459	37
7	16,930,286	11,063	558	0.647	30,341	19.827	0.131	4.218	25
8	15,168,472	303,981	448	0.735	33,858	678.531	3.604	3.779	20
9	17,598,451	38,635	527	0.829	33,393	73.312	0.458	4.385	15

Lane 6 - Ladder

#	Vol. (Int.)	Local Bg. Corr. Vol.	Area	Rf	Density	Local Bg. Corr. Den.	% band purity	% lane purity	Mol. Wt.
1	22,630,392	3,033,501	682	0.094	33,182	4,447.95	19.189	7.389	211.538
2	21,817,948	2,665,716	720	0.153	30,302	3,702.385	16.862	7.124	137.037
3	14,113,215	2,007,160	493	0.229	28,627	4,071.32	12.696	4.608	88.158
4	13,414,734	1,316,502	527	0.278	25,454	2,498.108	8.328	4.38	71.053
5	13,052,131	1,792,183	527	0.426	24,766	3,400.728	11.337	4.262	47.341
6	12,273,081	1,379,943	527	0.527	23,288	2,618.489	8.729	4.007	36.04
7	11,068,474	418,527	527	0.665	21,002	794.169	2.647	3.614	23.971
8	13,171,197	1,894,539	527	0.758	24,992	3,594.952	11.984	4.301	18.75
9	22,147,048	1,300,834	888	0.86	24,940	1,464.904	8.228	7.231	NA

Frame: 1
Channel: Chemi
Sensitivity: 100
Molecular Weight Analysis Regression Method : Point to Point

Lane 2 - t = 0

#	Vol. (Int.)	Local Bg. Corr. Vol.	Area	Rf	Density	Local Bg. Corr. Den.	% band purity	% lane purity	Mol. Wt.	Rel. Quant. (w/ LB Corr. Vol.)
1	2,212,266	1,739,494	555	0.74	3,986.065	3,134.225	100	25.422	19.722	0.445

Lane 3 - Vehicle

#	Vol. (Int.)	Local Bg. Corr. Vol.	Area	Rf	Density	Local Bg. Corr. Den.	% band purity	% lane purity	Mol. Wt.	Rel. Quant. (w/ LB Corr. Vol.)
1	1,683,830	1,190,048	510	0.748	3,301.627	2,333.429	100	19.565	19.306	0.304

Lane 4 - 5uM Ionomycin

#	Vol. (Int.)	Local Bg. Corr. Vol.	Area	Rf	Density	Local Bg. Corr. Den.	% band purity	% lane purity	Mol. Wt.	Rel. Quant. (w/ LB Corr. Vol.)
1	2,168,561	1,643,157	476	0.753	4,555.8	3,452.012	100	23.131	19.028	0.42

Lane 5 - 2uM Thapsigargin

#	Vol. (Int.)	Local Bg. Corr. Vol.	Area	Rf	Density	Local Bg. Corr. Den.	% band purity	% lane purity	Mol. Wt.	Rel. Quant. (w/ LB Corr. Vol.)
1	1,234,767	788,057	518	0.756	2,383.72	1,521.347	100	14.413	18.889	0.201

Lane 6 - Ladder

#	Vol. (Int.)	Local Bg. Corr. Vol.	Area	Rf	Density	Local Bg. Corr. Den.	% band purity	% lane purity	Mol. Wt.	Rel. Quant. (w/ LB Corr. Vol.)
1	3,175,587	2,288,604	660	0.101	4,811.495	3,467.582	37.421	19.372	200	0.585
2	2,028,875	1,388,366	364	0.158	5,573.832	3,814.194	22.701	12.377	133.333	0.355
3	901,505	540,920	324	0.226	2,782.423	1,669.507	8.845	5.499	89.474	0.138
4	1,673,947	1,257,759	432	0.275	3,874.877	2,911.48	20.566	10.212	71.491	0.322
5	971,325	640,163	480	0.431	2,023.594	1,333.674	10.467	5.925	46.75	0.164

Lane 7 - t = 0

#	Vol. (Int.)	Local Bg. Corr. Vol.	Area	Rf	Density	Local Bg. Corr. Den.	% band purity	% lane purity	Mol. Wt.	Rel. Quant. (w/ LB Corr. Vol.)
1	481,161	313,089	264	0.262	1,822.58	1,185.945	3.378	2.44	73.684	0.08
2	10,631,338	8,955,736	518	0.758	20,523	17,289	96.622	53.923	18.75	2.29

Lane 8 - Vehicle

#	Vol. (Int.)	Local Bg. Corr. Vol.	Area	Rf	Density	Local Bg. Corr. Den.	% band purity	% lane purity	Mol. Wt.	Rel. Quant. (w/ LB Corr. Vol.)
1	333,857	192,619	256	0.262	1,304.129	752.42	4.694	2.367	73.684	0.049
2	5,603,739	3,911,148	540	0.758	10,377	7,242.868	95.306	39.732	18.75	1

Lane 9 - 5uM Ionomycin

#	Vol. (Int.)	Local Bg. Corr. Vol.	Area	Rf	Density	Local Bg. Corr. Den.	% band purity	% lane purity	Mol. Wt.	Rel. Quant. (w/ LB Corr. Vol.)
1	483,932	298,747	288	0.262	1,680.319	1,037.318	2.607	2.006	73.684	0.076
2	13,389,439	11,161,382	476	0.753	28,129	23,448	97.393	55.51	19.028	2.854

Lane 10 - 2uM Thapsigargin

#	Vol. (Int.)	Local Bg. Corr. Vol.	Area	Rf	Density	Local Bg. Corr. Den.	% band purity	% lane purity	Mol. Wt.	Rel. Quant. (w/ LB Corr. Vol.)
1	476,720	288,979	297	0.257	1,605.118	972.995	2.377	2.035	74.561	0.074
2	13,670,505	11,870,380	560	0.745	24,411	21,197	97.623	58.366	19.444	3.035

iBright™ Image Analysis Report

Katarina+ Chang

18 November 2022

total MYPT1 CHEMI_10142021_145939

Date:	14 October 2021 02:59:39PM
Mode:	Chemi Blots
Notes:	
Model:	FL1500
Instrument name:	2462619090234
Serial No:	2462619090234
Firmware version:	1.6.0
iBA version:	5.0
Image size:	615px X 491px
Image area:	112.7mm X 90.16mm
Optical Zoom:	2x
Digital Zoom:	1.1x
Focus level:	455
Resolution:	5 x 5
Exposure time:	859 ms
Exposure mode:	Normal

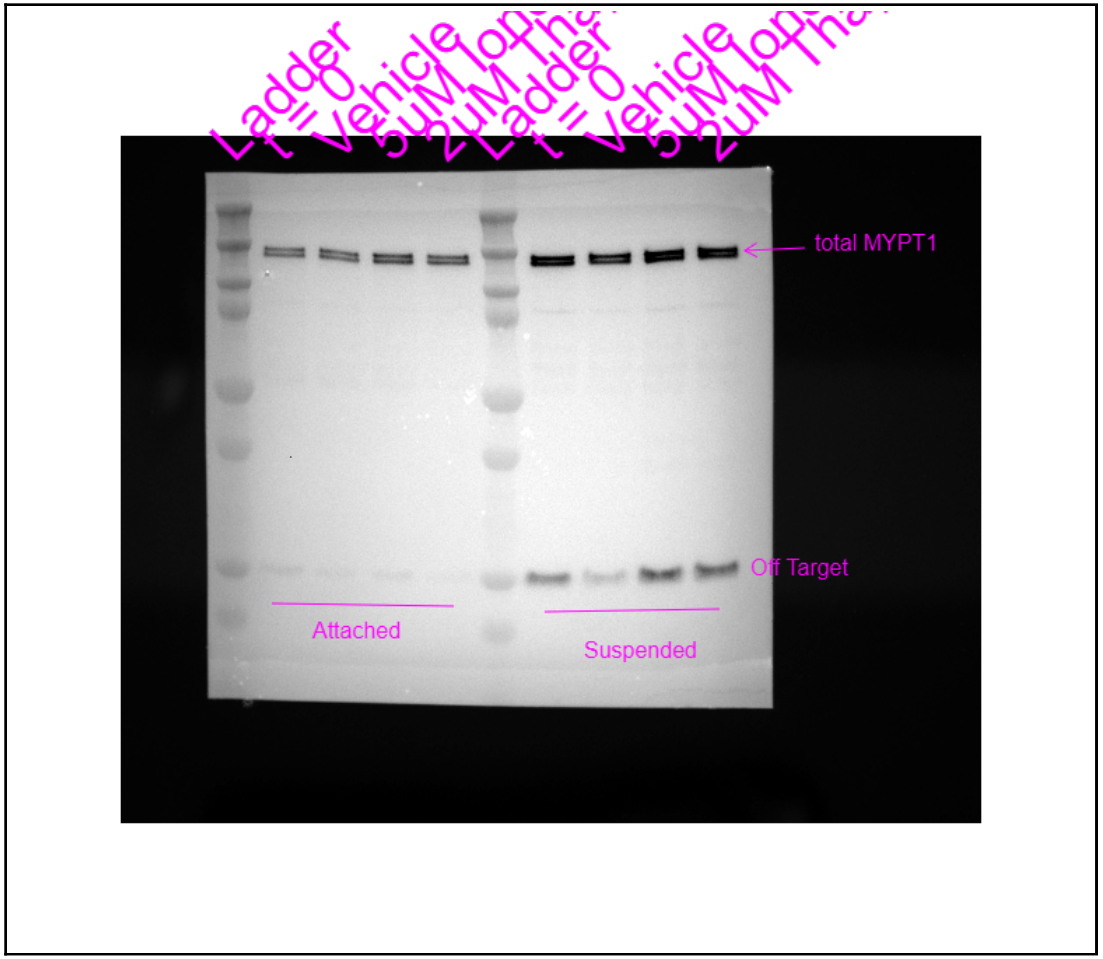

total MYPT1 CHEMI_10142021_145939

Date:	14 October 2021 02:59:39PM
Mode:	Chemi Blots
Notes:	
Model:	FL1500
Instrument name:	2462619090234
Serial No:	2462619090234
Firmware version:	1.6.0
iBA version:	5.0
Image size:	615px X 491px
Image area:	112.7mm X 90.16mm
Optical Zoom:	2x
Digital Zoom:	1.1x
Focus level:	455
Resolution:	5 x 5
Exposure time:	859 ms
Exposure mode:	Normal

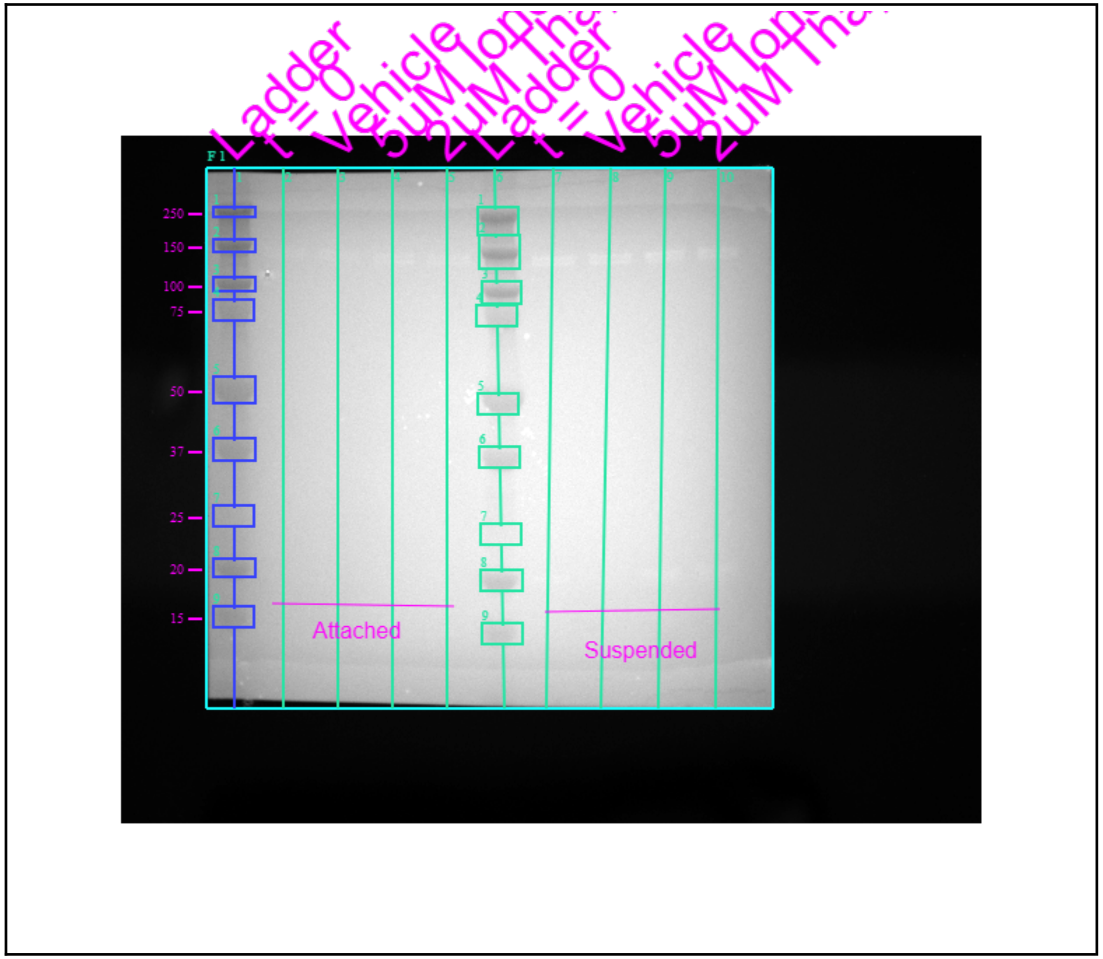

total MYPT1 CHEMI_10142021_145939

Date:	14 October 2021 02:59:39PM
Mode:	Chemi Blots
Notes:	
Model:	FL1500
Instrument name:	2462619090234
Serial No:	2462619090234
Firmware version:	1.6.0
iBA version:	5.0
Image size:	615px X 491px
Image area:	112.7mm X 90.16mm
Optical Zoom:	2x
Digital Zoom:	1.1x
Focus level:	455
Resolution:	5 x 5
Exposure time:	859 ms
Exposure mode:	Normal

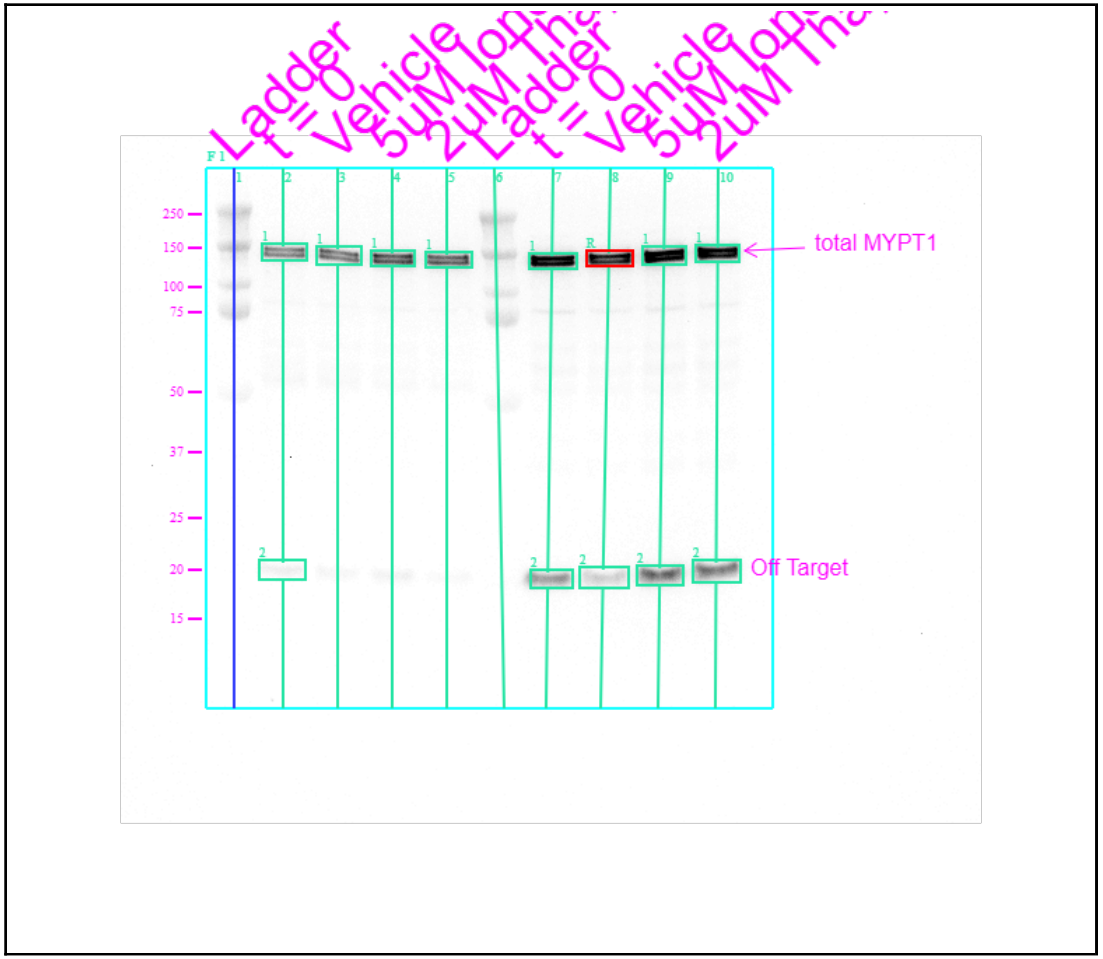

LANE AND BAND ANALYSIS DATA TABLE

total MYPT1 CHEMI_10142021_145939

Frame: 1
Channel: Membrane
Sensitivity: 100
Molecular Weight Analysis Regression Method : Point to Point

Lane 1 - Ladder

#	Vol. (Int.)	Local Bg. Corr. Vol.	Area	Rf	Density	Local Bg. Corr. Den.	% band purity	% lane purity	Mol. Wt.
1	10,378,291	1,043,803	248	0.08	41,847	4,208.887	12.243	2.432	250
2	12,286,943	1,266,344	310	0.142	39,635	4,084.982	14.853	2.879	150
3	12,836,198	1,289,242	341	0.215	37,642	3,780.769	15.122	3.008	100
4	16,859,514	906,510	480	0.262	35,123	1,888.563	10.633	3.951	75
5	21,469,955	1,688,584	620	0.409	34,628	2,723.524	19.806	5.031	50
6	17,854,093	1,136,137	527	0.521	33,878	2,155.858	13.326	4.184	37
7	15,599,672	167,828	480	0.642	32,499	349.642	1.969	3.656	25
8	15,321,519	666,363	434	0.738	35,303	1,535.401	7.816	3.591	20
9	16,984,889	360,787	480	0.829	35,385	751.641	4.232	3.98	15

Lane 6 - Ladder

#	Vol. (Int.)	Local Bg. Corr. Vol.	Area	Rf	Density	Local Bg. Corr. Den.	% band purity	% lane purity	Mol. Wt.
1	20,386,143	2,935,991	630	0.098	32,358	4,660.303	20.951	6.849	220.833
2	21,872,324	2,637,383	750	0.155	29,163	3,516.512	18.82	7.348	141.071
3	13,492,172	1,934,319	493	0.231	27,367	3,923.569	13.803	4.533	91.667
4	11,809,125	942,933	480	0.272	24,602	1,964.445	6.729	3.967	73.246
5	11,294,992	1,507,899	480	0.435	23,531	3,141.457	10.76	3.795	46.977
6	11,032,627	1,501,283	480	0.534	22,984	3,127.675	10.713	3.707	35.723
7	9,775,942	239,562	480	0.676	20,366	499.089	1.709	3.284	23.243
8	11,894,421	1,462,316	496	0.762	23,980	2,948.219	10.435	3.996	18.714
9	12,050,356	852,124	480	0.86	25,104	1,775.26	6.081	4.049	NA

Frame: 1
Channel: Chemi
Sensitivity: 100
Molecular Weight Analysis Regression Method : Point to Point

Lane 2 - t = 0

#	Vol. (Int.)	Local Bg. Corr. Vol.	Area	Rf	Density	Local Bg. Corr. Den.	% band purity	% lane purity	Mol. Wt.	Rel. Quant. (w/ LB Corr. Vol.)
1	2,088,480	1,788,788	429	0.155	4,868.252	4,169.67	87.701	48.238	141.071	0.525
2	295,017	250,852	510	0.744	578.465	491.867	12.299	6.814	19.714	0.074

Lane 3 - Vehicle

#	Vol. (Int.)	Local Bg. Corr. Vol.	Area	Rf	Density	Local Bg. Corr. Den.	% band purity	% lane purity	Mol. Wt.	Rel. Quant. (w/ LB Corr. Vol.)
1	2,314,976	1,975,476	462	0.161	5,010.771	4,275.923	100	53.191	137.5	0.58

Lane 4 - 5uM Ionomycin

#	Vol. (Int.)	Local Bg. Corr. Vol.	Area	Rf	Density	Local Bg. Corr. Den.	% band purity	% lane purity	Mol. Wt.	Rel. Quant. (w/ LB Corr. Vol.)
1	2,832,041	2,443,504	384	0.166	7,375.107	6,363.294	100	54.804	133.929	0.717

Lane 5 - 2uM Thapsigargin

#	Vol. (Int.)	Local Bg. Corr. Vol.	Area	Rf	Density	Local Bg. Corr. Den.	% band purity	% lane purity	Mol. Wt.	Rel. Quant. (w/ LB Corr. Vol.)
1	2,673,672	2,276,693	408	0.168	6,553.118	5,580.132	100	55.54	132.143	0.668

Lane 7 - t = 0

#	Vol. (Int.)	Local Bg. Corr. Vol.	Area	Rf	Density	Local Bg. Corr. Den.	% band purity	% lane purity	Mol. Wt.	Rel. Quant. (w/ LB Corr. Vol.)
1	4,765,051	4,142,150	420	0.171	11,345	9,862.263	76.115	49.249	130.357	1.216
2	1,660,544	1,299,818	434	0.759	3,826.138	2,994.973	23.885	17.162	18.857	0.382

Lane 8 - Vehicle

#	Vol. (Int.)	Local Bg. Corr. Vol.	Area	Rf	Density	Local Bg. Corr. Den.	% band purity	% lane purity	Mol. Wt.	Rel. Quant. (w/ LB Corr. Vol.)
1	4,011,275	3,406,518	408	0.166	9,831.556	8,349.31	82.604	50.045	133.929	1
2	988,348	717,396	576	0.756	1,715.882	1,245.48	17.396	12.331	19	0.211

Lane 9 - 5uM Ionomycin

#	Vol. (Int.)	Local Bg. Corr. Vol.	Area	Rf	Density	Local Bg. Corr. Den.	% band purity	% lane purity	Mol. Wt.	Rel. Quant. (w/ LB Corr. Vol.)
1	5,185,725	4,519,330	448	0.161	11,575	10,087	65.947	44.653	137.5	1.327
2	2,730,386	2,333,646	510	0.754	5,353.698	4,575.778	34.053	23.511	19.143	0.685

Lane 10 - 2uM Thapsigargin

#	Vol. (Int.)	Local Bg. Corr. Vol.	Area	Rf	Density	Local Bg. Corr. Den.	% band purity	% lane purity	Mol. Wt.	Rel. Quant. (w/ LB Corr. Vol.)
1	4,496,406	4,036,229	416	0.158	10,808	9,702.474	63.271	45.241	139.286	1.185
2	2,643,071	2,343,043	595	0.746	4,442.136	3,937.889	36.729	26.594	19.571	0.688

iBright™ Image Analysis Report

Katarina+ Chang

18 November 2022

GAPDH_CHEMI_10162021_144358

Date:	16 October 2021 02:43:58PM
Mode:	Chemi Blots
Notes:	
Model:	FL1500
Instrument name:	2462619090234
Serial No:	2462619090234
Firmware version:	1.6.0
iBA version:	5.0
Image size:	676px X 540px
Image area:	125.22mm X 100.18mm
Optical Zoom:	1.8x
Digital Zoom:	1x
Focus level:	405
Resolution:	5 x 5
Exposure time:	10000 ms
Exposure mode:	Normal

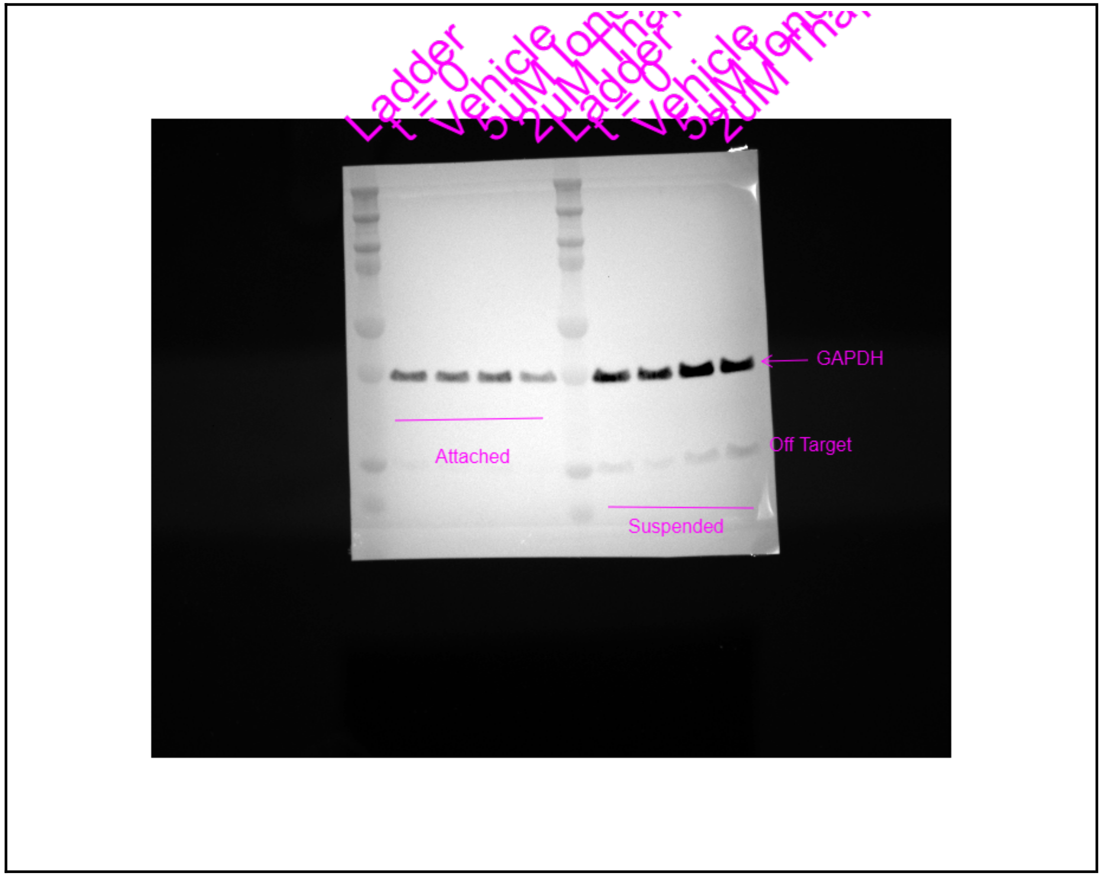

GAPDH_CHEMI_10162021_144358

Date:	16 October 2021 02:43:58PM
Mode:	Chemi Blots
Notes:	
Model:	FL1500
Instrument name:	2462619090234
Serial No:	2462619090234
Firmware version:	1.6.0
iBA version:	5.0
Image size:	676px X 540px
Image area:	125.22mm X 100.18mm
Optical Zoom:	1.8x
Digital Zoom:	1x
Focus level:	405
Resolution:	5 x 5
Exposure time:	10000 ms
Exposure mode:	Normal

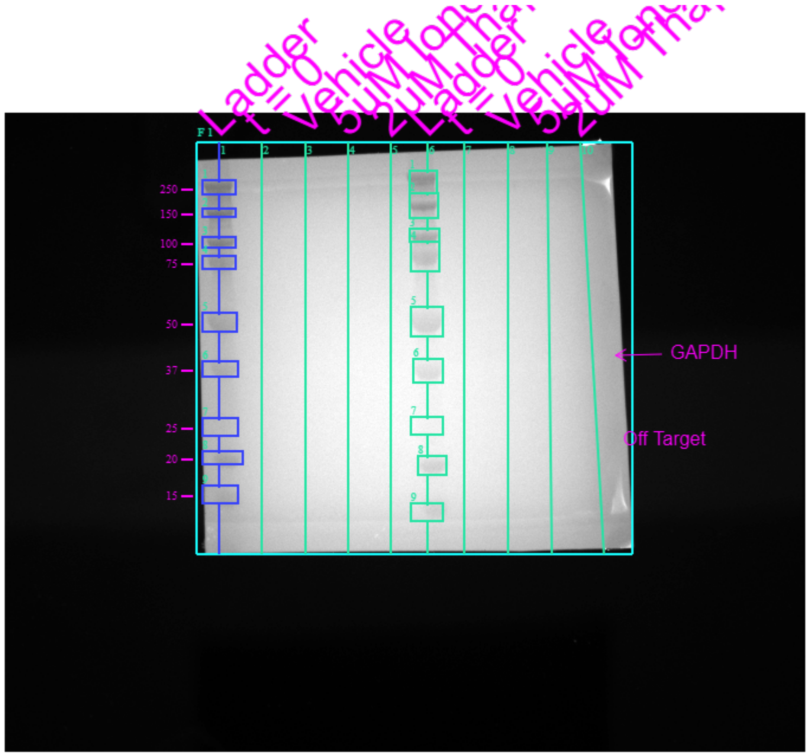

GAPDH_CHEMI_10162021_144358

Date:	16 October 2021 02:43:58PM
Mode:	Chemi Blots
Notes:	
Model:	FL1500
Instrument name:	2462619090234
Serial No:	2462619090234
Firmware version:	1.6.0
iBA version:	5.0
Image size:	676px X 540px
Image area:	125.22mm X 100.18mm
Optical Zoom:	1.8x
Digital Zoom:	1x
Focus level:	405
Resolution:	5 x 5
Exposure time:	10000 ms
Exposure mode:	Normal

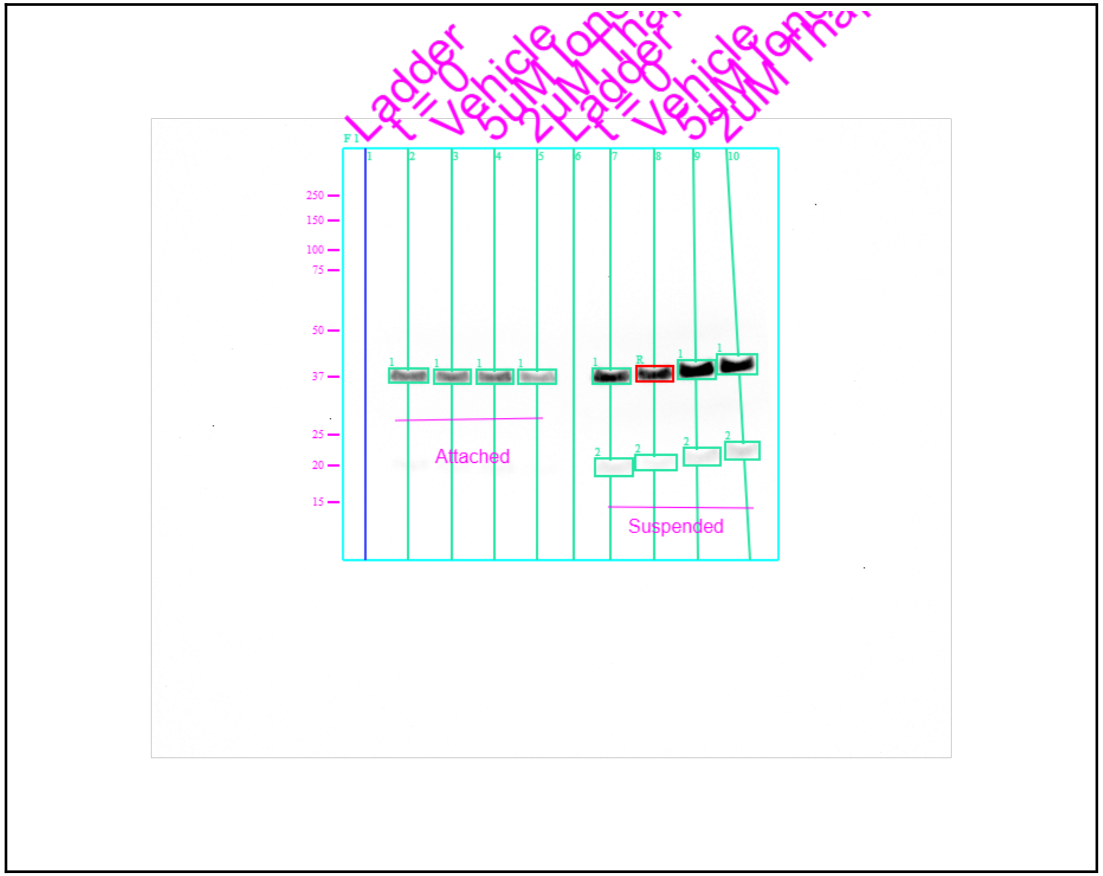

LANE AND BAND ANALYSIS DATA TABLE

GAPDH_CHEMI_10162021_144358

Frame: 1
Channel: Membrane
Sensitivity: 100
Molecular Weight Analysis Regression Method : Point to Point

Lane 1 - Ladder

#	Vol. (Int.)	Local Bg. Corr. Vol.	Area	Rf	Density	Local Bg. Corr. Den.	% band purity	% lane purity	Mol. Wt.
1	14,534,636	1,554,313	377	0.109	38,553	4,122.847	33.35	4.206	250
2	8,895,926	1,030,306	232	0.17	38,344	4,440.978	22.106	2.574	150
3	10,577,525	965,742	290	0.241	36,474	3,330.145	20.721	3.061	100
4	11,937,153	508,347	348	0.29	34,302	1,460.77	10.907	3.454	75
5	16,482,454	91,217	510	0.437	32,318	178.859	1.957	4.77	50
6	13,453,940	NA	434	0.549	30,999	NA	NA	3.893	37
7	14,688,584	NA	496	0.69	29,614	NA	NA	4.251	25
8	13,488,540	104,094	420	0.764	32,115	247.843	2.233	3.903	20
9	16,473,270	406,656	496	0.853	33,212	819.871	8.725	4.767	15

Lane 6 - Ladder

#	Vol. (Int.)	Local Bg. Corr. Vol.	Area	Rf	Density	Local Bg. Corr. Den.	% band purity	% lane purity	Mol. Wt.
1	16,348,176	2,127,167	480	0.095	34,058	4,431.599	17.76	6.479	NA
2	16,909,941	1,991,691	550	0.152	30,745	3,621.256	16.629	6.702	178.571
3	9,089,677	1,411,066	312	0.224	29,133	4,522.648	11.781	3.602	112
4	16,813,926	1,442,069	650	0.276	25,867	2,218.568	12.04	6.664	82.353
5	16,279,471	1,872,750	728	0.434	22,361	2,572.46	15.636	6.452	50.49
6	11,320,491	1,175,280	546	0.555	20,733	2,152.529	9.813	4.487	36.51
7	8,502,428	211,186	448	0.687	18,978	471.398	1.763	3.37	25.245
8	9,611,747	1,197,298	425	0.784	22,615	2,817.173	9.996	3.809	18.871
9	10,791,704	548,803	448	0.897	24,088	1,225.007	4.582	4.277	NA

Frame: 1
Channel: Chemi
Sensitivity: 100
Molecular Weight Analysis Regression Method : Point to Point

Lane 2 - t = 0

#	Vol. (Int.)	Local Bg. Corr. Vol.	Area	Rf	Density	Local Bg. Corr. Den.	% band purity	% lane purity	Mol. Wt.	Rel. Quant. (w/ LB Corr. Vol.)
1	4,014,781	3,781,242	442	0.552	9,083.215	8,554.847	100	76.587	36.755	0.64

Lane 3 - Vehicle

#	Vol. (Int.)	Local Bg. Corr. Vol.	Area	Rf	Density	Local Bg. Corr. Den.	% band purity	% lane purity	Mol. Wt.	Rel. Quant. (w/ LB Corr. Vol.)
1	3,504,950	3,229,813	416	0.555	8,425.361	7,763.975	100	69.275	36.51	0.547

Lane 4 - 5uM Ionomycin

#	Vol. (Int.)	Local Bg. Corr. Vol.	Area	Rf	Density	Local Bg. Corr. Den.	% band purity	% lane purity	Mol. Wt.	Rel. Quant. (w/ LB Corr. Vol.)
1	3,779,730	3,507,885	416	0.555	9,085.889	8,432.418	100	71.006	36.51	0.594

Lane 5 - 2uM Thapsigargin

#	Vol. (Int.)	Local Bg. Corr. Vol.	Area	Rf	Density	Local Bg. Corr. Den.	% band purity	% lane purity	Mol. Wt.	Rel. Quant. (w/ LB Corr. Vol.)
1	2,350,237	2,131,120	429	0.555	5,478.408	4,967.646	100	64.397	36.51	0.361

Lane 7 - t = 0

#	Vol. (Int.)	Local Bg. Corr. Vol.	Area	Rf	Density	Local Bg. Corr. Den.	% band purity	% lane purity	Mol. Wt.	Rel. Quant. (w/ LB Corr. Vol.)
1	7,165,613	6,668,310	462	0.552	15,509	14,433	93.034	76.998	36.755	1.129
2	585,458	499,312	528	0.773	1,108.822	945.668	6.966	6.291	19.516	0.085

Lane 8 - Vehicle

#	Vol. (Int.)	Local Bg. Corr. Vol.	Area	Rf	Density	Local Bg. Corr. Den.	% band purity	% lane purity	Mol. Wt.	Rel. Quant. (w/ LB Corr. Vol.)
1	6,653,138	5,903,895	448	0.546	14,850	13,178	96.729	71.584	37.333	1
2	328,961	199,660	504	0.761	652.7	396.152	3.271	3.539	20.192	0.034

Lane 9 - 5uM Ionomycin

#	Vol. (Int.)	Local Bg. Corr. Vol.	Area	Rf	Density	Local Bg. Corr. Den.	% band purity	% lane purity	Mol. Wt.	Rel. Quant. (w/ LB Corr. Vol.)

#	Vol. (Int.)	Local Bg. Corr. Vol.	Area	Rf	Density	Local Bg. Corr. Den.	% band purity	% lane purity	Mol. Wt.	Rel. Quant. (w/ LB Corr. Vol.)
1	11,818,333	10,605,589	561	0.537	21,066	18,904	94.883	79.926	38.333	1.796
2	714,731	571,976	512	0.747	1,395.959	1,117.142	5.117	4.834	21.154	0.097

Lane 10 - 2uM Thapsigargin

#	Vol. (Int.)	Local Bg. Corr. Vol.	Area	Rf	Density	Local Bg. Corr. Den.	% band purity	% lane purity	Mol. Wt.	Rel. Quant. (w/ LB Corr. Vol.)
1	9,628,796	8,699,989	630	0.523	15,283	13,809	90.939	80.121	40	1.474
2	967,986	866,895	480	0.733	2,016.638	1,806.033	9.061	8.055	22.115	0.147

iBright™ Image Analysis Report

Katarina+ Chang

18 November 2022

total cofilin_CHEMI_10142021_145000

Date:	14 October 2021 02:50:00PM
Mode:	Chemi Blots
Notes:	
Model:	FL1500
Instrument name:	2462619090234
Serial No:	2462619090234
Firmware version:	1.6.0
iBA version:	5.0
Image size:	676px X 540px
Image area:	118.63mm X 94.91mm
Optical Zoom:	1.9x
Digital Zoom:	1x
Focus level:	430
Resolution:	5 x 5
Exposure time:	12056 ms
Exposure mode:	Normal

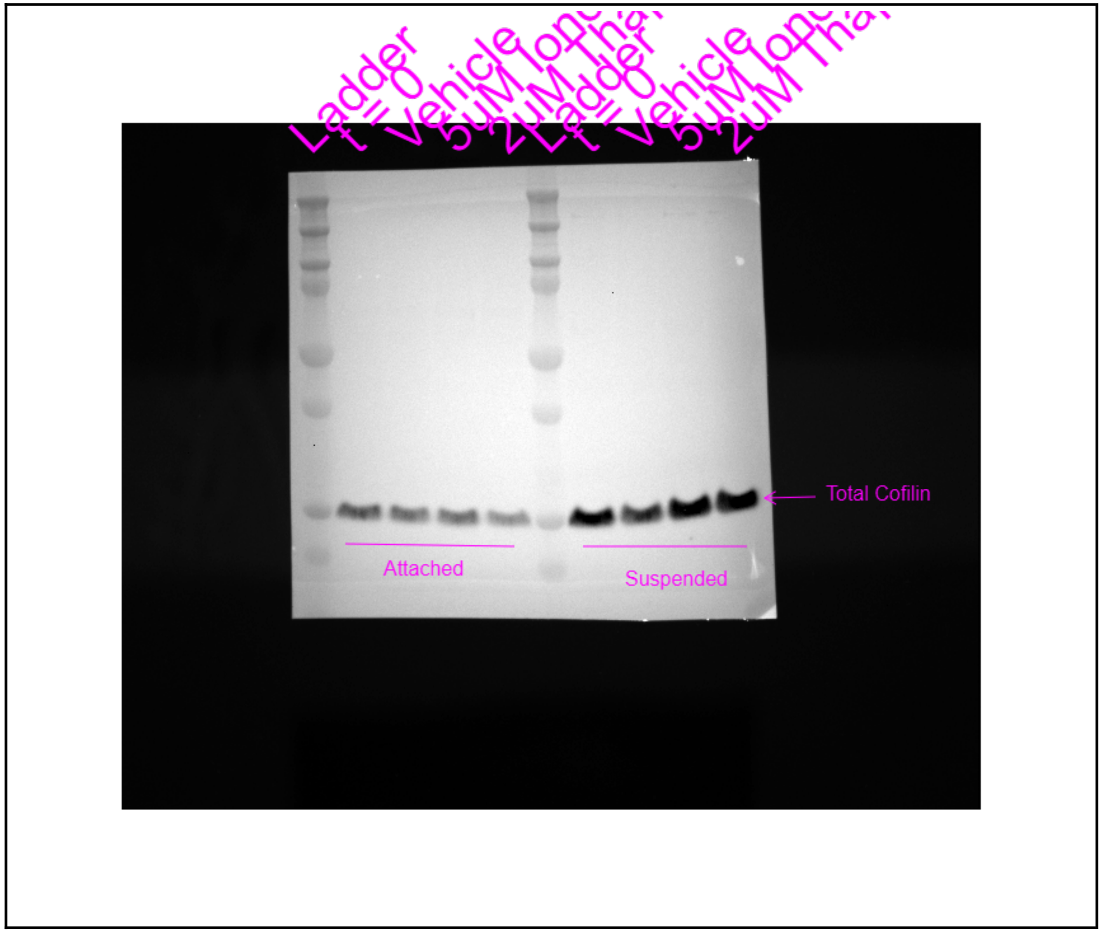

total cofilin_CHEMI_10142021_145000

Date:	14 October 2021 02:50:00PM
Mode:	Chemi Blots
Notes:	
Model:	FL1500
Instrument name:	2462619090234
Serial No:	2462619090234
Firmware version:	1.6.0
iBA version:	5.0
Image size:	676px X 540px
Image area:	118.63mm X 94.91mm
Optical Zoom:	1.9x
Digital Zoom:	1x
Focus level:	430
Resolution:	5 x 5
Exposure time:	12056 ms
Exposure mode:	Normal

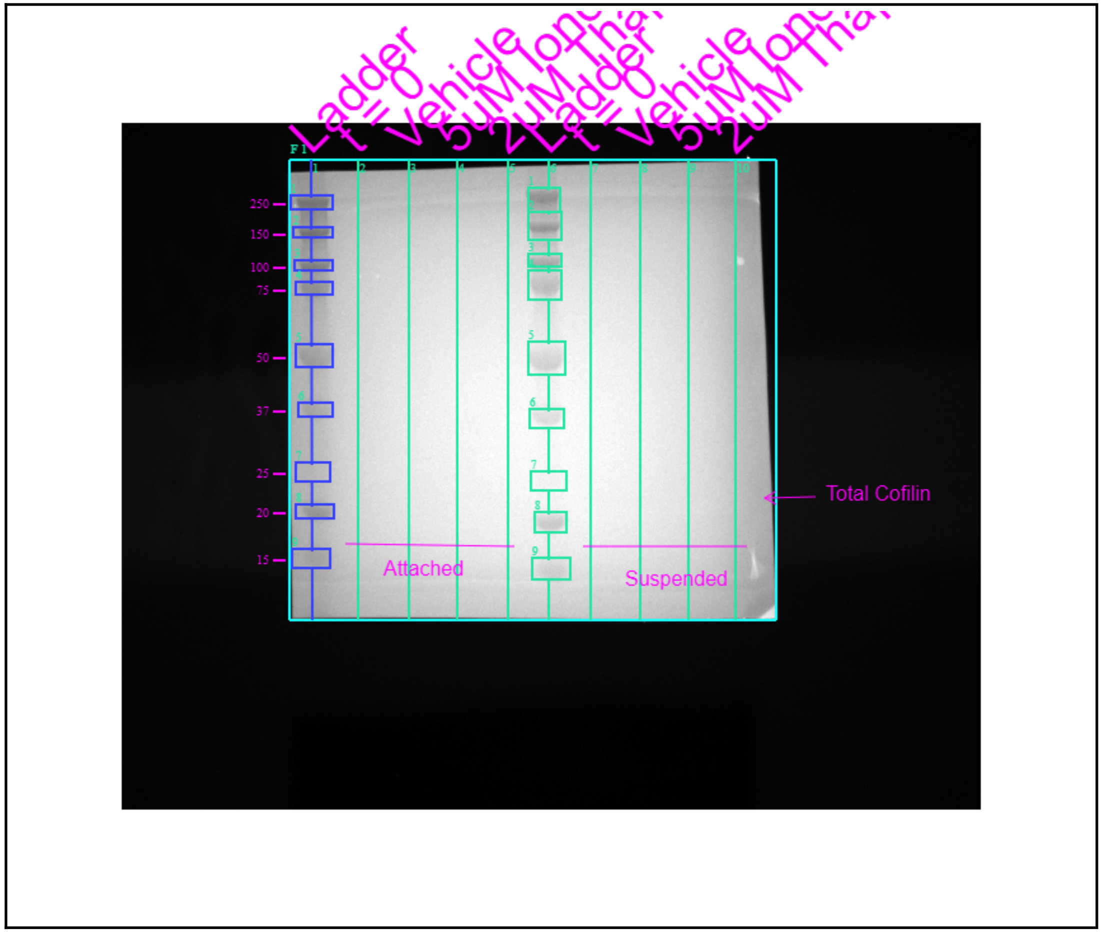

total cofilin_CHEMI_10142021_145000

Date:	14 October 2021 02:50:00PM
Mode:	Chemi Blots
Notes:	
Model:	FL1500
Instrument name:	2462619090234
Serial No:	2462619090234
Firmware version:	1.6.0
iBA version:	5.0
Image size:	676px X 540px
Image area:	118.63mm X 94.91mm
Optical Zoom:	1.9x
Digital Zoom:	1x
Focus level:	430
Resolution:	5 x 5
Exposure time:	12056 ms
Exposure mode:	Normal

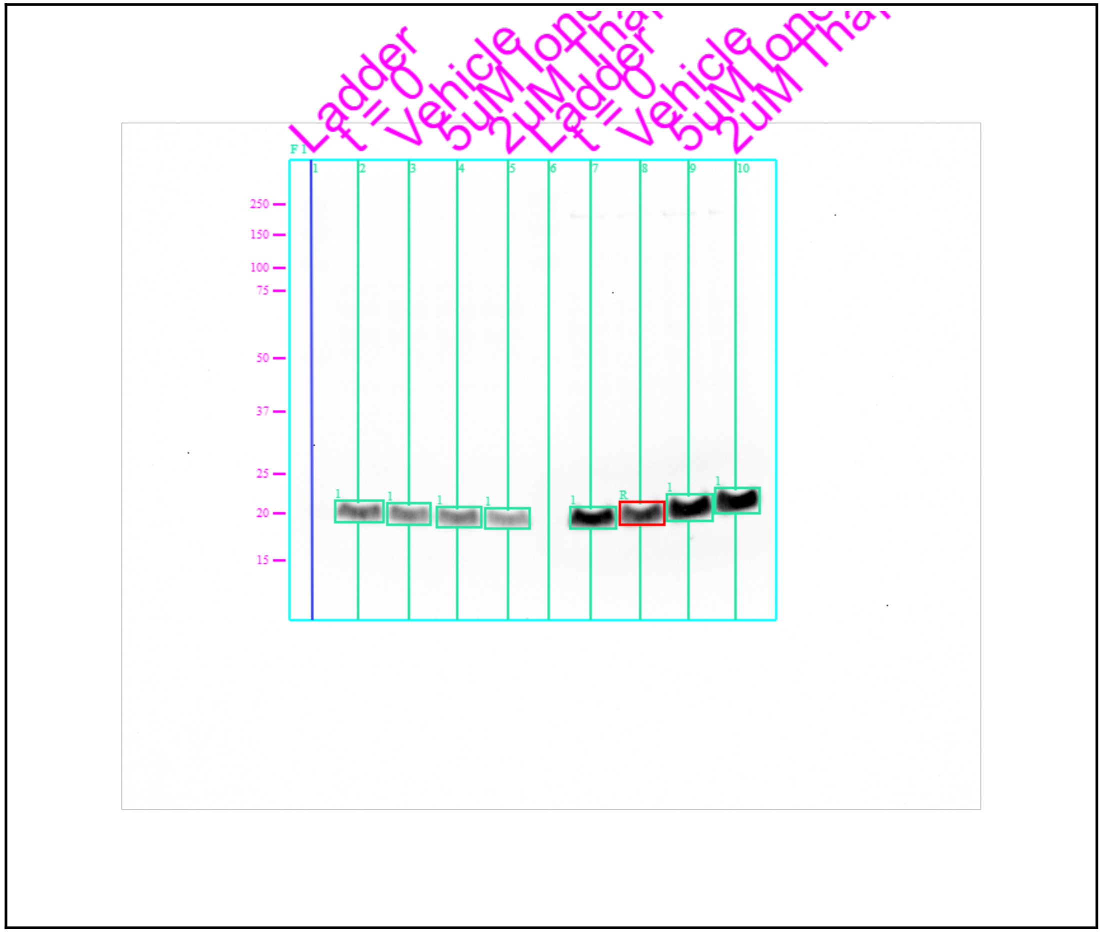

LANE AND BAND ANALYSIS DATA TABLE

total cofilin_CHEMI_10142021_145000

Frame: 1
Channel: Membrane
Sensitivity: 100
Molecular Weight Analysis Regression Method : Point to Point

Lane 1 - Ladder

#	Vol. (Int.)	Local Bg. Corr. Vol.	Area	Rf	Density	Local Bg. Corr. Den.	% band purity	% lane purity	Mol. Wt.
1	15,990,823	659,907	408	0.091	39,193	1,617.419	9.137	3.9	250
2	11,151,176	1,024,108	288	0.157	38,719	3,555.933	14.179	2.72	150
3	10,448,658	1,149,044	279	0.229	37,450	4,118.438	15.909	2.548	100
4	11,668,189	1,072,603	330	0.279	35,358	3,250.315	14.851	2.846	75
5	18,991,782	1,654,890	570	0.425	33,318	2,903.316	22.913	4.632	50
6	10,827,368	829,552	336	0.541	32,224	2,468.905	11.486	2.641	37
7	13,624,383	67,769	448	0.677	30,411	151.271	0.938	3.323	25
8	12,598,021	764,402	372	0.762	33,865	2,054.846	10.583	3.073	20
9	16,757,581	316.523	496	0.865	33,785	0.638	0.004	4.087	15

Lane 6 - Ladder

#	Vol. (Int.)	Local Bg. Corr. Vol.	Area	Rf	Density	Local Bg. Corr. Den.	% band purity	% lane purity	Mol. Wt.
1	17,690,693	2,421,935	520	0.086	34,020	4,657.568	17.089	6.077	NA
2	19,161,136	2,353,071	621	0.144	30,855	3,789.165	16.603	6.582	170.833
3	8,829,150	1,494,625	297	0.218	29,727	5,032.409	10.546	3.033	107.692
4	16,824,621	1,763,426	648	0.271	25,963	2,721.338	12.443	5.779	79.167
5	18,114,638	2,311,580	810	0.431	22,363	2,853.803	16.311	6.222	49.381
6	9,377,057	1,010,622	448	0.561	20,930	2,255.855	7.131	3.221	35.286
7	8,862,534	205,396	464	0.696	19,100	442.665	1.449	3.044	23.871
8	10,436,384	1,624,561	442	0.787	23,611	3,675.479	11.463	3.585	18.784
9	13,516,095	987,018	558	0.887	24,222	1,768.85	6.964	4.643	NA

Frame: 1
Channel: Chemi
Sensitivity: 100
Molecular Weight Analysis Regression Method : Point to Point

Lane 2 - t = 0

#	Vol. (Int.)	Local Bg. Corr. Vol.	Area	Rf	Density	Local Bg. Corr. Den.	% band purity	% lane purity	Mol. Wt.	Rel. Quant. (w/ LB Corr. Vol.)
1	8,754,594	7,763,619	702	0.762	12,470	11,059	100	69.788	20	0.822

Lane 3 - Vehicle

#	Vol. (Int.)	Local Bg. Corr. Vol.	Area	Rf	Density	Local Bg. Corr. Den.	% band purity	% lane purity	Mol. Wt.	Rel. Quant. (w/ LB Corr. Vol.)
1	6,911,179	5,828,621	630	0.768	10,970	9,251.781	100	60.731	19.73	0.617

Lane 4 - 5uM Ionomycin

#	Vol. (Int.)	Local Bg. Corr. Vol.	Area	Rf	Density	Local Bg. Corr. Den.	% band purity	% lane purity	Mol. Wt.	Rel. Quant. (w/ LB Corr. Vol.)
1	7,196,003	6,095,166	612	0.776	11,758	9,959.423	100	62.109	19.324	0.646

Lane 5 - 2uM Thapsigargin

#	Vol. (Int.)	Local Bg. Corr. Vol.	Area	Rf	Density	Local Bg. Corr. Den.	% band purity	% lane purity	Mol. Wt.	Rel. Quant. (w/ LB Corr. Vol.)
1	5,410,776	4,539,198	612	0.779	8,841.137	7,416.991	100	56.396	19.189	0.481

Lane 7 - t = 0

#	Vol. (Int.)	Local Bg. Corr. Vol.	Area	Rf	Density	Local Bg. Corr. Den.	% band purity	% lane purity	Mol. Wt.	Rel. Quant. (w/ LB Corr. Vol.)
1	15,937,813	13,697,410	666	0.776	23,930	20,566	100	74.392	19.324	1.451

Lane 8 - Vehicle

#	Vol. (Int.)	Local Bg. Corr. Vol.	Area	Rf	Density	Local Bg. Corr. Den.	% band purity	% lane purity	Mol. Wt.	Rel. Quant. (w/ LB Corr. Vol.)
1	12,555,449	9,439,267	684	0.768	18,355	13,800	100	66.216	19.73	1

Lane 9 - 5uM Ionomycin

#	Vol. (Int.)	Local Bg. Corr. Vol.	Area	Rf	Density	Local Bg. Corr. Den.	% band purity	% lane purity	Mol. Wt.	Rel. Quant. (w/ LB Corr. Vol.)
1	19,215,788	15,223,912	814	0.754	23,606	18,702	100	74.345	20.484	1.613

Lane 10 - 2uM Thapsigargin

#	Vol. (Int.)	Local Bg. Corr. Vol.	Area	Rf	Density	Local Bg. Corr. Den.	% band purity	% lane purity	Mol. Wt.	Rel. Quant. (w/ LB Corr. Vol.)
1	19,461,137	17,075,863	756	0.74	25,742	22,587	100	77.204	21.29	1.809

iBright™ Image Analysis Report

Katarina+ Chang

18 November 2022

detyrosinated tubulin_CHEMI_10132021_122753

Date:	13 October 2021 12:27:53PM
Mode:	Chemi Blots
Notes:	
Model:	FL1500
Instrument name:	2462619090234
Serial No:	2462619090234
Firmware version:	1.6.0
iBA version:	5.0
Image size:	615px X 491px
Image area:	112.7mm X 90.16mm
Optical Zoom:	2x
Digital Zoom:	1.1x
Focus level:	455
Resolution:	5 x 5
Exposure time:	120000 ms
Exposure mode:	Normal

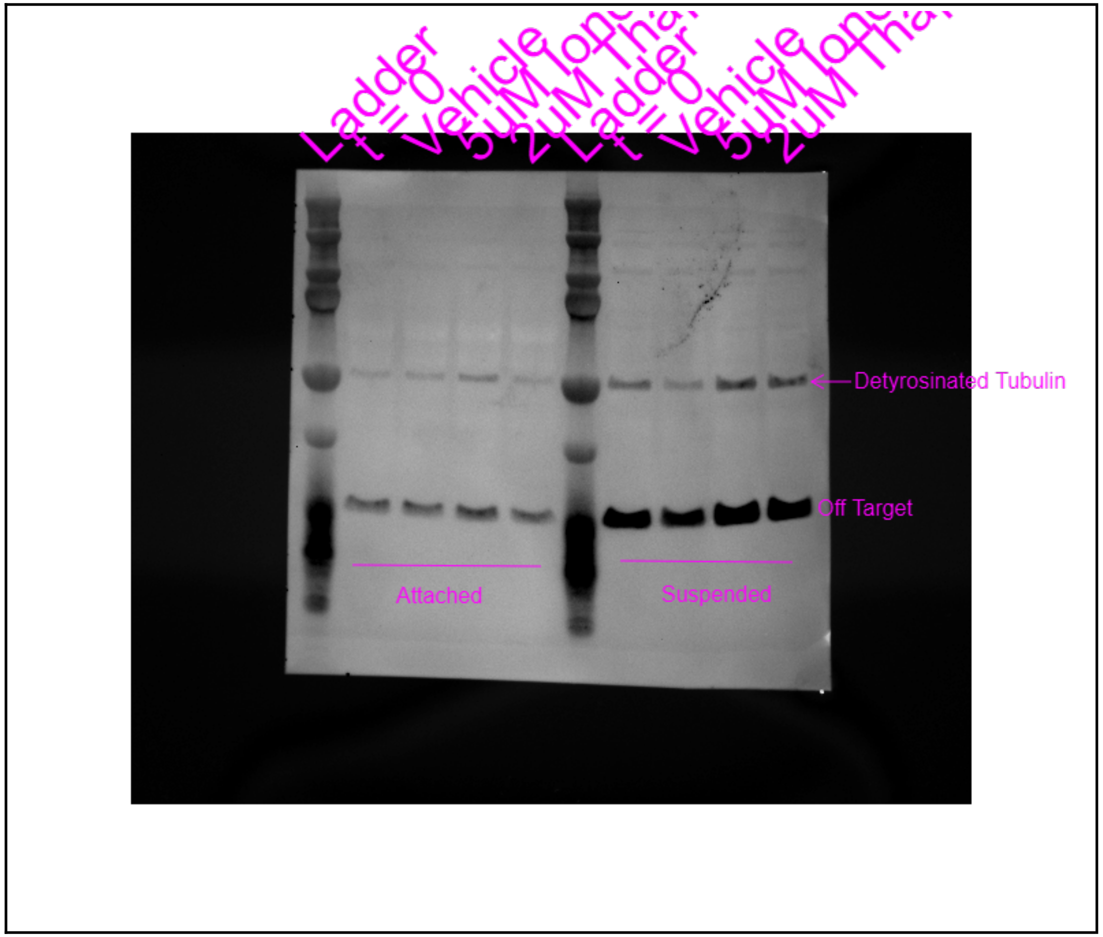

detyrosinated tubulin_CHEMI_10132021_122753

Date:	13 October 2021 12:27:53PM
Mode:	Chemi Blots
Notes:	
Model:	FL1500
Instrument name:	2462619090234
Serial No:	2462619090234
Firmware version:	1.6.0
iBA version:	5.0
Image size:	615px X 491px
Image area:	112.7mm X 90.16mm
Optical Zoom:	2x
Digital Zoom:	1.1x
Focus level:	455
Resolution:	5 x 5
Exposure time:	120000 ms
Exposure mode:	Normal

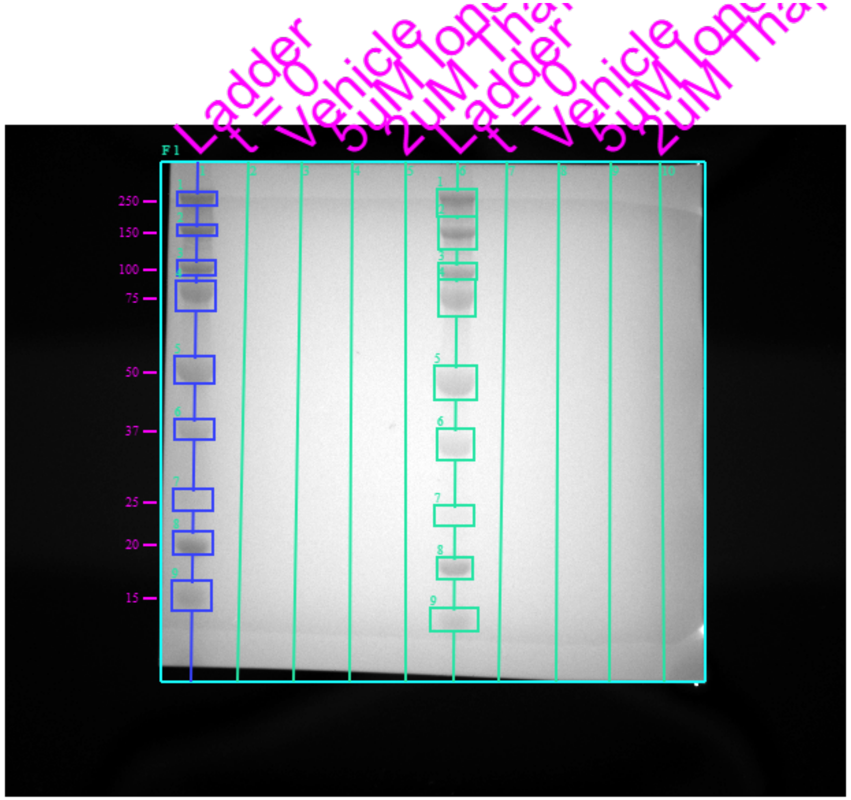

detyrosinated tubulin_CHEMI_10132021_122753

Date:	13 October 2021 12:27:53PM
Mode:	Chemi Blots
Notes:	
Model:	FL1500
Instrument name:	2462619090234
Serial No:	2462619090234
Firmware version:	1.6.0
iBA version:	5.0
Image size:	615px X 491px
Image area:	112.7mm X 90.16mm
Optical Zoom:	2x
Digital Zoom:	1.1x
Focus level:	455
Resolution:	5 x 5
Exposure time:	120000 ms
Exposure mode:	Normal

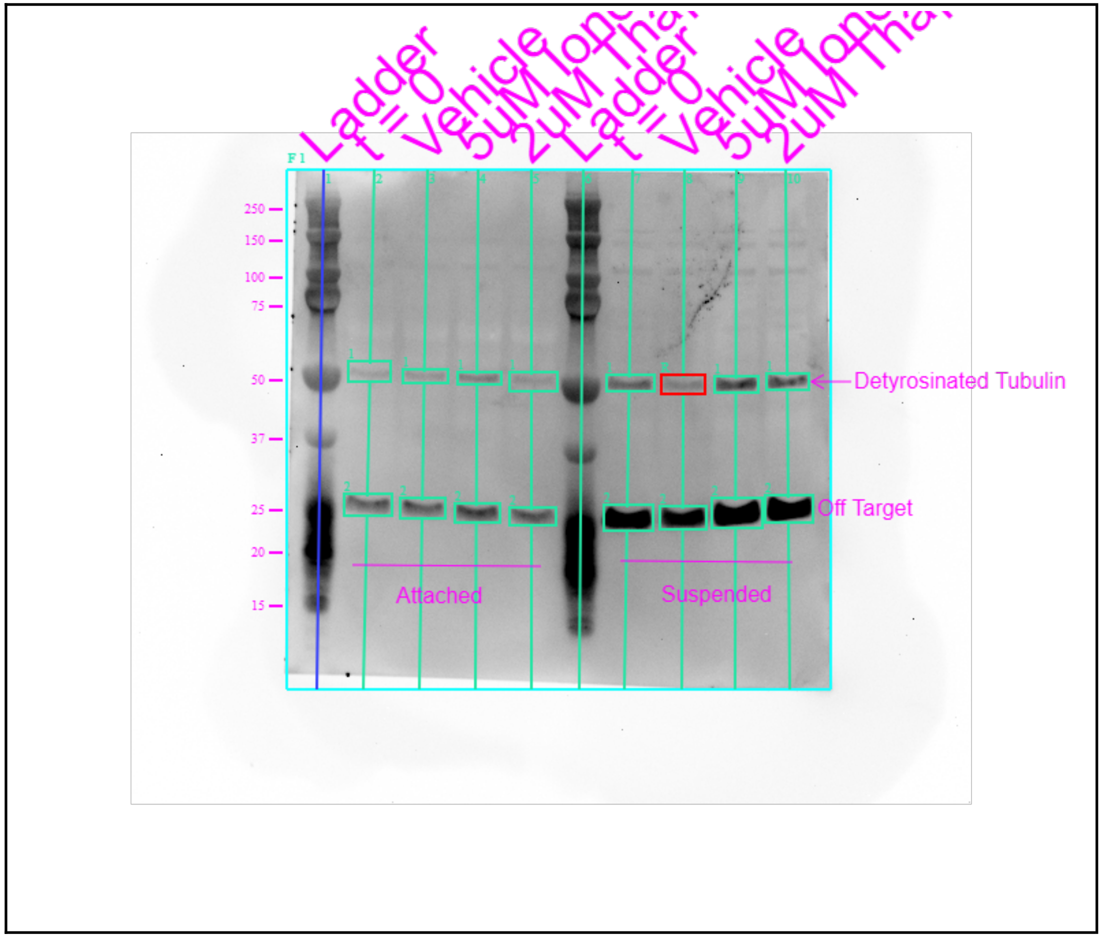

LANE AND BAND ANALYSIS DATA TABLE

detyrosinated tubulin_CHEMI_10132021_122753

Frame: 1
Channel: Membrane
Sensitivity: 100
Molecular Weight Analysis Regression Method : Point to Point

Lane 1 - Ladder

#	Vol. (Int.)	Local Bg. Corr. Vol.	Area	Rf	Density	Local Bg. Corr. Den.	% band purity	% lane purity	Mol. Wt.
1	13,398,471	1,646,185	330	0.071	40,601	4,988.442	11.282	3.566	250
2	10,674,261	1,401,772	270	0.132	39,534	5,191.75	9.607	2.841	150
3	12,993,721	1,617,949	348	0.203	37,338	4,649.28	11.088	3.458	100
4	23,752,636	2,140,052	690	0.258	34,424	3,101.525	14.666	6.321	75
5	20,971,799	2,350,870	630	0.4	33,288	3,731.54	16.111	5.581	50
6	15,137,113	1,323,805	480	0.513	31,535	2,757.928	9.072	4.028	37
7	15,299,057	564,948	510	0.65	29,998	1,107.743	3.872	4.071	25
8	18,204,389	2,259,781	540	0.732	33,711	4,184.781	15.487	4.844	20
9	22,809,700	1,286,099	690	0.834	33,057	1,863.913	8.814	6.07	15

Lane 6 - Ladder

#	Vol. (Int.)	Local Bg. Corr. Vol.	Area	Rf	Density	Local Bg. Corr. Den.	% band purity	% lane purity	Mol. Wt.
1	21,169,801	2,976,854	630	0.079	33,602	4,725.166	16.752	7.67	236.957
2	21,935,769	2,711,456	725	0.137	30,256	3,739.941	15.259	7.948	146.296
3	10,841,542	1,807,449	377	0.211	28,757	4,794.295	10.171	3.928	96.429
4	20,328,270	2,339,823	784	0.261	25,928	2,984.468	13.167	7.365	74.537
5	18,781,013	2,817,234	832	0.424	22,573	3,386.099	15.854	6.805	47.279
6	13,973,067	1,593,405	672	0.542	20,793	2,371.139	8.967	5.063	34.462
7	9,521,320	396,017	480	0.679	19,836	825.037	2.229	3.45	23.226
8	11,705,379	2,122,660	459	0.782	25,501	4,624.533	11.945	4.241	17.564
9	16,588,595	1,005,109	648	0.879	25,599	1,551.095	5.656	6.01	NA

Frame: 1
Channel: Chemi
Sensitivity: 100
Molecular Weight Analysis Regression Method : Point to Point

Lane 2 - t = 0

#	Vol. (Int.)	Local Bg. Corr. Vol.	Area	Rf	Density	Local Bg. Corr. Den.	% band purity	% lane purity	Mol. Wt.	Rel. Quant. (w/ LB Corr. Vol.)
1	10,859,504	837,235	512	0.387	21,209	1,635.227	12.366	5.217	52.315	0.488
2	17,909,650	5,933,060	612	0.645	29,264	9,694.544	87.634	8.604	25.462	3.46

Lane 3 - Vehicle

#	Vol. (Int.)	Local Bg. Corr. Vol.	Area	Rf	Density	Local Bg. Corr. Den.	% band purity	% lane purity	Mol. Wt.	Rel. Quant. (w/ LB Corr. Vol.)
1	9,075,879	830,960	374	0.397	24,267	2,221.819	13.662	3.97	50.463	0.485
2	17,092,455	5,251,111	544	0.65	31,419	9,652.778	86.338	7.477	25	3.062

Lane 4 - 5uM Ionomycin

#	Vol. (Int.)	Local Bg. Corr. Vol.	Area	Rf	Density	Local Bg. Corr. Den.	% band purity	% lane purity	Mol. Wt.	Rel. Quant. (w/ LB Corr. Vol.)
1	9,195,779	1,530,470	330	0.4	27,865	4,637.791	18.587	3.906	50	0.893
2	18,352,520	6,703,542	510	0.661	35,985	13,144	81.413	7.795	24.355	3.91

Lane 5 - 2uM Thapsigargin

#	Vol. (Int.)	Local Bg. Corr. Vol.	Area	Rf	Density	Local Bg. Corr. Den.	% band purity	% lane purity	Mol. Wt.	Rel. Quant. (w/ LB Corr. Vol.)
1	14,097,923	387,264	540	0.408	26,107	717.156	8.345	5.745	49.093	0.226
2	16,853,467	4,253,649	490	0.666	34,394	8,680.917	91.655	6.867	24.032	2.481

Lane 7 - t = 0

#	Vol. (Int.)	Local Bg. Corr. Vol.	Area	Rf	Density	Local Bg. Corr. Den.	% band purity	% lane purity	Mol. Wt.	Rel. Quant. (w/ LB Corr. Vol.)
1	15,180,658	2,990,967	468	0.411	32,437	6,390.955	15.321	5.435	48.791	1.744
2	37,951,791	16,530,976	740	0.668	51,286	22,339	84.679	13.588	23.871	9.641

Lane 8 - Vehicle

#	Vol. (Int.)	Local Bg. Corr. Vol.	Area	Rf	Density	Local Bg. Corr. Den.	% band purity	% lane purity	Mol. Wt.	Rel. Quant. (w/ LB Corr. Vol.)
1	14,059,114	1,714,673	495	0.413	28,402	3,463.988	11.045	5.108	48.488	1

#	Vol. (Int.)	Local Bg. Corr. Vol.	Area	Rf	Density	Local Bg. Corr. Den.	% band purity	% lane purity	Mol. Wt.	Rel. Quant. (w/ LB Corr. Vol.)
2	31,195,481	13,810,067	630	0.668	49,516	21,920	88.955	11.333	23.871	8.054

Lane 9 - 5uM Ionomycin

#	Vol. (Int.)	Local Bg. Corr. Vol.	Area	Rf	Density	Local Bg. Corr. Den.	% band purity	% lane purity	Mol. Wt.	Rel. Quant. (w/ LB Corr. Vol.)
1	15,328,803	5,252,293	429	0.413	35,731	12,243	21.49	5.851	48.488	3.063
2	42,452,785	19,187,957	874	0.661	48,572	21,954	78.51	16.204	24.355	11.19

Lane 10 - 2uM Thapsigargin

#	Vol. (Int.)	Local Bg. Corr. Vol.	Area	Rf	Density	Local Bg. Corr. Den.	% band purity	% lane purity	Mol. Wt.	Rel. Quant. (w/ LB Corr. Vol.)
1	13,393,952	4,178,796	416	0.408	32,197	10,045	17.776	5.393	49.093	2.437
2	37,422,948	19,329,294	756	0.653	49,501	25,567	82.224	15.067	24.839	11.273

iBright™ Image Analysis Report

Katarina+ Chang

18 November 2022

GAPDH_CHEMI_10162021_143848

Date:	16 October 2021 02:38:48PM
Mode:	Chemi Blots
Notes:	
Model:	FL1500
Instrument name:	2462619090234
Serial No:	2462619090234
Firmware version:	1.6.0
iBA version:	5.0
Image size:	676px X 540px
Image area:	118.63mm X 94.91mm
Optical Zoom:	1.9x
Digital Zoom:	1x
Focus level:	430
Resolution:	5 x 5
Exposure time:	12605 ms
Exposure mode:	Normal

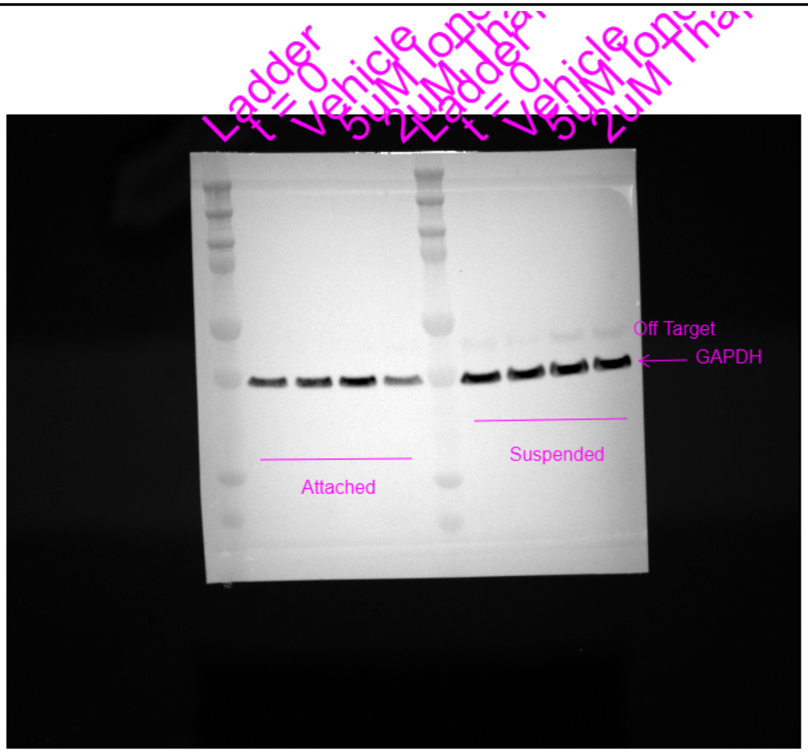

GAPDH_CHEMI_10162021_143848

Date:	16 October 2021 02:38:48PM
Mode:	Chemi Blots
Notes:	
Model:	FL1500
Instrument name:	2462619090234
Serial No:	2462619090234
Firmware version:	1.6.0
iBA version:	5.0
Image size:	676px X 540px
Image area:	118.63mm X 94.91mm
Optical Zoom:	1.9x
Digital Zoom:	1x
Focus level:	430
Resolution:	5 x 5
Exposure time:	12605 ms
Exposure mode:	Normal

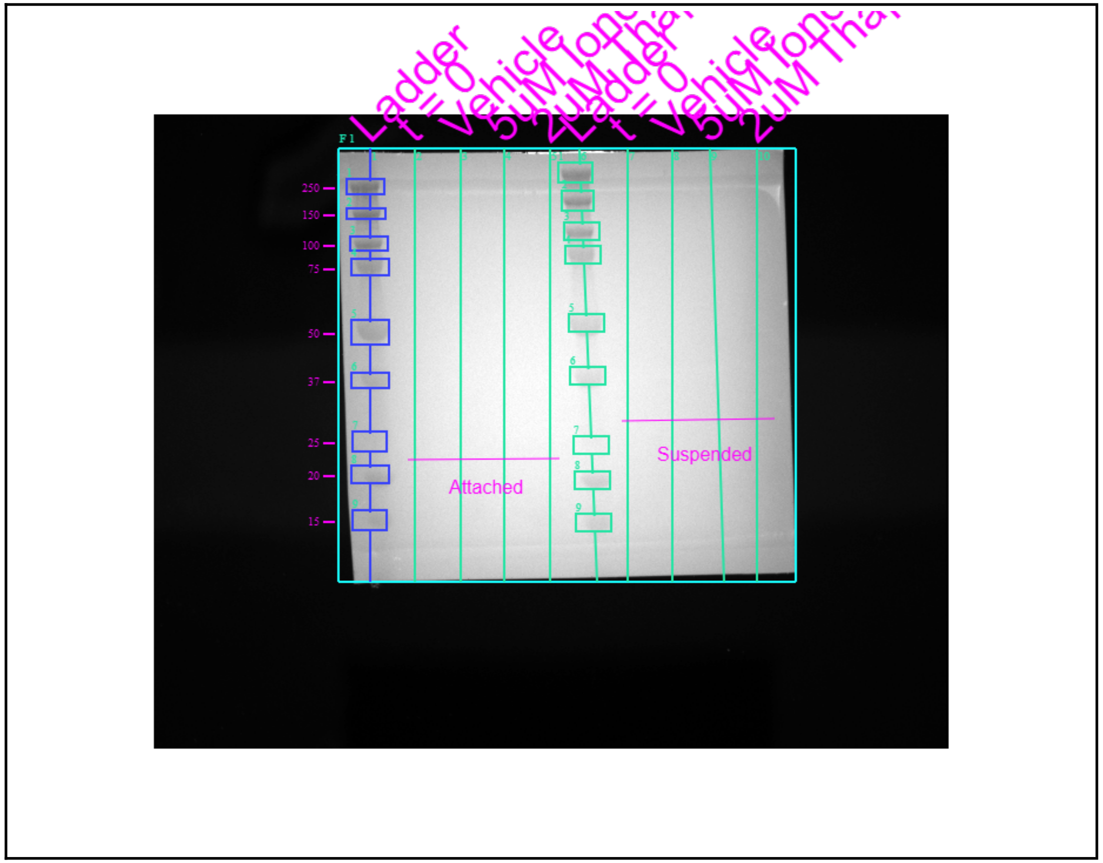

GAPDH_CHEMI_10162021_143848

Date:	16 October 2021 02:38:48PM
Mode:	Chemi Blots
Notes:	
Model:	FL1500
Instrument name:	2462619090234
Serial No:	2462619090234
Firmware version:	1.6.0
iBA version:	5.0
Image size:	676px X 540px
Image area:	118.63mm X 94.91mm
Optical Zoom:	1.9x
Digital Zoom:	1x
Focus level:	430
Resolution:	5 x 5
Exposure time:	12605 ms
Exposure mode:	Normal

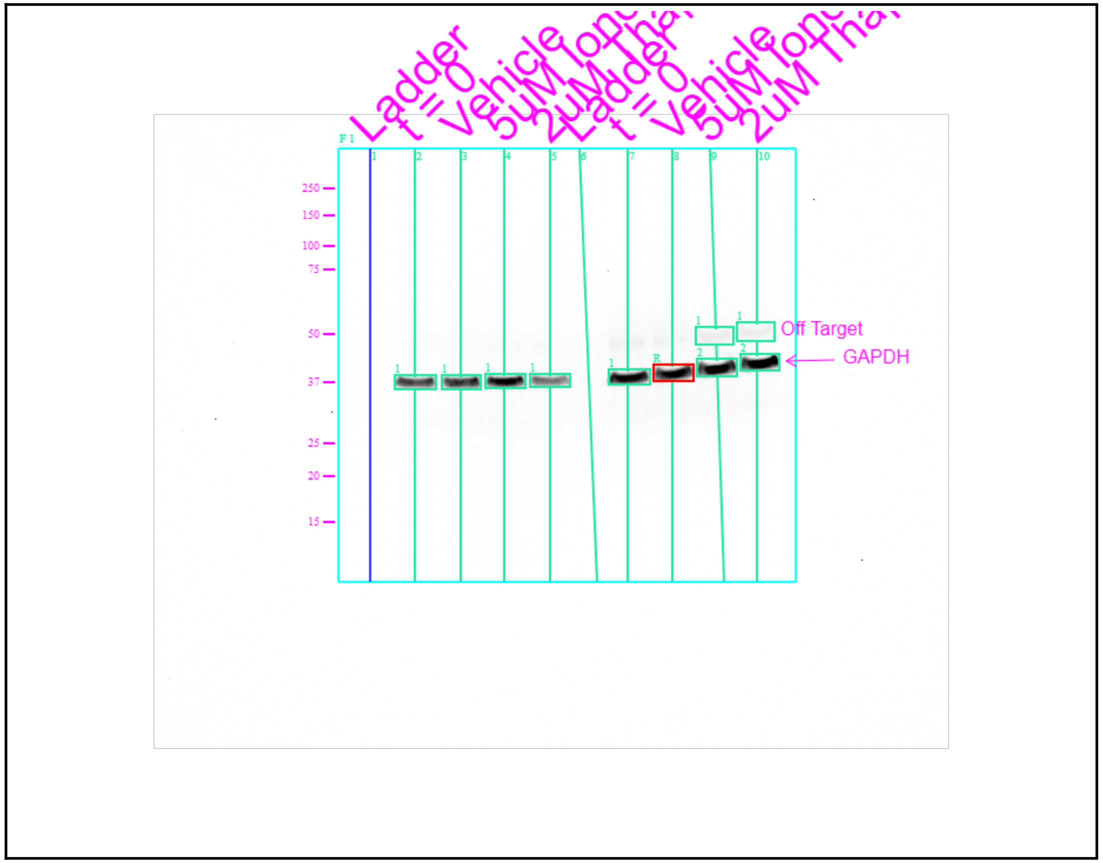

LANE AND BAND ANALYSIS DATA TABLE

GAPDH_CHEMI_10162021_143848

Frame: 1
Channel: Membrane
Sensitivity: 100
Molecular Weight Analysis Regression Method : Point to Point

Lane 1 - Ladder

#	Vol. (Int.)	Local Bg. Corr. Vol.	Area	Rf	Density	Local Bg. Corr. Den.	% band purity	% lane purity	Mol. Wt.
1	17,733,173	1,941,724	462	0.087	38,383	4,202.868	19.412	4.598	250
2	12,582,214	1,471,675	340	0.149	37,006	4,328.457	14.713	3.263	150
3	14,944,535	1,696,964	429	0.22	34,835	3,955.628	16.965	3.875	100
4	16,323,871	1,476,870	495	0.274	32,977	2,983.577	14.765	4.233	75
5	22,893,151	1,978,917	726	0.423	31,533	2,725.782	19.784	5.936	50
6	14,268,141	984,367	462	0.534	30,883	2,130.666	9.841	3.7	37
7	16,094,178	308,844	540	0.675	29,804	571.934	3.088	4.173	25
8	16,828,239	143,104	528	0.751	31,871	271.032	1.431	4.364	20
9	17,706,966	NA	540	0.856	32,790	NA	NA	4.592	15

Lane 6 - Ladder

#	Vol. (Int.)	Local Bg. Corr. Vol.	Area	Rf	Density	Local Bg. Corr. Den.	% band purity	% lane purity	Mol. Wt.
1	18,560,900	2,090,331	540	0.054	34,372	3,870.984	17.087	6.317	NA
2	16,012,064	2,111,977	504	0.119	31,769	4,190.431	17.264	5.449	197.826
3	14,160,132	1,850,282	496	0.19	28,548	3,730.408	15.125	4.819	121.154
4	13,095,712	1,521,604	496	0.244	26,402	3,067.751	12.438	4.457	88.75
5	11,800,603	1,428,223	496	0.401	23,791	2,879.483	11.675	4.016	53.636
6	10,833,038	1,191,409	496	0.523	21,840	2,402.034	9.739	3.687	38.268
7	10,018,012	209,281	496	0.683	20,197	421.938	1.711	3.409	24.464
8	11,499,150	1,121,136	496	0.764	23,183	2,260.357	9.165	3.913	19.359
9	12,155,594	708,987	496	0.862	24,507	1,429.411	5.796	4.137	NA

Frame: 1
Channel: Chemi
Sensitivity: 100
Molecular Weight Analysis Regression Method : Point to Point

Lane 2 - t = 0

#	Vol. (Int.)	Local Bg. Corr. Vol.	Area	Rf	Density	Local Bg. Corr. Den.	% band purity	% lane purity	Mol. Wt.	Rel. Quant. (w/ LB Corr. Vol.)
1	5,863,282	5,587,607	468	0.539	12,528	11,939	100	76.776	36.538	0.741

Lane 3 - Vehicle

#	Vol. (Int.)	Local Bg. Corr. Vol.	Area	Rf	Density	Local Bg. Corr. Den.	% band purity	% lane purity	Mol. Wt.	Rel. Quant. (w/ LB Corr. Vol.)
1	6,372,041	5,930,298	442	0.539	14,416	13,416	100	71.928	36.538	0.786

Lane 4 - 5uM Ionomycin

#	Vol. (Int.)	Local Bg. Corr. Vol.	Area	Rf	Density	Local Bg. Corr. Den.	% band purity	% lane purity	Mol. Wt.	Rel. Quant. (w/ LB Corr. Vol.)
1	7,621,853	7,090,901	455	0.537	16,751	15,584	100	73.429	36.769	0.94

Lane 5 - 2uM Thapsigargin

#	Vol. (Int.)	Local Bg. Corr. Vol.	Area	Rf	Density	Local Bg. Corr. Den.	% band purity	% lane purity	Mol. Wt.	Rel. Quant. (w/ LB Corr. Vol.)
1	4,282,010	3,901,197	420	0.534	10,195	9,288.565	100	59.593	37	0.517

Lane 7 - t = 0

#	Vol. (Int.)	Local Bg. Corr. Vol.	Area	Rf	Density	Local Bg. Corr. Den.	% band purity	% lane purity	Mol. Wt.	Rel. Quant. (w/ LB Corr. Vol.)
1	8,996,370	8,075,776	504	0.526	17,849	16,023	100	73.395	37.951	1.07

Lane 8 - Vehicle

#	Vol. (Int.)	Local Bg. Corr. Vol.	Area	Rf	Density	Local Bg. Corr. Den.	% band purity	% lane purity	Mol. Wt.	Rel. Quant. (w/ LB Corr. Vol.)
1	8,734,069	7,544,318	525	0.518	16,636	14,370	100	72.833	38.902	1

Lane 9 - 5uM Ionomycin

#	Vol. (Int.)	Local Bg. Corr. Vol.	Area	Rf	Density	Local Bg. Corr. Den.	% band purity	% lane purity	Mol. Wt.	Rel. Quant. (w/ LB Corr. Vol.)
1	859,684	465,619	528	0.431	1,628.189	881.856	4.923	6.297	49.049	0.062
2	10,283,002	8,993,128	560	0.504	18,362	16,059	95.077	75.318	40.488	1.192

Lane 10 - 2uM Thapsigargin

#	Vol. (Int.)	Local Bg. Corr. Vol.	Area	Rf	Density	Local Bg. Corr. Den.	% band purity	% lane purity	Mol. Wt.	Rel. Quant. (w/ LB Corr. Vol.)
1	795,804	478,550	561	0.423	1,418.545	853.031	4.61	6.129	50	0.063
2	10,509,758	9,901,184	510	0.493	20,607	19,414	95.39	80.946	41.756	1.312

iBright™ Image Analysis Report

Katarina+ Chang

18 November 2022

pCofilin CHEMI_10142021_150512

Date:	14 October 2021 03:05:12PM
Mode:	Chemi Blots
Notes:	
Model:	FL1500
Instrument name:	2462619090234
Serial No:	2462619090234
Firmware version:	1.6.0
iBA version:	5.0
Image size:	676px X 540px
Image area:	125.22mm X 100.18mm
Optical Zoom:	1.8x
Digital Zoom:	1x
Focus level:	405
Resolution:	5 x 5
Exposure time:	18242 ms
Exposure mode:	Normal

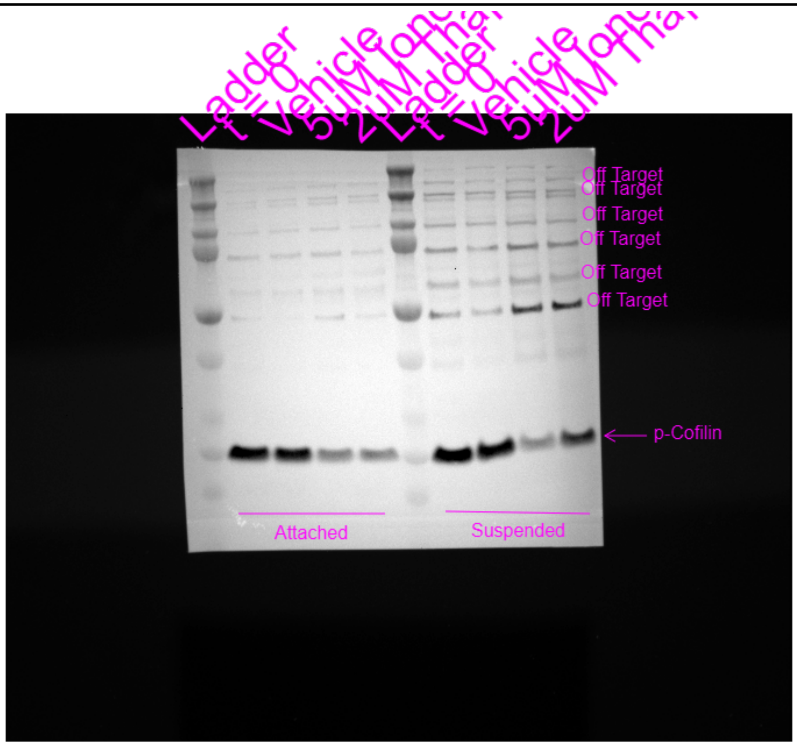

pCofilin CHEMI_10142021_150512

Date:	14 October 2021 03:05:12PM
Mode:	Chemi Blots
Notes:	
Model:	FL1500
Instrument name:	2462619090234
Serial No:	2462619090234
Firmware version:	1.6.0
iBA version:	5.0
Image size:	676px X 540px
Image area:	125.22mm X 100.18mm
Optical Zoom:	1.8x
Digital Zoom:	1x
Focus level:	405
Resolution:	5 x 5
Exposure time:	18242 ms
Exposure mode:	Normal

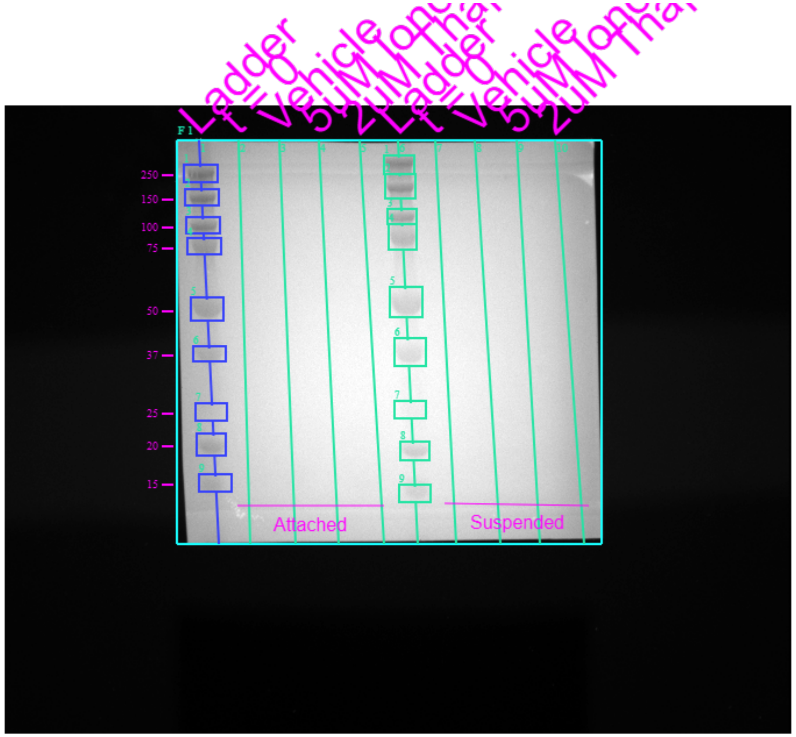

pCofilin CHEMI_10142021_150512

Date:	14 October 2021 03:05:12PM
Mode:	Chemi Blots
Notes:	
Model:	FL1500
Instrument name:	2462619090234
Serial No:	2462619090234
Firmware version:	1.6.0
iBA version:	5.0
Image size:	676px X 540px
Image area:	125.22mm X 100.18mm
Optical Zoom:	1.8x
Digital Zoom:	1x
Focus level:	405
Resolution:	5 x 5
Exposure time:	18242 ms
Exposure mode:	Normal

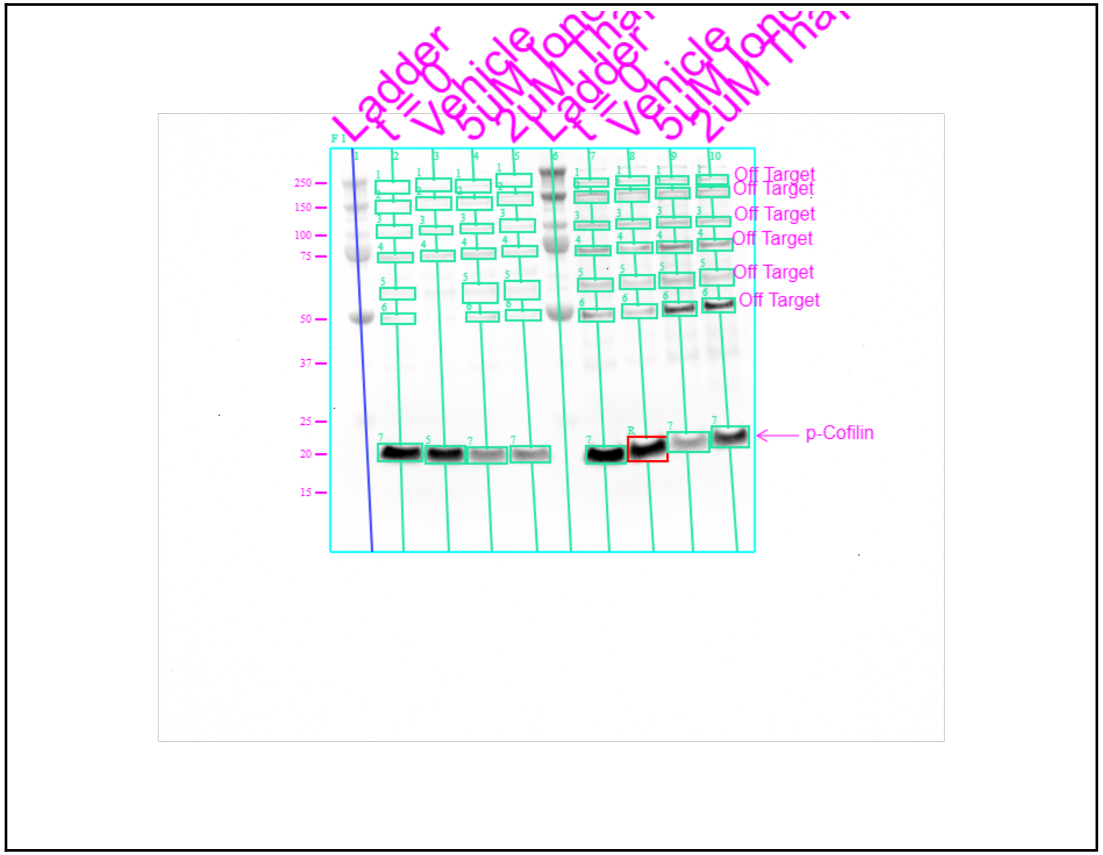

LANE AND BAND ANALYSIS DATA TABLE

pCofilin CHEMI_10142021_150512

Frame: 1
Channel: Membrane
Sensitivity: 100
Molecular Weight Analysis Regression Method : Point to Point

Lane 1 - Ladder

#	Vol. (Int.)	Local Bg. Corr. Vol.	Area	Rf	Density	Local Bg. Corr. Den.	% band purity	% lane purity	Mol. Wt.
1	18,380,461	1,822,440	480	0.081	38,292	3,796.751	15.28	5.85	250
2	16,513,943	1,646,102	450	0.141	36,697	3,658.007	13.802	5.256	150
3	15,783,108	1,597,023	450	0.21	35,073	3,548.942	13.39	5.023	100
4	15,239,673	1,418,912	450	0.262	33,865	3,153.139	11.897	4.85	75
5	19,455,774	1,868,991	609	0.418	31,947	3,068.951	15.671	6.192	50
6	12,473,159	1,050,615	406	0.527	30,722	2,587.724	8.809	3.97	37
7	13,007,114	435,441	448	0.671	29,033	971.969	3.651	4.14	25
8	16,372,974	1,257,297	520	0.752	31,486	2,417.879	10.542	5.211	20
9	14,569,171	829,868	464	0.847	31,399	1,788.51	6.958	4.637	15

Lane 6 - Ladder

#	Vol. (Int.)	Local Bg. Corr. Vol.	Area	Rf	Density	Local Bg. Corr. Den.	% band purity	% lane purity	Mol. Wt.
1	15,885,072	2,163,098	459	0.061	34,608	4,712.634	15.766	1.07	NA
2	18,797,488	2,349,297	594	0.112	31,645	3,955.046	17.123	1.266	197.619
3	10,711,004	1,654,545	364	0.187	29,425	4,545.454	12.059	0.721	116.667
4	15,379,457	1,627,093	575	0.239	26,746	2,829.728	11.859	1.036	86.111
5	18,003,650	2,361,900	783	0.401	22,993	3,016.476	17.214	1.213	52.778
6	14,619,793	1,503,084	700	0.524	20,885	2,147.263	10.955	0.985	37.342
7	8,790,179	313,169	448	0.666	19,620	699.04	2.283	0.592	25.48
8	10,119,122	1,173,583	442	0.769	22,893	2,655.166	8.554	0.682	19.091
9	10,673,904	574,655	448	0.873	23,825	1,282.714	4.188	0.719	NA

Frame: 1
Channel: Chemi
Sensitivity: 100
Molecular Weight Analysis Regression Method : Point to Point

Lane 2 - t = 0

#	Vol. (Int.)	Local Bg. Corr. Vol.	Area	Rf	Density	Local Bg. Corr. Den.	% band purity	% lane purity	Mol. Wt.	Rel. Quant. (w/ LB Corr. Vol.)
1	182,073	89,250	360	0.095	505.758	247.917	0.642	0.009	226.19	0.008
2	318,750	196,478	372	0.144	856.855	528.168	1.413	0.016	147.917	0.017
3	358,928	227,105	372	0.205	964.86	610.498	1.633	0.018	104.167	0.019
4	630,666	509,320	279	0.271	2,260.452	1,825.52	3.663	0.032	73.611	0.043
5	440,364	276,201	341	0.36	1,291.39	809.975	1.986	0.022	59.259	0.024
6	352,122	247,215	300	0.421	1,173.74	824.051	1.778	0.018	49.658	0.021
7	14,178,103	12,359,617	624	0.752	22,721	19,807	88.885	0.722	20	1.052

Lane 3 - Vehicle

#	Vol. (Int.)	Local Bg. Corr. Vol.	Area	Rf	Density	Local Bg. Corr. Den.	% band purity	% lane purity	Mol. Wt.	Rel. Quant. (w/ LB Corr. Vol.)
1	246,762	123,404	372	0.089	663.339	331.731	1.125	0.014	235.714	0.011
2	438,238	303,226	372	0.135	1,178.059	815.125	2.765	0.025	159.524	0.026
3	331,946	218,940	232	0.202	1,430.802	943.71	1.997	0.019	106.25	0.019
4	613,483	479,244	300	0.265	2,044.943	1,597.481	4.371	0.035	74.537	0.041
5	11,718,006	9,839,891	560	0.758	20,925	17,571	89.741	0.671	19.697	0.838

Lane 4 - 5uM Ionomycin

#	Vol. (Int.)	Local Bg. Corr. Vol.	Area	Rf	Density	Local Bg. Corr. Den.	% band purity	% lane purity	Mol. Wt.	Rel. Quant. (w/ LB Corr. Vol.)
1	318,312	94,414	372	0.092	855.677	253.802	1.528	0.021	230.952	0.008
2	612,429	409,695	372	0.135	1,646.315	1,101.332	6.629	0.04	159.524	0.035
3	436,764	288,200	261	0.199	1,673.425	1,104.217	4.663	0.028	108.333	0.025
4	751,523	597,649	300	0.259	2,505.077	1,992.165	9.67	0.049	76.389	0.051
5	744,040	461,382	558	0.357	1,333.405	826.85	7.465	0.048	59.722	0.039
6	525,483	409,951	261	0.418	2,013.345	1,570.695	6.633	0.034	50	0.035
7	5,319,721	3,919,408	525	0.761	10,132	7,465.539	63.414	0.346	19.545	0.334

Lane 5 - 2uM Thapsigargin

#	Vol. (Int.)	Local Bg. Corr. Vol.	Area	Rf	Density	Local Bg. Corr. Den.	% band purity	% lane purity	Mol. Wt.	Rel. Quant. (w/ LB Corr. Vol.)
1	337,667	126,358	372	0.078	907.707	339.674	2.071	0.025	NA	0.011
2	661,350	426,892	372	0.124	1,777.823	1,147.561	6.998	0.05	178.571	0.036
3	494,023	249,007	372	0.19	1,328.019	669.375	4.082	0.037	114.583	0.021
4	591,783	344,401	310	0.254	1,908.977	1,110.973	5.646	0.044	79.167	0.029
5	766,698	396,440	527	0.352	1,454.835	752.259	6.499	0.058	60.648	0.034
6	392,114	177,388	310	0.412	1,264.884	572.221	2.908	0.029	50.926	0.015
7	5,093,214	4,379,771	525	0.758	9,701.36	8,342.422	71.796	0.383	19.697	0.373

Lane 7 - t = 0

#	Vol. (Int.)	Local Bg. Corr. Vol.	Area	Rf	Density	Local Bg. Corr. Den.	% band purity	% lane purity	Mol. Wt.	Rel. Quant. (w/ LB Corr. Vol.)
1	604,327	311,163	240	0.084	2,518.029	1,296.513	1.606	0.066	245.238	0.026
2	1,600,395	1,210,164	330	0.121	4,849.682	3,667.165	6.246	0.174	183.333	0.103
3	1,127,096	771,240	248	0.19	4,544.742	3,109.843	3.98	0.122	114.583	0.066
4	1,752,683	1,422,340	279	0.254	6,282.018	5,097.996	7.341	0.19	79.167	0.121
5	1,507,046	1,066,444	372	0.337	4,051.199	2,866.785	5.504	0.164	62.963	0.091
6	1,964,150	1,612,394	372	0.412	5,279.973	4,334.395	8.322	0.213	50.926	0.137
7	15,596,493	12,982,117	560	0.758	27,850	23,182	67.001	1.694	19.697	1.105

Lane 8 - Vehicle

#	Vol. (Int.)	Local Bg. Corr. Vol.	Area	Rf	Density	Local Bg. Corr. Den.	% band purity	% lane purity	Mol. Wt.	Rel. Quant. (w/ LB Corr. Vol.)
1	445,689	190,199	261	0.081	1,707.621	728.732	1.196	0.063	250	0.016
2	1,255,593	923,715	360	0.115	3,487.758	2,565.875	5.811	0.177	192.857	0.079
3	953,689	607,755	270	0.187	3,532.181	2,250.946	3.823	0.134	116.667	0.052
4	1,292,147	911,418	310	0.245	4,168.216	2,940.058	5.733	0.182	83.333	0.078
5	1,127,483	649,870	403	0.331	2,797.725	1,612.581	4.088	0.159	63.889	0.055
6	1,286,060	865,580	403	0.403	3,191.216	2,147.843	5.445	0.181	52.315	0.074
7	14,350,236	11,748,160	770	0.744	18,636	15,257	73.903	2.018	20.536	1

Lane 9 - 5uM Ionomycin

#	Vol. (Int.)	Local Bg. Corr. Vol.	Area	Rf	Density	Local Bg. Corr. Den.	% band purity	% lane purity	Mol. Wt.	Rel. Quant. (w/ LB Corr. Vol.)
1	512,290	283,357	232	0.078	2,208.147	1,221.369	2.454	0.107	NA	0.024

#	Vol. (Int.)	Local Bg. Corr. Vol.	Area	Rf	Density	Local Bg. Corr. Den.	% band purity	% lane purity	Mol. Wt.	Rel. Quant. (w/ LB Corr. Vol.)
2	1,441,743	1,110,265	300	0.11	4,805.81	3,700.884	9.615	0.301	202.381	0.095
3	1,343,175	891,703	300	0.182	4,477.25	2,972.347	7.722	0.28	120.833	0.076
4	2,517,140	1,986,502	341	0.245	7,381.642	5,825.52	17.203	0.525	83.333	0.169
5	2,096,974	1,326,203	434	0.326	4,831.737	3,055.768	11.485	0.437	64.815	0.113
6	4,325,225	3,785,206	390	0.398	11,090	9,705.657	32.779	0.902	53.241	0.322
7	5,017,966	2,164,482	666	0.726	7,534.483	3,249.973	18.744	1.046	21.607	0.184

Lane 10 - 2uM Thapsigargin

#	Vol. (Int.)	Local Bg. Corr. Vol.	Area	Rf	Density	Local Bg. Corr. Den.	% band purity	% lane purity	Mol. Wt.	Rel. Quant. (w/ LB Corr. Vol.)
1	552,806	258,107	252	0.078	2,193.675	1,024.235	1.587	0.273	NA	0.022
2	1,276,954	987,583	290	0.107	4,403.29	3,405.462	6.071	0.631	207.143	0.084
3	933,797	619,442	270	0.182	3,458.507	2,294.231	3.808	0.461	120.833	0.053
4	1,861,833	1,470,131	341	0.239	5,459.921	4,311.235	9.038	0.92	86.111	0.125
5	1,555,787	951,465	450	0.32	3,457.304	2,114.369	5.849	0.769	65.741	0.081
6	4,350,610	3,906,938	377	0.389	11,540	10,363	24.019	2.149	54.63	0.333
7	9,016,389	8,072,330	627	0.715	14,380	12,874	49.627	4.454	22.321	0.687

iBright™ Image Analysis Report

Katarina+ Chang

18 November 2022

Total MLCK_CHEMI_10132021_121955

Date:	13 October 2021 12:19:55PM
Mode:	Chemi Blots
Notes:	
Model:	FL1500
Instrument name:	2462619090234
Serial No:	2462619090234
Firmware version:	1.6.0
iBA version:	5.0
Image size:	563px X 450px
Image area:	112.7mm X 90.16mm
Optical Zoom:	2x
Digital Zoom:	1.2x
Focus level:	455
Resolution:	5 x 5
Exposure time:	120000 ms
Exposure mode:	Normal

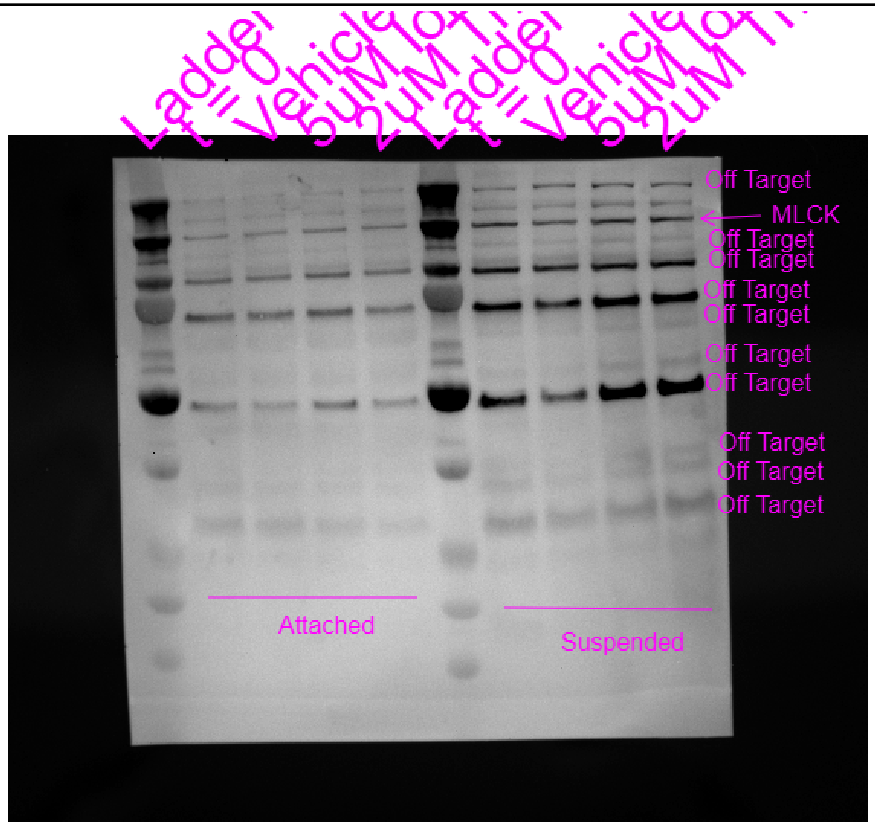

Total MLCK_CHEMI_10132021_121955

Date:	13 October 2021 12:19:55PM
Mode:	Chemi Blots
Notes:	
Model:	FL1500
Instrument name:	2462619090234
Serial No:	2462619090234
Firmware version:	1.6.0
iBA version:	5.0
Image size:	563px X 450px
Image area:	112.7mm X 90.16mm
Optical Zoom:	2x
Digital Zoom:	1.2x
Focus level:	455
Resolution:	5 x 5
Exposure time:	120000 ms
Exposure mode:	Normal

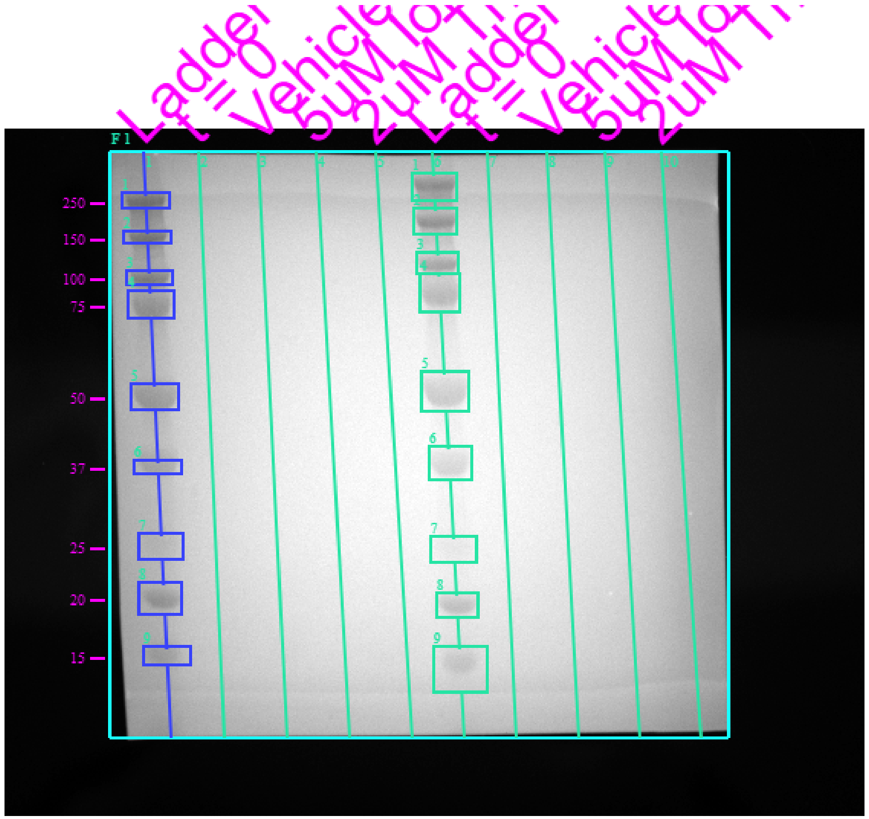

Total MLCK_CHEMI_10132021_121955

Date:	13 October 2021 12:19:55PM
Mode:	Chemi Blots
Notes:	
Model:	FL1500
Instrument name:	2462619090234
Serial No:	2462619090234
Firmware version:	1.6.0
iBA version:	5.0
Image size:	563px X 450px
Image area:	112.7mm X 90.16mm
Optical Zoom:	2x
Digital Zoom:	1.2x
Focus level:	455
Resolution:	5 x 5
Exposure time:	120000 ms
Exposure mode:	Normal

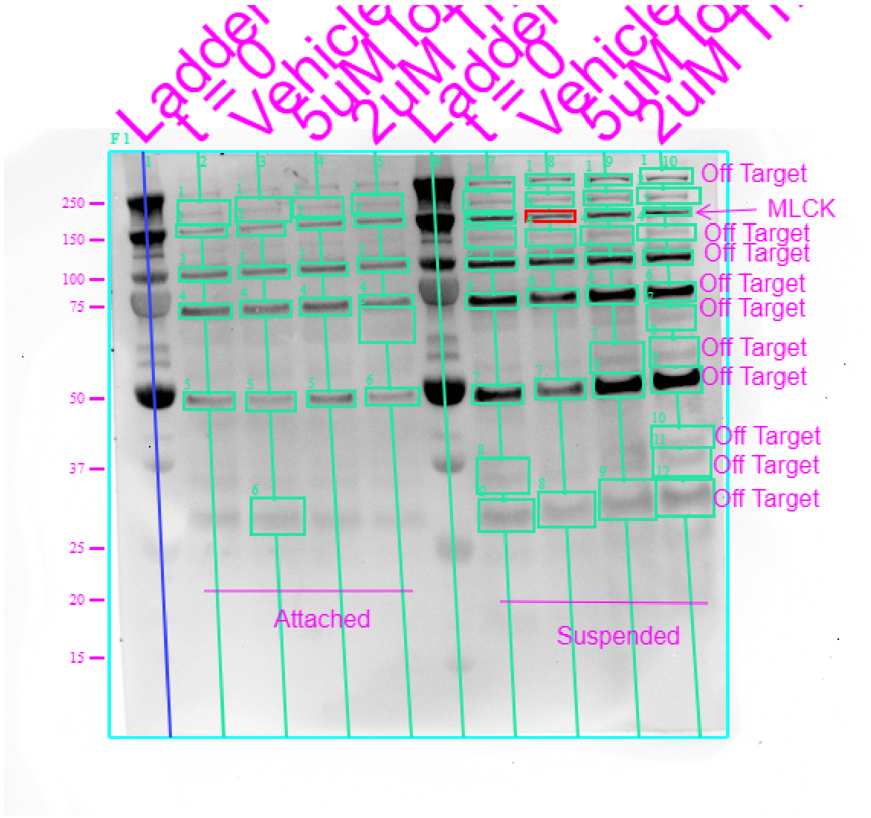

LANE AND BAND ANALYSIS DATA TABLE

Total MLCK_CHEMI_10132021_121955

Frame: 1
Channel: Membrane
Sensitivity: 100
Molecular Weight Analysis Regression Method : Point to Point

Lane 1 - Ladder

#	Vol. (Int.)	Local Bg. Corr. Vol.	Area	Rf	Density	Local Bg. Corr. Den.	% band purity	% lane purity	Mol. Wt.
1	14,037,351	1,767,676	352	0.083	39,878	5,021.807	13.358	3.724	250
2	11,012,727	1,444,798	288	0.146	38,238	5,016.662	10.918	2.922	150
3	11,339,949	1,549,748	310	0.214	36,580	4,999.187	11.711	3.009	100
4	20,036,561	1,963,700	589	0.26	34,017	3,333.957	14.84	5.316	75
5	18,991,327	2,346,564	576	0.417	32,971	4,073.897	17.733	5.039	50
6	10,011,462	860,804	320	0.536	31,285	2,690.013	6.505	2.656	37
7	16,064,619	633,647	540	0.672	29,749	1,173.421	4.788	4.262	25
8	20,859,062	1,794,145	638	0.76	32,694	2,812.14	13.558	5.534	20
9	13,677,533	871,839	416	0.859	32,878	2,095.769	6.588	3.629	15

Lane 6 - Ladder

#	Vol. (Int.)	Local Bg. Corr. Vol.	Area	Rf	Density	Local Bg. Corr. Den.	% band purity	% lane purity	Mol. Wt.
1	19,017,713	2,710,375	570	0.06	33,364	4,755.045	14.703	6.8	NA
2	16,197,070	2,406,866	522	0.117	31,028	4,610.856	13.056	5.792	195.833
3	11,940,154	2,141,871	420	0.19	28,428	5,099.694	11.619	4.27	117.308
4	18,017,974	2,393,389	702	0.24	25,666	3,409.387	12.983	6.443	86.111
5	19,206,394	3,213,827	864	0.409	22,229	3,719.708	17.434	6.868	51.25
6	13,824,400	1,902,804	667	0.531	20,726	2,852.781	10.322	4.943	37.565
7	11,080,299	491,055	558	0.677	19,857	880.028	2.664	3.962	24.706
8	11,570,053	1,656,965	476	0.773	24,306	3,481.02	8.988	4.137	19.342
9	27,874,310	1,517,238	1,116	0.883	24,976	1,359.532	8.23	9.967	NA

Frame: 1
Channel: Chemi
Sensitivity: 100
Molecular Weight Analysis Regression Method : Point to Point

Lane 2 - t = 0

#	Vol. (Int.)	Local Bg. Corr. Vol.	Area	Rf	Density	Local Bg. Corr. Den.	% band purity	% lane purity	Mol. Wt.	Rel. Quant. (w/ LB Corr. Vol.)
1	11,440,858	249,685	595	0.104	19,228	419.64	2.541	6.838	216.667	0.104
2	6,768,576	984,578	306	0.135	22,119	3,217.578	10.018	4.045	166.667	0.408
3	9,396,135	2,362,758	340	0.208	27,635	6,949.289	24.041	5.616	103.846	0.98
4	10,781,982	3,737,988	350	0.271	30,805	10,679	38.033	6.444	73.333	1.551
5	9,622,445	2,493,144	408	0.424	23,584	6,110.649	25.367	5.751	49.152	1.034

Lane 3 - Vehicle

#	Vol. (Int.)	Local Bg. Corr. Vol.	Area	Rf	Density	Local Bg. Corr. Den.	% band purity	% lane purity	Mol. Wt.	Rel. Quant. (w/ LB Corr. Vol.)
1	12,955,259	188,396	629	0.099	20,596	299.518	1.952	7.203	225	0.078
2	7,485,562	984,029	320	0.13	23,392	3,075.091	10.196	4.162	175	0.408
3	8,273,809	1,818,786	306	0.206	27,038	5,943.747	18.846	4.6	105.769	0.754
4	10,441,997	2,984,627	374	0.268	27,919	7,980.287	30.926	5.806	73.75	1.238
5	9,306,753	1,565,242	429	0.427	21,694	3,648.584	16.219	5.174	48.87	0.649
6	14,754,495	2,109,701	900	0.622	16,393	2,344.112	21.86	8.203	29.385	0.875

Lane 4 - 5uM Ionomycin

#	Vol. (Int.)	Local Bg. Corr. Vol.	Area	Rf	Density	Local Bg. Corr. Den.	% band purity	% lane purity	Mol. Wt.	Rel. Quant. (w/ LB Corr. Vol.)
1	10,143,618	152,553	476	0.094	21,310	320.491	1.421	5.385	233.333	0.063
2	6,453,374	1,205,571	256	0.122	25,208	4,709.264	11.23	3.426	187.5	0.5
3	8,831,477	2,428,064	297	0.201	29,735	8,175.3	22.618	4.689	109.615	1.007
4	12,040,113	4,063,068	408	0.263	29,510	9,958.5	37.848	6.392	74.583	1.685
5	9,059,646	2,885,904	352	0.422	25,737	8,198.591	26.883	4.81	49.435	1.197

Lane 5 - 2uM Thapsigargin

#	Vol. (Int.)	Local Bg. Corr. Vol.	Area	Rf	Density	Local Bg. Corr. Den.	% band purity	% lane purity	Mol. Wt.	Rel. Quant. (w/ LB Corr. Vol.)
1	11,016,570	195,077	476	0.089	23,144	409.826	2.76	5.696	241.667	0.081

#	Vol. (Int.)	Local Bg. Corr. Vol.	Area	Rf	Density	Local Bg. Corr. Den.	% band purity	% lane purity	Mol. Wt.	Rel. Quant. (w/ LB Corr. Vol.)
2	7,181,413	1,257,290	264	0.117	27,202	4,762.465	17.788	3.713	195.833	0.522
3	9,618,435	1,960,843	340	0.193	28,289	5,767.186	27.741	4.973	115.385	0.813
4	9,376,257	2,247,866	324	0.255	28,939	6,937.859	31.802	4.848	77.778	0.932
5	18,797,325	471,300	888	0.294	21,168	530.744	6.668	9.72	69.583	0.195
6	9,650,687	935,912	432	0.417	22,339	2,166.464	13.241	4.99	50	0.388

Lane 7 - t = 0

#	Vol. (Int.)	Local Bg. Corr. Vol.	Area	Rf	Density	Local Bg. Corr. Den.	% band purity	% lane purity	Mol. Wt.	Rel. Quant. (w/ LB Corr. Vol.)
1	6,589,615	1,174,383	288	0.055	22,880	4,077.719	3.588	2.925	NA	0.487
2	10,576,430	228,533	476	0.083	22,219	480.112	0.698	4.695	NA	0.095
3	8,021,321	3,087,267	238	0.115	33,703	12,971	9.431	3.561	200	1.281
4	10,978,359	305,622	476	0.146	23,063	642.064	0.934	4.873	150	0.127
5	12,322,497	5,078,753	306	0.193	40,269	16,597	15.515	5.47	115.385	2.107
6	17,108,499	7,912,076	385	0.255	44,437	20,550	24.17	7.595	77.778	3.282
7	18,233,893	9,227,251	442	0.414	41,253	20,876	28.188	8.094	50.417	3.828
8	16,021,524	1,843,787	840	0.552	19,073	2,194.985	5.633	7.112	35.615	0.765
9	16,230,855	3,876,927	814	0.62	19,939	4,762.81	11.844	7.205	29.615	1.608

Lane 8 - Vehicle

#	Vol. (Int.)	Local Bg. Corr. Vol.	Area	Rf	Density	Local Bg. Corr. Den.	% band purity	% lane purity	Mol. Wt.	Rel. Quant. (w/ LB Corr. Vol.)
1	5,517,840	1,715,489	256	0.047	21,554	6,701.129	7.296	2.703	NA	0.712
2	6,134,798	575,654	320	0.081	19,171	1,798.919	2.448	3.005	NA	0.239
3	6,933,509	2,410,755	264	0.109	26,263	9,131.649	10.253	3.396	208.333	1
4	8,183,221	315,390	396	0.146	20,664	796.44	1.341	4.008	150	0.131
5	11,008,355	4,365,634	297	0.188	37,065	14,699	18.567	5.392	119.231	1.811
6	15,345,160	6,270,260	408	0.247	37,610	15,368	26.667	7.516	81.944	2.601
7	14,861,541	5,416,798	476	0.404	31,221	11,379	23.037	7.279	52.083	2.247
8	16,623,935	2,443,432	912	0.609	18,227	2,679.202	10.392	8.142	30.538	1.014

Lane 9 - 5uM Ionomycin

#	Vol. (Int.)	Local Bg. Corr. Vol.	Area	Rf	Density	Local Bg. Corr. Den.	% band purity	% lane purity	Mol. Wt.	Rel. Quant. (w/ LB Corr. Vol.)

#	Vol. (Int.)	Local Bg. Corr. Vol.	Area	Rf	Density	Local Bg. Corr. Den.	% band purity	% lane purity	Mol. Wt.	Rel. Quant. (w/ LB Corr. Vol.)
1	5,266,771	1,972,358	248	0.047	21,236	7,953.058	4.849	2.253	NA	0.818
2	6,427,640	1,081,725	320	0.078	20,086	3,380.392	2.659	2.75	NA	0.449
3	7,922,553	3,710,428	256	0.107	30,947	14,493	9.122	3.389	212.5	1.539
4	9,752,079	807,724	455	0.143	21,433	1,775.219	1.986	4.172	154.167	0.335
5	12,677,247	5,509,819	330	0.185	38,415	16,696	13.546	5.423	121.154	2.286
6	19,672,628	10,043,750	442	0.245	44,508	22,723	24.692	8.415	83.333	4.166
7	17,334,700	275,888	756	0.352	22,929	364.932	0.678	7.415	60.417	0.114
8	26,011,982	13,678,713	578	0.398	45,003	23,665	33.629	11.127	52.917	5.674
9	20,905,638	3,594,926	1,026	0.594	20,375	3,503.827	8.838	8.943	31.923	1.491

Lane 10 - 2uM Thapsigargin

#	Vol. (Int.)	Local Bg. Corr. Vol.	Area	Rf	Density	Local Bg. Corr. Den.	% band purity	% lane purity	Mol. Wt.	Rel. Quant. (w/ LB Corr. Vol.)
1	5,869,747	1,761,116	385	0.042	15,246	4,574.329	3.819	2.89	NA	0.731
2	6,610,398	893,823	462	0.076	14,308	1,934.684	1.938	3.255	NA	0.371
3	5,845,780	3,347,737	192	0.102	30,446	17,436	7.26	2.878	220.833	1.389
4	6,894,193	632,992	429	0.138	16,070	1,475.507	1.373	3.394	162.5	0.263
5	11,872,885	6,235,018	330	0.18	35,978	18,893	13.521	5.846	125	2.586
6	18,348,522	10,627,007	442	0.24	41,512	24,043	23.045	9.034	86.111	4.408
7	11,052,419	1,039,735	561	0.284	19,701	1,853.361	2.255	5.442	71.25	0.431
8	12,688,159	290,176	660	0.341	19,224	439.661	0.629	6.247	62.083	0.12
9	25,470,349	14,548,745	612	0.388	41,618	23,772	31.55	12.54	54.583	6.035
10	10,195,315	571,052	630	0.487	16,183	906.432	1.238	5.02	42.37	0.237
11	12,575,292	1,074,409	780	0.529	16,122	1,377.448	2.33	6.191	37.848	0.446
12	18,724,206	5,091,467	975	0.591	19,204	5,222.018	11.041	9.219	32.154	2.112
