# Supplementary material for: Elevation of Cytoplasmic Calcium Suppresses Microtentacle Formation and Function in Breast Tumor Cells
Source: Cancers (Basel). 2023 Jan 31;15(3):884. doi: 10.3390/cancers15030884 (PMC9913253; doi:10.3390/cancers15030884)
Supplement: Supplementary file 1 [file cancers-15-00884-s001.zip › cancers-2080686-Supplementary/File S1_ Original Blots/Original Immunoblot Images MDAMB231 Biological Replicate 3.pdf]

iBright™ Image Analysis Report

Katarina+ Chang

18 November 2022

CHEMI_03202022_134003

Date:	20 March 2022 01:40:03PM
Mode:	Chemi Blots
Notes:	
Model:	FL1500
Instrument name:	2462619090234
Serial No:	2462619090234
Firmware version:	1.6.0
iBA version:	5.0
Image size:	676px X 540px
Image area:	118.63mm X 94.91mm
Optical Zoom:	1.9x
Digital Zoom:	1x
Focus level:	430
Resolution:	5 x 5
Exposure time:	950 ms
Exposure mode:	Normal

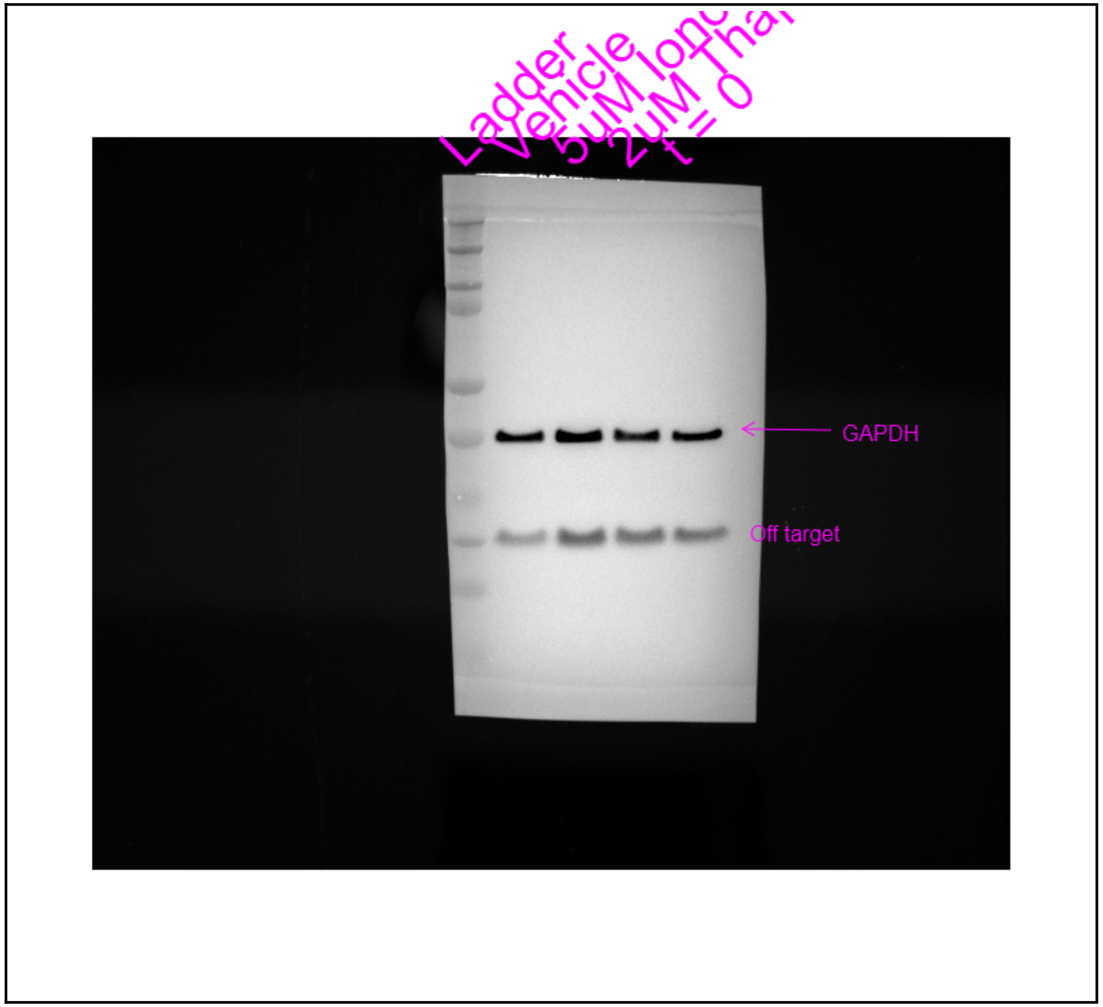

CHEMI_03202022_134003

Date:	20 March 2022 01:40:03PM
Mode:	Chemi Blots
Notes:	
Model:	FL1500
Instrument name:	2462619090234
Serial No:	2462619090234
Firmware version:	1.6.0
iBA version:	5.0
Image size:	676px X 540px
Image area:	118.63mm X 94.91mm
Optical Zoom:	1.9x
Digital Zoom:	1x
Focus level:	430
Resolution:	5 x 5
Exposure time:	950 ms
Exposure mode:	Normal

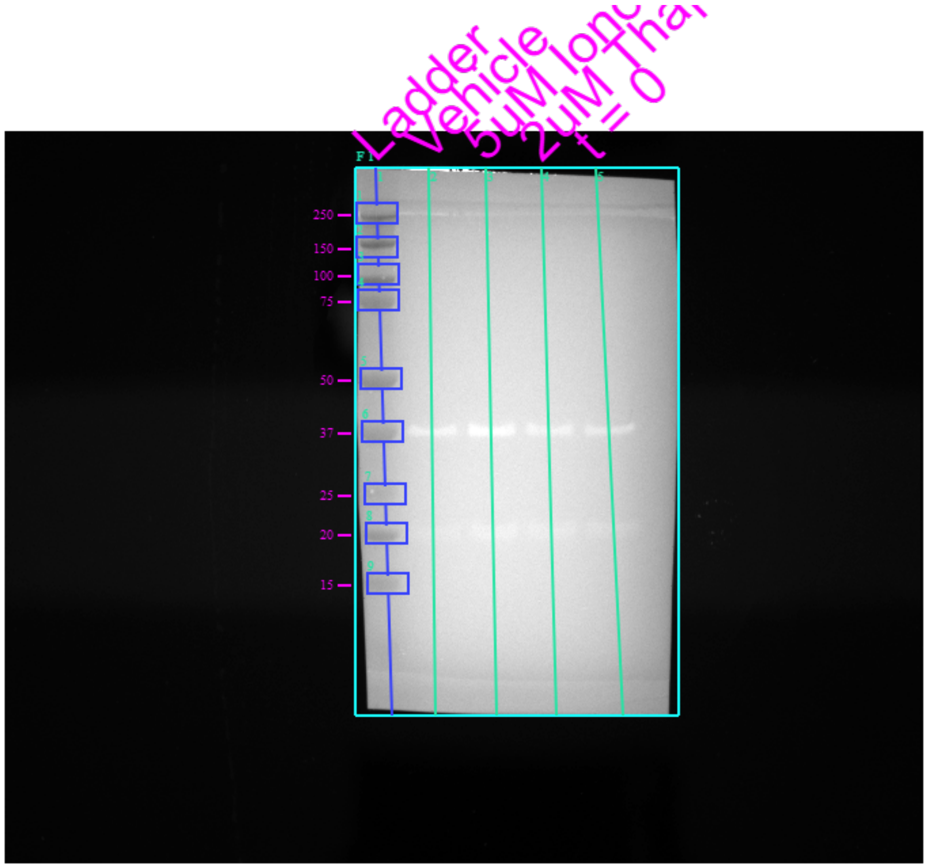

CHEMI_03202022_134003

Date:	20 March 2022 01:40:03PM
Mode:	Chemi Blots
Notes:	
Model:	FL1500
Instrument name:	2462619090234
Serial No:	2462619090234
Firmware version:	1.6.0
iBA version:	5.0
Image size:	676px X 540px
Image area:	118.63mm X 94.91mm
Optical Zoom:	1.9x
Digital Zoom:	1x
Focus level:	430
Resolution:	5 x 5
Exposure time:	950 ms
Exposure mode:	Normal

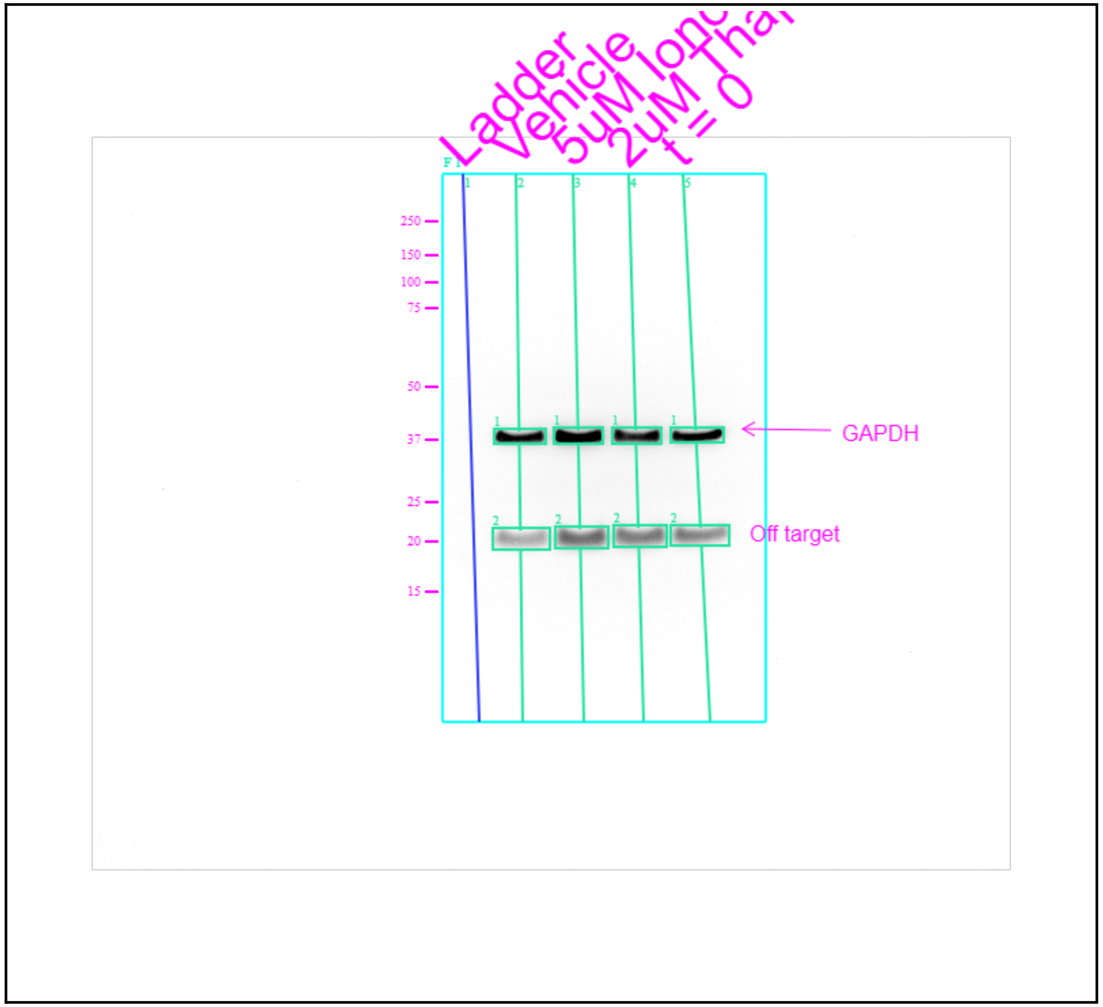

LANE AND BAND ANALYSIS DATA TABLE

CHEMI_03202022_134003

Frame: 1
Channel: Membrane
Sensitivity: 100
Molecular Weight Analysis Regression Method : Point to Point

Lane 1 - Ladder

#	Vol. (Int.)	Local Bg. Corr. Vol.	Area	Rf	Density	Local Bg. Corr. Den.	% band purity	% lane purity	Mol. Wt.
1	17,941,217	129,239	496	0.082	36,171	260.564	2.843	3.943	250
2	17,759,861	292,887	496	0.144	35,806	590.498	6.444	3.904	150
3	16,970,424	231,995	496	0.193	34,214	467.733	5.104	3.73	100
4	16,873,840	120,956	496	0.24	34,019	243.864	2.661	3.709	75
5	15,754,607	790,049	496	0.384	31,763	1,592.843	17.381	3.463	50
6	14,926,458	885,797	496	0.48	30,093	1,785.881	19.488	3.281	37
7	13,762,584	588,379	496	0.594	27,747	1,186.248	12.944	3.025	25
8	14,372,826	1,052,341	496	0.666	28,977	2,121.655	23.152	3.159	20
9	14,340,592	453,792	496	0.757	28,912	914.905	9.983	3.152	15

Frame: 1
Channel: Chemi
Sensitivity: 100
Molecular Weight Analysis Regression Method : Point to Point

Lane 2 - Vehicle

#	Vol. (Int.)	Local Bg. Corr. Vol.	Area	Rf	Density	Local Bg. Corr. Den.	% band purity	% lane purity	Mol. Wt.
1	9,655,023	8,465,892	468	0.478	20,630	18,089	68.911	49.764	37.333
2	4,979,969	3,819,432	731	0.666	6,812.543	5,224.942	31.089	25.668	20

Lane 3 - 5uM Ionomycin

#	Vol. (Int.)	Local Bg. Corr. Vol.	Area	Rf	Density	Local Bg. Corr. Den.	% band purity	% lane purity	Mol. Wt.
1	13,224,720	11,348,451	481	0.478	27,494	23,593	63.21	44.723	37.333
2	8,452,247	6,605,193	680	0.663	12,429	9,713.519	36.79	28.584	20.172

Lane 4 - 2uM Thapsigargin

#	Vol. (Int.)	Local Bg. Corr. Vol.	Area	Rf	Density	Local Bg. Corr. Den.	% band purity	% lane purity	Mol. Wt.
1	9,420,729	7,847,870	481	0.478	19,585	16,315	57.56	38.752	37.333
2	7,575,947	5,786,446	680	0.661	11,141	8,509.48	42.44	31.164	20.345

Lane 5 - t = 0

#	Vol. (Int.)	Local Bg. Corr. Vol.	Area	Rf	Density	Local Bg. Corr. Den.	% band purity	% lane purity	Mol. Wt.
1	10,431,543	9,182,270	480	0.475	21,732	19,129	62.965	47.728	37.667
2	6,654,743	5,400,964	704	0.658	9,452.76	7,671.825	37.035	30.448	20.517

iBright™ Image Analysis Report

Katarina+ Chang

18 November 2022

CHEMI_03182022_122312

Date:	18 March 2022 12:23:12PM
Mode:	Chemi Blots
Notes:	
Model:	FL1500
Instrument name:	2462619090234
Serial No:	2462619090234
Firmware version:	1.6.0
iBA version:	5.0
Image size:	563px X 450px
Image area:	112.7mm X 90.16mm
Optical Zoom:	2x
Digital Zoom:	1.2x
Focus level:	455
Resolution:	5 x 5
Exposure time:	807 ms
Exposure mode:	Normal

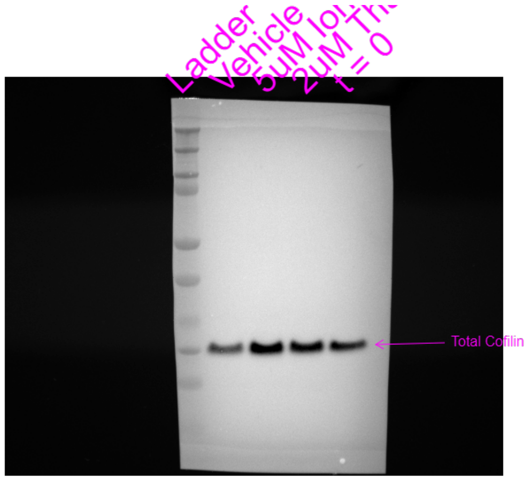

CHEMI_03182022_122312

Date:	18 March 2022 12:23:12PM
Mode:	Chemi Blots
Notes:	
Model:	FL1500
Instrument name:	2462619090234
Serial No:	2462619090234
Firmware version:	1.6.0
iBA version:	5.0
Image size:	563px X 450px
Image area:	112.7mm X 90.16mm
Optical Zoom:	2x
Digital Zoom:	1.2x
Focus level:	455
Resolution:	5 x 5
Label:	Ladder
Exposure time:	807 ms
Exposure mode:	Normal

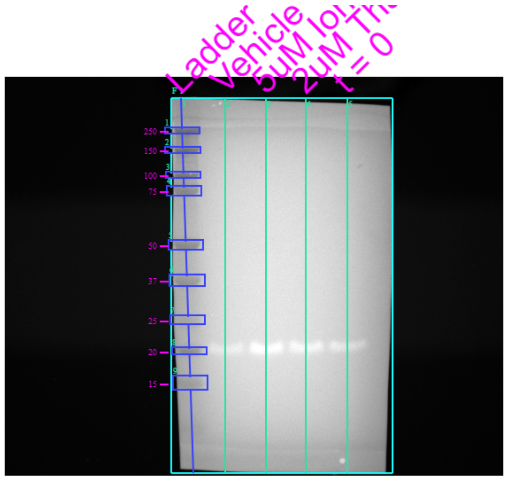

CHEMI_03182022_122312

Date:	18 March 2022 12:23:12PM
Mode:	Chemi Blots
Notes:	
Model:	FL1500
Instrument name:	2462619090234
Serial No:	2462619090234
Firmware version:	1.6.0
iBA version:	5.0
Image size:	563px X 450px
Image area:	112.7mm X 90.16mm
Optical Zoom:	2x
Digital Zoom:	1.2x
Focus level:	455
Resolution:	5 x 5
Exposure time:	807 ms
Exposure mode:	Normal

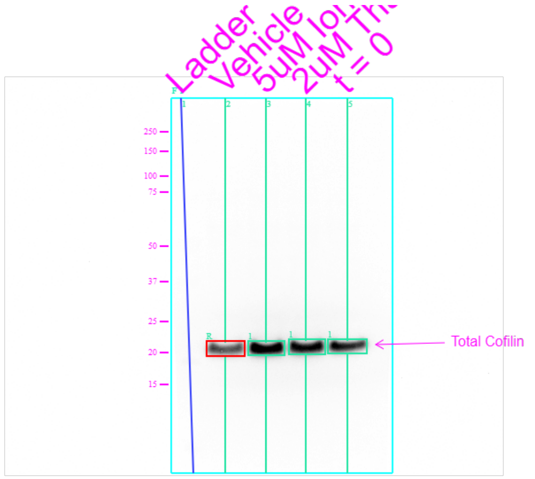

LANE AND BAND ANALYSIS DATA TABLE

CHEMI_03182022_122312

Frame: 1
Channel: Membrane
Sensitivity: 100
Molecular Weight Analysis Regression Method : Point to Point

Lane 1 - Ladder

#	Vol. (Int.)	Local Bg. Corr. Vol.	Area	Rf	Density	Local Bg. Corr. Den.	% band purity	% lane purity	Mol. Wt.
1	13,992,785	1,397,641	320	0.085	43,727	4,367.628	10.465	2.403	250
2	14,006,521	1,499,956	328	0.137	42,702	4,573.038	11.231	2.405	150
3	13,392,613	1,541,106	320	0.203	41,851	4,815.959	11.539	2.3	100
4	18,831,414	2,095,311	480	0.246	39,232	4,365.232	15.688	3.233	75
5	17,364,406	1,442,562	480	0.39	36,175	3,005.339	10.801	2.982	50
6	19,557,984	1,438,267	574	0.485	34,073	2,505.693	10.769	3.358	37
7	14,319,062	1,097,430	440	0.591	32,543	2,494.159	8.217	2.459	25
8	12,502,656	944,656	360	0.674	34,729	2,624.047	7.073	2.147	20
9	23,465,415	1,899,060	680	0.759	34,507	2,792.735	14.219	4.029	15

Frame: 1
Channel: Chemi
Sensitivity: 100
Molecular Weight Analysis Regression Method : Point to Point

Lane 2 - Vehicle

#	Vol. (Int.)	Local Bg. Corr. Vol.	Area	Rf	Density	Local Bg. Corr. Den.	% band purity	% lane purity	Mol. Wt.	Rel. Quant. (w/ LB Corr. Vol.)
1	14,837,679	11,642,366	792	0.667	18,734	14,699	100	58.793	20.429	1

Lane 3 - 5uM Ionomycin

#	Vol. (Int.)	Local Bg. Corr. Vol.	Area	Rf	Density	Local Bg. Corr. Den.	% band purity	% lane purity	Mol. Wt.	Rel. Quant. (w/ LB Corr. Vol.)
1	24,121,807	19,111,894	714	0.667	33,784	26,767	100	61.611	20.429	1.642

Lane 4 - 2uM Thapsigargin

#	Vol. (Int.)	Local Bg. Corr. Vol.	Area	Rf	Density	Local Bg. Corr. Den.	% band purity	% lane purity	Mol. Wt.	Rel. Quant. (w/ LB Corr. Vol.)

#	Vol. (Int.)	Local Bg. Corr. Vol.	Area	Rf	Density	Local Bg. Corr. Den.	% band purity	% lane purity	Mol. Wt.	Rel. Quant. (w/ LB Corr. Vol.)
1	21,483,808	16,617,118	756	0.662	28,417	21,980	100	59.984	20.714	1.427

Lane 5 - t = 0

#	Vol. (Int.)	Local Bg. Corr. Vol.	Area	Rf	Density	Local Bg. Corr. Den.	% band purity	% lane purity	Mol. Wt.	Rel. Quant. (w/ LB Corr. Vol.)
1	18,167,584	14,976,935	765	0.662	23,748	19,577	100	63.719	20.714	1.286

iBright™ Image Analysis Report

Katarina+ Chang

18 November 2022

CHEMI_03202022_142119

Date:	20 March 2022 02:21:19PM
Mode:	Chemi Blots
Notes:	
Model:	FL1500
Instrument name:	2462619090234
Serial No:	2462619090234
Firmware version:	1.6.0
iBA version:	5.0
Image size:	615px X 491px
Image area:	112.7mm X 90.16mm
Optical Zoom:	2x
Digital Zoom:	1.1x
Focus level:	455
Resolution:	5 x 5
Exposure time:	60000 ms
Exposure mode:	Normal

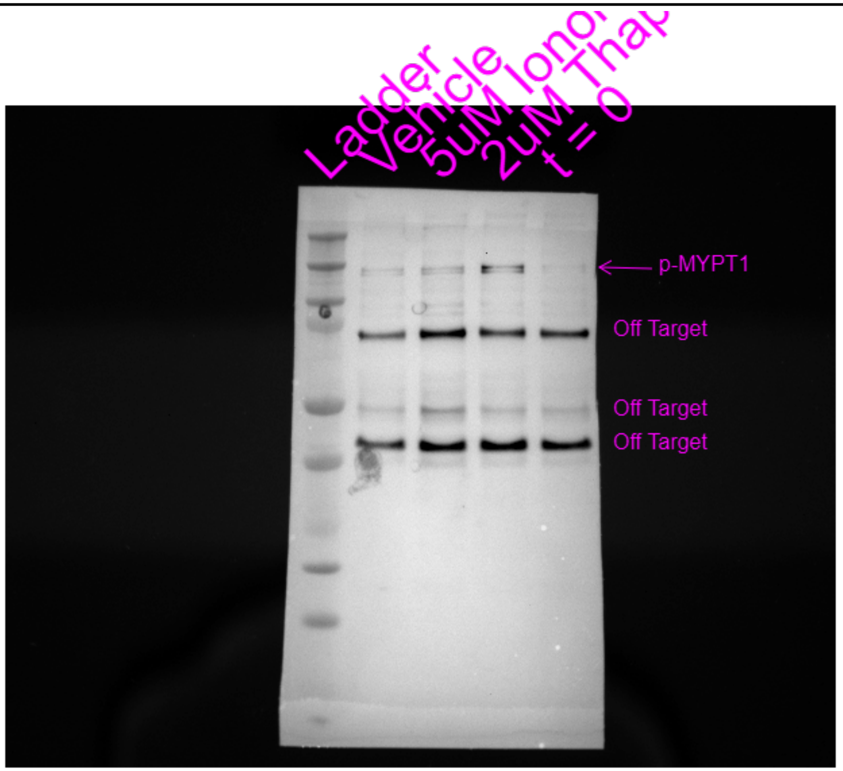

CHEMI_03202022_142119

Date:	20 March 2022 02:21:19PM
Mode:	Chemi Blots
Notes:	
Model:	FL1500
Instrument name:	2462619090234
Serial No:	2462619090234
Firmware version:	1.6.0
iBA version:	5.0
Image size:	615px X 491px
Image area:	112.7mm X 90.16mm
Optical Zoom:	2x
Digital Zoom:	1.1x
Focus level:	455
Resolution:	5 x 5
Exposure time:	60000 ms
Exposure mode:	Normal

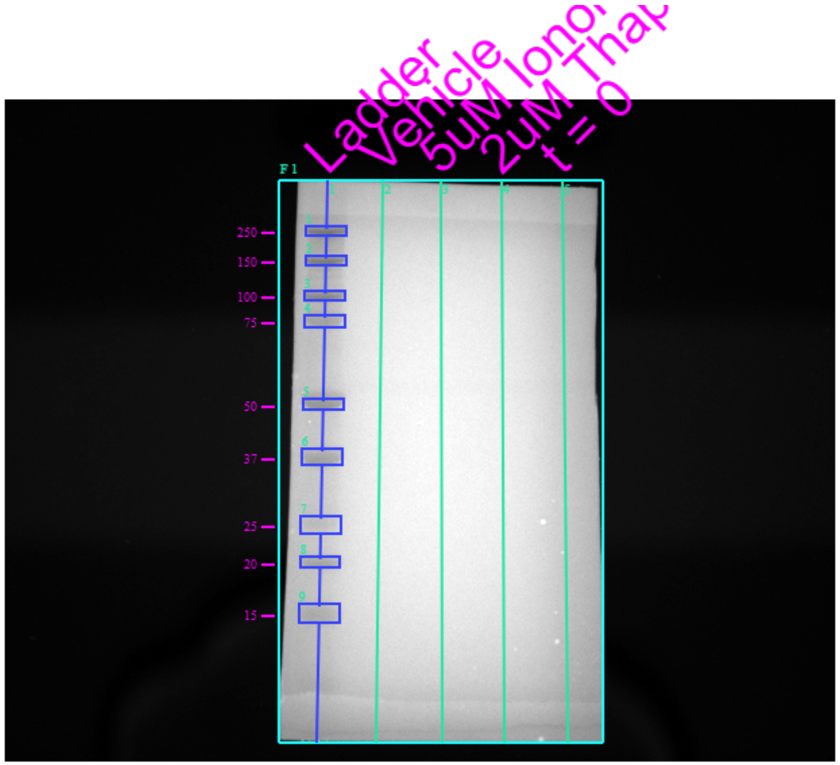

CHEMI_03202022_142119

Date:	20 March 2022 02:21:19PM
Mode:	Chemi Blots
Notes:	
Model:	FL1500
Instrument name:	2462619090234
Serial No:	2462619090234
Firmware version:	1.6.0
iBA version:	5.0
Image size:	615px X 491px
Image area:	112.7mm X 90.16mm
Optical Zoom:	2x
Digital Zoom:	1.1x
Focus level:	455
Resolution:	5 x 5
Exposure time:	60000 ms
Exposure mode:	Normal

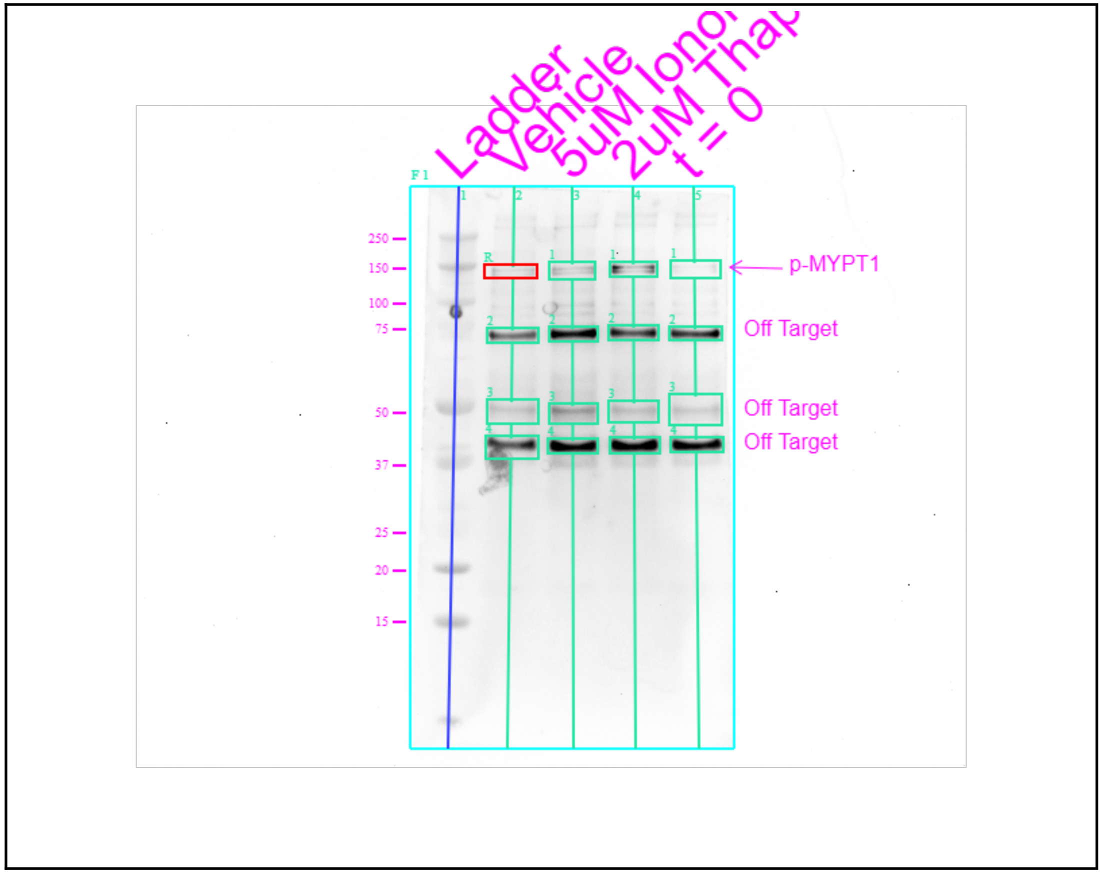

LANE AND BAND ANALYSIS DATA TABLE

CHEMI_03202022_142119

Frame: 1
Channel: Membrane
Sensitivity: 100
Molecular Weight Analysis Regression Method : Point to Point

Lane 1 - Ladder

#	Vol. (Int.)	Local Bg. Corr. Vol.	Area	Rf	Density	Local Bg. Corr. Den.	% band purity	% lane purity	Mol. Wt.
1	9,351,735	1,516,014	248	0.089	37,708	6,112.963	12.048	2.499	250
2	8,933,278	1,619,744	248	0.141	36,021	6,531.226	12.873	2.387	150
3	8,614,170	1,595,864	248	0.204	34,734	6,434.936	12.683	2.302	100
4	9,627,045	1,195,430	310	0.249	31,054	3,856.227	9.5	2.572	75
5	9,045,968	1,485,943	279	0.398	32,422	5,325.963	11.809	2.417	50
6	12,375,038	1,986,917	403	0.492	30,707	4,930.317	15.791	3.306	37
7	11,828,897	735,312	434	0.612	27,255	1,694.269	5.844	3.16	25
8	8,315,726	1,117,259	270	0.679	30,798	4,137.997	8.879	2.222	20
9	14,078,962	1,330,435	465	0.77	30,277	2,861.152	10.573	3.762	15

Frame: 1
Channel: Chemi
Sensitivity: 100
Molecular Weight Analysis Regression Method : Point to Point

Lane 2 - Vehicle

#	Vol. (Int.)	Local Bg. Corr. Vol.	Area	Rf	Density	Local Bg. Corr. Den.	% band purity	% lane purity	Mol. Wt.	Rel. Quant. (w/ LB Corr. Vol.)
1	3,177,157	1,128,224	440	0.151	7,220.811	2,564.147	5.392	3.959	142.308	1
2	9,493,799	6,563,133	468	0.264	20,285	14,023	31.364	11.829	72.581	5.817
3	7,605,219	2,553,638	741	0.4	10,263	3,446.205	12.203	9.476	49.667	2.263
4	17,754,097	10,680,915	720	0.463	24,658	14,834	51.042	22.121	41	9.467

Lane 3 - 5uM Ionomycin

#	Vol. (Int.)	Local Bg. Corr. Vol.	Area	Rf	Density	Local Bg. Corr. Den.	% band purity	% lane purity	Mol. Wt.	Rel. Quant. (w/ LB Corr. Vol.)
1	4,831,623	1,974,467	490	0.149	9,860.455	4,029.525	6.383	4.848	144.231	1.75

#	Vol. (Int.)	Local Bg. Corr. Vol.	Area	Rf	Density	Local Bg. Corr. Den.	% band purity	% lane purity	Mol. Wt.	Rel. Quant. (w/ LB Corr. Vol.)
2	15,708,899	11,623,962	444	0.261	35,380	26,180	37.575	15.762	72.984	10.303
3	10,142,419	4,572,022	592	0.403	17,132	7,723.01	14.779	10.177	49.333	4.052
4	18,071,331	12,764,527	456	0.46	39,630	27,992	41.262	18.133	41.333	11.314

Lane 4 - 2uM Thapsigargin

#	Vol. (Int.)	Local Bg. Corr. Vol.	Area	Rf	Density	Local Bg. Corr. Den.	% band purity	% lane purity	Mol. Wt.	Rel. Quant. (w/ LB Corr. Vol.)
1	6,156,013	4,117,013	432	0.146	14,250	9,530.124	14.052	6.781	146.154	3.649
2	12,074,732	8,563,694	456	0.259	26,479	18,780	29.23	13.301	73.387	7.59
3	7,615,189	2,635,162	646	0.4	11,788	4,079.199	8.994	8.388	49.667	2.336
4	18,954,675	13,982,131	494	0.46	38,369	28,303	47.724	20.879	41.333	12.393

Lane 5 - t = 0

#	Vol. (Int.)	Local Bg. Corr. Vol.	Area	Rf	Density	Local Bg. Corr. Den.	% band purity	% lane purity	Mol. Wt.	Rel. Quant. (w/ LB Corr. Vol.)
1	1,842,719	471,286	532	0.146	3,463.758	885.878	1.782	3.039	146.154	0.418
2	11,800,291	9,796,257	440	0.261	26,818	22,264	37.051	19.458	72.984	8.683
3	7,569,011	2,693,523	960	0.396	7,884.386	2,805.753	10.187	12.481	50.403	2.387
4	16,826,043	13,478,984	480	0.458	35,054	28,081	50.979	27.746	41.667	11.947

iBright™ Image Analysis Report

Katarina+ Chang

18 November 2022

CHEMI_03222022_123455

Date:	22 March 2022 12:34:55PM
Mode:	Chemi Blots
Notes:	
Model:	FL1500
Instrument name:	2462619090234
Serial No:	2462619090234
Firmware version:	1.6.0
iBA version:	5.0
Image size:	615px X 491px
Image area:	112.7mm X 90.16mm
Optical Zoom:	2x
Digital Zoom:	1.1x
Focus level:	455
Resolution:	5 x 5
Exposure time:	8983 ms
Exposure mode:	Normal

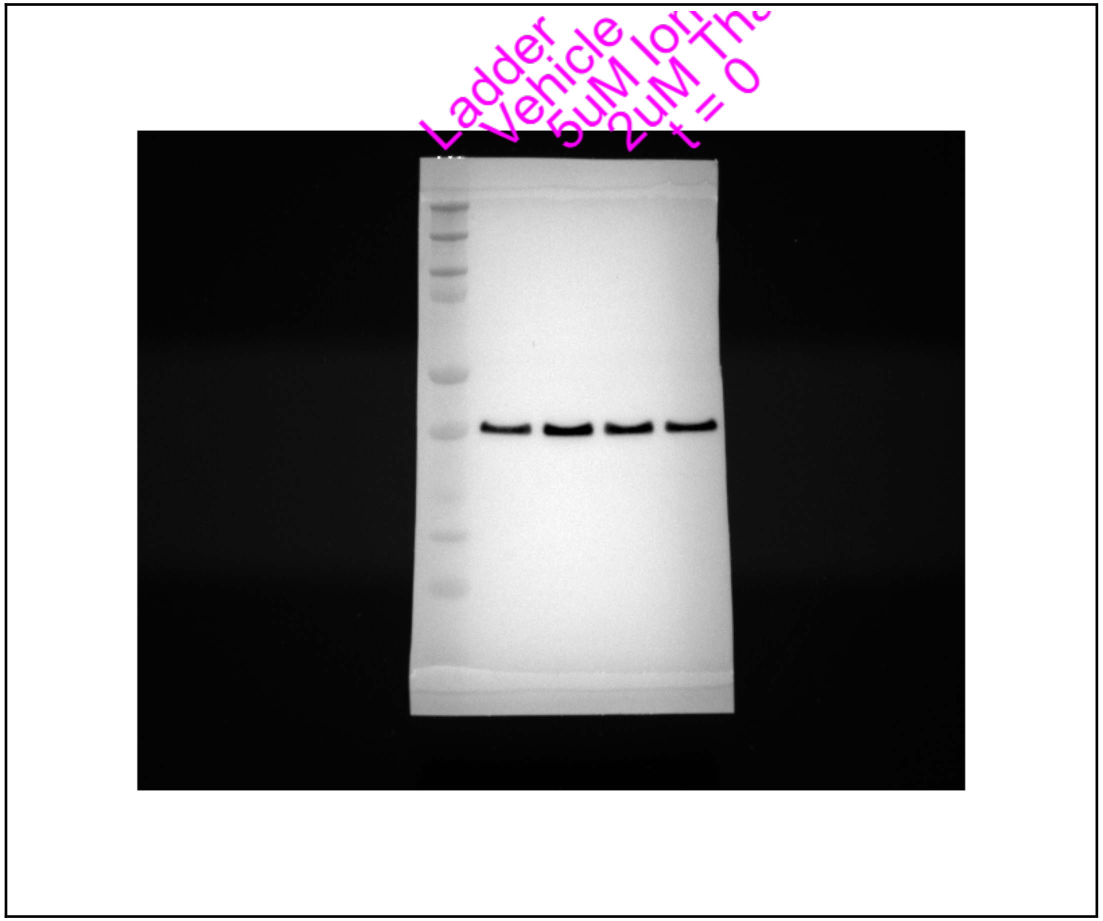

CHEMI_03222022_123455

Date: 22 March 2022 12:34:55PM
Mode: Chemi Blots
Notes:
Model: FL1500
Instrument name: 2462619090234
Serial No: 2462619090234
Firmware version: 1.6.0
iBA version: 5.0
Image size: 615px X 491px
Image area: 112.7mm X 90.16mm
Optical Zoom: 2x
Digital Zoom: 1.1x
Focus level: 455
Resolution: 5 x 5
Exposure time: 8983 ms
Exposure mode: Normal

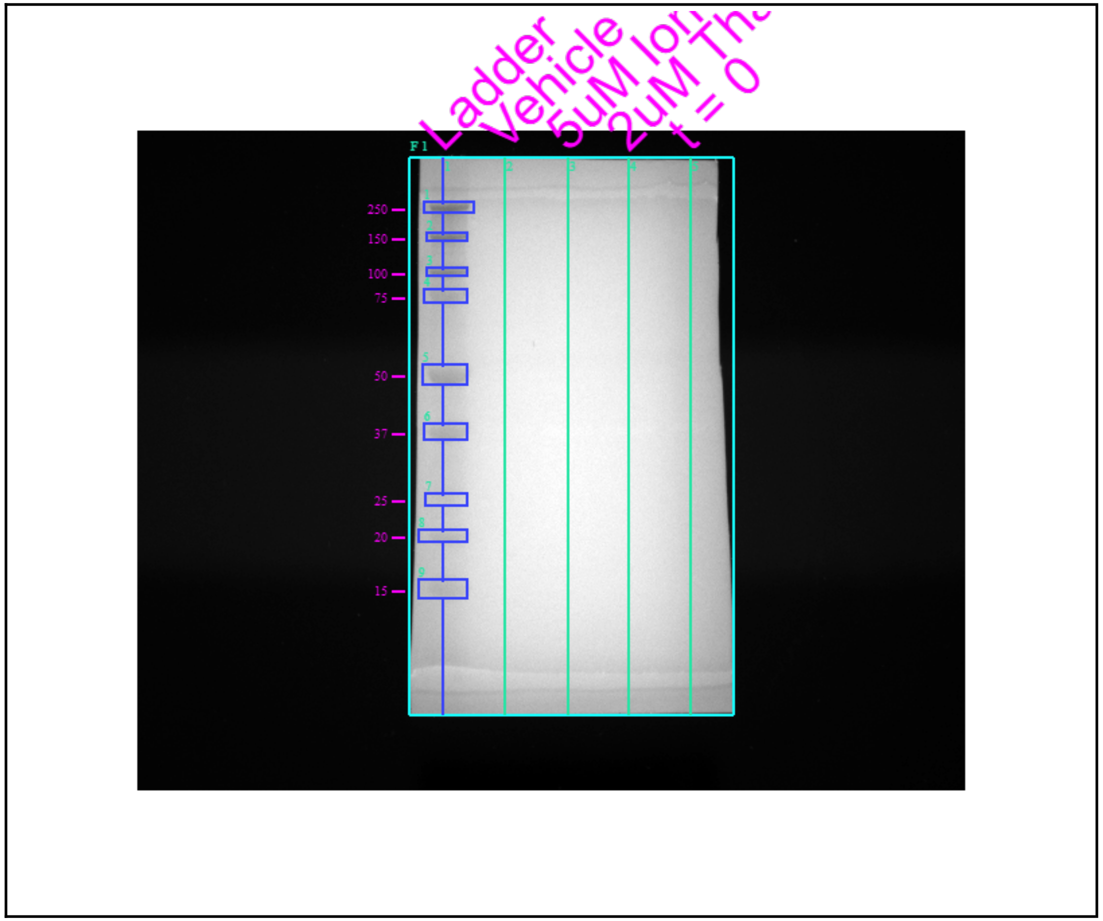

CHEMI_03222022_123455

Date:	22 March 2022 12:34:55PM
Mode:	Chemi Blots
Notes:	
Model:	FL1500
Instrument name:	2462619090234
Serial No:	2462619090234
Firmware version:	1.6.0
iBA version:	5.0
Image size:	615px X 491px
Image area:	112.7mm X 90.16mm
Optical Zoom:	2x
Digital Zoom:	1.1x
Focus level:	455
Resolution:	5 x 5
Exposure time:	8983 ms
Exposure mode:	Normal

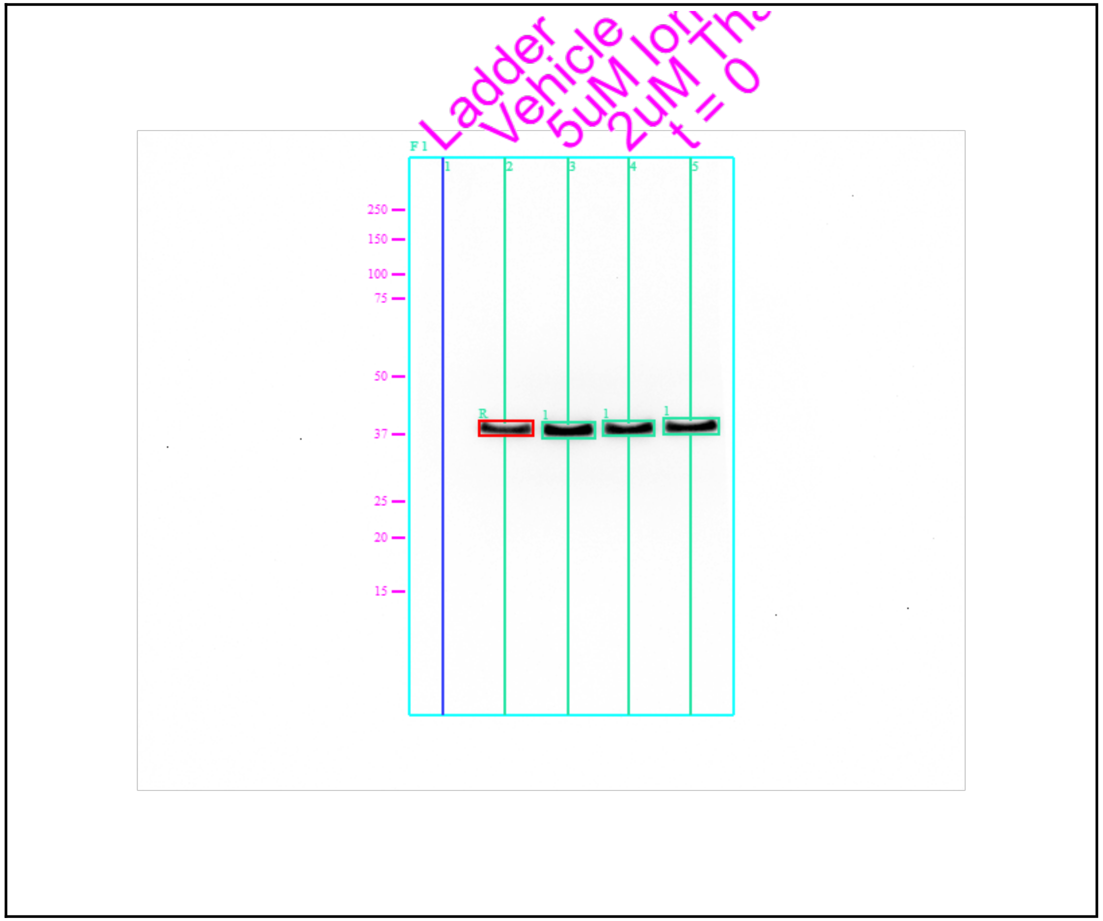

LANE AND BAND ANALYSIS DATA TABLE

CHEMI_03222022_123455

Frame: 1
Channel: Membrane
Sensitivity: 100
Molecular Weight Analysis Regression Method : Point to Point

Lane 1 - Ladder

#	Vol. (Int.)	Local Bg. Corr. Vol.	Area	Rf	Density	Local Bg. Corr. Den.	% band purity	% lane purity	Mol. Wt.
1	12,660,561	1,479,834	342	0.089	37,019	4,327.002	17.558	2.738	250
2	8,176,095	1,322,130	217	0.142	37,677	6,092.767	15.686	1.768	150
3	7,820,664	1,238,482	217	0.205	36,039	5,707.291	14.694	1.691	100
4	11,531,159	696,059	363	0.248	31,766	1,917.519	8.258	2.494	75
5	16,736,675	1,254,092	544	0.388	30,765	2,305.317	14.879	3.619	50
6	12,792,965	1,391,057	429	0.492	29,820	3,242.558	16.504	2.766	37
7	8,724,359	428,194	320	0.612	27,263	1,338.108	5.08	1.887	25
8	10,681,049	104,248	370	0.677	28,867	281.752	1.237	2.31	20
9	16,362,160	514,376	555	0.773	29,481	926.804	6.103	3.538	15

Frame: 1
Channel: Chemi
Sensitivity: 100
Molecular Weight Analysis Regression Method : Point to Point

Lane 2 - Vehicle

#	Vol. (Int.)	Local Bg. Corr. Vol.	Area	Rf	Density	Local Bg. Corr. Den.	% band purity	% lane purity	Mol. Wt.	Rel. Quant. (w/ LB Corr. Vol.)
1	11,651,286	10,096,994	492	0.484	23,681	20,522	100	58.447	37.907	1

Lane 3 - 5uM Ionomycin

#	Vol. (Int.)	Local Bg. Corr. Vol.	Area	Rf	Density	Local Bg. Corr. Den.	% band purity	% lane purity	Mol. Wt.	Rel. Quant. (w/ LB Corr. Vol.)
1	16,262,922	14,038,060	520	0.489	31,274	26,996	100	61.768	37.302	1.39

Lane 4 - 2uM Thapsigargin

#	Vol. (Int.)	Local Bg. Corr. Vol.	Area	Rf	Density	Local Bg. Corr. Den.	% band purity	% lane purity	Mol. Wt.	Rel. Quant. (w/ LB Corr. Vol.)

#	Vol. (Int.)	Local Bg. Corr. Vol.	Area	Rf	Density	Local Bg. Corr. Den.	% band purity	% lane purity	Mol. Wt.	Rel. Quant. (w/ LB Corr. Vol.)
1	13,670,386	11,773,814	468	0.484	29,210	25,157	100	57.581	37.907	1.166

Lane 5 - t = 0

#	Vol. (Int.)	Local Bg. Corr. Vol.	Area	Rf	Density	Local Bg. Corr. Den.	% band purity	% lane purity	Mol. Wt.	Rel. Quant. (w/ LB Corr. Vol.)
1	13,975,887	12,592,519	546	0.482	25,596	23,063	100	63.521	38.209	1.247

iBright™ Image Analysis Report

Katarina+ Chang

18 November 2022

CHEMI_03212022_132658

Date:	21 March 2022 01:26:58PM
Mode:	Chemi Blots
Notes:	
Model:	FL1500
Instrument name:	2462619090234
Serial No:	2462619090234
Firmware version:	1.6.0
iBA version:	5.0
Image size:	676px X 540px
Image area:	112.7mm X 90.16mm
Optical Zoom:	2x
Digital Zoom:	1x
Focus level:	455
Resolution:	5 x 5
Exposure time:	3258 ms
Exposure mode:	Normal

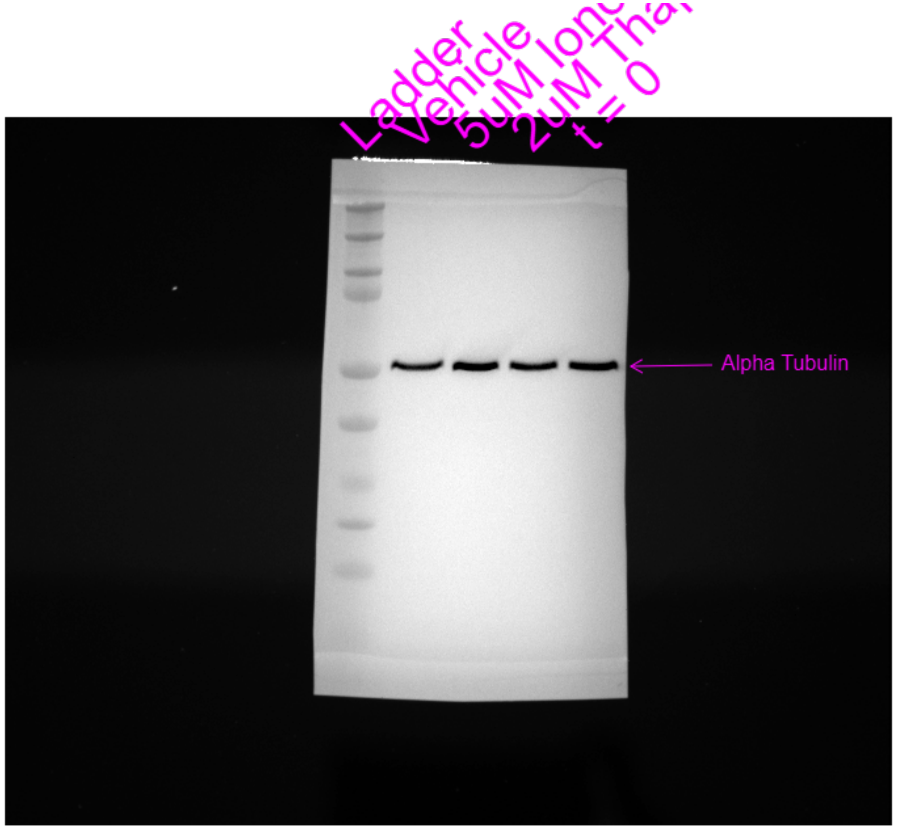

CHEMI_03212022_132658

Date: 21 March 2022 01:26:58PM
Mode: Chemi Blots
Notes:
Model: FL1500
Instrument name: 2462619090234
Serial No: 2462619090234
Firmware version: 1.6.0
iBA version: 5.0
Image size: 676px X 540px
Image area: 112.7mm X 90.16mm
Optical Zoom: 2x
Digital Zoom: 1x
Focus level: 455
Resolution: 5 x 5
Exposure time: 3258 ms
Exposure mode: Normal

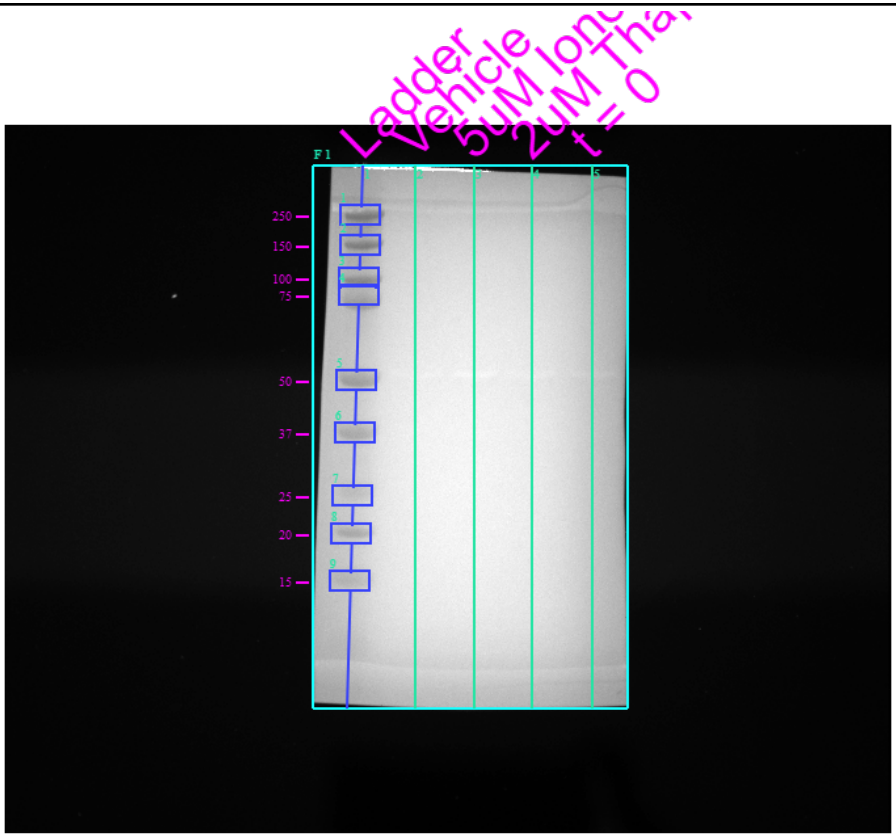

CHEMI_03212022_132658

Date:	21 March 2022 01:26:58PM
Mode:	Chemi Blots
Notes:	
Model:	FL1500
Instrument name:	2462619090234
Serial No:	2462619090234
Firmware version:	1.6.0
iBA version:	5.0
Image size:	676px X 540px
Image area:	112.7mm X 90.16mm
Optical Zoom:	2x
Digital Zoom:	1x
Focus level:	455
Resolution:	5 x 5
Exposure time:	3258 ms
Exposure mode:	Normal

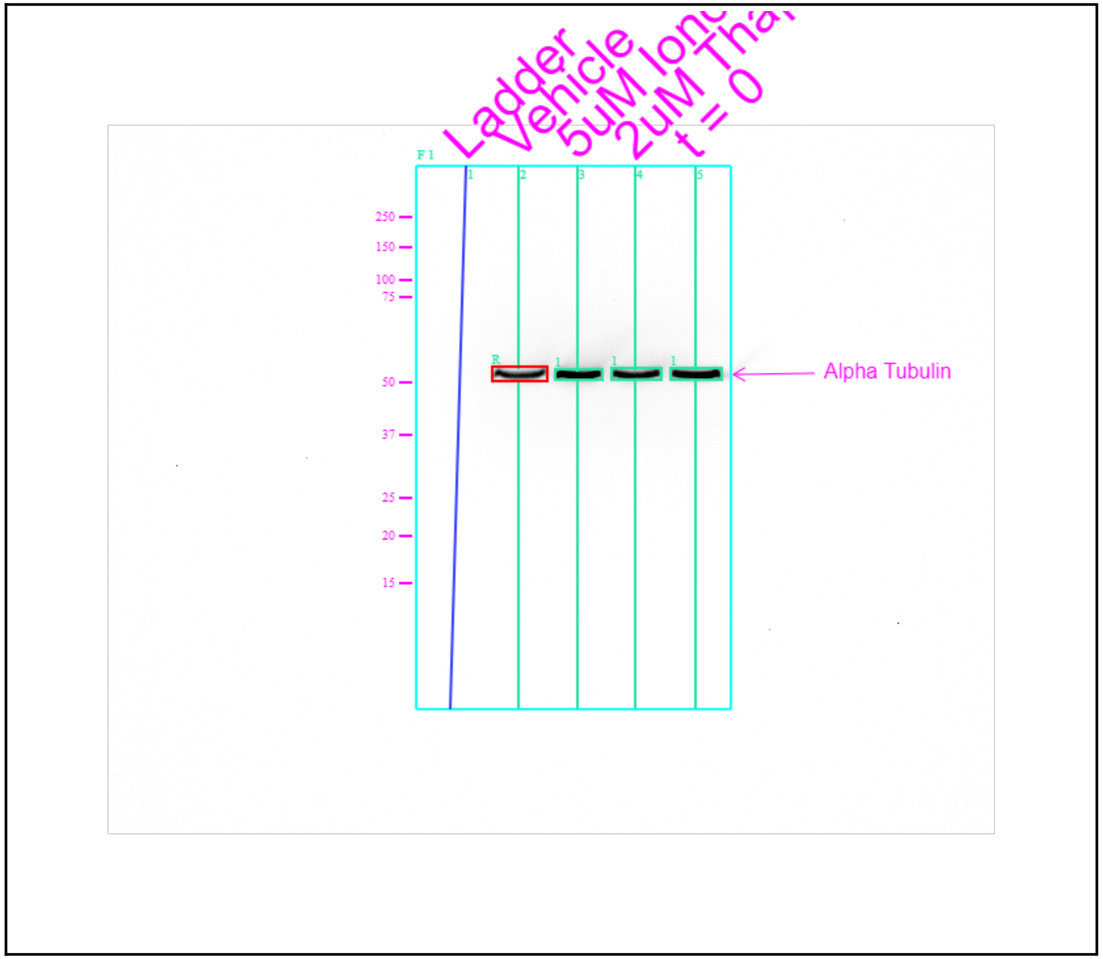

LANE AND BAND ANALYSIS DATA TABLE

CHEMI_03212022_132658

Frame: 1
Channel: Membrane
Sensitivity: 100
Molecular Weight Analysis Regression Method : Point to Point

Lane 1 - Ladder

#	Vol. (Int.)	Local Bg. Corr. Vol.	Area	Rf	Density	Local Bg. Corr. Den.	% band purity	% lane purity	Mol. Wt.
1	17,675,981	1,685,249	496	0.089	35,637	3,397.681	12.736	4.043	250
2	17,086,002	1,848,380	496	0.145	34,447	3,726.574	13.969	3.908	150
3	16,257,147	1,649,777	496	0.205	32,776	3,326.164	12.468	3.719	100
4	15,771,155	674,572	496	0.237	31,796	1,360.024	5.098	3.607	75
5	15,133,205	2,178,602	496	0.394	30,510	4,392.343	16.465	3.462	50
6	14,367,988	1,828,547	496	0.49	28,967	3,686.588	13.819	3.286	37
7	13,364,840	833,290	496	0.606	26,945	1,680.022	6.298	3.057	25
8	14,033,229	1,392,082	496	0.676	28,292	2,806.618	10.521	3.21	20
9	14,125,248	1,141,205	496	0.763	28,478	2,300.817	8.625	3.231	15

Frame: 1
Channel: Chemi
Sensitivity: 100
Molecular Weight Analysis Regression Method : Point to Point

Lane 2 - Vehicle

#	Vol. (Int.)	Local Bg. Corr. Vol.	Area	Rf	Density	Local Bg. Corr. Den.	% band purity	% lane purity	Mol. Wt.	Rel. Quant. (w/ LB Corr. Vol.)
1	5,792,534	4,974,522	516	0.382	11,225	9,640.547	100	67.718	51.923	1

Lane 3 - 5uM Ionomycin

#	Vol. (Int.)	Local Bg. Corr. Vol.	Area	Rf	Density	Local Bg. Corr. Den.	% band purity	% lane purity	Mol. Wt.	Rel. Quant. (w/ LB Corr. Vol.)
1	7,965,401	6,930,366	333	0.384	23,920	20,811	100	60.842	51.538	1.393

Lane 4 - 2uM Thapsigargin

#	Vol. (Int.)	Local Bg. Corr. Vol.	Area	Rf	Density	Local Bg. Corr. Den.	% band purity	% lane purity	Mol. Wt.	Rel. Quant. (w/ LB Corr. Vol.)

#	Vol. (Int.)	Local Bg. Corr. Vol.	Area	Rf	Density	Local Bg. Corr. Den.	% band purity	% lane purity	Mol. Wt.	Rel. Quant. (w/ LB Corr. Vol.)
1	6,072,075	5,139,623	390	0.382	15,569	13,178	100	57.329	51.923	1.033

Lane 5 - t = 0

#	Vol. (Int.)	Local Bg. Corr. Vol.	Area	Rf	Density	Local Bg. Corr. Den.	% band purity	% lane purity	Mol. Wt.	Rel. Quant. (w/ LB Corr. Vol.)
1	7,680,386	6,932,991	400	0.382	19,200	17,332	100	70.261	51.923	1.394

iBright™ Image Analysis Report

Katarina+ Chang

18 November 2022

CHEMI_03222022_124428

Date: 22 March 2022 12:44:28PM
Mode: Chemi Blots
Notes:
Model: FL1500
Instrument name: 2462619090234
Serial No: 2462619090234
Firmware version: 1.6.0
iBA version: 5.0
Image size: 615px X 491px
Image area: 112.7mm X 90.16mm
Optical Zoom: 2x
Digital Zoom: 1.1x
Focus level: 455
Resolution: 5 x 5
Exposure time: 30554 ms
Exposure mode: Normal

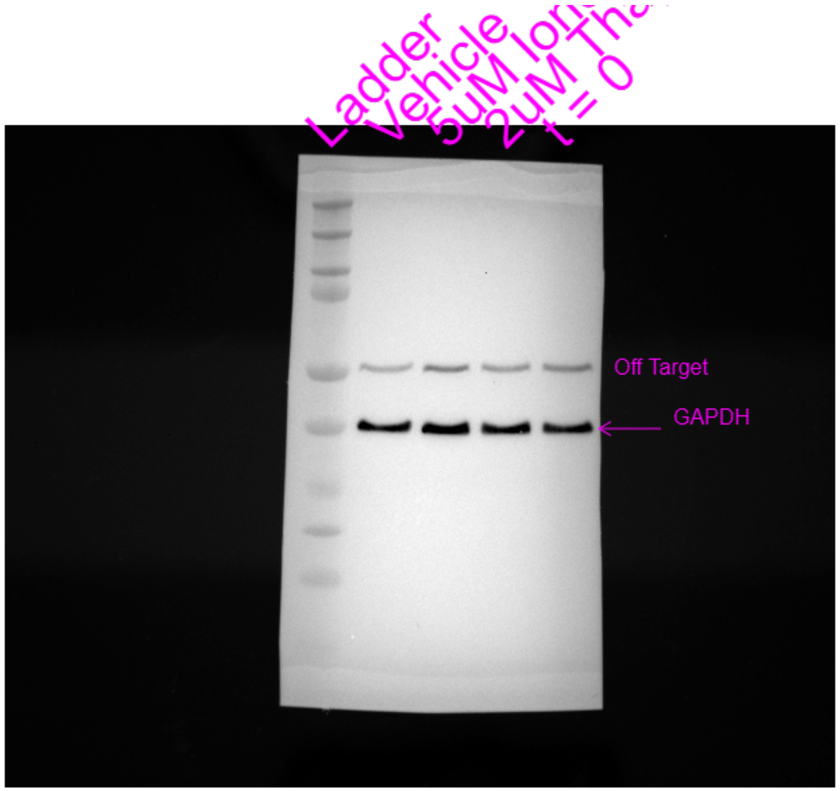

CHEMI_03222022_124428

Date:	22 March 2022 12:44:28PM
Mode:	Chemi Blots
Notes:	
Model:	FL1500
Instrument name:	2462619090234
Serial No:	2462619090234
Firmware version:	1.6.0
iBA version:	5.0
Image size:	615px X 491px
Image area:	112.7mm X 90.16mm
Optical Zoom:	2x
Digital Zoom:	1.1x
Focus level:	455
Resolution:	5 x 5
Exposure time:	30554 ms
Exposure mode:	Normal

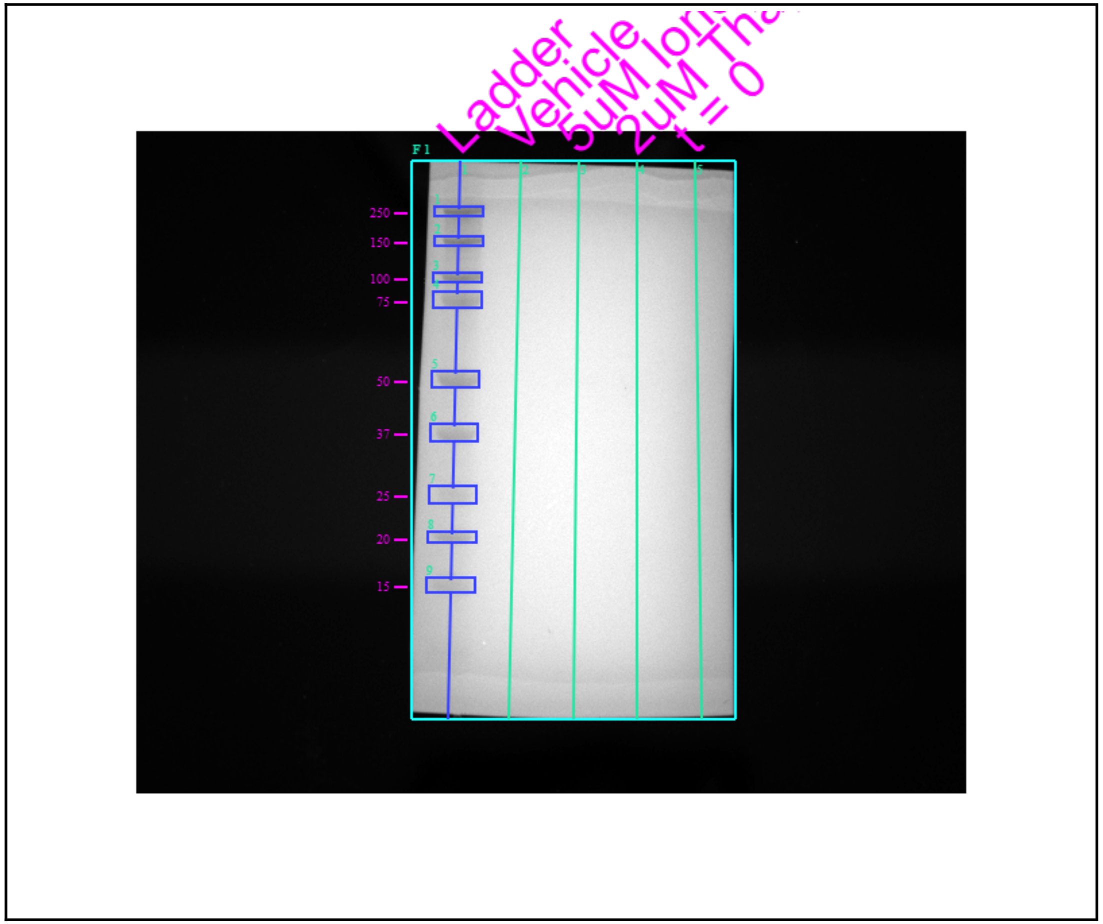

CHEMI_03222022_124428

Date:	22 March 2022 12:44:28PM
Mode:	Chemi Blots
Notes:	
Model:	FL1500
Instrument name:	2462619090234
Serial No:	2462619090234
Firmware version:	1.6.0
iBA version:	5.0
Image size:	615px X 491px
Image area:	112.7mm X 90.16mm
Optical Zoom:	2x
Digital Zoom:	1.1x
Focus level:	455
Resolution:	5 x 5
Exposure time:	30554 ms
Exposure mode:	Normal

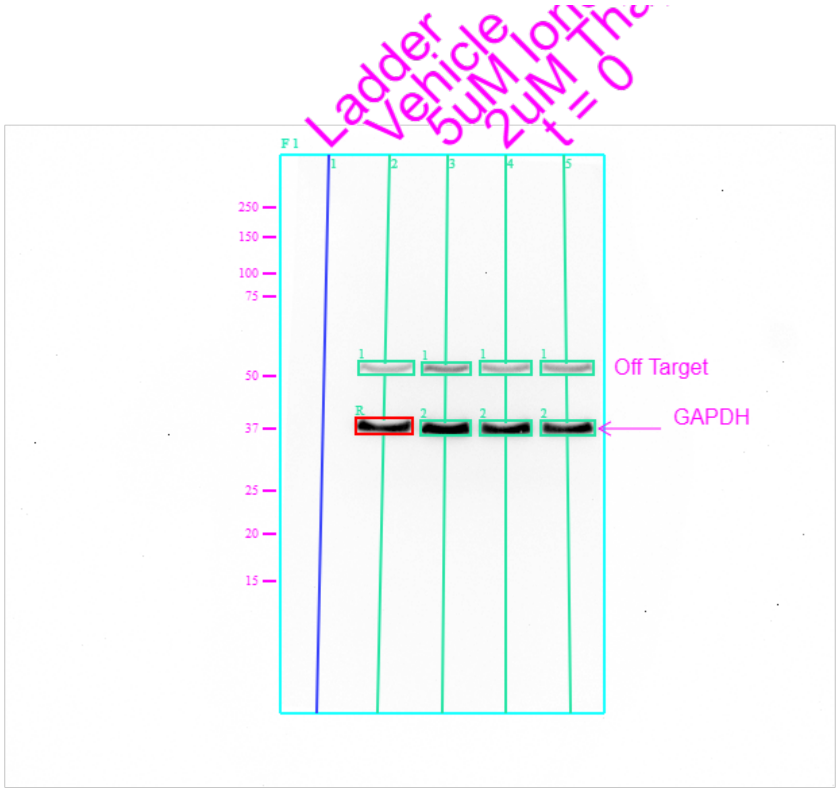

LANE AND BAND ANALYSIS DATA TABLE

CHEMI_03222022_124428

Frame: 1
Channel: Membrane
Sensitivity: 100
Molecular Weight Analysis Regression Method : Point to Point

Lane 1 - Ladder

#	Vol. (Int.)	Local Bg. Corr. Vol.	Area	Rf	Density	Local Bg. Corr. Den.	% band purity	% lane purity	Rolling Bg. Corr. Vol.	Rolling Bg. Corr. Den.	Mol. Wt.
1	11,111,944	1,325,331	296	0.089	37,540	4,477.472	13.633	3.17	1,843,456	6,227.892	250
2	10,494,368	1,412,436	296	0.143	35,453	4,771.744	9.745	2.266	1,317,632	4,451.459	150
3	9,997,372	1,404,362	296	0.208	33,774	4,744.467	9.837	2.287	1,330,176	4,493.838	100
4	15,011,343	1,709,133	481	0.249	31,208	3,553.292	11.011	2.56	1,488,896	3,095.418	75
5	14,124,866	2,193,210	468	0.391	30,181	4,686.346	16.557	3.849	2,238,720	4,783.59	50
6	14,371,951	1,927,530	504	0.486	28,515	3,824.464	14.33	3.332	1,937,664	3,844.571	37
7	13,438,651	993,061	504	0.597	26,663	1,970.36	6.738	1.567	911,104	1,807.746	25
8	9,549,346	1,175,156	333	0.674	28,676	3,528.999	9.313	2.165	1,259,264	3,781.574	20
9	12,555,256	1,069,660	444	0.758	28,277	2,409.144	8.836	2.054	1,194,752	2,690.883	15

Frame: 1
Channel: Chemi
Sensitivity: 100
Molecular Weight Analysis Regression Method : Point to Point

Lane 2 - Vehicle

#	Vol. (Int.)	Local Bg. Corr. Vol.	Area	Rf	Density	Local Bg. Corr. Den.
1	2,905,568	2,021,041	462	0.382	6,289.108	4,374.548
2	12,124,621	10,395,346	559	0.486	21,689	18,596

#	% band purity	% lane purity	Rolling Bg. Corr. Vol.	Rolling Bg. Corr. Den.	Mol. Wt.	Rel. Quant. (w/ LB Corr. Vol.)
1	16.121	15.053	2,109,952	4,566.996	51.695	0.194
2	83.879	78.321	10,978,304	19,639	37	1

Lane 3 - 5uM Ionomycin

#	Vol. (Int.)	Local Bg. Corr. Vol.	Area	Rf	Density	Local Bg. Corr. Den.

#	Vol. (Int.)	Local Bg. Corr. Vol.	Area	Rf	Density	Local Bg. Corr. Den.
1	3,845,016	2,936,790	370	0.382	10,391	7,937.27
2	14,113,627	12,069,193	456	0.488	30,950	26,467

#	% band purity	% lane purity	Rolling Bg. Corr. Vol.	Rolling Bg. Corr. Den.	Mol. Wt.	Rel. Quant. (w/ LB Corr. Vol.)
1	19.126	16.437	3,088,896	8,348.368	51.695	0.283
2	80.874	69.505	13,061,632	28,643	36.739	1.161

Lane 4 - 2uM Thapsigargin

#	Vol. (Int.)	Local Bg. Corr. Vol.	Area	Rf	Density	Local Bg. Corr. Den.
1	3,290,486	2,345,565	429	0.382	7,670.131	5,467.519
2	11,983,128	10,149,395	468	0.488	25,604	21,686

#	% band purity	% lane purity	Rolling Bg. Corr. Vol.	Rolling Bg. Corr. Den.	Mol. Wt.	Rel. Quant. (w/ LB Corr. Vol.)
1	18.427	15.767	2,451,712	5,714.946	51.695	0.226
2	81.573	69.799	10,853,120	23,190	36.739	0.976

Lane 5 - t = 0

#	Vol. (Int.)	Local Bg. Corr. Vol.	Area	Rf	Density	Local Bg. Corr. Den.
1	3,695,934	2,860,622	440	0.382	8,399.85	6,501.416
2	11,215,145	9,761,518	492	0.488	22,795	19,840

#	% band purity	% lane purity	Rolling Bg. Corr. Vol.	Rolling Bg. Corr. Den.	Mol. Wt.	Rel. Quant. (w/ LB Corr. Vol.)
1	22.178	19.595	2,909,184	6,611.782	51.695	0.275
2	77.822	68.759	10,208,512	20,749	36.739	0.939

iBright™ Image Analysis Report

Katarina+ Chang

18 November 2022

CHEMI_03172022_142137

Date:	17 March 2022 02:21:37PM
Mode:	Chemi Blots
Notes:	
Model:	FL1500
Instrument name:	2462619090234
Serial No:	2462619090234
Firmware version:	1.6.0
iBA version:	5.0
Image size:	615px X 491px
Image area:	112.7mm X 90.16mm
Optical Zoom:	2x
Digital Zoom:	1.1x
Focus level:	455
Resolution:	5 x 5
Exposure time:	30000 ms
Exposure mode:	Normal

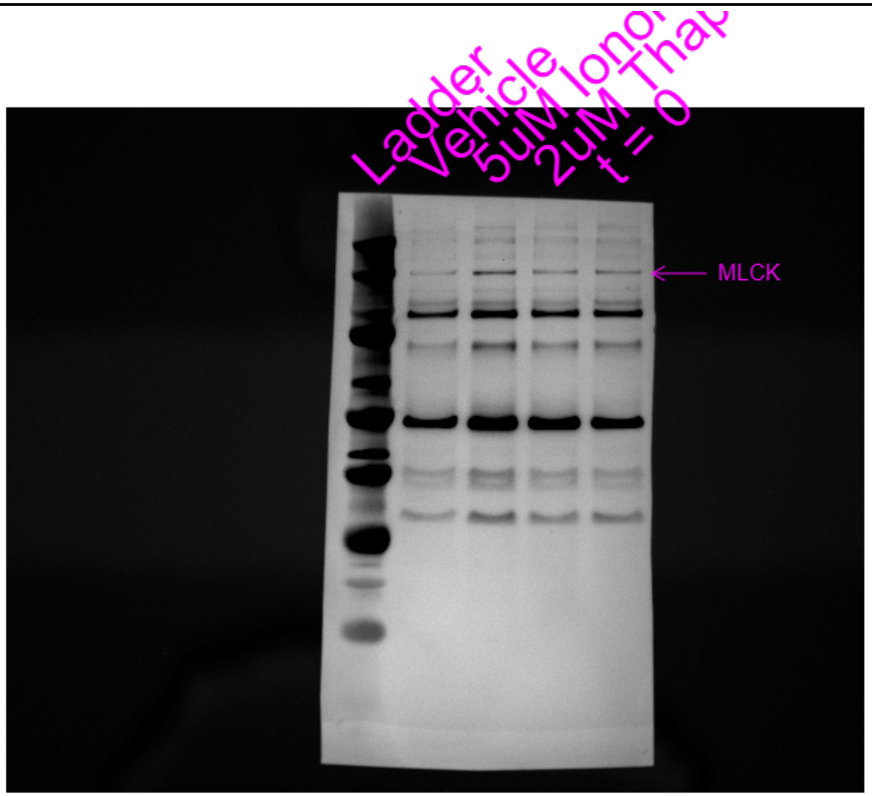

CHEMI_03172022_142137

Date:	17 March 2022 02:21:37PM
Mode:	Chemi Blots
Notes:	
Model:	FL1500
Instrument name:	2462619090234
Serial No:	2462619090234
Firmware version:	1.6.0
iBA version:	5.0
Image size:	615px X 491px
Image area:	112.7mm X 90.16mm
Optical Zoom:	2x
Digital Zoom:	1.1x
Focus level:	455
Resolution:	5 x 5
Exposure time:	30000 ms
Exposure mode:	Normal

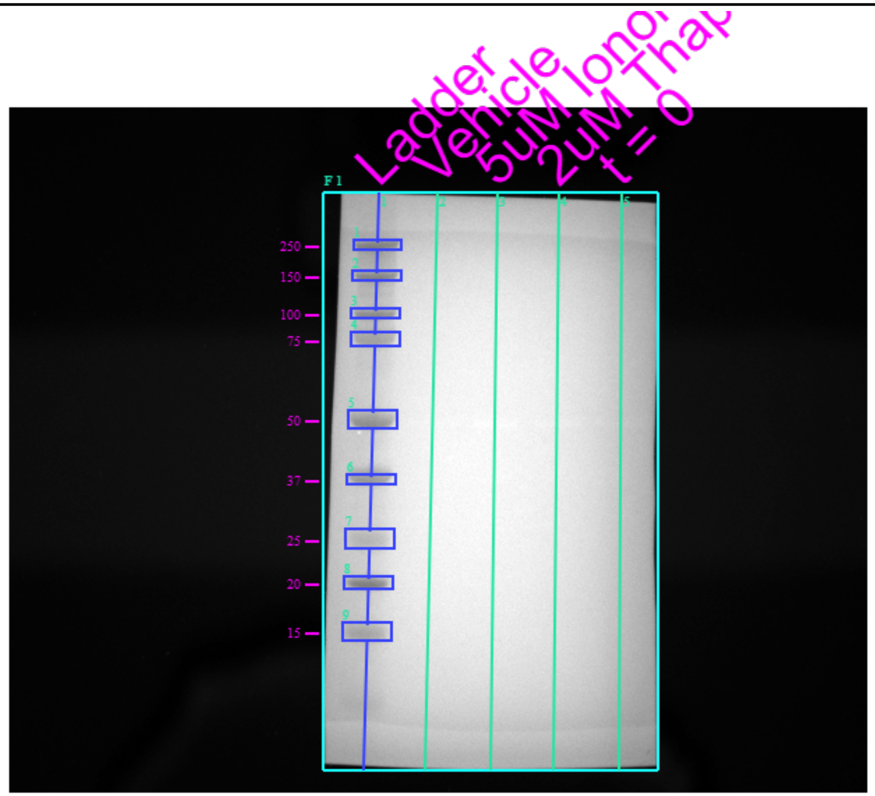

CHEMI_03172022_142137

Date:	17 March 2022 02:21:37PM
Mode:	Chemi Blots
Notes:	
Model:	FL1500
Instrument name:	2462619090234
Serial No:	2462619090234
Firmware version:	1.6.0
iBA version:	5.0
Image size:	615px X 491px
Image area:	112.7mm X 90.16mm
Optical Zoom:	2x
Digital Zoom:	1.1x
Focus level:	455
Resolution:	5 x 5
Exposure time:	30000 ms
Exposure mode:	Normal

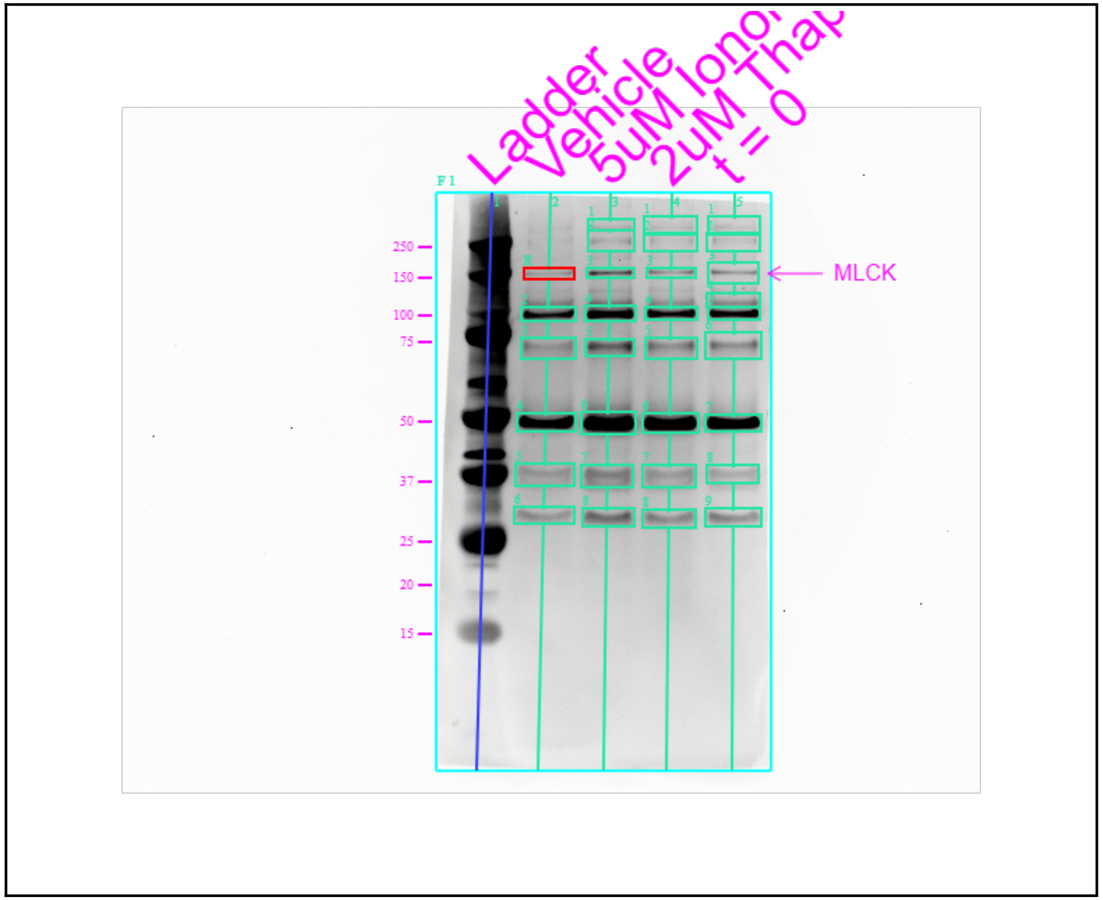

LANE AND BAND ANALYSIS DATA TABLE

CHEMI_03172022_142137

Frame: 1
Channel: Membrane
Sensitivity: 100
Molecular Weight Analysis Regression Method : Point to Point

Lane 1 - Ladder

#	Vol. (Int.)	Local Bg. Corr. Vol.	Area	Rf	Density	Local Bg. Corr. Den.	% band purity	% lane purity	Rolling Bg. Corr. Vol.	Rolling Bg. Corr. Den.	Mol. Wt.
1	10,282,455	1,521,557	280	0.089	36,723	5,434.132	9.637	3.445	1,812,224	6,472.229	250
2	9,995,782	1,672,019	288	0.143	34,707	5,805.624	8.144	2.911	1,531,392	5,317.333	150
3	9,626,582	1,782,629	288	0.208	33,425	6,189.686	8.668	3.099	1,629,952	5,659.556	100
4	12,150,025	1,796,183	396	0.254	30,681	4,535.817	8.683	3.104	1,632,768	4,123.152	75
5	15,983,209	3,468,040	504	0.391	31,712	6,881.033	18.45	6.596	3,469,568	6,884.063	50
6	9,028,399	1,612,381	288	0.495	31,348	5,598.548	10.843	3.876	2,039,040	7,080	37
7	15,029,465	1,676,537	540	0.599	27,832	3,104.698	9.013	3.222	1,694,976	3,138.844	25
8	11,974,230	2,740,332	360	0.674	33,261	7,612.035	15.376	5.497	2,891,520	8,032	20
9	15,329,448	1,934,752	504	0.758	30,415	3,838.795	11.186	3.999	2,103,552	4,173.714	15

Frame: 1
Channel: Chemi
Sensitivity: 100
Molecular Weight Analysis Regression Method : Point to Point

Lane 2 - Vehicle

#	Vol. (Int.)	Local Bg. Corr. Vol.	Area	Rf	Density	Local Bg. Corr. Den.
1	7,229,748	840,797	333	0.14	21,710	2,524.919
2	18,235,267	7,215,691	440	0.21	41,443	16,399
3	16,485,294	2,910,753	640	0.268	25,758	4,548.053
4	28,555,952	13,803,252	602	0.396	47,435	22,928
5	17,149,655	3,028,912	731	0.488	23,460	4,143.52
6	12,516,558	3,249,719	572	0.558	21,882	5,681.327

#	% band purity	% lane purity	Rolling Bg. Corr. Vol.	Rolling Bg. Corr. Den.	Mol. Wt.	Rel. Quant. (w/ LB Corr. Vol.)
1	2.935	1.819	1,034,752	3,107.363	154.545	1

#	% band purity	% lane purity	Rolling Bg. Corr. Vol.	Rolling Bg. Corr. Den.	Mol. Wt.	Rel. Quant. (w/ LB Corr. Vol.)
2	24.817	15.382	8,748,544	19,883	98.684	8.582
3	7.762	4.811	2,736,384	4,275.6	72.368	3.462
4	45.967	28.492	16,204,800	26,918	49.395	16.417
5	9.459	5.863	3,334,656	4,561.773	37.907	3.602
6	9.059	5.615	3,193,600	5,583.217	29.744	3.865

Lane 3 - 5uM Ionomycin

#	Vol. (Int.)	Local Bg. Corr. Vol.	Area	Rf	Density	Local Bg. Corr. Den.
1	5,196,465	781,141	306	0.056	16,981	2,552.75
2	9,922,875	966,230	544	0.082	18,240	1,776.158
3	7,909,683	2,833,665	280	0.138	28,248	10,120
4	20,172,428	9,668,163	444	0.208	45,433	21,775
5	15,546,412	5,673,783	468	0.268	33,218	12,123
6	35,208,612	17,901,548	680	0.399	51,777	26,325
7	17,727,991	5,359,113	646	0.49	27,442	8,295.842
8	14,462,154	5,878,856	532	0.56	27,184	11,050

#	% band purity	% lane purity	Rolling Bg. Corr. Vol.	Rolling Bg. Corr. Den.	Mol. Wt.	Rel. Quant. (w/ LB Corr. Vol.)
1	4.839	3.627	2,862,080	9,353.203	NA	0.929
2	6.755	5.064	3,995,648	7,344.941	NA	1.149
3	5.173	3.877	3,059,456	10,926	159.091	3.37
4	18.659	13.987	11,036,416	24,856	100	11.499
5	9.565	7.17	5,657,344	12,088	72.368	6.748
6	35.112	26.32	20,767,488	30,540	49.093	21.291
7	9.755	7.312	5,769,728	8,931.467	37.605	6.374
8	10.142	7.602	5,998,592	11,275	29.465	6.992

Lane 4 - 2uM Thapsigargin

#	Vol. (Int.)	Local Bg. Corr. Vol.	Area	Rf	Density	Local Bg. Corr. Den.
1	7,930,115	706,552	546	0.056	14,524	1,294.053
2	8,312,070	486,682	546	0.085	15,223	891.359
3	5,983,852	1,519,966	288	0.138	20,777	5,277.663
4	16,148,448	8,259,569	380	0.208	42,495	21,735

#	Vol. (Int.)	Local Bg. Corr. Vol.	Area	Rf	Density	Local Bg. Corr. Den.	[redacted]
5	14,746,877	4,224,654	608	0.268	24,254	6,948.444	[redacted]
6	30,983,008	16,724,975	615	0.399	50,378	27,195	[redacted]
7	14,914,042	3,674,662	663	0.49	22,494	5,542.477	[redacted]
8	12,197,605	4,261,047	546	0.563	22,339	7,804.116	[redacted]

#	% band purity	% lane purity	Rolling Bg. Corr. Vol.	Rolling Bg. Corr. Den.	Mol. Wt.	Rel. Quant. (w/ LB Corr. Vol.)
1	3.472	2.546	1,604,608	2,938.842	NA	0.84
2	1.875	1.375	866,304	1,586.637	NA	0.579
3	3.228	2.367	1,491,712	5,179.556	159.091	1.808
4	21.036	15.428	9,721,600	25,583	100	9.823
5	8.762	6.426	4,049,152	6,659.789	72.368	5.025
6	43.737	32.078	20,213,248	32,867	49.093	19.892
7	8.551	6.272	3,951,872	5,960.591	37.605	4.37
8	9.34	6.85	4,316,672	7,905.993	29.186	5.068

Lane 5 - t = 0

#	Vol. (Int.)	Local Bg. Corr. Vol.	Area	Rf	Density	Local Bg. Corr. Den.	[redacted]
1	6,929,557	760,247	532	0.056	13,025	1,429.037	[redacted]
2	7,552,340	696,183	546	0.085	13,832	1,275.062	[redacted]
3	9,632,961	1,613,095	592	0.138	16,271	2,724.824	[redacted]
4	9,867,180	439,120	429	0.186	23,000	1,023.591	[redacted]
5	16,068,134	8,870,193	380	0.208	42,284	23,342	[redacted]
6	16,442,718	5,039,801	820	0.263	20,052	6,146.099	[redacted]
7	25,391,215	16,554,303	533	0.399	47,638	31,058	[redacted]
8	9,470,897	2,483,117	532	0.486	17,802	4,667.514	[redacted]
9	10,854,519	5,256,718	574	0.56	18,910	9,158.045	[redacted]

#	% band purity	% lane purity	Rolling Bg. Corr. Vol.	Rolling Bg. Corr. Den.	Mol. Wt.	Rel. Quant. (w/ LB Corr. Vol.)
1	5.377	4.488	2,831,616	5,322.586	NA	0.904
2	2.91	2.429	1,532,416	2,806.623	NA	0.828
3	3.439	2.87	1,810,944	3,059.027	159.091	1.919
4	7.49	6.251	3,944,192	9,193.921	116.667	0.522
5	20.172	16.835	10,621,952	27,952	100	10.55

#	% band purity	% lane purity	Rolling Bg. Corr. Vol.	Rolling Bg. Corr. Den.	Mol. Wt.	Rel. Quant. (w/ LB Corr. Vol.)
6	9.752	8.138	5,134,848	6,262.01	73.246	5.994
7	34.463	28.762	18,147,071	34,047	49.093	19.689
8	6.244	5.211	3,287,808	6,180.09	38.209	2.953
9	10.153	8.473	5,346,048	9,313.672	29.465	6.252

iBright™ Image Analysis Report

Katarina+ Chang
18 November 2022

CHEMI_03212022_133508

Date:	21 March 2022 01:35:08PM
Mode:	Chemi Blots
Notes:	
Model:	FL1500
Instrument name:	2462619090234
Serial No:	2462619090234
Firmware version:	1.6.0
iBA version:	5.0
Image size:	615px X 491px
Image area:	112.7mm X 90.16mm
Optical Zoom:	2x
Digital Zoom:	1.1x
Focus level:	455
Resolution:	5 x 5
Exposure time:	733 ms
Exposure mode:	Normal

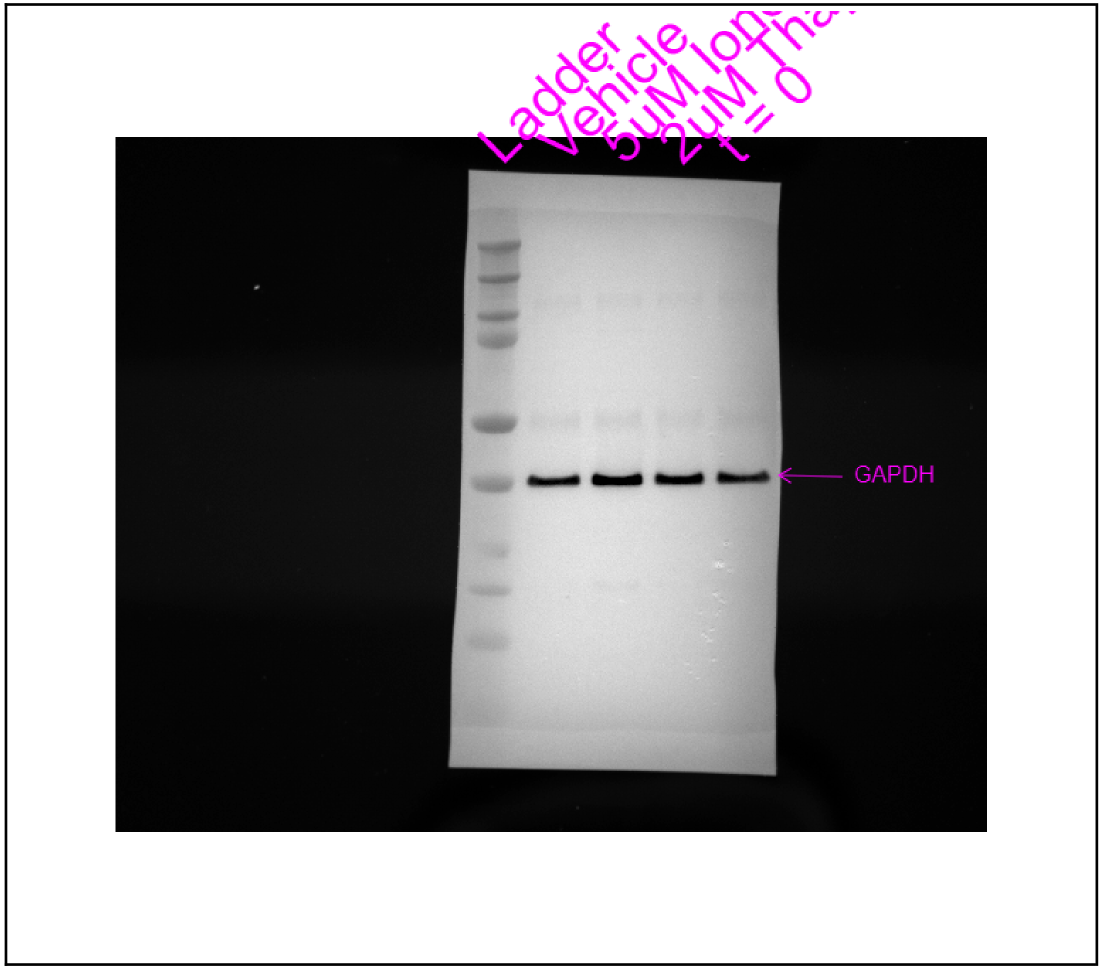

CHEMI_03212022_133508

Date:	21 March 2022 01:35:08PM
Mode:	Chemi Blots
Notes:	
Model:	FL1500
Instrument name:	2462619090234
Serial No:	2462619090234
Firmware version:	1.6.0
iBA version:	5.0
Image size:	615px X 491px
Image area:	112.7mm X 90.16mm
Optical Zoom:	2x
Digital Zoom:	1.1x
Focus level:	455
Resolution:	5 x 5
Exposure time:	733 ms
Exposure mode:	Normal

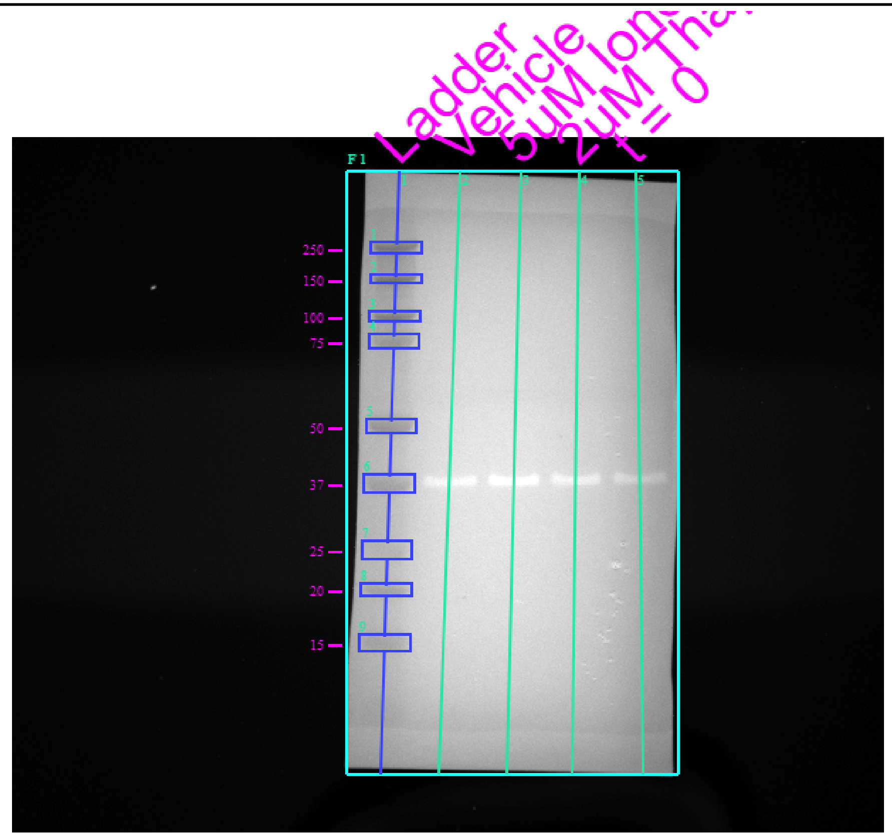

CHEMI_03212022_133508

Date:	21 March 2022 01:35:08PM
Mode:	Chemi Blots
Notes:	
Model:	FL1500
Instrument name:	2462619090234
Serial No:	2462619090234
Firmware version:	1.6.0
iBA version:	5.0
Image size:	615px X 491px
Image area:	112.7mm X 90.16mm
Optical Zoom:	2x
Digital Zoom:	1.1x
Focus level:	455
Resolution:	5 x 5
Exposure time:	733 ms
Exposure mode:	Normal

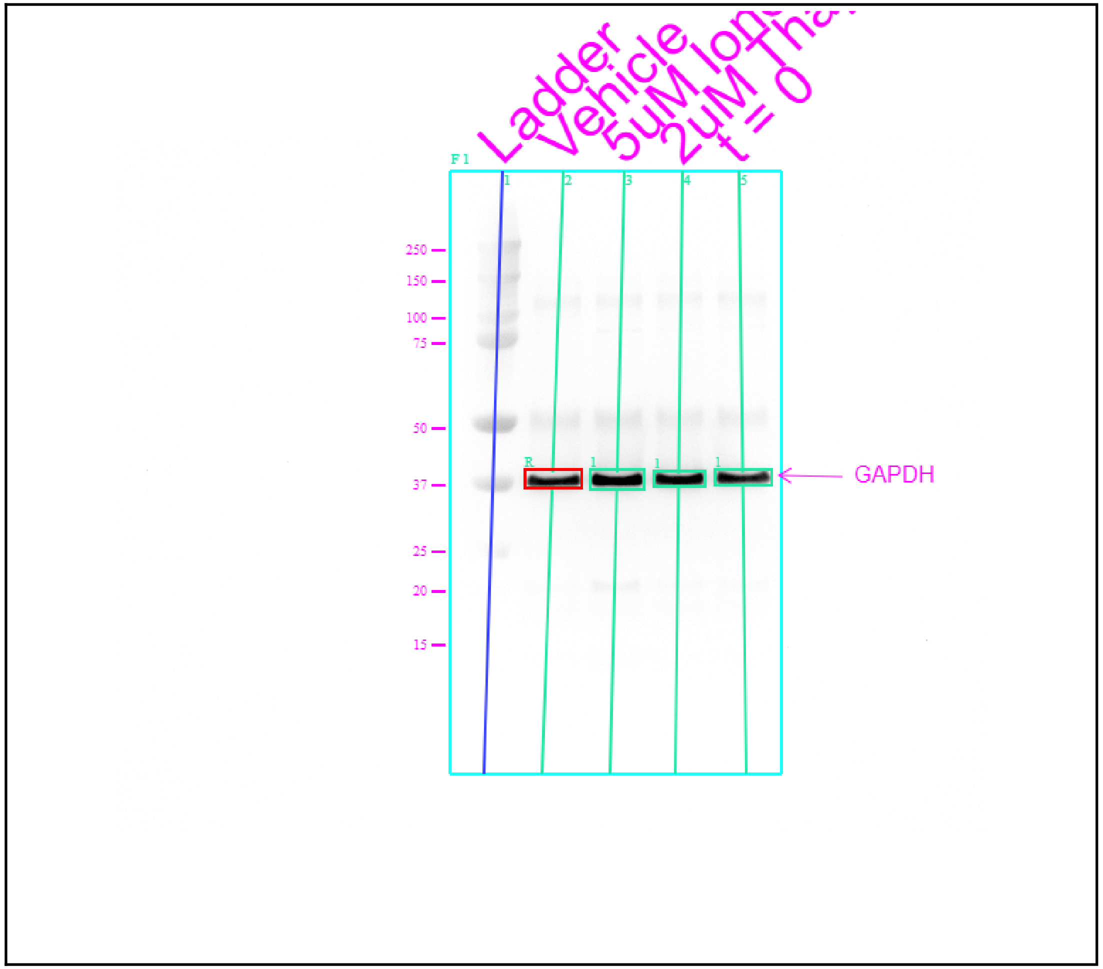

LANE AND BAND ANALYSIS DATA TABLE

CHEMI_03212022_133508

Frame: 1
Channel: Membrane
Sensitivity: 100
Molecular Weight Analysis Regression Method : Point to Point

Lane 1 - Ladder

#	Vol. (Int.)	Local Bg. Corr. Vol.	Area	Rf	Density	Local Bg. Corr. Den.	% band purity	% lane purity	Mol. Wt.
1	11,979,833	1,545,950	333	0.127	35,975	4,642.494	12.944	2.6	250
2	9,232,227	1,451,599	259	0.178	35,645	5,604.631	12.154	2.004	150
3	9,975,632	1,571,314	296	0.239	33,701	5,308.494	13.156	2.165	100
4	12,184,642	1,350,680	396	0.282	30,769	3,410.81	11.309	2.644	75
5	12,198,046	1,763,412	396	0.423	30,803	4,453.063	14.764	2.647	50
6	15,088,366	1,363,398	518	0.516	29,128	2,632.043	11.415	3.274	37
7	13,644,563	618,552	504	0.627	27,072	1,227.286	5.179	2.961	25
8	10,728,762	1,087,802	370	0.692	28,996	2,940.008	9.108	2.328	20
9	14,113,190	1,191,062	481	0.782	29,341	2,476.221	9.972	3.063	15

Frame: 1
Channel: Chemi
Sensitivity: 100
Molecular Weight Analysis Regression Method : Point to Point

Lane 2 - Vehicle

#	Vol. (Int.)	Local Bg. Corr. Vol.	Area	Rf	Density	Local Bg. Corr. Den.	% band purity	% lane purity	Mol. Wt.	Rel. Quant. (w/ LB Corr. Vol.)
1	10,627,009	9,023,221	574	0.509	18,513	15,719	100	55.487	37.975	1

Lane 3 - 5uM Ionomycin

#	Vol. (Int.)	Local Bg. Corr. Vol.	Area	Rf	Density	Local Bg. Corr. Den.	% band purity	% lane purity	Mol. Wt.	Rel. Quant. (w/ LB Corr. Vol.)
1	14,539,747	12,451,030	585	0.512	24,854	21,283	100	57.582	37.65	1.38

Lane 4 - 2uM Thapsigargin

#	Vol. (Int.)	Local Bg. Corr. Vol.	Area	Rf	Density	Local Bg. Corr. Den.	% band purity	% lane purity	Mol. Wt.	Rel. Quant. (w/ LB Corr. Vol.)

#	Vol. (Int.)	Local Bg. Corr. Vol.	Area	Rf	Density	Local Bg. Corr. Den.	% band purity	% lane purity	Mol. Wt.	Rel. Quant. (w/ LB Corr. Vol.)
1	11,258,177	9,572,595	444	0.509	25,356	21,559	100	53.305	37.975	1.061

Lane 5 - t = 0

#	Vol. (Int.)	Local Bg. Corr. Vol.	Area	Rf	Density	Local Bg. Corr. Den.	% band purity	% lane purity	Mol. Wt.	Rel. Quant. (w/ LB Corr. Vol.)
1	9,953,854	8,812,112	492	0.507	20,231	17,910	100	59.909	38.3	0.977

iBright™ Image Analysis Report

Katarina+ Chang

18 November 2022

CHEMI_03202022_143320

Date:	20 March 2022 02:33:20PM
Mode:	Chemi Blots
Notes:	
Model:	FL1500
Instrument name:	2462619090234
Serial No:	2462619090234
Firmware version:	1.6.0
iBA version:	5.0
Image size:	615px X 491px
Image area:	112.7mm X 90.16mm
Optical Zoom:	2x
Digital Zoom:	1.1x
Focus level:	455
Resolution:	5 x 5
Exposure time:	10000 ms
Exposure mode:	Normal

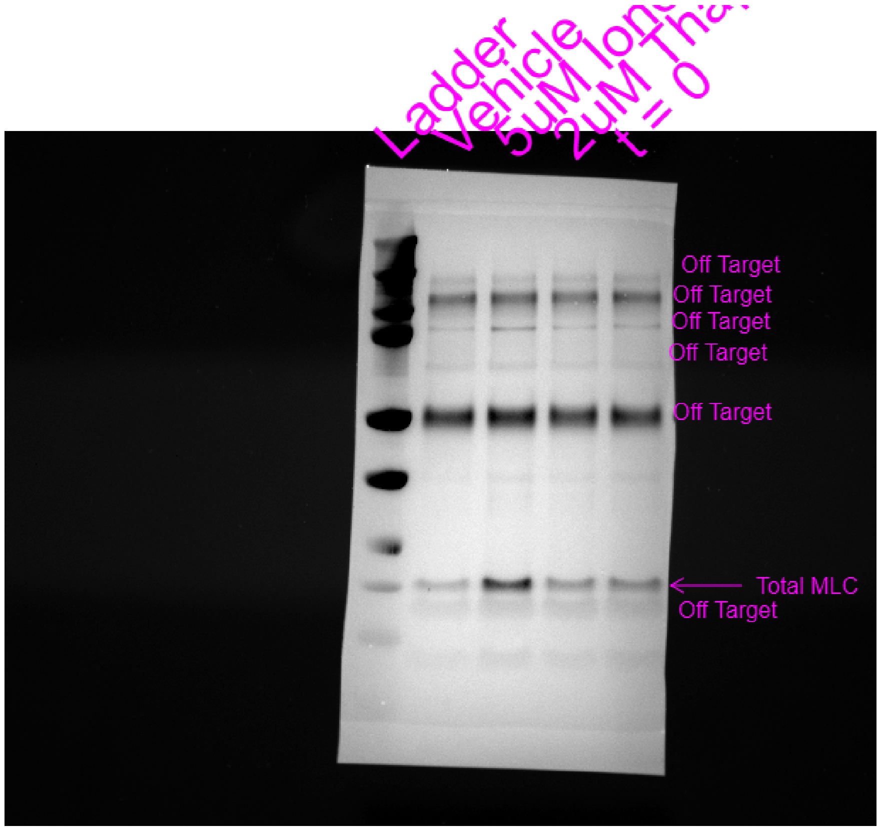

CHEMI_03202022_143320

Date:	20 March 2022 02:33:20PM
Mode:	Chemi Blots
Notes:	
Model:	FL1500
Instrument name:	2462619090234
Serial No:	2462619090234
Firmware version:	1.6.0
iBA version:	5.0
Image size:	615px X 491px
Image area:	112.7mm X 90.16mm
Optical Zoom:	2x
Digital Zoom:	1.1x
Focus level:	455
Resolution:	5 x 5
Exposure time:	10000 ms
Exposure mode:	Normal

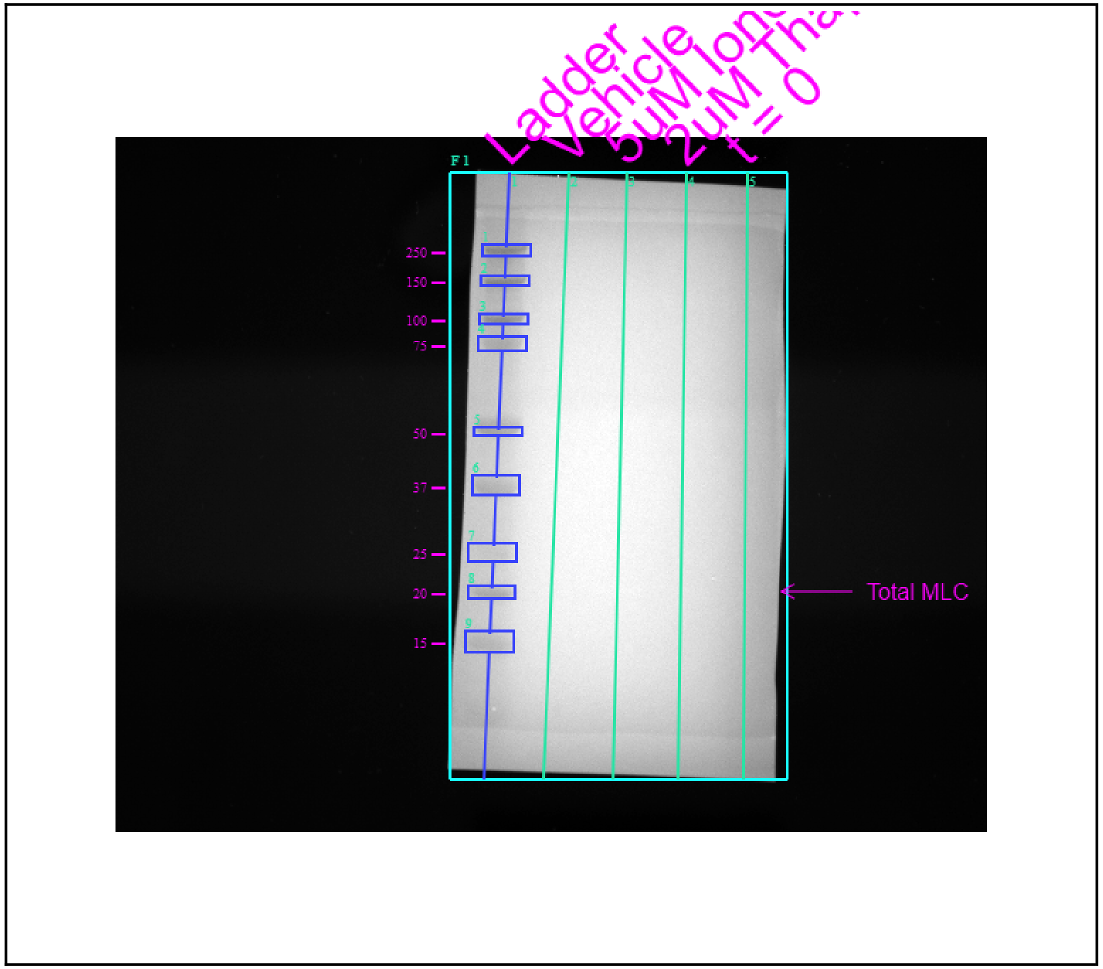

CHEMI_03202022_143320

Date:	20 March 2022 02:33:20PM
Mode:	Chemi Blots
Notes:	
Model:	FL1500
Instrument name:	2462619090234
Serial No:	2462619090234
Firmware version:	1.6.0
iBA version:	5.0
Image size:	615px X 491px
Image area:	112.7mm X 90.16mm
Optical Zoom:	2x
Digital Zoom:	1.1x
Focus level:	455
Resolution:	5 x 5
Exposure time:	10000 ms
Exposure mode:	Normal

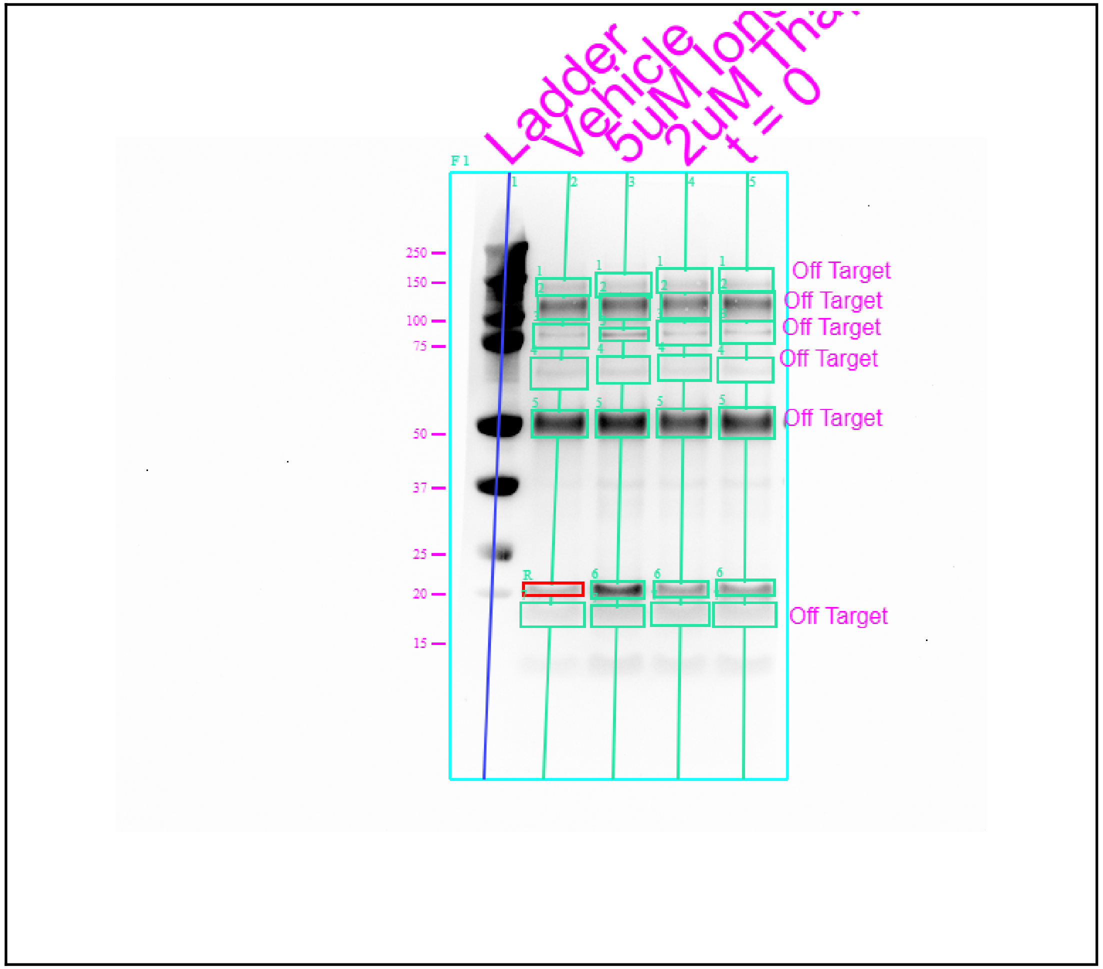

LANE AND BAND ANALYSIS DATA TABLE

CHEMI_03202022_143320

Frame: 1
Channel: Membrane
Sensitivity: 100
Molecular Weight Analysis Regression Method : Point to Point

Lane 1 - Ladder

#	Vol. (Int.)	Local Bg. Corr. Vol.	Area	Rf	Density	Local Bg. Corr. Den.	% band purity	% lane purity	Rolling Bg. Corr. Vol.	Rolling Bg. Corr. Den.	Mol. Wt.
1	11,241,339	1,645,054	315	0.128	35,686	5,222.397	13.164	2.81	1,603,328	5,089.93	250
2	9,741,686	1,514,567	280	0.177	34,791	5,409.17	10.784	2.302	1,313,536	4,691.2	150
3	9,332,514	1,514,561	280	0.24	33,330	5,409.147	11.203	2.391	1,364,480	4,873.143	100
4	11,646,756	1,284,091	385	0.282	30,251	3,335.302	8.987	1.918	1,094,656	2,843.262	75
5	7,517,801	974,078	245	0.427	30,684	3,975.83	10.253	2.188	1,248,768	5,097.012	50
6	14,752,416	1,918,148	510	0.515	28,926	3,761.075	15.812	3.375	1,925,888	3,776.251	37
7	13,161,234	661,144	490	0.625	26,859	1,349.274	6.028	1.287	734,208	1,498.384	25
8	10,131,851	1,343,508	340	0.69	29,799	3,951.496	11.463	2.447	1,396,224	4,106.541	20
9	16,286,609	1,414,269	560	0.772	29,083	2,525.482	12.306	2.627	1,498,880	2,676.571	15

Frame: 1
Channel: Chemi
Sensitivity: 100
Molecular Weight Analysis Regression Method : Point to Point

Lane 2 - Vehicle

#	Vol. (Int.)	Local Bg. Corr. Vol.	Area	Rf	Density	Local Bg. Corr. Den.
1	6,153,467	479,670	507	0.189	12,137	946.095
2	16,587,519	9,187,030	666	0.221	24,906	13,794
3	8,089,115	746,479	720	0.268	11,234	1,036.777
4	8,886,577	1,612,355	943	0.331	9,423.73	1,709.815
5	28,078,557	19,097,381	800	0.413	35,098	23,871
6	4,736,441	2,728,263	430	0.685	11,014	6,344.799
7	4,505,993	1,140,721	828	0.727	5,442.021	1,377.683

#	% band purity	% lane purity	Rolling Bg. Corr. Vol.	Rolling Bg. Corr. Den.	Mol. Wt.	Rel. Quant. (w/ LB Corr. Vol.)

#	% band purity	% lane purity	Rolling Bg. Corr. Vol.	Rolling Bg. Corr. Den.	Mol. Wt.	Rel. Quant. (w/ LB Corr. Vol.)
1	3.71	3.43	1,455,104	2,870.028	140.741	0.176
2	25.826	23.876	10,128,128	15,207	114.815	3.367
3	3.432	3.173	1,346,048	1,869.511	83.333	0.274
4	2.887	2.669	1,132,032	1,200.458	66.532	0.591
5	53.224	49.205	20,872,960	26,091	52.419	7
6	7.684	7.104	3,013,376	7,007.851	20.357	1
7	3.238	2.993	1,269,760	1,533.527	17.714	0.418

Lane 3 - 5uM Ionomycin

#	Vol. (Int.)	Local Bg. Corr. Vol.	Area	Rf	Density	Local Bg. Corr. Den.
1	7,128,402	744,712	720	0.184	9,900.558	1,034.323
2	16,621,533	10,337,538	684	0.221	24,300	15,113
3	4,678,131	1,684,224	350	0.266	13,366	4,812.07
4	6,136,805	1,546,057	760	0.324	8,074.743	2,034.286
5	28,324,345	20,572,566	760	0.413	37,268	27,069
6	14,470,812	10,736,220	494	0.688	29,293	21,733
7	5,304,004	1,363,451	608	0.73	8,723.691	2,242.519

#	% band purity	% lane purity	Rolling Bg. Corr. Vol.	Rolling Bg. Corr. Den.	Mol. Wt.	Rel. Quant. (w/ LB Corr. Vol.)
1	4.033	3.637	2,135,808	2,966.4	144.444	0.273
2	20.732	18.696	10,980,096	16,052	114.815	3.789
3	3.233	2.916	1,712,384	4,892.526	84.722	0.617
4	2.067	1.864	1,094,656	1,440.337	67.742	0.567
5	42.605	38.422	22,565,120	29,690	52.419	7.541
6	22.719	20.488	12,032,768	24,357	20.179	3.935
7	4.611	4.158	2,442,240	4,016.842	17.571	0.5

Lane 4 - 2uM Thapsigargin

#	Vol. (Int.)	Local Bg. Corr. Vol.	Area	Rf	Density	Local Bg. Corr. Den.
1	5,682,876	996,675	720	0.177	7,892.883	1,384.272
2	14,301,177	8,734,804	684	0.219	20,908	12,770
3	5,883,947	951,370	702	0.263	8,381.691	1,355.228
4	4,915,107	1,279,874	722	0.322	6,807.627	1,772.679

#	Vol. (Int.)	Local Bg. Corr. Vol.	Area	Rf	Density	Local Bg. Corr. Den.
5	25,167,045	18,138,882	819	0.413	30,728	22,147
6	7,125,752	4,512,462	468	0.685	15,225	9,642.014
7	5,581,639	1,542,251	714	0.727	7,817.422	2,160.015

#	% band purity	% lane purity	Rolling Bg. Corr. Vol.	Rolling Bg. Corr. Den.	Mol. Wt.	Rel. Quant. (w/ LB Corr. Vol.)
1	3.696	3.345	1,530,624	2,125.867	150	0.365
2	23.163	20.967	9,593,088	14,024	116.667	3.202
3	3.029	2.742	1,254,400	1,786.895	86.111	0.349
4	1.912	1.731	792,064	1,097.042	68.145	0.469
5	47.729	43.205	19,767,552	24,136	52.419	6.649
6	13.35	12.085	5,529,088	11,814	20.357	1.654
7	7.121	6.446	2,949,376	4,130.779	17.714	0.565

Lane 5 - t = 0

#	Vol. (Int.)	Local Bg. Corr. Vol.	Area	Rf	Density	Local Bg. Corr. Den.
1	4,601,272	820,237	702	0.177	6,554.519	1,168.429
2	15,726,150	10,814,821	858	0.219	18,328	12,604
3	4,602,136	1,018,668	663	0.263	6,941.382	1,536.453
4	3,342,295	989,589	720	0.324	4,642.076	1,374.43
5	26,153,674	21,086,571	920	0.413	28,427	22,920
6	7,075,531	4,881,028	504	0.683	14,038	9,684.581
7	4,839,461	1,622,611	810	0.727	5,974.643	2,003.224

#	% band purity	% lane purity	Rolling Bg. Corr. Vol.	Rolling Bg. Corr. Den.	Mol. Wt.	Rel. Quant. (w/ LB Corr. Vol.)
1	4.126	3.933	1,959,168	2,790.838	150	0.301
2	24.781	23.622	11,766,784	13,714	116.667	3.964
3	3.29	3.136	1,562,112	2,356.127	86.111	0.373
4	2.047	1.951	972,032	1,350.044	67.742	0.363
5	47.39	45.173	22,502,144	24,458	52.419	7.729
6	12.348	11.77	5,863,168	11,633	20.536	1.789
7	6.017	5.736	2,857,216	3,527.427	17.714	0.595

iBright™ Image Analysis Report

Katarina+ Chang

18 November 2022

CHEMI_03212022_133112

Date:	21 March 2022 01:31:12PM
Mode:	Chemi Blots
Notes:	
Model:	FL1500
Instrument name:	2462619090234
Serial No:	2462619090234
Firmware version:	1.6.0
iBA version:	5.0
Image size:	676px X 540px
Image area:	118.63mm X 94.91mm
Optical Zoom:	1.9x
Digital Zoom:	1x
Focus level:	430
Resolution:	5 x 5
Exposure time:	574 ms
Exposure mode:	Normal

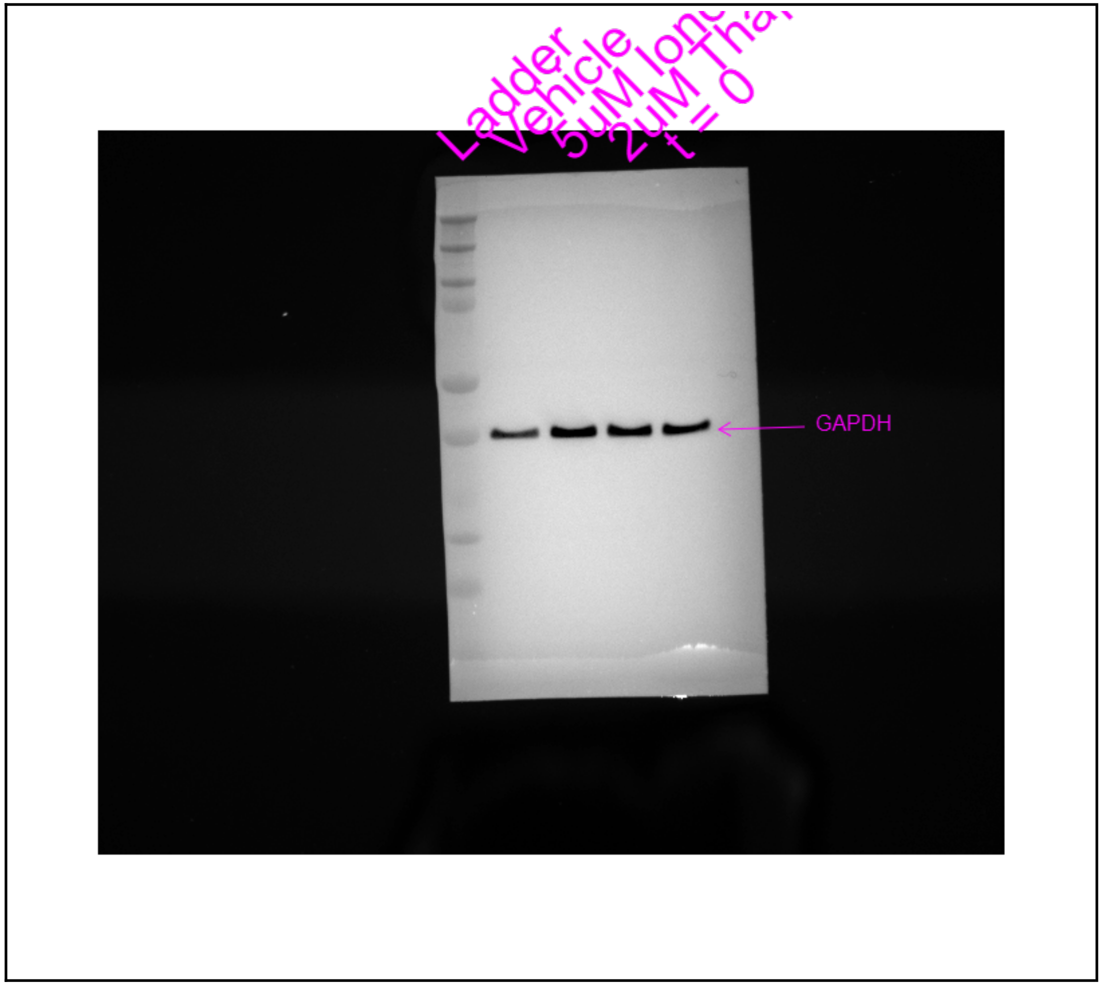

CHEMI_03212022_133112

Date:	21 March 2022 01:31:12PM
Mode:	Chemi Blots
Notes:	
Model:	FL1500
Instrument name:	2462619090234
Serial No:	2462619090234
Firmware version:	1.6.0
iBA version:	5.0
Image size:	676px X 540px
Image area:	118.63mm X 94.91mm
Optical Zoom:	1.9x
Digital Zoom:	1x
Focus level:	430
Resolution:	5 x 5
Exposure time:	574 ms
Exposure mode:	Normal

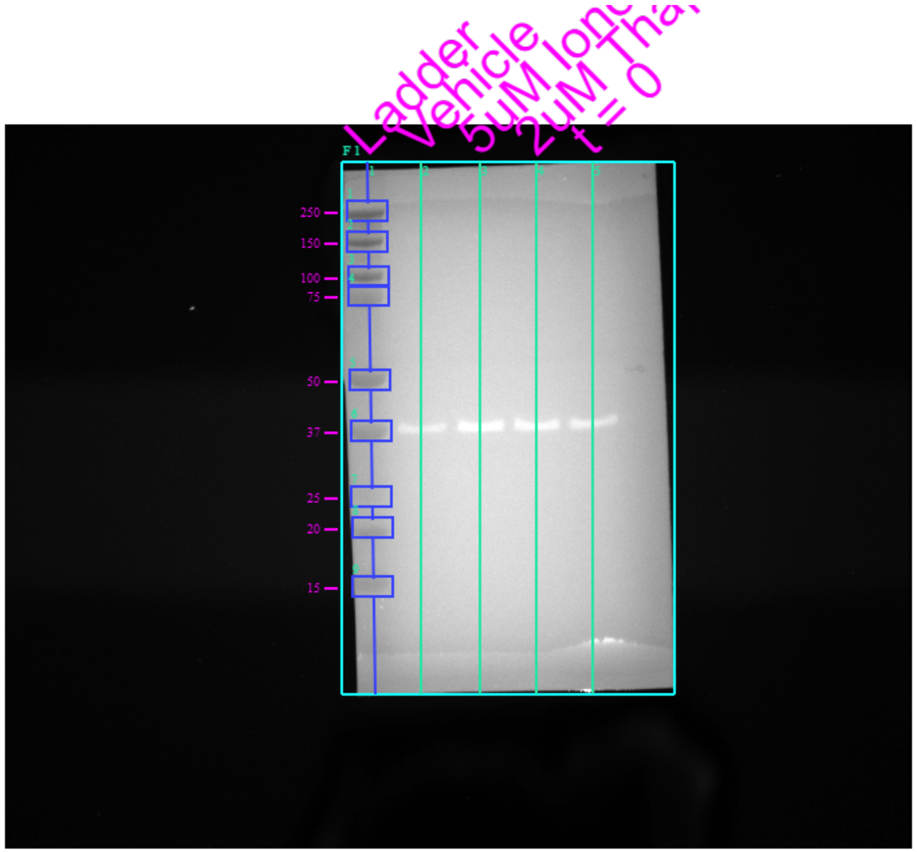

CHEMI_03212022_133112

Date:	21 March 2022 01:31:12PM
Mode:	Chemi Blots
Notes:	
Model:	FL1500
Instrument name:	2462619090234
Serial No:	2462619090234
Firmware version:	1.6.0
iBA version:	5.0
Image size:	676px X 540px
Image area:	118.63mm X 94.91mm
Optical Zoom:	1.9x
Digital Zoom:	1x
Focus level:	430
Resolution:	5 x 5
Exposure time:	574 ms
Exposure mode:	Normal

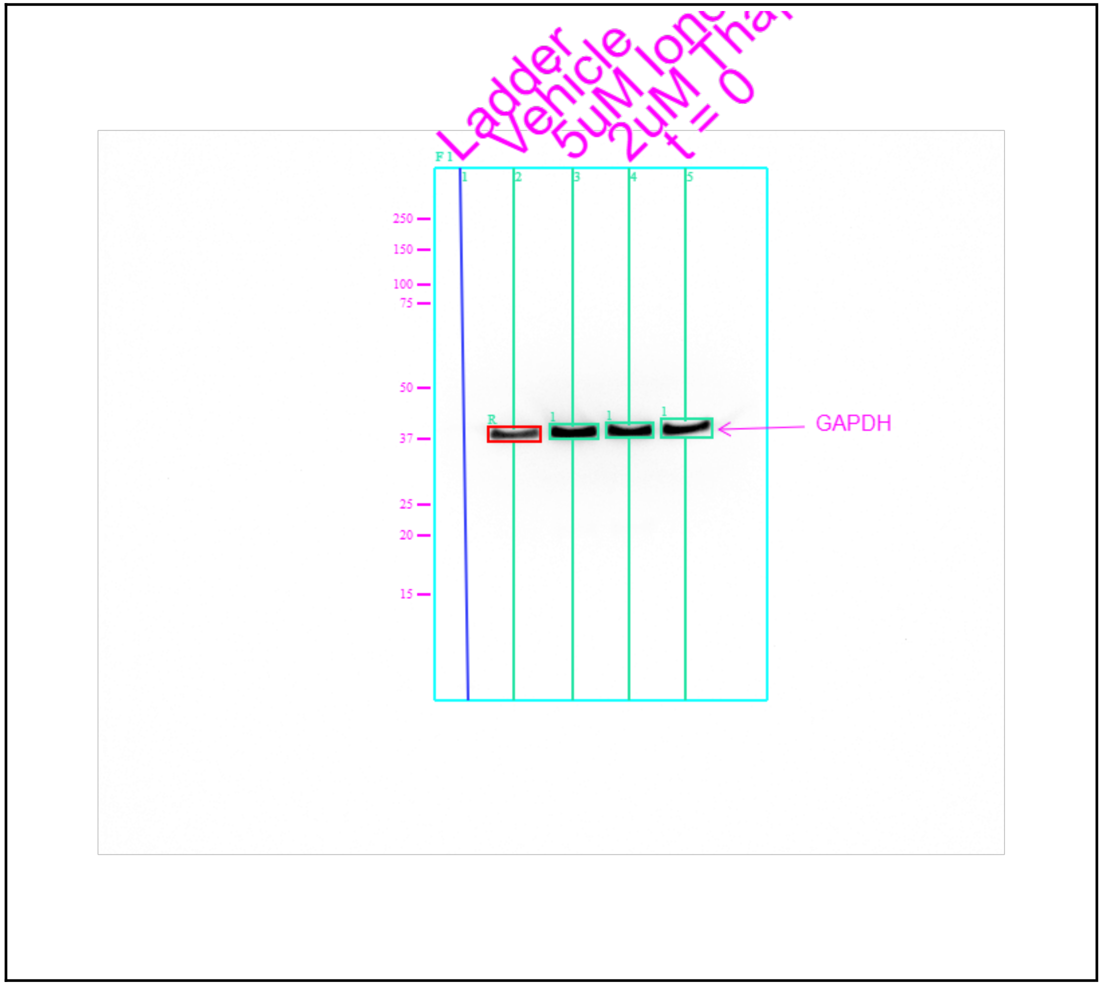

LANE AND BAND ANALYSIS DATA TABLE

CHEMI_03212022_133112

Frame: 1
Channel: Membrane
Sensitivity: 100
Molecular Weight Analysis Regression Method : Point to Point

Lane 1 - Ladder

#	Vol. (Int.)	Local Bg. Corr. Vol.	Area	Rf	Density	Local Bg. Corr. Den.	% band purity	% lane purity	Mol. Wt.
1	18,321,071	1,171,577	496	0.091	36,937	2,362.051	16.18	4.164	250
2	17,759,560	1,390,784	496	0.149	35,805	2,804.001	19.207	4.037	150
3	16,725,589	1,359,117	496	0.214	33,720	2,740.157	18.77	3.802	100
4	15,945,382	738,157	496	0.249	32,147	1,488.22	10.194	3.624	75
5	15,546,853	1,453,750	496	0.408	31,344	2,930.949	20.077	3.534	50
6	14,785,987	712,079	496	0.504	29,810	1,435.644	9.834	3.361	37
7	13,706,916	NA	496	0.627	27,634	NA	NA	3.116	25
8	14,477,739	31,210	496	0.685	29,188	62.925	0.431	3.291	20
9	15,477,754	384,227	496	0.796	31,205	774.652	5.306	3.518	15

Frame: 1
Channel: Chemi
Sensitivity: 100
Molecular Weight Analysis Regression Method : Point to Point

Lane 2 - Vehicle

#	Vol. (Int.)	Local Bg. Corr. Vol.	Area	Rf	Density	Local Bg. Corr. Den.	% band purity	% lane purity	Mol. Wt.	Rel. Quant. (w/ LB Corr. Vol.)
1	7,688,772	6,569,802	480	0.499	16,018	13,687	100	67.892	37.684	1

Lane 3 - 5uM Ionomycin

#	Vol. (Int.)	Local Bg. Corr. Vol.	Area	Rf	Density	Local Bg. Corr. Den.	% band purity	% lane purity	Mol. Wt.	Rel. Quant. (w/ LB Corr. Vol.)
1	12,414,203	10,471,574	444	0.494	27,959	23,584	100	67.019	38.368	1.594

Lane 4 - 2uM Thapsigargin

#	Vol. (Int.)	Local Bg. Corr. Vol.	Area	Rf	Density	Local Bg. Corr. Den.	% band purity	% lane purity	Mol. Wt.	Rel. Quant. (w/ LB Corr. Vol.)

#	Vol. (Int.)	Local Bg. Corr. Vol.	Area	Rf	Density	Local Bg. Corr. Den.	% band purity	% lane purity	Mol. Wt.	Rel. Quant. (w/ LB Corr. Vol.)
1	10,754,484	9,101,112	432	0.491	24,894	21,067	100	65.778	38.711	1.385

Lane 5 - t = 0

#	Vol. (Int.)	Local Bg. Corr. Vol.	Area	Rf	Density	Local Bg. Corr. Den.	% band purity	% lane purity	Mol. Wt.	Rel. Quant. (w/ LB Corr. Vol.)
1	10,630,964	9,674,113	585	0.489	18,172	16,536	100	78.283	39.053	1.473

iBright™ Image Analysis Report

Katarina+ Chang

18 November 2022

CHEMI_03202022_142709

Date:	20 March 2022 02:27:09PM
Mode:	Chemi Blots
Notes:	
Model:	FL1500
Instrument name:	2462619090234
Serial No:	2462619090234
Firmware version:	1.6.0
iBA version:	5.0
Image size:	615px X 491px
Image area:	112.7mm X 90.16mm
Optical Zoom:	2x
Digital Zoom:	1.1x
Focus level:	455
Resolution:	5 x 5
Exposure time:	44304 ms
Exposure mode:	Normal

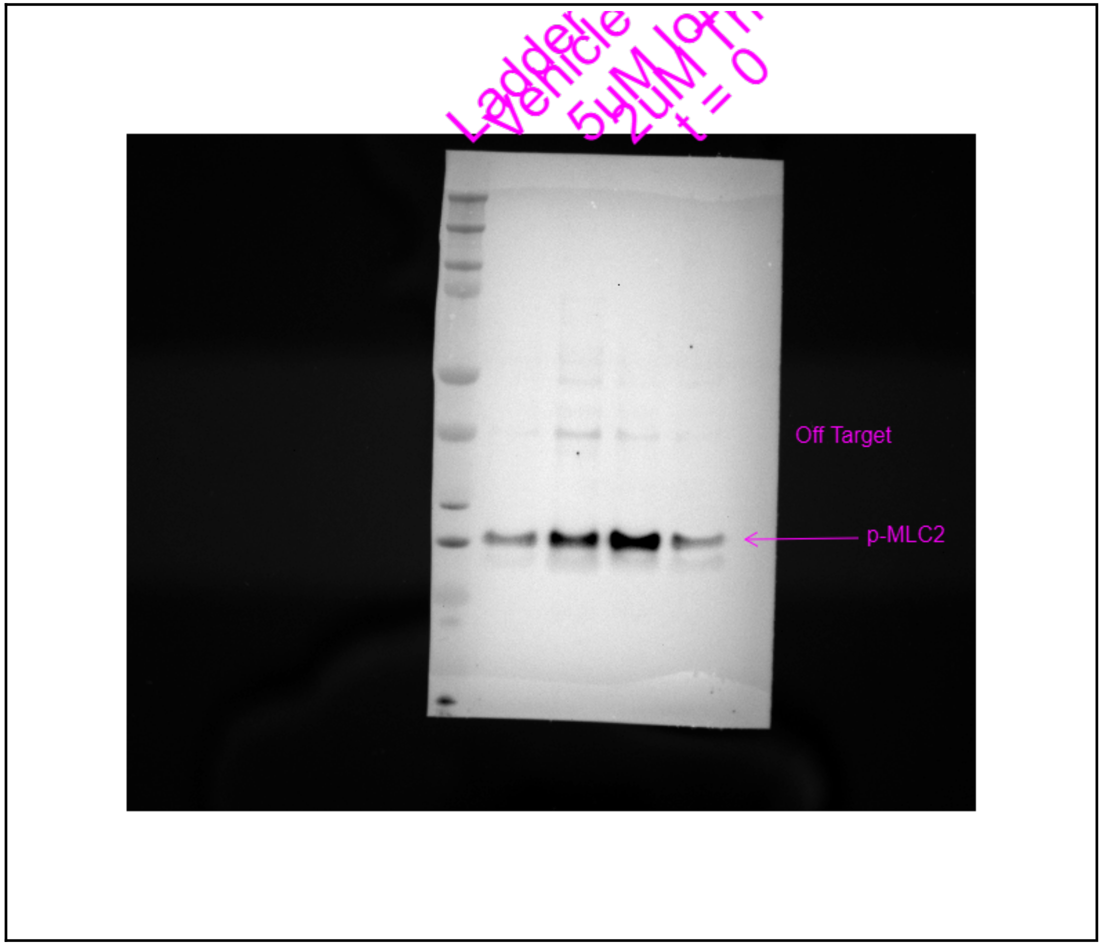

CHEMI_03202022_142709

Date:	20 March 2022 02:27:09PM
Mode:	Chemi Blots
Notes:	
Model:	FL1500
Instrument name:	2462619090234
Serial No:	2462619090234
Firmware version:	1.6.0
iBA version:	5.0
Image size:	615px X 491px
Image area:	112.7mm X 90.16mm
Optical Zoom:	2x
Digital Zoom:	1.1x
Focus level:	455
Resolution:	5 x 5
Exposure time:	44304 ms
Exposure mode:	Normal

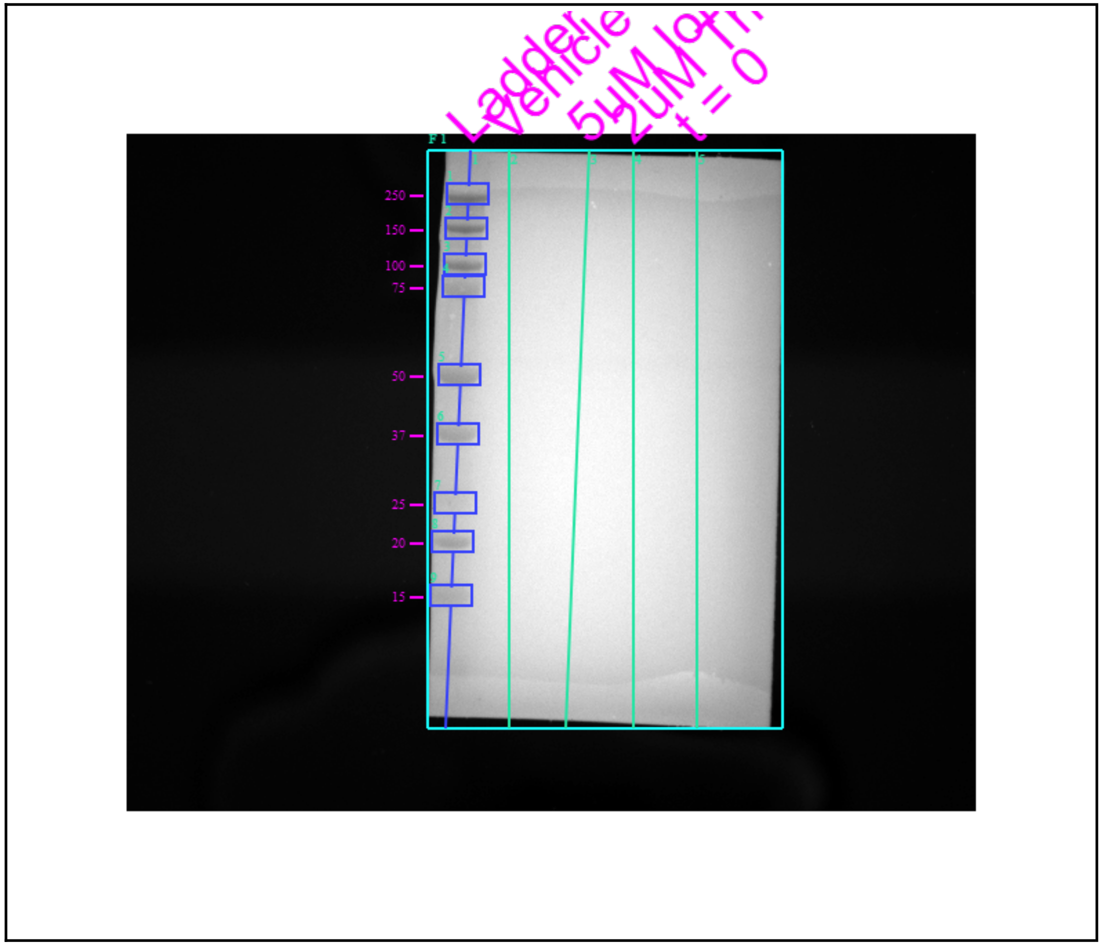

CHEMI_03202022_142709

Date:	20 March 2022 02:27:09PM
Mode:	Chemi Blots
Notes:	
Model:	FL1500
Instrument name:	2462619090234
Serial No:	2462619090234
Firmware version:	1.6.0
iBA version:	5.0
Image size:	615px X 491px
Image area:	112.7mm X 90.16mm
Optical Zoom:	2x
Digital Zoom:	1.1x
Focus level:	455
Resolution:	5 x 5
Exposure time:	44304 ms
Exposure mode:	Normal

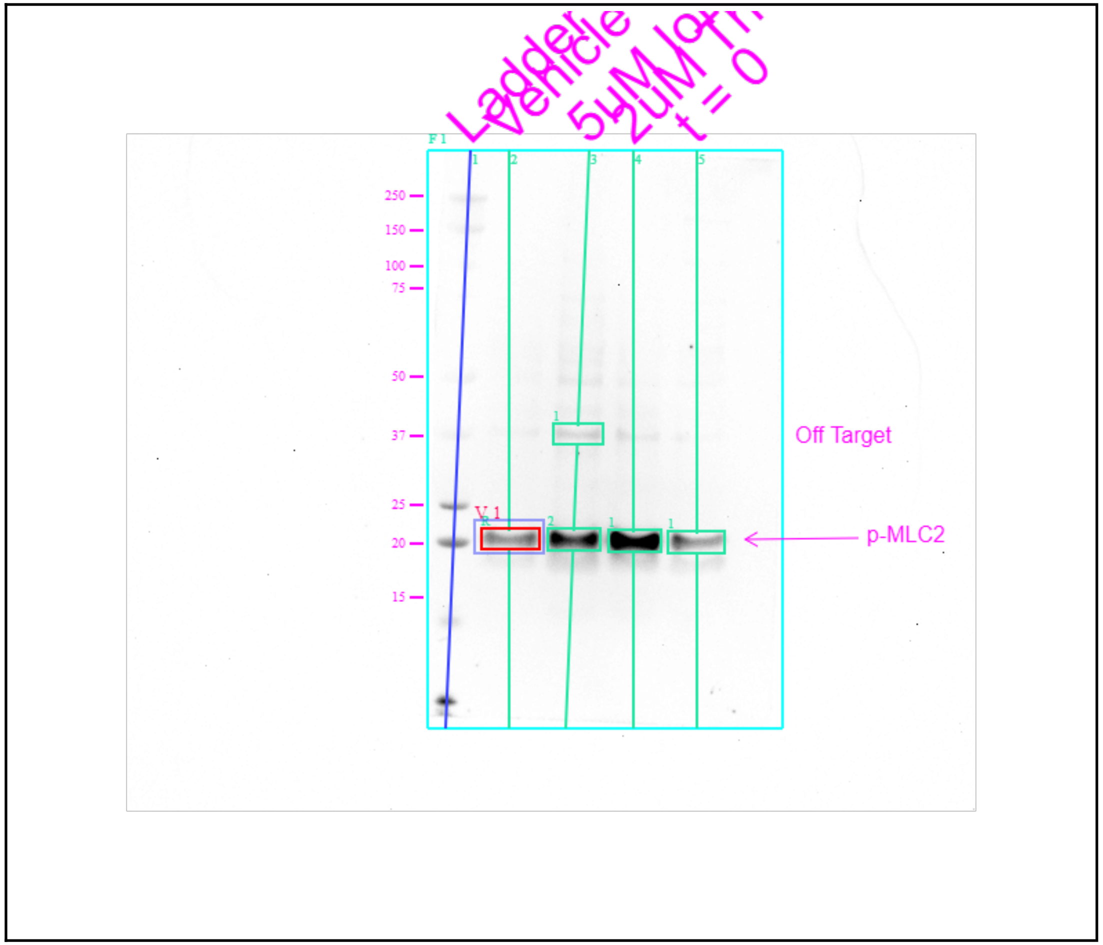

LANE AND BAND ANALYSIS DATA TABLE

CHEMI_03202022_142709

Frame: 1
Channel: Membrane
Sensitivity: 100
Molecular Weight Analysis Regression Method : Point to Point

Lane 1 - Ladder

#	Vol. (Int.)	Local Bg. Corr. Vol.	Area	Rf	Density	Local Bg. Corr. Den.	% band purity	% lane purity	Mol. Wt.
1	18,554,548	895,090	496	0.074	37,408	1,804.618	13.429	3.926	250
2	17,974,815	1,445,825	496	0.134	36,239	2,914.97	21.692	3.804	150
3	16,945,044	1,253,121	496	0.196	34,163	2,526.454	18.801	3.586	100
4	16,164,552	728,319	496	0.234	32,589	1,468.387	10.927	3.421	75
5	16,114,111	1,507,154	496	0.387	32,488	3,038.618	22.612	3.41	50
6	15,171,675	696,803	496	0.489	30,588	1,404.845	10.454	3.21	37
7	13,792,962	237.309	496	0.609	27,808	0.478	0.004	2.919	25
8	15,123,870	34,999	496	0.675	30,491	70.564	0.525	3.2	20
9	15,539,330	103,708	496	0.768	31,329	209.089	1.556	3.288	15

Frame: 1
Channel: Chemi
Sensitivity: 100
Molecular Weight Analysis Regression Method : Point to Point

Lane 2 - Vehicle

#	Vol. (Int.)	Local Bg. Corr. Vol.	Area	Rf	Density	Local Bg. Corr. Den.	% band purity	% lane purity	Mol. Wt.	Rel. Quant. (w/ LB Corr. Vol.)
1	4,850,583	3,451,135	688	0.671	7,050.266	5,016.185	100	34.149	20.357	1

Lane 3 - 5uM Ionomycin

#	Vol. (Int.)	Local Bg. Corr. Vol.	Area	Rf	Density	Local Bg. Corr. Den.	% band purity	% lane purity	Mol. Wt.	Rel. Quant. (w/ LB Corr. Vol.)
1	1,705,524	701,531	592	0.489	2,880.953	1,185.019	8.934	6.292	37	0.203
2	9,831,111	7,151,072	663	0.673	14,828	10,785	91.066	36.267	20.179	2.072

Lane 4 - 2uM Thapsigargin

#	Vol. (Int.)	Local Bg. Corr. Vol.	Area	Rf	Density	Local Bg. Corr. Den.	% band purity	% lane purity	Mol. Wt.	Rel. Quant. (w/ LB Corr. Vol.)
1	14,548,279	11,826,735	663	0.675	21,943	17,838	100	52.944	20	3.427

Lane 5 - t = 0

#	Vol. (Int.)	Local Bg. Corr. Vol.	Area	Rf	Density	Local Bg. Corr. Den.	% band purity	% lane purity	Mol. Wt.	Rel. Quant. (w/ LB Corr. Vol.)
1	4,553,663	3,372,900	714	0.678	6,377.679	4,723.95	100	37.299	19.872	0.977

REGION ANALYSIS DATA TABLE

CHEMI_03202022_142709
Chemi

#	Ref. name	Vol. (Int.)	Local Bg. Corr. Vol.	Median Intensity	Area	Density	Local Bg. Corr. Den.	Purity
1	V 1	5,921,170	3,297,075	2,830	1,275	4,644.055	2,585.942	1

iBright™ Image Analysis Report

Katarina+ Chang

18 November 2022

CHEMI_03252022_113001

Date:	25 March 2022 11:30:01AM
Mode:	Chemi Blots
Notes:	
Model:	FL1500
Instrument name:	2462619090234
Serial No:	2462619090234
Firmware version:	1.6.0
iBA version:	5.0
Image size:	520px X 415px
Image area:	112.7mm X 90.16mm
Optical Zoom:	2x
Digital Zoom:	1.3x
Focus level:	455
Resolution:	5 x 5
Exposure time:	1411 ms
Exposure mode:	Normal

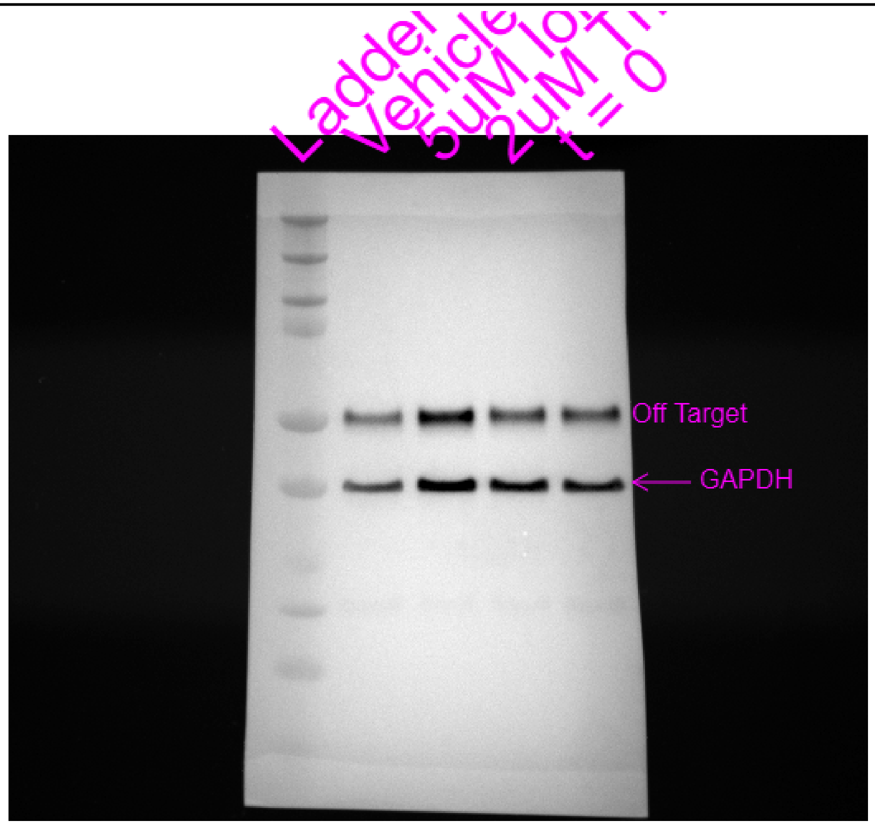

CHEMI_03252022_113001

Date:	25 March 2022 11:30:01AM
Mode:	Chemi Blots
Notes:	
Model:	FL1500
Instrument name:	2462619090234
Serial No:	2462619090234
Firmware version:	1.6.0
iBA version:	5.0
Image size:	520px X 415px
Image area:	112.7mm X 90.16mm
Optical Zoom:	2x
Digital Zoom:	1.3x
Focus level:	455
Resolution:	5 x 5
Exposure time:	1411 ms
Exposure mode:	Normal

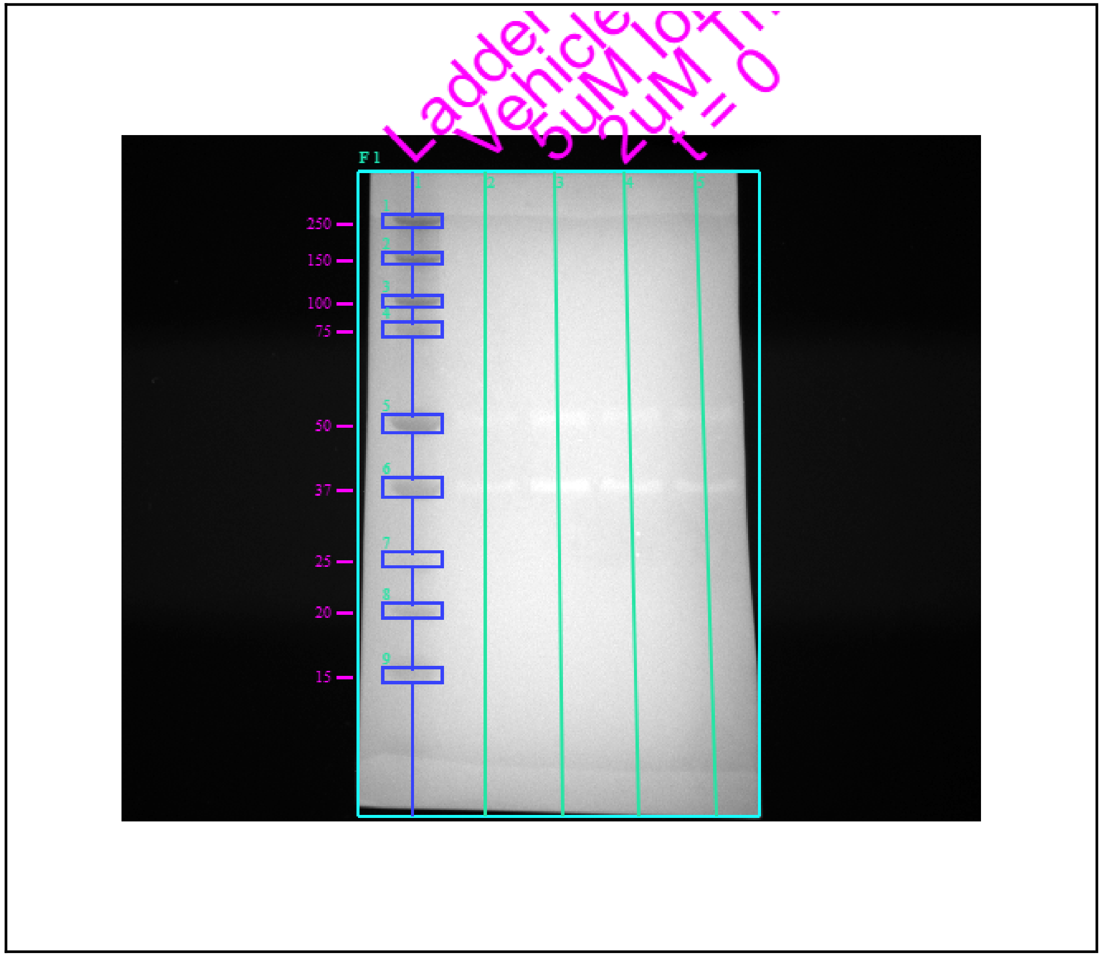

CHEMI_03252022_113001

Date:	25 March 2022 11:30:01AM
Mode:	Chemi Blots
Notes:	
Model:	FL1500
Instrument name:	2462619090234
Serial No:	2462619090234
Firmware version:	1.6.0
iBA version:	5.0
Image size:	520px X 415px
Image area:	112.7mm X 90.16mm
Optical Zoom:	2x
Digital Zoom:	1.3x
Focus level:	455
Resolution:	5 x 5
Exposure time:	1411 ms
Exposure mode:	Normal

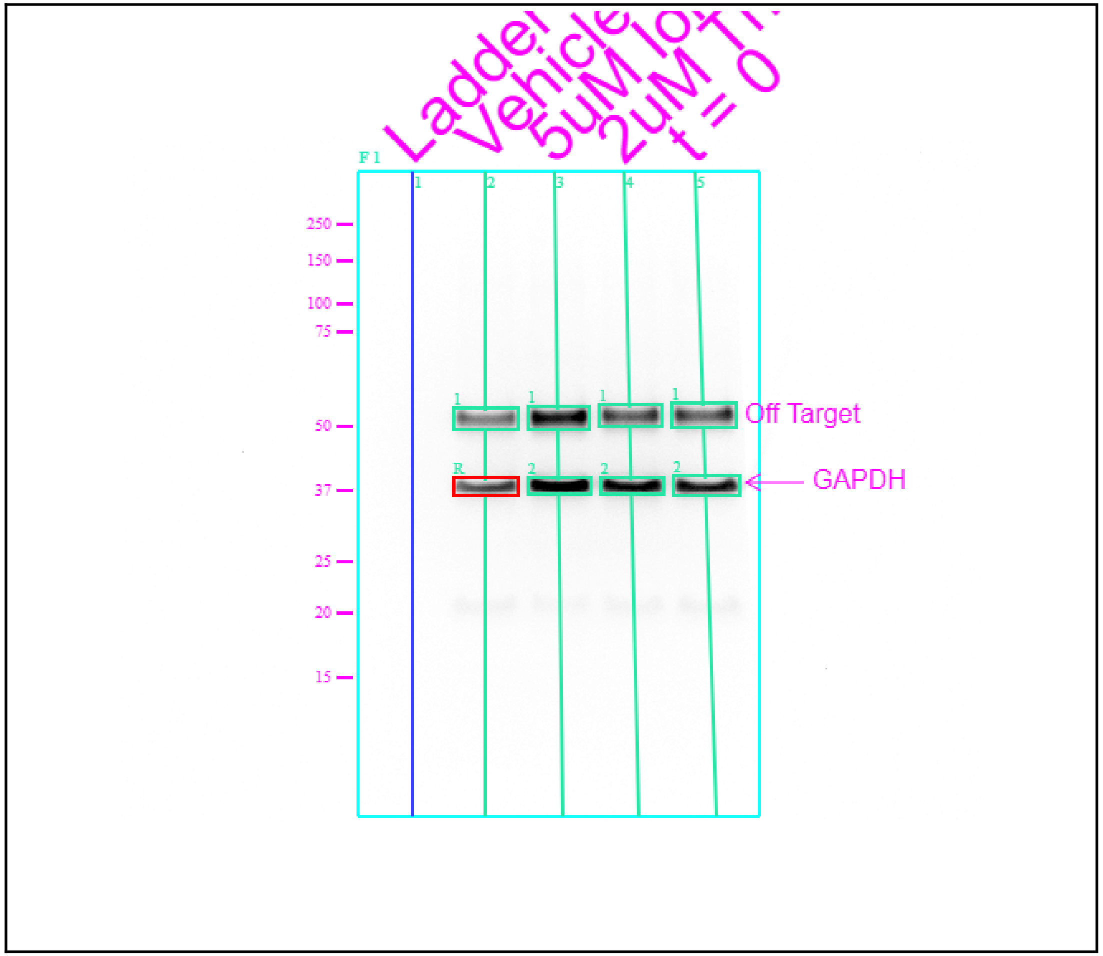

LANE AND BAND ANALYSIS DATA TABLE

CHEMI_03252022_113001

Frame: 1
Channel: Membrane
Sensitivity: 100
Molecular Weight Analysis Regression Method : Point to Point

Lane 1 - Ladder

#	Vol. (Int.)	Local Bg. Corr. Vol.	Area	Rf	Density	Local Bg. Corr. Den.	% band purity	% lane purity	Rolling Bg. Corr. Vol.	Rolling Bg. Corr. Den.	Mol. Wt.
1	12,533,239	1,776,564	333	0.077	37,637	5,335.027	16.178	3.39	1,903,104	5,715.027	250
2	10,531,945	1,432,086	296	0.133	35,580	4,838.131	10.705	2.243	1,259,264	4,254.27	150
3	9,994,453	1,450,755	296	0.2	33,765	4,901.199	11.371	2.383	1,337,600	4,518.919	100
4	11,356,453	1,094,407	370	0.244	30,693	2,957.858	8.159	1.71	959,744	2,593.903	75
5	13,608,766	1,983,925	444	0.39	30,650	4,468.3	18.259	3.826	2,147,840	4,837.477	50
6	13,829,460	1,704,603	481	0.49	28,751	3,543.875	15.064	3.157	1,772,032	3,684.058	37
7	9,598,510	467,985	370	0.6	25,941	1,264.826	4.812	1.008	566,016	1,529.773	25
8	10,257,452	1,011,411	370	0.679	27,722	2,733.544	8.355	1.751	982,784	2,656.173	20
9	10,412,371	734,592	370	0.779	28,141	1,985.386	7.099	1.488	835,072	2,256.951	15

Frame: 1
Channel: Chemi
Sensitivity: 100
Molecular Weight Analysis Regression Method : Point to Point

Lane 2 - Vehicle

#	Vol. (Int.)	Local Bg. Corr. Vol.	Area	Rf	Density	Local Bg. Corr. Den.
1	6,261,348	4,840,435	560	0.382	11,180	8,643.634
2	6,374,822	5,203,347	480	0.487	13,280	10,840

#	% band purity	% lane purity	Rolling Bg. Corr. Vol.	Rolling Bg. Corr. Den.	Mol. Wt.	Rel. Quant. (w/ LB Corr. Vol.)
1	48.811	42.16	5,260,544	9,393.829	51.316	0.93
2	51.189	44.213	5,516,800	11,493	37.333	1

Lane 3 - 5uM Ionomycin

#	Vol. (Int.)	Local Bg. Corr. Vol.	Area	Rf	Density	Local Bg. Corr. Den.

#	Vol. (Int.)	Local Bg. Corr. Vol.	Area	Rf	Density	Local Bg. Corr. Den.
1	11,242,132	9,336,247	570	0.382	19,723	16,379
2	10,941,207	9,238,061	429	0.487	25,503	21,533

#	% band purity	% lane purity	Rolling Bg. Corr. Vol.	Rolling Bg. Corr. Den.	Mol. Wt.	Rel. Quant. (w/ LB Corr. Vol.)
1	50.531	45.511	10,076,416	17,677	51.316	1.794
2	49.469	44.555	9,864,704	22,994	37.333	1.775

Lane 4 - 2uM Thapsigargin

#	Vol. (Int.)	Local Bg. Corr. Vol.	Area	Rf	Density	Local Bg. Corr. Den.
1	8,116,494	6,340,664	546	0.377	14,865	11,612
2	9,198,399	7,470,742	429	0.487	21,441	17,414

#	% band purity	% lane purity	Rolling Bg. Corr. Vol.	Rolling Bg. Corr. Den.	Mol. Wt.	Rel. Quant. (w/ LB Corr. Vol.)
1	46.154	39.628	7,089,152	12,983	52.193	1.219
2	53.846	46.232	8,270,592	19,278	37.333	1.436

Lane 5 - t = 0

#	Vol. (Int.)	Local Bg. Corr. Vol.	Area	Rf	Density	Local Bg. Corr. Den.
1	8,559,832	7,142,745	640	0.377	13,374	11,160
2	8,886,728	7,629,139	533	0.487	16,673	14,313

#	% band purity	% lane purity	Rolling Bg. Corr. Vol.	Rolling Bg. Corr. Den.	Mol. Wt.	Rel. Quant. (w/ LB Corr. Vol.)
1	48.778	44.755	7,666,944	11,979	52.193	1.373
2	51.222	46.997	8,050,944	15,104	37.333	1.466

iBright™ Image Analysis Report

Katarina+ Chang

18 November 2022

CHEMI_03232022_132434

Date:	23 March 2022 01:24:34PM
Mode:	Chemi Blots
Notes:	
Model:	FL1500
Instrument name:	2462619090234
Serial No:	2462619090234
Firmware version:	1.6.0
iBA version:	5.0
Image size:	520px X 415px
Image area:	112.7mm X 90.16mm
Optical Zoom:	2x
Digital Zoom:	1.3x
Focus level:	455
Resolution:	5 x 5
Exposure time:	26687 ms
Exposure mode:	Normal

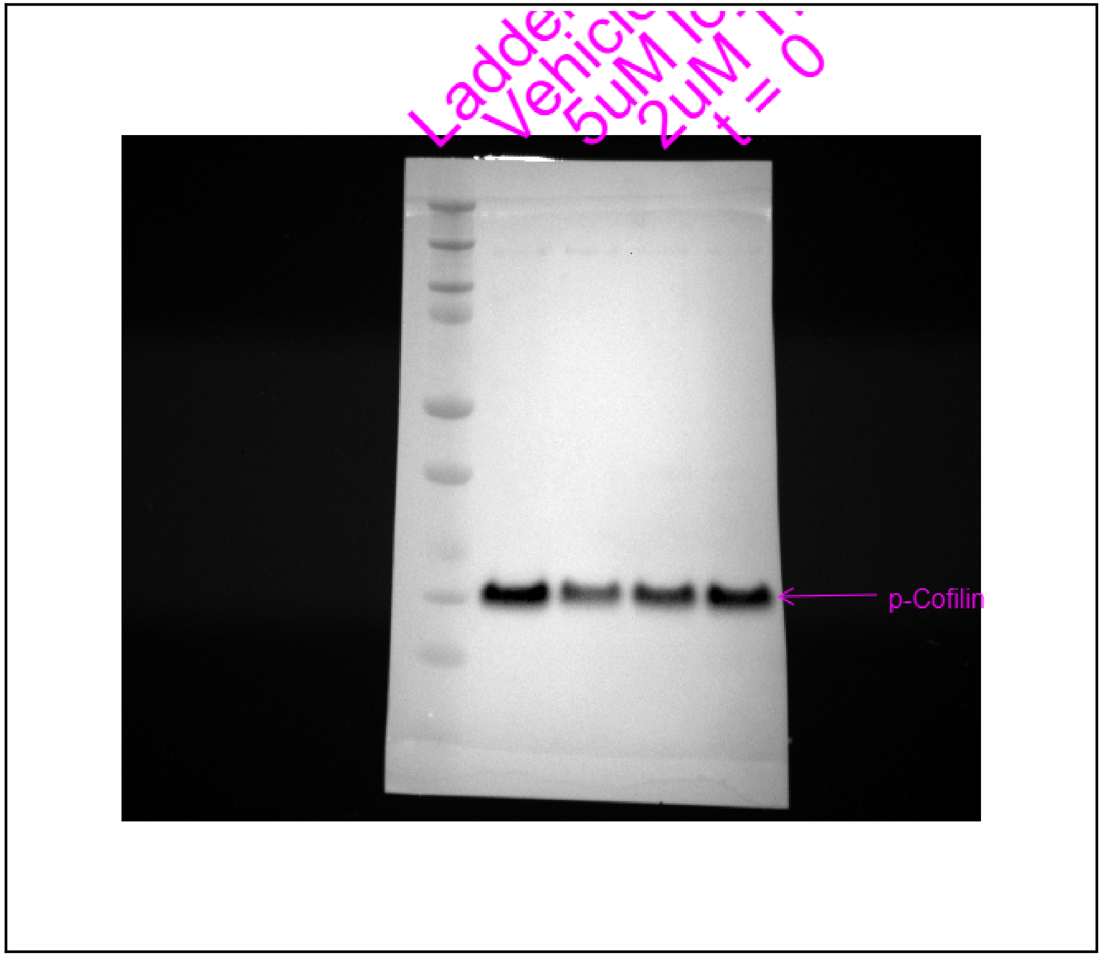

CHEMI_03232022_132434

Date:	23 March 2022 01:24:34PM
Mode:	Chemi Blots
Notes:	
Model:	FL1500
Instrument name:	2462619090234
Serial No:	2462619090234
Firmware version:	1.6.0
iBA version:	5.0
Image size:	520px X 415px
Image area:	112.7mm X 90.16mm
Optical Zoom:	2x
Digital Zoom:	1.3x
Focus level:	455
Resolution:	5 x 5
Exposure time:	26687 ms
Exposure mode:	Normal

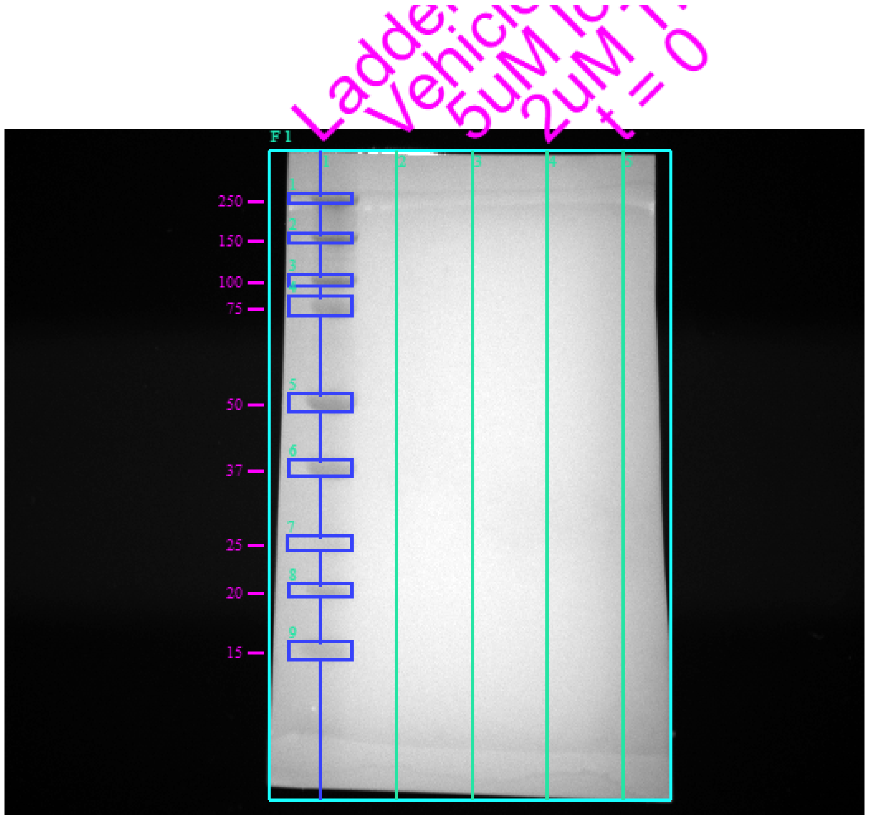

CHEMI_03232022_132434

Date:	23 March 2022 01:24:34PM
Mode:	Chemi Blots
Notes:	
Model:	FL1500
Instrument name:	2462619090234
Serial No:	2462619090234
Firmware version:	1.6.0
iBA version:	5.0
Image size:	520px X 415px
Image area:	112.7mm X 90.16mm
Optical Zoom:	2x
Digital Zoom:	1.3x
Focus level:	455
Resolution:	5 x 5
Exposure time:	26687 ms
Exposure mode:	Normal

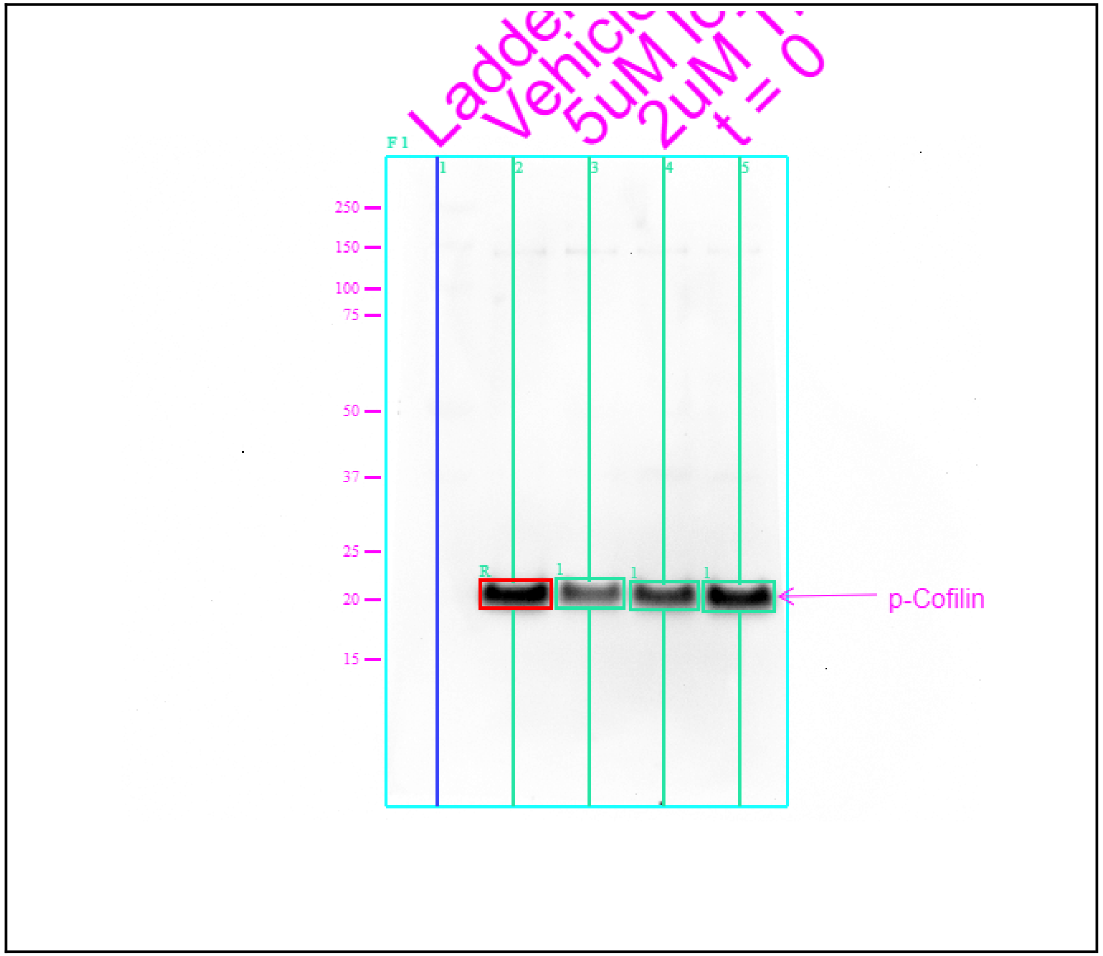

LANE AND BAND ANALYSIS DATA TABLE

CHEMI_03232022_132434

Frame: 1
Channel: Membrane
Sensitivity: 100
Molecular Weight Analysis Regression Method : Point to Point

Lane 1 - Ladder

#	Vol. (Int.)	Local Bg. Corr. Vol.	Area	Rf	Density	Local Bg. Corr. Den.	% band purity	% lane purity	Rolling Bg. Corr. Vol.	Rolling Bg. Corr. Den.	Mol. Wt.
1	10,053,915	418,163	273	0.074	36,827	1,531.736	10.578	1.527	1,220,096	4,469.216	250
2	9,809,161	638,463	273	0.135	35,930	2,338.692	10.096	1.457	1,164,544	4,265.729	150
3	10,622,935	730,957	312	0.198	34,047	2,342.814	10.942	1.58	1,262,080	4,045.128	100
4	15,899,401	370,270	507	0.239	31,359	730.317	10.485	1.514	1,209,344	2,385.294	75
5	14,571,994	1,559,425	468	0.387	31,136	3,332.107	19.647	2.836	2,266,112	4,842.12	50
6	12,634,015	1,460,955	429	0.489	29,449	3,405.492	15.07	2.176	1,738,240	4,051.841	37
7	10,593,555	515,942	400	0.603	26,483	1,289.856	3.742	0.54	431,616	1,079.04	25
8	9,938,286	1,042,408	351	0.677	28,314	2,969.824	9.162	1.323	1,056,768	3,010.735	20
9	13,296,568	1,138,702	468	0.768	28,411	2,433.124	10.278	1.484	1,185,536	2,533.197	15

Frame: 1
Channel: Chemi
Sensitivity: 100
Molecular Weight Analysis Regression Method : Point to Point

Lane 2 - Vehicle

#	Vol. (Int.)	Local Bg. Corr. Vol.	Area	Rf	Density	Local Bg. Corr. Den.
1	22,313,841	17,514,030	792	0.672	28,174	22,113

#	% band purity	% lane purity	Rolling Bg. Corr. Vol.	Rolling Bg. Corr. Den.	Mol. Wt.	Rel. Quant. (w/ LB Corr. Vol.)
1	100	91.032	19,926,272	25,159	20.345	1

Lane 3 - 5uM Ionomycin

#	Vol. (Int.)	Local Bg. Corr. Vol.	Area	Rf	Density	Local Bg. Corr. Den.
1	13,830,703	9,233,984	798	0.672	17,331	11,571

#	% band purity	% lane purity	Rolling Bg. Corr. Vol.	Rolling Bg. Corr. Den.	Mol. Wt.	Rel. Quant. (w/ LB Corr. Vol.)

#	% band purity	% lane purity	Rolling Bg. Corr. Vol.	Rolling Bg. Corr. Den.	Mol. Wt.	Rel. Quant. (w/ LB Corr. Vol.)
1	100	82.655	11,390,464	14,273	20.345	0.527

Lane 4 - 2uM Thapsigargin

#	Vol. (Int.)	Local Bg. Corr. Vol.	Area	Rf	Density	Local Bg. Corr. Den.	
1	16,924,810	11,476,364	756	0.674	22,387	15,180	

#	% band purity	% lane purity	Rolling Bg. Corr. Vol.	Rolling Bg. Corr. Den.	Mol. Wt.	Rel. Quant. (w/ LB Corr. Vol.)
1	100	80.21	13,817,600	18,277	20.172	0.655

Lane 5 - t = 0

#	Vol. (Int.)	Local Bg. Corr. Vol.	Area	Rf	Density	Local Bg. Corr. Den.	
1	21,320,223	16,420,546	836	0.677	25,502	19,641	

#	% band purity	% lane purity	Rolling Bg. Corr. Vol.	Rolling Bg. Corr. Den.	Mol. Wt.	Rel. Quant. (w/ LB Corr. Vol.)
1	100	85.132	18,514,176	22,146	20	0.938

iBright™ Image Analysis Report

Katarina+ Chang

18 November 2022

CHEMI_03262022_153757

Date:	26 March 2022 03:37:57PM
Mode:	Chemi Blots
Notes:	
Model:	FL1500
Instrument name:	2462619090234
Serial No:	2462619090234
Firmware version:	1.6.0
iBA version:	5.0
Image size:	676px X 540px
Image area:	112.7mm X 90.16mm
Optical Zoom:	2x
Digital Zoom:	1x
Focus level:	455
Resolution:	5 x 5
Exposure time:	15607 ms
Exposure mode:	Normal

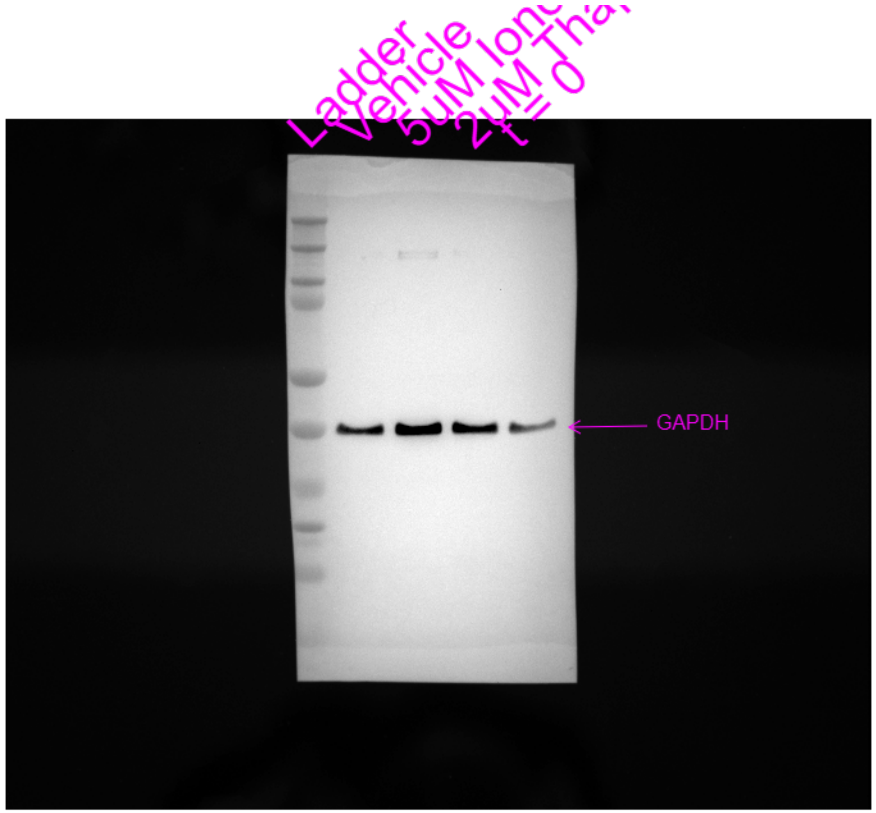

CHEMI_03262022_153757

Date:	26 March 2022 03:37:57PM
Mode:	Chemi Blots
Notes:	
Model:	FL1500
Instrument name:	2462619090234
Serial No:	2462619090234
Firmware version:	1.6.0
iBA version:	5.0
Image size:	676px X 540px
Image area:	112.7mm X 90.16mm
Optical Zoom:	2x
Digital Zoom:	1x
Focus level:	455
Resolution:	5 x 5
Exposure time:	15607 ms
Exposure mode:	Normal

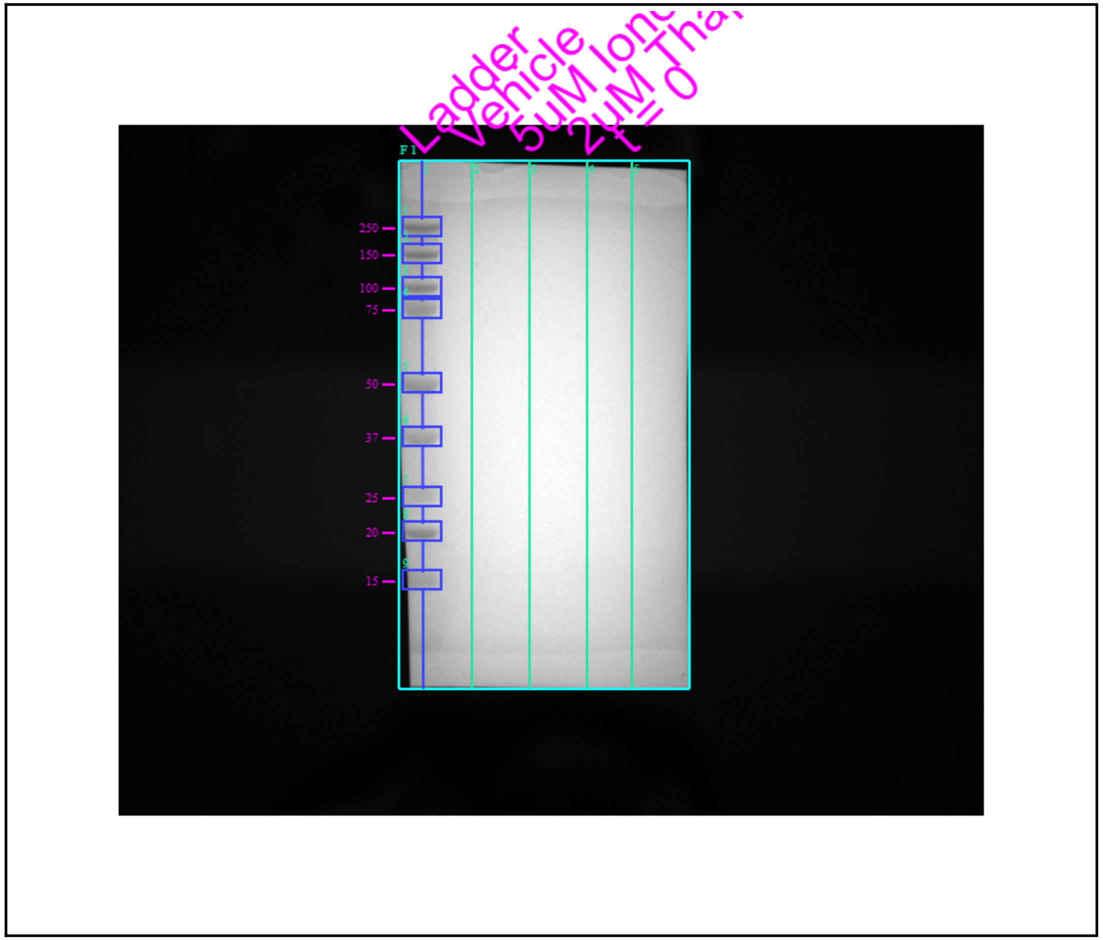

CHEMI_03262022_153757

Date:	26 March 2022 03:37:57PM
Mode:	Chemi Blots
Notes:	
Model:	FL1500
Instrument name:	2462619090234
Serial No:	2462619090234
Firmware version:	1.6.0
iBA version:	5.0
Image size:	676px X 540px
Image area:	112.7mm X 90.16mm
Optical Zoom:	2x
Digital Zoom:	1x
Focus level:	455
Resolution:	5 x 5
Exposure time:	15607 ms
Exposure mode:	Normal

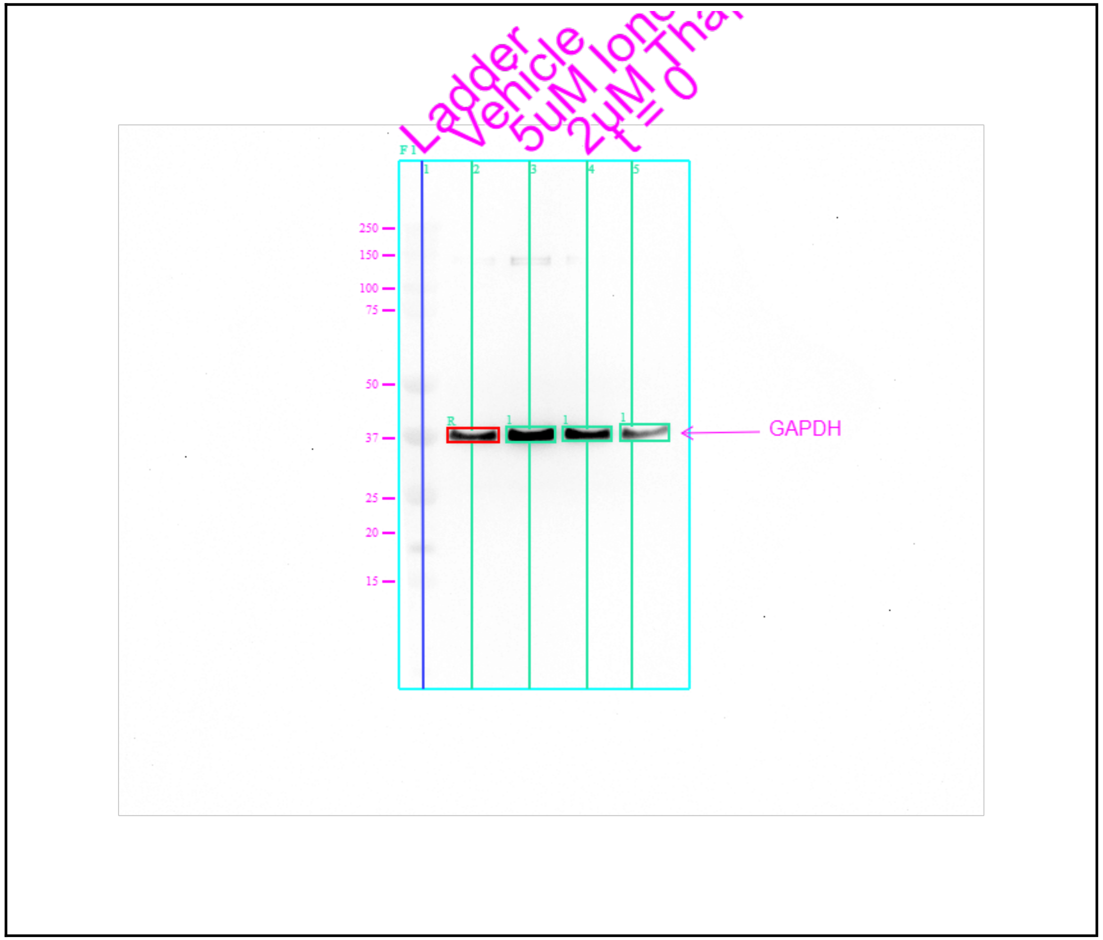

LANE AND BAND ANALYSIS DATA TABLE

CHEMI_03262022_153757

Frame: 1
Channel: Membrane
Sensitivity: 100
Molecular Weight Analysis Regression Method : Point to Point

Lane 1 - Ladder

#	Vol. (Int.)	Local Bg. Corr. Vol.	Area	Rf	Density	Local Bg. Corr. Den.	% band purity	% lane purity	Mol. Wt.
1	18,301,418	766,621	496	0.123	36,898	1,545.608	10.491	3.616	250
2	17,732,873	893,378	496	0.174	35,751	1,801.167	12.226	3.503	150
3	16,902,633	1,040,932	496	0.237	34,077	2,098.655	14.245	3.339	100
4	16,436,987	1,064,610	496	0.278	33,139	2,146.393	14.569	3.247	75
5	16,171,673	945,907	496	0.419	32,604	1,907.072	12.945	3.195	50
6	15,711,016	405,571	496	0.521	31,675	817.684	5.55	3.104	37
7	15,258,802	37,649	496	0.634	30,763	75.906	0.515	3.015	25
8	17,148,212	787,222	496	0.7	34,573	1,587.141	10.773	3.388	20
9	18,421,729	1,365,368	496	0.792	37,140	2,752.759	18.685	3.639	15

Frame: 1
Channel: Chemi
Sensitivity: 100
Molecular Weight Analysis Regression Method : Point to Point

Lane 2 - Vehicle

#	Vol. (Int.)	Local Bg. Corr. Vol.	Area	Rf	Density	Local Bg. Corr. Den.	% band purity	% lane purity	Mol. Wt.	Rel. Quant. (w/ LB Corr. Vol.)
1	6,966,087	5,897,708	492	0.518	14,158	11,987	100	50.552	37.31	1

Lane 3 - 5uM Ionomycin

#	Vol. (Int.)	Local Bg. Corr. Vol.	Area	Rf	Density	Local Bg. Corr. Den.	% band purity	% lane purity	Mol. Wt.	Rel. Quant. (w/ LB Corr. Vol.)
1	12,803,699	10,892,693	507	0.518	25,253	21,484	100	57.275	37.31	1.847

Lane 4 - 2uM Thapsigargin

#	Vol. (Int.)	Local Bg. Corr. Vol.	Area	Rf	Density	Local Bg. Corr. Den.	% band purity	% lane purity	Mol. Wt.	Rel. Quant. (w/ LB Corr. Vol.)

#	Vol. (Int.)	Local Bg. Corr. Vol.	Area	Rf	Density	Local Bg. Corr. Den.	% band purity	% lane purity	Mol. Wt.	Rel. Quant. (w/ LB Corr. Vol.)
1	8,736,394	7,487,142	468	0.516	18,667	15,998	100	56.16	37.619	1.27

Lane 5 - t = 0

#	Vol. (Int.)	Local Bg. Corr. Vol.	Area	Rf	Density	Local Bg. Corr. Den.	% band purity	% lane purity	Mol. Wt.	Rel. Quant. (w/ LB Corr. Vol.)
1	4,145,703	3,722,406	546	0.513	7,592.863	6,817.594	100	52.419	37.929	0.631

iBright™ Image Analysis Report

Katarina+ Chang

18 November 2022

CHEMI_03252022_112519

Date:	25 March 2022 11:25:19AM
Mode:	Chemi Blots
Notes:	
Model:	FL1500
Instrument name:	2462619090234
Serial No:	2462619090234
Firmware version:	1.6.0
iBA version:	5.0
Image size:	563px X 450px
Image area:	112.7mm X 90.16mm
Optical Zoom:	2x
Digital Zoom:	1.2x
Focus level:	455
Resolution:	5 x 5
Exposure time:	4748 ms
Exposure mode:	Normal

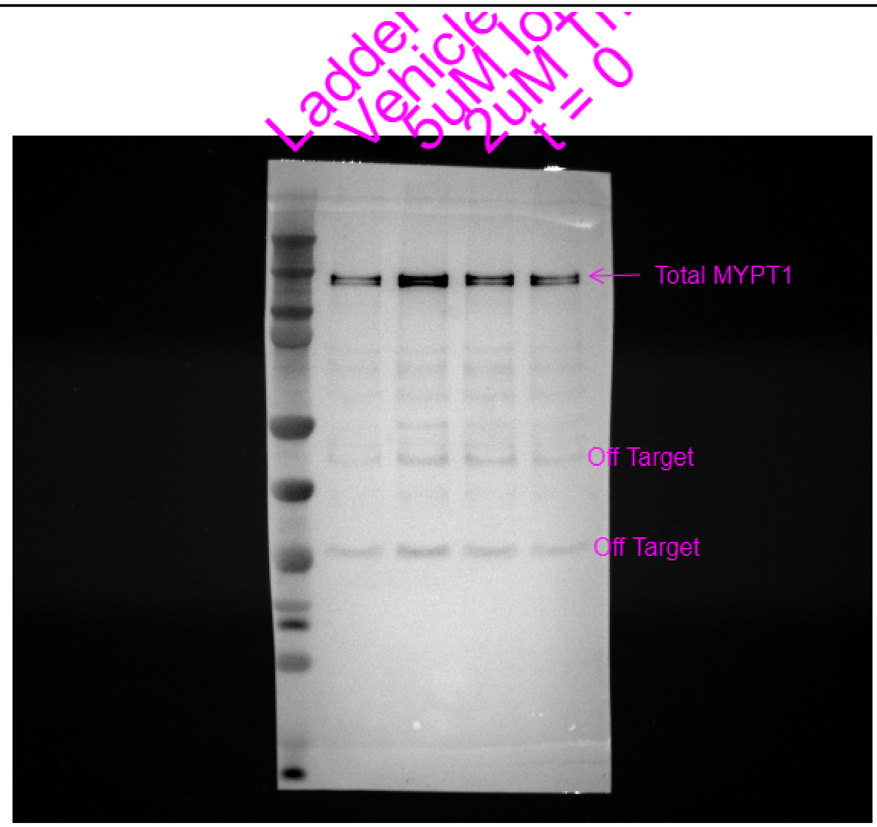

CHEMI_03252022_112519

Date:	25 March 2022 11:25:19AM
Mode:	Chemi Blots
Notes:	
Model:	FL1500
Instrument name:	2462619090234
Serial No:	2462619090234
Firmware version:	1.6.0
iBA version:	5.0
Image size:	563px X 450px
Image area:	112.7mm X 90.16mm
Optical Zoom:	2x
Digital Zoom:	1.2x
Focus level:	455
Resolution:	5 x 5
Exposure time:	4748 ms
Exposure mode:	Normal

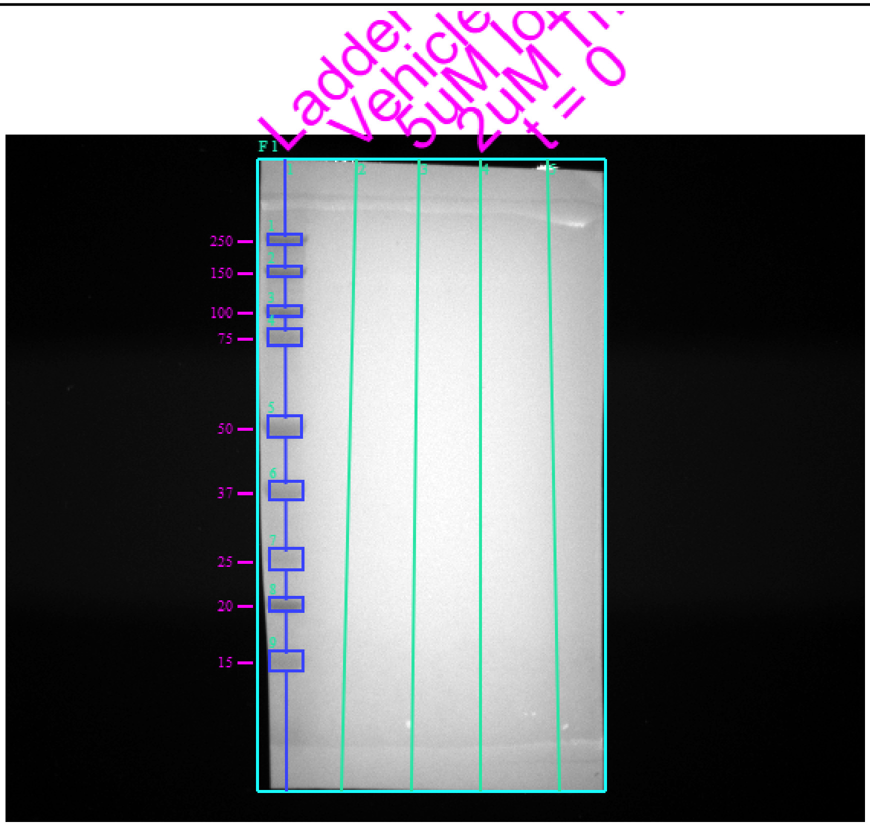

CHEMI_03252022_112519

Date:	25 March 2022 11:25:19AM
Mode:	Chemi Blots
Notes:	
Model:	FL1500
Instrument name:	2462619090234
Serial No:	2462619090234
Firmware version:	1.6.0
iBA version:	5.0
Image size:	563px X 450px
Image area:	112.7mm X 90.16mm
Optical Zoom:	2x
Digital Zoom:	1.2x
Focus level:	455
Resolution:	5 x 5
Exposure time:	4748 ms
Exposure mode:	Normal

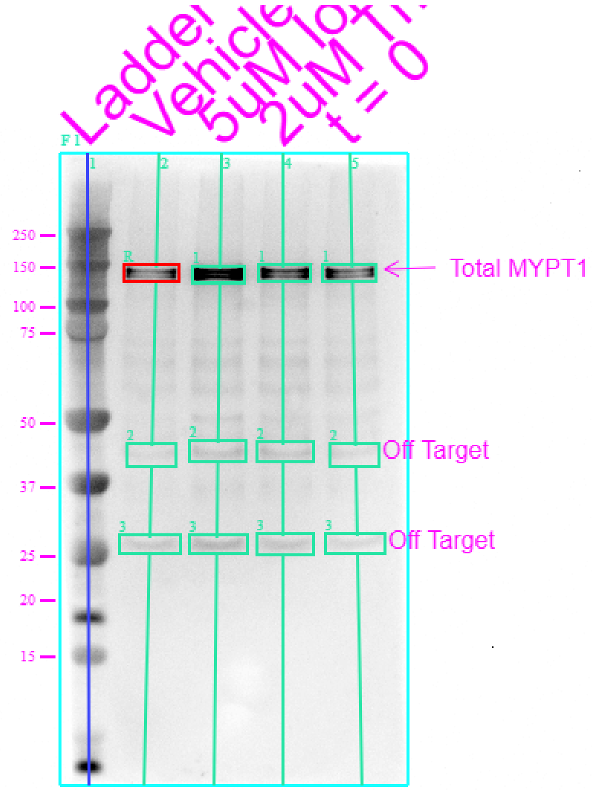

LANE AND BAND ANALYSIS DATA TABLE

CHEMI_03252022_112519

Frame: 1
Channel: Membrane
Sensitivity: 100
Molecular Weight Analysis Regression Method : Point to Point

Lane 1 - Ladder

#	Vol. (Int.)	Local Bg. Corr. Vol.	Area	Rf	Density	Local Bg. Corr. Den.	% band purity	% lane purity	Mol. Wt.
1	7,212,610	1,027,312	184	0.126	39,198	5,583.217	10.43	2.402	250
2	7,095,574	1,148,866	184	0.176	38,562	6,243.842	11.664	2.363	150
3	6,888,875	1,184,686	184	0.239	37,439	6,438.514	12.027	2.294	100
4	9,618,348	1,385,412	276	0.28	34,849	5,019.612	14.065	3.203	75
5	11,687,006	1,859,065	345	0.423	33,875	5,388.595	18.874	3.892	50
6	9,968,978	1,454,122	299	0.524	33,341	4,863.287	14.763	3.32	37
7	10,940,175	795,855	345	0.633	31,710	2,306.828	8.08	3.644	25
8	8,546,596	994,174	230	0.703	37,159	4,322.498	10.093	2.846	20
9	11,311,178	382.828	322	0.792	35,127	1.189	0.004	3.767	15

Frame: 1
Channel: Chemi
Sensitivity: 100
Molecular Weight Analysis Regression Method : Point to Point

Lane 2 - Vehicle

#	Vol. (Int.)	Local Bg. Corr. Vol.	Area	Rf	Density	Local Bg. Corr. Den.	% band purity	% lane purity	Mol. Wt.	Rel. Quant. (w/ LB Corr. Vol.)
1	4,610,513	2,572,359	444	0.188	10,384	5,793.603	78.709	15.921	140.385	1
2	1,867,223	184,983	528	0.476	3,536.407	350.347	5.66	6.448	43.19	0.072
3	2,057,462	510,831	533	0.618	3,860.154	958.409	15.63	7.105	26.6	0.199

Lane 3 - 5uM Ionomycin

#	Vol. (Int.)	Local Bg. Corr. Vol.	Area	Rf	Density	Local Bg. Corr. Den.	% band purity	% lane purity	Mol. Wt.	Rel. Quant. (w/ LB Corr. Vol.)
1	8,841,872	6,360,984	420	0.191	21,052	15,145	81.741	26.934	138.462	2.473
2	2,598,259	534,833	570	0.471	4,558.349	938.304	6.873	7.915	43.81	0.208

#	Vol. (Int.)	Local Bg. Corr. Vol.	Area	Rf	Density	Local Bg. Corr. Den.	% band purity	% lane purity	Mol. Wt.	Rel. Quant. (w/ LB Corr. Vol.)
3	2,472,743	886,053	507	0.618	4,877.205	1,747.64	11.386	7.533	26.6	0.344

Lane 4 - 2uM Thapsigargin

#	Vol. (Int.)	Local Bg. Corr. Vol.	Area	Rf	Density	Local Bg. Corr. Den.	% band purity	% lane purity	Mol. Wt.	Rel. Quant. (w/ LB Corr. Vol.)
1	5,796,069	3,654,266	420	0.188	13,800	8,700.635	76.173	19.351	140.385	1.421
2	2,402,247	478,513	532	0.471	4,515.502	899.461	9.975	8.02	43.81	0.186
3	2,260,723	664,545	570	0.618	3,966.181	1,165.869	13.852	7.548	26.6	0.258

Lane 5 - t = 0

#	Vol. (Int.)	Local Bg. Corr. Vol.	Area	Rf	Density	Local Bg. Corr. Den.	% band purity	% lane purity	Mol. Wt.	Rel. Quant. (w/ LB Corr. Vol.)
1	4,802,816	3,042,870	432	0.188	11,117	7,043.681	81.134	19.807	140.385	1.183
2	1,626,099	277,250	528	0.476	3,079.733	525.097	7.392	6.706	43.19	0.108
3	1,528,255	430,324	560	0.616	2,729.027	768.437	11.474	6.302	26.867	0.167

iBright™ Image Analysis Report

Katarina+ Chang

18 November 2022

CHEMI_03242022_150645

Date:	24 March 2022 03:06:45PM
Mode:	Chemi Blots
Notes:	
Model:	FL1500
Instrument name:	2462619090234
Serial No:	2462619090234
Firmware version:	1.6.0
iBA version:	5.0
Image size:	615px X 491px
Image area:	112.7mm X 90.16mm
Optical Zoom:	2x
Digital Zoom:	1.1x
Focus level:	455
Resolution:	5 x 5
Exposure time:	1142 ms
Exposure mode:	Normal

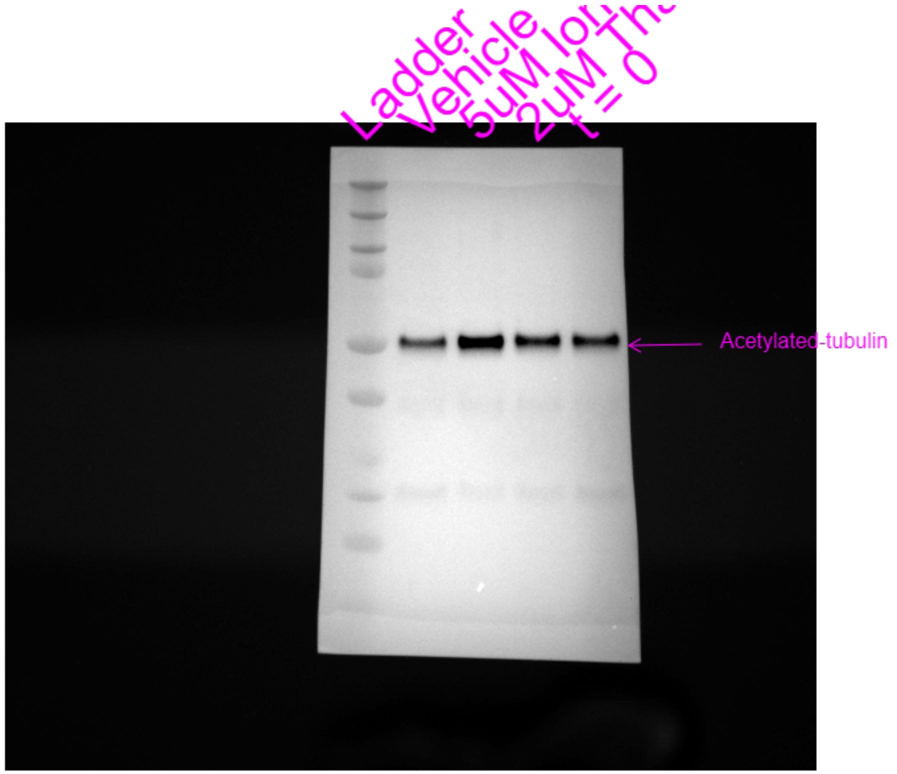

CHEMI_03242022_150645

Date:	24 March 2022 03:06:45PM
Mode:	Chemi Blots
Notes:	
Model:	FL1500
Instrument name:	2462619090234
Serial No:	2462619090234
Firmware version:	1.6.0
iBA version:	5.0
Image size:	615px X 491px
Image area:	112.7mm X 90.16mm
Optical Zoom:	2x
Digital Zoom:	1.1x
Focus level:	455
Resolution:	5 x 5
Exposure time:	1142 ms
Exposure mode:	Normal

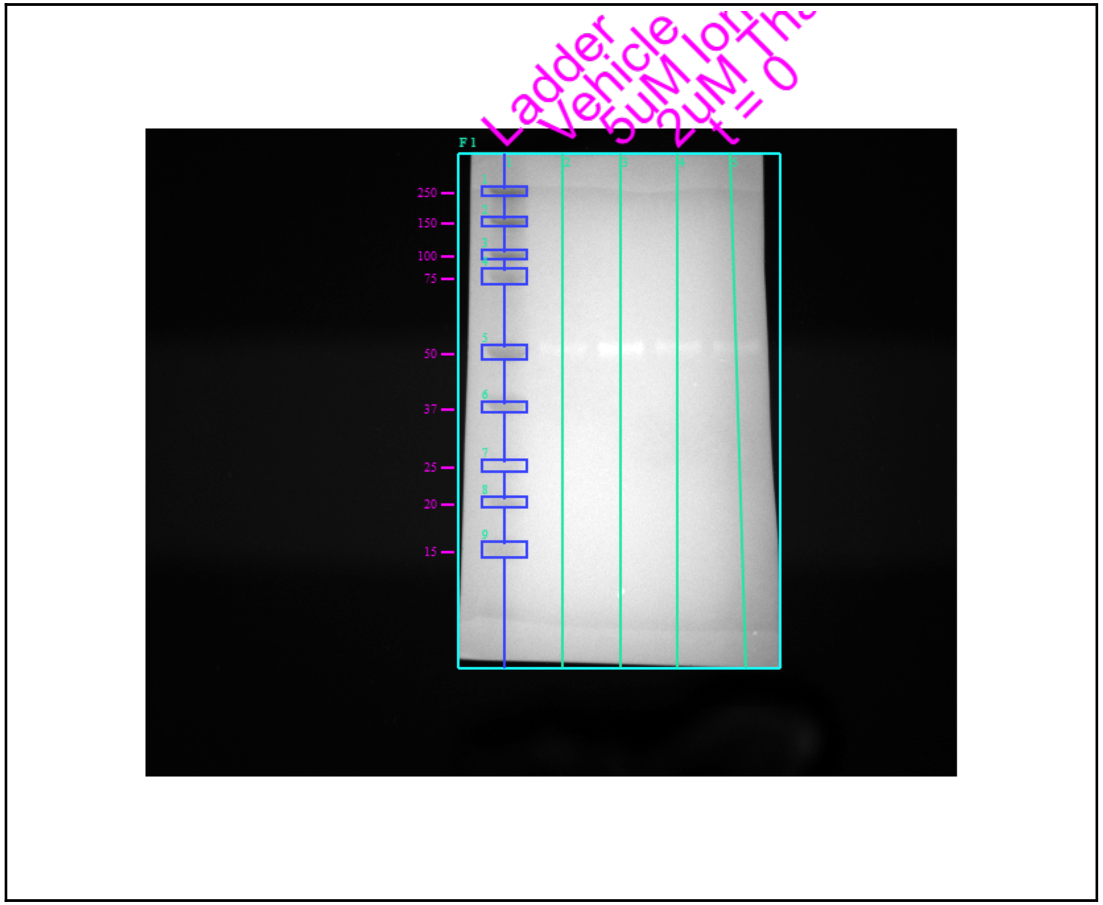

CHEMI_03242022_150645

Date:	24 March 2022 03:06:45PM
Mode:	Chemi Blots
Notes:	
Model:	FL1500
Instrument name:	2462619090234
Serial No:	2462619090234
Firmware version:	1.6.0
iBA version:	5.0
Image size:	615px X 491px
Image area:	112.7mm X 90.16mm
Optical Zoom:	2x
Digital Zoom:	1.1x
Focus level:	455
Resolution:	5 x 5
Exposure time:	1142 ms
Exposure mode:	Normal

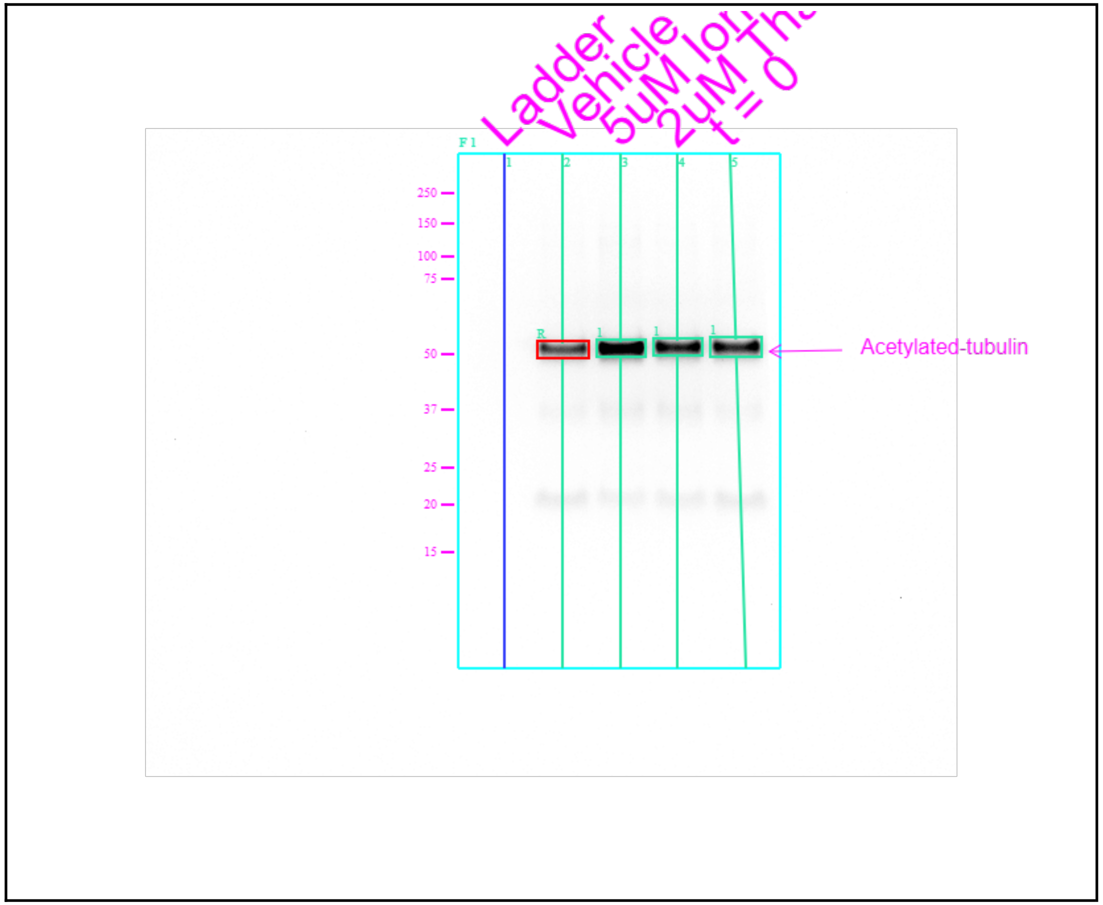

LANE AND BAND ANALYSIS DATA TABLE

CHEMI_03242022_150645

Frame: 1
Channel: Membrane
Sensitivity: 100
Molecular Weight Analysis Regression Method : Point to Point

Lane 1 - Ladder

#	Vol. (Int.)	Local Bg. Corr. Vol.	Area	Rf	Density	Local Bg. Corr. Den.	% band purity	% lane purity	Mol. Wt.
1	10,929,779	1,535,475	280	0.072	39,034	5,483.841	13.572	2.763	250
2	10,234,798	1,375,347	280	0.131	36,552	4,911.955	12.156	2.587	150
3	9,745,166	1,407,850	280	0.195	34,804	5,028.036	12.444	2.463	100
4	14,330,674	1,398,971	455	0.238	31,495	3,074.663	12.365	3.622	75
5	12,964,029	1,966,714	420	0.385	30,866	4,682.654	17.383	3.277	50
6	9,039,608	1,049,833	315	0.492	28,697	3,332.804	9.279	2.285	37
7	8,819,678	499,597	350	0.605	25,199	1,427.421	4.416	2.229	25
8	8,446,408	916,812	315	0.677	26,813	2,910.514	8.103	2.135	20
9	12,184,883	1,163,240	455	0.769	26,779	2,556.573	10.282	3.08	15

Frame: 1
Channel: Chemi
Sensitivity: 100
Molecular Weight Analysis Regression Method : Point to Point

Lane 2 - Vehicle

#	Vol. (Int.)	Local Bg. Corr. Vol.	Area	Rf	Density	Local Bg. Corr. Den.	% band purity	% lane purity	Mol. Wt.	Rel. Quant. (w/ LB Corr. Vol.)
1	7,189,477	5,692,914	560	0.379	12,838	10,165	100	52.797	50.877	1

Lane 3 - 5uM Ionomycin

#	Vol. (Int.)	Local Bg. Corr. Vol.	Area	Rf	Density	Local Bg. Corr. Den.	% band purity	% lane purity	Mol. Wt.	Rel. Quant. (w/ LB Corr. Vol.)
1	13,460,185	11,267,909	532	0.377	25,301	21,180	100	61.304	51.316	1.979

Lane 4 - 2uM Thapsigargin

#	Vol. (Int.)	Local Bg. Corr. Vol.	Area	Rf	Density	Local Bg. Corr. Den.	% band purity	% lane purity	Mol. Wt.	Rel. Quant. (w/ LB Corr. Vol.)

#	Vol. (Int.)	Local Bg. Corr. Vol.	Area	Rf	Density	Local Bg. Corr. Den.	% band purity	% lane purity	Mol. Wt.	Rel. Quant. (w/ LB Corr. Vol.)
1	9,355,784	7,523,296	532	0.374	17,586	14,141	100	55.26	51.754	1.322

Lane 5 - t = 0

#	Vol. (Int.)	Local Bg. Corr. Vol.	Area	Rf	Density	Local Bg. Corr. Den.	% band purity	% lane purity	Mol. Wt.	Rel. Quant. (w/ LB Corr. Vol.)
1	9,650,451	8,184,430	640	0.374	15,078	12,788	100	62.812	51.754	1.438

iBright™ Image Analysis Report

Katarina+ Chang

18 November 2022

CHEMI_03252022_113001

Date:	25 March 2022 11:30:01AM
Mode:	Chemi Blots
Notes:	
Model:	FL1500
Instrument name:	2462619090234
Serial No:	2462619090234
Firmware version:	1.6.0
iBA version:	5.0
Image size:	520px X 415px
Image area:	112.7mm X 90.16mm
Optical Zoom:	2x
Digital Zoom:	1.3x
Focus level:	455
Resolution:	5 x 5
Exposure time:	1411 ms
Exposure mode:	Normal

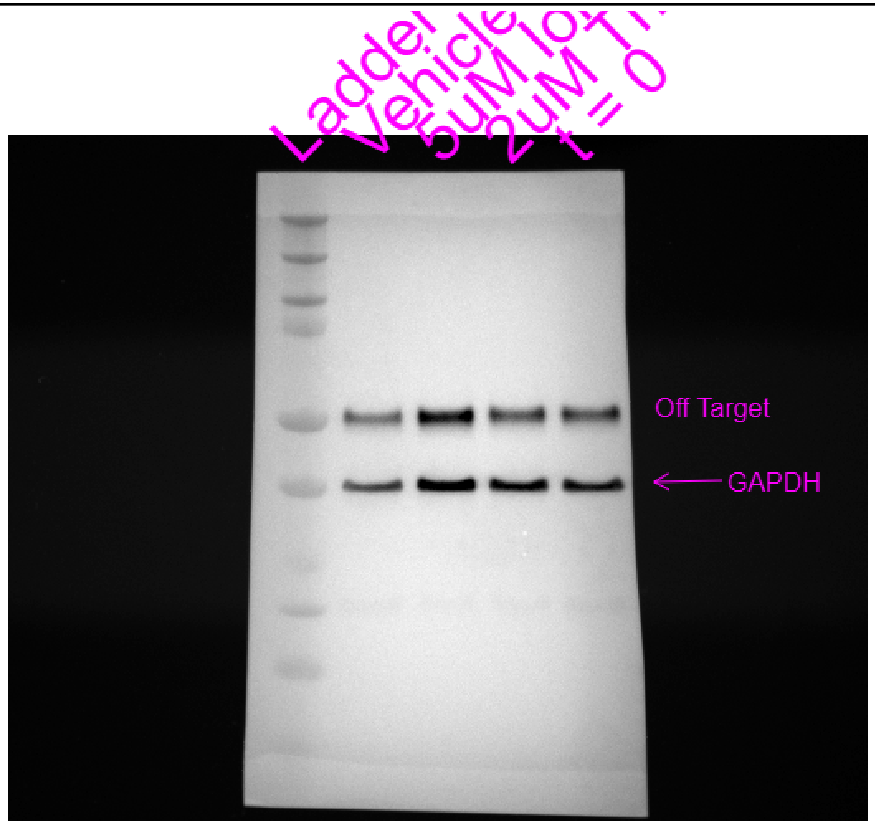

CHEMI_03252022_113001

Date:	25 March 2022 11:30:01AM
Mode:	Chemi Blots
Notes:	
Model:	FL1500
Instrument name:	2462619090234
Serial No:	2462619090234
Firmware version:	1.6.0
iBA version:	5.0
Image size:	520px X 415px
Image area:	112.7mm X 90.16mm
Optical Zoom:	2x
Digital Zoom:	1.3x
Focus level:	455
Resolution:	5 x 5
Exposure time:	1411 ms
Exposure mode:	Normal

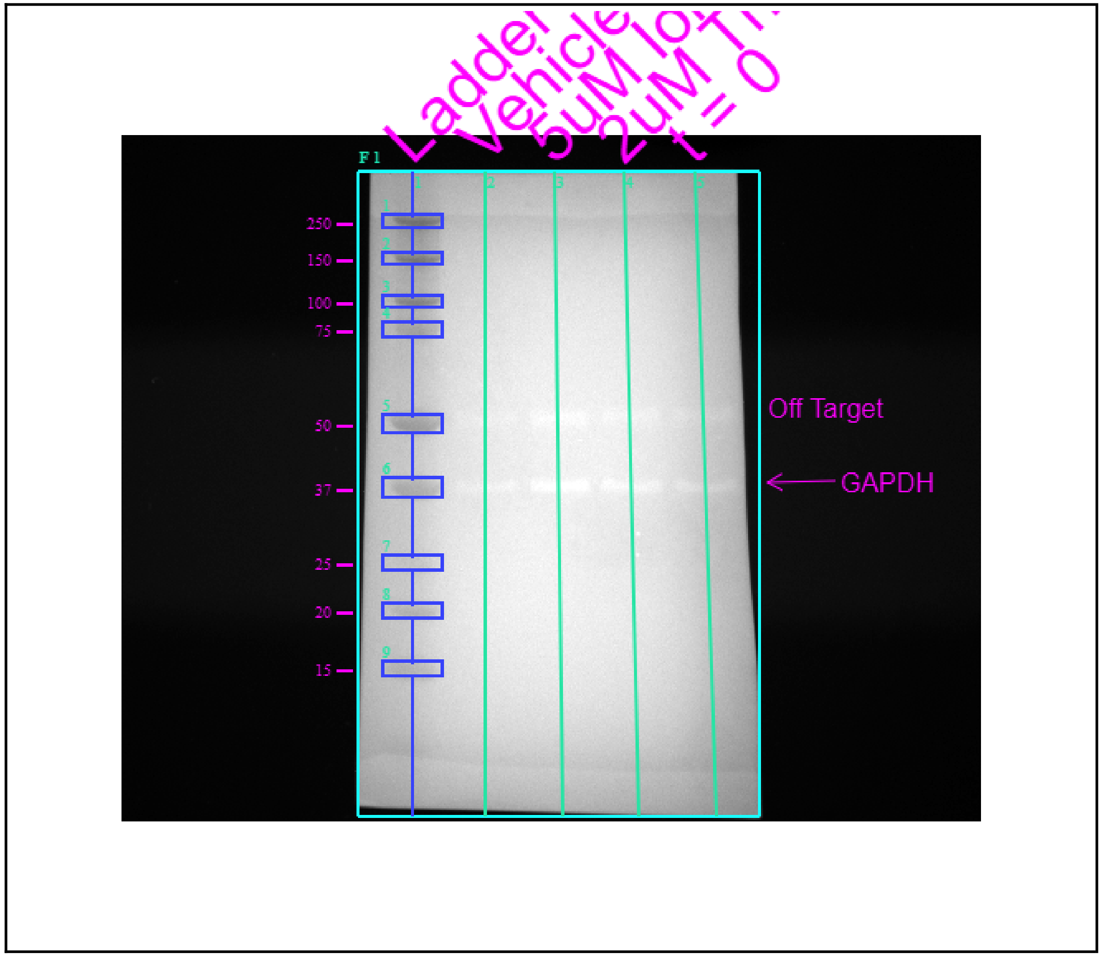

CHEMI_03252022_113001

Date:	25 March 2022 11:30:01AM
Mode:	Chemi Blots
Notes:	
Model:	FL1500
Instrument name:	2462619090234
Serial No:	2462619090234
Firmware version:	1.6.0
iBA version:	5.0
Image size:	520px X 415px
Image area:	112.7mm X 90.16mm
Optical Zoom:	2x
Digital Zoom:	1.3x
Focus level:	455
Resolution:	5 x 5
Exposure time:	1411 ms
Exposure mode:	Normal

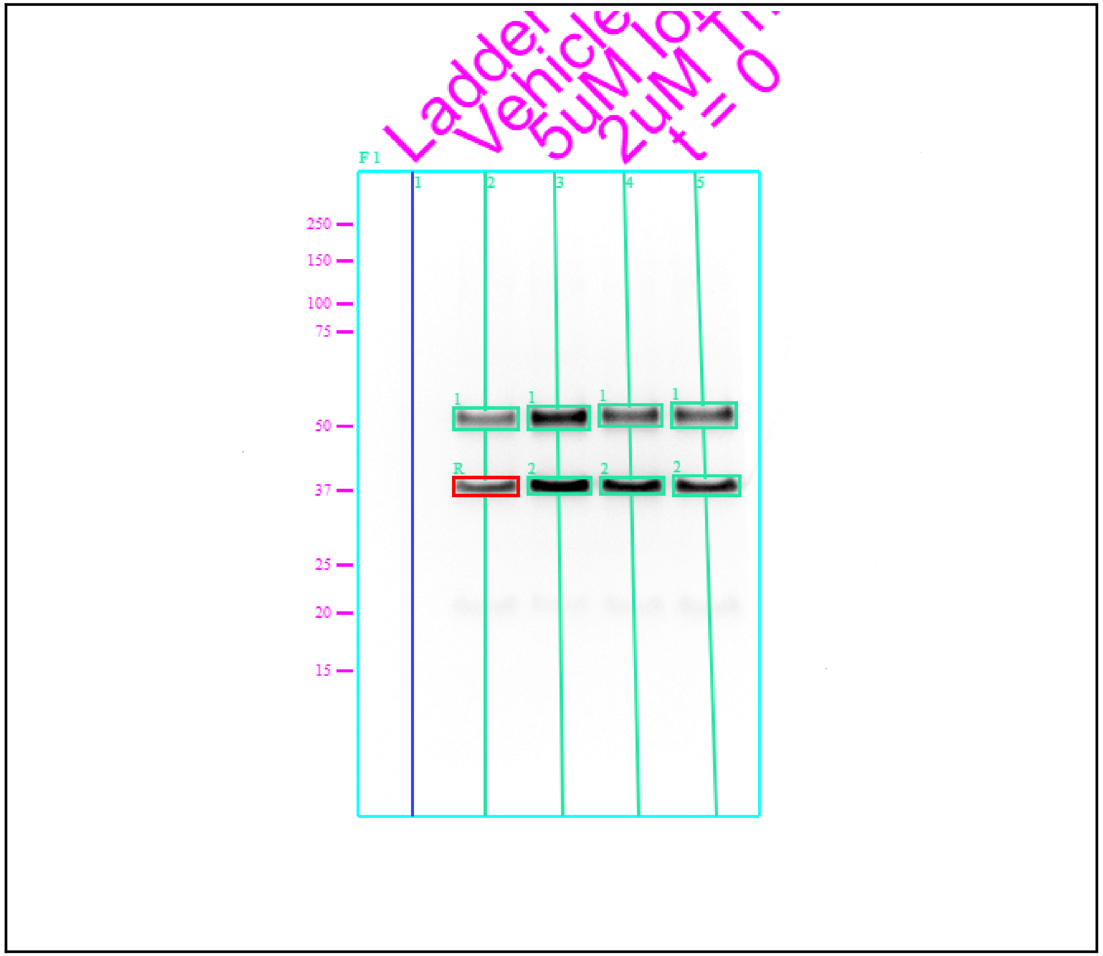

LANE AND BAND ANALYSIS DATA TABLE

CHEMI_03252022_113001

Frame: 1
Channel: Membrane
Sensitivity: 100
Molecular Weight Analysis Regression Method : Point to Point

Lane 1 - Ladder

#	Vol. (Int.)	Local Bg. Corr. Vol.	Area	Rf	Density	Local Bg. Corr. Den.	% band purity	% lane purity	Rolling Bg. Corr. Vol.	Rolling Bg. Corr. Den.	Mol. Wt.
1	12,533,239	1,776,564	333	0.077	37,637	5,335.027	15.969	3.39	1,903,104	5,715.027	250
2	10,531,945	1,432,086	296	0.133	35,580	4,838.131	10.567	2.243	1,259,264	4,254.27	150
3	9,994,453	1,450,755	296	0.2	33,765	4,901.199	11.224	2.383	1,337,600	4,518.919	100
4	11,356,453	1,094,407	370	0.244	30,693	2,957.858	8.053	1.71	959,744	2,593.903	75
5	13,608,766	1,983,925	444	0.39	30,650	4,468.3	18.023	3.826	2,147,840	4,837.477	50
6	13,829,460	1,704,603	481	0.49	28,751	3,543.875	14.869	3.156	1,772,032	3,684.058	37
7	9,636,898	516,232	370	0.605	26,045	1,395.224	5.02	1.066	598,272	1,616.951	25
8	10,257,452	1,011,411	370	0.679	27,722	2,733.544	8.247	1.751	982,784	2,656.173	20
9	10,509,220	878,499	370	0.769	28,403	2,374.322	8.028	1.704	956,672	2,585.6	15

Frame: 1
Channel: Chemi
Sensitivity: 100
Molecular Weight Analysis Regression Method : Point to Point

Lane 2 - Vehicle

#	Vol. (Int.)	Local Bg. Corr. Vol.	Area	Rf	Density	Local Bg. Corr. Den.	[redacted]
1	6,261,348	4,840,435	560	0.382	11,180	8,643.634	[redacted]
2	6,374,822	5,203,347	480	0.487	13,280	10,840	[redacted]

#	% band purity	% lane purity	Rolling Bg. Corr. Vol.	Rolling Bg. Corr. Den.	Mol. Wt.	Rel. Quant. (w/ LB Corr. Vol.)
1	48.811	42.16	5,260,544	9,393.829	51.316	0.93
2	51.189	44.213	5,516,800	11,493	37.333	1

Lane 3 - 5uM Ionomycin

#	Vol. (Int.)	Local Bg. Corr. Vol.	Area	Rf	Density	Local Bg. Corr. Den.	[redacted]

#	Vol. (Int.)	Local Bg. Corr. Vol.	Area	Rf	Density	Local Bg. Corr. Den.
1	11,242,132	9,336,247	570	0.382	19,723	16,379
2	10,941,207	9,238,061	429	0.487	25,503	21,533

#	% band purity	% lane purity	Rolling Bg. Corr. Vol.	Rolling Bg. Corr. Den.	Mol. Wt.	Rel. Quant. (w/ LB Corr. Vol.)
1	50.531	45.511	10,076,416	17,677	51.316	1.794
2	49.469	44.555	9,864,704	22,994	37.333	1.775

Lane 4 - 2uM Thapsigargin

#	Vol. (Int.)	Local Bg. Corr. Vol.	Area	Rf	Density	Local Bg. Corr. Den.
1	8,116,494	6,340,664	546	0.377	14,865	11,612
2	9,198,399	7,470,742	429	0.487	21,441	17,414

#	% band purity	% lane purity	Rolling Bg. Corr. Vol.	Rolling Bg. Corr. Den.	Mol. Wt.	Rel. Quant. (w/ LB Corr. Vol.)
1	46.154	39.628	7,089,152	12,983	52.193	1.219
2	53.846	46.232	8,270,592	19,278	37.333	1.436

Lane 5 - t = 0

#	Vol. (Int.)	Local Bg. Corr. Vol.	Area	Rf	Density	Local Bg. Corr. Den.
1	8,559,832	7,142,745	640	0.377	13,374	11,160
2	8,886,728	7,629,139	533	0.487	16,673	14,313

#	% band purity	% lane purity	Rolling Bg. Corr. Vol.	Rolling Bg. Corr. Den.	Mol. Wt.	Rel. Quant. (w/ LB Corr. Vol.)
1	48.778	44.755	7,666,944	11,979	52.193	1.373
2	51.222	46.997	8,050,944	15,104	37.333	1.466

iBright™ Image Analysis Report

Katarina+ Chang

18 November 2022

CHEMI_03172022_141742

Date:	17 March 2022 02:17:42PM
Mode:	Chemi Blots
Notes:	
Model:	FL1500
Instrument name:	2462619090234
Serial No:	2462619090234
Firmware version:	1.6.0
iBA version:	5.0
Image size:	615px X 491px
Image area:	112.7mm X 90.16mm
Optical Zoom:	2x
Digital Zoom:	1.1x
Focus level:	455
Resolution:	5 x 5
Exposure time:	60000 ms
Exposure mode:	Normal

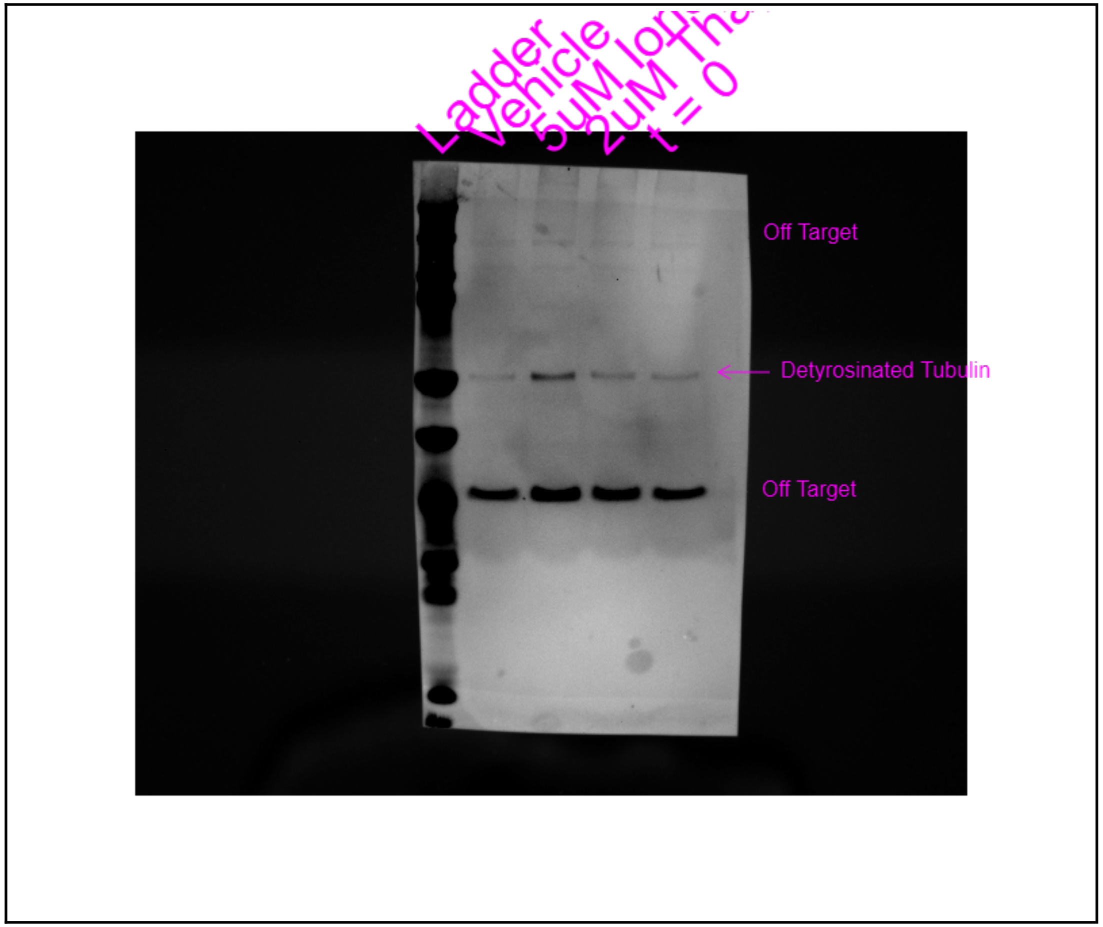

CHEMI_03172022_141742

Date:	17 March 2022 02:17:42PM
Mode:	Chemi Blots
Notes:	
Model:	FL1500
Instrument name:	2462619090234
Serial No:	2462619090234
Firmware version:	1.6.0
iBA version:	5.0
Image size:	615px X 491px
Image area:	112.7mm X 90.16mm
Optical Zoom:	2x
Digital Zoom:	1.1x
Focus level:	455
Resolution:	5 x 5
Exposure time:	60000 ms
Exposure mode:	Normal

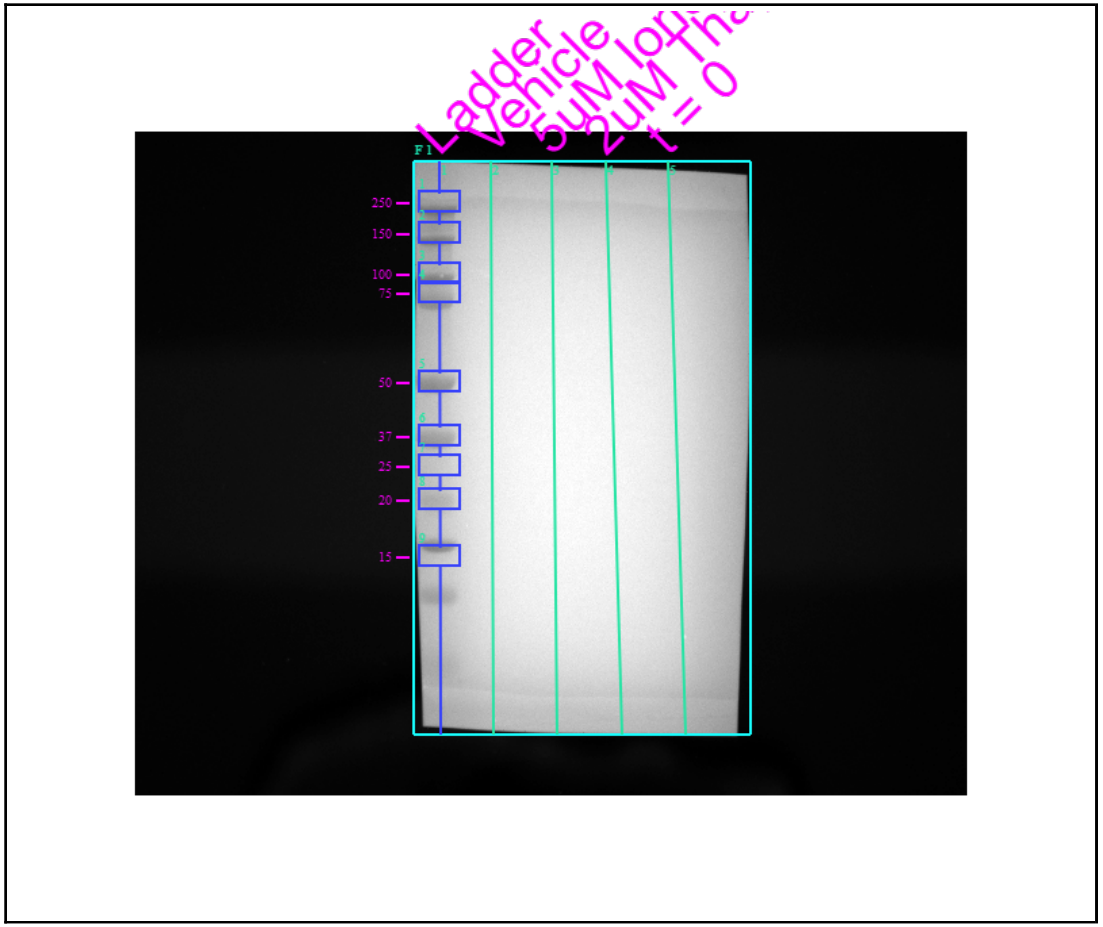

CHEMI_03172022_141742

Date:	17 March 2022 02:17:42PM
Mode:	Chemi Blots
Notes:	
Model:	FL1500
Instrument name:	2462619090234
Serial No:	2462619090234
Firmware version:	1.6.0
iBA version:	5.0
Image size:	615px X 491px
Image area:	112.7mm X 90.16mm
Optical Zoom:	2x
Digital Zoom:	1.1x
Focus level:	455
Resolution:	5 x 5
Exposure time:	60000 ms
Exposure mode:	Normal

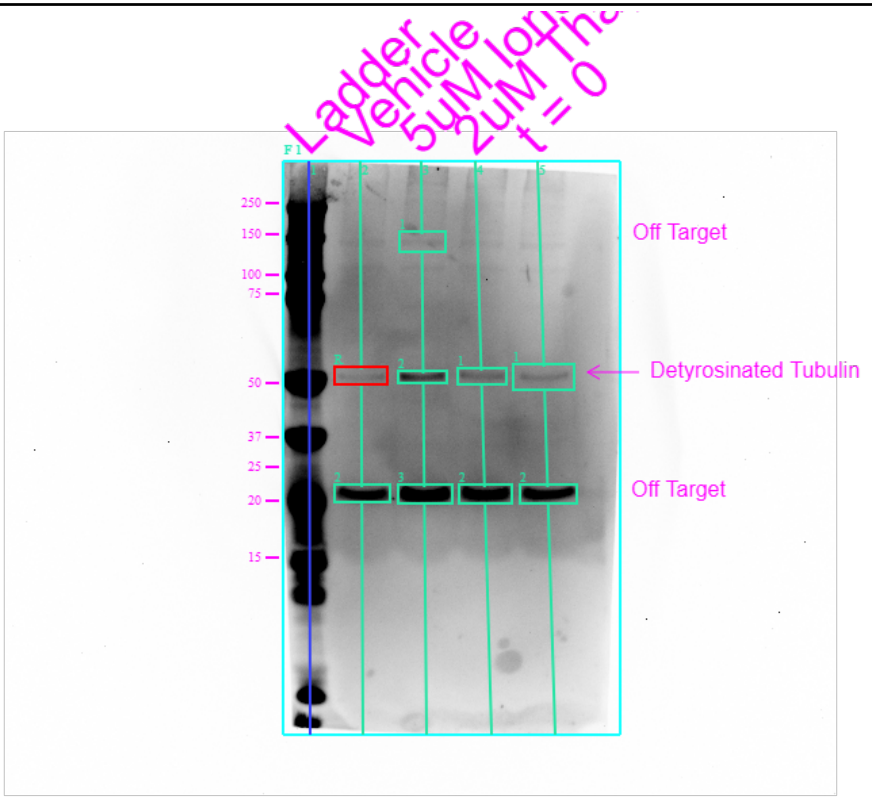

LANE AND BAND ANALYSIS DATA TABLE

CHEMI_03172022_141742

Frame: 1
Channel: Membrane
Sensitivity: 100
Molecular Weight Analysis Regression Method : Point to Point

Lane 1 - Ladder

#	Vol. (Int.)	Local Bg. Corr. Vol.	Area	Rf	Density	Local Bg. Corr. Den.	% band purity	% lane purity	Mol. Wt.
1	17,928,454	787,803	496	0.068	36,146	1,588.313	11.047	3.879	250
2	17,821,594	814,375	496	0.123	35,930	1,641.887	11.42	3.855	150
3	16,808,100	889,470	496	0.193	33,887	1,793.287	12.473	3.636	100
4	16,467,244	142,273	496	0.226	33,200	286.841	1.995	3.562	75
5	16,325,746	2,048,410	496	0.382	32,914	4,129.859	28.725	3.532	50
6	15,602,101	1,546,273	496	0.476	31,455	3,117.486	21.683	3.375	37
7	13,518,776	9,184.223	496	0.528	27,255	18.517	0.129	2.925	25
8	14,721,317	428,060	496	0.587	29,680	863.026	6.003	3.185	20
9	15,163,389	465,301	496	0.686	30,571	938.108	6.525	3.28	15

Frame: 1
Channel: Chemi
Sensitivity: 100
Molecular Weight Analysis Regression Method : Point to Point

Lane 2 - Vehicle

#	Vol. (Int.)	Local Bg. Corr. Vol.	Area	Rf	Density	Local Bg. Corr. Den.	% band purity	% lane purity	Mol. Wt.	Rel. Quant. (w/ LB Corr. Vol.)
1	18,201,576	931,514	560	0.373	32,502	1,663.418	8.43	4.68	51.515	1
2	30,205,673	10,117,936	588	0.578	51,370	17,207	91.57	7.766	20.8	10.862

Lane 3 - 5uM Ionomycin

#	Vol. (Int.)	Local Bg. Corr. Vol.	Area	Rf	Density	Local Bg. Corr. Den.	% band purity	% lane purity	Mol. Wt.	Rel. Quant. (w/ LB Corr. Vol.)
1	16,521,935	553,750	560	0.139	29,503	988.84	3.492	4.227	138.333	0.594
2	16,121,234	3,252,053	370	0.375	43,570	8,789.334	20.511	4.124	51.136	3.491
3	34,417,531	12,049,662	615	0.58	55,963	19,592	75.997	8.805	20.6	12.936

Lane 4 - 2uM Thapsigargin

#	Vol. (Int.)	Local Bg. Corr. Vol.	Area	Rf	Density	Local Bg. Corr. Den.	% band purity	% lane purity	Mol. Wt.	Rel. Quant. (w/ LB Corr. Vol.)
1	17,499,750	1,503,045	481	0.375	36,382	3,124.834	12.521	4.676	51.136	1.614
2	32,549,298	10,500,872	600	0.58	54,248	17,501	87.479	8.698	20.6	11.273

Lane 5 - t = 0

#	Vol. (Int.)	Local Bg. Corr. Vol.	Area	Rf	Density	Local Bg. Corr. Den.	% band purity	% lane purity	Mol. Wt.	Rel. Quant. (w/ LB Corr. Vol.)
1	29,136,990	2,074,760	920	0.375	31,670	2,255.175	16.052	9.238	51.136	2.227
2	32,108,327	10,850,142	645	0.58	49,780	16,821	83.948	10.18	20.6	11.648

iBright™ Image Analysis Report

Katarina+ Chang

18 November 2022

CHEMI_03202022_134003

Date:	20 March 2022 01:40:03PM
Mode:	Chemi Blots
Notes:	
Model:	FL1500
Instrument name:	2462619090234
Serial No:	2462619090234
Firmware version:	1.6.0
iBA version:	5.0
Image size:	676px X 540px
Image area:	118.63mm X 94.91mm
Optical Zoom:	1.9x
Digital Zoom:	1x
Focus level:	430
Resolution:	5 x 5
Exposure time:	950 ms
Exposure mode:	Normal

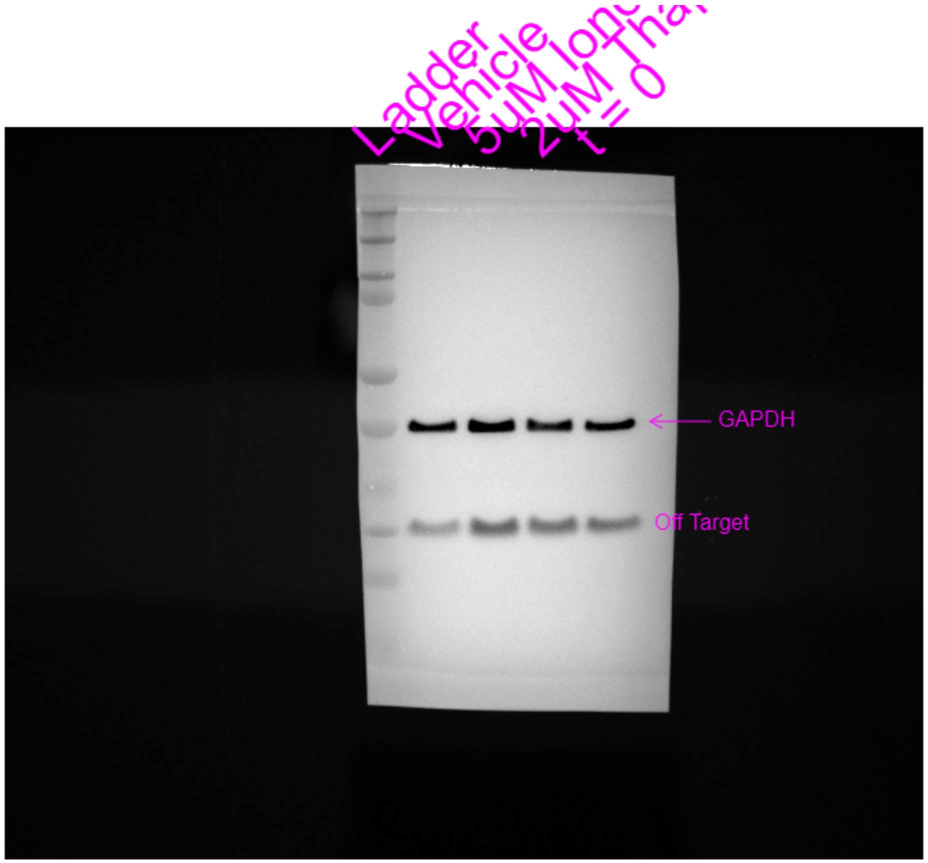

CHEMI_03202022_134003

Date:	20 March 2022 01:40:03PM
Mode:	Chemi Blots
Notes:	
Model:	FL1500
Instrument name:	2462619090234
Serial No:	2462619090234
Firmware version:	1.6.0
iBA version:	5.0
Image size:	676px X 540px
Image area:	118.63mm X 94.91mm
Optical Zoom:	1.9x
Digital Zoom:	1x
Focus level:	430
Resolution:	5 x 5
Exposure time:	950 ms
Exposure mode:	Normal

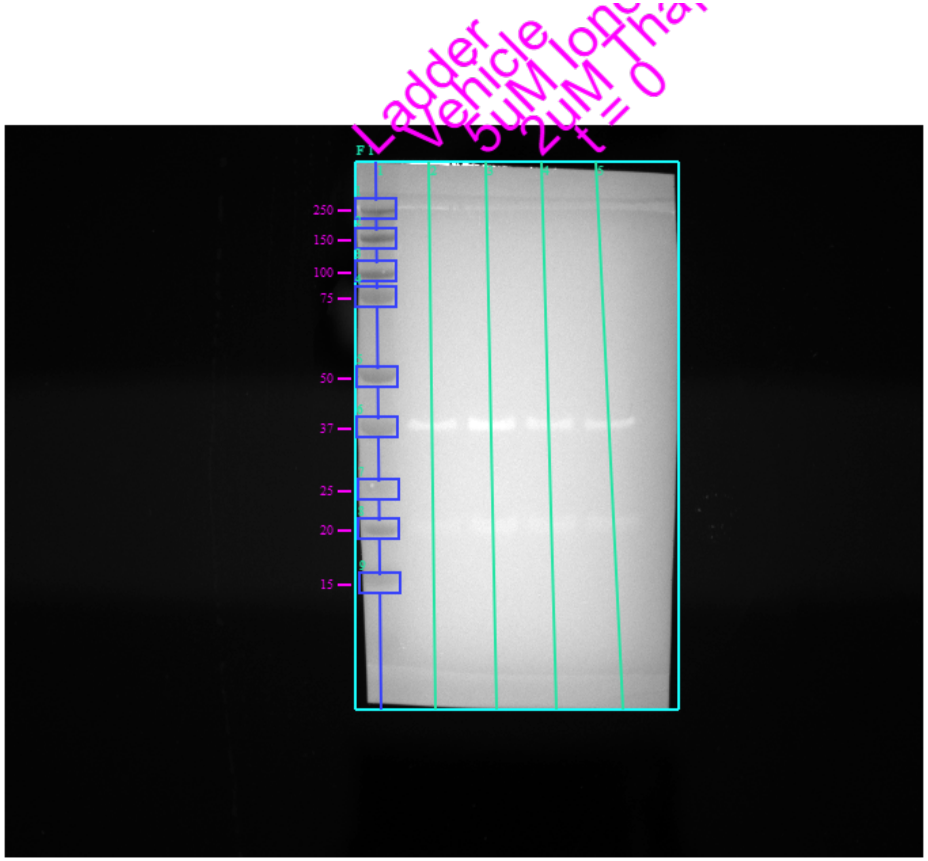

CHEMI_03202022_134003

Date:	20 March 2022 01:40:03PM
Mode:	Chemi Blots
Notes:	
Model:	FL1500
Instrument name:	2462619090234
Serial No:	2462619090234
Firmware version:	1.6.0
iBA version:	5.0
Image size:	676px X 540px
Image area:	118.63mm X 94.91mm
Optical Zoom:	1.9x
Digital Zoom:	1x
Focus level:	430
Resolution:	5 x 5
Exposure time:	950 ms
Exposure mode:	Normal

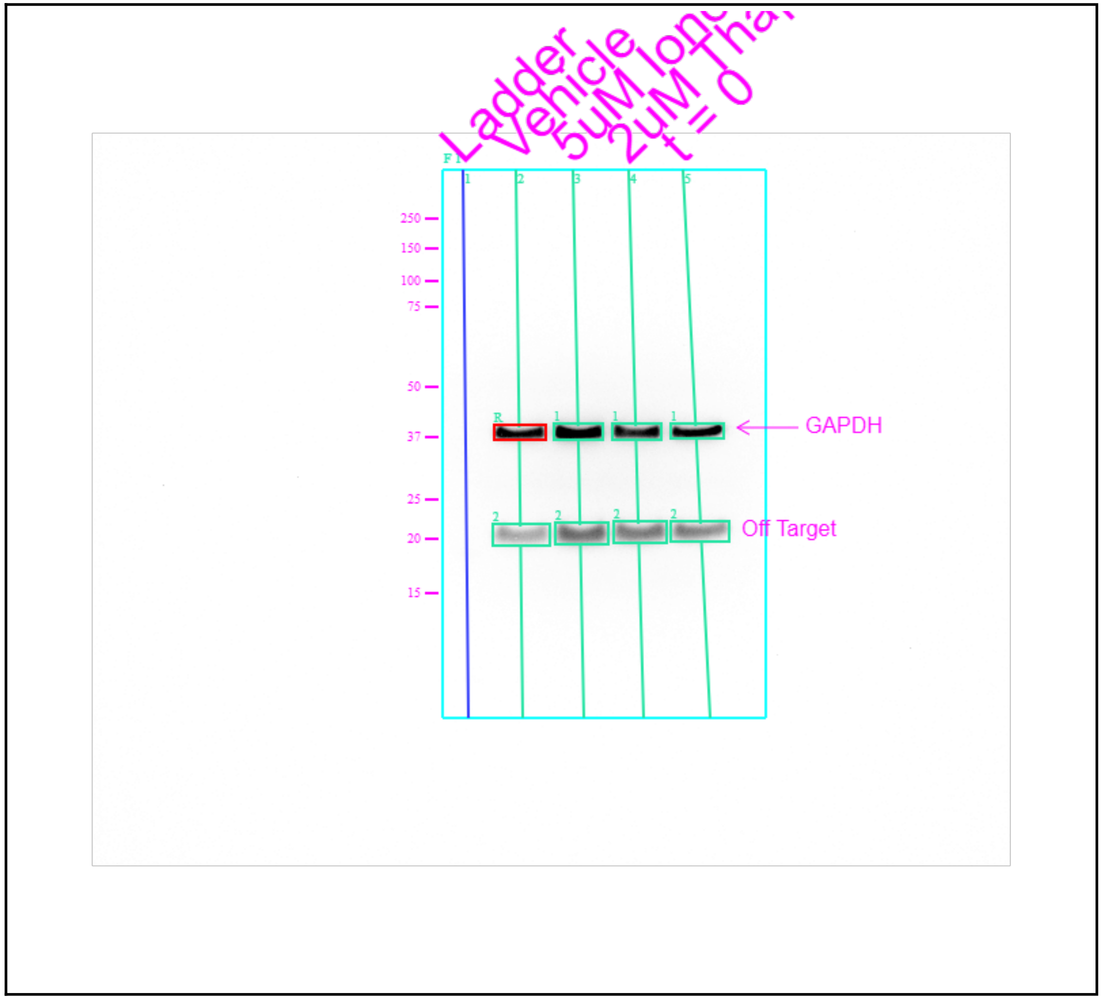

LANE AND BAND ANALYSIS DATA TABLE

CHEMI_03202022_134003

Frame: 1
Channel: Membrane
Sensitivity: 100
Molecular Weight Analysis Regression Method : Point to Point

Lane 1 - Ladder

#	Vol. (Int.)	Local Bg. Corr. Vol.	Area	Rf	Density	Local Bg. Corr. Den.	% band purity	% lane purity	Mol. Wt.
1	18,281,667	227,318	496	0.084	36,858	458.304	5.053	3.601	250
2	18,368,783	516,189	496	0.139	37,033	1,040.705	11.474	3.618	150
3	18,098,581	775,610	496	0.198	36,489	1,563.731	17.241	3.565	100
4	17,810,913	784,567	496	0.245	35,909	1,581.79	17.44	3.508	75
5	16,173,049	134,901	496	0.391	32,606	271.979	2.999	3.186	50
6	15,689,504	213,358	496	0.483	31,632	430.157	4.743	3.091	37
7	14,599,141	161,063	496	0.597	29,433	324.724	3.58	2.876	25
8	16,000,837	679,144	496	0.668	32,259	1,369.242	15.097	3.152	20
9	16,223,690	1,006,447	496	0.767	32,709	2,029.129	22.372	3.196	15

Frame: 1
Channel: Chemi
Sensitivity: 100
Molecular Weight Analysis Regression Method : Point to Point

Lane 2 - Vehicle

#	Vol. (Int.)	Local Bg. Corr. Vol.	Area	Rf	Density	Local Bg. Corr. Den.	% band purity	% lane purity	Mol. Wt.	Rel. Quant. (w/ LB Corr. Vol.)
1	9,655,023	8,465,892	468	0.478	20,630	18,089	68.911	49.764	37.703	1
2	4,979,969	3,819,432	731	0.666	6,812.543	5,224.942	31.089	25.668	20.172	0.451

Lane 3 - 5uM Ionomycin

#	Vol. (Int.)	Local Bg. Corr. Vol.	Area	Rf	Density	Local Bg. Corr. Den.	% band purity	% lane purity	Mol. Wt.	Rel. Quant. (w/ LB Corr. Vol.)
1	13,224,720	11,348,451	481	0.478	27,494	23,593	63.21	44.723	37.703	1.34
2	8,452,247	6,605,193	680	0.663	12,429	9,713.519	36.79	28.584	20.345	0.78

Lane 4 - 2uM Thapsigargin

#	Vol. (Int.)	Local Bg. Corr. Vol.	Area	Rf	Density	Local Bg. Corr. Den.	% band purity	% lane purity	Mol. Wt.	Rel. Quant. (w/ LB Corr. Vol.)
1	9,420,729	7,847,870	481	0.478	19,585	16,315	57.56	38.752	37.703	0.927
2	7,575,947	5,786,446	680	0.661	11,141	8,509.48	42.44	31.164	20.517	0.684

Lane 5 - t = 0

#	Vol. (Int.)	Local Bg. Corr. Vol.	Area	Rf	Density	Local Bg. Corr. Den.	% band purity	% lane purity	Mol. Wt.	Rel. Quant. (w/ LB Corr. Vol.)
1	10,431,543	9,182,270	480	0.475	21,732	19,129	62.965	47.728	38.054	1.085
2	6,654,743	5,400,964	704	0.658	9,452.76	7,671.825	37.035	30.448	20.69	0.638
