# Supplementary material for: Elevation of Cytoplasmic Calcium Suppresses Microtentacle Formation and Function in Breast Tumor Cells
Source: Cancers (Basel). 2023 Jan 31;15(3):884. doi: 10.3390/cancers15030884 (PMC9913253; doi:10.3390/cancers15030884)
Supplement: Supplementary file 1 [file cancers-15-00884-s001.zip › cancers-2080686-Supplementary/File S1_ Original Blots/Original Immunoblot Images MDAMB436 Biological Replicate 1.pdf]

iBright™ Image Analysis Report

Katarina+ Chang

19 November 2022

GAPDH CHEMI_01242022_133752

Date:	24 January 2022 01:37:52PM
Mode:	Chemi Blots
Notes:	
Model:	FL1500
Instrument name:	2462619090234
Serial No:	2462619090234
Firmware version:	1.6.0
iBA version:	5.0
Image size:	615px X 491px
Image area:	112.7mm X 90.16mm
Optical Zoom:	2x
Digital Zoom:	1.1x
Focus level:	455
Resolution:	5 x 5
Exposure time:	1072 ms
Exposure mode:	Normal

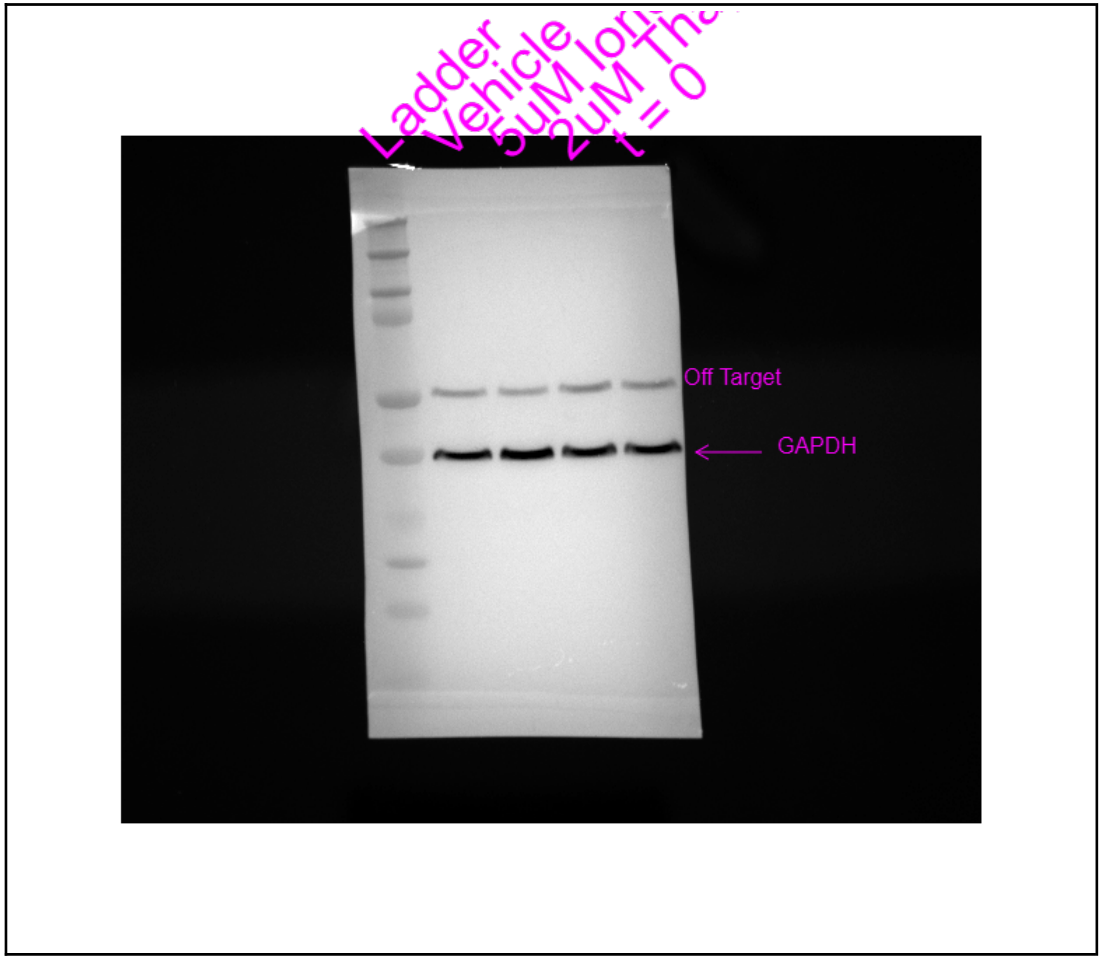

GAPDH CHEMI_01242022_133752

Date:	24 January 2022 01:37:52PM
Mode:	Chemi Blots
Notes:	
Model:	FL1500
Instrument name:	2462619090234
Serial No:	2462619090234
Firmware version:	1.6.0
iBA version:	5.0
Image size:	615px X 491px
Image area:	112.7mm X 90.16mm
Optical Zoom:	2x
Digital Zoom:	1.1x
Focus level:	455
Resolution:	5 x 5
Exposure time:	1072 ms
Exposure mode:	Normal

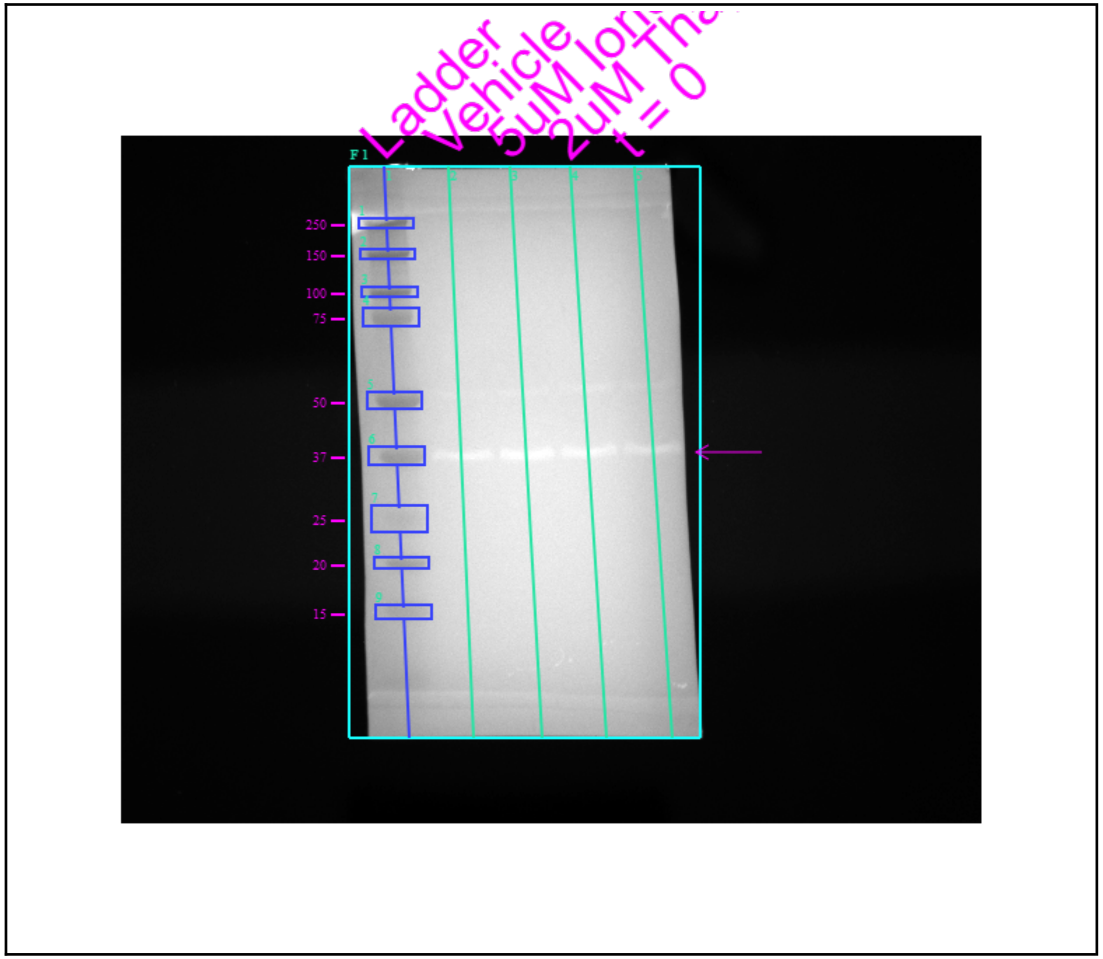

GAPDH CHEMI_01242022_133752

Date: 24 January 2022 01:37:52PM
Mode: Chemi Blots
Notes:
Model: FL1500
Instrument name: 2462619090234
Serial No: 2462619090234
Firmware version: 1.6.0
iBA version: 5.0
Image size: 615px X 491px
Image area: 112.7mm X 90.16mm
Optical Zoom: 2x
Digital Zoom: 1.1x
Focus level: 455
Resolution: 5 x 5
Exposure time: 1072 ms
Exposure mode: Normal

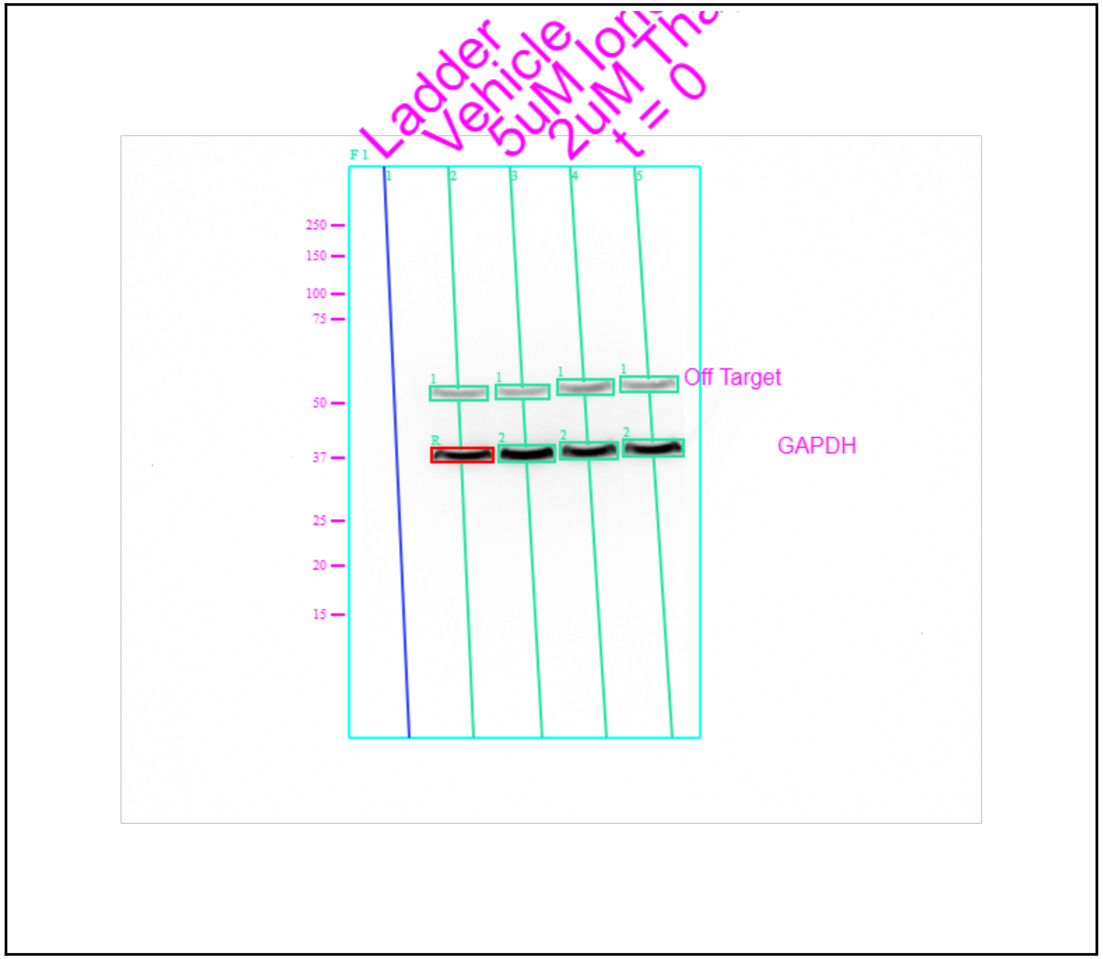

LANE AND BAND ANALYSIS DATA TABLE

GAPDH CHEMI_01242022_133752

Frame: 1
Channel: Membrane
Sensitivity: 100
Molecular Weight Analysis Regression Method : Point to Point

Lane 1 - Ladder

#	Vol. (Int.)	Local Bg. Corr. Vol.	Area	Rf	Density	Local Bg. Corr. Den.	% band purity	% lane purity	Rolling Bg. Corr. Vol.	Rolling Bg. Corr. Den.	Mol. Wt.
1	10,642,050	1,875,712	320	0.098	33,256	5,861.602	12.432	2.524	1,992,192	6,225.6	250
2	11,552,015	1,777,553	320	0.152	36,100	5,554.853	10.486	2.129	1,680,384	5,251.2	150
3	11,252,165	1,782,831	328	0.218	34,305	5,435.462	10.568	2.145	1,693,440	5,162.927	100
4	18,126,185	2,121,774	574	0.262	31,578	3,696.471	11.13	2.259	1,783,552	3,107.233	75
5	17,029,171	2,545,316	520	0.409	32,748	4,894.84	17.228	3.497	2,760,704	5,309.046	50
6	17,381,530	2,030,047	574	0.505	30,281	3,536.668	14.349	2.913	2,299,392	4,005.909	37
7	22,292,624	1,122,709	820	0.615	27,186	1,369.158	7.814	1.586	1,252,096	1,526.946	25
8	10,913,215	1,222,899	360	0.694	30,314	3,396.943	8.662	1.758	1,388,032	3,855.644	20
9	13,465,634	989,021	451	0.779	29,857	2,192.952	7.33	1.488	1,174,528	2,604.275	15

Frame: 1
Channel: Chemi
Sensitivity: 100
Molecular Weight Analysis Regression Method : Point to Point

Lane 2 - Vehicle

#	Vol. (Int.)	Local Bg. Corr. Vol.	Area	Rf	Density	Local Bg. Corr. Den.
1	3,461,341	2,682,751	462	0.397	7,492.08	5,806.821
2	11,295,821	9,263,447	495	0.505	22,819	18,714

#	% band purity	% lane purity	Rolling Bg. Corr. Vol.	Rolling Bg. Corr. Den.	Mol. Wt.	Rel. Quant. (w/ LB Corr. Vol.)
1	21.536	19.949	2,847,744	6,163.948	52.083	0.29
2	78.464	72.682	10,375,424	20,960	37	1

Lane 3 - 5uM Ionomycin

#	Vol. (Int.)	Local Bg. Corr. Vol.	Area	Rf	Density	Local Bg. Corr. Den.

#	Vol. (Int.)	Local Bg. Corr. Vol.	Area	Rf	Density	Local Bg. Corr. Den.	
1	3,053,396	2,203,429	429	0.395	7,117.473	5,136.199	
2	13,678,806	11,126,150	533	0.502	25,663	20,874	

#	% band purity	% lane purity	Rolling Bg. Corr. Vol.	Rolling Bg. Corr. Den.	Mol. Wt.	Rel. Quant. (w/ LB Corr. Vol.)
1	31.866	28.247	2,322,176	5,412.998	52.5	0.238
2	68.134	60.396	4,965,120	9,315.422	37.333	1.201

Lane 4 - 2uM Thapsgargin

#	Vol. (Int.)	Local Bg. Corr. Vol.	Area	Rf	Density	Local Bg. Corr. Den.	
1	4,384,157	3,397,786	492	0.385	8,910.888	6,906.07	
2	12,086,223	9,516,140	546	0.498	22,135	17,428	

#	% band purity	% lane purity	Rolling Bg. Corr. Vol.	Rolling Bg. Corr. Den.	Mol. Wt.	Rel. Quant. (w/ LB Corr. Vol.)
1	58.214	50.287	3,660,544	7,440.13	54.167	0.367
2	41.786	36.096	2,627,584	4,812.425	38	1.027

Lane 5 - t = 0

#	Vol. (Int.)	Local Bg. Corr. Vol.	Area	Rf	Density	Local Bg. Corr. Den.	
1	3,802,779	3,118,754	504	0.38	7,545.196	6,188.006	
2	12,513,572	10,741,828	572	0.493	21,876	18,779	

#	% band purity	% lane purity	Rolling Bg. Corr. Vol.	Rolling Bg. Corr. Den.	Mol. Wt.	Rel. Quant. (w/ LB Corr. Vol.)
1	37.726	34.28	3,156,480	6,262.857	55	0.337
2	62.274	56.585	5,210,368	9,109.035	38.667	1.16

iBright™ Image Analysis Report

Katarina+ Chang

19 November 2022

MLCK CHEMI_01202022_124024

Date:	20 January 2022 12:40:24PM
Mode:	Chemi Blots
Notes:	
Model:	FL1500
Instrument name:	2462619090234
Serial No:	2462619090234
Firmware version:	1.6.0
iBA version:	5.0
Image size:	563px X 450px
Image area:	112.7mm X 90.16mm
Optical Zoom:	2x
Digital Zoom:	1.2x
Focus level:	455
Resolution:	5 x 5
Exposure time:	54318 ms
Exposure mode:	Normal

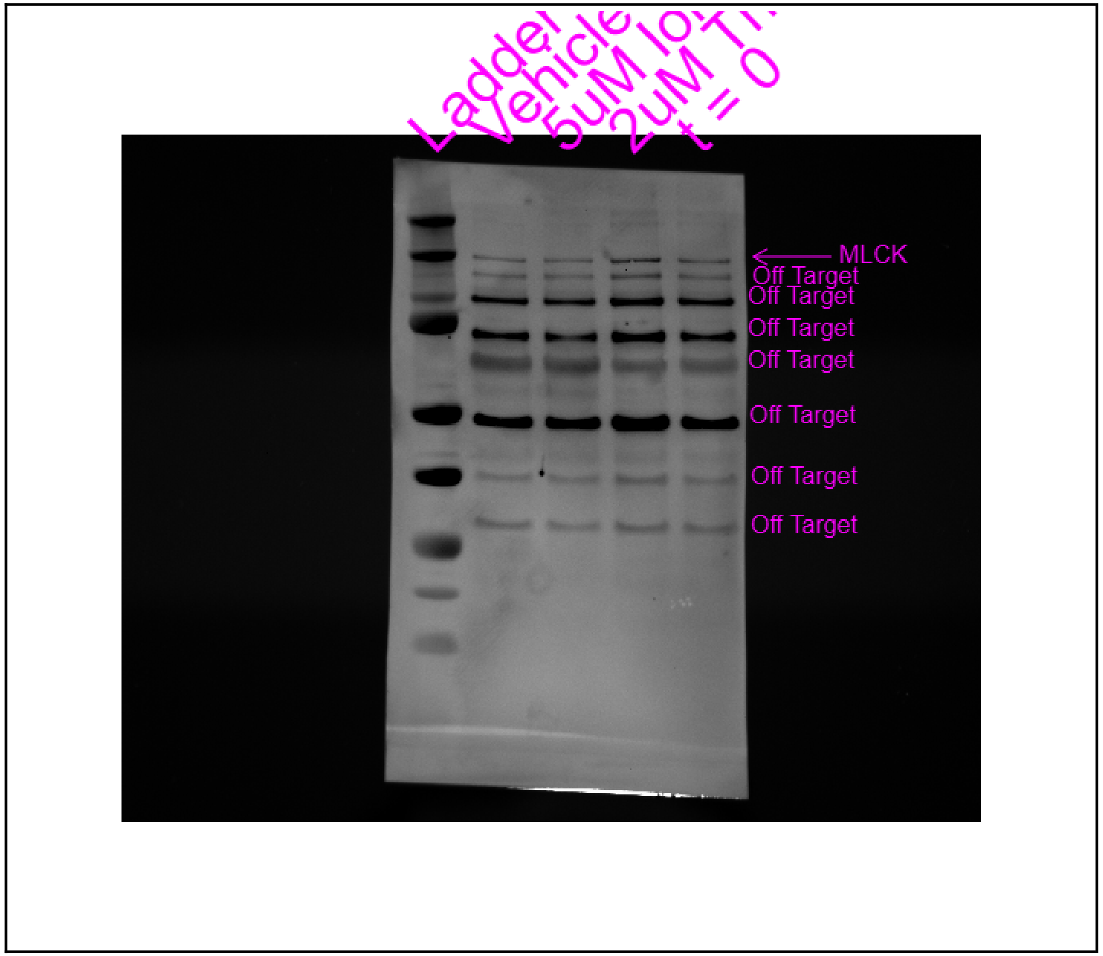

MLCK CHEMI_01202022_124024

Date:	20 January 2022 12:40:24PM
Mode:	Chemi Blots
Notes:	
Model:	FL1500
Instrument name:	2462619090234
Serial No:	2462619090234
Firmware version:	1.6.0
iBA version:	5.0
Image size:	563px X 450px
Image area:	112.7mm X 90.16mm
Optical Zoom:	2x
Digital Zoom:	1.2x
Focus level:	455
Resolution:	5 x 5
Exposure time:	54318 ms
Exposure mode:	Normal

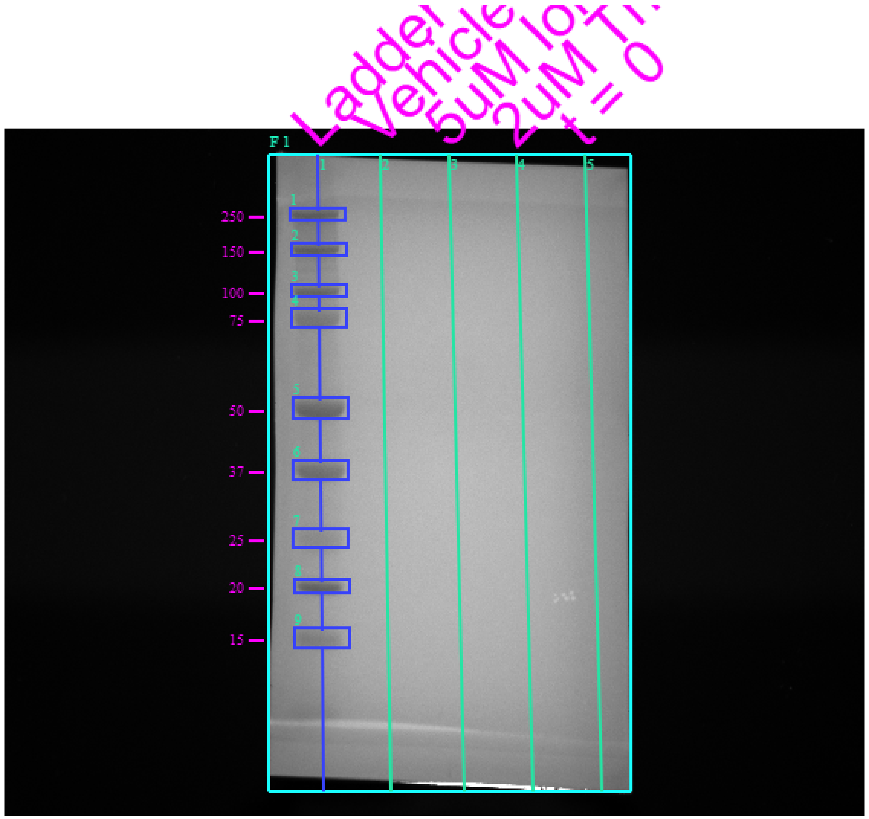

MLCK CHEMI_01202022_124024

Date:	20 January 2022 12:40:24PM
Mode:	Chemi Blots
Notes:	
Model:	FL1500
Instrument name:	2462619090234
Serial No:	2462619090234
Firmware version:	1.6.0
iBA version:	5.0
Image size:	563px X 450px
Image area:	112.7mm X 90.16mm
Optical Zoom:	2x
Digital Zoom:	1.2x
Focus level:	455
Resolution:	5 x 5
Exposure time:	54318 ms
Exposure mode:	Normal

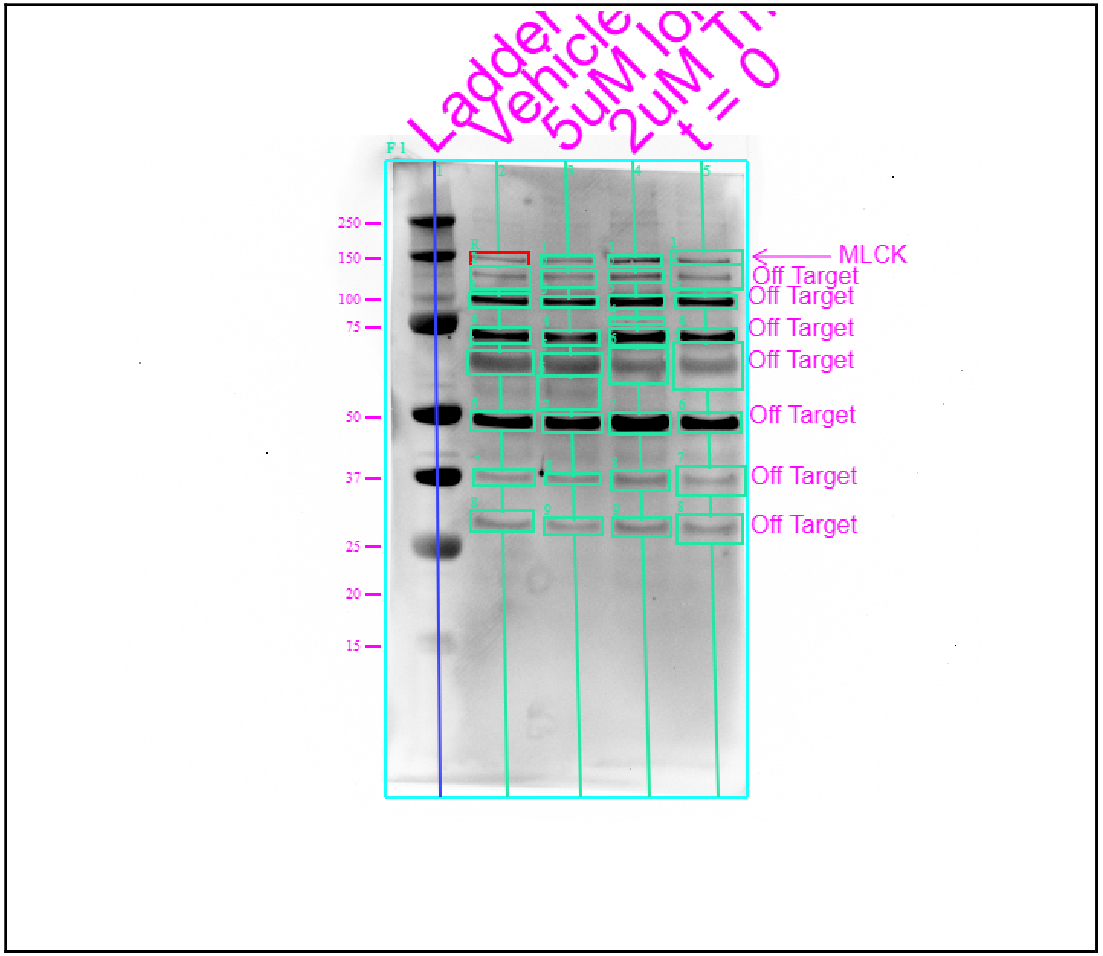

LANE AND BAND ANALYSIS DATA TABLE

MLCK CHEMI_01202022_124024

Frame: 1
Channel: Membrane
Sensitivity: 100
Molecular Weight Analysis Regression Method : Point to Point

Lane 1 - Ladder

#	Vol. (Int.)	Local Bg. Corr. Vol.	Area	Rf	Density	Local Bg. Corr. Den.	% band purity	% lane purity	Mol. Wt.
1	12,153,302	1,948,706	333	0.094	36,496	5,851.973	8.599	2.683	250
2	11,608,521	2,106,175	333	0.149	34,860	6,324.852	9.294	2.563	150
3	11,119,493	2,156,291	333	0.213	33,391	6,475.35	9.515	2.455	100
4	14,911,519	2,375,647	481	0.257	31,001	4,938.975	10.483	3.292	75
5	18,572,794	4,252,176	555	0.398	33,464	7,661.579	18.764	4.1	50
6	16,480,587	3,524,604	518	0.494	31,815	6,804.255	15.554	3.638	37
7	13,274,789	1,389,303	481	0.602	27,598	2,888.364	6.131	2.93	25
8	12,118,667	2,934,403	370	0.676	32,753	7,930.821	12.949	2.675	20
9	15,267,944	1,973,718	518	0.758	29,474	3,810.267	8.71	3.37	15

Frame: 1
Channel: Chemi
Sensitivity: 100
Molecular Weight Analysis Regression Method : Point to Point

Lane 2 - Vehicle

#	Vol. (Int.)	Local Bg. Corr. Vol.	Area	Rf	Density	Local Bg. Corr. Den.	% band purity	% lane purity	Mol. Wt.	Rel. Quant. (w/ LB Corr. Vol.)
1	9,545,910	1,119,443	390	0.153	24,476	2,870.368	2.621	2.884	146.296	1
2	17,050,474	931,969	640	0.182	26,641	1,456.202	2.182	5.151	124.074	0.833
3	17,914,404	7,272,791	410	0.218	43,693	17,738	17.03	5.412	97.222	6.497
4	22,267,022	9,135,129	480	0.273	46,389	19,031	21.391	6.727	72.034	8.16
5	27,722,301	7,190,550	748	0.317	37,061	9,613.036	16.838	8.375	64.407	6.423
6	27,885,509	12,810,806	602	0.408	46,321	21,280	29.998	8.424	48.7	11.444
7	11,868,384	1,428,611	492	0.496	24,122	2,903.682	3.345	3.585	36.733	1.276
8	14,810,582	2,815,900	630	0.566	23,508	4,469.683	6.594	4.474	29	2.515

Lane 3 - 5uM Ionomycin

#	Vol. (Int.)	Local Bg. Corr. Vol.	Area	Rf	Density	Local Bg. Corr. Den.	% band purity	% lane purity	Mol. Wt.	Rel. Quant. (w/ LB Corr. Vol.)
1	8,171,765	959,748	288	0.156	28,374	3,332.459	2.712	2.394	144.444	0.857
2	12,873,504	1,052,432	444	0.185	28,994	2,370.344	2.974	3.771	122.222	0.94
3	13,927,084	5,571,669	304	0.221	45,812	18,327	15.743	4.08	95.833	4.977
4	18,336,680	6,481,895	418	0.278	43,867	15,506	18.315	5.371	71.186	5.79
5	25,124,486	6,364,773	656	0.319	38,299	9,702.399	17.984	7.36	63.983	5.686
6	27,982,237	267,383	943	0.365	29,673	283.546	0.756	8.197	55.932	0.239
7	26,017,564	11,514,330	520	0.412	50,033	22,142	32.535	7.622	48.05	10.286
8	8,261,393	1,105,802	296	0.499	27,910	3,735.819	3.125	2.42	36.467	0.988
9	10,998,516	2,072,673	468	0.573	23,501	4,428.79	5.857	3.222	28.2	1.852

Lane 4 - 2uM Thapsigargin

#	Vol. (Int.)	Local Bg. Corr. Vol.	Area	Rf	Density	Local Bg. Corr. Den.	% band purity	% lane purity	Mol. Wt.	Rel. Quant. (w/ LB Corr. Vol.)
1	9,680,196	2,101,149	288	0.156	33,611	7,295.659	4.359	2.769	144.444	1.877
2	10,881,039	2,120,927	333	0.182	32,675	6,369.152	4.4	3.113	124.074	1.895
3	18,326,621	8,228,406	380	0.221	48,227	21,653	17.07	5.243	95.833	7.35
4	4,999,338	143,507	185	0.252	27,023	775.718	0.298	1.43	77.778	0.128
5	22,701,050	10,244,062	468	0.276	48,506	21,889	21.251	6.494	71.61	9.151
6	30,261,282	3,185,866	975	0.321	31,037	3,267.555	6.609	8.657	63.559	2.846
7	32,059,531	16,265,725	615	0.412	52,129	26,448	33.743	9.171	48.05	14.53
8	15,154,289	2,942,192	546	0.501	27,755	5,388.631	6.104	4.335	36.2	2.628
9	13,384,795	2,972,469	507	0.576	26,399	5,862.859	6.166	3.829	27.933	2.655

Lane 5 - t = 0

#	Vol. (Int.)	Local Bg. Corr. Vol.	Area	Rf	Density	Local Bg. Corr. Den.	% band purity	% lane purity	Mol. Wt.	Rel. Quant. (w/ LB Corr. Vol.)
1	11,510,360	2,969,208	480	0.151	23,979	6,185.851	5.563	3.97	148.148	2.652
2	19,230,561	2,209,884	816	0.182	23,566	2,708.191	4.14	6.633	124.074	1.974
3	17,338,234	8,526,105	410	0.221	42,288	20,795	15.973	5.98	95.833	7.616
4	17,527,718	9,287,864	390	0.273	44,942	23,815	17.4	6.046	72.034	8.297
5	35,164,881	4,125,557	1,472	0.321	23,889	2,802.688	7.729	12.129	63.559	3.685
6	26,184,509	16,095,296	588	0.41	44,531	27,372	30.153	9.032	48.375	14.378

#	Vol. (Int.)	Local Bg. Corr. Vol.	Area	Rf	Density	Local Bg. Corr. Den.	% band purity	% lane purity	Mol. Wt.	Rel. Quant. (w/ LB Corr. Vol.)
7	17,838,225	4,800,480	920	0.501	19,389	5,217.913	8.993	6.153	36.2	4.288
8	18,319,014	5,364,477	880	0.578	20,817	6,095.997	10.05	6.319	27.667	4.792

iBright™ Image Analysis Report

Katarina+ Chang

19 November 2022

pCofilin CHEMI_01212022_124603

Date:	21 January 2022 12:46:03PM
Mode:	Chemi Blots
Notes:	
Model:	FL1500
Instrument name:	2462619090234
Serial No:	2462619090234
Firmware version:	1.6.0
iBA version:	5.0
Image size:	676px X 540px
Image area:	118.63mm X 94.91mm
Optical Zoom:	1.9x
Digital Zoom:	1x
Focus level:	430
Resolution:	5 x 5
Exposure time:	17975 ms
Exposure mode:	Normal

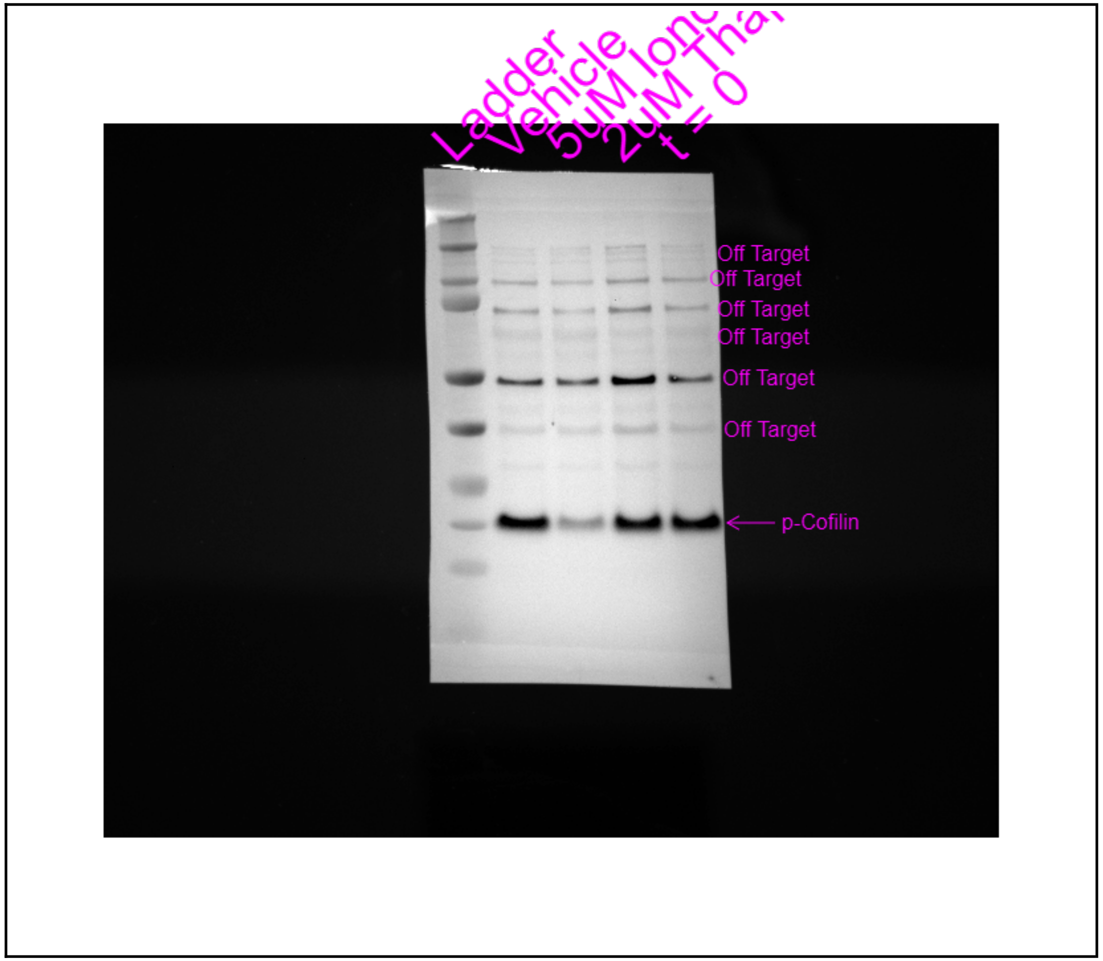

pCofilin CHEMI_01212022_124603

Date:	21 January 2022 12:46:03PM
Mode:	Chemi Blots
Notes:	
Model:	FL1500
Instrument name:	2462619090234
Serial No:	2462619090234
Firmware version:	1.6.0
iBA version:	5.0
Image size:	676px X 540px
Image area:	118.63mm X 94.91mm
Optical Zoom:	1.9x
Digital Zoom:	1x
Focus level:	430
Resolution:	5 x 5
Exposure time:	17975 ms
Exposure mode:	Normal

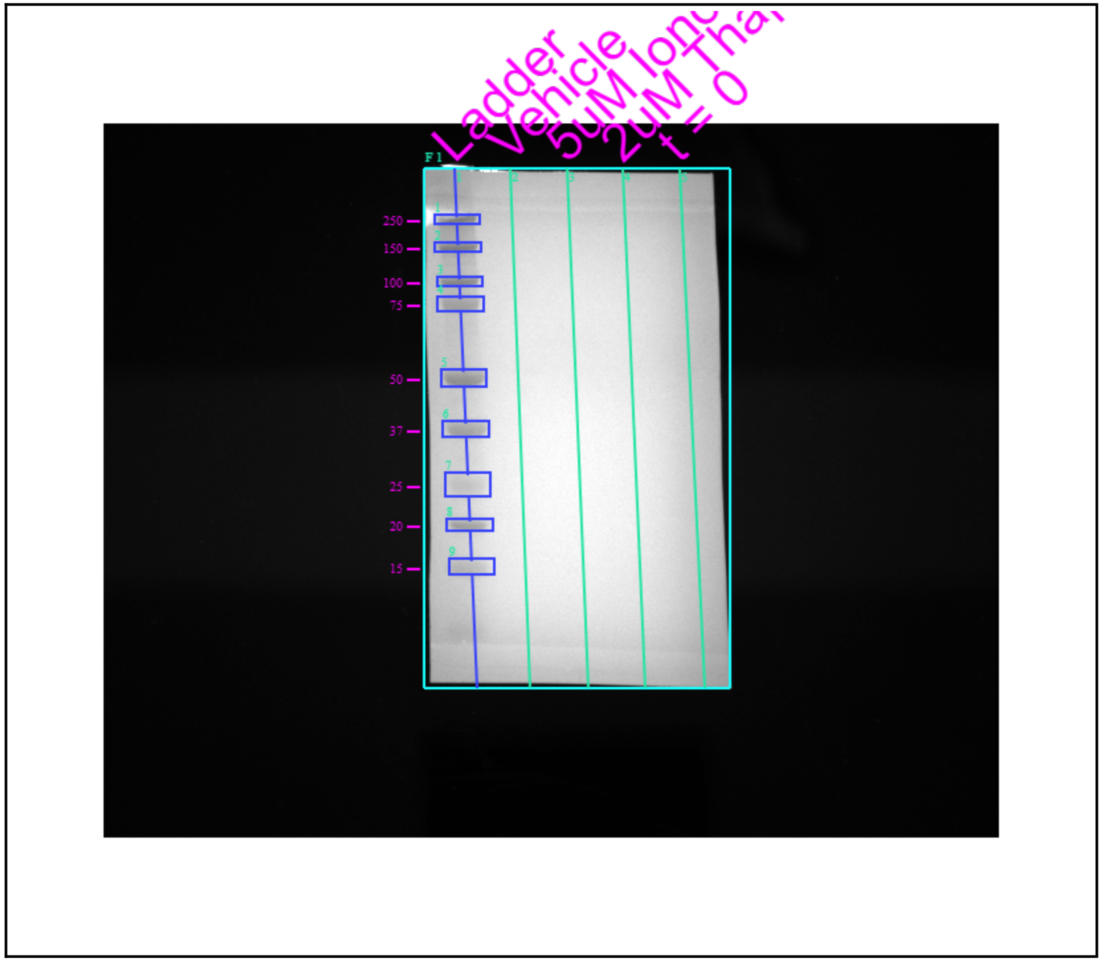

pCofilin CHEMI_01212022_124603

Date:	21 January 2022 12:46:03PM
Mode:	Chemi Blots
Notes:	
Model:	FL1500
Instrument name:	2462619090234
Serial No:	2462619090234
Firmware version:	1.6.0
iBA version:	5.0
Image size:	676px X 540px
Image area:	118.63mm X 94.91mm
Optical Zoom:	1.9x
Digital Zoom:	1x
Focus level:	430
Resolution:	5 x 5
Exposure time:	17975 ms
Exposure mode:	Normal

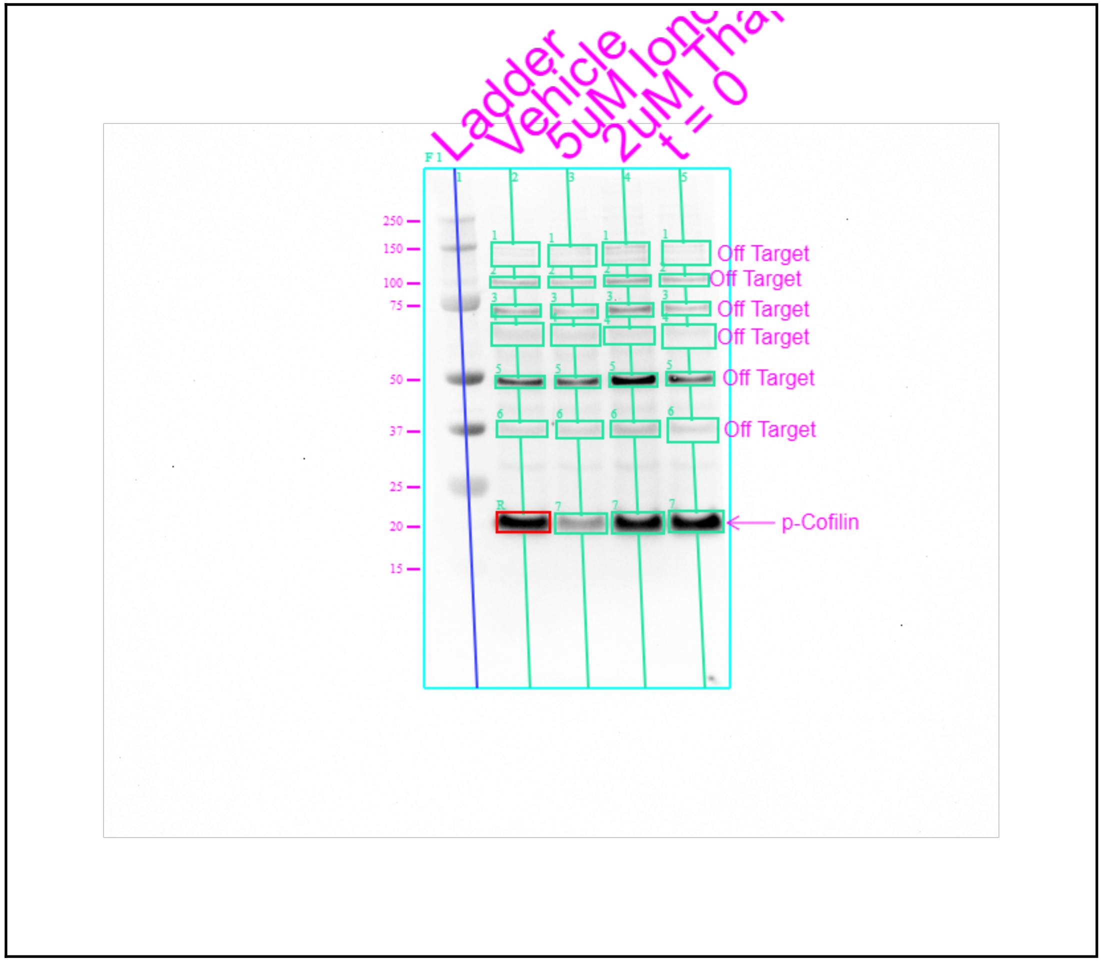

LANE AND BAND ANALYSIS DATA TABLE

pCofilin CHEMI_01212022_124603

Frame: 1
Channel: Membrane
Sensitivity: 100
Molecular Weight Analysis Regression Method : Point to Point

Lane 1 - Ladder

#	Vol. (Int.)	Local Bg. Corr. Vol.	Area	Rf	Density	Local Bg. Corr. Den.	% band purity	% lane purity	Rolling Bg. Corr. Vol.	Rolling Bg. Corr. Den.	Mol. Wt.
1	9,587,836	1,770,259	280	0.097	34,242	6,322.356	12.477	3.814	2,321,152	8,289.829	250
2	10,196,021	1,810,481	288	0.15	35,402	6,286.393	12.362	3.779	2,299,648	7,984.889	150
3	9,498,231	1,778,510	280	0.216	33,922	6,351.822	8.672	2.651	1,613,312	5,761.829	100
4	13,477,856	1,960,490	432	0.26	31,198	4,538.173	9.051	2.767	1,683,712	3,897.481	75
5	15,873,033	3,193,951	490	0.402	32,393	6,518.269	16.927	5.174	3,148,800	6,426.122	50
6	14,019,412	2,531,053	468	0.501	29,956	5,408.234	13.684	4.183	2,545,664	5,439.453	37
7	17,159,989	1,498,743	665	0.608	25,804	2,253.75	7.977	2.439	1,484,032	2,231.627	25
8	10,494,649	1,873,631	360	0.684	29,151	5,204.531	10.427	3.187	1,939,712	5,388.089	20
9	12,510,498	1,457,523	455	0.766	27,495	3,203.347	8.422	2.574	1,566,720	3,443.341	15

Frame: 1
Channel: Chemi
Sensitivity: 100
Molecular Weight Analysis Regression Method : Point to Point

Lane 2 - Vehicle

#	Vol. (Int.)	Local Bg. Corr. Vol.	Area	Rf	Density	Local Bg. Corr. Den.
1	1,713,158	557,703	703	0.165	2,436.925	793.319
2	1,862,722	1,108,599	342	0.219	5,446.556	3,241.518
3	2,500,271	1,497,810	380	0.272	6,579.661	3,941.605
4	3,073,590	930,422	720	0.318	4,268.875	1,292.253
5	5,785,308	4,413,974	380	0.41	15,224	11,615
6	2,227,511	823,280	507	0.501	4,393.513	1,623.828
7	16,914,176	13,412,861	656	0.679	25,783	20,446

#	% band purity	% lane purity	Rolling Bg. Corr. Vol.	Rolling Bg. Corr. Den.	Mol. Wt.	Rel. Quant. (w/ LB Corr. Vol.)

#	% band purity	% lane purity	Rolling Bg. Corr. Vol.	Rolling Bg. Corr. Den.	Mol. Wt.	Rel. Quant. (w/ LB Corr. Vol.)
1	1.64	1.536	394,496	561.161	138.462	0.042
2	4.483	4.199	1,078,272	3,152.842	98.529	0.083
3	6.037	5.655	1,452,032	3,821.137	72.768	0.112
4	3.073	2.878	739,072	1,026.489	64.732	0.069
5	19.21	17.994	4,620,288	12,158	49	0.329
6	3.411	3.195	820,480	1,618.304	37	0.061
7	62.146	58.212	14,947,328	22,785	20.333	1

Lane 3 - 5uM Ionomycin

#	Vol. (Int.)	Local Bg. Corr. Vol.	Area	Rf	Density	Local Bg. Corr. Den.
1	1,868,062	614,139	629	0.168	2,969.892	976.375
2	1,600,997	806,271	324	0.219	4,941.349	2,488.491
3	2,000,147	913,005	396	0.275	5,050.876	2,305.568
4	2,813,170	809,594	646	0.321	4,354.752	1,253.243
5	5,033,378	3,678,891	350	0.41	14,381	10,511
6	2,325,962	808,327	504	0.501	4,615.004	1,603.824
7	6,649,239	3,460,901	640	0.682	10,389	5,407.658

#	% band purity	% lane purity	Rolling Bg. Corr. Vol.	Rolling Bg. Corr. Den.	Mol. Wt.	Rel. Quant. (w/ LB Corr. Vol.)
1	4.499	3.7	551,680	877.075	136.538	0.046
2	6.344	5.217	777,984	2,401.185	98.529	0.06
3	6.881	5.659	843,776	2,130.747	72.321	0.068
4	5.035	4.141	617,472	955.839	64.286	0.06
5	31.854	26.195	3,906,048	11,160	49	0.274
6	6.528	5.368	800,512	1,588.317	37	0.06
7	38.858	31.955	4,764,928	7,445.2	20.167	0.258

Lane 4 - 2uM Thapsigargin

#	Vol. (Int.)	Local Bg. Corr. Vol.	Area	Rf	Density	Local Bg. Corr. Den.
1	2,720,723	1,281,575	648	0.163	4,198.647	1,977.74
2	2,369,283	1,429,260	324	0.216	7,312.602	4,411.299
3	3,548,285	2,249,698	396	0.272	8,960.316	5,681.057
4	2,456,974	599,805	546	0.321	4,499.952	1,098.544

#	Vol. (Int.)	Local Bg. Corr. Vol.	Area	Rf	Density	Local Bg. Corr. Den.
5	10,130,805	8,275,314	407	0.407	24,891	20,332
6	3,274,822	1,635,856	494	0.501	6,629.194	3,311.45
7	16,352,057	12,150,530	680	0.682	24,047	17,868

#	% band purity	% lane purity	Rolling Bg. Corr. Vol.	Rolling Bg. Corr. Den.	Mol. Wt.	Rel. Quant. (w/ LB Corr. Vol.)
1	3.684	3.291	1,085,952	1,675.852	140.385	0.096
2	4.679	4.179	1,379,072	4,256.395	100	0.107
3	7.32	6.539	2,157,568	5,448.404	72.768	0.168
4	1.157	1.033	340,992	624.527	64.286	0.045
5	29.168	26.056	8,597,760	21,124	49.333	0.617
6	5.014	4.479	1,477,888	2,991.676	37	0.122
7	48.979	43.753	14,437,376	21,231	20.167	0.906

Lane 5 - t = 0

#	Vol. (Int.)	Local Bg. Corr. Vol.	Area	Rf	Density	Local Bg. Corr. Den.
1	1,628,690	534,640	703	0.163	2,316.771	760.513
2	1,699,394	1,007,084	380	0.214	4,472.089	2,650.222
3	1,904,642	1,129,452	407	0.27	4,679.71	2,775.066
4	2,412,002	772,924	779	0.323	3,096.28	992.201
5	5,222,419	4,084,223	407	0.405	12,831	10,034
6	2,647,714	1,159,149	741	0.504	3,573.163	1,564.305
7	17,547,000	14,432,705	714	0.679	24,575	20,213

#	% band purity	% lane purity	Rolling Bg. Corr. Vol.	Rolling Bg. Corr. Den.	Mol. Wt.	Rel. Quant. (w/ LB Corr. Vol.)
1	1.957	1.753	428,800	609.957	140.385	0.04
2	4.722	4.23	1,034,496	2,722.358	101.923	0.075
3	5.149	4.612	1,127,936	2,771.342	73.214	0.084
4	2.645	2.369	579,328	743.682	63.839	0.058
5	7.557	6.77	1,655,552	4,067.695	49.667	0.305
6	4.427	3.965	969,728	1,308.675	36.714	0.086
7	73.543	65.882	16,110,848	22,564	20.333	1.076

iBright™ Image Analysis Report

Katarina+ Chang

19 November 2022

Alpha Tubulin CHEMI_01232022_133851

Date:	23 January 2022 01:38:51PM
Mode:	Chemi Blots
Notes:	
Model:	FL1500
Instrument name:	2462619090234
Serial No:	2462619090234
Firmware version:	1.6.0
iBA version:	5.0
Image size:	615px X 491px
Image area:	112.7mm X 90.16mm
Optical Zoom:	2x
Digital Zoom:	1.1x
Focus level:	455
Resolution:	5 x 5
Exposure time:	15151 ms
Exposure mode:	Normal

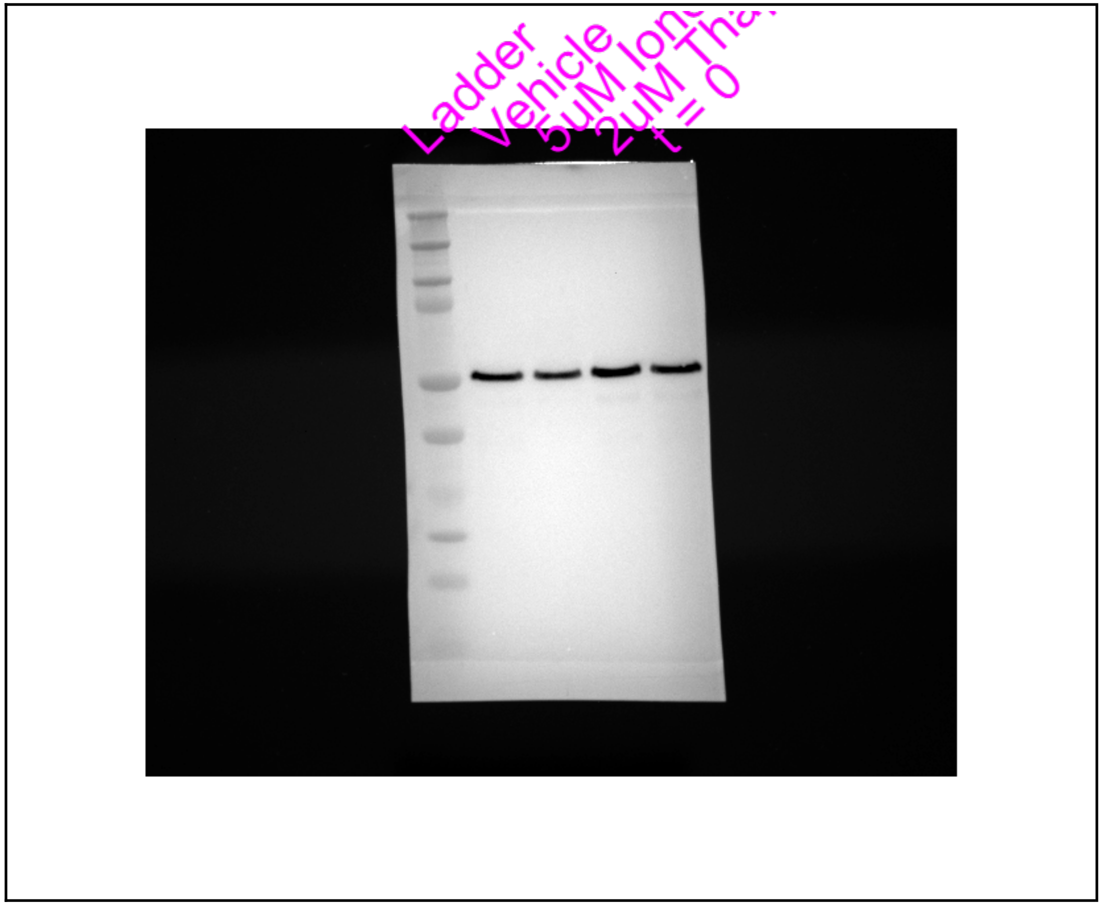

Alpha Tubulin CHEMI_01232022_133851

Date:	23 January 2022 01:38:51PM
Mode:	Chemi Blots
Notes:	
Model:	FL1500
Instrument name:	2462619090234
Serial No:	2462619090234
Firmware version:	1.6.0
iBA version:	5.0
Image size:	615px X 491px
Image area:	112.7mm X 90.16mm
Optical Zoom:	2x
Digital Zoom:	1.1x
Focus level:	455
Resolution:	5 x 5
Exposure time:	15151 ms
Exposure mode:	Normal

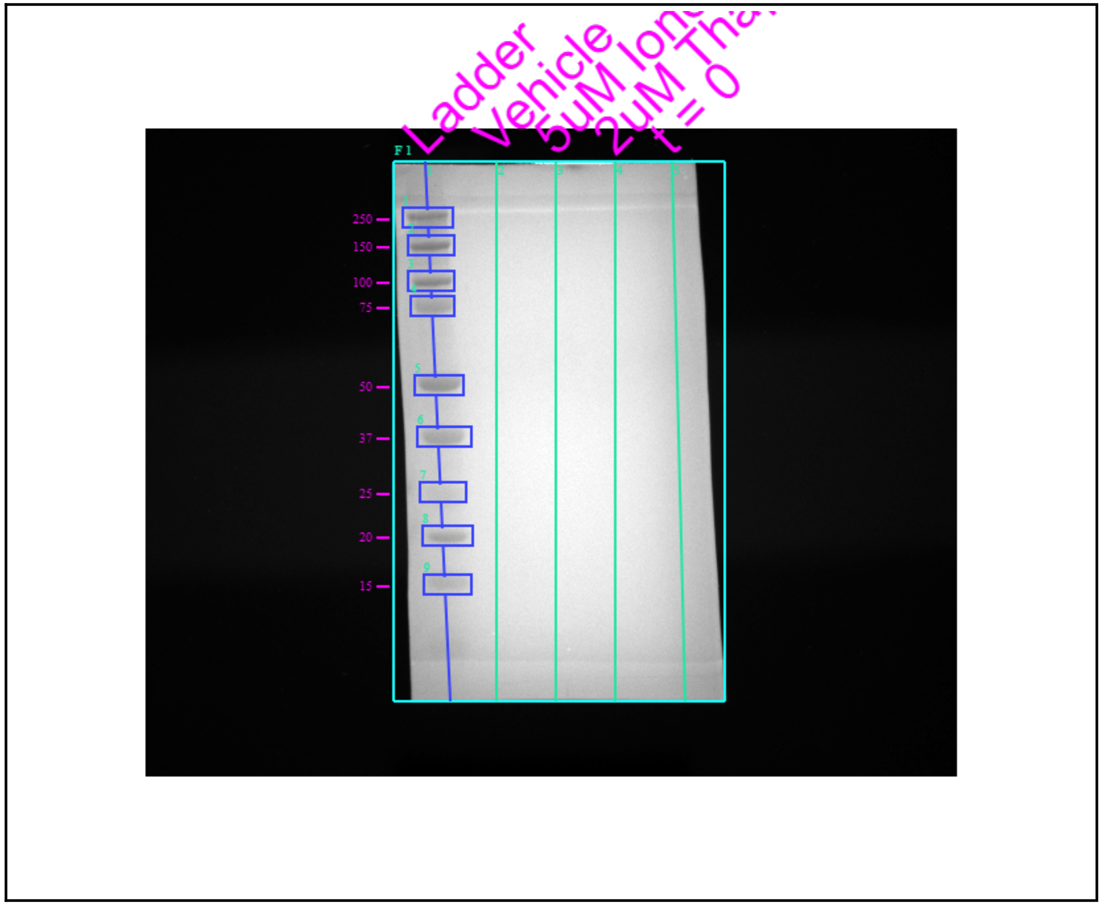

Alpha Tubulin CHEMI_01232022_133851

Date:	23 January 2022 01:38:51PM
Mode:	Chemi Blots
Notes:	
Model:	FL1500
Instrument name:	2462619090234
Serial No:	2462619090234
Firmware version:	1.6.0
iBA version:	5.0
Image size:	615px X 491px
Image area:	112.7mm X 90.16mm
Optical Zoom:	2x
Digital Zoom:	1.1x
Focus level:	455
Resolution:	5 x 5
Exposure time:	15151 ms
Exposure mode:	Normal

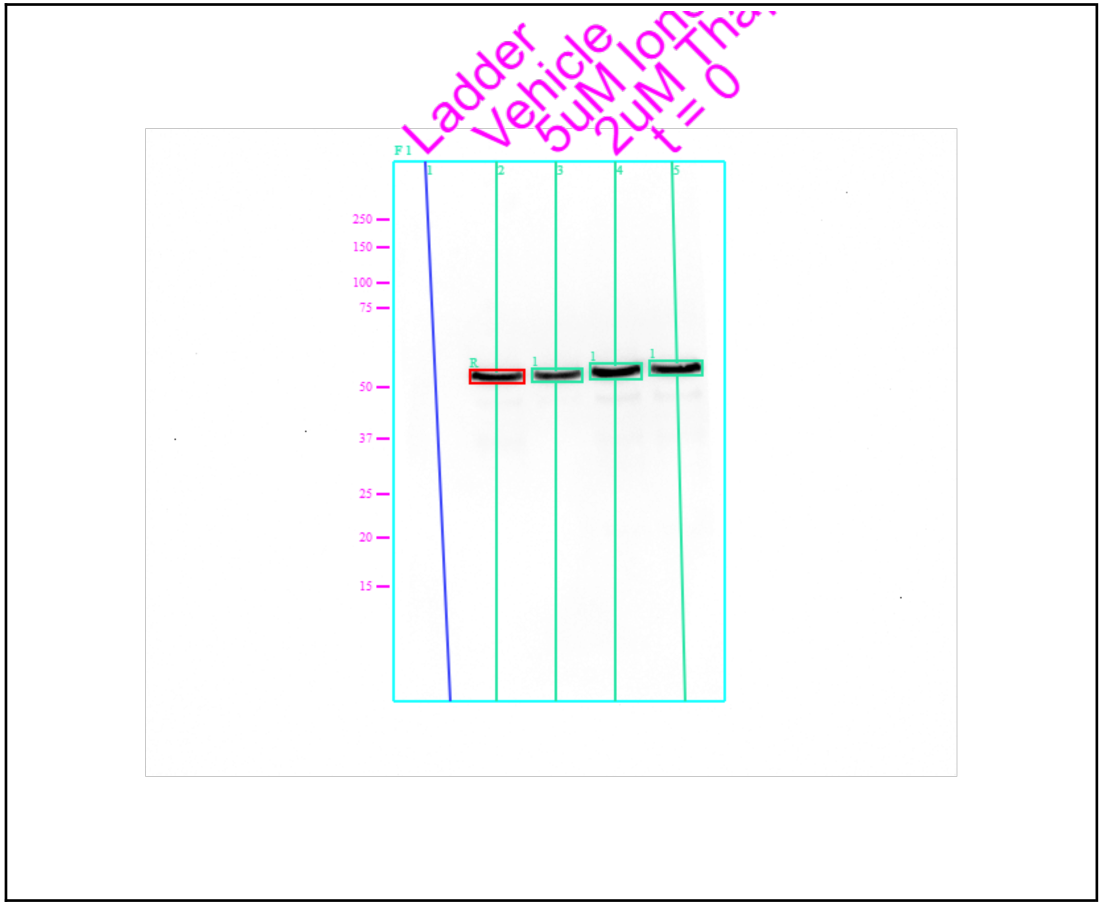

LANE AND BAND ANALYSIS DATA TABLE

Alpha Tubulin CHEMI_01232022_133851

Frame: 1
Channel: Membrane
Sensitivity: 100
Molecular Weight Analysis Regression Method : Point to Point

Lane 1 - Ladder

#	Vol. (Int.)	Local Bg. Corr. Vol.	Area	Rf	Density	Local Bg. Corr. Den.	% band purity	% lane purity	Rolling Bg. Corr. Vol.	Rolling Bg. Corr. Den.	Mol. Wt.
1	20,932,425	2,262,811	624	0.103	33,545	3,626.3	12.005	2.51	2,081,536	3,335.795	250
2	19,358,797	2,721,781	576	0.154	33,609	4,725.315	12.369	2.586	2,144,512	3,723.111	150
3	18,339,186	2,382,237	576	0.22	31,838	4,135.829	11.903	2.489	2,063,872	3,583.111	100
4	16,888,604	2,147,766	544	0.267	31,045	3,948.1	10.708	2.239	1,856,512	3,412.706	75
5	18,997,198	3,039,789	608	0.413	31,245	4,999.654	16.489	3.448	2,859,008	4,702.316	50
6	19,184,965	2,757,355	672	0.509	28,549	4,103.208	12.792	2.675	2,217,984	3,300.571	37
7	15,302,152	909,344	576	0.611	26,566	1,578.723	5.187	1.085	899,328	1,561.333	25
8	17,501,934	1,997,198	624	0.692	28,047	3,200.639	10.462	2.188	1,814,016	2,907.077	20
9	16,911,806	1,402,416	592	0.782	28,567	2,368.947	8.084	1.69	1,401,600	2,367.568	15

Frame: 1
Channel: Chemi
Sensitivity: 100
Molecular Weight Analysis Regression Method : Point to Point

Lane 2 - Vehicle

#	Vol. (Int.)	Local Bg. Corr. Vol.	Area	Rf	Density	Local Bg. Corr. Den.
1	8,721,782	7,665,671	462	0.399	18,878	16,592

#	% band purity	% lane purity	Rolling Bg. Corr. Vol.	Rolling Bg. Corr. Den.	Mol. Wt.	Rel. Quant. (w/ LB Corr. Vol.)
1	100	87.452	8,251,392	17,860	52.5	1

Lane 3 - 5uM Ionomycin

#	Vol. (Int.)	Local Bg. Corr. Vol.	Area	Rf	Density	Local Bg. Corr. Den.
1	6,920,439	5,864,429	429	0.396	16,131	13,669

#	% band purity	% lane purity	Rolling Bg. Corr. Vol.	Rolling Bg. Corr. Den.	Mol. Wt.	Rel. Quant. (w/ LB Corr. Vol.)

#	% band purity	% lane purity	Rolling Bg. Corr. Vol.	Rolling Bg. Corr. Den.	Mol. Wt.	Rel. Quant. (w/ LB Corr. Vol.)
1	100	83.207	6,261,248	14,594	52.917	0.765

Lane 4 - 2uM Thapsigargin

#	Vol. (Int.)	Local Bg. Corr. Vol.	Area	Rf	Density	Local Bg. Corr. Den.
1	11,410,934	9,929,773	520	0.389	21,944	19,095

#	% band purity	% lane purity	Rolling Bg. Corr. Vol.	Rolling Bg. Corr. Den.	Mol. Wt.	Rel. Quant. (w/ LB Corr. Vol.)
1	100	84.284	10,612,480	20,408	54.167	1.295

Lane 5 - t = 0

#	Vol. (Int.)	Local Bg. Corr. Vol.	Area	Rf	Density	Local Bg. Corr. Den.
1	9,829,101	8,732,871	492	0.381	19,977	17,749

#	% band purity	% lane purity	Rolling Bg. Corr. Vol.	Rolling Bg. Corr. Den.	Mol. Wt.	Rel. Quant. (w/ LB Corr. Vol.)
1	100	61.452	2,132,736	4,334.829	55.417	1.139

iBright™ Image Analysis Report

Katarina+ Chang

19 November 2022

GAPDH CHEMI_01232022_134418

Date:	23 January 2022 01:44:18PM
Mode:	Chemi Blots
Notes:	
Model:	FL1500
Instrument name:	2462619090234
Serial No:	2462619090234
Firmware version:	1.6.0
iBA version:	5.0
Image size:	563px X 450px
Image area:	112.7mm X 90.16mm
Optical Zoom:	2x
Digital Zoom:	1.2x
Focus level:	455
Resolution:	5 x 5
Exposure time:	5823 ms
Exposure mode:	Normal

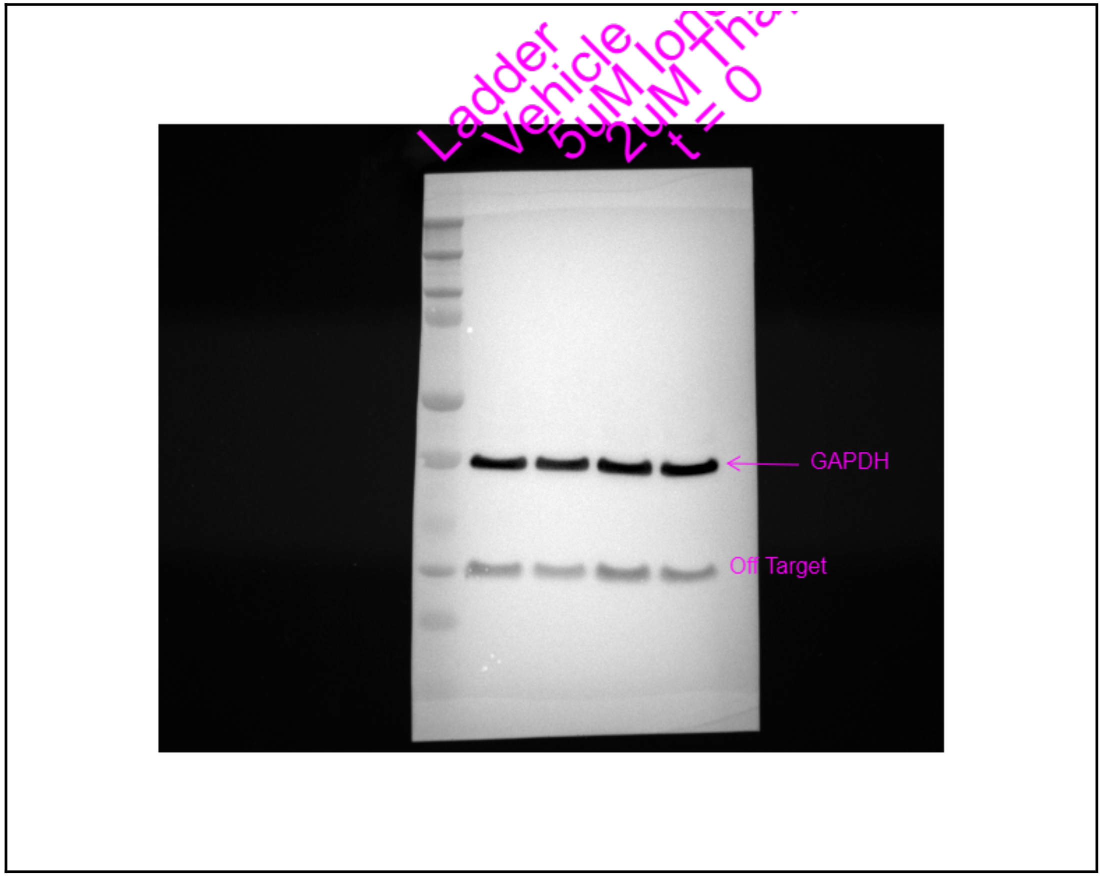

GAPDH CHEMI_01232022_134418

Date:	23 January 2022 01:44:18PM
Mode:	Chemi Blots
Notes:	
Model:	FL1500
Instrument name:	2462619090234
Serial No:	2462619090234
Firmware version:	1.6.0
iBA version:	5.0
Image size:	563px X 450px
Image area:	112.7mm X 90.16mm
Optical Zoom:	2x
Digital Zoom:	1.2x
Focus level:	455
Resolution:	5 x 5
Exposure time:	5823 ms
Exposure mode:	Normal

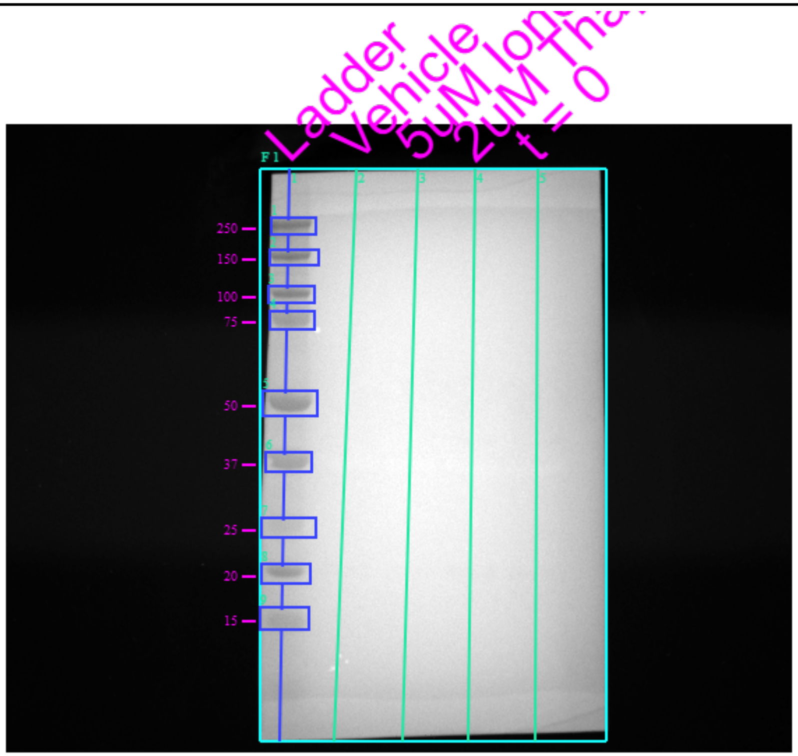

GAPDH CHEMI_01232022_134418

Date:	23 January 2022 01:44:18PM
Mode:	Chemi Blots
Notes:	
Model:	FL1500
Instrument name:	2462619090234
Serial No:	2462619090234
Firmware version:	1.6.0
iBA version:	5.0
Image size:	563px X 450px
Image area:	112.7mm X 90.16mm
Optical Zoom:	2x
Digital Zoom:	1.2x
Focus level:	455
Resolution:	5 x 5
Exposure time:	5823 ms
Exposure mode:	Normal

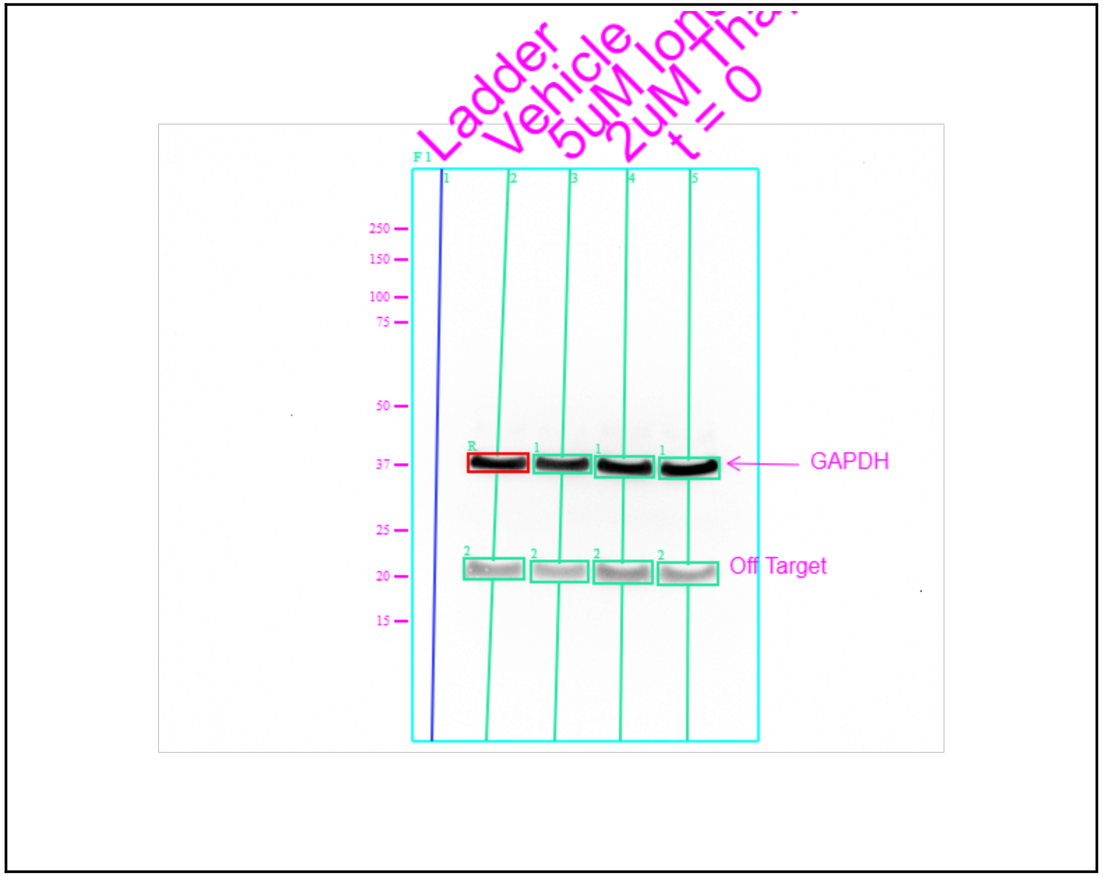

LANE AND BAND ANALYSIS DATA TABLE

GAPDH CHEMI_01232022_134418

Frame: 1
Channel: Membrane
Sensitivity: 100
Molecular Weight Analysis Regression Method : Point to Point

Lane 1 - Ladder

#	Vol. (Int.)	Local Bg. Corr. Vol.	Area	Rf	Density	Local Bg. Corr. Den.	% band purity	% lane purity	Rolling Bg. Corr. Vol.	Rolling Bg. Corr. Den.	Mol. Wt.
1	15,815,073	767,749	429	0.1	36,864	1,789.627	13.468	1.441	1,204,736	2,808.242	250
2	14,964,243	683,969	432	0.154	34,639	1,583.264	8.262	0.884	739,072	1,710.815	150
3	15,128,098	597,433	442	0.22	34,226	1,351.659	10.034	1.074	897,536	2,030.624	100
4	14,714,318	120,036	462	0.263	31,849	259.818	2.035	0.218	182,016	393.974	75
5	24,828,849	1,082,899	760	0.41	32,669	1,424.867	29.094	3.114	2,602,496	3,424.337	50
6	15,735,516	498,399	510	0.512	30,853	977.255	12.495	1.337	1,117,696	2,191.561	37
7	16,893,350	18,291	600	0.627	28,155	30.485	4.261	0.456	381,184	635.307	25
8	16,762,641	319,104	540	0.707	31,041	590.935	12.924	1.383	1,156,096	2,140.919	20
9	19,430,369	31,331	612	0.785	31,748	51.196	7.427	0.795	664,320	1,085.49	15

Frame: 1
Channel: Chemi
Sensitivity: 100
Molecular Weight Analysis Regression Method : Point to Point

Lane 2 - Vehicle

#	Vol. (Int.)	Local Bg. Corr. Vol.	Area	Rf	Density	Local Bg. Corr. Den.
1	13,540,370	11,927,630	616	0.512	21,981	19,363
2	5,599,881	4,791,611	704	0.698	7,954.376	6,806.267

#	% band purity	% lane purity	Rolling Bg. Corr. Vol.	Rolling Bg. Corr. Den.	Mol. Wt.	Rel. Quant. (w/ LB Corr. Vol.)
1	71.346	69.507	12,604,928	20,462	37	1
2	28.654	27.915	5,062,400	7,190.909	20.606	0.402

Lane 3 - 5uM Ionomycin

#	Vol. (Int.)	Local Bg. Corr. Vol.	Area	Rf	Density	Local Bg. Corr. Den.

#	Vol. (Int.)	Local Bg. Corr. Vol.	Area	Rf	Density	Local Bg. Corr. Den.
1	12,222,261	9,889,737	588	0.515	20,786	16,819
2	4,407,874	3,343,253	672	0.702	6,559.336	4,975.079

#	% band purity	% lane purity	Rolling Bg. Corr. Vol.	Rolling Bg. Corr. Den.	Mol. Wt.	Rel. Quant. (w/ LB Corr. Vol.)
1	74.765	69.942	11,018,240	18,738	36.745	0.829
2	25.235	23.607	3,718,912	5,534.095	20.303	0.28

Lane 4 - 2uM Thapsigargin

#	Vol. (Int.)	Local Bg. Corr. Vol.	Area	Rf	Density	Local Bg. Corr. Den.
1	15,239,864	12,414,358	688	0.52	22,150	18,044
2	6,639,224	5,278,925	731	0.705	9,082.386	7,221.512

#	% band purity	% lane purity	Rolling Bg. Corr. Vol.	Rolling Bg. Corr. Den.	Mol. Wt.	Rel. Quant. (w/ LB Corr. Vol.)
1	70.601	67.441	13,830,144	20,101	36.234	1.041
2	29.399	28.083	5,758,976	7,878.216	20.152	0.443

Lane 5 - t = 0

#	Vol. (Int.)	Local Bg. Corr. Vol.	Area	Rf	Density	Local Bg. Corr. Den.
1	17,317,097	15,482,677	704	0.522	24,598	21,992
2	5,416,449	4,543,350	748	0.707	7,241.242	6,073.998

#	% band purity	% lane purity	Rolling Bg. Corr. Vol.	Rolling Bg. Corr. Den.	Mol. Wt.	Rel. Quant. (w/ LB Corr. Vol.)
1	77.722	76.918	16,236,800	23,063	35.979	1.298
2	22.278	22.048	4,654,080	6,222.032	20	0.381

iBright™ Image Analysis Report

Katarina+ Chang

19 November 2022

total cofilin CHEMI_01212022_123145

Date:	21 January 2022 12:31:45PM
Mode:	Chemi Blots
Notes:	
Model:	FL1500
Instrument name:	2462619090234
Serial No:	2462619090234
Firmware version:	1.6.0
iBA version:	5.0
Image size:	615px X 491px
Image area:	112.7mm X 90.16mm
Optical Zoom:	2x
Digital Zoom:	1.1x
Focus level:	455
Resolution:	5 x 5
Exposure time:	2938 ms
Exposure mode:	Normal

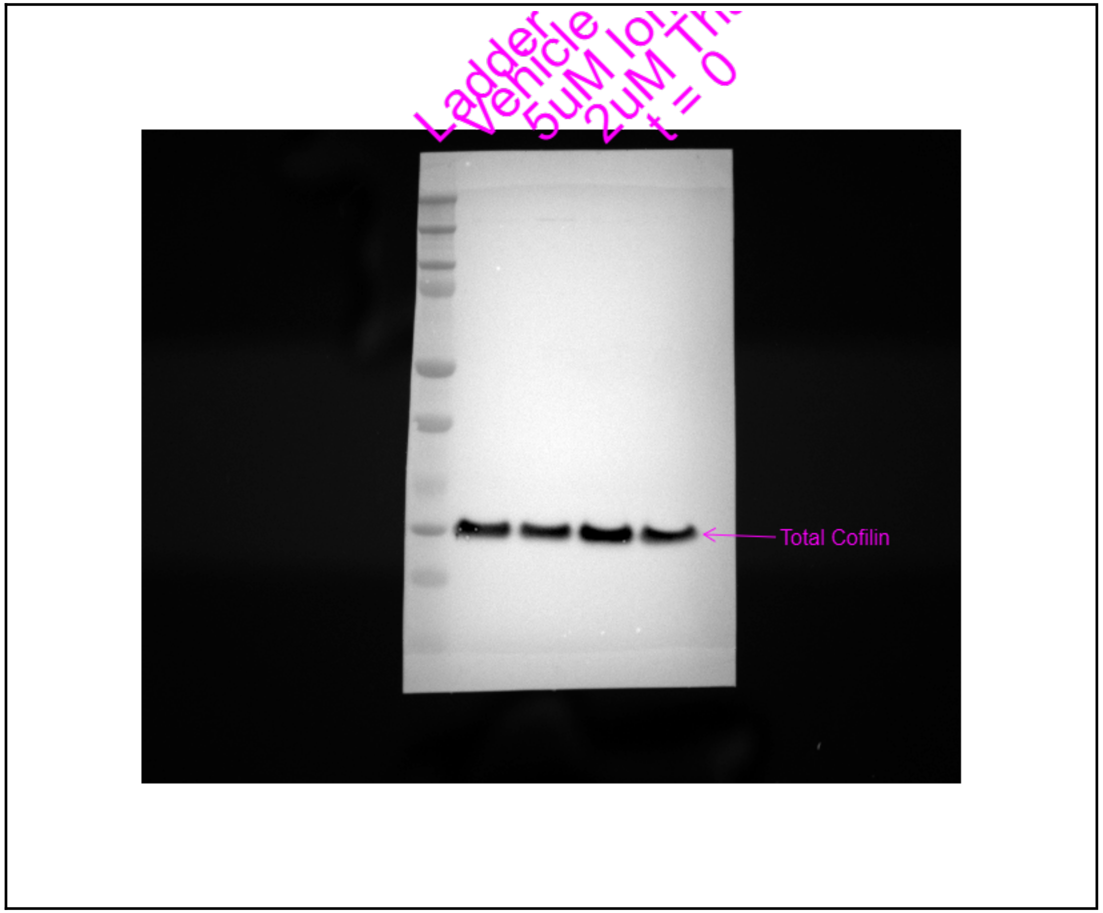

total cofilin CHEMI_01212022_123145

Date:	21 January 2022 12:31:45PM
Mode:	Chemi Blots
Notes:	
Model:	FL1500
Instrument name:	2462619090234
Serial No:	2462619090234
Firmware version:	1.6.0
iBA version:	5.0
Image size:	615px X 491px
Image area:	112.7mm X 90.16mm
Optical Zoom:	2x
Digital Zoom:	1.1x
Focus level:	455
Resolution:	5 x 5
Exposure time:	2938 ms
Exposure mode:	Normal

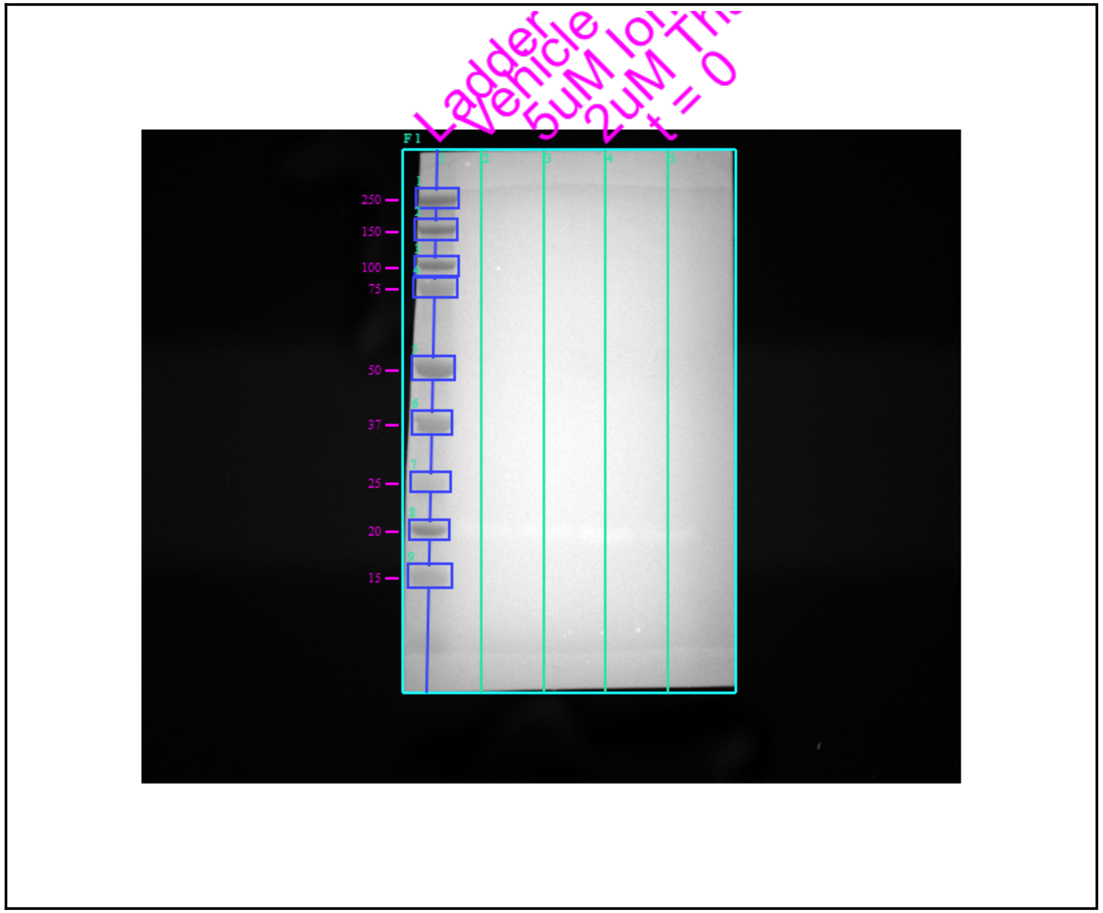

total cofilin CHEMI_01212022_123145

Date:	21 January 2022 12:31:45PM
Mode:	Chemi Blots
Notes:	
Model:	FL1500
Instrument name:	2462619090234
Serial No:	2462619090234
Firmware version:	1.6.0
iBA version:	5.0
Image size:	615px X 491px
Image area:	112.7mm X 90.16mm
Optical Zoom:	2x
Digital Zoom:	1.1x
Focus level:	455
Resolution:	5 x 5
Exposure time:	2938 ms
Exposure mode:	Normal

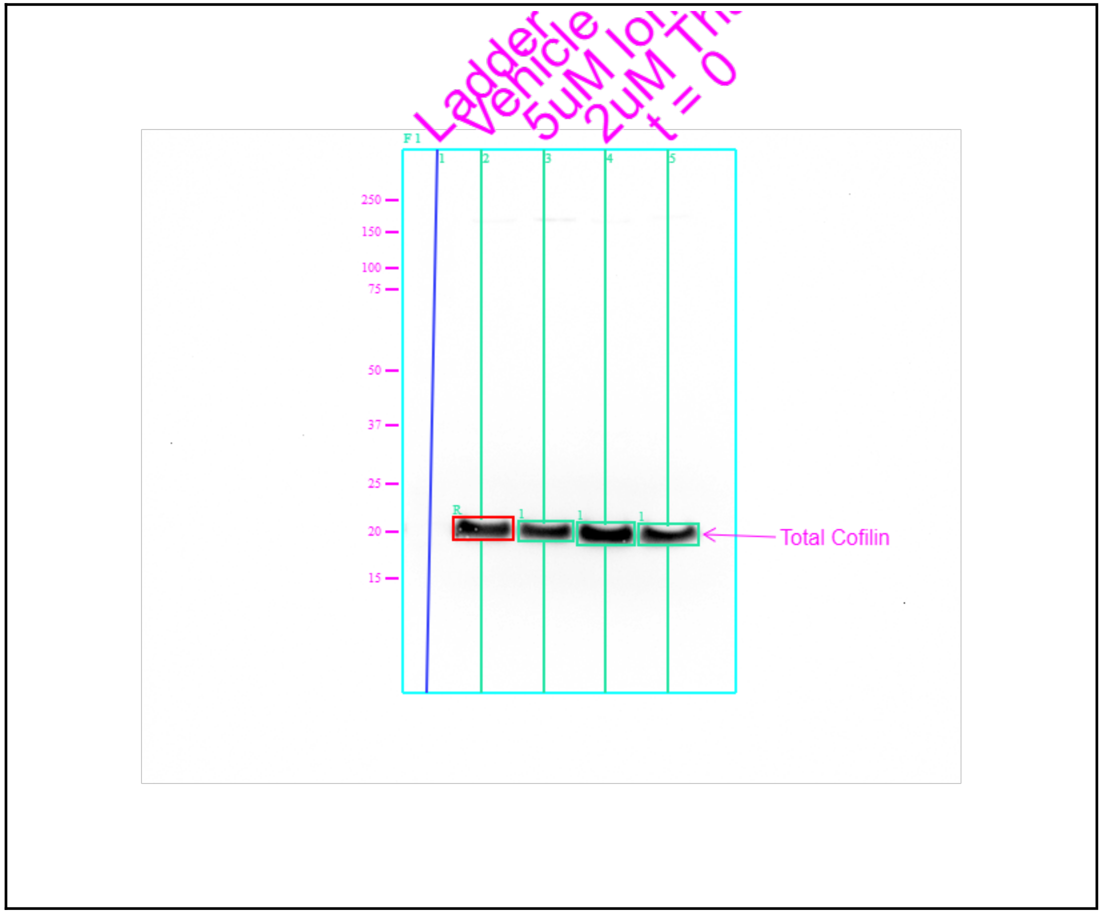

LANE AND BAND ANALYSIS DATA TABLE

total cofilin CHEMI_01212022_123145

Frame: 1
Channel: Membrane
Sensitivity: 100
Molecular Weight Analysis Regression Method : Point to Point

Lane 1 - Ladder

#	Vol. (Int.)	Local Bg. Corr. Vol.	Area	Rf	Density	Local Bg. Corr. Den.	% band purity	% lane purity	Mol. Wt.
1	20,362,981	917,971	528	0.088	38,566	1,738.583	12.814	3.718	250
2	21,006,040	920,644	561	0.147	37,443	1,641.077	12.851	3.835	150
3	19,232,400	842,365	544	0.213	35,353	1,548.466	11.759	3.512	100
4	19,088,989	826,225	544	0.252	35,090	1,518.797	11.533	3.485	75
5	20,878,832	640,815	627	0.402	33,299	1,022.034	8.945	3.812	50
6	18,303,305	836,939	589	0.502	31,075	1,420.95	11.683	3.342	37
7	14,135,814	259,957	496	0.61	28,499	524.109	3.629	2.581	25
8	15,779,179	1,480,020	496	0.699	31,812	2,983.912	20.66	2.881	20
9	19,618,211	438,814	646	0.784	30,368	679.279	6.125	3.582	15

Frame: 1
Channel: Chemi
Sensitivity: 100
Molecular Weight Analysis Regression Method : Point to Point

Lane 2 - Vehicle

#	Vol. (Int.)	Local Bg. Corr. Vol.	Area	Rf	Density	Local Bg. Corr. Den.	% band purity	% lane purity	Mol. Wt.	Rel. Quant. (w/ LB Corr. Vol.)
1	19,712,738	16,922,648	828	0.696	23,807	20,437	100	79.127	20.139	1

Lane 3 - 5uM Ionomycin

#	Vol. (Int.)	Local Bg. Corr. Vol.	Area	Rf	Density	Local Bg. Corr. Den.	% band purity	% lane purity	Mol. Wt.	Rel. Quant. (w/ LB Corr. Vol.)
1	16,635,591	13,136,729	672	0.701	24,755	19,548	100	68.744	19.857	0.776

Lane 4 - 2uM Thapsigargin

#	Vol. (Int.)	Local Bg. Corr. Vol.	Area	Rf	Density	Local Bg. Corr. Den.	% band purity	% lane purity	Mol. Wt.	Rel. Quant. (w/ LB Corr. Vol.)

#	Vol. (Int.)	Local Bg. Corr. Vol.	Area	Rf	Density	Local Bg. Corr. Den.	% band purity	% lane purity	Mol. Wt.	Rel. Quant. (w/ LB Corr. Vol.)
1	23,795,962	19,598,561	792	0.706	30,045	24,745	100	76.33	19.571	1.158

Lane 5 - t = 0

#	Vol. (Int.)	Local Bg. Corr. Vol.	Area	Rf	Density	Local Bg. Corr. Den.	% band purity	% lane purity	Mol. Wt.	Rel. Quant. (w/ LB Corr. Vol.)
1	17,600,510	15,117,576	782	0.708	22,507	19,331	100	77.193	19.429	0.893

iBright™ Image Analysis Report

Katarina+ Chang

19 November 2022

detyrosinated tubulin CHEMI_0120202 2_124633

Date:	20 January 2022 12:46:33PM
Mode:	Chemi Blots
Notes:	
Model:	FL1500
Instrument name:	2462619090234
Serial No:	2462619090234
Firmware version:	1.6.0
iBA version:	5.0
Image size:	563px X 450px
Image area:	112.7mm X 90.16mm
Optical Zoom:	2x
Digital Zoom:	1.2x
Focus level:	455
Resolution:	5 x 5
Exposure time:	75246 ms
Exposure mode:	Normal

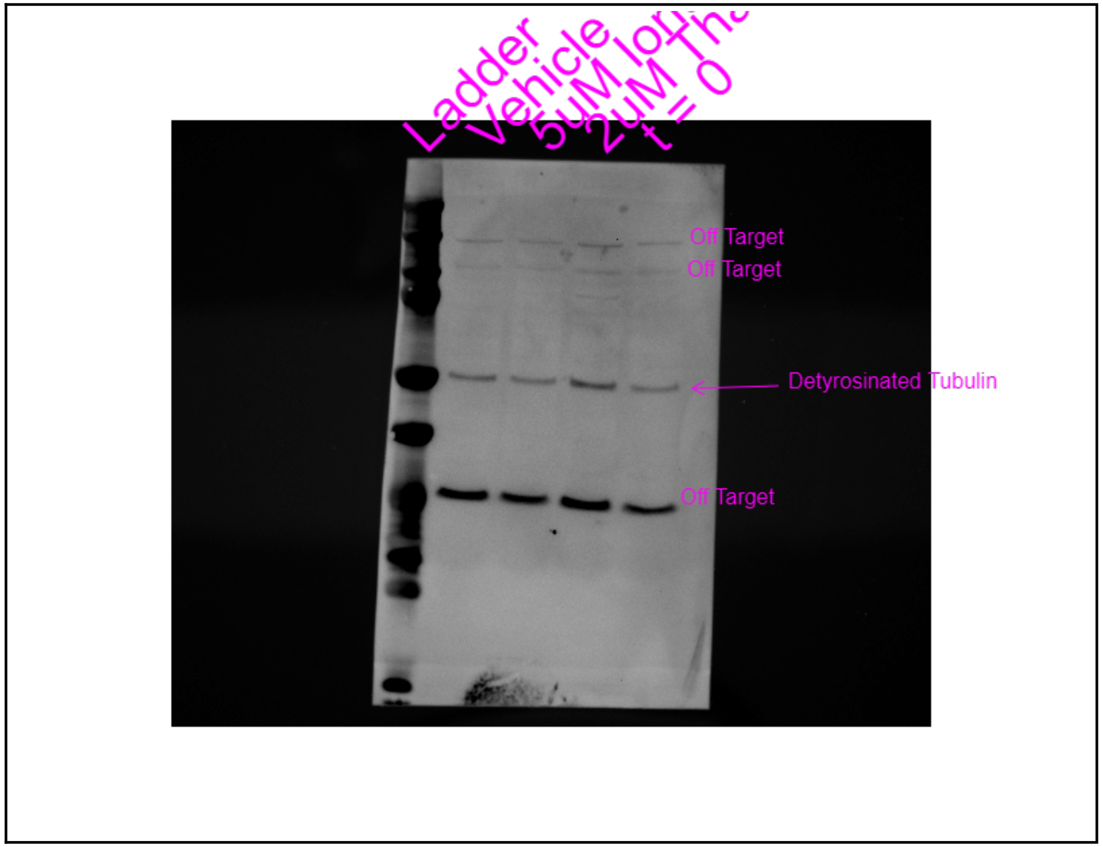

detyrosinated tubulin CHEMI_0120202 2_124633

Date:	20 January 2022 12:46:33PM
Mode:	Chemi Blots
Notes:	
Model:	FL1500
Instrument name:	2462619090234
Serial No:	2462619090234
Firmware version:	1.6.0
iBA version:	5.0
Image size:	563px X 450px
Image area:	112.7mm X 90.16mm
Optical Zoom:	2x
Digital Zoom:	1.2x
Focus level:	455
Resolution:	5 x 5
Exposure time:	75246 ms
Exposure mode:	Normal

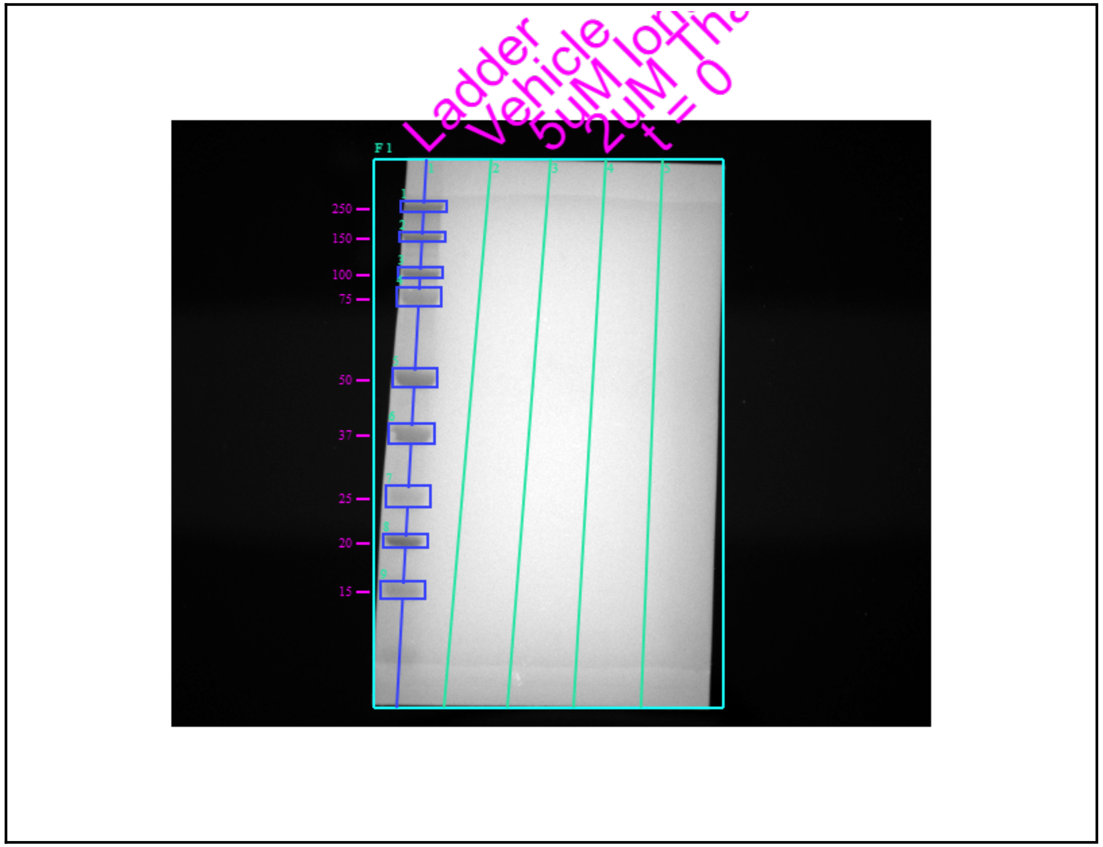

detyrosinated tubulin CHEMI_01202022_124633

Date:	20 January 2022 12:46:33PM
Mode:	Chemi Blots
Notes:	
Model:	FL1500
Instrument name:	2462619090234
Serial No:	2462619090234
Firmware version:	1.6.0
iBA version:	5.0
Image size:	563px X 450px
Image area:	112.7mm X 90.16mm
Optical Zoom:	2x
Digital Zoom:	1.2x
Focus level:	455
Resolution:	5 x 5
Exposure time:	75246 ms
Exposure mode:	Normal

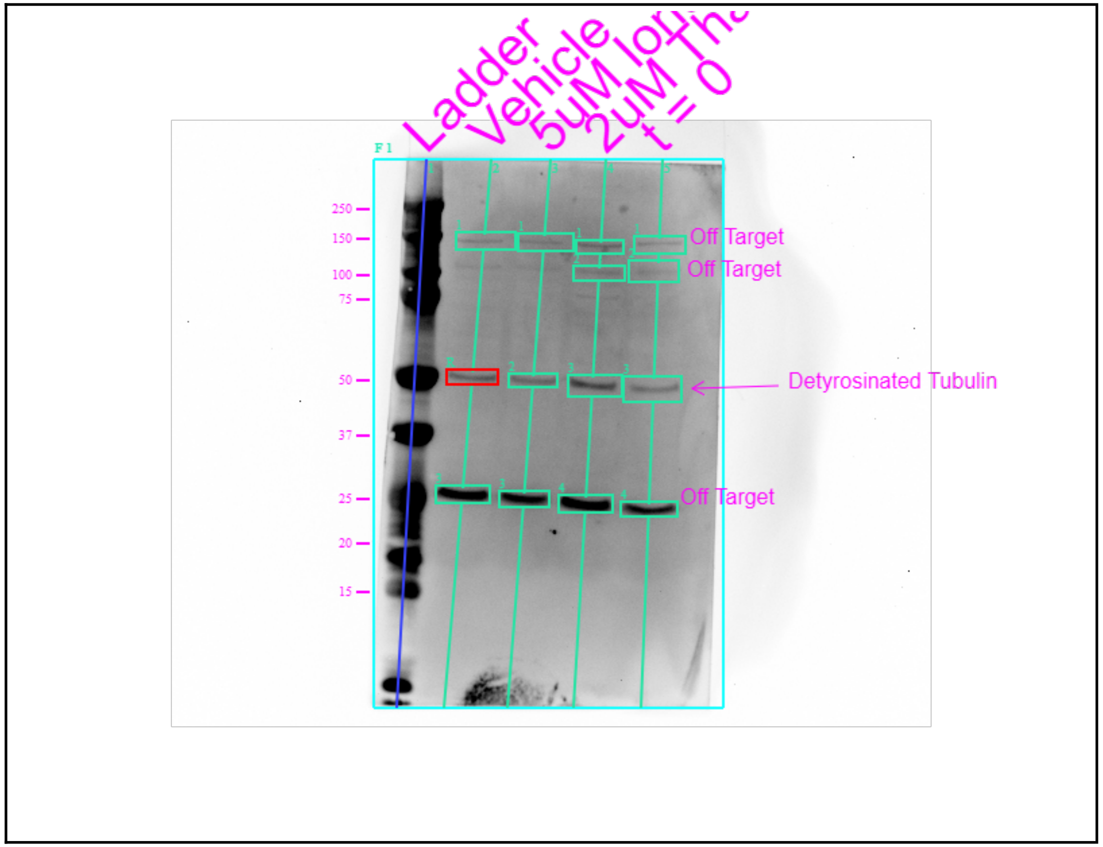

LANE AND BAND ANALYSIS DATA TABLE

detyrosinated tubulin CHEMI_01202022_124633

Frame: 1
Channel: Membrane
Sensitivity: 100
Molecular Weight Analysis Regression Method : Point to Point

Lane 1 - Ladder

#	Vol. (Int.)	Local Bg. Corr. Vol.	Area	Rf	Density	Local Bg. Corr. Den.	% band purity	% lane purity	Rolling Bg. Corr. Vol.	Rolling Bg. Corr. Den.	Mol. Wt.
1	12,736,733	815,780	315	0.086	40,434	2,589.779	11.474	3.089	2,598,912	8,250.514	250
2	10,890,168	775,848	280	0.14	38,893	2,770.886	8.444	2.273	1,912,576	6,830.629	150
3	11,468,228	774,440	306	0.206	37,477	2,530.85	9.395	2.529	2,127,872	6,953.83	100
4	17,666,189	557,904	510	0.251	34,639	1,093.931	11.203	3.016	2,537,472	4,975.435	75
5	18,015,031	1,434,563	510	0.398	35,323	2,812.869	16.284	4.383	3,688,192	7,231.749	50
6	18,408,817	1,099,827	560	0.499	32,872	1,963.977	14.232	3.831	3,223,552	5,756.343	37
7	17,334,091	433,654	578	0.614	29,989	750.267	6.357	1.711	1,439,744	2,490.907	25
8	13,460,000	2,070,433	374	0.695	35,989	5,535.919	13.785	3.711	3,122,176	8,348.064	20
9	15,872,314	628,953	476	0.784	33,345	1,321.331	8.827	2.376	1,999,360	4,200.336	15

Frame: 1
Channel: Chemi
Sensitivity: 100
Molecular Weight Analysis Regression Method : Point to Point

Lane 2 - Vehicle

#	Vol. (Int.)	Local Bg. Corr. Vol.	Area	Rf	Density	Local Bg. Corr. Den.
1	18,390,644	1,181,894	630	0.147	29,191	1,876.022
2	15,615,662	2,282,130	468	0.396	33,366	4,876.348
3	26,890,101	10,822,062	574	0.609	46,846	18,853

#	% band purity	% lane purity	Rolling Bg. Corr. Vol.	Rolling Bg. Corr. Den.	Mol. Wt.	Rel. Quant. (w/ LB Corr. Vol.)
1	8.081	3.063	1,330,944	2,112.61	144.444	0.518
2	17.128	6.491	2,820,864	6,027.487	50.417	1
3	74.791	28.344	12,317,440	21,458	25.511	4.742

Lane 3 - 5uM Ionomycin

#	Vol. (Int.)	Local Bg. Corr. Vol.	Area	Rf	Density	Local Bg. Corr. Den.	[redacted]
1	19,105,143	673,442	588	0.15	32,491	1,145.31	[redacted]
2	13,755,351	1,661,800	407	0.403	33,796	4,083.049	[redacted]
3	23,312,878	7,753,866	494	0.619	47,192	15,696	[redacted]

#	% band purity	% lane purity	Rolling Bg. Corr. Vol.	Rolling Bg. Corr. Den.	Mol. Wt.	Rel. Quant. (w/ LB Corr. Vol.)
1	9.46	2.42	1,186,048	2,017.088	142.593	0.295
2	15.135	3.872	1,897,472	4,662.093	49.366	0.728
3	75.405	19.293	9,453,568	19,136	24.697	3.398

Lane 4 - 2uM Thapsigargin

#	Vol. (Int.)	Local Bg. Corr. Vol.	Area	Rf	Density	Local Bg. Corr. Den.	[redacted]
1	13,622,846	1,050,943	396	0.16	34,401	2,653.897	[redacted]
2	15,559,401	579,039	468	0.206	33,246	1,237.263	[redacted]
3	24,443,667	3,563,598	697	0.413	35,069	5,112.767	[redacted]
4	28,382,069	10,440,649	574	0.627	49,446	18,189	[redacted]

#	% band purity	% lane purity	Rolling Bg. Corr. Vol.	Rolling Bg. Corr. Den.	Mol. Wt.	Rel. Quant. (w/ LB Corr. Vol.)
1	8.875	2.846	1,565,696	3,953.778	135.185	0.461
2	4.78	1.533	843,264	1,801.846	100	0.254
3	21.259	6.818	3,750,656	5,381.142	48.098	1.562
4	65.086	20.873	11,482,880	20,005	24.242	4.575

Lane 5 - t = 0

#	Vol. (Int.)	Local Bg. Corr. Vol.	Area	Rf	Density	Local Bg. Corr. Den.	[redacted]
1	14,051,053	1,006,579	546	0.155	25,734	1,843.552	[redacted]
2	18,363,993	1,178,950	646	0.204	28,427	1,825.001	[redacted]
3	25,532,718	2,014,008	880	0.418	29,014	2,288.646	[redacted]
4	22,073,623	7,572,050	516	0.636	42,778	14,674	[redacted]

#	% band purity	% lane purity	Rolling Bg. Corr. Vol.	Rolling Bg. Corr. Den.	Mol. Wt.	Rel. Quant. (w/ LB Corr. Vol.)
1	15.674	3.055	1,036,288	1,897.963	138.889	0.441
2	23.167	4.515	1,531,648	2,370.972	101.852	0.517
3	35.65	6.948	2,356,992	2,678.4	47.463	0.883
4	25.509	4.971	1,686,528	3,268.465	23.636	3.318

iBright™ Image Analysis Report

Katarina+ Chang

19 November 2022

pMYPT1 CHEMI_01222022_145324

Date:	22 January 2022 02:53:24PM
Mode:	Chemi Blots
Notes:	
Model:	FL1500
Instrument name:	2462619090234
Serial No:	2462619090234
Firmware version:	1.6.0
iBA version:	5.0
Image size:	615px X 491px
Image area:	112.7mm X 90.16mm
Optical Zoom:	2x
Digital Zoom:	1.1x
Focus level:	455
Resolution:	5 x 5
Exposure time:	9000 ms
Exposure mode:	Normal

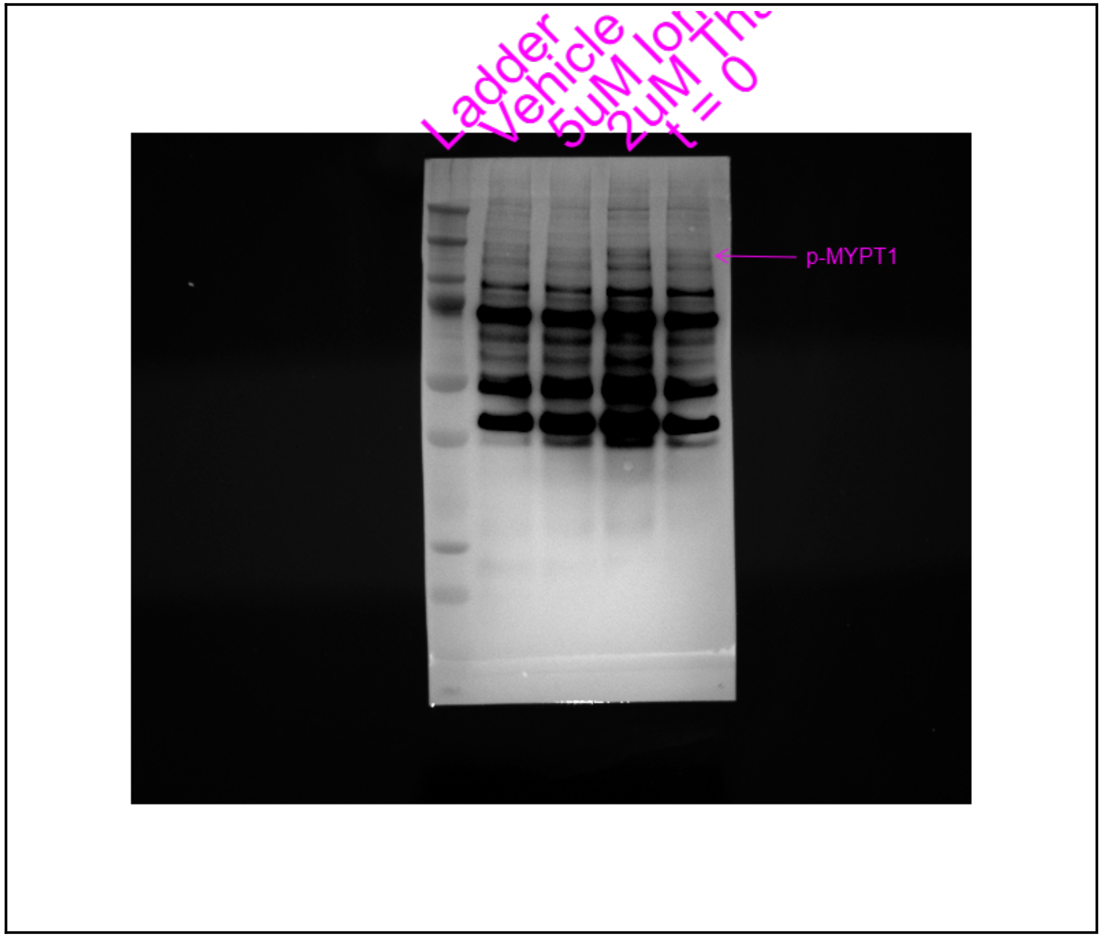

pMYPT1 CHEMI_01222022_145324

Date:	22 January 2022 02:53:24PM
Mode:	Chemi Blots
Notes:	
Model:	FL1500
Instrument name:	2462619090234
Serial No:	2462619090234
Firmware version:	1.6.0
iBA version:	5.0
Image size:	615px X 491px
Image area:	112.7mm X 90.16mm
Optical Zoom:	2x
Digital Zoom:	1.1x
Focus level:	455
Resolution:	5 x 5
Exposure time:	9000 ms
Exposure mode:	Normal

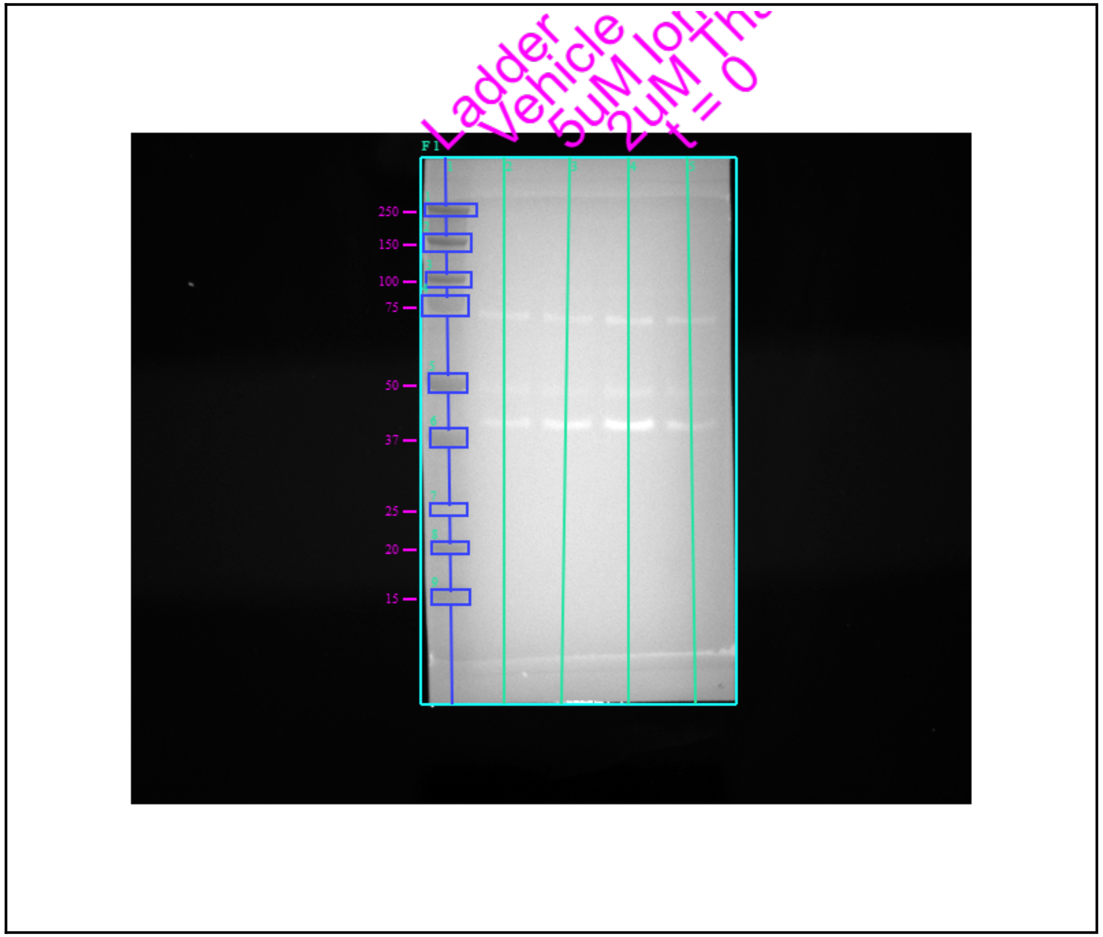

pMYPT1 CHEMI_01222022_145324

Date:	22 January 2022 02:53:24PM
Mode:	Chemi Blots
Notes:	
Model:	FL1500
Instrument name:	2462619090234
Serial No:	2462619090234
Firmware version:	1.6.0
iBA version:	5.0
Image size:	615px X 491px
Image area:	112.7mm X 90.16mm
Optical Zoom:	2x
Digital Zoom:	1.1x
Focus level:	455
Resolution:	5 x 5
Exposure time:	9000 ms
Exposure mode:	Normal

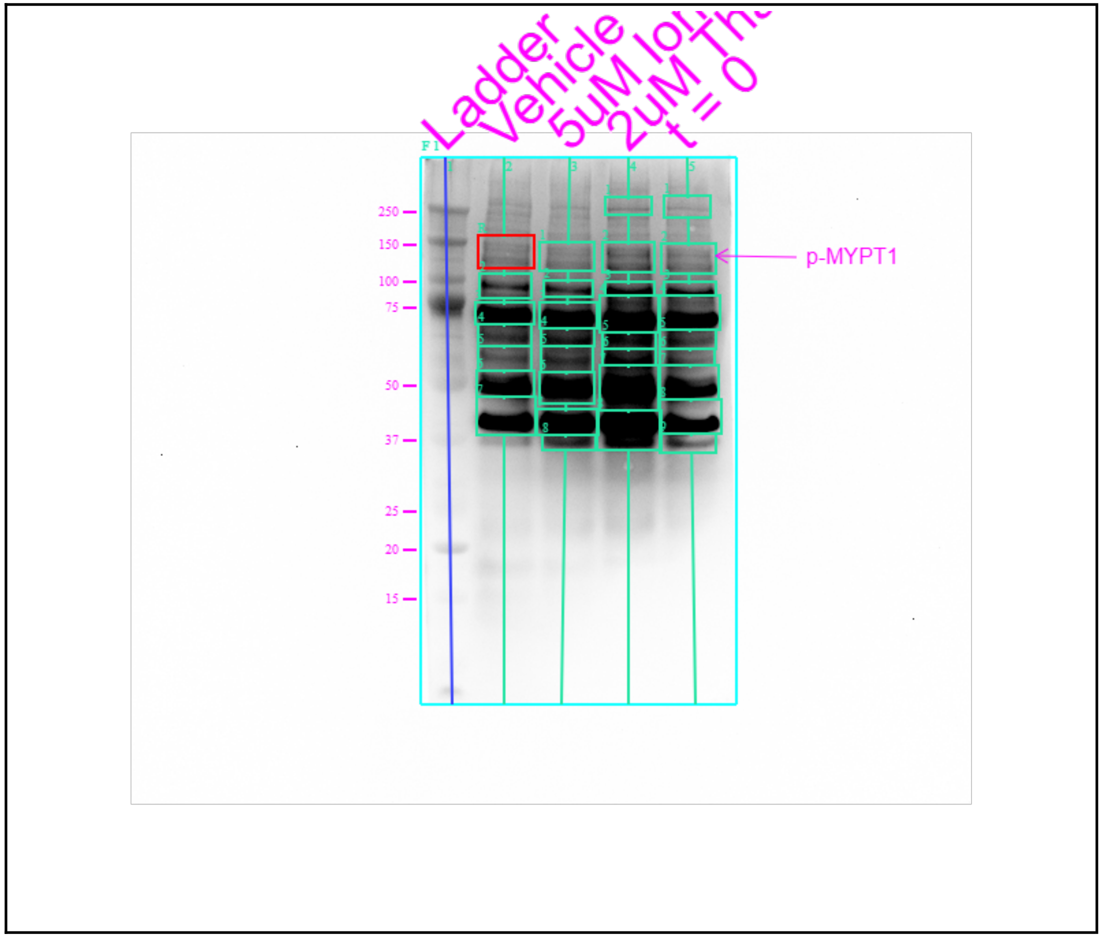

LANE AND BAND ANALYSIS DATA TABLE

pMYPT1 CHEMI_01222022_145324

Frame: 1
Channel: Membrane
Sensitivity: 100
Molecular Weight Analysis Regression Method : Point to Point

Lane 1 - Ladder

#	Vol. (Int.)	Local Bg. Corr. Vol.	Area	Rf	Density	Local Bg. Corr. Den.	% band purity	% lane purity	Rolling Bg. Corr. Vol.	Rolling Bg. Corr. Den.	Mol. Wt.
1	14,327,863	818,455	390	0.095	36,738	2,098.603	15.982	6.674	1,940,480	4,975.59	250
2	17,565,908	513,752	504	0.155	34,852	1,019.349	11.482	4.795	1,394,176	2,766.222	150
3	13,818,445	874,447	408	0.222	33,868	2,143.254	11.331	4.732	1,375,744	3,371.922	100
4	17,389,796	14,355	560	0.27	31,053	25.635	7.666	3.202	930,816	1,662.171	75
5	14,002,133	2,232,927	435	0.412	32,188	5,133.166	18.145	7.578	2,203,136	5,064.68	50
6	13,011,631	1,913,591	420	0.512	30,980	4,556.17	15.28	6.381	1,855,232	4,417.219	37
7	7,698,881	143,700	280	0.642	27,496	513.217	1.727	0.721	209,664	748.8	25
8	8,664,438	979,441	280	0.712	30,944	3,498.007	8.99	3.755	1,091,584	3,898.514	20
9	10,838,488	703,496	348	0.803	31,145	2,021.541	9.397	3.925	1,140,992	3,278.713	15

Frame: 1
Channel: Chemi
Sensitivity: 100
Molecular Weight Analysis Regression Method : Point to Point

Lane 2 - Vehicle

#	Vol. (Int.)	Local Bg. Corr. Vol.	Area	Rf	Density	Local Bg. Corr. Den.
1	32,104,964	6,123,054	1,050	0.172	30,576	5,831.48
2	32,072,436	7,815,733	741	0.235	43,282	10,547
3	43,703,921	14,684,989	748	0.285	58,427	19,632
4	33,320,780	4,856,151	680	0.325	49,001	7,141.399
5	31,552,188	3,201,405	760	0.368	41,516	4,212.375
6	47,338,117	19,087,138	860	0.412	55,044	22,194
7	59,871,123	17,493,526	1,276	0.472	46,920	13,709

#	% band purity	% lane purity	Rolling Bg. Corr. Vol.	Rolling Bg. Corr. Den.	Mol. Wt.	Rel. Quant. (w/ LB Corr. Vol.)

#	% band purity	% lane purity	Rolling Bg. Corr. Vol.	Rolling Bg. Corr. Den.	Mol. Wt.	Rel. Quant. (w/ LB Corr. Vol.)
1	4.623	6.089	3,884,800	3,699.81	137.037	1
2	11.111	14.634	9,337,088	12,600	93.421	1.276
3	22.499	29.634	18,907,648	25,277	72.368	2.398
4	11.333	14.926	9,523,712	14,005	65.351	0.793
5	4.609	6.07	3,873,024	5,096.084	57.895	0.523
6	19.573	25.779	16,448,512	19,126	50	3.117
7	26.253	34.577	22,062,080	17,290	42.2	2.857

Lane 3 - 5uM Ionomycin

#	Vol. (Int.)	Local Bg. Corr. Vol.	Area	Rf	Density	Local Bg. Corr. Den.	[redacted]
1	28,360,003	4,651,424	902	0.18	31,441	5,156.789	[redacted]
2	22,358,455	5,223,537	481	0.24	46,483	10,859	[redacted]
3	49,415,806	12,316,669	860	0.287	57,460	14,321	[redacted]
4	30,751,851	4,664,683	560	0.327	54,914	8,329.792	[redacted]
5	38,955,557	3,791,164	780	0.368	49,943	4,860.467	[redacted]
6	57,272,774	16,391,990	984	0.42	58,204	16,658	[redacted]
7	54,630,714	12,790,448	920	0.482	59,381	13,902	[redacted]
8	22,128,950	4,375,459	480	0.52	46,101	9,115.54	[redacted]

#	% band purity	% lane purity	Rolling Bg. Corr. Vol.	Rolling Bg. Corr. Den.	Mol. Wt.	Rel. Quant. (w/ LB Corr. Vol.)
1	4.386	5.575	3,013,376	3,340.772	131.481	0.76
2	8.576	10.9	5,891,328	12,248	90.789	0.853
3	23.702	30.126	16,283,392	18,934	71.93	2.012
4	10.584	13.453	7,271,168	12,984	64.912	0.762
5	5.62	7.143	3,860,736	4,949.662	57.895	0.619
6	17.048	21.668	11,711,744	11,902	49.025	2.677
7	24.562	31.219	16,873,981	18,341	40.9	2.089
8	5.522	7.019	3,793,664	7,903.467	36.308	0.715

Lane 4 - 2uM Thapsigargin

#	Vol. (Int.)	Local Bg. Corr. Vol.	Area	Rf	Density	Local Bg. Corr. Den.	[redacted]
1	13,038,794	2,063,100	490	0.087	26,609	4,210.41	[redacted]
2	33,230,289	8,110,725	897	0.183	37,046	9,042.057	[redacted]

#	Vol. (Int.)	Local Bg. Corr. Vol.	Area	Rf	Density	Local Bg. Corr. Den.
3	21,827,588	5,941,305	407	0.24	53,630	14,597
4	71,396,085	13,300,543	1,232	0.285	57,951	10,795
5	30,268,111	4,752,066	520	0.335	58,207	9,138.59
6	30,338,325	5,896,836	520	0.365	58,342	11,340
7	89,812,372	16,673,302	1,496	0.42	60,035	11,145
8	84,028,719	24,674,598	1,440	0.498	58,353	17,135

#	% band purity	% lane purity	Rolling Bg. Corr. Vol.	Rolling Bg. Corr. Den.	Mol. Wt.	Rel. Quant. (w/ LB Corr. Vol.)
1	4.211	5.158	2,356,736	4,809.665	NA	0.337
2	9.111	11.159	5,098,752	5,684.227	129.63	1.325
3	10.502	12.862	5,877,248	14,440	90.789	0.97
4	30.09	36.852	16,838,912	13,667	72.368	2.172
5	7.474	9.153	4,182,272	8,042.831	63.596	0.776
6	5.323	6.519	2,978,816	5,728.492	58.333	0.963
7	15.322	18.765	8,574,208	5,731.422	49.025	2.723
8	17.966	22.004	10,054,144	6,982.044	38.95	4.03

Lane 5 - t = 0

#	Vol. (Int.)	Local Bg. Corr. Vol.	Area	Rf	Density	Local Bg. Corr. Den.
1	13,681,168	2,112,469	595	0.09	22,993	3,550.368
2	29,045,301	6,450,950	943	0.185	30,800	6,840.881
3	20,575,505	6,485,248	429	0.24	47,961	15,117
4	61,108,820	16,423,963	1,170	0.282	52,229	14,037
5	29,407,743	4,062,189	630	0.333	46,678	6,447.92
6	22,031,917	3,404,387	533	0.365	41,335	6,387.218
7	56,388,334	19,514,688	1,144	0.41	49,290	17,058
8	54,544,335	15,417,038	1,196	0.472	45,605	12,890
9	20,345,897	2,759,575	630	0.522	32,295	4,380.279

#	% band purity	% lane purity	Rolling Bg. Corr. Vol.	Rolling Bg. Corr. Den.	Mol. Wt.	Rel. Quant. (w/ LB Corr. Vol.)
1	1.991	2.786	1,978,880	3,325.849	NA	0.345
2	3.936	5.508	3,912,704	4,149.209	127.778	1.054
3	7.77	10.872	7,723,008	18,002	90.789	1.059

#	% band purity	% lane purity	Rolling Bg. Corr. Vol.	Rolling Bg. Corr. Den.	Mol. Wt.	Rel. Quant. (w/ LB Corr. Vol.)
4	24.528	34.321	24,380,672	20,838	72.807	2.682
5	8.291	11.601	8,241,152	13,081	64.035	0.663
6	3.724	5.21	3,701,248	6,944.18	58.333	0.556
7	18.47	25.844	18,358,784	16,047	50.439	3.187
8	23.686	33.143	23,543,808	19,685	42.2	2.518
9	7.606	10.642	7,559,936	11,999	36.077	0.451

iBright™ Image Analysis Report

Katarina+ Chang

19 November 2022

total MLC CHEMI_01242022_132647

Date:	24 January 2022 01:26:47PM
Mode:	Chemi Blots
Notes:	
Model:	FL1500
Instrument name:	2462619090234
Serial No:	2462619090234
Firmware version:	1.6.0
iBA version:	5.0
Image size:	676px X 540px
Image area:	118.63mm X 94.91mm
Optical Zoom:	1.9x
Digital Zoom:	1x
Focus level:	430
Resolution:	5 x 5
Exposure time:	5400 ms
Exposure mode:	Normal

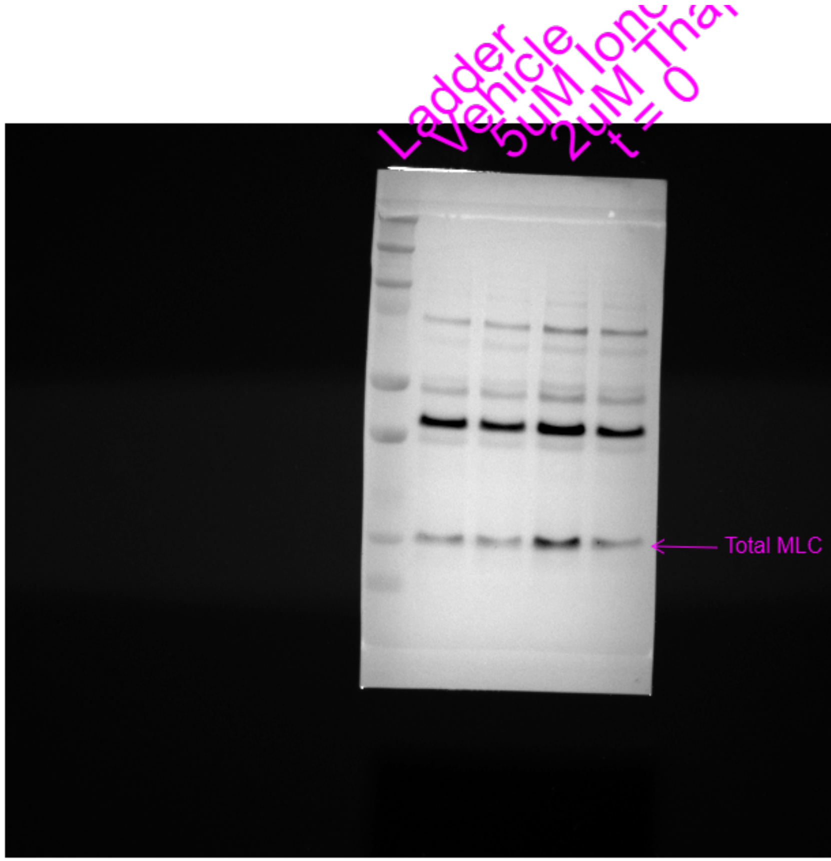

total MLC CHEMI_01242022_132647

Date:	24 January 2022 01:26:47PM
Mode:	Chemi Blots
Notes:	
Model:	FL1500
Instrument name:	2462619090234
Serial No:	2462619090234
Firmware version:	1.6.0
iBA version:	5.0
Image size:	676px X 540px
Image area:	118.63mm X 94.91mm
Optical Zoom:	1.9x
Digital Zoom:	1x
Focus level:	430
Resolution:	5 x 5
Exposure time:	5400 ms
Exposure mode:	Normal

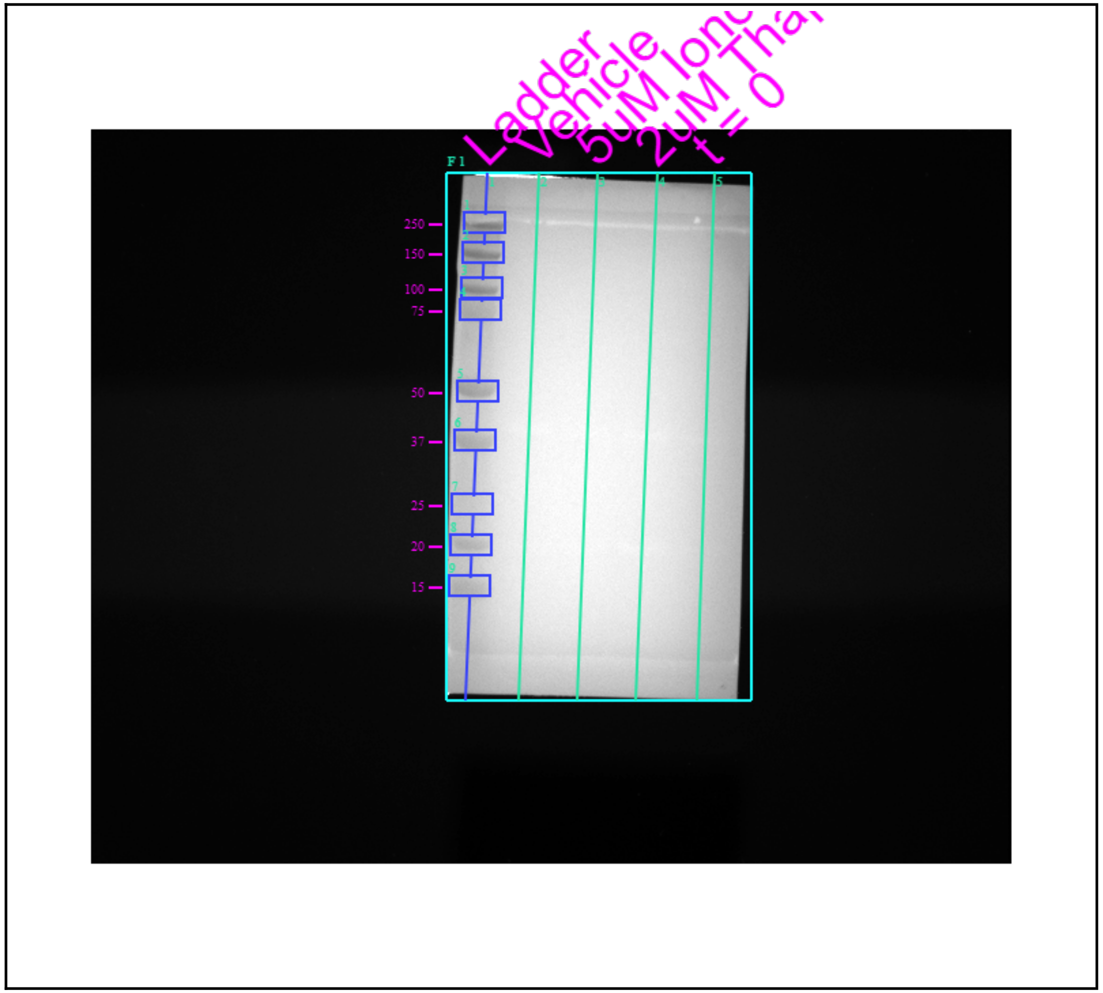

total MLC CHEMI_01242022_132647

Date:	24 January 2022 01:26:47PM
Mode:	Chemi Blots
Notes:	
Model:	FL1500
Instrument name:	2462619090234
Serial No:	2462619090234
Firmware version:	1.6.0
iBA version:	5.0
Image size:	676px X 540px
Image area:	118.63mm X 94.91mm
Optical Zoom:	1.9x
Digital Zoom:	1x
Focus level:	430
Resolution:	5 x 5
Exposure time:	5400 ms
Exposure mode:	Normal

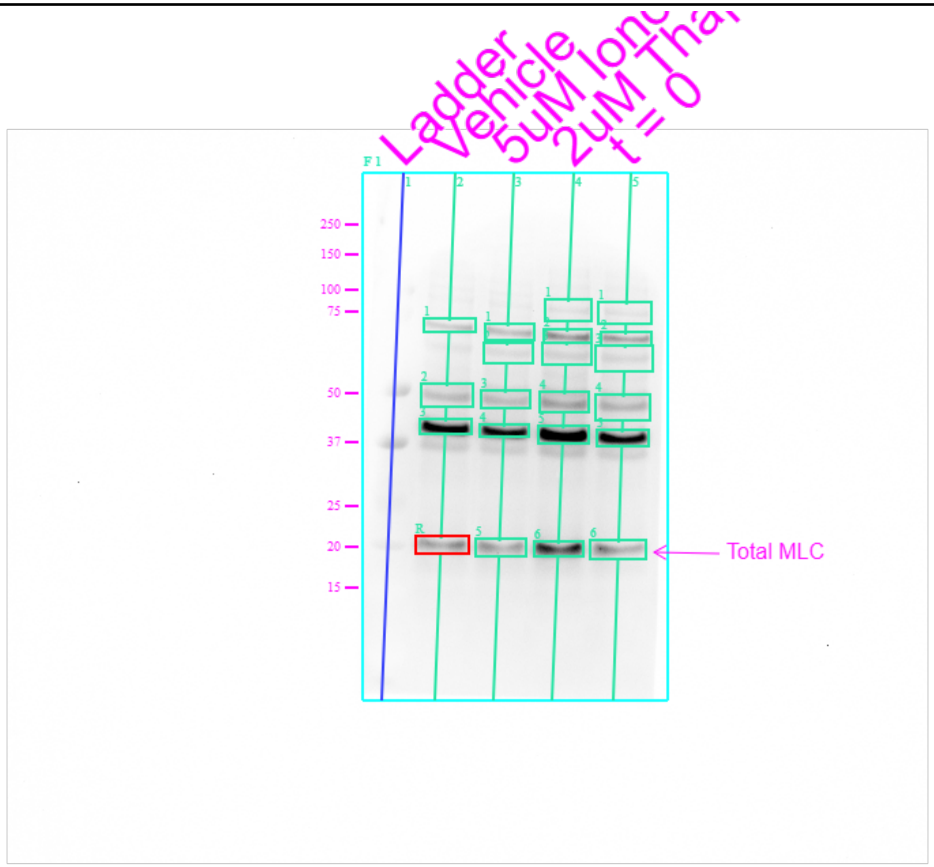

LANE AND BAND ANALYSIS DATA TABLE

total MLC CHEMI_01242022_132647

Frame: 1
Channel: Membrane
Sensitivity: 100
Molecular Weight Analysis Regression Method : Point to Point

Lane 1 - Ladder

#	Vol. (Int.)	Local Bg. Corr. Vol.	Area	Rf	Density	Local Bg. Corr. Den.	% band purity	% lane purity	Mol. Wt.
1	17,197,622	641,371	496	0.093	34,672	1,293.088	10.477	4.27	250
2	17,194,721	1,229,812	496	0.149	34,666	2,479.46	20.089	4.269	150
3	16,125,171	990,920	496	0.216	32,510	1,997.823	16.186	4.003	100
4	15,059,098	274,577	496	0.258	30,361	553.585	4.485	3.739	75
5	14,776,474	1,772,507	496	0.412	29,791	3,573.604	28.953	3.669	50
6	14,348,815	1,119,294	496	0.505	28,929	2,256.642	18.283	3.562	37
7	12,926,538	NA	496	0.626	26,061	NA	NA	3.209	25
8	13,870,454	70,188	496	0.704	27,964	141.51	1.147	3.444	20
9	14,366,855	23,274	496	0.781	28,965	46.925	0.38	3.567	15

Frame: 1
Channel: Chemi
Sensitivity: 100
Molecular Weight Analysis Regression Method : Point to Point

Lane 2 - Vehicle

#	Vol. (Int.)	Local Bg. Corr. Vol.	Area	Rf	Density	Local Bg. Corr. Den.	% band purity	% lane purity	Mol. Wt.	Rel. Quant. (w/ LB Corr. Vol.)
1	3,190,970	1,695,711	429	0.289	7,438.159	3,952.708	7.578	4.008	70	0.389
2	7,429,158	2,963,543	702	0.42	10,582	4,221.572	13.244	9.33	48.917	0.679
3	17,990,089	13,355,325	468	0.479	38,440	28,537	59.686	22.594	40.611	3.062
4	7,897,313	4,361,485	560	0.704	14,102	7,788.366	19.492	9.918	20	1

Lane 3 - 5uM Ionomycin

#	Vol. (Int.)	Local Bg. Corr. Vol.	Area	Rf	Density	Local Bg. Corr. Den.	% band purity	% lane purity	Mol. Wt.	Rel. Quant. (w/ LB Corr. Vol.)
1	4,451,489	2,017,571	481	0.302	9,254.655	4,194.535	10.716	5.21	67.917	0.463

#	Vol. (Int.)	Local Bg. Corr. Vol.	Area	Rf	Density	Local Bg. Corr. Den.	% band purity	% lane purity	Mol. Wt.	Rel. Quant. (w/ LB Corr. Vol.)
2	4,135,322	708,538	592	0.34	6,985.341	1,196.855	3.763	4.84	61.667	0.162
3	6,410,649	2,314,367	518	0.428	12,375	4,467.89	12.292	7.503	47.833	0.531
4	14,395,314	10,133,559	370	0.487	38,906	27,387	53.823	16.848	39.528	2.323
5	7,532,908	3,653,539	532	0.709	14,159	6,867.555	19.405	8.816	19.667	0.838

Lane 4 - 2uM Thapsigargin

#	Vol. (Int.)	Local Bg. Corr. Vol.	Area	Rf	Density	Local Bg. Corr. Den.	% band purity	% lane purity	Mol. Wt.	Rel. Quant. (w/ LB Corr. Vol.)
1	3,589,741	700,106	595	0.26	6,033.178	1,176.65	2.033	3.182	74.583	0.161
2	6,157,665	3,601,962	396	0.309	15,549	9,095.864	10.46	5.458	66.667	0.826
3	5,526,459	186,488	629	0.343	8,786.103	296.485	0.542	4.899	61.25	0.043
4	9,783,279	4,355,385	592	0.433	16,525	7,357.07	12.649	8.672	47.111	0.999
5	22,367,909	15,954,968	532	0.495	42,044	29,990	46.335	19.827	38.444	3.658
6	13,833,038	9,635,037	444	0.711	31,155	21,700	27.981	12.261	19.5	2.209

Lane 5 - t = 0

#	Vol. (Int.)	Local Bg. Corr. Vol.	Area	Rf	Density	Local Bg. Corr. Den.	% band purity	% lane purity	Mol. Wt.	Rel. Quant. (w/ LB Corr. Vol.)
1	3,682,552	778,983	680	0.265	5,415.518	1,145.564	3.101	4.285	73.75	0.179
2	5,307,710	3,066,639	418	0.314	12,697	7,336.458	12.207	6.175	65.833	0.703
3	5,913,216	321,960	860	0.351	6,875.833	374.373	1.282	6.88	60	0.074
4	9,313,439	3,440,493	820	0.443	11,357	4,195.724	13.696	10.836	45.667	0.789
5	17,517,179	12,885,924	507	0.503	34,550	25,416	51.295	20.381	37.361	2.954
6	8,413,646	4,627,086	630	0.714	13,354	7,344.582	18.419	9.789	19.333	1.061

iBright™ Image Analysis Report

Katarina+ Chang

19 November 2022

GAPDH CHEMI_01252022_112132

Date:	25 January 2022 11:21:32AM
Mode:	Chemi Blots
Notes:	
Model:	FL1500
Instrument name:	2462619090234
Serial No:	2462619090234
Firmware version:	1.6.0
iBA version:	5.0
Image size:	676px X 540px
Image area:	118.63mm X 94.91mm
Optical Zoom:	1.9x
Digital Zoom:	1x
Focus level:	430
Resolution:	5 x 5
Exposure time:	11511 ms
Exposure mode:	Normal

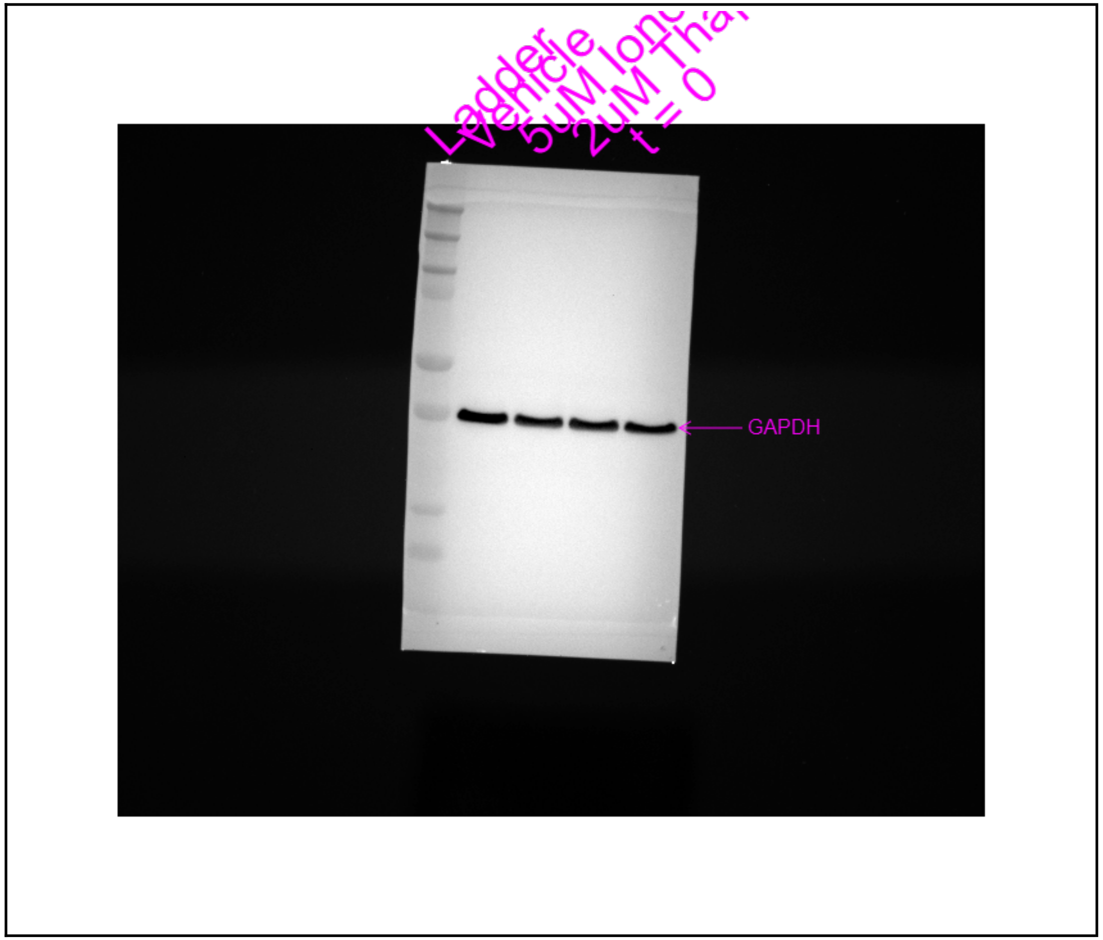

GAPDH CHEMI_01252022_112132

Date:	25 January 2022 11:21:32AM
Mode:	Chemi Blots
Notes:	
Model:	FL1500
Instrument name:	2462619090234
Serial No:	2462619090234
Firmware version:	1.6.0
iBA version:	5.0
Image size:	676px X 540px
Image area:	118.63mm X 94.91mm
Optical Zoom:	1.9x
Digital Zoom:	1x
Focus level:	430
Resolution:	5 x 5
Exposure time:	11511 ms
Exposure mode:	Normal

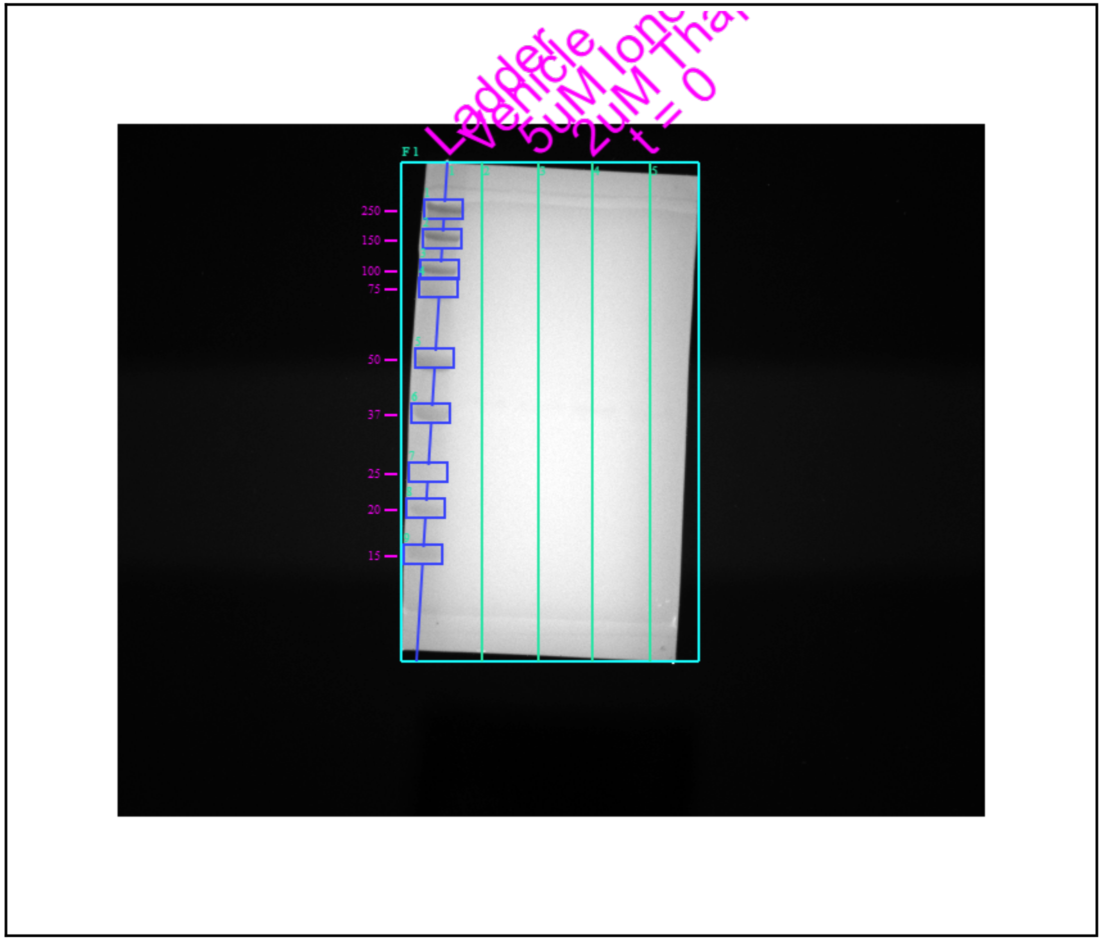

GAPDH CHEMI_01252022_112132

Date:	25 January 2022 11:21:32AM
Mode:	Chemi Blots
Notes:	
Model:	FL1500
Instrument name:	2462619090234
Serial No:	2462619090234
Firmware version:	1.6.0
iBA version:	5.0
Image size:	676px X 540px
Image area:	118.63mm X 94.91mm
Optical Zoom:	1.9x
Digital Zoom:	1x
Focus level:	430
Resolution:	5 x 5
Exposure time:	11511 ms
Exposure mode:	Normal

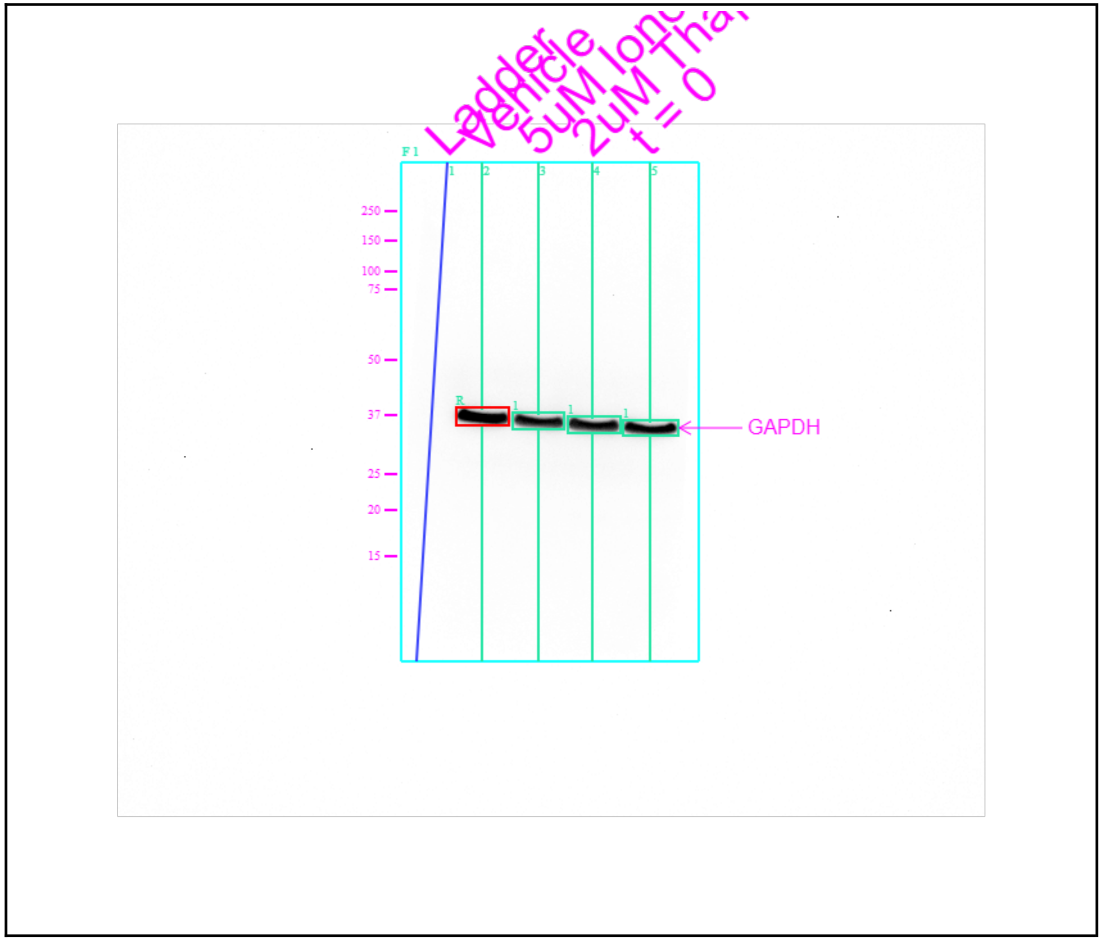

LANE AND BAND ANALYSIS DATA TABLE

GAPDH CHEMI_01252022_112132

Frame: 1
Channel: Membrane
Sensitivity: 100
Molecular Weight Analysis Regression Method : Point to Point

Lane 1 - Ladder

#	Vol. (Int.)	Local Bg. Corr. Vol.	Area	Rf	Density	Local Bg. Corr. Den.	% band purity	% lane purity	Mol. Wt.
1	18,123,694	630,639	496	0.093	36,539	1,271.45	22.362	3.647	250
2	17,395,096	780,124	496	0.152	35,070	1,572.831	27.663	3.5	150
3	16,667,062	437,409	496	0.213	33,602	881.874	15.51	3.354	100
4	15,639,794	434.059	496	0.249	31,531	0.875	0.015	3.147	75
5	15,437,768	663,261	496	0.391	31,124	1,337.221	23.519	3.106	50
6	15,011,992	292,020	496	0.501	30,266	588.75	10.355	3.021	37
7	13,600,834	NA	496	0.62	27,421	NA	NA	2.737	25
8	14,548,319	161.23	496	0.692	29,331	0.325	0.006	2.927	20
9	15,213,230	16,043	496	0.784	30,671	32.345	0.569	3.061	15

Frame: 1
Channel: Chemi
Sensitivity: 100
Molecular Weight Analysis Regression Method : Point to Point

Lane 2 - Vehicle

#	Vol. (Int.)	Local Bg. Corr. Vol.	Area	Rf	Density	Local Bg. Corr. Den.	% band purity	% lane purity	Mol. Wt.	Rel. Quant. (w/ LB Corr. Vol.)
1	16,355,798	14,319,854	630	0.509	25,961	22,729	100	70.82	36.217	1

Lane 3 - 5uM Ionomycin

#	Vol. (Int.)	Local Bg. Corr. Vol.	Area	Rf	Density	Local Bg. Corr. Den.	% band purity	% lane purity	Mol. Wt.	Rel. Quant. (w/ LB Corr. Vol.)
1	11,304,322	8,966,972	574	0.517	19,693	15,621	100	60.126	35.435	0.626

Lane 4 - 2uM Thapsigargin

#	Vol. (Int.)	Local Bg. Corr. Vol.	Area	Rf	Density	Local Bg. Corr. Den.	% band purity	% lane purity	Mol. Wt.	Rel. Quant. (w/ LB Corr. Vol.)

#	Vol. (Int.)	Local Bg. Corr. Vol.	Area	Rf	Density	Local Bg. Corr. Den.	% band purity	% lane purity	Mol. Wt.	Rel. Quant. (w/ LB Corr. Vol.)
1	11,769,926	9,206,991	588	0.524	20,016	15,658	100	60.679	34.652	0.643

Lane 5 - t = 0

#	Vol. (Int.)	Local Bg. Corr. Vol.	Area	Rf	Density	Local Bg. Corr. Den.	% band purity	% lane purity	Mol. Wt.	Rel. Quant. (w/ LB Corr. Vol.)
1	12,233,663	10,701,089	572	0.532	21,387	18,708	100	68.139	33.87	0.747

iBright™ Image Analysis Report

Katarina+ Chang

19 November 2022

GAPDH CHEMI_01252022_112516

Date:	25 January 2022 11:25:16AM
Mode:	Chemi Blots
Notes:	
Model:	FL1500
Instrument name:	2462619090234
Serial No:	2462619090234
Firmware version:	1.6.0
iBA version:	5.0
Image size:	676px X 540px
Image area:	118.63mm X 94.91mm
Optical Zoom:	1.9x
Digital Zoom:	1x
Focus level:	430
Resolution:	5 x 5
Exposure time:	7979 ms
Exposure mode:	Normal

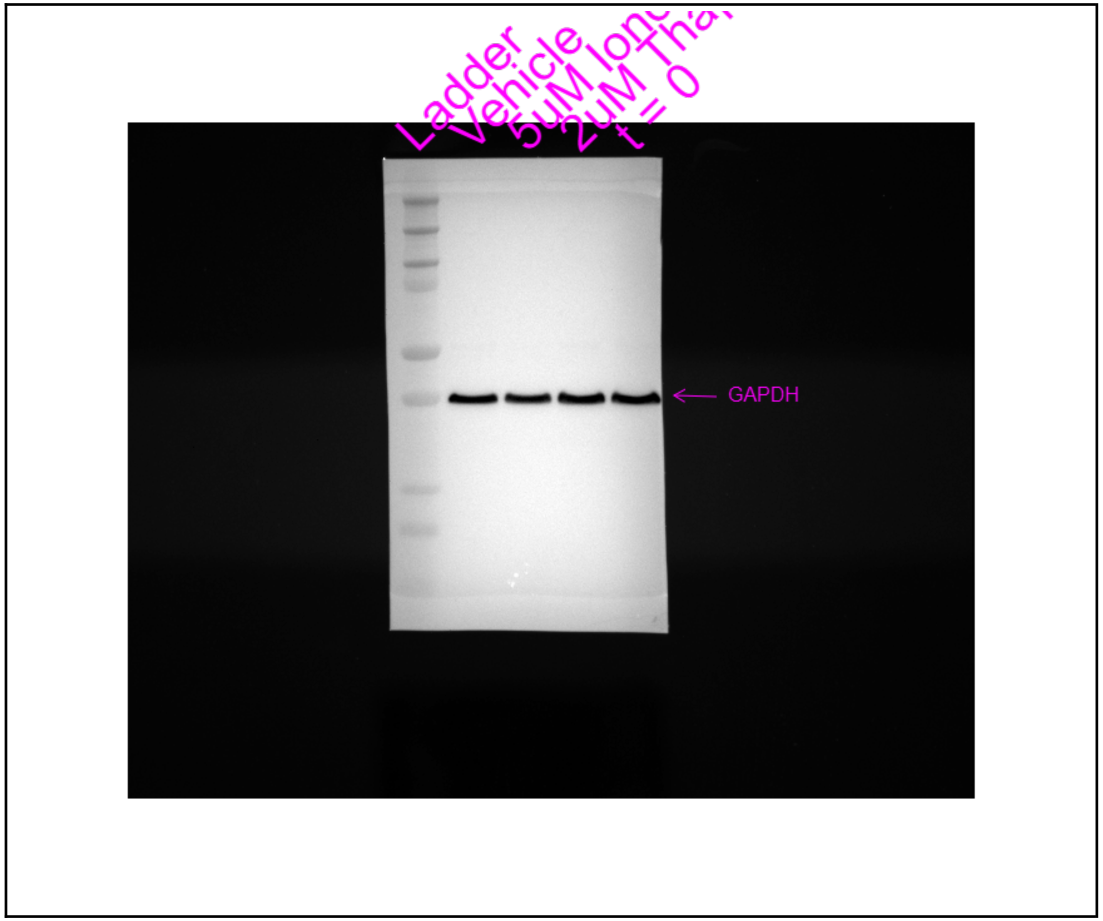

GAPDH CHEMI_01252022_112516

Date:	25 January 2022 11:25:16AM
Mode:	Chemi Blots
Notes:	
Model:	FL1500
Instrument name:	2462619090234
Serial No:	2462619090234
Firmware version:	1.6.0
iBA version:	5.0
Image size:	676px X 540px
Image area:	118.63mm X 94.91mm
Optical Zoom:	1.9x
Digital Zoom:	1x
Focus level:	430
Resolution:	5 x 5
Exposure time:	7979 ms
Exposure mode:	Normal

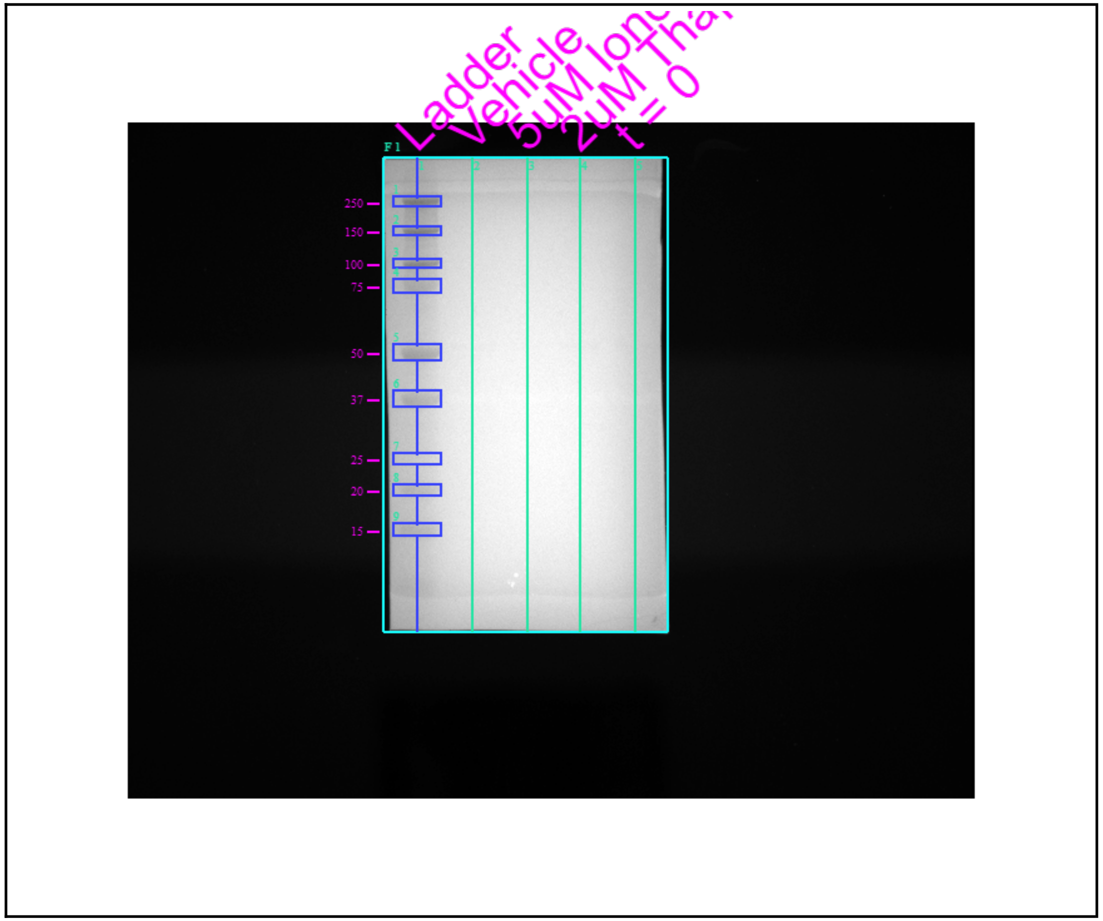

GAPDH CHEMI_01252022_112516

Date:	25 January 2022 11:25:16AM
Mode:	Chemi Blots
Notes:	
Model:	FL1500
Instrument name:	2462619090234
Serial No:	2462619090234
Firmware version:	1.6.0
iBA version:	5.0
Image size:	676px X 540px
Image area:	118.63mm X 94.91mm
Optical Zoom:	1.9x
Digital Zoom:	1x
Focus level:	430
Resolution:	5 x 5
Exposure time:	7979 ms
Exposure mode:	Normal

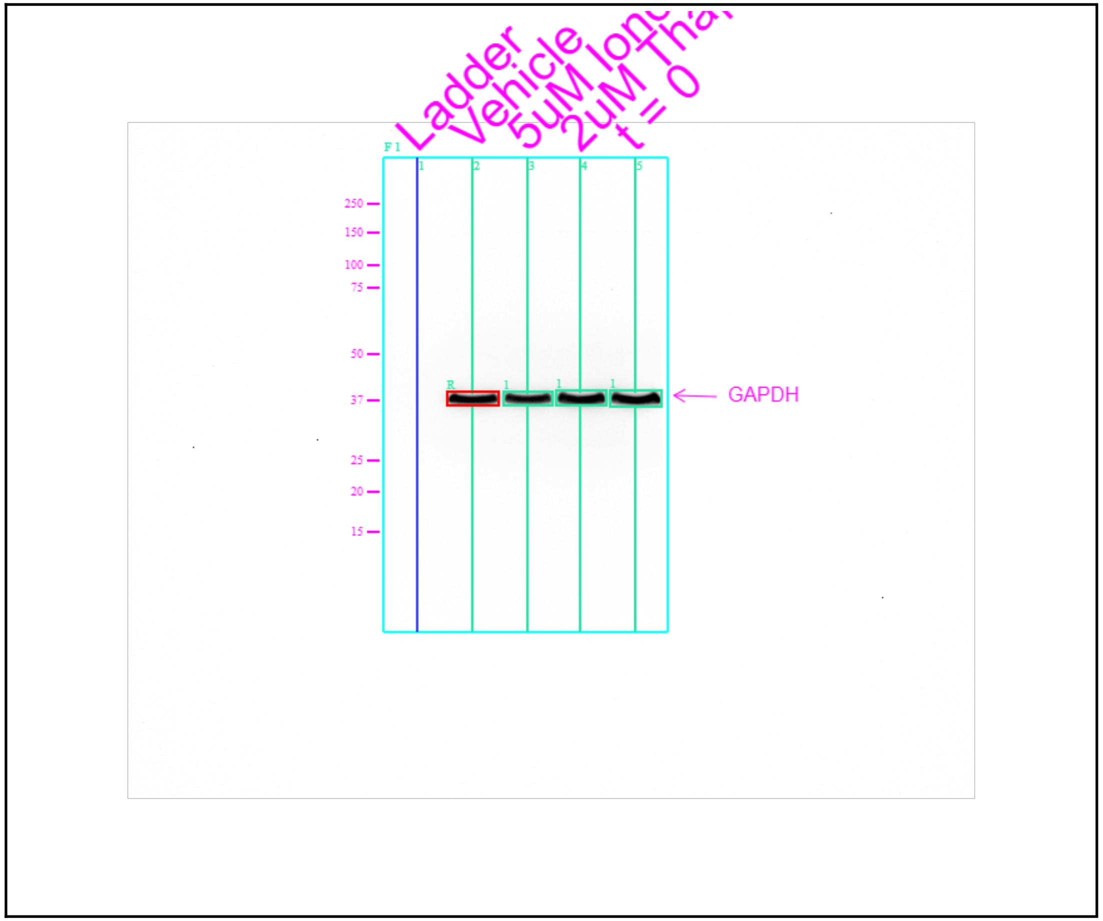

LANE AND BAND ANALYSIS DATA TABLE

GAPDH CHEMI_01252022_112516

Frame: 1
Channel: Membrane
Sensitivity: 100
Molecular Weight Analysis Regression Method : Point to Point

Lane 1 - Ladder

#	Vol. (Int.)	Local Bg. Corr. Vol.	Area	Rf	Density	Local Bg. Corr. Den.	% band purity	% lane purity	Rolling Bg. Corr. Vol.	Rolling Bg. Corr. Den.	Mol. Wt.
1	12,998,345	1,647,675	351	0.092	37,032	4,694.233	15.948	4.715	1,895,680	5,400.798	250
2	11,096,962	1,483,551	312	0.153	35,567	4,754.974	11.815	3.493	1,404,416	4,501.333	150
3	10,639,080	1,441,797	312	0.222	34,099	4,621.145	11.339	3.352	1,347,840	4,320	100
4	14,349,208	1,119,098	468	0.269	30,660	2,391.236	8.027	2.373	954,112	2,038.701	75
5	16,700,149	2,134,216	546	0.409	30,586	3,908.821	17.766	5.252	2,111,744	3,867.663	50
6	15,893,185	1,390,492	546	0.507	29,108	2,546.69	14.779	4.369	1,756,672	3,217.348	37
7	10,286,756	77,456	390	0.633	26,376	198.606	3.172	0.938	377,088	966.892	25
8	11,165,394	332,301	390	0.699	28,629	852.054	7.971	2.356	947,456	2,429.374	20
9	12,662,397	304,286	429	0.784	29,516	709.292	9.181	2.714	1,091,328	2,543.888	15

Frame: 1
Channel: Chemi
Sensitivity: 100
Molecular Weight Analysis Regression Method : Point to Point

Lane 2 - Vehicle

#	Vol. (Int.)	Local Bg. Corr. Vol.	Area	Rf	Density	Local Bg. Corr. Den.
1	14,435,627	12,566,260	504	0.507	28,642	24,933

#	% band purity	% lane purity	Rolling Bg. Corr. Vol.	Rolling Bg. Corr. Den.	Mol. Wt.	Rel. Quant. (w/ LB Corr. Vol.)
1	100	90.859	13,753,856	27,289	37	1

Lane 3 - 5uM Ionomycin

#	Vol. (Int.)	Local Bg. Corr. Vol.	Area	Rf	Density	Local Bg. Corr. Den.
1	12,701,366	10,416,652	480	0.507	26,461	21,701

#	% band purity	% lane purity	Rolling Bg. Corr. Vol.	Rolling Bg. Corr. Den.	Mol. Wt.	Rel. Quant. (w/ LB Corr. Vol.)

#	% band purity	% lane purity	Rolling Bg. Corr. Vol.	Rolling Bg. Corr. Den.	Mol. Wt.	Rel. Quant. (w/ LB Corr. Vol.)
1	100	87.539	11,850,240	24,688	37	0.829

Lane 4 - 2uM Thapsigargin

#	Vol. (Int.)	Local Bg. Corr. Vol.	Area	Rf	Density	Local Bg. Corr. Den.
1	15,380,958	12,454,503	533	0.507	28,857	23,366

#	% band purity	% lane purity	Rolling Bg. Corr. Vol.	Rolling Bg. Corr. Den.	Mol. Wt.	Rel. Quant. (w/ LB Corr. Vol.)
1	100	89.667	14,415,104	27,045	37	0.991

Lane 5 - t = 0

#	Vol. (Int.)	Local Bg. Corr. Vol.	Area	Rf	Density	Local Bg. Corr. Den.
1	16,699,299	14,781,248	588	0.507	28,400	25,138

#	% band purity	% lane purity	Rolling Bg. Corr. Vol.	Rolling Bg. Corr. Den.	Mol. Wt.	Rel. Quant. (w/ LB Corr. Vol.)
1	100	92.834	15,800,576	26,871	37	1.176

iBright™ Image Analysis Report

Katarina+ Chang

19 November 2022

pMLC CHEMI_01222022_143400

Date: 22 January 2022 02:34:00PM
Mode: Chemi Blots
Notes:
Model: FL1500
Instrument name: 2462619090234
Serial No: 2462619090234
Firmware version: 1.6.0
iBA version: 5.0
Image size: 563px X 450px
Image area: 112.7mm X 90.16mm
Optical Zoom: 2x
Digital Zoom: 1.2x
Focus level: 455
Resolution: 5 x 5
Exposure time: 60000 ms
Exposure mode: Normal

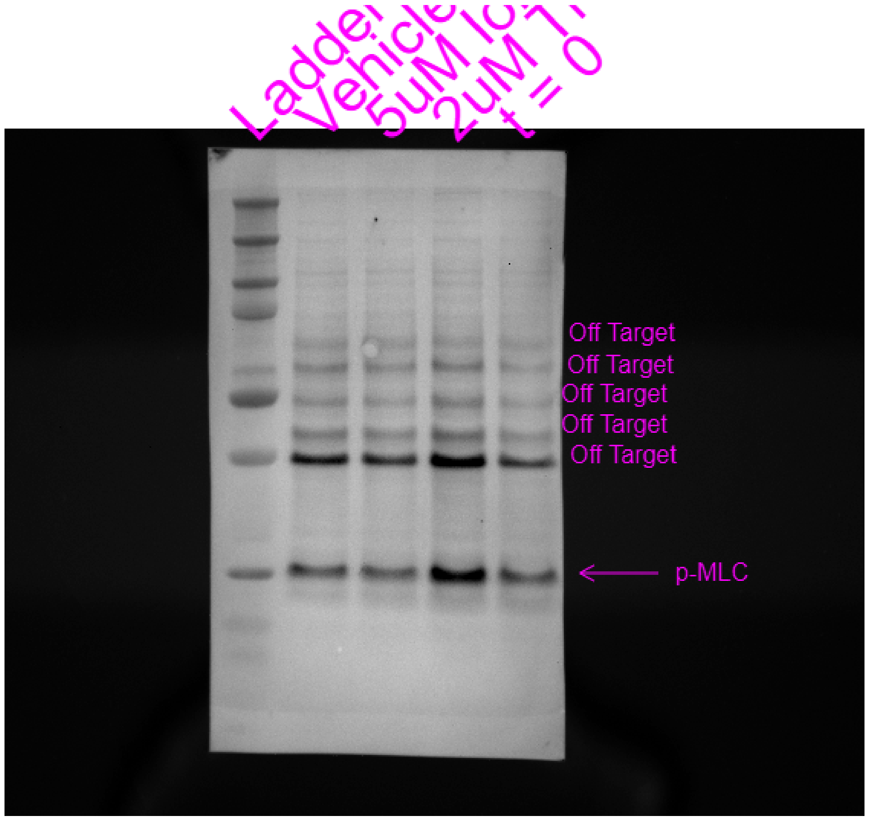

pMLC CHEMI_01222022_143400

Date:	22 January 2022 02:34:00PM
Mode:	Chemi Blots
Notes:	
Model:	FL1500
Instrument name:	2462619090234
Serial No:	2462619090234
Firmware version:	1.6.0
iBA version:	5.0
Image size:	563px X 450px
Image area:	112.7mm X 90.16mm
Optical Zoom:	2x
Digital Zoom:	1.2x
Focus level:	455
Resolution:	5 x 5
Exposure time:	60000 ms
Exposure mode:	Normal

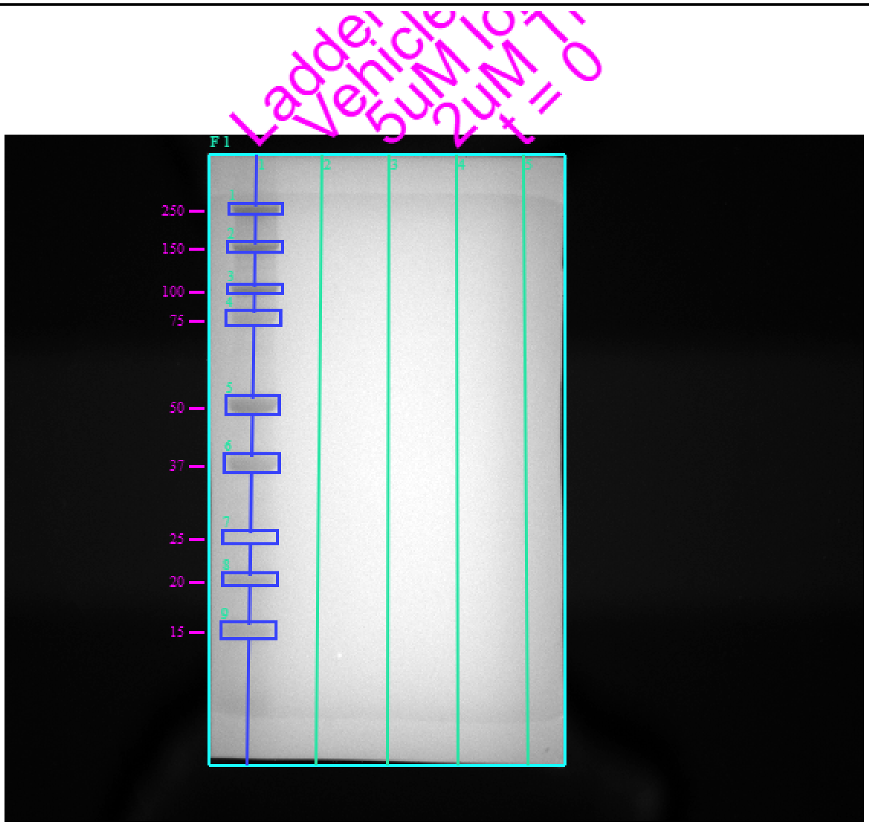

pMLC CHEMI_01222022_143400

Date:	22 January 2022 02:34:00PM
Mode:	Chemi Blots
Notes:	
Model:	FL1500
Instrument name:	2462619090234
Serial No:	2462619090234
Firmware version:	1.6.0
iBA version:	5.0
Image size:	563px X 450px
Image area:	112.7mm X 90.16mm
Optical Zoom:	2x
Digital Zoom:	1.2x
Focus level:	455
Resolution:	5 x 5
Exposure time:	60000 ms
Exposure mode:	Normal

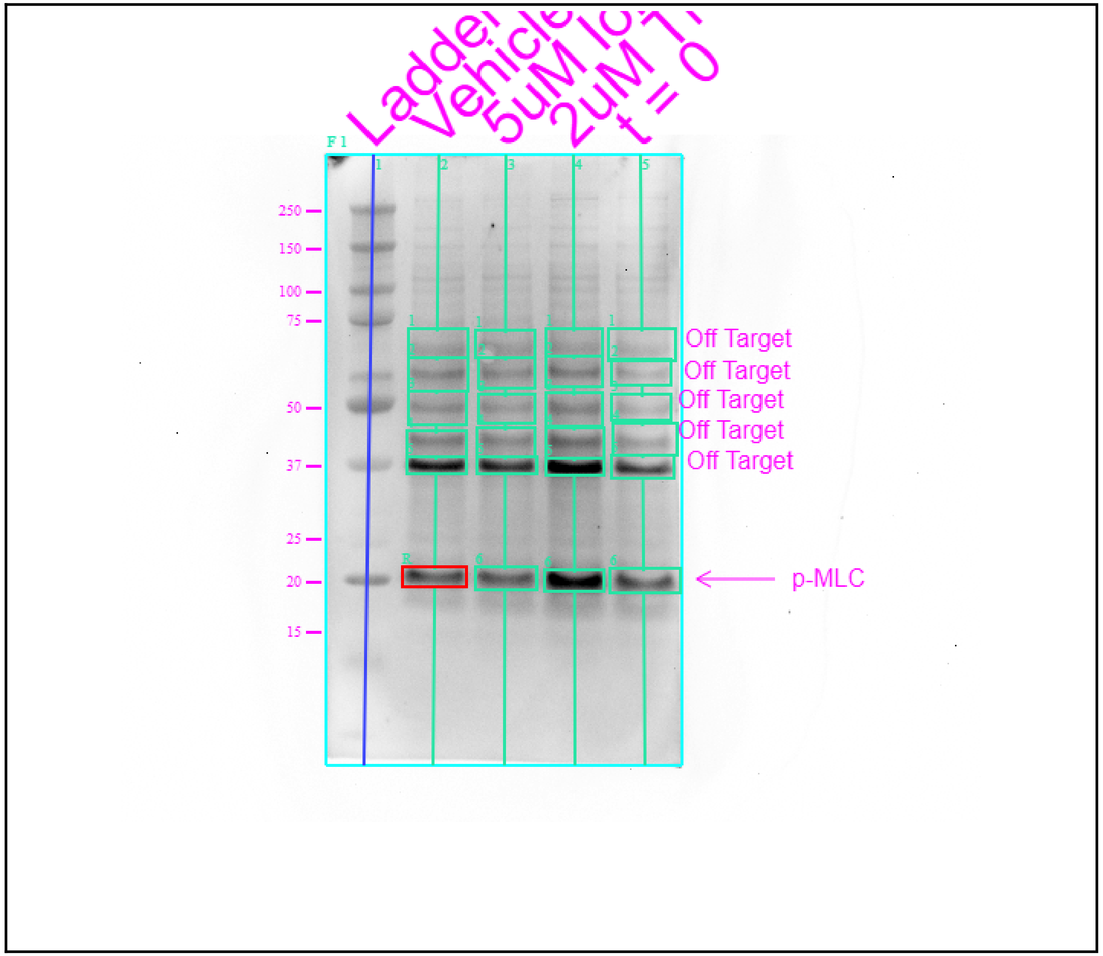

LANE AND BAND ANALYSIS DATA TABLE

pMLC CHEMI_01222022_143400

Frame: 1
Channel: Membrane
Sensitivity: 100
Molecular Weight Analysis Regression Method : Point to Point

Lane 1 - Ladder

#	Vol. (Int.)	Local Bg. Corr. Vol.	Area	Rf	Density	Local Bg. Corr. Den.	% band purity	% lane purity	Rolling Bg. Corr. Vol.	Rolling Bg. Corr. Den.	Mol. Wt.
1	10,926,376	1,575,185	288	0.087	37,938	5,469.393	13.341	3.324	1,841,920	6,395.556	250
2	10,663,716	1,714,607	296	0.15	36,026	5,792.594	11.527	2.872	1,591,552	5,376.865	150
3	9,084,151	1,607,616	259	0.22	35,073	6,207.014	10.936	2.725	1,509,888	5,829.683	100
4	12,662,998	1,328,493	407	0.268	31,113	3,264.111	8.59	2.141	1,186,048	2,914.123	75
5	15,056,241	2,661,497	468	0.41	32,171	5,686.96	19.574	4.878	2,702,592	5,774.769	50
6	14,613,838	2,078,816	481	0.505	30,382	4,321.864	15.716	3.916	2,169,856	4,511.135	37
7	10,048,555	404,066	370	0.625	27,158	1,092.071	2.529	0.63	349,184	943.741	25
8	10,159,223	1,087,683	333	0.695	30,508	3,266.318	8.364	2.084	1,154,816	3,467.916	20
9	13,761,054	1,202,272	444	0.777	30,993	2,707.821	9.423	2.348	1,300,992	2,930.162	15

Frame: 1
Channel: Chemi
Sensitivity: 100
Molecular Weight Analysis Regression Method : Point to Point

Lane 2 - Vehicle

#	Vol. (Int.)	Local Bg. Corr. Vol.	Area	Rf	Density	Local Bg. Corr. Den.
1	12,913,504	2,069,471	800	0.308	16,141	2,586.839
2	16,747,919	3,049,186	920	0.36	18,204	3,314.333
3	15,687,064	3,036,768	897	0.415	17,488	3,385.472
4	14,153,058	1,268,892	720	0.472	19,657	1,762.35
5	14,286,521	7,121,603	480	0.507	29,763	14,836
6	14,206,152	6,113,402	602	0.69	23,598	10,155

#	% band purity	% lane purity	Rolling Bg. Corr. Vol.	Rolling Bg. Corr. Den.	Mol. Wt.	Rel. Quant. (w/ LB Corr. Vol.)
1	4.863	3.16	1,050,880	1,313.6	67.982	0.339

#	% band purity	% lane purity	Rolling Bg. Corr. Vol.	Rolling Bg. Corr. Den.	Mol. Wt.	Rel. Quant. (w/ LB Corr. Vol.)
2	8.959	5.822	1,936,128	2,104.487	58.772	0.499
3	9.431	6.13	2,038,272	2,272.321	49.316	0.497
4	12.304	7.996	2,659,072	3,693.156	41.447	0.208
5	29.998	19.496	6,483,200	13,506	36.75	1.165
6	34.445	22.386	7,444,224	12,365	20.357	1

Lane 3 - 5uM Ionomycin

#	Vol. (Int.)	Local Bg. Corr. Vol.	Area	Rf	Density	Local Bg. Corr. Den.
1	12,854,771	1,748,426	760	0.31	16,914	2,300.561
2	14,709,738	2,628,471	798	0.357	18,433	3,293.823
3	13,066,551	2,195,407	760	0.415	17,192	2,888.694
4	14,751,141	1,146,313	780	0.47	18,911	1,469.633
5	13,404,431	5,583,695	480	0.507	27,925	11,632
6	15,305,808	4,963,220	656	0.692	23,332	7,565.885

#	% band purity	% lane purity	Rolling Bg. Corr. Vol.	Rolling Bg. Corr. Den.	Mol. Wt.	Rel. Quant. (w/ LB Corr. Vol.)
1	5.887	3.748	1,142,272	1,502.989	67.544	0.286
2	9.718	6.187	1,885,696	2,363.028	59.211	0.43
3	8.701	5.539	1,688,320	2,221.474	49.316	0.359
4	16.941	10.785	3,287,296	4,214.482	41.789	0.188
5	32.507	20.696	6,307,840	13,141	36.75	0.913
6	26.248	16.711	5,093,376	7,764.293	20.179	0.812

Lane 4 - 2uM Thapsigargin

#	Vol. (Int.)	Local Bg. Corr. Vol.	Area	Rf	Density	Local Bg. Corr. Den.
1	12,313,539	2,053,377	722	0.308	17,054	2,844.014
2	15,407,975	3,667,583	798	0.355	19,308	4,595.969
3	16,712,586	4,057,706	874	0.417	19,121	4,642.685
4	18,074,820	1,700,504	819	0.472	22,069	2,076.318
5	18,155,385	9,512,909	468	0.51	38,793	20,326
6	21,217,843	10,373,046	600	0.697	35,363	17,288

#	% band purity	% lane purity	Rolling Bg. Corr. Vol.	Rolling Bg. Corr. Den.	Mol. Wt.	Rel. Quant. (w/ LB Corr. Vol.)

#	% band purity	% lane purity	Rolling Bg. Corr. Vol.	Rolling Bg. Corr. Den.	Mol. Wt.	Rel. Quant. (w/ LB Corr. Vol.)
1	3.244	2.144	1,059,328	1,467.213	67.982	0.336
2	6.888	4.551	2,248,960	2,818.246	59.649	0.6
3	8.854	5.851	2,891,008	3,307.789	48.974	0.664
4	13.96	9.225	4,558,080	5,565.421	41.447	0.278
5	30.687	20.278	10,019,584	21,409	36.5	1.556
6	36.367	24.031	11,874,048	19,790	19.848	1.697

Lane 5 - t = 0

#	Vol. (Int.)	Local Bg. Corr. Vol.	Area	Rf	Density	Local Bg. Corr. Den.	[redacted]
1	11,818,554	2,454,187	990	0.31	11,937	2,478.977	[redacted]
2	9,183,471	1,761,013	720	0.355	12,754	2,445.852	[redacted]
3	8,910,868	1,874,062	720	0.412	12,376	2,602.864	[redacted]
4	12,356,646	2,970,276	946	0.465	13,061	3,139.827	[redacted]
5	13,820,829	6,251,649	672	0.51	20,566	9,303.05	[redacted]
6	16,368,031	7,264,118	799	0.697	20,485	9,091.513	[redacted]

#	% band purity	% lane purity	Rolling Bg. Corr. Vol.	Rolling Bg. Corr. Den.	Mol. Wt.	Rel. Quant. (w/ LB Corr. Vol.)
1	5.949	3.583	1,082,880	1,093.818	67.544	0.401
2	7.065	4.255	1,286,144	1,786.311	59.649	0.288
3	9.671	5.825	1,760,512	2,445.156	49.658	0.307
4	11.741	7.071	2,137,344	2,259.349	42.474	0.486
5	29.946	18.035	5,451,264	8,112	36.5	1.023
6	35.627	21.457	6,485,504	8,117.026	19.848	1.188

iBright™ Image Analysis Report

Katarina+ Chang

19 November 2022

total MYPT1 CHEMI_01232022_142151

Date:	23 January 2022 02:21:51PM
Mode:	Chemi Blots
Notes:	
Model:	FL1500
Instrument name:	2462619090234
Serial No:	2462619090234
Firmware version:	1.6.0
iBA version:	5.0
Image size:	563px X 450px
Image area:	112.7mm X 90.16mm
Optical Zoom:	2x
Digital Zoom:	1.2x
Focus level:	455
Resolution:	5 x 5
Exposure time:	3950 ms
Exposure mode:	Normal

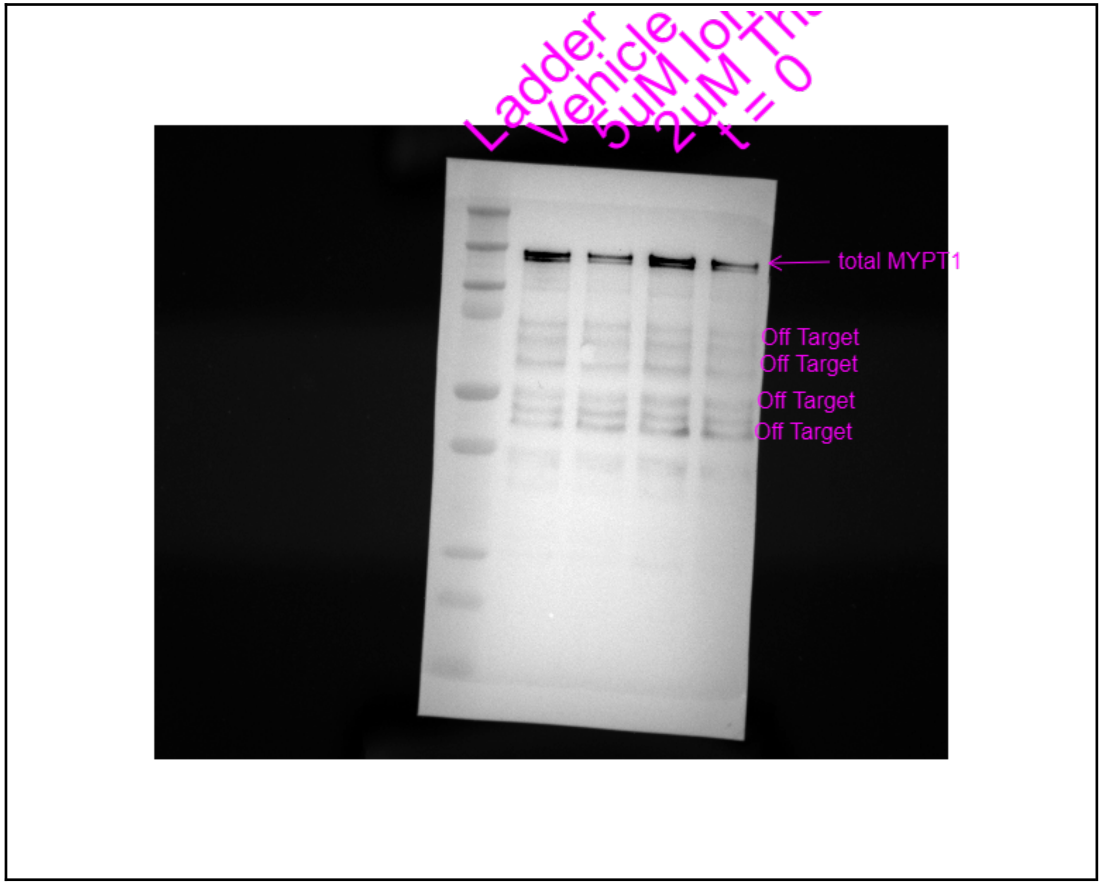

total MYPT1 CHEMI_01232022_142151

Date:	23 January 2022 02:21:51PM
Mode:	Chemi Blots
Notes:	
Model:	FL1500
Instrument name:	2462619090234
Serial No:	2462619090234
Firmware version:	1.6.0
iBA version:	5.0
Image size:	563px X 450px
Image area:	112.7mm X 90.16mm
Optical Zoom:	2x
Digital Zoom:	1.2x
Focus level:	455
Resolution:	5 x 5
Exposure time:	3950 ms
Exposure mode:	Normal

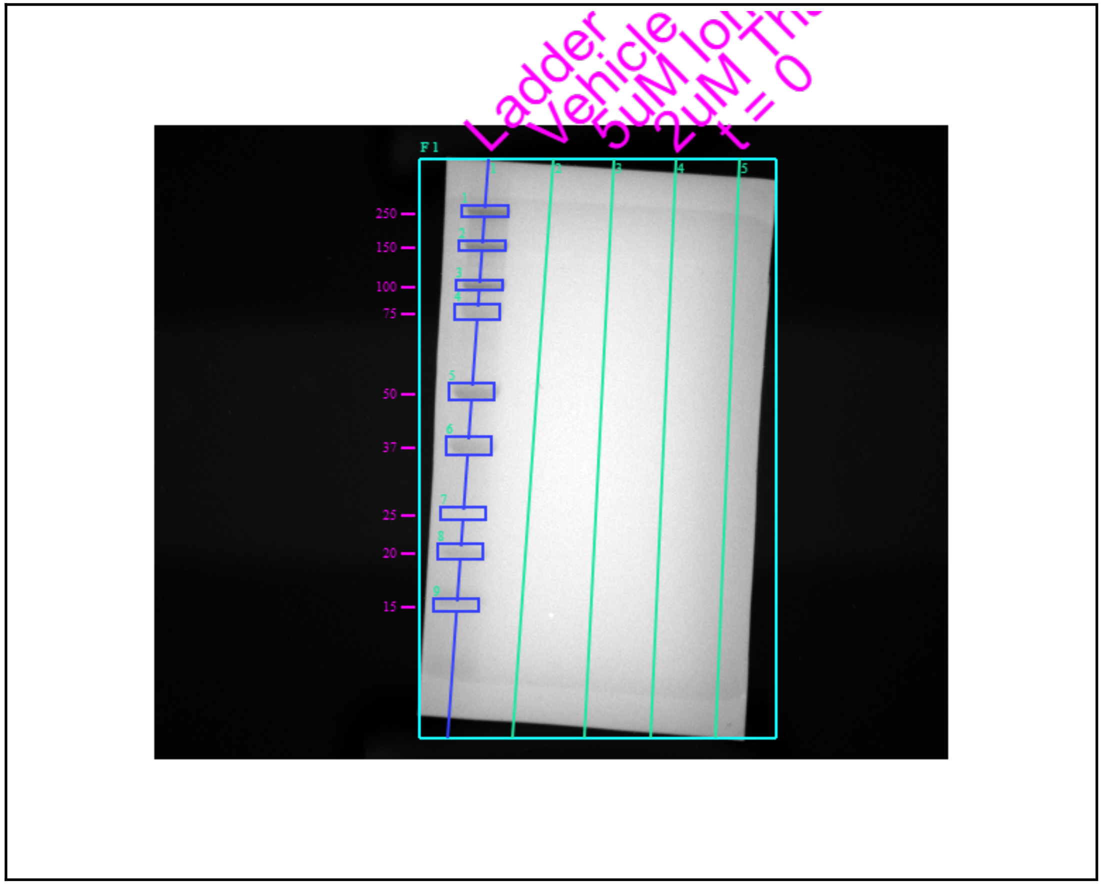

total MYPT1 CHEMI_01232022_142151

Date:	23 January 2022 02:21:51PM
Mode:	Chemi Blots
Notes:	
Model:	FL1500
Instrument name:	2462619090234
Serial No:	2462619090234
Firmware version:	1.6.0
iBA version:	5.0
Image size:	563px X 450px
Image area:	112.7mm X 90.16mm
Optical Zoom:	2x
Digital Zoom:	1.2x
Focus level:	455
Resolution:	5 x 5
Exposure time:	3950 ms
Exposure mode:	Normal

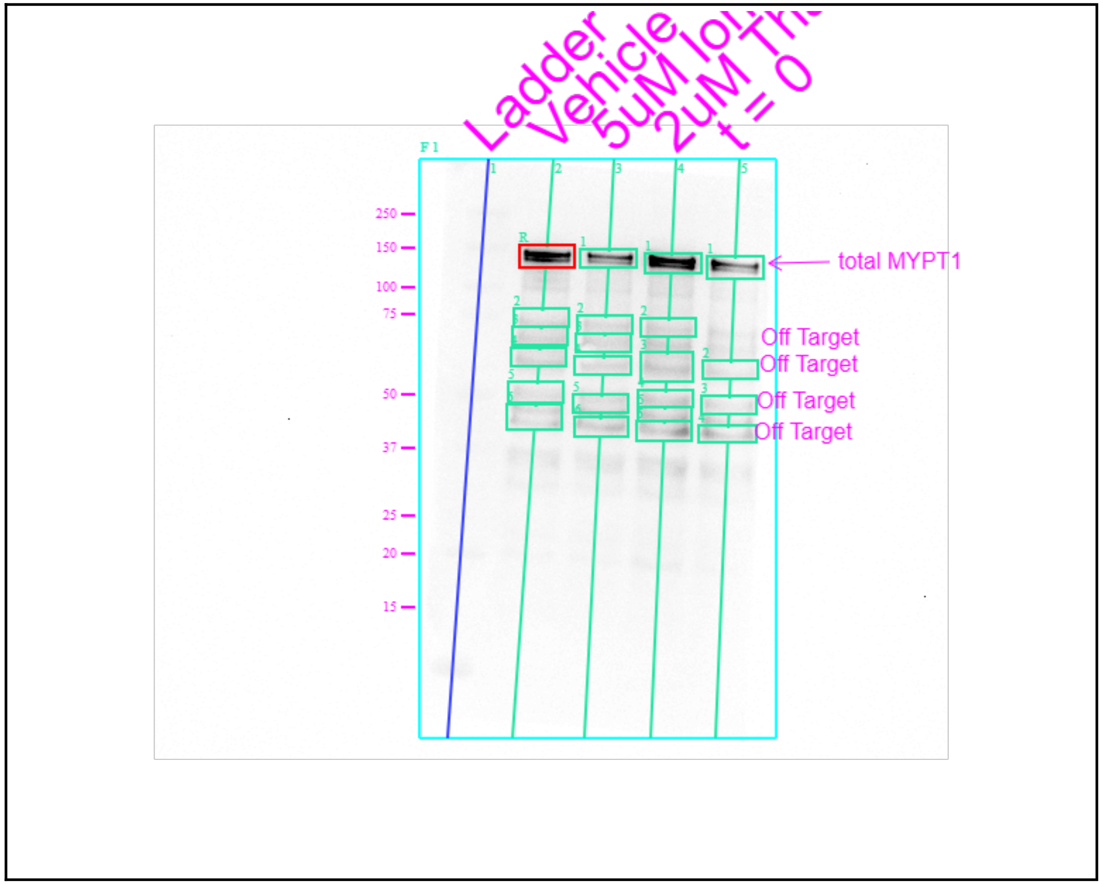

LANE AND BAND ANALYSIS DATA TABLE

total MYPT1 CHEMI_01232022_142151

Frame: 1
Channel: Membrane
Sensitivity: 100
Molecular Weight Analysis Regression Method : Point to Point

Lane 1 - Ladder

#	Vol. (Int.)	Local Bg. Corr. Vol.	Area	Rf	Density	Local Bg. Corr. Den.	% band purity	% lane purity	Rolling Bg. Corr. Vol.	Rolling Bg. Corr. Den.	Mol. Wt.
1	11,395,611	1,561,978	306	0.09	37,240	5,104.505	15.521	3.007	1,838,080	6,006.797	250
2	9,754,406	1,533,732	272	0.148	35,861	5,638.723	12.056	2.336	1,427,712	5,248.941	150
3	9,347,472	1,548,056	272	0.217	34,365	5,691.385	12.535	2.429	1,484,544	5,457.882	100
4	12,034,028	1,116,798	396	0.263	30,388	2,820.198	8.093	1.568	958,464	2,420.364	75
5	13,422,805	2,091,296	429	0.401	31,288	4,874.818	19.507	3.779	2,310,144	5,384.951	50
6	13,615,618	1,704,256	462	0.494	29,471	3,688.866	15.196	2.944	1,799,680	3,895.411	37
7	8,727,764	247,558	330	0.611	26,447	750.177	1.935	0.375	229,120	694.303	25
8	11,523,361	921,237	396	0.676	29,099	2,326.357	8.32	1.612	985,344	2,488.242	20
9	10,022,899	488,186	330	0.769	30,372	1,479.354	6.837	1.325	809,728	2,453.721	15

Frame: 1
Channel: Chemi
Sensitivity: 100
Molecular Weight Analysis Regression Method : Point to Point

Lane 2 - Vehicle

#	Vol. (Int.)	Local Bg. Corr. Vol.	Area	Rf	Density	Local Bg. Corr. Den.
1	6,859,968	5,580,482	680	0.168	10,088	8,206.592
2	1,280,597	368,423	560	0.273	2,286.78	657.899
3	1,584,333	446,763	560	0.304	2,829.166	797.792
4	1,538,956	489,382	560	0.341	2,748.136	873.897
5	1,298,660	330,446	640	0.401	2,029.156	516.323
6	1,831,822	795,097	760	0.445	2,410.292	1,046.181

#	% band purity	% lane purity	Rolling Bg. Corr. Vol.	Rolling Bg. Corr. Den.	Mol. Wt.	Rel. Quant. (w/ LB Corr. Vol.)
1	78.645	68.133	5,350,144	7,867.859	135.714	1

#	% band purity	% lane purity	Rolling Bg. Corr. Vol.	Rolling Bg. Corr. Den.	Mol. Wt.	Rel. Quant. (w/ LB Corr. Vol.)
2	2.687	2.328	182,784	326.4	73.246	0.066
3	3.304	2.862	224,768	401.371	67.544	0.08
4	4.99	4.323	339,456	606.171	60.965	0.088
5	3.752	3.25	255,232	398.8	50	0.059
6	6.623	5.738	450,560	592.842	43.842	0.142

Lane 3 - 5uM Ionomycin

#	Vol. (Int.)	Local Bg. Corr. Vol.	Area	Rf	Density	Local Bg. Corr. Den.
1	3,398,932	2,323,583	574	0.17	5,921.484	4,048.055
2	1,355,615	345,712	560	0.285	2,420.741	617.344
3	1,414,487	391,403	560	0.316	2,525.87	698.934
4	1,284,214	421,243	574	0.355	2,237.307	733.873
5	1,529,844	438,094	560	0.421	2,731.864	782.312
6	1,639,668	594,129	585	0.462	2,802.851	1,015.606

#	% band purity	% lane purity	Rolling Bg. Corr. Vol.	Rolling Bg. Corr. Den.	Mol. Wt.	Rel. Quant. (w/ LB Corr. Vol.)
1	57.774	44.072	2,367,744	4,124.99	133.929	0.416
2	4.572	3.488	187,392	334.629	71.053	0.062
3	5.472	4.174	224,256	400.457	65.351	0.07
4	7.158	5.461	293,376	511.108	58.333	0.075
5	8.595	6.557	352,256	629.029	47.263	0.079
6	16.428	12.532	673,280	1,150.906	41.447	0.106

Lane 4 - 2uM Thapsigargin

#	Vol. (Int.)	Local Bg. Corr. Vol.	Area	Rf	Density	Local Bg. Corr. Den.
1	7,660,952	6,220,432	615	0.18	12,456	10,114
2	1,498,507	394,919	560	0.29	2,675.905	705.213
3	2,498,384	907,850	836	0.358	2,988.498	1,085.946
4	1,490,212	547,770	520	0.414	2,865.792	1,053.405
5	1,437,242	306,759	429	0.44	3,350.214	715.057
6	2,055,929	901,597	600	0.47	3,426.548	1,502.662

#	% band purity	% lane purity	Rolling Bg. Corr. Vol.	Rolling Bg. Corr. Den.	Mol. Wt.	Rel. Quant. (w/ LB Corr. Vol.)

#	% band purity	% lane purity	Rolling Bg. Corr. Vol.	Rolling Bg. Corr. Den.	Mol. Wt.	Rel. Quant. (w/ LB Corr. Vol.)
1	73.156	61.41	6,262,784	10,183	126.786	1.115
2	1.89	1.586	161,792	288.914	70.175	0.071
3	5.84	4.902	499,968	598.048	57.895	0.163
4	4.45	3.735	380,928	732.554	48.289	0.098
5	4.028	3.381	344,832	803.804	44.526	0.055
6	10.637	8.929	910,592	1,517.653	40.421	0.162

Lane 5 - t = 0

#	Vol. (Int.)	Local Bg. Corr. Vol.	Area	Rf	Density	Local Bg. Corr. Den.
1	3,458,475	2,738,719	697	0.187	4,961.944	3,929.297
2	985,592	354,308	560	0.363	1,759.986	632.693
3	1,004,279	326,806	560	0.423	1,793.355	583.584
4	1,393,275	677,359	546	0.474	2,551.786	1,240.586

#	% band purity	% lane purity	Rolling Bg. Corr. Vol.	Rolling Bg. Corr. Den.	Mol. Wt.	Rel. Quant. (w/ LB Corr. Vol.)
1	68.839	51.566	2,776,832	3,983.977	121.429	0.491
2	6.569	4.92	264,960	473.143	57.018	0.063
3	7.33	5.491	295,680	528	46.921	0.059
4	17.262	12.931	696,320	1,275.311	39.737	0.121

iBright™ Image Analysis Report

Katarina+ Chang
19 November 2022

Acetyl tubulin CHEMI_01242022_13315 0

Date:	24 January 2022 01:31:50PM
Mode:	Chemi Blots
Notes:	
Model:	FL1500
Instrument name:	2462619090234
Serial No:	2462619090234
Firmware version:	1.6.0
iBA version:	5.0
Image size:	615px X 491px
Image area:	112.7mm X 90.16mm
Optical Zoom:	2x
Digital Zoom:	1.1x
Focus level:	455
Resolution:	5 x 5
Exposure time:	1723 ms
Exposure mode:	Normal

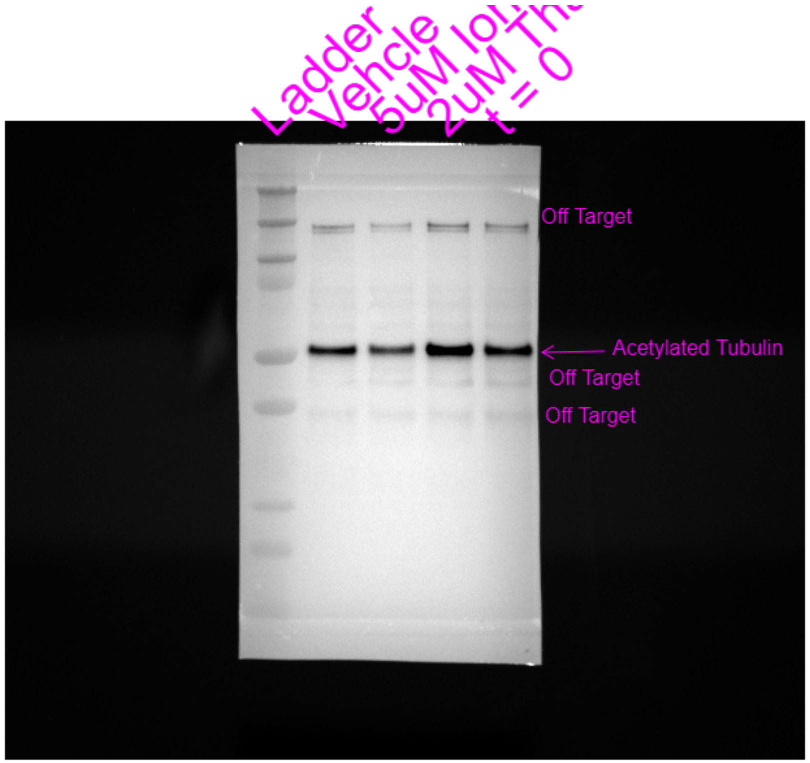

Acetyl tubulin CHEMI_01242022_13315
0

Date:	24 January 2022 01:31:50PM
Mode:	Chemi Blots
Notes:	
Model:	FL1500
Instrument name:	2462619090234
Serial No:	2462619090234
Firmware version:	1.6.0
iBA version:	5.0
Image size:	615px X 491px
Image area:	112.7mm X 90.16mm
Optical Zoom:	2x
Digital Zoom:	1.1x
Focus level:	455
Resolution:	5 x 5
Exposure time:	1723 ms
Exposure mode:	Normal

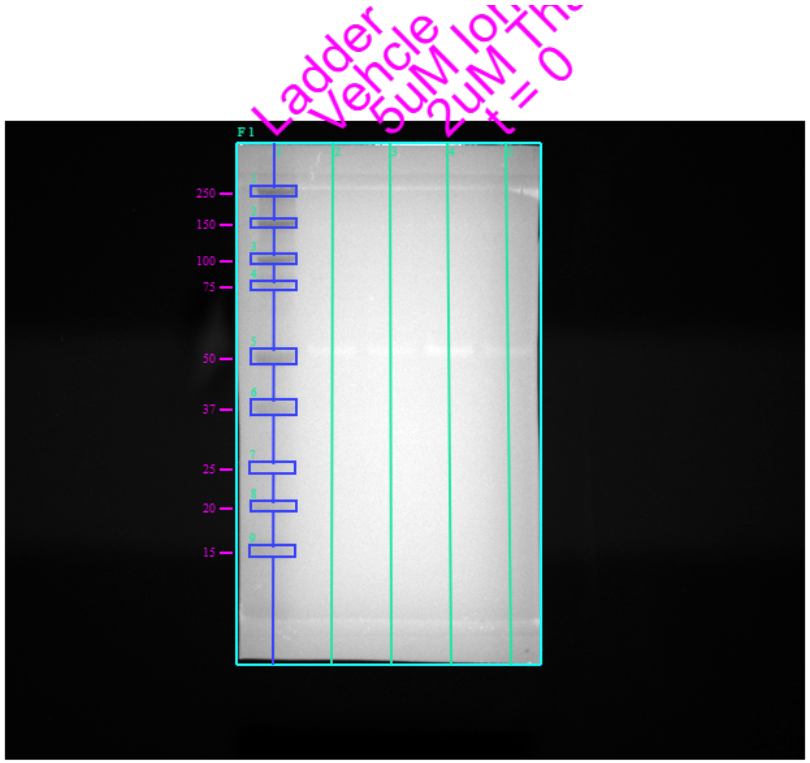

Acetyl tubulin CHEMI_01242022_13315 0

Date:	24 January 2022 01:31:50PM
Mode:	Chemi Blots
Notes:	
Model:	FL1500
Instrument name:	2462619090234
Serial No:	2462619090234
Firmware version:	1.6.0
iBA version:	5.0
Image size:	615px X 491px
Image area:	112.7mm X 90.16mm
Optical Zoom:	2x
Digital Zoom:	1.1x
Focus level:	455
Resolution:	5 x 5
Exposure time:	1723 ms
Exposure mode:	Normal

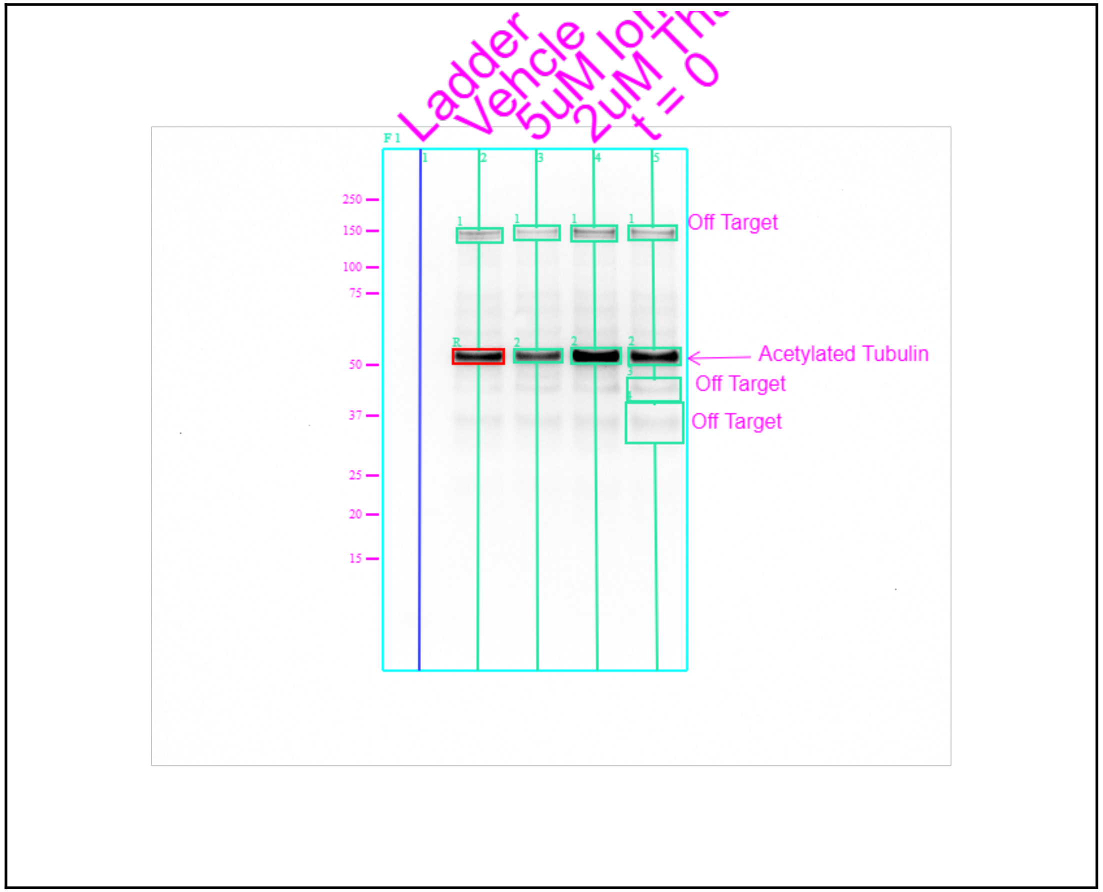

LANE AND BAND ANALYSIS DATA TABLE

Acetyl tubulin CHEMI_01242022_133150

Frame: 1
Channel: Membrane
Sensitivity: 100
Molecular Weight Analysis Regression Method : Point to Point

Lane 1 - Ladder

#	Vol. (Int.)	Local Bg. Corr. Vol.	Area	Rf	Density	Local Bg. Corr. Den.	% band purity	% lane purity	Rolling Bg. Corr. Vol.	Rolling Bg. Corr. Den.	Mol. Wt.
1	12,163,752	1,665,612	324	0.092	37,542	5,140.78	14.955	2.425	1,726,720	5,329.383	250
2	10,466,281	1,551,225	288	0.152	36,341	5,386.199	12.266	1.989	1,416,192	4,917.333	150
3	11,117,666	1,611,208	324	0.222	34,313	4,972.865	13.057	2.117	1,507,584	4,653.037	100
4	8,921,867	724,935	288	0.272	30,978	2,517.138	6.465	1.048	746,496	2,592	75
5	14,718,155	2,189,930	468	0.409	31,449	4,679.339	19.654	3.187	2,269,184	4,848.684	50
6	13,944,378	1,701,623	468	0.506	29,795	3,635.948	15.412	2.499	1,779,456	3,802.256	37
7	9,650,694	332,091	360	0.621	26,807	922.477	2.725	0.442	314,624	873.956	25
8	9,424,058	818,271	324	0.696	29,086	2,525.53	7.618	1.235	879,616	2,714.864	20
9	10,835,899	798,456	360	0.781	30,099	2,217.935	7.847	1.272	905,984	2,516.622	15

Frame: 1
Channel: Chemi
Sensitivity: 100
Molecular Weight Analysis Regression Method : Point to Point

Lane 2 - Vehcle

#	Vol. (Int.)	Local Bg. Corr. Vol.	Area	Rf	Density	Local Bg. Corr. Den.
1	2,375,913	1,811,397	432	0.165	5,499.799	4,193.05
2	8,784,113	7,106,862	480	0.397	18,300	14,805

#	% band purity	% lane purity	Rolling Bg. Corr. Vol.	Rolling Bg. Corr. Den.	Mol. Wt.	Rel. Quant. (w/ LB Corr. Vol.)
1	19.072	15.667	1,780,224	4,120.889	141.071	0.255
2	80.928	66.477	7,553,792	15,737	52.273	1

Lane 3 - 5uM Ionomycin

#	Vol. (Int.)	Local Bg. Corr. Vol.	Area	Rf	Density	Local Bg. Corr. Den.

#	Vol. (Int.)	Local Bg. Corr. Vol.	Area	Rf	Density	Local Bg. Corr. Den.
1	1,594,271	1,081,157	432	0.16	3,690.442	2,502.679
2	6,780,453	5,036,202	418	0.397	16,221	12,048

#	% band purity	% lane purity	Rolling Bg. Corr. Vol.	Rolling Bg. Corr. Den.	Mol. Wt.	Rel. Quant. (w/ LB Corr. Vol.)
1	16.016	11.491	1,068,800	2,474.074	144.643	0.152
2	83.984	60.252	5,604,352	13,407	52.273	0.709

Lane 4 - 2uM Thapsigargin

#	Vol. (Int.)	Local Bg. Corr. Vol.	Area	Rf	Density	Local Bg. Corr. Den.
1	3,073,695	2,357,021	468	0.162	6,567.724	5,036.371
2	14,065,279	11,302,265	507	0.397	27,742	22,292

#	% band purity	% lane purity	Rolling Bg. Corr. Vol.	Rolling Bg. Corr. Den.	Mol. Wt.	Rel. Quant. (w/ LB Corr. Vol.)
1	50.812	33.611	2,419,712	5,170.325	142.857	0.332
2	49.188	32.537	2,342,400	4,620.118	52.273	1.59

Lane 5 - t = 0

#	Vol. (Int.)	Local Bg. Corr. Vol.	Area	Rf	Density	Local Bg. Corr. Den.
1	2,490,602	1,923,014	456	0.16	5,461.846	4,217.136
2	10,732,177	8,707,504	574	0.397	18,697	15,169
3	2,315,548	726,771	798	0.461	2,901.689	910.741
4	2,908,285	1,295,566	1,440	0.524	2,019.642	899.699

#	% band purity	% lane purity	Rolling Bg. Corr. Vol.	Rolling Bg. Corr. Den.	Mol. Wt.	Rel. Quant. (w/ LB Corr. Vol.)
1	14.981	13.312	1,896,960	4,160	144.643	0.271
2	73.436	65.254	9,298,688	16,199	52.273	1.225
3	6.344	5.637	803,328	1,006.677	43	0.102
4	5.238	4.655	663,296	460.622	35.174	0.182
