# Supplementary material for: Elevation of Cytoplasmic Calcium Suppresses Microtentacle Formation and Function in Breast Tumor Cells
Source: Cancers (Basel). 2023 Jan 31;15(3):884. doi: 10.3390/cancers15030884 (PMC9913253; doi:10.3390/cancers15030884)
Supplement: Supplementary file 1 [file cancers-15-00884-s001.zip › cancers-2080686-Supplementary/File S1_ Original Blots/Original Immunoblot Images MDAMB436 Biological Replicate 2.pdf]

iBright™ Image Analysis Report

Katarina+ Chang

19 November 2022

GAPDH_CHEMI_01312022_112817

Date:	31 January 2022 11:28:17AM
Mode:	Chemi Blots
Notes:	
Model:	FL1500
Instrument name:	2462619090234
Serial No:	2462619090234
Firmware version:	1.6.0
iBA version:	5.0
Image size:	615px X 491px
Image area:	112.7mm X 90.16mm
Optical Zoom:	2x
Digital Zoom:	1.1x
Focus level:	455
Resolution:	5 x 5
Exposure time:	14270 ms
Exposure mode:	Normal

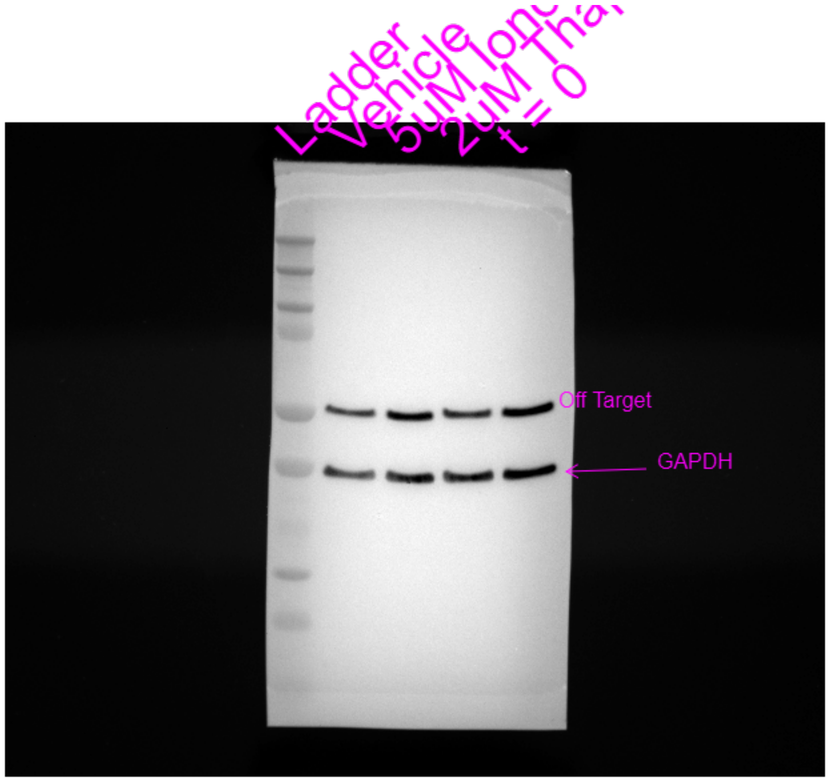

GAPDH_CHEMI_01312022_112817

Date:	31 January 2022 11:28:17AM
Mode:	Chemi Blots
Notes:	
Model:	FL1500
Instrument name:	2462619090234
Serial No:	2462619090234
Firmware version:	1.6.0
iBA version:	5.0
Image size:	615px X 491px
Image area:	112.7mm X 90.16mm
Optical Zoom:	2x
Digital Zoom:	1.1x
Focus level:	455
Resolution:	5 x 5
Exposure time:	14270 ms
Exposure mode:	Normal

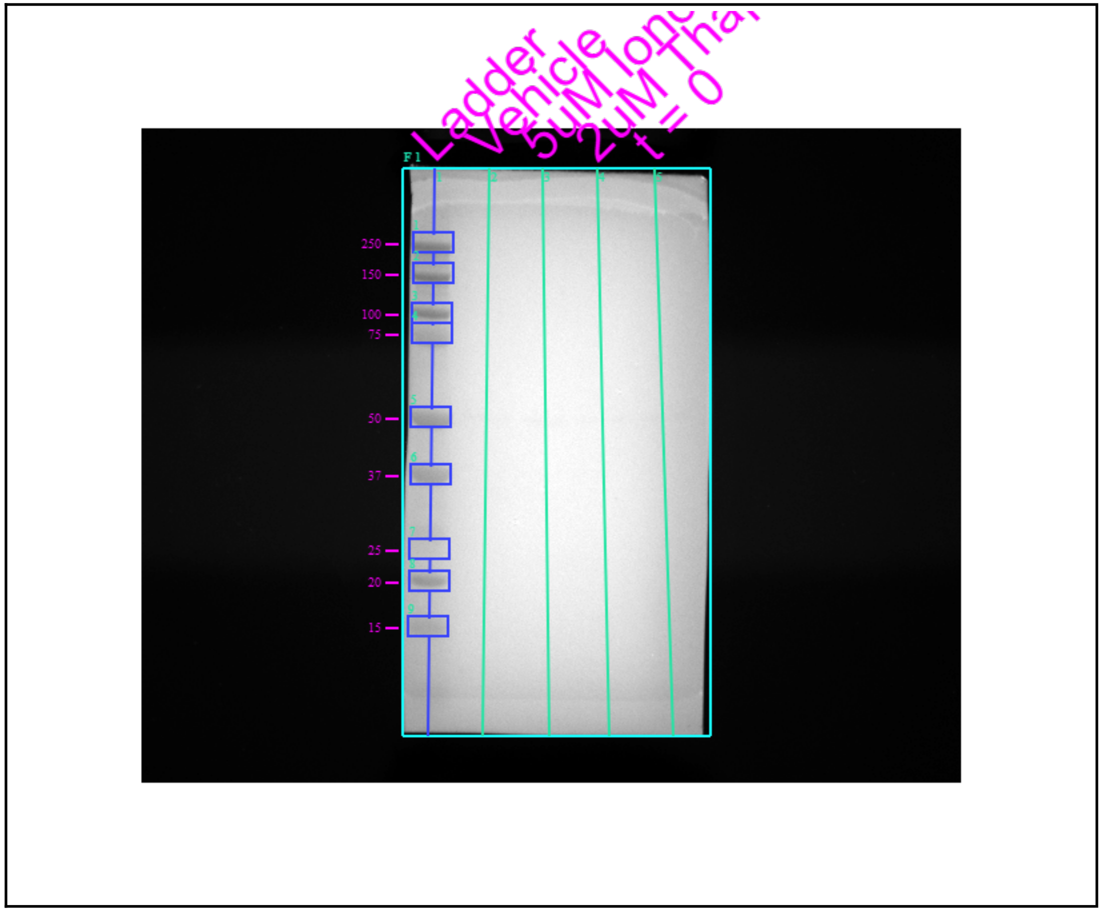

GAPDH_CHEMI_01312022_112817

Date:	31 January 2022 11:28:17AM
Mode:	Chemi Blots
Notes:	
Model:	FL1500
Instrument name:	2462619090234
Serial No:	2462619090234
Firmware version:	1.6.0
iBA version:	5.0
Image size:	615px X 491px
Image area:	112.7mm X 90.16mm
Optical Zoom:	2x
Digital Zoom:	1.1x
Focus level:	455
Resolution:	5 x 5
Exposure time:	14270 ms
Exposure mode:	Normal

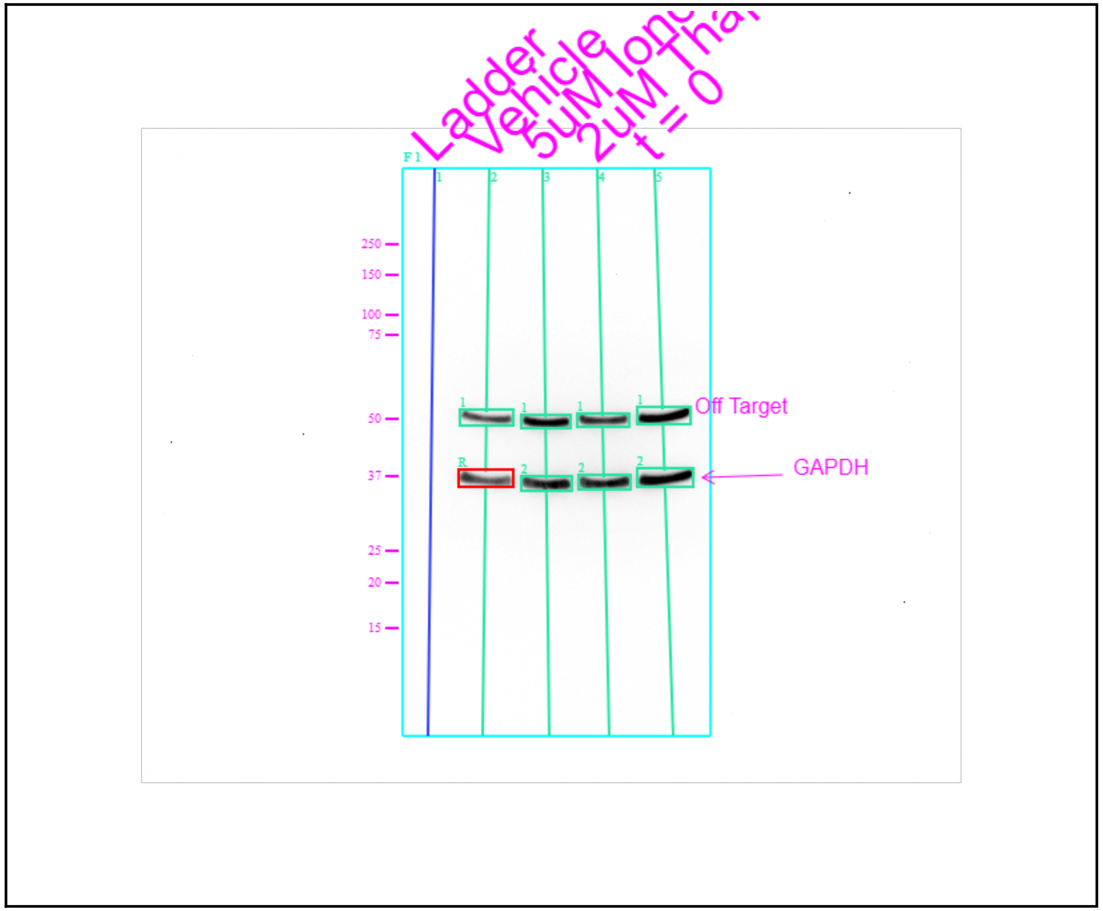

LANE AND BAND ANALYSIS DATA TABLE

GAPDH_CHEMI_01312022_112817

Frame: 1
Channel: Membrane
Sensitivity: 100
Molecular Weight Analysis Regression Method : Point to Point

Lane 1 - Ladder

#	Vol. (Int.)	Local Bg. Corr. Vol.	Area	Rf	Density	Local Bg. Corr. Den.	% band purity	% lane purity	Rolling Bg. Corr. Vol.	Rolling Bg. Corr. Den.	Mol. Wt.
1	17,399,806	779,132	496	0.129	35,080	1,570.831	12.212	3.932	1,963,776	3,959.226	250
2	17,328,676	937,069	496	0.183	34,936	1,889.253	12.36	3.98	1,987,584	4,007.226	150
3	16,701,661	726,901	496	0.254	33,672	1,465.527	12.604	4.058	2,026,752	4,086.194	100
4	15,753,633	55,066	496	0.289	31,761	111.022	8.453	2.722	1,359,360	2,740.645	75
5	16,130,978	745,153	496	0.437	32,522	1,502.325	16.856	5.428	2,710,528	5,464.774	50
6	15,654,083	1,421,053	496	0.538	31,560	2,865.026	14.323	4.612	2,303,232	4,643.613	37
7	13,977,547	126,842	496	0.669	28,180	255.73	3.249	1.046	522,496	1,053.419	25
8	15,582,895	1,558,652	496	0.725	31,417	3,142.445	11.177	3.599	1,797,376	3,623.742	20
9	15,805,070	800,429	496	0.805	31,865	1,613.769	8.765	2.822	1,409,536	2,841.806	15

Frame: 1
Channel: Chemi
Sensitivity: 100
Molecular Weight Analysis Regression Method : Point to Point

Lane 2 - Vehicle

#	Vol. (Int.)	Local Bg. Corr. Vol.	Area	Rf	Density	Local Bg. Corr. Den.
1	7,220,902	6,152,453	533	0.439	13,547	11,543
2	8,481,199	7,232,442	588	0.545	14,423	12,300

#	% band purity	% lane purity	Rolling Bg. Corr. Vol.	Rolling Bg. Corr. Den.	Mol. Wt.	Rel. Quant. (w/ LB Corr. Vol.)
1	45.933	44.243	6,538,240	12,266	49.698	0.851
2	54.067	52.079	7,696,128	13,088	36.357	1

Lane 3 - 5uM Ionomycin

#	Vol. (Int.)	Local Bg. Corr. Vol.	Area	Rf	Density	Local Bg. Corr. Den.

#	Vol. (Int.)	Local Bg. Corr. Vol.	Area	Rf	Density	Local Bg. Corr. Den.
1	10,029,935	8,532,756	418	0.446	23,995	20,413
2	10,681,672	8,825,363	468	0.554	22,824	18,857

#	% band purity	% lane purity	Rolling Bg. Corr. Vol.	Rolling Bg. Corr. Den.	Mol. Wt.	Rel. Quant. (w/ LB Corr. Vol.)
1	18.901	16.407	2,322,176	5,555.445	48.791	1.18
2	81.099	70.398	9,963,520	21,289	35.5	1.22

Lane 4 - 2uM Thapsigargin

#	Vol. (Int.)	Local Bg. Corr. Vol.	Area	Rf	Density	Local Bg. Corr. Den.
1	8,317,233	6,732,394	440	0.444	18,902	15,300
2	10,991,803	8,916,885	480	0.552	22,899	18,576

#	% band purity	% lane purity	Rolling Bg. Corr. Vol.	Rolling Bg. Corr. Den.	Mol. Wt.	Rel. Quant. (w/ LB Corr. Vol.)
1	42.258	38.035	7,324,416	16,646	49.093	0.931
2	57.742	51.972	10,008,320	20,850	35.714	1.233

Lane 5 - t = 0

#	Vol. (Int.)	Local Bg. Corr. Vol.	Area	Rf	Density	Local Bg. Corr. Den.
1	13,384,638	12,122,038	574	0.434	23,318	21,118
2	14,544,006	12,700,969	645	0.545	22,548	19,691

#	% band purity	% lane purity	Rolling Bg. Corr. Vol.	Rolling Bg. Corr. Den.	Mol. Wt.	Rel. Quant. (w/ LB Corr. Vol.)
1	48.646	48.15	12,651,520	22,040	50.397	1.676
2	51.354	50.832	13,356,032	20,707	36.357	1.756

iBright™ Image Analysis Report

Katarina+ Chang
19 November 2022

MLCK CHEMI_01262022_123956

Date:	26 January 2022 12:39:56PM
Mode:	Chemi Blots
Notes:	
Model:	FL1500
Instrument name:	2462619090234
Serial No:	2462619090234
Firmware version:	1.6.0
iBA version:	5.0
Image size:	615px X 491px
Image area:	112.7mm X 90.16mm
Optical Zoom:	2x
Digital Zoom:	1.1x
Focus level:	455
Resolution:	5 x 5
Exposure time:	60000 ms
Exposure mode:	Normal

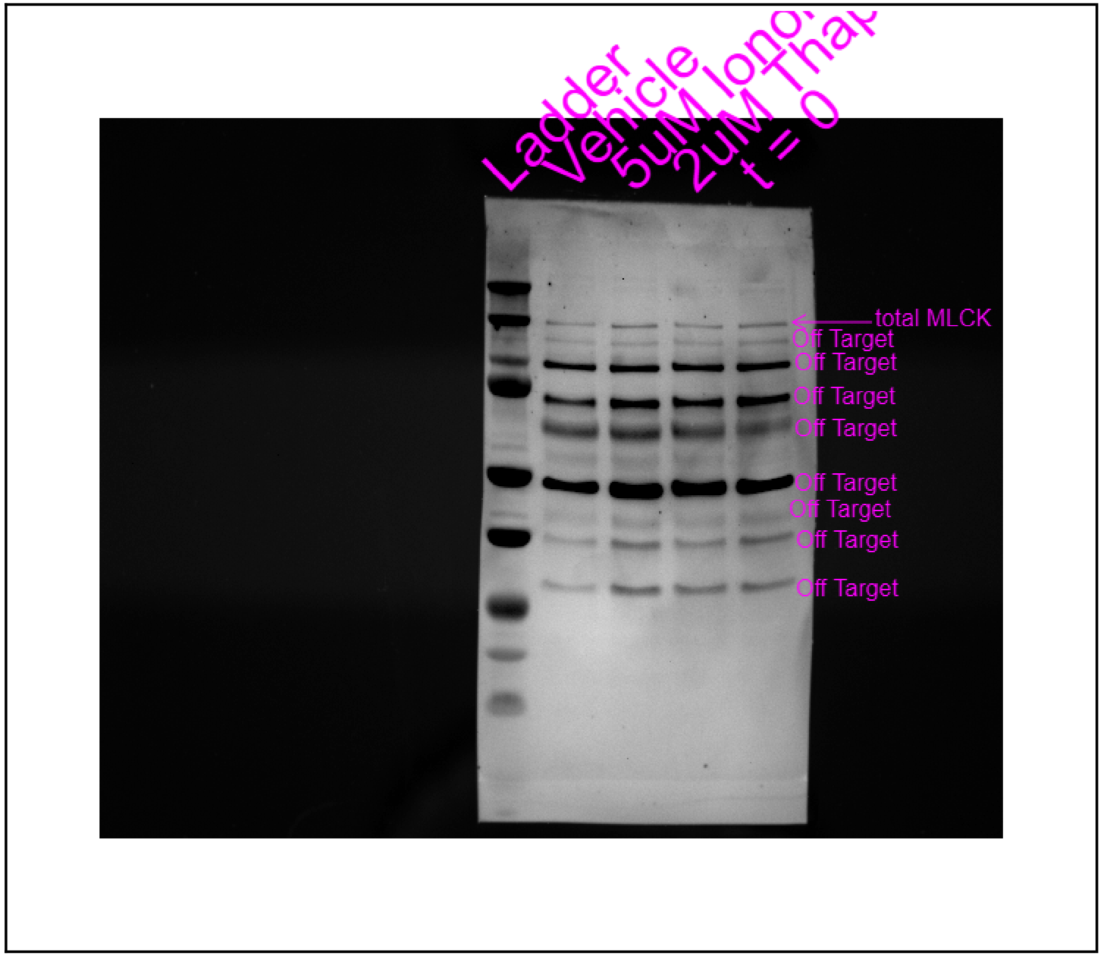

MLCK CHEMI_01262022_123956

Date:	26 January 2022 12:39:56PM
Mode:	Chemi Blots
Notes:	
Model:	FL1500
Instrument name:	2462619090234
Serial No:	2462619090234
Firmware version:	1.6.0
iBA version:	5.0
Image size:	615px X 491px
Image area:	112.7mm X 90.16mm
Optical Zoom:	2x
Digital Zoom:	1.1x
Focus level:	455
Resolution:	5 x 5
Exposure time:	60000 ms
Exposure mode:	Normal

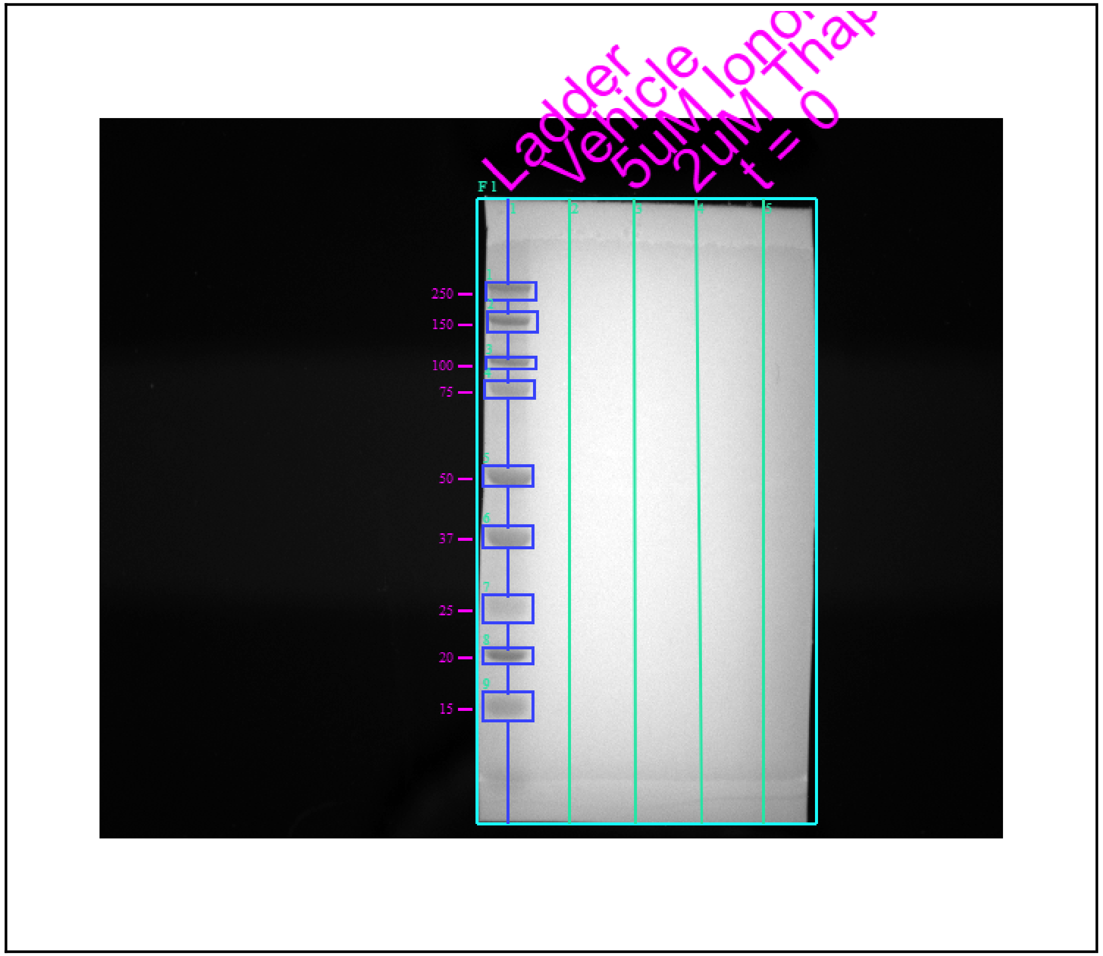

MLCK CHEMI_01262022_123956

Date:	26 January 2022 12:39:56PM
Mode:	Chemi Blots
Notes:	
Model:	FL1500
Instrument name:	2462619090234
Serial No:	2462619090234
Firmware version:	1.6.0
iBA version:	5.0
Image size:	615px X 491px
Image area:	112.7mm X 90.16mm
Optical Zoom:	2x
Digital Zoom:	1.1x
Focus level:	455
Resolution:	5 x 5
Exposure time:	60000 ms
Exposure mode:	Normal

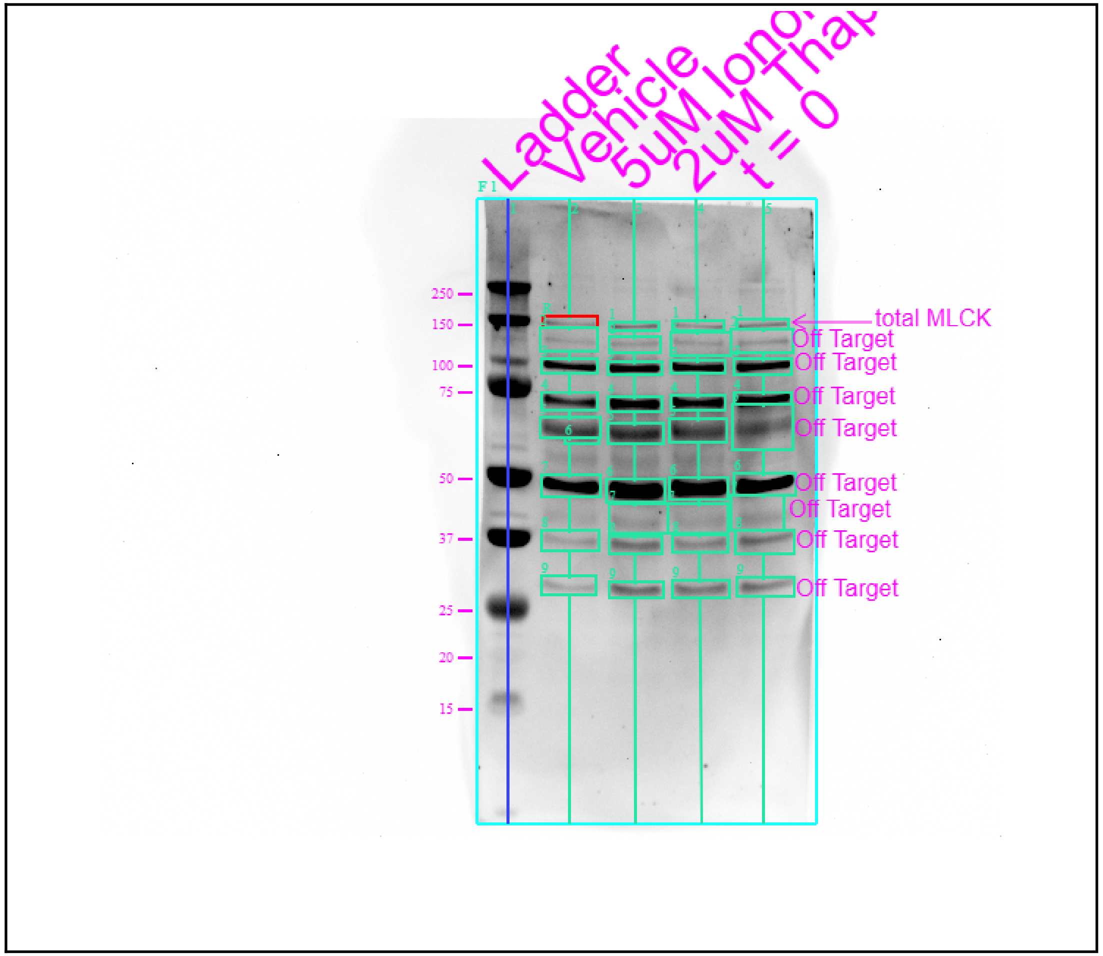

LANE AND BAND ANALYSIS DATA TABLE

MLCK CHEMI_01262022_123956

Frame: 1
Channel: Membrane
Sensitivity: 100
Molecular Weight Analysis Regression Method : Point to Point

Lane 1 - Ladder

#	Vol. (Int.)	Local Bg. Corr. Vol.	Area	Rf	Density	Local Bg. Corr. Den.	% band purity	% lane purity	Rolling Bg. Corr. Vol.	Rolling Bg. Corr. Den.	Mol. Wt.
1	15,601,926	573,158	455	0.148	34,289	1,259.689	9.132	3.22	1,960,448	4,308.677	250
2	17,141,492	1,049,846	525	0.197	32,650	1,999.708	8.92	3.146	1,914,880	3,647.39	150
3	10,337,019	726,754	315	0.263	32,815	2,307.157	7.217	2.545	1,549,312	4,918.451	100
4	14,050,552	192,716	455	0.305	30,880	423.552	8.282	2.921	1,777,920	3,907.516	75
5	17,480,217	1,075,077	525	0.444	33,295	2,047.767	18.575	6.551	3,987,712	7,595.642	50
6	17,719,278	1,232,800	560	0.54	31,641	2,201.429	15.668	5.525	3,363,584	6,006.4	37
7	19,699,214	214,270	700	0.655	28,141	306.1	7.739	2.729	1,661,440	2,373.486	25
8	14,025,294	2,059,481	420	0.73	33,393	4,903.528	13.391	4.723	2,874,880	6,844.952	20
9	22,902,987	1,481,261	735	0.812	31,160	2,015.322	11.078	3.907	2,378,240	3,235.701	15

Frame: 1
Channel: Chemi
Sensitivity: 100
Molecular Weight Analysis Regression Method : Point to Point

Lane 2 - Vehicle

#	Vol. (Int.)	Local Bg. Corr. Vol.	Area	Rf	Density	Local Bg. Corr. Den.
1	8,505,137	1,173,386	342	0.197	24,868	3,430.955
2	15,743,654	307,534	680	0.225	23,152	452.257
3	17,778,818	8,098,286	429	0.268	41,442	18,877
4	19,696,336	7,733,510	507	0.324	38,848	15,253
5	24,271,637	8,268,667	630	0.366	38,526	13,124
6	3,806,682	169,273	120	0.387	31,722	1,410.611
7	29,077,443	13,685,408	656	0.458	44,325	20,861
8	12,763,847	1,742,455	600	0.547	21,273	2,904.093

#	Vol. (Int.)	Local Bg. Corr. Vol.	Area	Rf	Density	Local Bg. Corr. Den.	[redacted]
9	9,650,144	2,207,740	532	0.617	18,139	4,149.888	

#	% band purity	% lane purity	Rolling Bg. Corr. Vol.	Rolling Bg. Corr. Den.	Mol. Wt.	Rel. Quant. (w/ LB Corr. Vol.)
1	2.385	1.681	1,156,864	3,382.643	150	1
2	1.892	1.334	917,760	1,349.647	128.571	0.262
3	17.705	12.481	8,587,264	20,016	97.222	6.902
4	17.635	12.431	8,553,216	16,870	71.61	6.591
5	19.527	13.765	9,470,720	15,032	63.983	7.047
6	1.749	1.233	848,384	7,069.867	60.169	0.144
7	29.084	20.502	14,106,112	21,503	48.098	11.663
8	4.612	3.251	2,236,672	3,727.787	36.265	1.485
9	5.412	3.815	2,624,768	4,933.774	28.918	1.882

Lane 3 - 5uM Ionomycin

#	Vol. (Int.)	Local Bg. Corr. Vol.	Area	Rf	Density	Local Bg. Corr. Den.	[redacted]
1	6,603,245	1,670,641	245	0.204	26,952	6,818.945	
2	10,382,962	665,919	432	0.232	24,034	1,541.48	
3	17,547,273	8,393,736	380	0.27	46,177	22,088	
4	20,785,979	9,320,495	418	0.329	49,727	22,297	
5	25,603,752	8,780,682	585	0.376	43,767	15,009	
6	35,945,975	16,206,979	697	0.467	51,572	23,252	
7	21,903,355	144,231	861	0.512	25,439	167.516	
8	13,909,212	3,840,947	481	0.554	28,917	7,985.337	
9	12,524,734	4,853,703	456	0.624	27,466	10,644	

#	% band purity	% lane purity	Rolling Bg. Corr. Vol.	Rolling Bg. Corr. Den.	Mol. Wt.	Rel. Quant. (w/ LB Corr. Vol.)
1	2.996	2.094	1,793,536	7,320.555	144.643	1.424
2	2.44	1.706	1,460,736	3,381.333	123.214	0.568
3	15.309	10.702	9,164,800	24,117	95.833	7.153
4	17.43	12.184	10,434,048	24,961	70.763	7.943
5	16.279	11.38	9,745,408	16,658	62.288	7.483
6	28.608	19.998	17,125,888	24,570	46.829	13.812
7	2.654	1.855	1,588,992	1,845.519	40.805	0.123

#	% band purity	% lane purity	Rolling Bg. Corr. Vol.	Rolling Bg. Corr. Den.	Mol. Wt.	Rel. Quant. (w/ LB Corr. Vol.)
8	6.358	4.444	3,806,208	7,913.114	35.531	3.273
9	7.925	5.54	4,744,448	10,404	28.184	4.136

Lane 4 - 2uM Thapsigargin

#	Vol. (Int.)	Local Bg. Corr. Vol.	Area	Rf	Density	Local Bg. Corr. Den.	[redacted]
1	6,909,854	1,096,447	288	0.202	23,992	3,807.11	[redacted]
2	17,187,489	190,786	714	0.232	24,072	267.208	[redacted]
3	17,967,905	8,124,533	380	0.268	47,283	21,380	[redacted]
4	20,431,348	8,232,621	418	0.326	48,878	19,695	[redacted]
5	28,479,563	7,946,461	680	0.371	41,881	11,685	[redacted]
6	34,155,081	14,940,313	656	0.462	52,065	22,774	[redacted]
7	22,148,931	245,782	882	0.512	25,112	278.665	[redacted]
8	13,449,543	2,497,400	507	0.552	26,527	4,925.838	[redacted]
9	14,020,679	4,010,701	520	0.624	26,962	7,712.888	[redacted]

#	% band purity	% lane purity	Rolling Bg. Corr. Vol.	Rolling Bg. Corr. Den.	Mol. Wt.	Rel. Quant. (w/ LB Corr. Vol.)
1	1.931	1.425	1,034,752	3,592.889	146.429	0.934
2	2.798	2.065	1,499,136	2,099.63	123.214	0.163
3	16.472	12.157	8,825,344	23,224	97.222	6.924
4	17.344	12.801	9,292,544	22,230	71.186	7.016
5	17.108	12.626	9,165,824	13,479	63.136	6.772
6	30.116	22.227	16,135,424	24,596	47.463	12.733
7	1.987	1.467	1,064,704	1,207.147	40.805	0.209
8	4.451	3.285	2,384,640	4,703.432	35.776	2.128
9	7.792	5.751	4,174,848	8,028.554	28.184	3.418

Lane 5 - t = 0

#	Vol. (Int.)	Local Bg. Corr. Vol.	Area	Rf	Density	Local Bg. Corr. Den.	[redacted]
1	7,147,365	1,764,534	288	0.2	24,817	6,126.855	[redacted]
2	16,571,067	483,061	731	0.228	22,669	660.823	[redacted]
3	20,835,355	8,825,388	480	0.268	43,406	18,386	[redacted]
4	18,679,325	7,634,334	360	0.319	51,887	21,206	[redacted]
5	47,435,856	5,101,118	1,344	0.364	35,294	3,795.475	[redacted]

#	Vol. (Int.)	Local Bg. Corr. Vol.	Area	Rf	Density	Local Bg. Corr. Den.	[redacted]
6	31,575,000	15,445,565	688	0.455	45,893	22,449	[redacted]
7	22,861,383	87,951	925	0.502	24,715	95.082	[redacted]
8	18,194,147	4,651,759	697	0.549	26,103	6,673.974	[redacted]
9	12,910,870	4,326,973	560	0.62	23,055	7,726.738	[redacted]

#	% band purity	% lane purity	Rolling Bg. Corr. Vol.	Rolling Bg. Corr. Den.	Mol. Wt.	Rel. Quant. (w/ LB Corr. Vol.)
1	2.736	2.348	1,774,080	6,160	148.214	1.504
2	2.794	2.398	1,812,224	2,479.103	126.786	0.412
3	14.424	12.38	9,354,240	19,488	97.222	7.521
4	14.752	12.662	9,567,488	26,576	72.458	6.506
5	21.914	18.809	14,212,352	10,574	64.407	4.347
6	25.337	21.747	16,432,127	23,883	48.415	13.163
7	4.533	3.891	2,939,904	3,178.275	42.073	0.075
8	6.583	5.65	4,269,056	6,124.901	36.02	3.964
9	6.927	5.945	4,492,288	8,021.943	28.673	3.688

iBright™ Image Analysis Report

Katarina+ Chang

19 November 2022

pCofilin CHEMI_01272022_142348

Date:	27 January 2022 02:23:48PM
Mode:	Chemi Blots
Notes:	
Model:	FL1500
Instrument name:	2462619090234
Serial No:	2462619090234
Firmware version:	1.6.0
iBA version:	5.0
Image size:	676px X 540px
Image area:	118.63mm X 94.91mm
Optical Zoom:	1.9x
Digital Zoom:	1x
Focus level:	430
Resolution:	5 x 5
Exposure time:	60748 ms
Exposure mode:	Normal

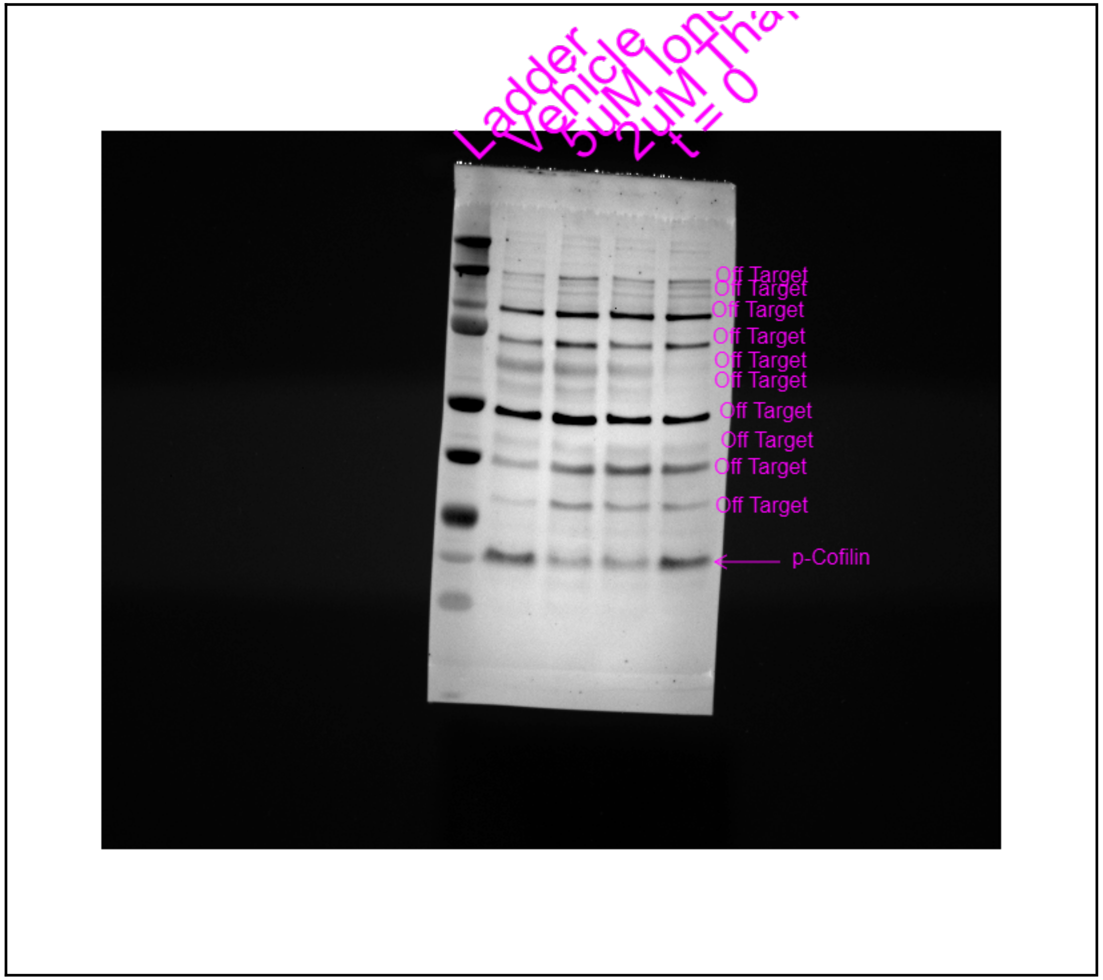

pCofilin CHEMI_01272022_142348

Date:	27 January 2022 02:23:48PM
Mode:	Chemi Blots
Notes:	
Model:	FL1500
Instrument name:	2462619090234
Serial No:	2462619090234
Firmware version:	1.6.0
iBA version:	5.0
Image size:	676px X 540px
Image area:	118.63mm X 94.91mm
Optical Zoom:	1.9x
Digital Zoom:	1x
Focus level:	430
Resolution:	5 x 5
Exposure time:	60748 ms
Exposure mode:	Normal

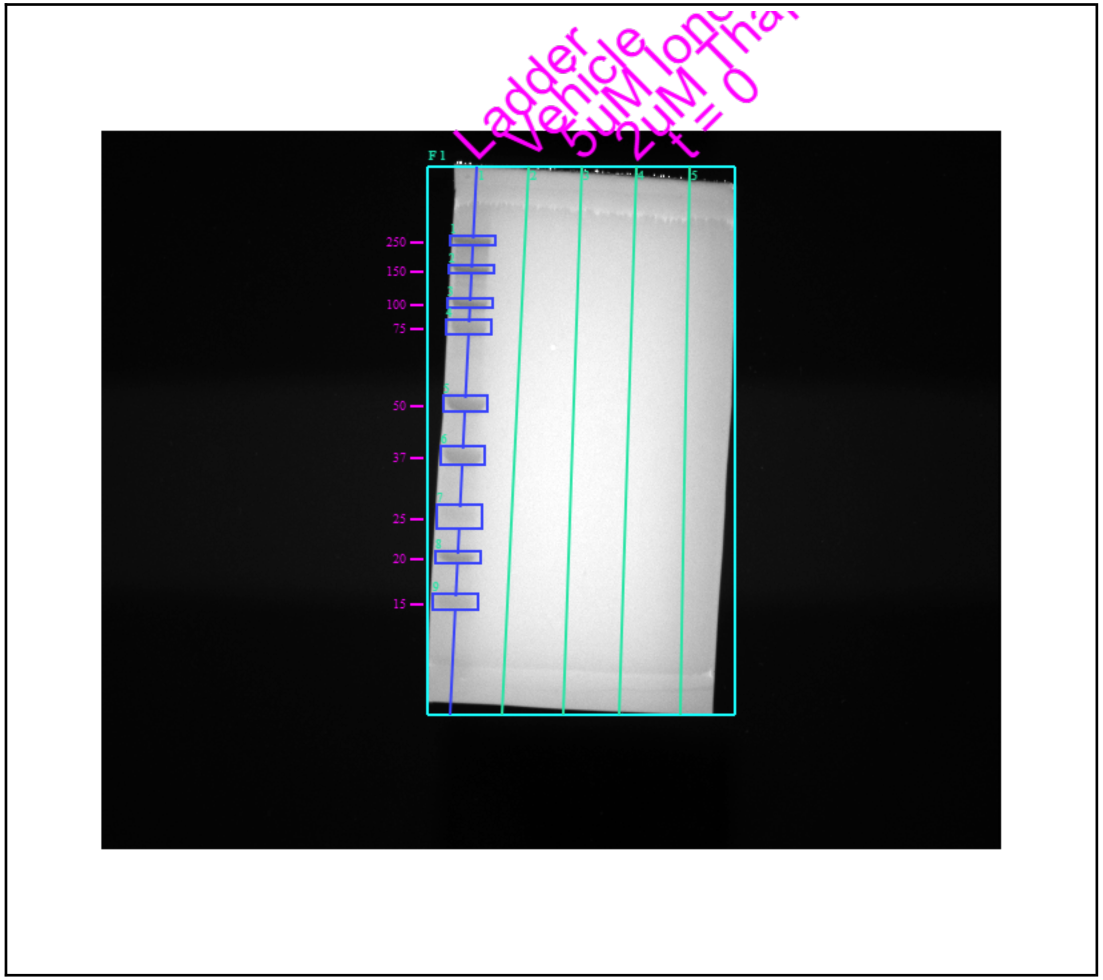

pCofilin CHEMI_01272022_142348

Date:	27 January 2022 02:23:48PM
Mode:	Chemi Blots
Notes:	
Model:	FL1500
Instrument name:	2462619090234
Serial No:	2462619090234
Firmware version:	1.6.0
iBA version:	5.0
Image size:	676px X 540px
Image area:	118.63mm X 94.91mm
Optical Zoom:	1.9x
Digital Zoom:	1x
Focus level:	430
Resolution:	5 x 5
Exposure time:	60748 ms
Exposure mode:	Normal

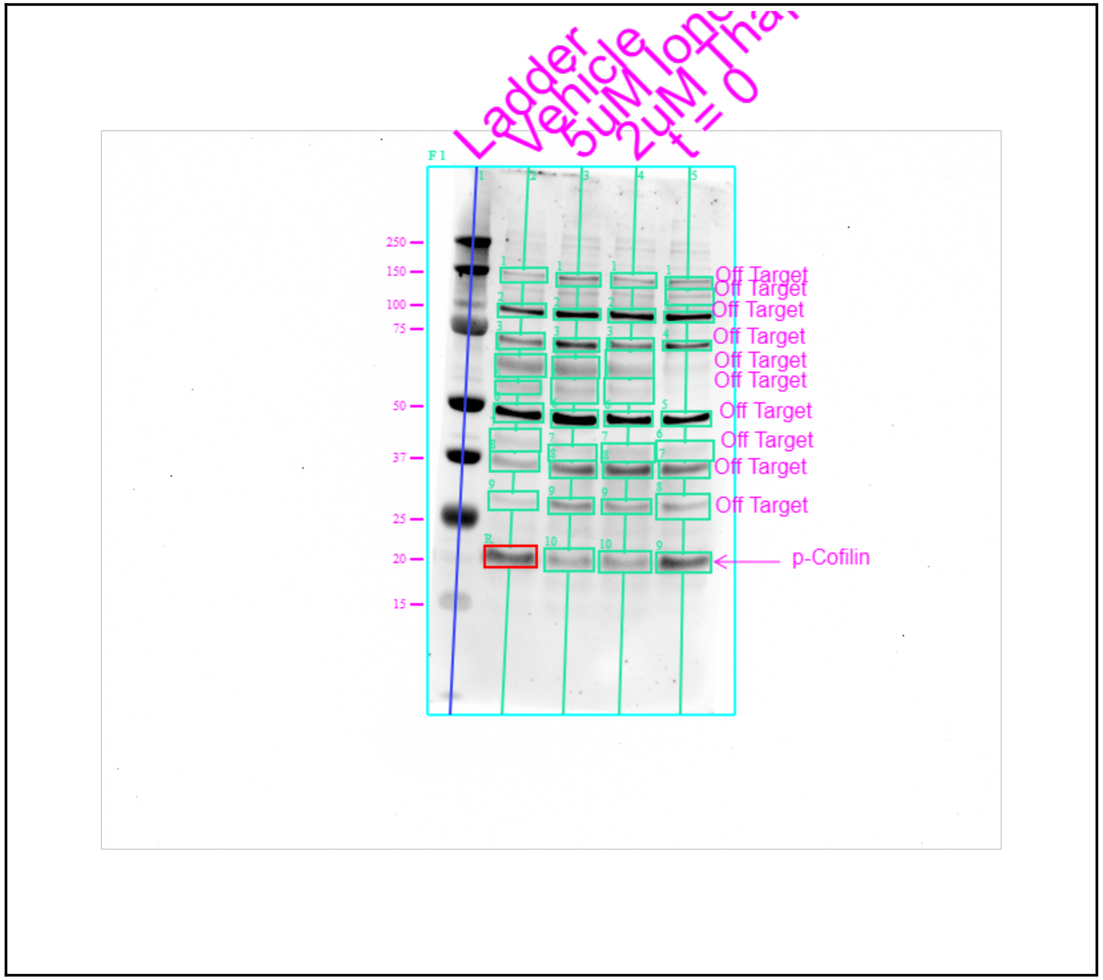

LANE AND BAND ANALYSIS DATA TABLE

pCofilin CHEMI_01272022_142348

Frame: 1
Channel: Membrane
Sensitivity: 100
Molecular Weight Analysis Regression Method : Point to Point

Lane 1 - Ladder

#	Vol. (Int.)	Local Bg. Corr. Vol.	Area	Rf	Density	Local Bg. Corr. Den.	% band purity	% lane purity	Rolling Bg. Corr. Vol.	Rolling Bg. Corr. Den.	Mol. Wt.
1	10,553,341	588,638	280	0.133	37,690	2,102.281	11.775	2.178	1,916,160	6,843.429	250
2	9,106,102	625,292	245	0.187	37,167	2,552.213	9.821	1.816	1,598,208	6,523.298	150
3	9,841,287	572,122	280	0.248	35,147	2,043.295	10.183	1.883	1,657,088	5,918.171	100
4	13,675,785	243,318	420	0.291	32,561	579.331	11.382	2.105	1,852,160	4,409.905	75
5	14,225,481	503,466	442	0.432	32,184	1,139.064	16.584	3.067	2,698,752	6,105.774	50
6	15,403,573	481,914	510	0.527	30,203	944.931	13.798	2.552	2,245,376	4,402.698	37
7	18,076,302	NA	665	0.638	27,182	NA	6.664	1.232	1,084,416	1,630.701	25
8	10,869,858	838,776	350	0.711	31,056	2,396.503	10.883	2.013	1,771,008	5,060.023	20
9	13,691,204	157,956	455	0.794	30,090	347.158	8.912	1.648	1,450,240	3,187.341	15

Frame: 1
Channel: Chemi
Sensitivity: 100
Molecular Weight Analysis Regression Method : Point to Point

Lane 2 - Vehicle

#	Vol. (Int.)	Local Bg. Corr. Vol.	Area	Rf	Density	Local Bg. Corr. Den.
1	5,363,839	1,548,398	432	0.197	12,416	3,584.255
2	10,468,728	6,994,118	407	0.262	25,721	17,184
3	8,915,187	4,886,165	481	0.318	18,534	10,158
4	12,940,680	5,883,845	702	0.362	18,434	8,381.547
5	5,635,729	1,760,826	385	0.403	14,638	4,573.575
6	18,212,860	13,334,861	570	0.449	31,952	23,394
7	6,846,031	1,242,830	702	0.498	9,752.181	1,770.414
8	7,860,551	3,368,190	608	0.536	12,928	5,539.787

#	Vol. (Int.)	Local Bg. Corr. Vol.	Area	Rf	Density	Local Bg. Corr. Den.
9	5,345,200	1,429,604	570	0.609	9,377.544	2,508.078
10	17,058,969	10,448,461	680	0.711	25,086	15,365

#	% band purity	% lane purity	Rolling Bg. Corr. Vol.	Rolling Bg. Corr. Den.	Mol. Wt.	Rel. Quant. (w/ LB Corr. Vol.)
1	2.719	2.271	1,409,536	3,262.815	142	0.148
2	13.272	11.085	6,879,232	16,902	91.667	0.669
3	9.684	8.088	5,019,136	10,434	70.259	0.468
4	12.661	10.575	6,562,560	9,348.376	62.5	0.563
5	3.398	2.838	1,761,280	4,574.753	55.172	0.169
6	24.751	20.673	12,828,928	22,506	47.667	1.276
7	2.06	1.721	1,067,776	1,521.048	41	0.119
8	5.84	4.878	3,026,944	4,978.526	35.957	0.322
9	2.247	1.877	1,164,544	2,043.06	28.13	0.137
10	23.368	19.518	12,111,872	17,811	20	1

Lane 3 - 5uM Ionomycin

#	Vol. (Int.)	Local Bg. Corr. Vol.	Area	Rf	Density	Local Bg. Corr. Den.
1	6,916,442	3,200,298	374	0.206	18,493	8,556.947
2	12,209,797	8,765,495	324	0.269	37,684	27,053
3	10,953,129	7,408,073	385	0.325	28,449	19,241
4	11,537,546	5,170,315	612	0.367	18,852	8,448.228
5	9,396,954	1,873,901	720	0.408	13,051	2,602.641
6	19,394,678	15,545,830	504	0.459	38,481	30,844
7	4,945,239	193,783	481	0.522	10,281	402.876
8	11,268,909	6,662,204	504	0.551	22,358	13,218
9	8,145,949	3,944,617	455	0.619	17,903	8,669.489
10	10,367,752	4,585,267	684	0.716	15,157	6,703.608

#	% band purity	% lane purity	Rolling Bg. Corr. Vol.	Rolling Bg. Corr. Den.	Mol. Wt.	Rel. Quant. (w/ LB Corr. Vol.)
1	6.254	4.936	3,845,120	10,281	134	0.306
2	15.283	12.062	9,395,968	28,999	87.5	0.839
3	12.206	9.634	7,504,128	19,491	68.966	0.709
4	9.694	7.651	5,959,680	9,738.039	61.638	0.495

#	% band purity	% lane purity	Rolling Bg. Corr. Vol.	Rolling Bg. Corr. Den.	Mol. Wt.	Rel. Quant. (w/ LB Corr. Vol.)
5	4.712	3.719	2,896,896	4,023.467	54.31	0.179
6	24.95	19.692	15,339,520	30,435	46.333	1.488
7	1.89	1.492	1,161,984	2,415.767	37.667	0.019
8	10.786	8.513	6,631,168	13,157	34.391	0.638
9	5.559	4.387	3,417,600	7,511.209	27.087	0.378
10	8.666	6.84	5,328,128	7,789.661	19.706	0.439

Lane 4 - 2uM Thapsigargin

#	Vol. (Int.)	Local Bg. Corr. Vol.	Area	Rf	Density	Local Bg. Corr. Den.
1	5,819,315	2,131,929	420	0.206	13,855	5,076.023
2	13,266,178	9,234,640	396	0.272	33,500	23,319
3	7,392,590	4,537,163	360	0.323	20,534	12,603
4	10,500,189	1,699,014	814	0.359	12,899	2,087.241
5	7,078,569	1,181,801	760	0.408	9,313.907	1,555.002
6	14,867,263	12,359,064	444	0.459	33,484	27,835
7	6,947,215	335,881	615	0.522	11,296	546.148
8	13,463,688	8,124,787	494	0.553	27,254	16,446
9	7,510,254	2,930,022	456	0.619	16,469	6,425.487
10	10,944,933	4,562,792	680	0.721	16,095	6,709.989

#	% band purity	% lane purity	Rolling Bg. Corr. Vol.	Rolling Bg. Corr. Den.	Mol. Wt.	Rel. Quant. (w/ LB Corr. Vol.)
1	5.144	4.168	2,738,944	6,521.295	134	0.204
2	19.002	15.395	10,117,120	25,548	86.111	0.884
3	8.131	6.587	4,328,960	12,024	69.397	0.434
4	6.592	5.341	3,509,760	4,311.744	62.931	0.163
5	2.396	1.941	1,275,648	1,678.484	54.31	0.113
6	22.908	18.559	12,196,608	27,469	46.333	1.183
7	4.294	3.479	2,286,336	3,717.62	37.667	0.032
8	16.326	13.227	8,692,480	17,596	34.13	0.778
9	4.864	3.941	2,589,696	5,679.158	27.087	0.28
10	10.343	8.38	5,506,816	8,098.259	19.412	0.437

Lane 5 - t = 0

#	Vol. (Int.)	Local Bg. Corr. Vol.	Area	Rf	Density	Local Bg. Corr. Den.
1	5,858,235	2,984,256	360	0.211	16,272	8,289.602
2	5,830,562	782,151	481	0.238	12,121	1,626.095
3	11,822,971	8,922,072	342	0.274	34,570	26,087
4	7,588,167	5,654,235	296	0.325	25,635	19,102
5	13,717,709	12,094,400	456	0.459	30,082	26,522
6	5,335,514	188,950	704	0.517	7,578.855	268.396
7	10,857,265	6,856,806	546	0.551	19,885	12,558
8	9,083,501	3,192,139	820	0.619	11,077	3,892.853
9	15,799,612	11,037,712	672	0.721	23,511	16,425

#	% band purity	% lane purity	Rolling Bg. Corr. Vol.	Rolling Bg. Corr. Den.	Mol. Wt.	Rel. Quant. (w/ LB Corr. Vol.)
1	6.237	5.271	3,546,880	9,852.444	130	0.286
2	4.697	3.97	2,671,104	5,553.231	108	0.075
3	16.74	14.149	9,520,384	27,837	84.722	0.854
4	9.937	8.399	5,651,456	19,092	68.966	0.541
5	21.285	17.989	12,104,704	26,545	46.333	1.158
6	3.021	2.553	1,718,016	2,440.364	38.333	0.018
7	12.653	10.694	7,196,160	13,179	34.391	0.656
8	4.87	4.116	2,769,664	3,377.639	27.087	0.306
9	20.56	17.377	11,692,544	17,399	19.412	1.056

iBright™ Image Analysis Report

Katarina+ Chang

19 November 2022

Alpha Tubulin CHEMI_01282022_123632

Date:	28 January 2022 12:36:32PM
Mode:	Chemi Blots
Notes:	
Model:	FL1500
Instrument name:	2462619090234
Serial No:	2462619090234
Firmware version:	1.6.0
iBA version:	5.0
Image size:	563px X 450px
Image area:	112.7mm X 90.16mm
Optical Zoom:	2x
Digital Zoom:	1.2x
Focus level:	455
Resolution:	5 x 5
Exposure time:	1877 ms
Exposure mode:	Normal

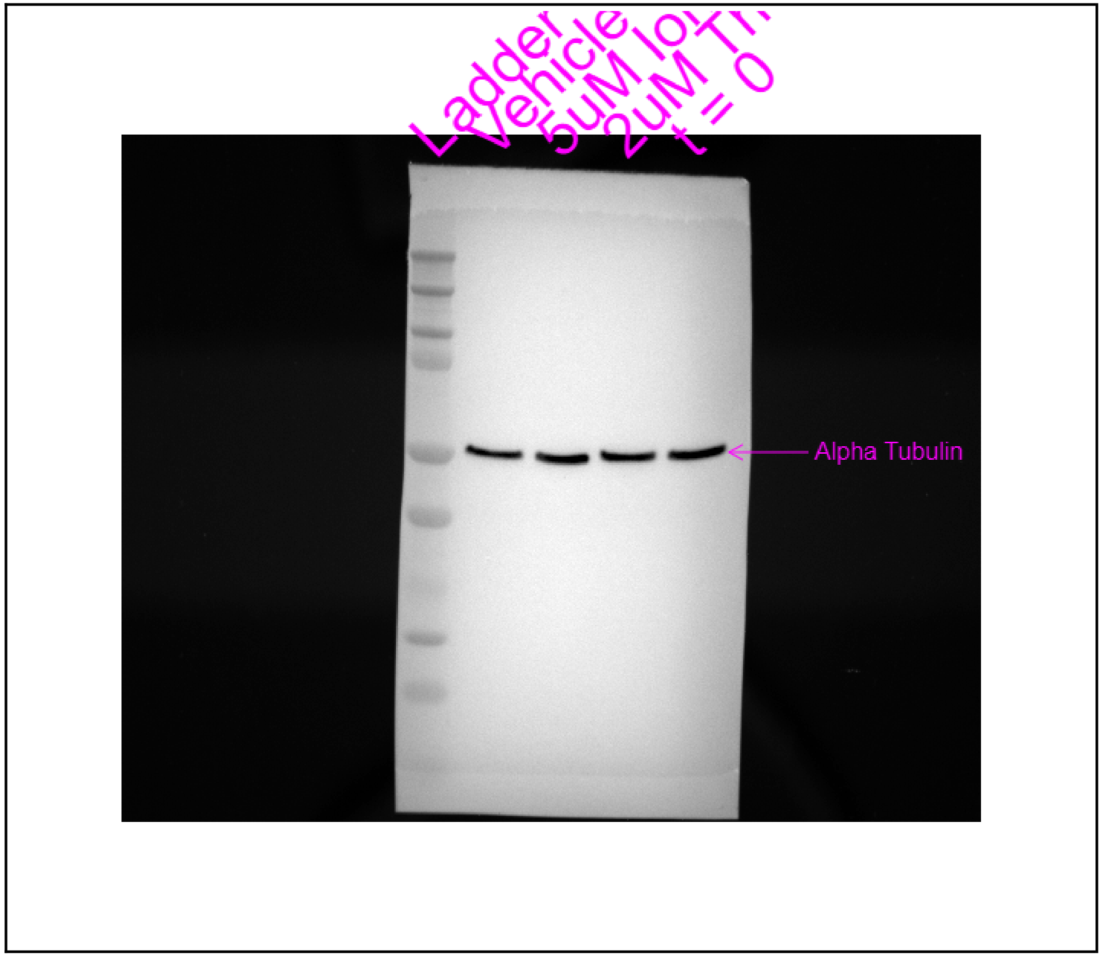

Alpha Tubulin CHEMI_01282022_123632

Date:	28 January 2022 12:36:32PM
Mode:	Chemi Blots
Notes:	
Model:	FL1500
Instrument name:	2462619090234
Serial No:	2462619090234
Firmware version:	1.6.0
iBA version:	5.0
Image size:	563px X 450px
Image area:	112.7mm X 90.16mm
Optical Zoom:	2x
Digital Zoom:	1.2x
Focus level:	455
Resolution:	5 x 5
Exposure time:	1877 ms
Exposure mode:	Normal

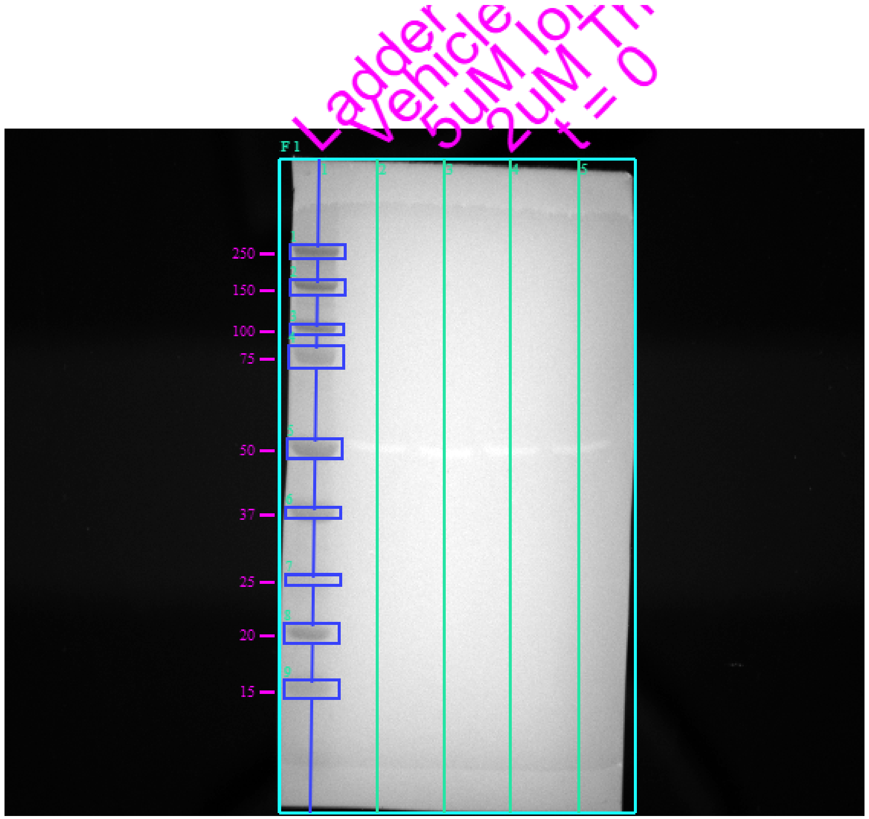

Alpha Tubulin CHEMI_01282022_123632

Date:	28 January 2022 12:36:32PM
Mode:	Chemi Blots
Notes:	
Model:	FL1500
Instrument name:	2462619090234
Serial No:	2462619090234
Firmware version:	1.6.0
iBA version:	5.0
Image size:	563px X 450px
Image area:	112.7mm X 90.16mm
Optical Zoom:	2x
Digital Zoom:	1.2x
Focus level:	455
Resolution:	5 x 5
Exposure time:	1877 ms
Exposure mode:	Normal

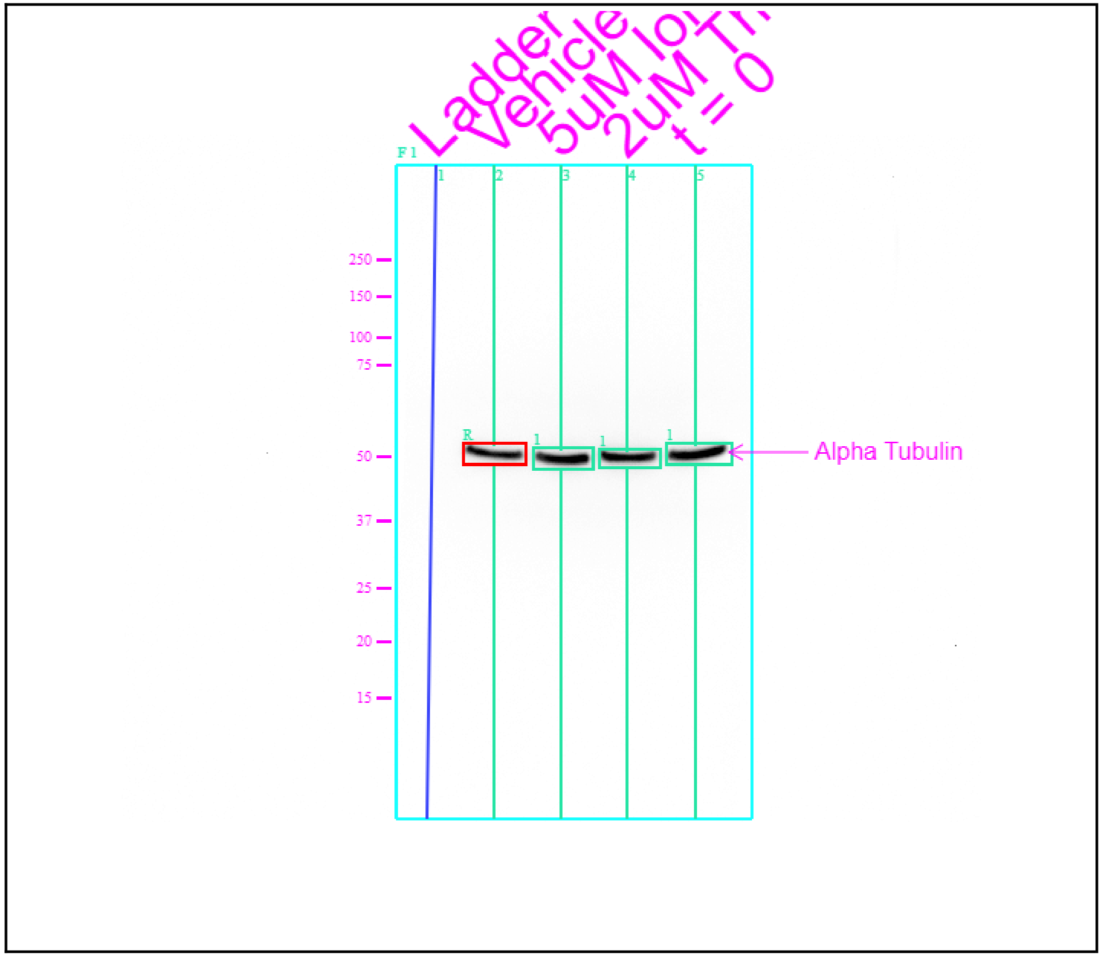

LANE AND BAND ANALYSIS DATA TABLE

Alpha Tubulin CHEMI_01282022_123632

Frame: 1
Channel: Membrane
Sensitivity: 100
Molecular Weight Analysis Regression Method : Point to Point

Lane 1 - Ladder

#	Vol. (Int.)	Local Bg. Corr. Vol.	Area	Rf	Density	Local Bg. Corr. Den.	% band purity	% lane purity	Rolling Bg. Corr. Vol.	Rolling Bg. Corr. Den.	Mol. Wt.
1	13,549,890	598,124	370	0.14	36,621	1,616.552	13.553	3.261	2,128,896	5,753.773	250
2	14,200,358	672,223	407	0.196	34,890	1,651.654	12.541	3.018	1,969,920	4,840.098	150
3	9,728,104	487,353	288	0.259	33,778	1,692.2	9.242	2.224	1,451,776	5,040.889	100
4	18,389,356	145,815	592	0.301	31,063	246.309	12.316	2.964	1,934,592	3,267.892	75
5	16,694,825	486,885	518	0.442	32,229	939.934	17.982	4.327	2,824,704	5,453.097	50
6	9,411,690	313,971	296	0.54	31,796	1,060.715	9.829	2.365	1,543,936	5,216	37
7	8,428,243	66,333	296	0.643	28,473	224.098	3.563	0.857	559,616	1,890.595	25
8	16,110,012	880,131	518	0.724	31,100	1,699.095	12.388	2.981	1,945,856	3,756.479	20
9	15,142,502	596,011	481	0.811	31,481	1,239.109	8.587	2.066	1,348,864	2,804.291	15

Frame: 1
Channel: Chemi
Sensitivity: 100
Molecular Weight Analysis Regression Method : Point to Point

Lane 2 - Vehicle

#	Vol. (Int.)	Local Bg. Corr. Vol.	Area	Rf	Density	Local Bg. Corr. Den.
1	8,784,452	7,665,809	630	0.442	13,943	12,167

#	% band purity	% lane purity	Rolling Bg. Corr. Vol.	Rolling Bg. Corr. Den.	Mol. Wt.	Rel. Quant. (w/ LB Corr. Vol.)
1	100	95.546	7,979,264	12,665	50	1

Lane 3 - 5uM Ionomycin

#	Vol. (Int.)	Local Bg. Corr. Vol.	Area	Rf	Density	Local Bg. Corr. Den.
1	10,272,212	8,667,490	600	0.449	17,120	14,445

#	% band purity	% lane purity	Rolling Bg. Corr. Vol.	Rolling Bg. Corr. Den.	Mol. Wt.	Rel. Quant. (w/ LB Corr. Vol.)

#	% band purity	% lane purity	Rolling Bg. Corr. Vol.	Rolling Bg. Corr. Den.	Mol. Wt.	Rel. Quant. (w/ LB Corr. Vol.)
1	100	93.276	9,350,400	15,584	49.071	1.131

Lane 4 - 2uM Thapsigargin

#	Vol. (Int.)	Local Bg. Corr. Vol.	Area	Rf	Density	Local Bg. Corr. Den.	
1	9,609,192	8,045,970	533	0.449	18,028	15,095	

#	% band purity	% lane purity	Rolling Bg. Corr. Vol.	Rolling Bg. Corr. Den.	Mol. Wt.	Rel. Quant. (w/ LB Corr. Vol.)
1	100	90.476	8,696,320	16,315	49.071	1.05

Lane 5 - t = 0

#	Vol. (Int.)	Local Bg. Corr. Vol.	Area	Rf	Density	Local Bg. Corr. Den.	
1	11,330,141	10,488,307	660	0.442	17,166	15,891	

#	% band purity	% lane purity	Rolling Bg. Corr. Vol.	Rolling Bg. Corr. Den.	Mol. Wt.	Rel. Quant. (w/ LB Corr. Vol.)
1	100	97.003	10,621,440	16,093	50	1.368

iBright™ Image Analysis Report

Katarina+ Chang

19 November 2022

detyrosinated tubulin CHEMI_01262022_122824

Date:	26 January 2022 12:28:24PM
Mode:	Chemi Blots
Notes:	
Model:	FL1500
Instrument name:	2462619090234
Serial No:	2462619090234
Firmware version:	1.6.0
iBA version:	5.0
Image size:	615px X 491px
Image area:	112.7mm X 90.16mm
Optical Zoom:	2x
Digital Zoom:	1.1x
Focus level:	455
Resolution:	5 x 5
Exposure time:	54160 ms
Exposure mode:	Normal

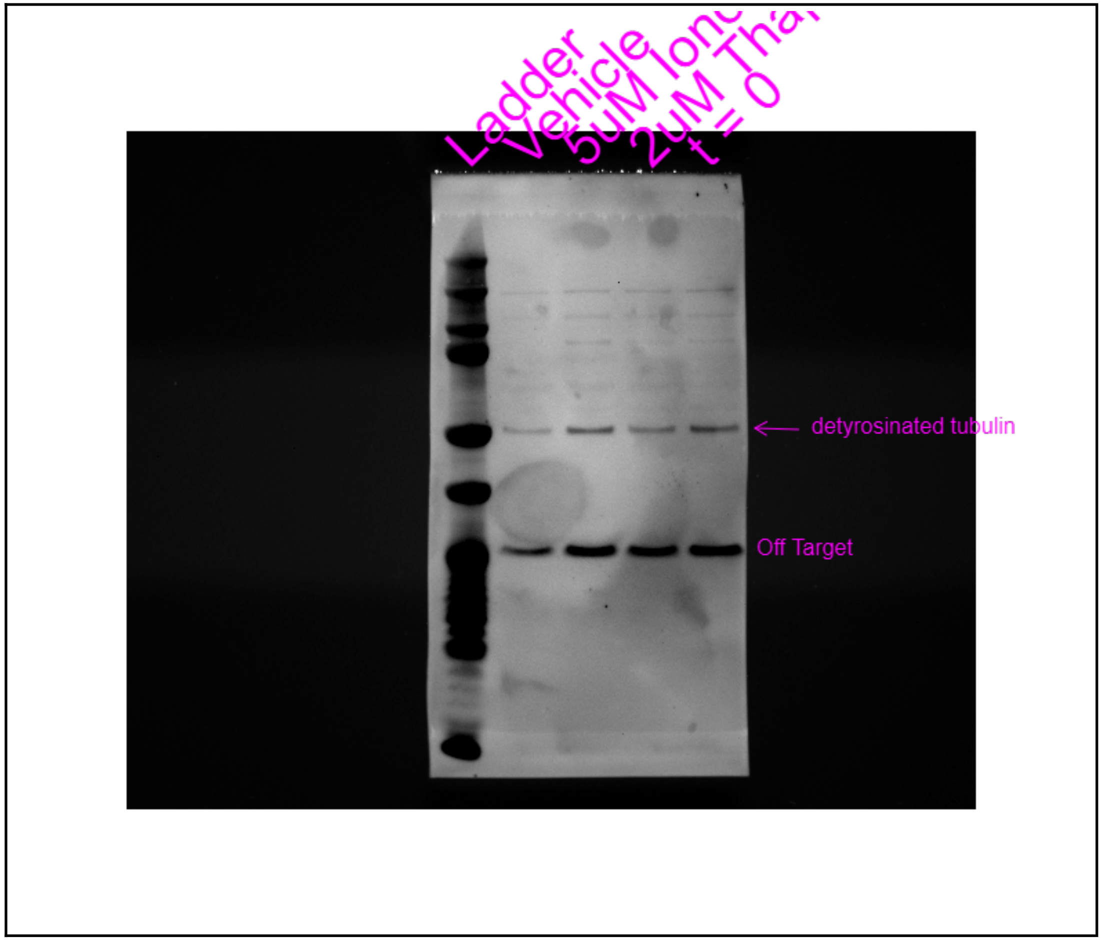

detyrosinated tubulin CHEMI_01262022_122824

Date: 26 January 2022 12:28:24PM
Mode: Chemi Blots
Notes:
Model: FL1500
Instrument name: 2462619090234
Serial No: 2462619090234
Firmware version: 1.6.0
iBA version: 5.0
Image size: 615px X 491px
Image area: 112.7mm X 90.16mm
Optical Zoom: 2x
Digital Zoom: 1.1x
Focus level: 455
Resolution: 5 x 5
Exposure time: 54160 ms
Exposure mode: Normal

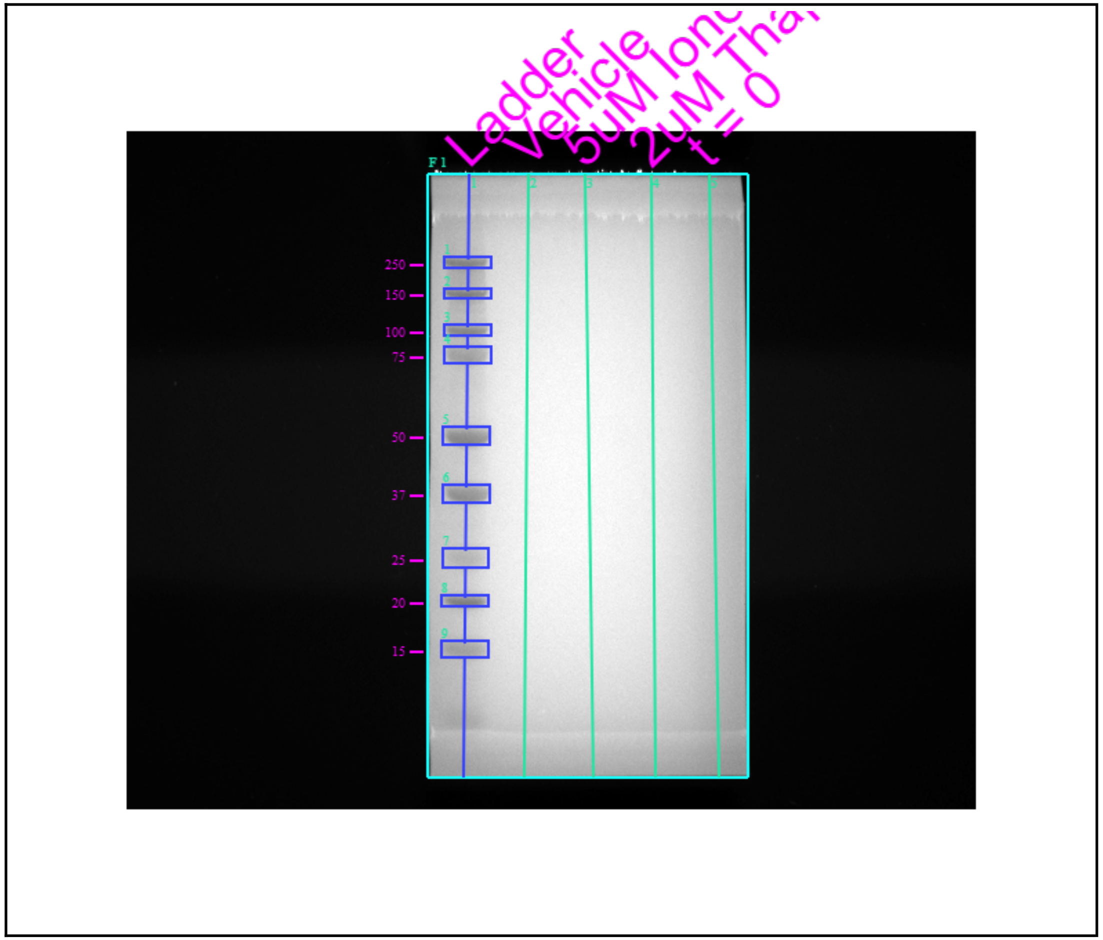

detyrosinated tubulin CHEMI_01262022_122824

Date:	26 January 2022 12:28:24PM
Mode:	Chemi Blots
Notes:	
Model:	FL1500
Instrument name:	2462619090234
Serial No:	2462619090234
Firmware version:	1.6.0
iBA version:	5.0
Image size:	615px X 491px
Image area:	112.7mm X 90.16mm
Optical Zoom:	2x
Digital Zoom:	1.1x
Focus level:	455
Resolution:	5 x 5
Exposure time:	54160 ms
Exposure mode:	Normal

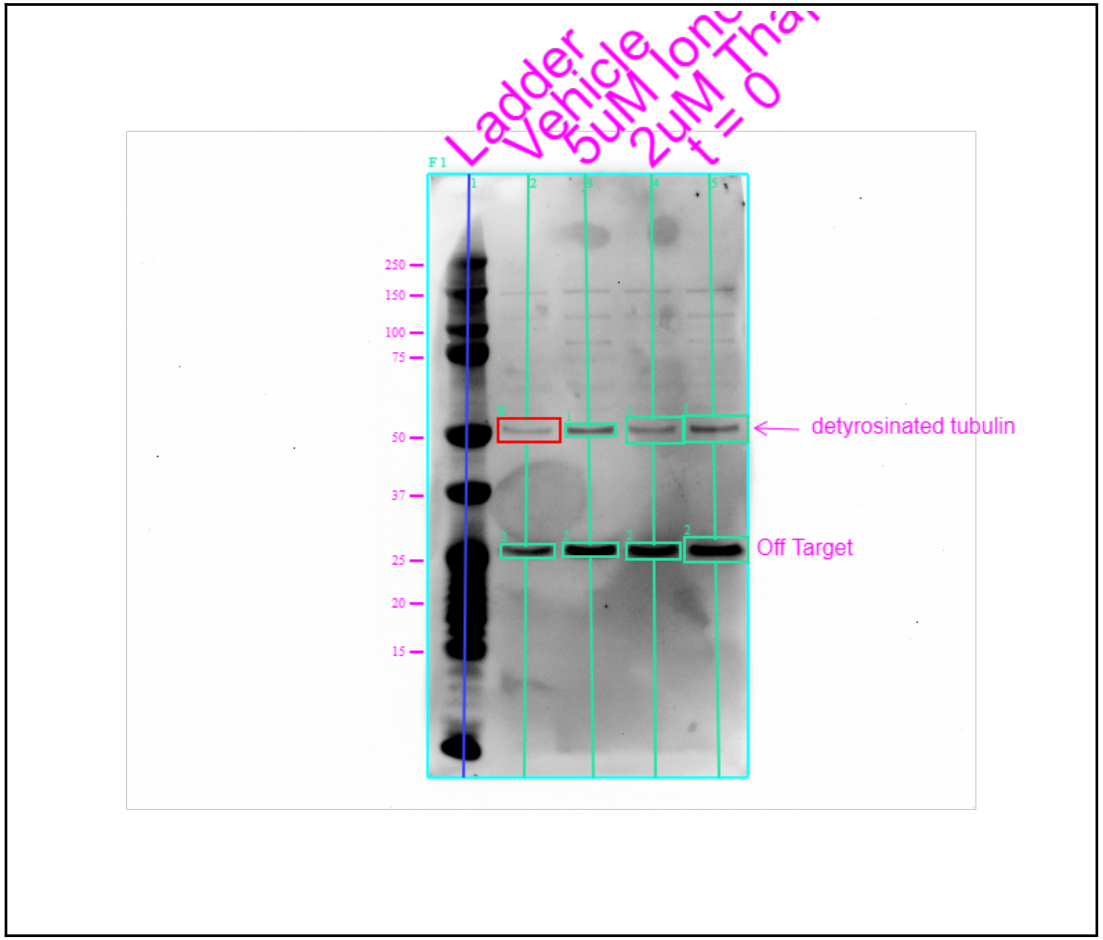

LANE AND BAND ANALYSIS DATA TABLE

detyrosinated tubulin CHEMI_01262022_122824

Frame: 1
Channel: Membrane
Sensitivity: 100
Molecular Weight Analysis Regression Method : Point to Point

Lane 1 - Ladder

#	Vol. (Int.)	Local Bg. Corr. Vol.	Area	Rf	Density	Local Bg. Corr. Den.	% band purity	% lane purity	Rolling Bg. Corr. Vol.	Rolling Bg. Corr. Den.	Mol. Wt.
1	11,122,192	1,972,036	315	0.146	35,308	6,260.434	8.852	2.473	1,893,632	6,011.53	250
2	9,885,985	1,912,376	280	0.197	35,307	6,829.918	7.941	2.219	1,698,816	6,067.2	150
3	10,638,971	2,054,613	315	0.259	33,774	6,522.583	8.474	2.368	1,812,736	5,754.717	100
4	14,251,340	2,238,974	455	0.3	31,321	4,920.822	9.005	2.516	1,926,400	4,233.846	75
5	16,587,569	3,849,179	490	0.432	33,852	7,855.469	19.054	5.324	4,076,032	8,318.433	50
6	16,045,753	3,313,571	490	0.529	32,746	6,762.39	16.329	4.563	3,493,120	7,128.816	37
7	14,708,132	1,571,492	510	0.636	28,839	3,081.358	7.641	2.135	1,634,560	3,205.02	25
8	11,017,903	2,506,592	315	0.707	34,977	7,957.435	12.845	3.589	2,747,904	8,723.505	20
9	14,527,954	1,858,777	455	0.787	31,929	4,085.226	9.858	2.755	2,108,928	4,635.007	15

Frame: 1
Channel: Chemi
Sensitivity: 100
Molecular Weight Analysis Regression Method : Point to Point

Lane 2 - Vehicle

#	Vol. (Int.)	Local Bg. Corr. Vol.	Area	Rf	Density	Local Bg. Corr. Den.	[redacted]
1	17,990,087	1,382,498	828	0.423	21,727	1,669.684	[redacted]
2	18,678,541	6,178,743	440	0.625	42,451	14,042	[redacted]

#	% band purity	% lane purity	Rolling Bg. Corr. Vol.	Rolling Bg. Corr. Den.	Mol. Wt.	Rel. Quant. (w/ LB Corr. Vol.)
1	18.949	4.012	1,752,832	2,116.947	51.724	1
2	81.051	17.161	7,497,472	17,039	26.277	4.469

Lane 3 - 5uM Ionomycin

#	Vol. (Int.)	Local Bg. Corr. Vol.	Area	Rf	Density	Local Bg. Corr. Den.	[redacted]

#	Vol. (Int.)	Local Bg. Corr. Vol.	Area	Rf	Density	Local Bg. Corr. Den.
1	11,001,569	3,876,129	370	0.423	29,733	10,476
2	24,030,066	10,441,697	451	0.622	53,281	23,152

#	% band purity	% lane purity	Rolling Bg. Corr. Vol.	Rolling Bg. Corr. Den.	Mol. Wt.	Rel. Quant. (w/ LB Corr. Vol.)
1	23.349	7.196	3,959,808	10,702	51.724	2.804
2	76.651	23.624	12,999,680	28,824	26.532	7.553

Lane 4 - 2uM Thapsigargin

#	Vol. (Int.)	Local Bg. Corr. Vol.	Area	Rf	Density	Local Bg. Corr. Den.
1	25,322,468	3,203,034	860	0.423	29,444	3,724.459
2	27,743,623	8,145,017	520	0.625	53,353	15,663

#	% band purity	% lane purity	Rolling Bg. Corr. Vol.	Rolling Bg. Corr. Den.	Mol. Wt.	Rel. Quant. (w/ LB Corr. Vol.)
1	25.402	6.306	3,345,920	3,890.605	51.724	2.317
2	74.598	18.52	9,826,048	18,896	26.277	5.892

Lane 5 - t = 0

#	Vol. (Int.)	Local Bg. Corr. Vol.	Area	Rf	Density	Local Bg. Corr. Den.
1	27,775,168	7,393,834	960	0.421	28,932	7,701.911
2	39,077,572	16,318,259	893	0.622	43,759	18,273

#	% band purity	% lane purity	Rolling Bg. Corr. Vol.	Rolling Bg. Corr. Den.	Mol. Wt.	Rel. Quant. (w/ LB Corr. Vol.)
1	23.324	7.862	4,378,368	4,560.8	52.155	5.348
2	76.676	25.846	14,393,344	16,117	26.532	11.803

iBright™ Image Analysis Report

Katarina+ Chang

19 November 2022

GAPDH_CHEMI_01282022_123954

Date:	28 January 2022 12:39:54PM
Mode:	Chemi Blots
Notes:	
Model:	FL1500
Instrument name:	2462619090234
Serial No:	2462619090234
Firmware version:	1.6.0
iBA version:	5.0
Image size:	676px X 540px
Image area:	118.63mm X 94.91mm
Optical Zoom:	1.9x
Digital Zoom:	1x
Focus level:	430
Resolution:	5 x 5
Exposure time:	3916 ms
Exposure mode:	Normal

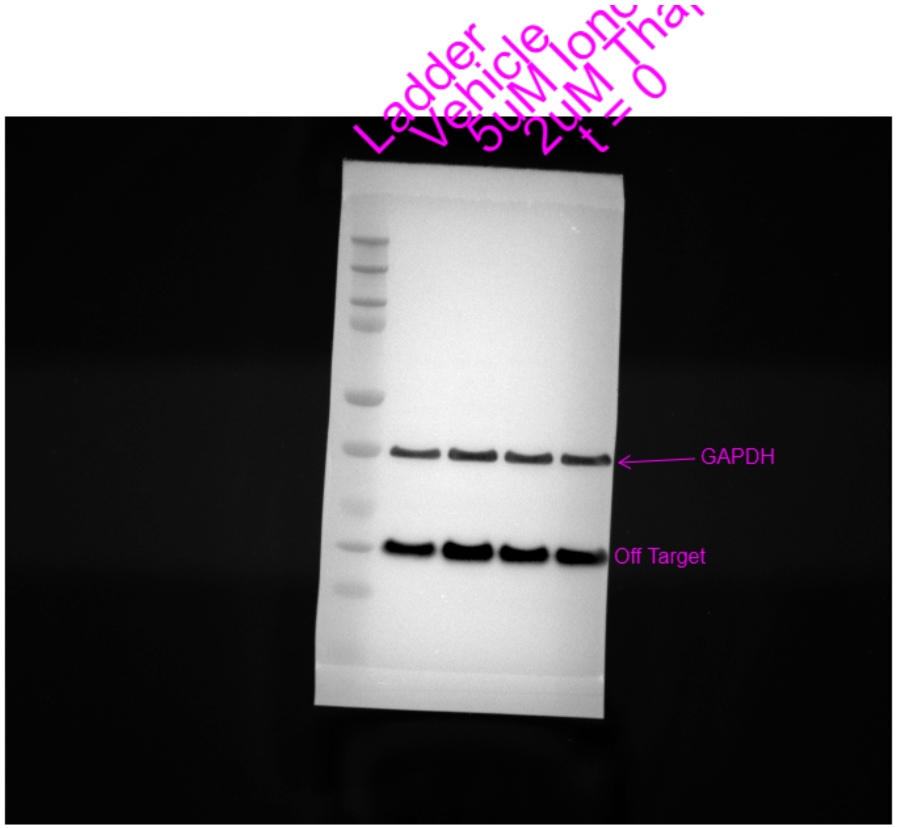

GAPDH_CHEMI_01282022_123954

Date:	28 January 2022 12:39:54PM
Mode:	Chemi Blots
Notes:	
Model:	FL1500
Instrument name:	2462619090234
Serial No:	2462619090234
Firmware version:	1.6.0
iBA version:	5.0
Image size:	676px X 540px
Image area:	118.63mm X 94.91mm
Optical Zoom:	1.9x
Digital Zoom:	1x
Focus level:	430
Resolution:	5 x 5
Exposure time:	3916 ms
Exposure mode:	Normal

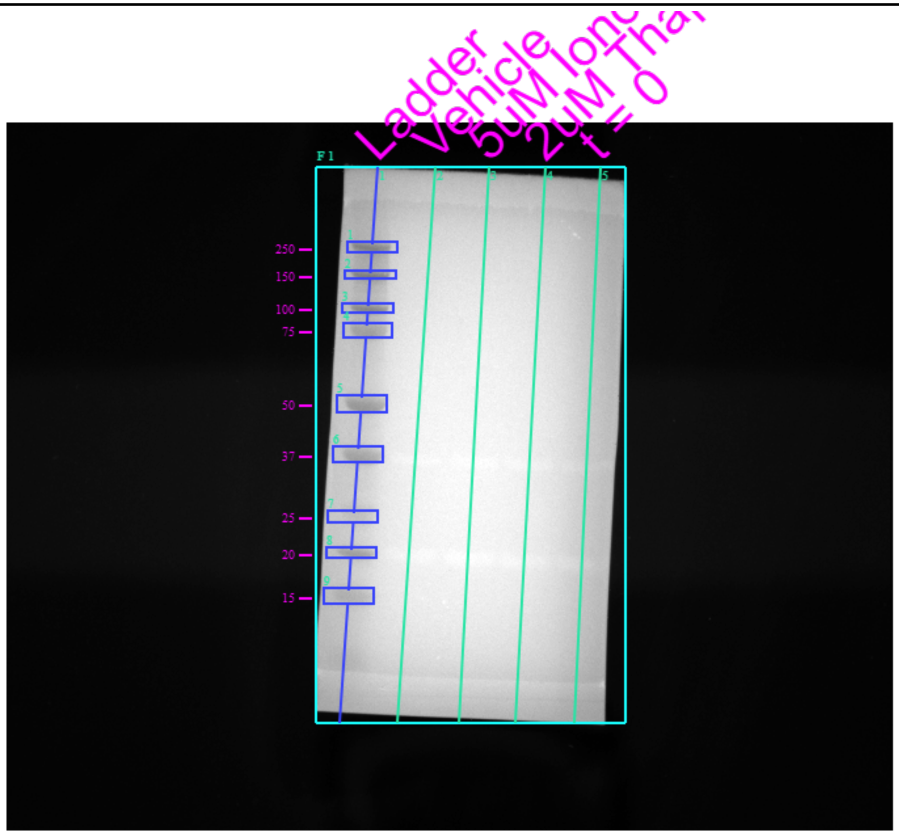

GAPDH_CHEMI_01282022_123954

Date:	28 January 2022 12:39:54PM
Mode:	Chemi Blots
Notes:	
Model:	FL1500
Instrument name:	2462619090234
Serial No:	2462619090234
Firmware version:	1.6.0
iBA version:	5.0
Image size:	676px X 540px
Image area:	118.63mm X 94.91mm
Optical Zoom:	1.9x
Digital Zoom:	1x
Focus level:	430
Resolution:	5 x 5
Exposure time:	3916 ms
Exposure mode:	Normal

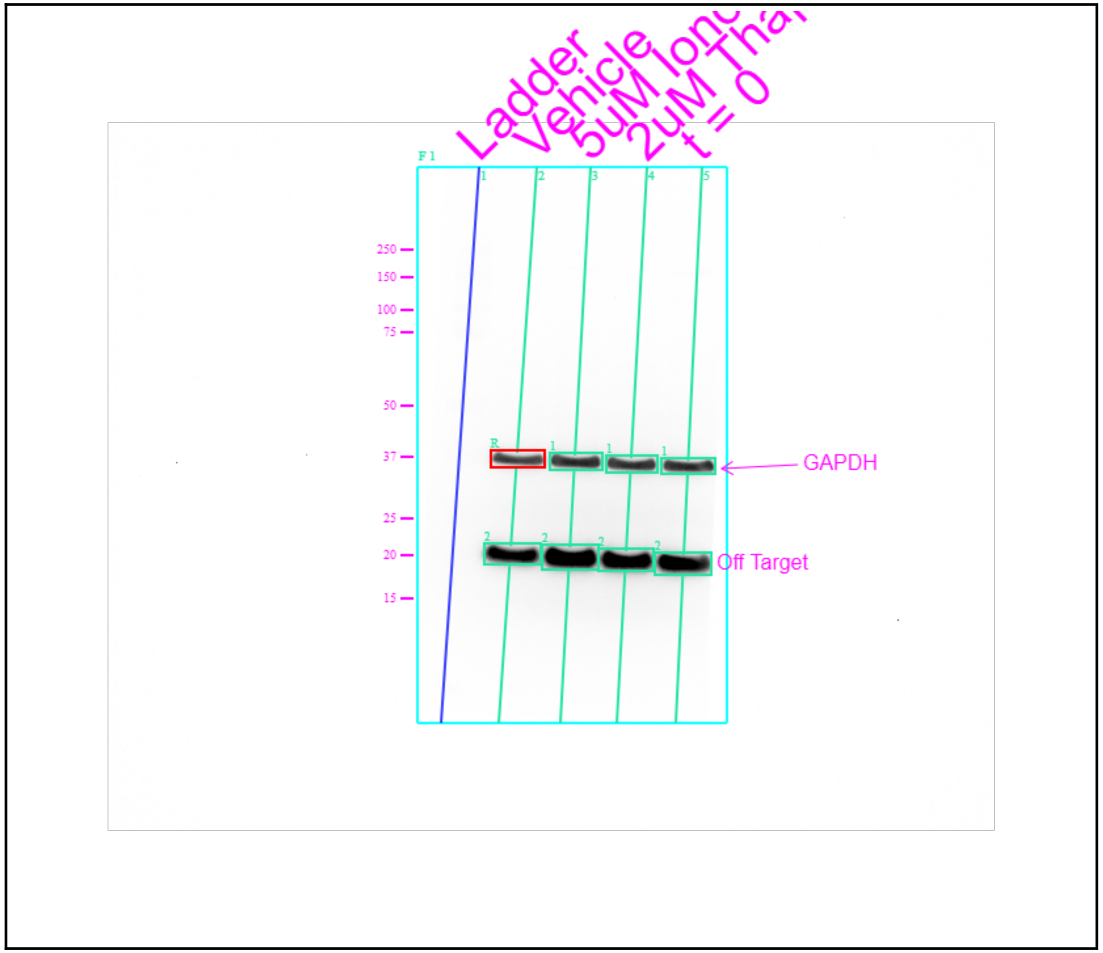

LANE AND BAND ANALYSIS DATA TABLE

GAPDH_CHEMI_01282022_123954

Frame: 1
Channel: Membrane
Sensitivity: 100
Molecular Weight Analysis Regression Method : Point to Point

Lane 1 - Ladder

#	Vol. (Int.)	Local Bg. Corr. Vol.	Area	Rf	Density	Local Bg. Corr. Den.	% band purity	% lane purity	Rolling Bg. Corr. Vol.	Rolling Bg. Corr. Den.	Mol. Wt.
1	12,089,443	1,807,999	351	0.144	34,442	5,150.996	11.49	1.903	1,790,720	5,101.766	250
2	9,663,006	1,523,984	280	0.193	34,510	5,442.801	9.475	1.569	1,476,608	5,273.6	150
3	10,548,732	1,469,454	320	0.252	32,964	4,592.047	9.941	1.646	1,549,312	4,841.6	100
4	14,003,618	1,823,279	456	0.292	30,709	3,998.42	10.326	1.71	1,609,216	3,528.982	75
5	16,815,111	2,403,605	546	0.425	30,796	4,402.209	18.501	3.064	2,883,328	5,280.821	50
6	15,246,370	1,612,971	507	0.517	30,071	3,181.403	15.286	2.531	2,382,336	4,698.888	37
7	10,621,665	66,475	390	0.627	27,235	170.451	5.215	0.864	812,800	2,084.103	25
8	10,641,212	934,031	351	0.693	30,316	2,661.057	10.751	1.78	1,675,520	4,773.561	20
9	14,910,698	355,651	507	0.771	29,409	701.481	9.015	1.493	1,404,928	2,771.061	15

Frame: 1
Channel: Chemi
Sensitivity: 100
Molecular Weight Analysis Regression Method : Point to Point

Lane 2 - Vehicle

#	Vol. (Int.)	Local Bg. Corr. Vol.	Area	Rf	Density	Local Bg. Corr. Den.
1	14,512,497	11,976,652	588	0.524	24,681	20,368
2	29,199,292	21,991,554	748	0.696	39,036	29,400

#	% band purity	% lane purity	Rolling Bg. Corr. Vol.	Rolling Bg. Corr. Den.	Mol. Wt.	Rel. Quant. (w/ LB Corr. Vol.)
1	33	31.609	13,156,352	22,374	36.234	1
2	67	64.176	26,711,552	35,710	19.848	1.836

Lane 3 - 5uM Ionomycin

#	Vol. (Int.)	Local Bg. Corr. Vol.	Area	Rf	Density	Local Bg. Corr. Den.

#	Vol. (Int.)	Local Bg. Corr. Vol.	Area	Rf	Density	Local Bg. Corr. Den.
1	17,421,985	13,894,673	574	0.528	30,351	24,206
2	38,688,769	27,379,302	880	0.7	43,964	31,112

#	% band purity	% lane purity	Rolling Bg. Corr. Vol.	Rolling Bg. Corr. Den.	Mol. Wt.	Rel. Quant. (w/ LB Corr. Vol.)
1	31.157	29.922	15,741,952	27,425	35.723	1.16
2	68.843	66.112	34,781,952	39,524	19.545	2.286

Lane 4 - 2uM Thapsigargin

#	Vol. (Int.)	Local Bg. Corr. Vol.	Area	Rf	Density	Local Bg. Corr. Den.
1	16,174,785	12,836,194	560	0.533	28,883	22,921
2	33,637,914	22,523,147	774	0.705	43,459	29,099

#	% band purity	% lane purity	Rolling Bg. Corr. Vol.	Rolling Bg. Corr. Den.	Mol. Wt.	Rel. Quant. (w/ LB Corr. Vol.)
1	32.205	30.003	14,465,280	25,830	35.213	1.072
2	67.795	63.16	30,451,456	39,342	19.242	1.881

Lane 5 - t = 0

#	Vol. (Int.)	Local Bg. Corr. Vol.	Area	Rf	Density	Local Bg. Corr. Den.
1	16,503,464	14,350,304	546	0.538	30,226	26,282
2	32,369,812	26,115,120	792	0.712	40,870	32,973

#	% band purity	% lane purity	Rolling Bg. Corr. Vol.	Rolling Bg. Corr. Den.	Mol. Wt.	Rel. Quant. (w/ LB Corr. Vol.)
1	33.677	31.72	15,247,360	27,925	34.702	1.198
2	66.323	62.47	30,028,288	37,914	18.788	2.181

iBright™ Image Analysis Report

Katarina+ Chang

19 November 2022

total Cofilin CHEMI_01272022_142940

Date:	27 January 2022 02:29:40PM
Mode:	Chemi Blots
Notes:	
Model:	FL1500
Instrument name:	2462619090234
Serial No:	2462619090234
Firmware version:	1.6.0
iBA version:	5.0
Image size:	676px X 540px
Image area:	132.59mm X 106.07mm
Optical Zoom:	1.7x
Digital Zoom:	1x
Focus level:	380
Resolution:	5 x 5
Exposure time:	170 ms
Exposure mode:	Normal

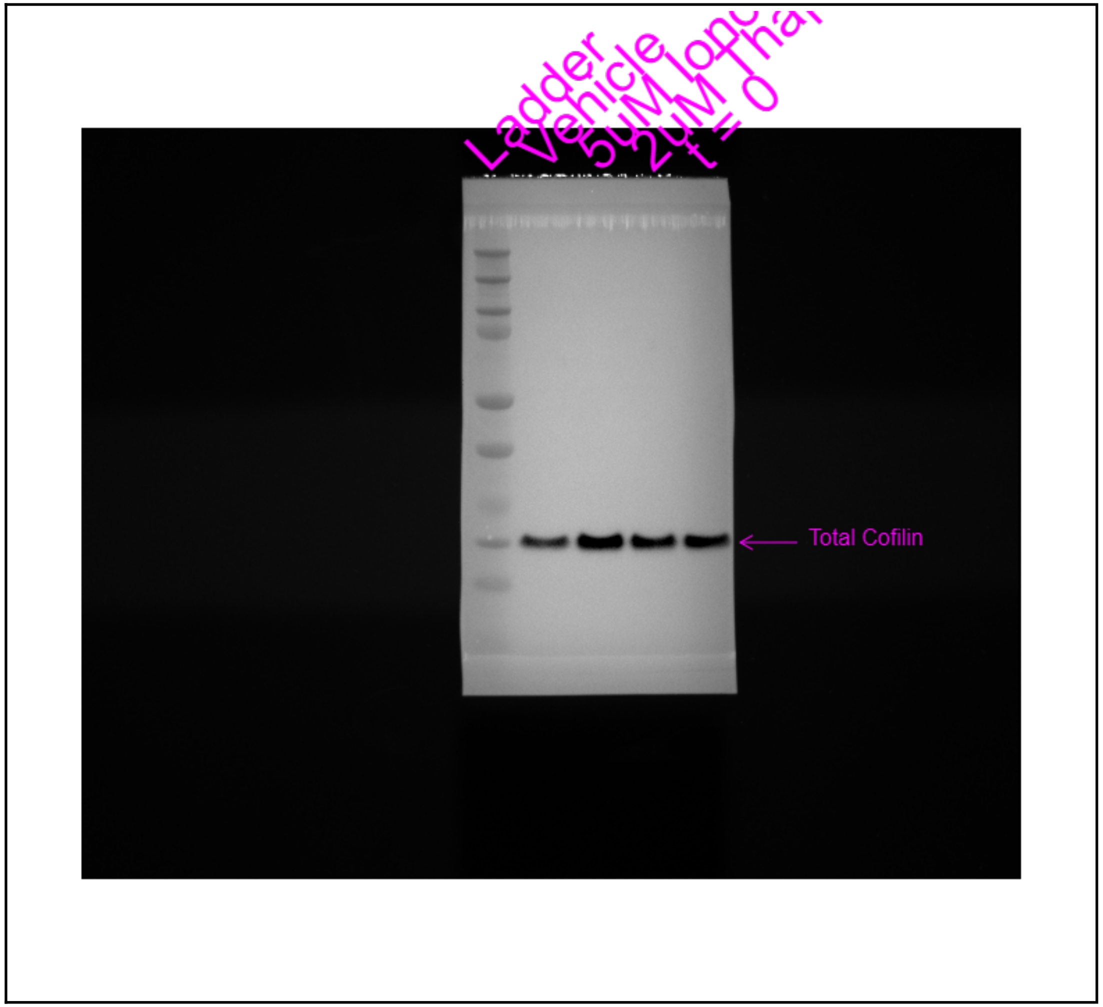

total Cofilin CHEMI_01272022_142940

Date: 27 January 2022 02:29:40PM
Mode: Chemi Blots
Notes:
Model: FL1500
Instrument name: 2462619090234
Serial No: 2462619090234
Firmware version: 1.6.0
iBA version: 5.0
Image size: 676px X 540px
Image area: 132.59mm X 106.07mm
Optical Zoom: 1.7x
Digital Zoom: 1x
Focus level: 380
Resolution: 5 x 5
Exposure time: 170 ms
Exposure mode: Normal

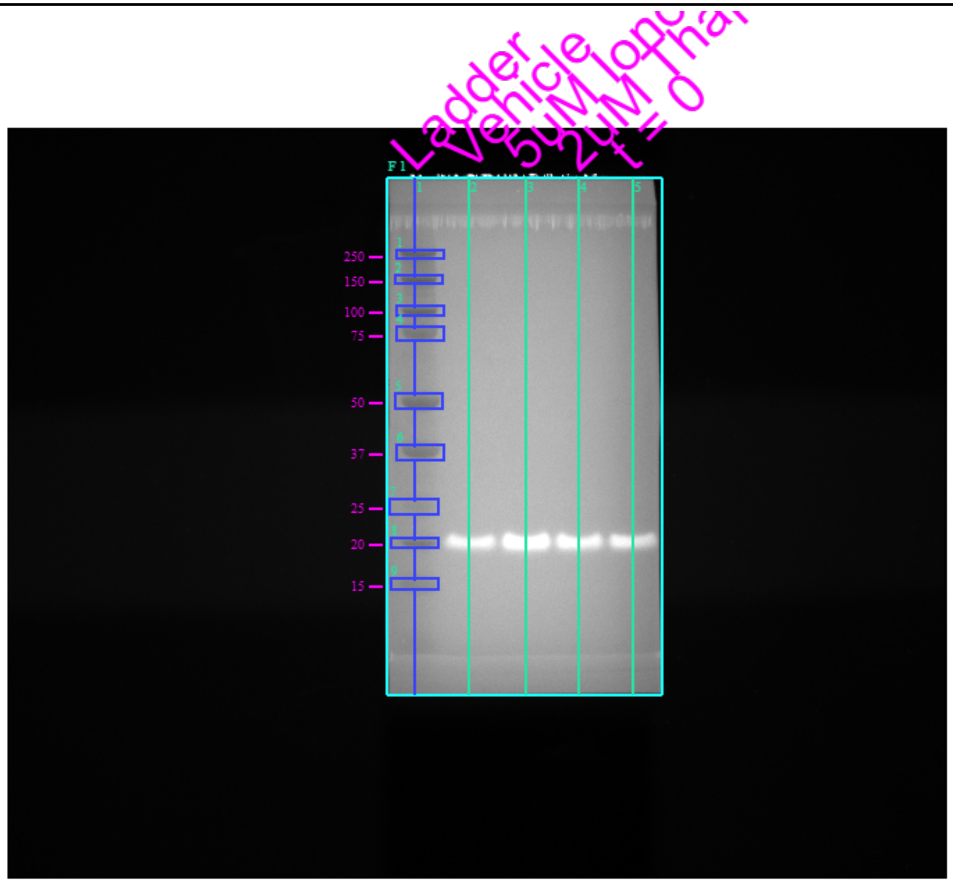

total Cofilin CHEMI_01272022_142940

Date:	27 January 2022 02:29:40PM
Mode:	Chemi Blots
Notes:	
Model:	FL1500
Instrument name:	2462619090234
Serial No:	2462619090234
Firmware version:	1.6.0
iBA version:	5.0
Image size:	676px X 540px
Image area:	132.59mm X 106.07mm
Optical Zoom:	1.7x
Digital Zoom:	1x
Focus level:	380
Resolution:	5 x 5
Exposure time:	170 ms
Exposure mode:	Normal

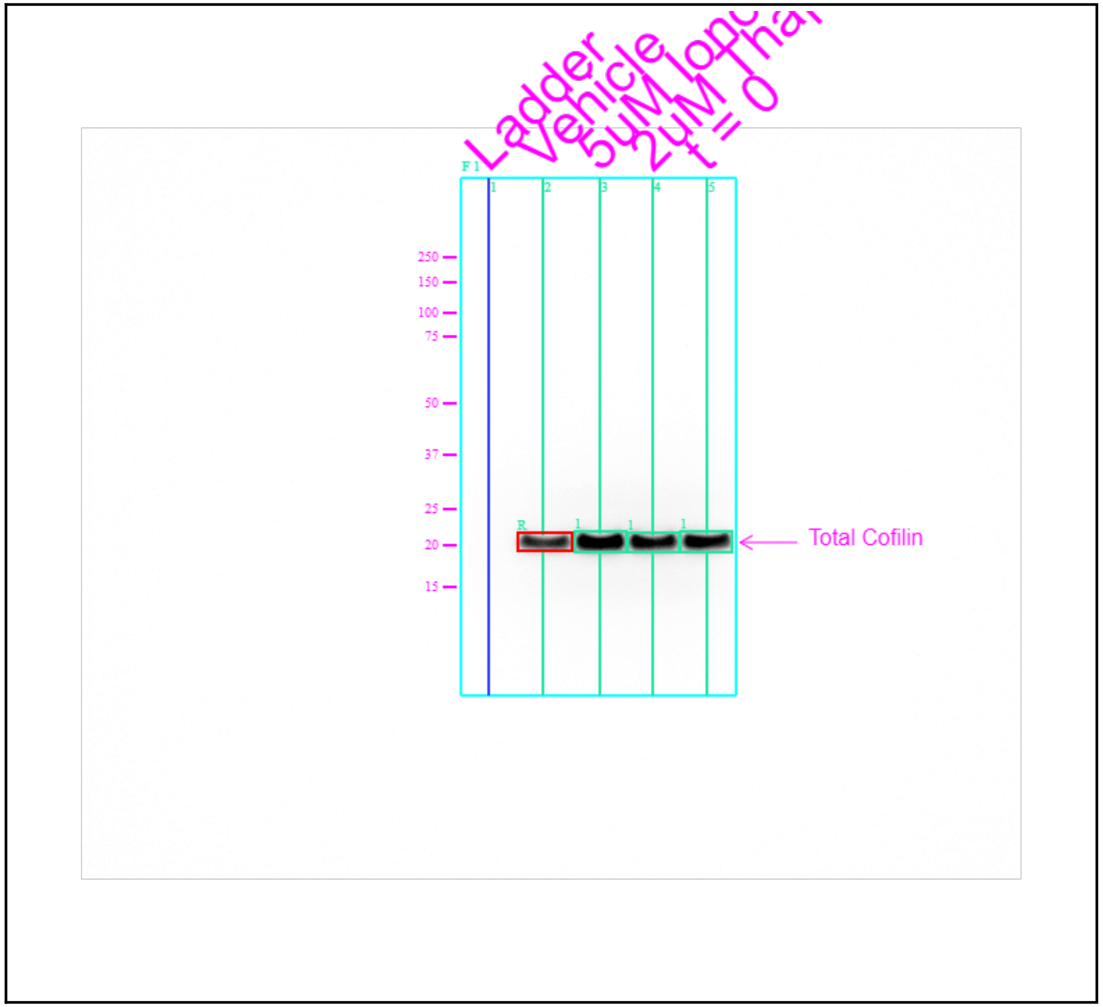

LANE AND BAND ANALYSIS DATA TABLE

total Cofilin CHEMI_01272022_142940

Frame: 1
Channel: Membrane
Sensitivity: 100
Molecular Weight Analysis Regression Method : Point to Point

Lane 1 - Ladder

#	Vol. (Int.)	Local Bg. Corr. Vol.	Area	Rf	Density	Local Bg. Corr. Den.	% band purity	% lane purity	Rolling Bg. Corr. Vol.	Rolling Bg. Corr. Den.	Mol. Wt.
1	8,395,091	1,370,771	245	0.148	34,265	5,594.986	11.609	3.075	1,445,120	5,898.449	250
2	8,383,476	1,378,785	245	0.196	34,218	5,627.695	9.768	2.587	1,216,000	4,963.265	150
3	9,154,359	1,587,530	280	0.255	32,694	5,669.751	10.373	2.747	1,291,264	4,611.657	100
4	11,609,112	1,571,240	385	0.301	30,153	4,081.145	9.596	2.541	1,194,496	3,102.587	75
5	12,956,350	2,388,532	420	0.43	30,848	5,686.983	17.593	4.66	2,190,080	5,214.476	50
6	12,158,345	1,965,626	420	0.53	28,948	4,680.063	14.219	3.766	1,769,984	4,214.248	37
7	11,417,393	2,957.336	432	0.634	26,429	6.846	7.173	1.9	892,928	2,066.963	25
8	8,332,789	841,415	280	0.704	29,759	3,005.056	11.407	3.021	1,420,032	5,071.543	20
9	9,041,308	279,302	315	0.785	28,702	886.674	8.261	2.188	1,028,352	3,264.61	15

Frame: 1
Channel: Chemi
Sensitivity: 100
Molecular Weight Analysis Regression Method : Point to Point

Lane 2 - Vehicle

#	Vol. (Int.)	Local Bg. Corr. Vol.	Area	Rf	Density	Local Bg. Corr. Den.
1	10,672,692	8,187,108	560	0.702	19,058	14,619

#	% band purity	% lane purity	Rolling Bg. Corr. Vol.	Rolling Bg. Corr. Den.	Mol. Wt.	Rel. Quant. (w/ LB Corr. Vol.)
1	100	95.33	9,955,328	17,777	20.192	1

Lane 3 - 5uM Ionomycin

#	Vol. (Int.)	Local Bg. Corr. Vol.	Area	Rf	Density	Local Bg. Corr. Den.
1	16,683,675	12,884,917	608	0.702	27,440	21,192

#	% band purity	% lane purity	Rolling Bg. Corr. Vol.	Rolling Bg. Corr. Den.	Mol. Wt.	Rel. Quant. (w/ LB Corr. Vol.)

#	% band purity	% lane purity	Rolling Bg. Corr. Vol.	Rolling Bg. Corr. Den.	Mol. Wt.	Rel. Quant. (w/ LB Corr. Vol.)
1	100	94.402	15,544,832	25,567	20.192	1.574

Lane 4 - 2uM Thapsigargin

#	Vol. (Int.)	Local Bg. Corr. Vol.	Area	Rf	Density	Local Bg. Corr. Den.	
1	13,587,432	9,608,389	570	0.704	23,837	16,856	

#	% band purity	% lane purity	Rolling Bg. Corr. Vol.	Rolling Bg. Corr. Den.	Mol. Wt.	Rel. Quant. (w/ LB Corr. Vol.)
1	100	92.438	12,285,184	21,552	20	1.174

Lane 5 - t = 0

#	Vol. (Int.)	Local Bg. Corr. Vol.	Area	Rf	Density	Local Bg. Corr. Den.	
1	13,979,708	11,815,652	608	0.702	22,992	19,433	

#	% band purity	% lane purity	Rolling Bg. Corr. Vol.	Rolling Bg. Corr. Den.	Mol. Wt.	Rel. Quant. (w/ LB Corr. Vol.)
1	100	93.509	13,085,184	21,521	20.192	1.443

iBright™ Image Analysis Report

Katarina+ Chang
19 November 2022

GAPDH CHEMI_02012022_153602

Date:	1 February 2022 03:36:02PM
Mode:	Chemi Blots
Notes:	
Model:	FL1500
Instrument name:	2462619090234
Serial No:	2462619090234
Firmware version:	1.6.0
iBA version:	5.0
Image size:	676px X 540px
Image area:	118.63mm X 94.91mm
Optical Zoom:	1.9x
Digital Zoom:	1x
Focus level:	430
Resolution:	5 x 5
Exposure time:	39327 ms
Exposure mode:	Normal

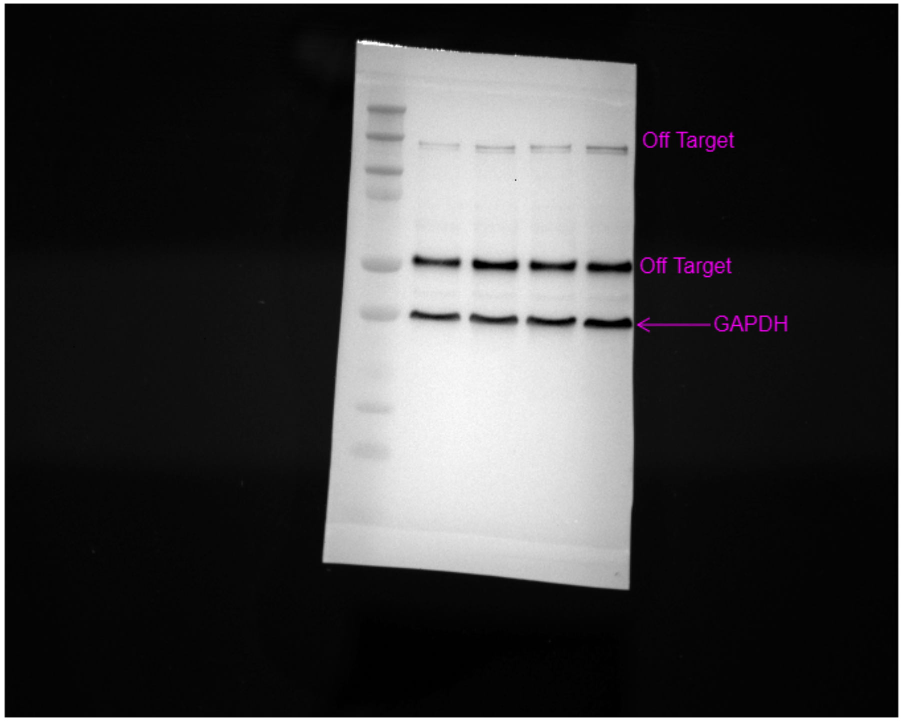

GAPDH CHEMI_02012022_153602

Date:	1 February 2022 03:36:02PM
Mode:	Chemi Blots
Notes:	
Model:	FL1500
Instrument name:	2462619090234
Serial No:	2462619090234
Firmware version:	1.6.0
iBA version:	5.0
Image size:	676px X 540px
Image area:	118.63mm X 94.91mm
Optical Zoom:	1.9x
Digital Zoom:	1x
Focus level:	430
Resolution:	5 x 5
Exposure time:	39327 ms
Exposure mode:	Normal

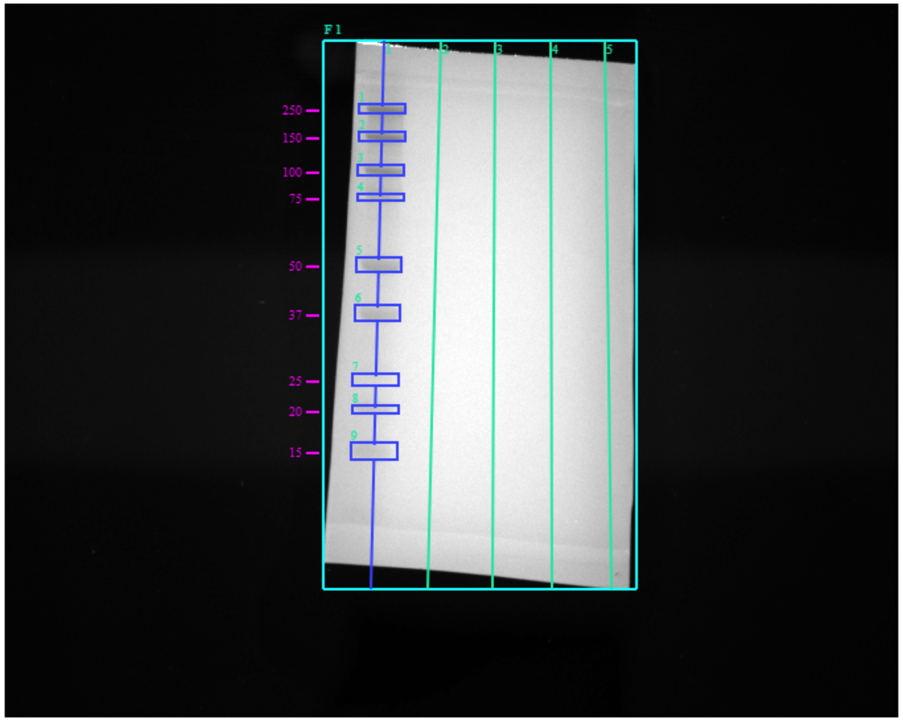

GAPDH CHEMI_02012022_153602

Date:	1 February 2022 03:36:02PM
Mode:	Chemi Blots
Notes:	
Model:	FL1500
Instrument name:	2462619090234
Serial No:	2462619090234
Firmware version:	1.6.0
iBA version:	5.0
Image size:	676px X 540px
Image area:	118.63mm X 94.91mm
Optical Zoom:	1.9x
Digital Zoom:	1x
Focus level:	430
Resolution:	5 x 5
Exposure time:	39327 ms
Exposure mode:	Normal

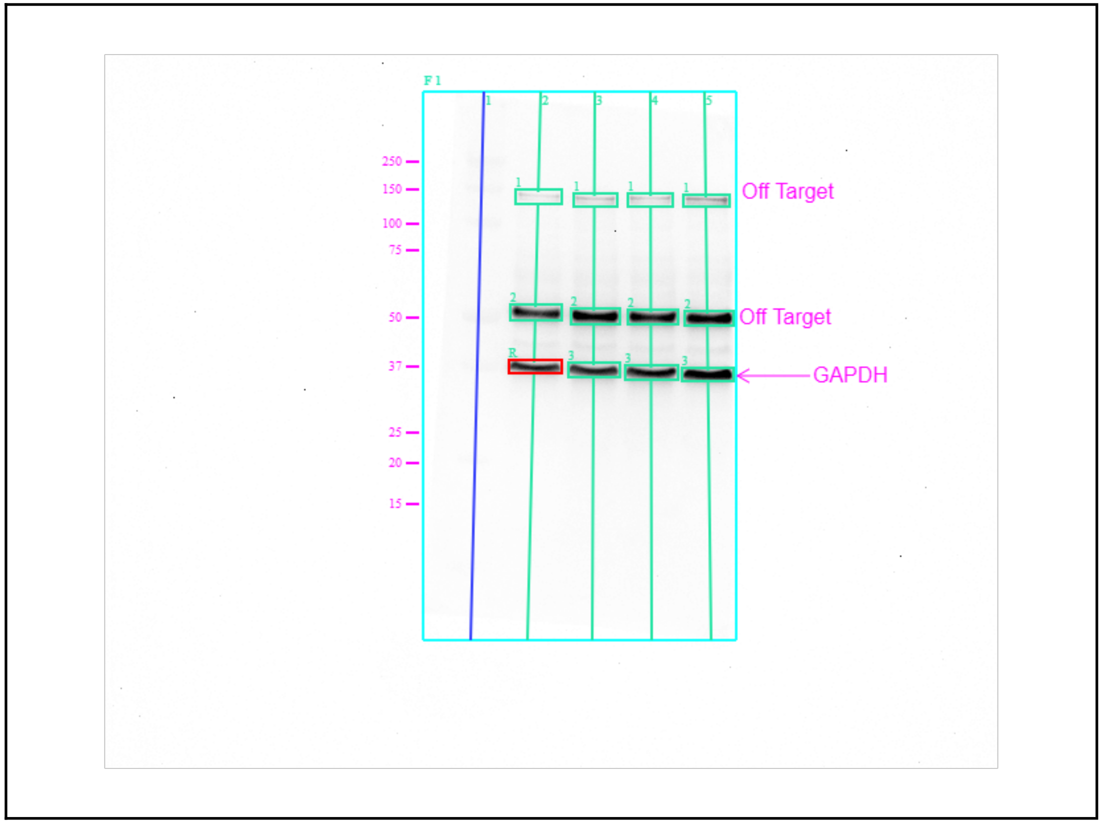

LANE AND BAND ANALYSIS DATA TABLE

GAPDH CHEMI_02012022_153602

Frame: 1
Channel: Membrane
Sensitivity: 100
Molecular Weight Analysis Regression Method : Point to Point

Lane 1

#	Vol. (Int.)	Local Bg. Corr. Vol.	Area	Rf	Density	Local Bg. Corr. Den.	% band purity	% lane purity	Mol. Wt.
1	10,408,382	1,335,139	288	0.123	36,140	4,635.903	12.313	2.447	250
2	10,156,216	1,527,701	288	0.173	35,264	5,304.52	14.089	2.388	150
3	10,774,534	1,565,158	324	0.236	33,254	4,830.735	14.434	2.533	100
4	6,420,816	544,632	216	0.284	29,726	2,521.449	5.023	1.509	75
5	12,525,058	2,003,993	420	0.407	29,821	4,771.414	18.481	2.945	50
6	12,747,959	1,738,351	455	0.496	28,017	3,820.552	16.031	2.997	37
7	8,760,221	351,535	360	0.617	24,333	976.486	3.242	2.059	25
8	6,595,206	594,731	252	0.672	26,171	2,360.047	5.485	1.55	20
9	13,221,618	1,182,305	504	0.747	26,233	2,345.845	10.903	3.108	15

Frame: 1
Channel: Chemi
Sensitivity: 100
Molecular Weight Analysis Regression Method : Point to Point

Lane 2

#	Vol. (Int.)	Local Bg. Corr. Vol.	Area	Rf	Density	Local Bg. Corr. Den.	% band purity	% lane purity	Mol. Wt.	Rel. Quant. (w/ LB Corr. Vol.)
1	1,114,161	623,209	432	0.19	2,579.076	1,442.614	4.185	3.351	136.538	0.095
2	9,736,679	7,675,993	520	0.402	18,724	14,761	51.542	29.287	50.98	1.164
3	8,288,516	6,593,630	451	0.501	18,378	14,620	44.274	24.931	36.52	1

Lane 3

#	Vol. (Int.)	Local Bg. Corr. Vol.	Area	Rf	Density	Local Bg. Corr. Den.	% band purity	% lane purity	Mol. Wt.	Rel. Quant. (w/ LB Corr. Vol.)
1	1,634,323	1,084,447	374	0.198	4,369.848	2,899.593	6.151	3.886	130.769	0.164
2	12,413,770	9,800,147	494	0.41	25,129	19,838	55.587	29.516	49.649	1.486

#	Vol. (Int.)	Local Bg. Corr. Vol.	Area	Rf	Density	Local Bg. Corr. Den.	% band purity	% lane purity	Mol. Wt.	Rel. Quant. (w/ LB Corr. Vol.)
3	9,076,326	6,745,806	480	0.506	18,909	14,053	38.262	21.581	36.04	1.023

Lane 4

#	Vol. (Int.)	Local Bg. Corr. Vol.	Area	Rf	Density	Local Bg. Corr. Den.	% band purity	% lane purity	Mol. Wt.	Rel. Quant. (w/ LB Corr. Vol.)
1	1,571,541	1,033,156	385	0.198	4,081.925	2,683.523	6.086	3.842	130.769	0.157
2	10,868,777	8,407,780	468	0.41	23,223	17,965	49.529	26.57	49.649	1.275
3	10,178,392	7,534,438	492	0.511	20,687	15,313	44.385	24.882	35.56	1.143

Lane 5

#	Vol. (Int.)	Local Bg. Corr. Vol.	Area	Rf	Density	Local Bg. Corr. Den.	% band purity	% lane purity	Mol. Wt.	Rel. Quant. (w/ LB Corr. Vol.)
1	2,585,334	2,083,901	360	0.198	7,181.483	5,788.616	9.441	5.908	130.769	0.316
2	11,900,655	9,974,807	456	0.412	26,097	21,874	45.188	27.197	49.297	1.513
3	11,746,468	10,015,189	440	0.516	26,696	22,761	45.371	26.844	35.08	1.519

iBright™ Image Analysis Report

Katarina+ Chang

19 November 2022

pMLC CHEMI_01282022_125247

Date:	28 January 2022 12:52:47PM
Mode:	Chemi Blots
Notes:	
Model:	FL1500
Instrument name:	2462619090234
Serial No:	2462619090234
Firmware version:	1.6.0
iBA version:	5.0
Image size:	676px X 540px
Image area:	118.63mm X 94.91mm
Optical Zoom:	1.9x
Digital Zoom:	1x
Focus level:	430
Resolution:	5 x 5
Exposure time:	5653 ms
Exposure mode:	Normal

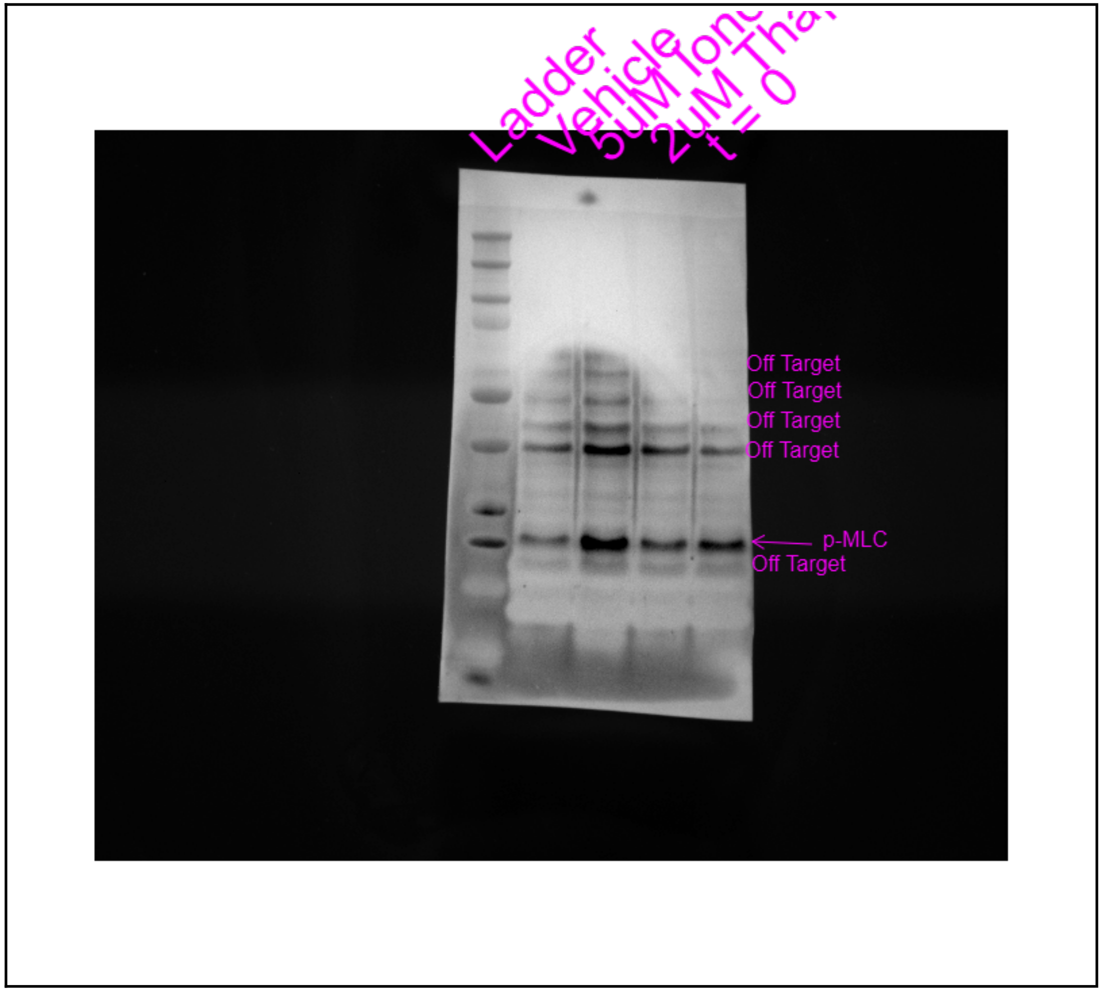

pMLC CHEMI_01282022_125247

Date:	28 January 2022 12:52:47PM
Mode:	Chemi Blots
Notes:	
Model:	FL1500
Instrument name:	2462619090234
Serial No:	2462619090234
Firmware version:	1.6.0
iBA version:	5.0
Image size:	676px X 540px
Image area:	118.63mm X 94.91mm
Optical Zoom:	1.9x
Digital Zoom:	1x
Focus level:	430
Resolution:	5 x 5
Exposure time:	5653 ms
Exposure mode:	Normal

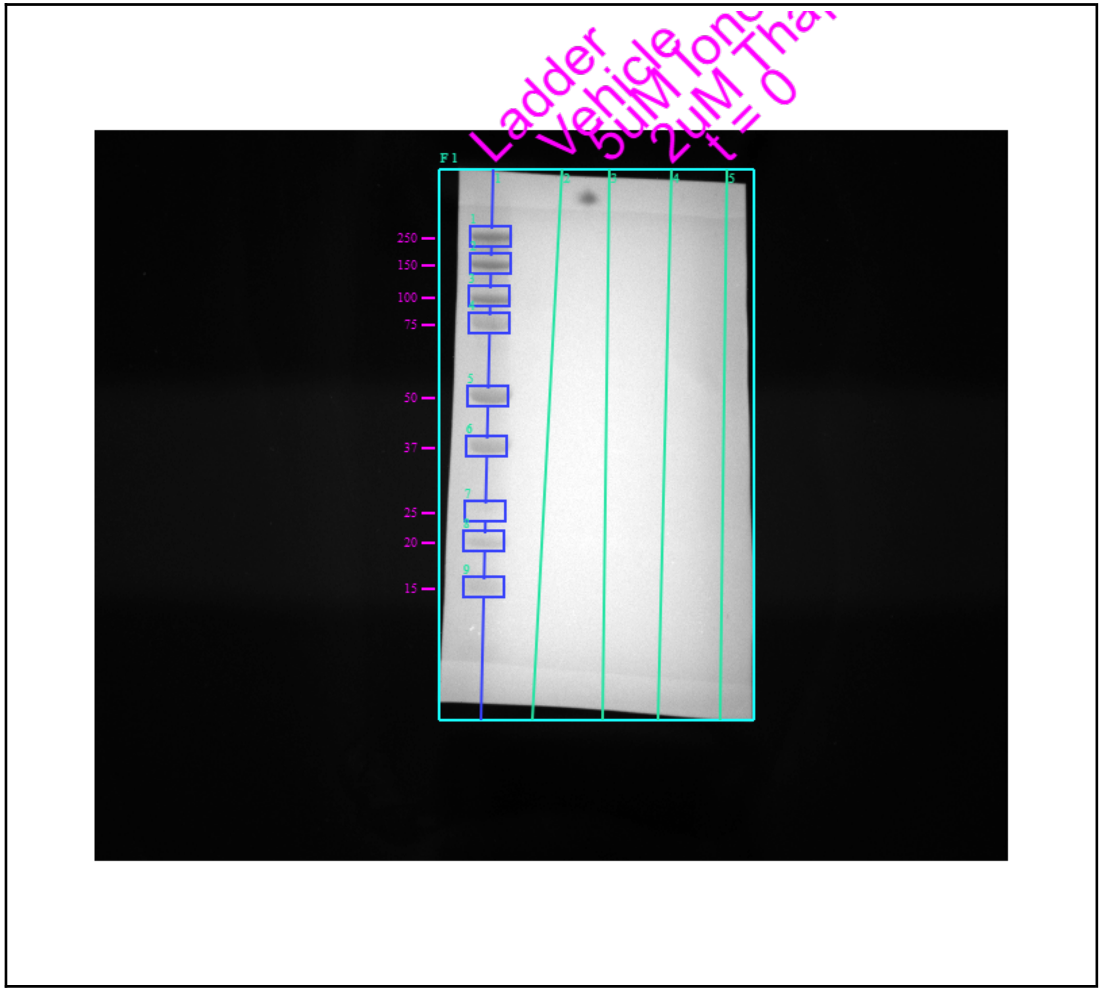

pMLC CHEMI_01282022_125247

Date:	28 January 2022 12:52:47PM
Mode:	Chemi Blots
Notes:	
Model:	FL1500
Instrument name:	2462619090234
Serial No:	2462619090234
Firmware version:	1.6.0
iBA version:	5.0
Image size:	676px X 540px
Image area:	118.63mm X 94.91mm
Optical Zoom:	1.9x
Digital Zoom:	1x
Focus level:	430
Resolution:	5 x 5
Exposure time:	5653 ms
Exposure mode:	Normal

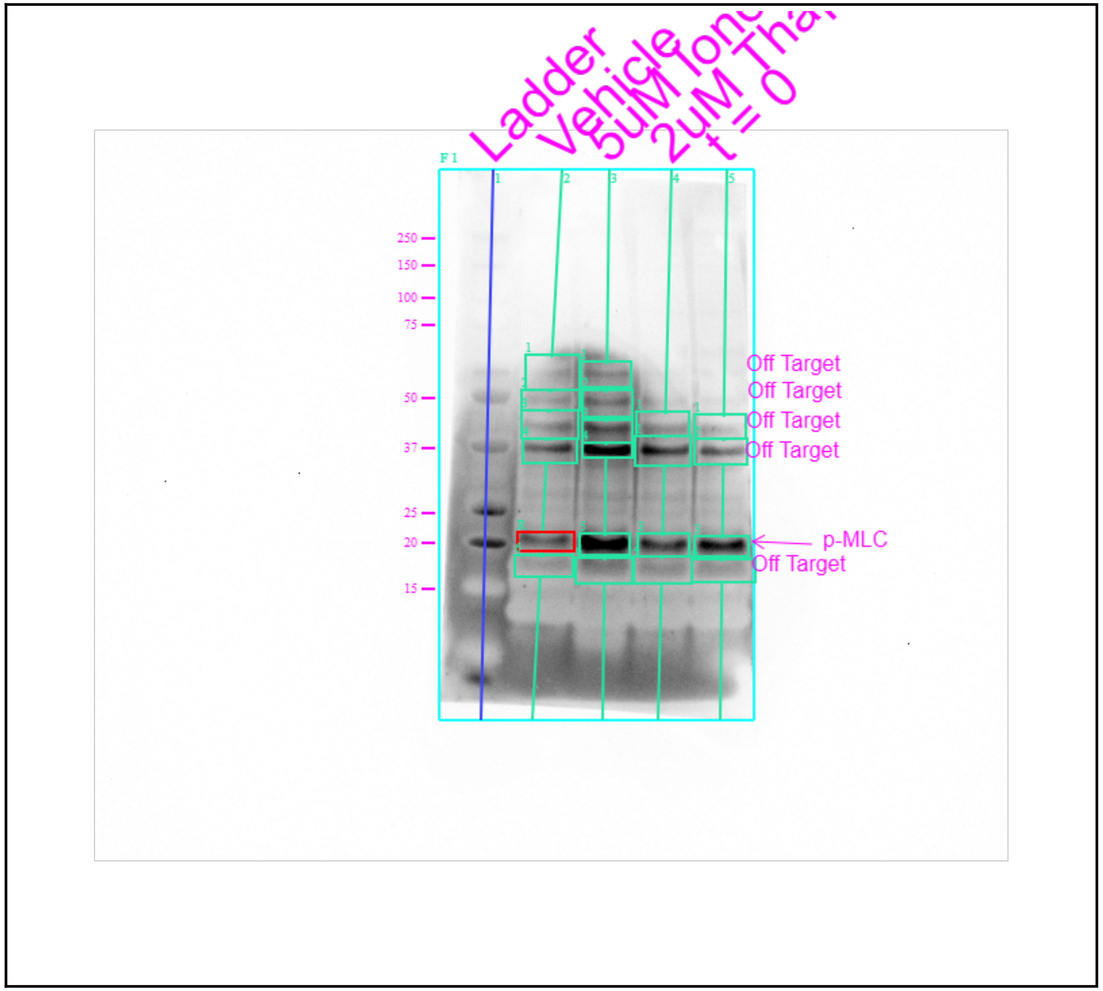

LANE AND BAND ANALYSIS DATA TABLE

pMLC CHEMI_01282022_125247

Frame: 1
Channel: Membrane
Sensitivity: 100
Molecular Weight Analysis Regression Method : Point to Point

Lane 1 - Ladder

#	Vol. (Int.)	Local Bg. Corr. Vol.	Area	Rf	Density	Local Bg. Corr. Den.	% band purity	% lane purity	Mol. Wt.
1	17,258,596	2,411,289	496	0.12	34,795	4,861.47	15.158	4.142	250
2	16,853,377	2,399,820	496	0.17	33,978	4,838.348	15.086	4.044	150
3	16,023,051	2,175,499	496	0.229	32,304	4,386.088	13.676	3.845	100
4	14,933,152	1,731,936	496	0.278	30,107	3,491.807	10.887	3.584	75
5	14,932,173	2,416,733	496	0.41	30,105	4,872.446	15.192	3.583	50
6	14,160,173	1,943,568	496	0.501	28,548	3,918.484	12.218	3.398	37
7	12,386,929	531,496	496	0.619	24,973	1,071.565	3.341	2.973	25
8	13,090,300	1,073,897	496	0.673	26,391	2,165.116	6.751	3.141	20
9	13,477,086	1,223,700	496	0.757	27,171	2,467.139	7.692	3.234	15

Frame: 1
Channel: Chemi
Sensitivity: 100
Molecular Weight Analysis Regression Method : Point to Point

Lane 2 - Vehicle

#	Vol. (Int.)	Local Bg. Corr. Vol.	Area	Rf	Density	Local Bg. Corr. Den.	% band purity	% lane purity	Mol. Wt.	Rel. Quant. (w/ LB Corr. Vol.)
1	10,091,423	2,018,721	1,107	0.369	9,116.01	1,823.597	22.106	8.665	57.87	0.936
2	7,622,139	1,038,029	704	0.418	10,826	1,474.474	11.367	6.545	48.946	0.481
3	11,194,348	983,759	946	0.462	11,833	1,039.915	10.773	9.612	42.622	0.456
4	11,927,925	2,410,298	779	0.511	15,311	3,094.093	26.394	10.242	36	1.117
5	10,568,184	2,157,870	645	0.676	16,384	3,345.536	23.63	9.074	19.853	1
6	8,540,372	523,410	748	0.72	11,417	699.746	5.732	7.333	17.206	0.243

Lane 3 - 5uM Ionomycin

#	Vol. (Int.)	Local Bg. Corr. Vol.	Area	Rf	Density	Local Bg. Corr. Den.	% band purity	% lane purity	Mol. Wt.	Rel. Quant. (w/ LB Corr. Vol.)
1	9,861,371	950,920	760	0.371	12,975	1,251.211	6.123	6.741	57.407	0.441
2	11,628,059	820,879	798	0.425	14,571	1,028.672	5.286	7.949	47.892	0.38
3	12,534,164	658,180	684	0.474	18,324	962.252	4.238	8.568	40.865	0.305
4	11,943,217	4,015,033	456	0.509	26,191	8,804.899	25.854	8.164	36.25	1.861
5	17,521,100	7,783,471	646	0.681	27,122	12,048	50.12	11.977	19.559	3.607
6	11,636,657	1,301,290	860	0.727	13,530	1,513.128	8.379	7.955	16.765	0.603

Lane 4 - 2uM Thapsigargin

#	Vol. (Int.)	Local Bg. Corr. Vol.	Area	Rf	Density	Local Bg. Corr. Den.	% band purity	% lane purity	Mol. Wt.	Rel. Quant. (w/ LB Corr. Vol.)
1	8,833,967	1,097,440	760	0.462	11,623	1,444.001	12.883	7.531	42.622	0.509
2	15,848,207	2,974,978	966	0.511	16,406	3,079.688	34.925	13.51	36	1.379
3	12,473,366	3,689,046	702	0.681	17,768	5,255.051	43.307	10.633	19.559	1.71
4	11,042,770	756,831	924	0.727	11,951	819.082	8.885	9.414	16.765	0.351

Lane 5 - t = 0

#	Vol. (Int.)	Local Bg. Corr. Vol.	Area	Rf	Density	Local Bg. Corr. Den.	% band purity	% lane purity	Mol. Wt.	Rel. Quant. (w/ LB Corr. Vol.)
1	4,936,755	1,184,182	741	0.467	6,662.287	1,598.087	10.002	5.761	41.919	0.549
2	8,510,103	2,834,172	800	0.511	10,637	3,542.716	23.939	9.931	36	1.313
3	12,631,330	5,760,052	656	0.683	19,255	8,780.567	48.653	14.741	19.412	2.669
4	8,929,766	2,060,730	864	0.727	10,335	2,385.105	17.406	10.421	16.765	0.955

iBright™ Image Analysis Report

Katarina+ Chang

19 November 2022

total MYPT1 CHEMI_01312022_123511

Date:	31 January 2022 12:35:11PM
Mode:	Chemi Blots
Notes:	
Model:	FL1500
Instrument name:	2462619090234
Serial No:	2462619090234
Firmware version:	1.6.0
iBA version:	5.0
Image size:	615px X 491px
Image area:	112.7mm X 90.16mm
Optical Zoom:	2x
Digital Zoom:	1.1x
Focus level:	455
Resolution:	5 x 5
Exposure time:	2694 ms
Exposure mode:	Normal

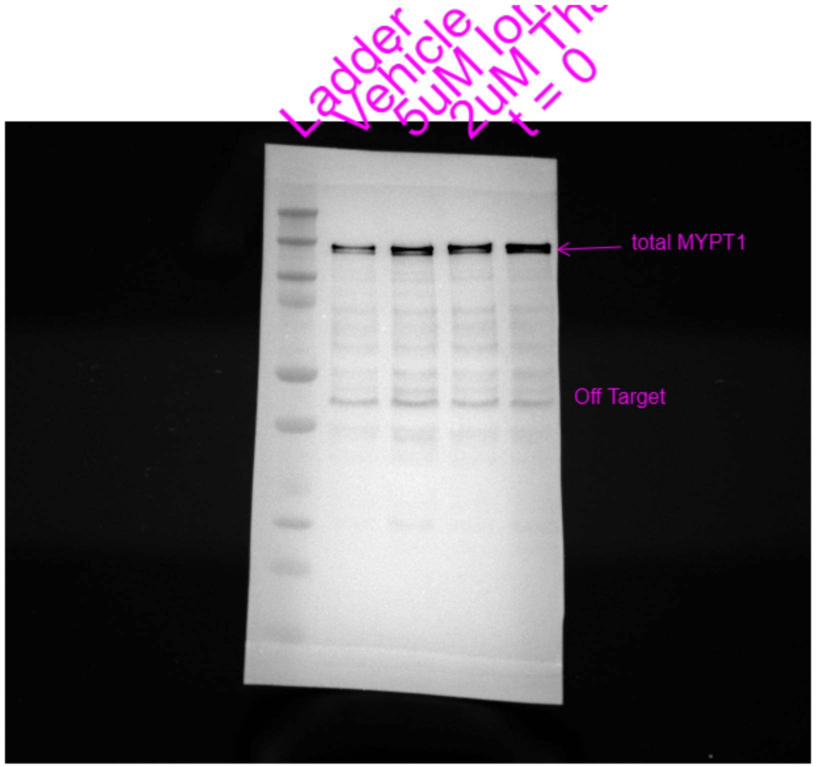

total MYPT1 CHEMI_01312022_123511

Date:	31 January 2022 12:35:11PM
Mode:	Chemi Blots
Notes:	
Model:	FL1500
Instrument name:	2462619090234
Serial No:	2462619090234
Firmware version:	1.6.0
iBA version:	5.0
Image size:	615px X 491px
Image area:	112.7mm X 90.16mm
Optical Zoom:	2x
Digital Zoom:	1.1x
Focus level:	455
Resolution:	5 x 5
Exposure time:	2694 ms
Exposure mode:	Normal

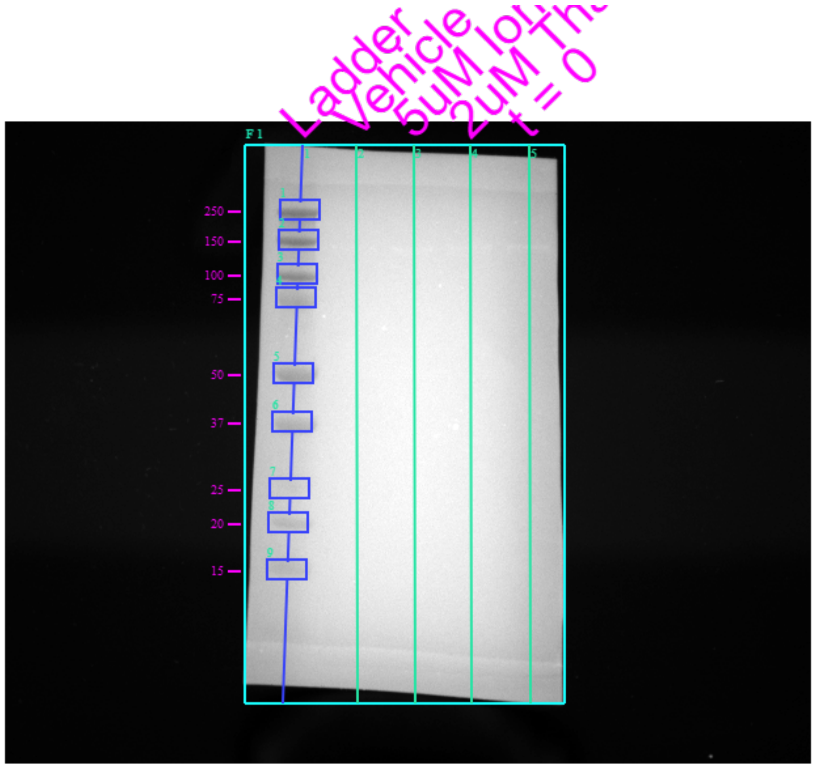

total MYPT1 CHEMI_01312022_123511

Date:	31 January 2022 12:35:11PM
Mode:	Chemi Blots
Notes:	
Model:	FL1500
Instrument name:	2462619090234
Serial No:	2462619090234
Firmware version:	1.6.0
iBA version:	5.0
Image size:	615px X 491px
Image area:	112.7mm X 90.16mm
Optical Zoom:	2x
Digital Zoom:	1.1x
Focus level:	455
Resolution:	5 x 5
Exposure time:	2694 ms
Exposure mode:	Normal

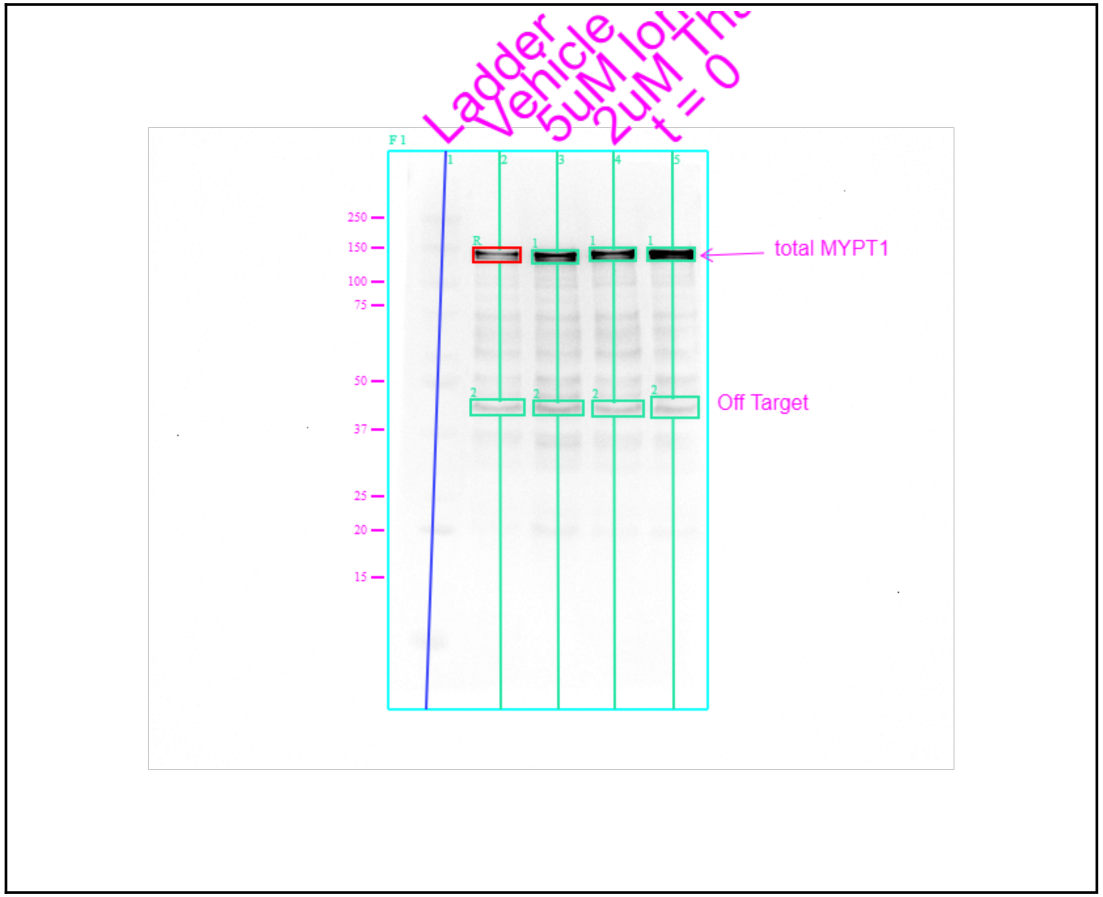

LANE AND BAND ANALYSIS DATA TABLE

total MYPT1 CHEMI_01312022_123511

Frame: 1
Channel: Membrane
Sensitivity: 100
Molecular Weight Analysis Regression Method : Point to Point

Lane 1 - Ladder

#	Vol. (Int.)	Local Bg. Corr. Vol.	Area	Rf	Density	Local Bg. Corr. Den.	% band purity	% lane purity	Mol. Wt.
1	17,412,028	2,133,413	496	0.115	35,104	4,301.238	14.502	2.729	250
2	17,085,522	2,371,882	496	0.169	34,446	4,782.022	16.123	2.678	150
3	16,230,590	2,221,203	496	0.23	32,722	4,478.233	15.099	2.544	100
4	15,260,193	1,538,836	496	0.272	30,766	3,102.492	10.46	2.391	75
5	15,227,204	2,311,624	496	0.407	30,700	4,660.533	15.713	2.386	50
6	14,453,954	1,620,861	496	0.494	29,141	3,267.866	11.018	2.265	37
7	13,114,698	532,591	496	0.614	26,440	1,073.774	3.62	2.055	25
8	13,781,078	989,583	496	0.674	27,784	1,995.128	6.727	2.16	20
9	14,353,604	991,312	496	0.759	28,938	1,998.614	6.738	2.249	15

Frame: 1
Channel: Chemi
Sensitivity: 100
Molecular Weight Analysis Regression Method : Point to Point

Lane 2 - Vehicle

#	Vol. (Int.)	Local Bg. Corr. Vol.	Area	Rf	Density	Local Bg. Corr. Den.	% band purity	% lane purity	Mol. Wt.	Rel. Quant. (w/ LB Corr. Vol.)
1	3,353,501	2,547,488	444	0.185	7,552.93	5,737.586	81.118	0.275	136.538	1
2	1,383,580	592,978	546	0.459	2,534.029	1,086.042	18.882	0.114	42.27	0.233

Lane 3 - 5uM Ionomycin

#	Vol. (Int.)	Local Bg. Corr. Vol.	Area	Rf	Density	Local Bg. Corr. Den.	% band purity	% lane purity	Mol. Wt.	Rel. Quant. (w/ LB Corr. Vol.)
1	5,606,239	4,426,594	396	0.19	14,157	11,178	85.094	0.599	132.692	1.738
2	1,734,039	775,424	468	0.459	3,705.212	1,656.889	14.906	0.185	42.27	0.304

Lane 4 - 2uM Thapsigargin

#	Vol. (Int.)	Local Bg. Corr. Vol.	Area	Rf	Density	Local Bg. Corr. Den.	% band purity	% lane purity	Mol. Wt.	Rel. Quant. (w/ LB Corr. Vol.)
1	5,366,284	4,383,555	396	0.185	13,551	11,069	87.637	0.843	136.538	1.721
2	1,424,561	618,375	520	0.461	2,739.54	1,189.184	12.363	0.224	41.919	0.243

Lane 5 - t = 0

#	Vol. (Int.)	Local Bg. Corr. Vol.	Area	Rf	Density	Local Bg. Corr. Den.	% band purity	% lane purity	Mol. Wt.	Rel. Quant. (w/ LB Corr. Vol.)
1	7,775,896	7,137,034	407	0.185	19,105	17,535	90.53	2.893	136.538	2.802
2	1,417,008	746,619	629	0.459	2,252.795	1,186.995	9.47	0.527	42.27	0.293

iBright™ Image Analysis Report

Katarina+ Chang

19 November 2022

Acetyl Tubulin CHEMI_02012022_115309

Date:	1 February 2022 11:53:09AM
Mode:	Chemi Blots
Notes:	
Model:	FL1500
Instrument name:	2462619090234
Serial No:	2462619090234
Firmware version:	1.6.0
iBA version:	5.0
Image size:	615px X 491px
Image area:	112.7mm X 90.16mm
Optical Zoom:	2x
Digital Zoom:	1.1x
Focus level:	455
Resolution:	5 x 5
Exposure time:	38000 ms
Exposure mode:	Normal

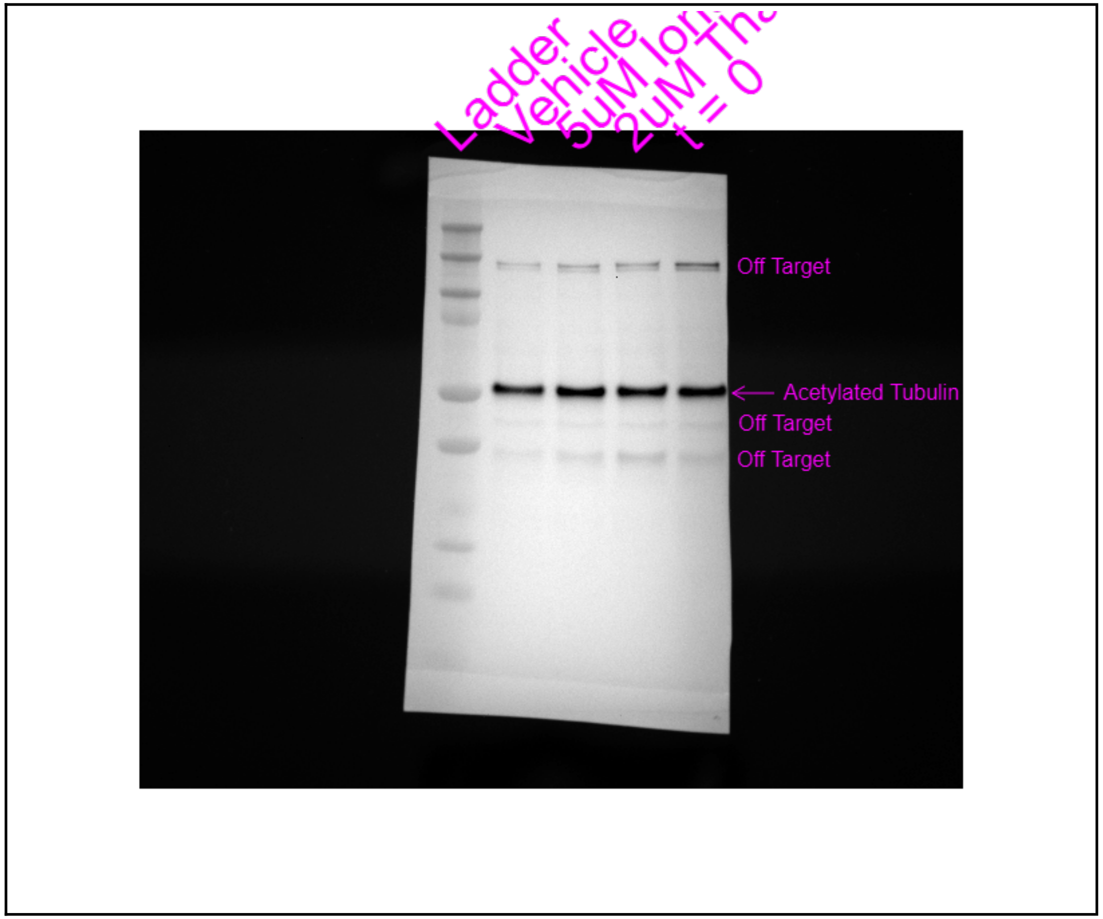

Acetyl Tubulin CHEMI_02012022_115309

Date:	1 February 2022 11:53:09AM
Mode:	Chemi Blots
Notes:	
Model:	FL1500
Instrument name:	2462619090234
Serial No:	2462619090234
Firmware version:	1.6.0
iBA version:	5.0
Image size:	615px X 491px
Image area:	112.7mm X 90.16mm
Optical Zoom:	2x
Digital Zoom:	1.1x
Focus level:	455
Resolution:	5 x 5
Exposure time:	38000 ms
Exposure mode:	Normal

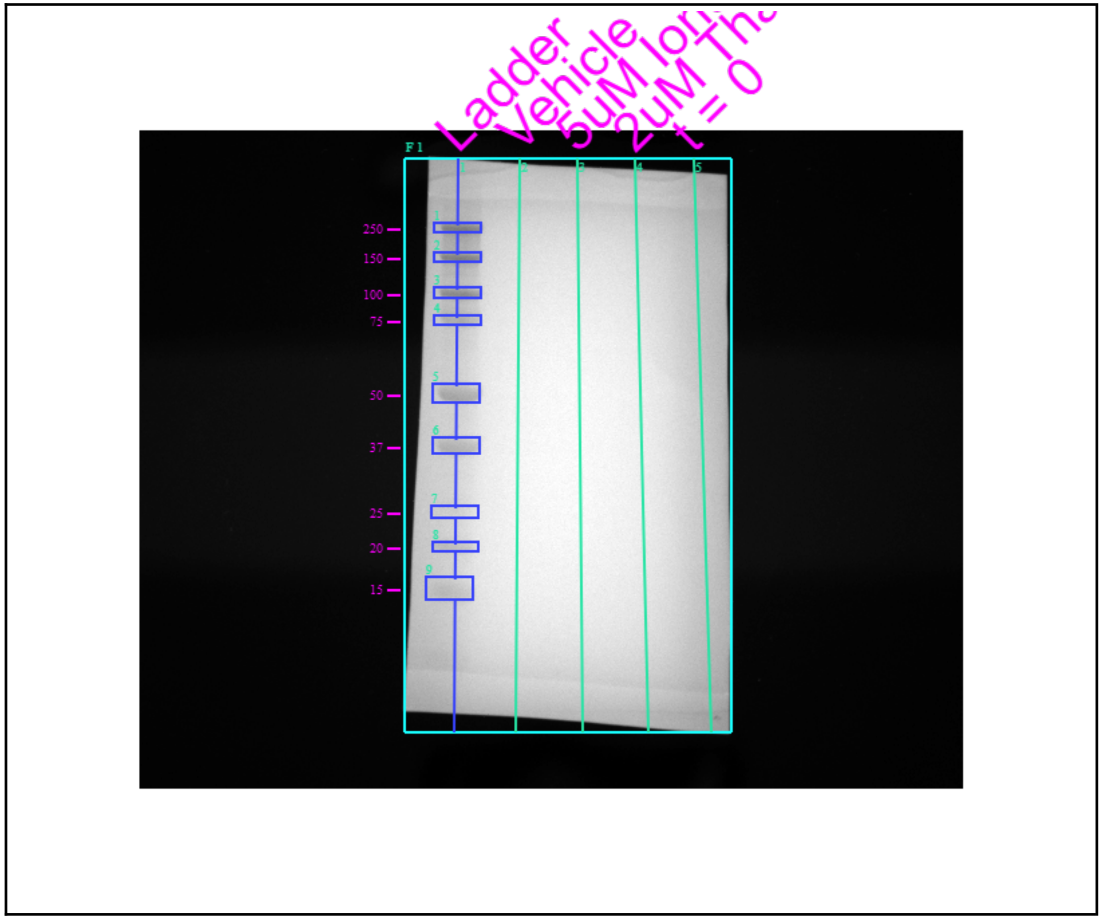

Acetyl Tubulin CHEMI_02012022_11530 9

Date:	1 February 2022 11:53:09AM
Mode:	Chemi Blots
Notes:	
Model:	FL1500
Instrument name:	2462619090234
Serial No:	2462619090234
Firmware version:	1.6.0
iBA version:	5.0
Image size:	615px X 491px
Image area:	112.7mm X 90.16mm
Optical Zoom:	2x
Digital Zoom:	1.1x
Focus level:	455
Resolution:	5 x 5
Exposure time:	38000 ms
Exposure mode:	Normal

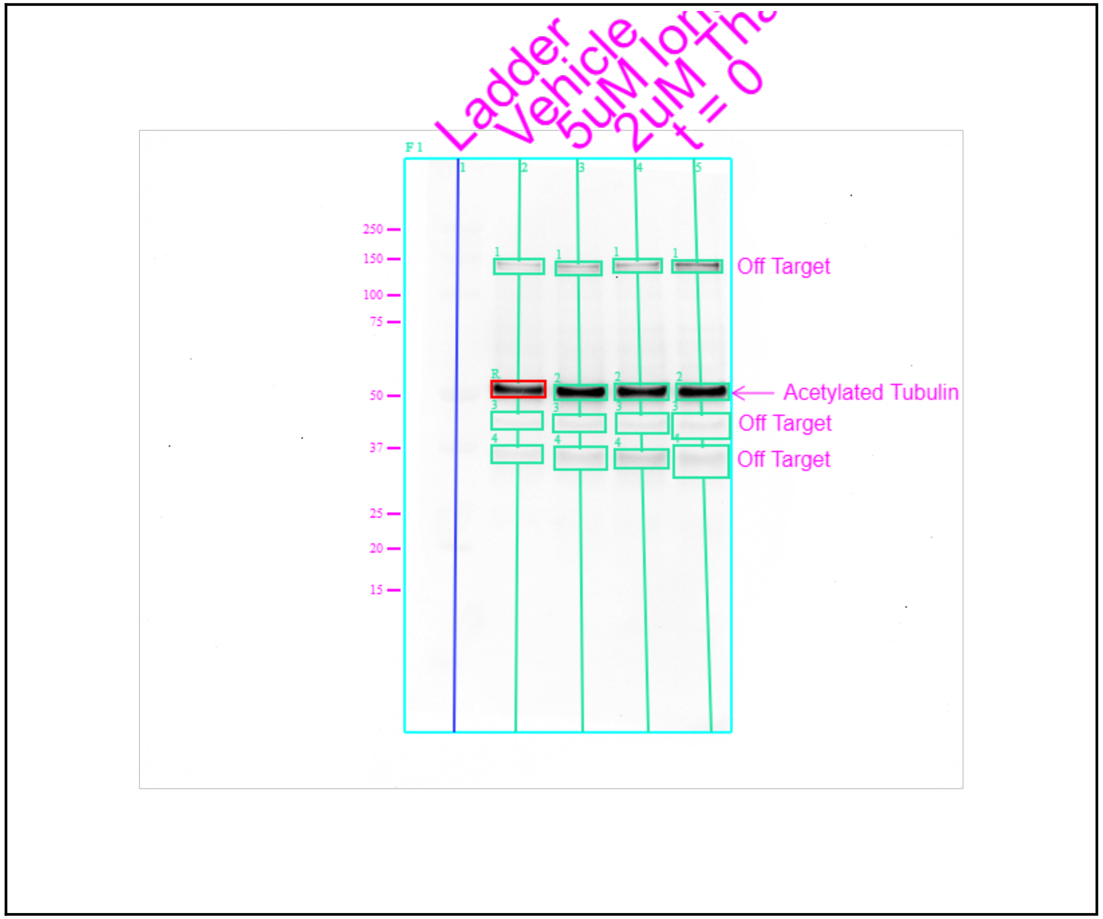

LANE AND BAND ANALYSIS DATA TABLE

Acetyl Tubulin CHEMI_02012022_115309

Frame: 1
Channel: Membrane
Sensitivity: 100
Molecular Weight Analysis Regression Method : Point to Point

Lane 1 - Ladder

#	Vol. (Int.)	Local Bg. Corr. Vol.	Area	Rf	Density	Local Bg. Corr. Den.	% band purity	% lane purity	Mol. Wt.
1	10,600,281	1,671,904	288	0.119	36,806	5,805.224	12.651	2.404	250
2	10,309,708	1,762,987	288	0.171	35,797	6,121.484	13.34	2.338	150
3	10,913,384	1,888,168	324	0.234	33,683	5,827.682	14.287	2.475	100
4	8,618,821	893,519	288	0.28	29,926	3,102.497	6.761	1.954	75
5	15,950,991	2,562,569	540	0.409	29,538	4,745.499	19.391	3.617	50
6	13,362,613	1,905,079	468	0.5	28,552	4,070.683	14.415	3.03	37
7	9,028,649	404,079	360	0.614	25,079	1,122.444	3.058	2.047	25
8	7,652,581	780,239	280	0.675	27,330	2,786.568	5.904	1.735	20
9	17,863,554	1,347,023	648	0.748	27,567	2,078.74	10.193	4.05	15

Frame: 1
Channel: Chemi
Sensitivity: 100
Molecular Weight Analysis Regression Method : Point to Point

Lane 2 - Vehicle

#	Vol. (Int.)	Local Bg. Corr. Vol.	Area	Rf	Density	Local Bg. Corr. Den.	% band purity	% lane purity	Mol. Wt.	Rel. Quant. (w/ LB Corr. Vol.)
1	1,818,688	1,051,153	456	0.187	3,988.351	2,305.16	9.075	5.62	137.037	0.109
2	11,966,927	9,653,138	533	0.402	22,452	18,110	83.343	36.978	51.364	1
3	1,363,452	241,265	546	0.456	2,497.165	441.878	2.083	4.213	43.333	0.025
4	1,593,868	636,882	546	0.514	2,919.172	1,166.451	5.499	4.925	35.531	0.066

Lane 3 - 5uM Ionomycin

#	Vol. (Int.)	Local Bg. Corr. Vol.	Area	Rf	Density	Local Bg. Corr. Den.	% band purity	% lane purity	Mol. Wt.	Rel. Quant. (w/ LB Corr. Vol.)
1	2,404,950	1,603,816	385	0.192	6,246.623	4,165.757	10.822	5.946	133.333	0.166

#	Vol. (Int.)	Local Bg. Corr. Vol.	Area	Rf	Density	Local Bg. Corr. Den.	% band purity	% lane purity	Mol. Wt.	Rel. Quant. (w/ LB Corr. Vol.)
2	14,513,610	11,676,486	480	0.407	30,236	24,326	78.786	35.884	50.455	1.21
3	1,819,973	318,884	560	0.46	3,249.952	569.436	2.152	4.5	42.667	0.033
4	2,744,056	1,221,303	720	0.521	3,811.189	1,696.255	8.241	6.784	34.796	0.127

Lane 4 - 2uM Thapsigargin

#	Vol. (Int.)	Local Bg. Corr. Vol.	Area	Rf	Density	Local Bg. Corr. Den.	% band purity	% lane purity	Mol. Wt.	Rel. Quant. (w/ LB Corr. Vol.)
1	2,583,047	1,890,436	407	0.187	6,346.553	4,644.808	12.399	6.335	137.037	0.196
2	14,028,753	11,287,847	533	0.407	26,320	21,177	74.035	34.408	50.455	1.169
3	1,999,584	304,468	640	0.46	3,124.35	475.732	1.997	4.904	42.667	0.032
4	3,275,418	1,763,931	615	0.523	5,325.883	2,868.181	11.569	8.034	34.551	0.183

Lane 5 - t = 0

#	Vol. (Int.)	Local Bg. Corr. Vol.	Area	Rf	Density	Local Bg. Corr. Den.	% band purity	% lane purity	Mol. Wt.	Rel. Quant. (w/ LB Corr. Vol.)
1	4,100,925	3,578,845	380	0.187	10,791	9,418.015	19.731	10.352	137.037	0.371
2	14,256,300	12,264,113	520	0.407	27,415	23,584	67.615	35.989	50.455	1.27
3	2,277,209	625,026	880	0.465	2,587.738	710.257	3.446	5.749	42	0.065
4	3,263,836	1,670,219	1,050	0.528	3,108.415	1,590.685	9.208	8.239	34.061	0.173

iBright™ Image Analysis Report

Katarina+ Chang

19 November 2022

pMYPT1 CHEMI_01282022_124830

Date:	28 January 2022 12:48:30PM
Mode:	Chemi Blots
Notes:	
Model:	FL1500
Instrument name:	2462619090234
Serial No:	2462619090234
Firmware version:	1.6.0
iBA version:	5.0
Image size:	563px X 450px
Image area:	112.7mm X 90.16mm
Optical Zoom:	2x
Digital Zoom:	1.2x
Focus level:	455
Resolution:	5 x 5
Exposure time:	28500 ms
Exposure mode:	Normal

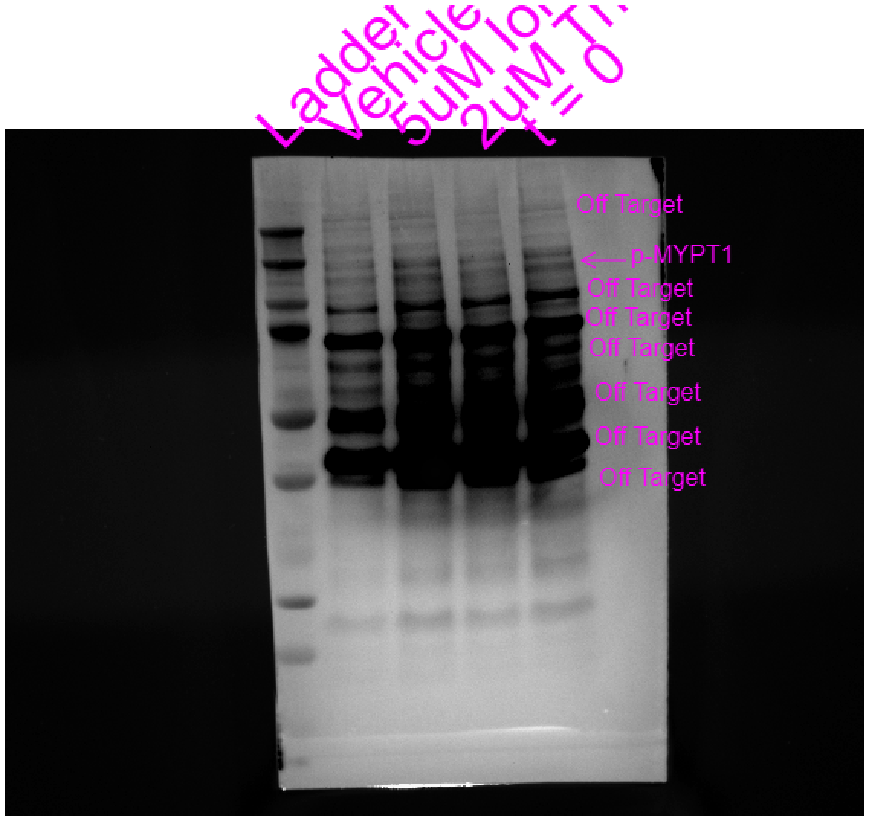

pMYPT1 CHEMI_01282022_124830

Date:	28 January 2022 12:48:30PM
Mode:	Chemi Blots
Notes:	
Model:	FL1500
Instrument name:	2462619090234
Serial No:	2462619090234
Firmware version:	1.6.0
iBA version:	5.0
Image size:	563px X 450px
Image area:	112.7mm X 90.16mm
Optical Zoom:	2x
Digital Zoom:	1.2x
Focus level:	455
Resolution:	5 x 5
Exposure time:	28500 ms
Exposure mode:	Normal

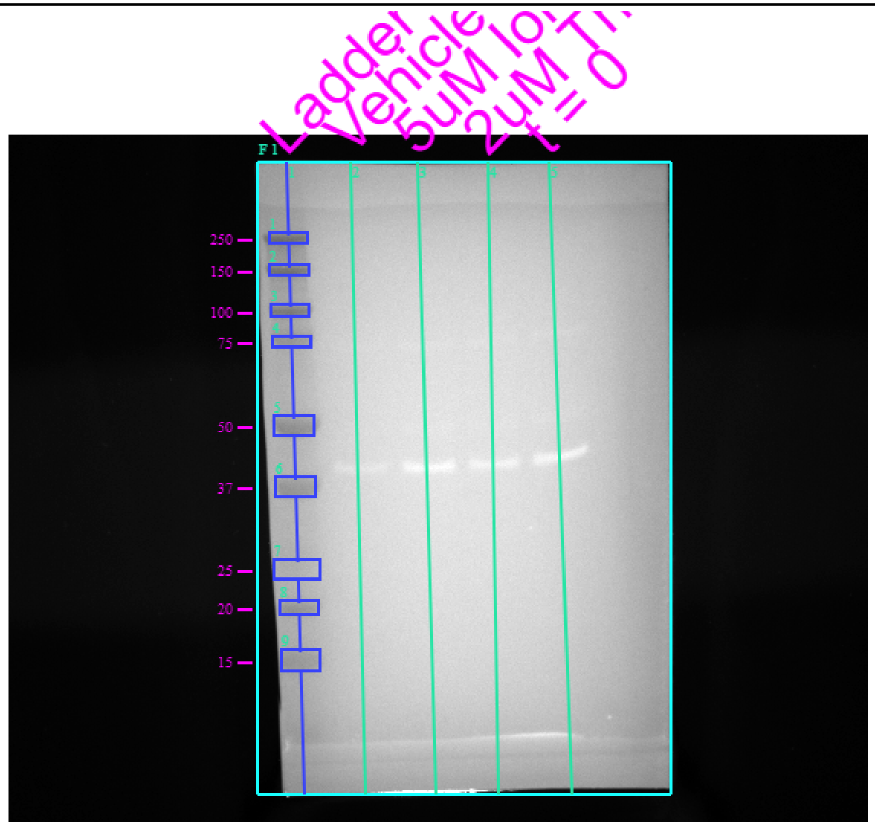

pMYPT1 CHEMI_01282022_124830

Date:	28 January 2022 12:48:30PM
Mode:	Chemi Blots
Notes:	
Model:	FL1500
Instrument name:	2462619090234
Serial No:	2462619090234
Firmware version:	1.6.0
iBA version:	5.0
Image size:	563px X 450px
Image area:	112.7mm X 90.16mm
Optical Zoom:	2x
Digital Zoom:	1.2x
Focus level:	455
Resolution:	5 x 5
Exposure time:	28500 ms
Exposure mode:	Normal

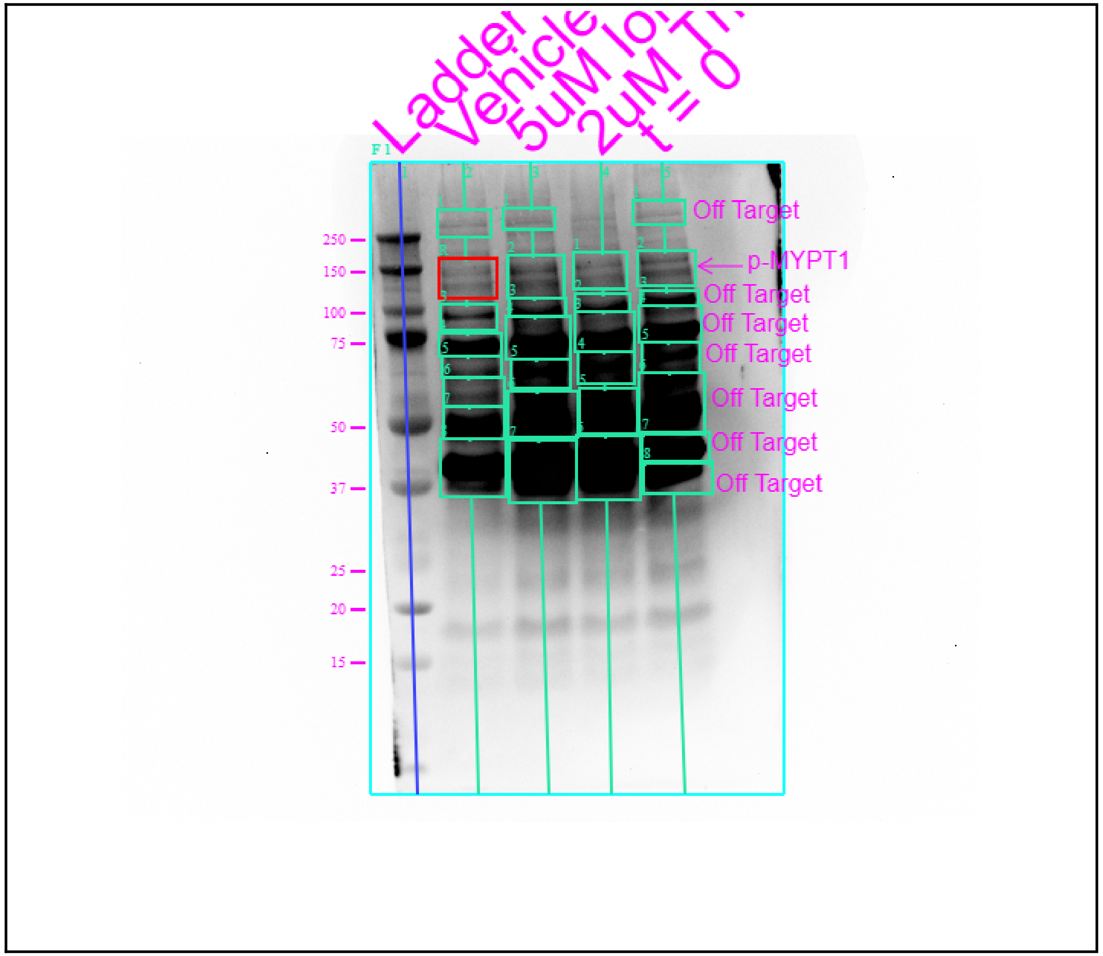

LANE AND BAND ANALYSIS DATA TABLE

pMYPT1 CHEMI_01282022_124830

Frame: 1
Channel: Membrane
Sensitivity: 100
Molecular Weight Analysis Regression Method : Point to Point

Lane 1 - Ladder

#	Vol. (Int.)	Local Bg. Corr. Vol.	Area	Rf	Density	Local Bg. Corr. Den.	% band purity	% lane purity	Mol. Wt.
1	8,065,424	1,264,018	208	0.118	38,776	6,077.013	14.782	2.5	250
2	8,186,060	1,392,926	216	0.169	37,898	6,448.733	16.289	2.538	150
3	8,494,906	1,454,448	234	0.234	36,303	6,215.59	17.009	2.633	100
4	6,777,436	679,008	208	0.283	32,583	3,264.464	7.941	2.101	75
5	12,482,780	1,890,897	378	0.415	33,023	5,002.374	22.113	3.869	50
6	12,093,174	1,469,762	378	0.512	31,992	3,888.26	17.188	3.749	37
7	12,324,718	NA	434	0.643	28,397	NA	NA	3.82	25
8	8,415,926	390,926	260	0.703	32,368	1,503.563	4.572	2.609	20
9	12,620,727	9,159.977	390	0.787	32,360	23.487	0.107	3.912	15

Frame: 1
Channel: Chemi
Sensitivity: 100
Molecular Weight Analysis Regression Method : Point to Point

Lane 2 - Vehicle

#	Vol. (Int.)	Local Bg. Corr. Vol.	Area	Rf	Density	Local Bg. Corr. Den.	% band purity	% lane purity	Mol. Wt.	Rel. Quant. (w/ LB Corr. Vol.)
1	18,951,967	2,614,718	684	0.097	27,707	3,822.687	3.477	6.576	NA	0.443
2	36,468,447	5,903,296	1,053	0.184	34,632	5,606.17	7.85	12.654	138.889	1
3	29,972,964	6,516,139	684	0.244	43,820	9,526.519	8.665	10.4	95	1.104
4	38,106,895	12,761,553	672	0.287	56,706	18,990	16.969	13.223	74.091	2.162
5	30,019,095	3,334,566	600	0.324	50,031	5,557.611	4.434	10.416	67.273	0.565
6	39,664,530	3,917,932	820	0.362	48,371	4,777.967	5.21	13.763	60	0.664
7	53,622,384	13,975,060	902	0.411	59,448	15,493	18.583	18.607	50.909	2.367
8	95,969,791	26,180,487	1,716	0.483	55,926	15,256	34.813	33.301	40.9	4.435

Lane 3 - 5uM Ionomycin

#	Vol. (Int.)	Local Bg. Corr. Vol.	Area	Rf	Density	Local Bg. Corr. Den.	% band purity	% lane purity	Mol. Wt.	Rel. Quant. (w/ LB Corr. Vol.)
1	15,628,274	1,917,841	525	0.089	29,768	3,653.031	2.863	4.466	NA	0.325
2	47,776,041	10,543,283	1,102	0.181	43,353	9,567.408	15.737	13.652	140.741	1.786
3	27,205,433	6,299,366	494	0.229	55,071	12,751	9.402	7.774	103.704	1.067
4	73,311,318	13,274,456	1,247	0.278	58,790	10,645	19.813	20.948	77.5	2.249
5	49,404,226	7,836,560	800	0.333	61,755	9,795.7	11.697	14.117	65.455	1.327
6	91,123,295	9,305,289	1,457	0.399	62,541	6,386.609	13.889	26.038	53.182	1.576
7	116,812,279	17,820,428	1,890	0.488	61,805	9,428.798	26.599	33.378	40.25	3.019

Lane 4 - 2uM Thapsigargin

#	Vol. (Int.)	Local Bg. Corr. Vol.	Area	Rf	Density	Local Bg. Corr. Den.	% band purity	% lane purity	Mol. Wt.	Rel. Quant. (w/ LB Corr. Vol.)
1	34,937,413	5,837,772	900	0.171	38,819	6,486.414	11.896	10.224	148.148	0.989
2	25,026,571	5,874,990	481	0.222	52,030	12,214	11.972	7.324	109.259	0.995
3	61,621,105	9,797,535	1,080	0.268	57,056	9,071.792	19.966	18.032	82.5	1.66
4	51,499,565	7,752,790	836	0.324	61,602	9,273.673	15.799	15.071	67.273	1.313
5	77,742,357	6,830,093	1,209	0.394	64,303	5,649.374	13.919	22.75	54.091	1.157
6	115,699,569	12,978,664	1,849	0.483	62,574	7,019.289	26.448	33.858	40.9	2.199

Lane 5 - t = 0

#	Vol. (Int.)	Local Bg. Corr. Vol.	Area	Rf	Density	Local Bg. Corr. Den.	% band purity	% lane purity	Mol. Wt.	Rel. Quant. (w/ LB Corr. Vol.)
1	15,603,837	2,216,387	595	0.08	26,224	3,725.021	2.454	4.664	NA	0.375
2	37,344,233	9,256,489	936	0.167	39,897	9,889.412	10.249	11.162	154.762	1.568
3	23,786,717	7,208,406	429	0.215	55,446	16,802	7.981	7.11	114.815	1.221
4	58,977,041	13,578,439	1,050	0.256	56,168	12,931	15.034	17.628	88.75	2.3
5	49,160,525	10,093,606	819	0.309	60,025	12,324	11.176	14.694	70	1.71
6	112,786,907	25,143,619	1,848	0.382	61,031	13,605	27.84	33.712	56.364	4.259
7	60,689,261	10,854,898	990	0.452	61,302	10,964	12.019	18.14	45.125	1.839
8	53,702,478	11,963,936	1,012	0.498	53,065	11,822	13.247	16.052	38.95	2.027

iBright™ Image Analysis Report

Katarina+ Chang
19 November 2022

total MLC CHEMI_01312022_123004

Date:	31 January 2022 12:30:04PM
Mode:	Chemi Blots
Notes:	
Model:	FL1500
Instrument name:	2462619090234
Serial No:	2462619090234
Firmware version:	1.6.0
iBA version:	5.0
Image size:	615px X 491px
Image area:	112.7mm X 90.16mm
Optical Zoom:	2x
Digital Zoom:	1.1x
Focus level:	455
Resolution:	5 x 5
Exposure time:	8000 ms
Exposure mode:	Normal

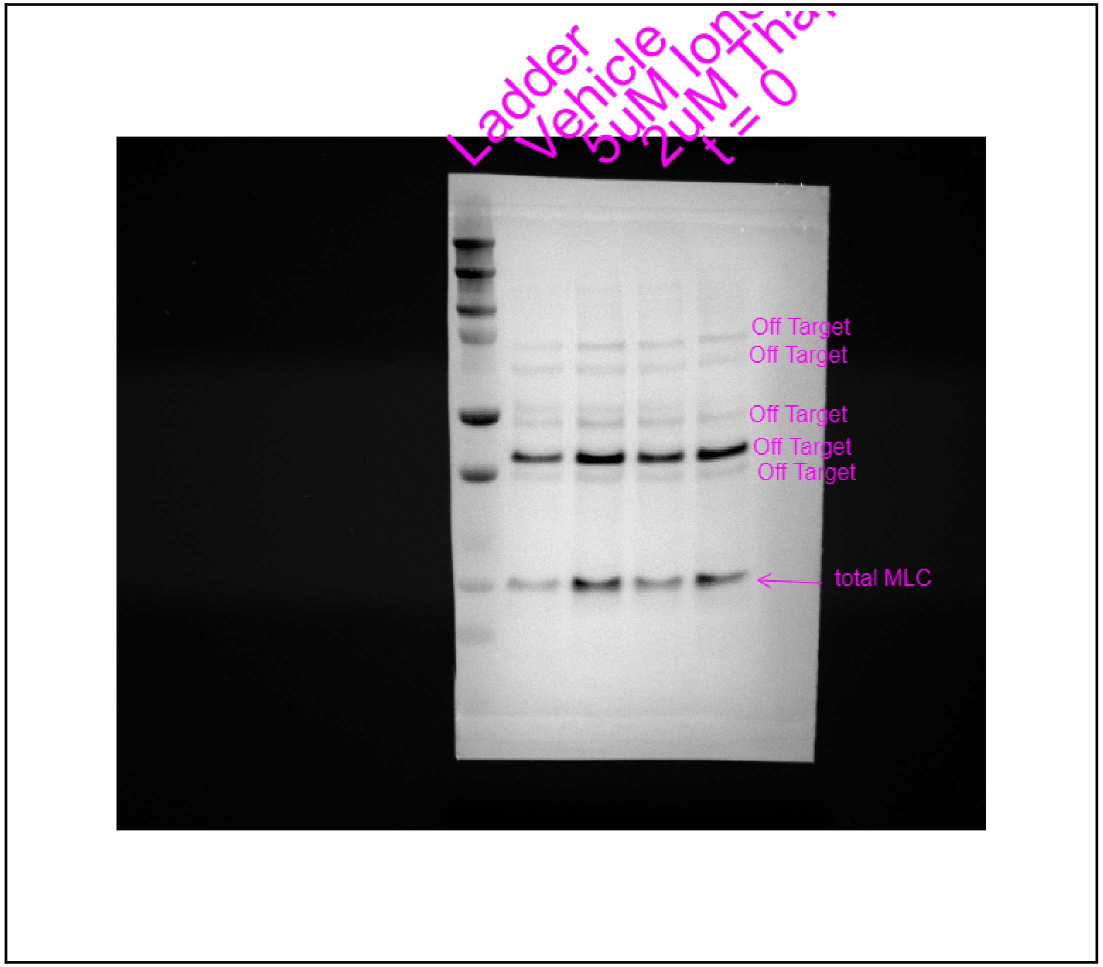

total MLC CHEMI_01312022_123004

Date:	31 January 2022 12:30:04PM
Mode:	Chemi Blots
Notes:	
Model:	FL1500
Instrument name:	2462619090234
Serial No:	2462619090234
Firmware version:	1.6.0
iBA version:	5.0
Image size:	615px X 491px
Image area:	112.7mm X 90.16mm
Optical Zoom:	2x
Digital Zoom:	1.1x
Focus level:	455
Resolution:	5 x 5
Exposure time:	8000 ms
Exposure mode:	Normal

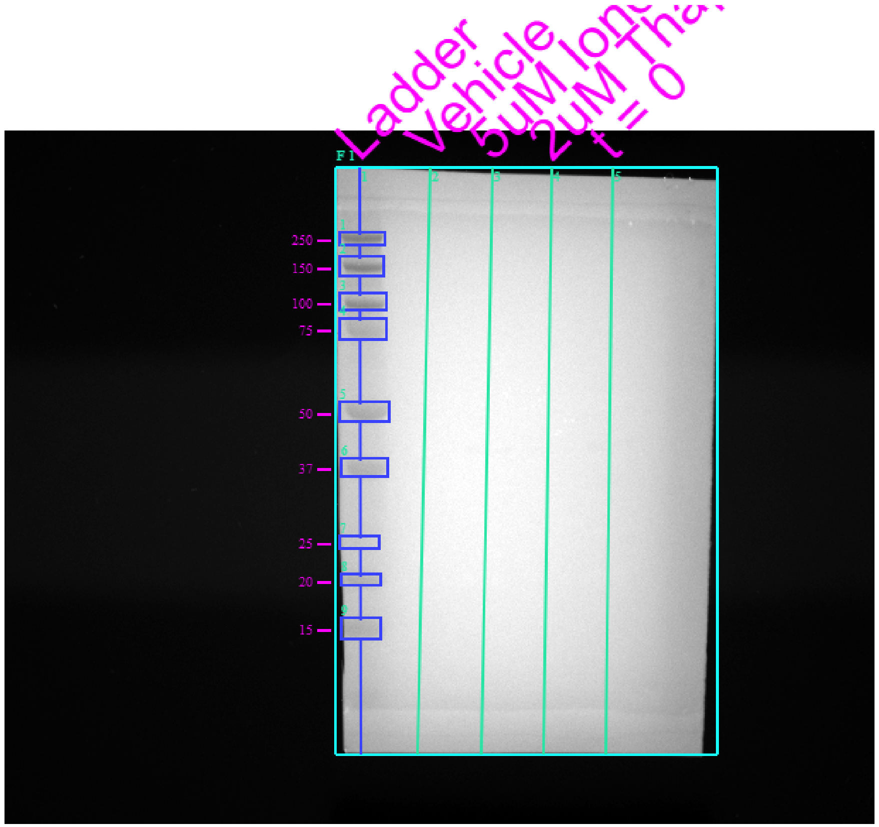

total MLC CHEMI_01312022_123004

Date: 31 January 2022 12:30:04PM
Mode: Chemi Blots
Notes:
Model: FL1500
Instrument name: 2462619090234
Serial No: 2462619090234
Firmware version: 1.6.0
iBA version: 5.0
Image size: 615px X 491px
Image area: 112.7mm X 90.16mm
Optical Zoom: 2x
Digital Zoom: 1.1x
Focus level: 455
Resolution: 5 x 5
Exposure time: 8000 ms
Exposure mode: Normal

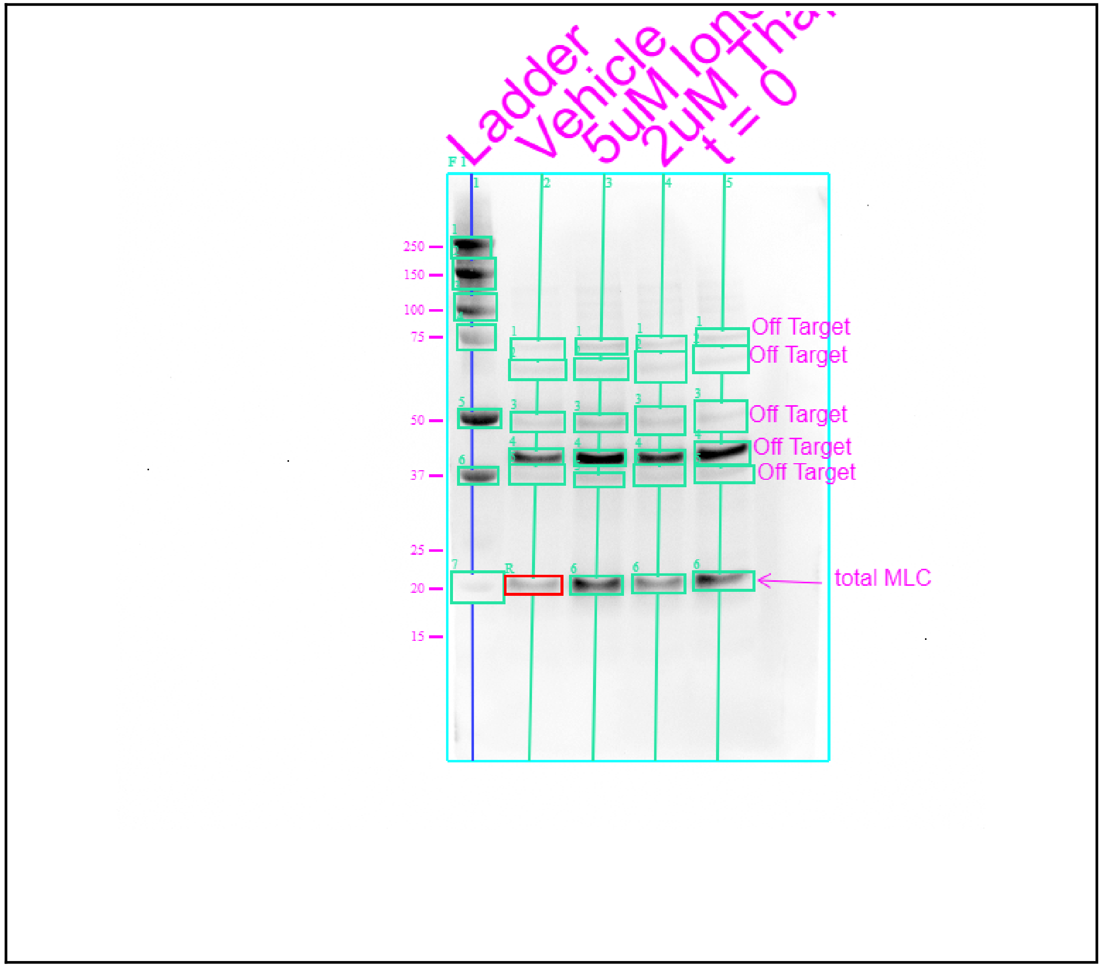

LANE AND BAND ANALYSIS DATA TABLE

total MLC CHEMI_01312022_123004

Frame: 1
Channel: Membrane
Sensitivity: 100
Molecular Weight Analysis Regression Method : Point to Point

Lane 1 - Ladder

#	Vol. (Int.)	Local Bg. Corr. Vol.	Area	Rf	Density	Local Bg. Corr. Den.	% band purity	% lane purity	Mol. Wt.
1	11,981,551	1,001,228	330	0.12	36,307	3,034.027	24.9	3.352	250
2	16,324,742	765,328	480	0.168	34,009	1,594.433	19.034	4.567	150
3	14,326,751	902,932	442	0.228	32,413	2,042.835	22.456	4.008	100
4	16,215,389	131,678	544	0.274	29,807	242.056	3.275	4.537	75
5	16,012,862	313,012	540	0.416	29,653	579.652	7.785	4.48	50
6	13,947,446	126,451	476	0.51	29,301	265.654	3.145	3.902	37
7	8,109,728	79,143	290	0.637	27,964	272.909	1.968	2.269	25
8	8,161,067	118,289	261	0.702	31,268	453.216	2.942	2.283	20
9	15,142,093	582,870	464	0.784	32,633	1,256.187	14.496	4.237	15

Frame: 1
Channel: Chemi
Sensitivity: 100
Molecular Weight Analysis Regression Method : Point to Point

Lane 1 - Ladder

#	Vol. (Int.)	Local Bg. Corr. Vol.	Area	Rf	Density	Local Bg. Corr. Den.	% band purity	% lane purity	Mol. Wt.	Rel. Quant. (w/ LB Corr. Vol.)
1	13,623,436	9,478,274	464	0.125	29,360	20,427	22.396	14.709	240	3.596
2	17,218,458	10,792,039	713	0.171	24,149	15,136	25.5	18.591	148	4.094
3	9,850,922	5,615,326	620	0.226	15,888	9,056.978	13.268	10.636	102	2.13
4	5,159,410	2,520,789	532	0.279	9,698.139	4,738.327	5.956	5.571	74.153	0.956
5	11,020,542	8,481,416	434	0.416	25,392	19,542	20.04	11.899	50	3.218
6	7,148,708	4,939,462	377	0.514	18,962	13,102	11.671	7.719	36.547	1.874
7	2,710,529	494,735	874	0.704	3,101.292	566.059	1.169	2.927	19.853	0.188

Lane 2 - Vehicle

#	Vol. (Int.)	Local Bg. Corr. Vol.	Area	Rf	Density	Local Bg. Corr. Den.	% band purity	% lane purity	Mol. Wt.	Rel. Quant. (w/ LB Corr. Vol.)
1	2,726,958	354,489	624	0.298	4,370.125	568.092	3.154	5.695	70.763	0.134
2	3,204,735	736,662	615	0.334	5,210.951	1,197.825	6.554	6.693	64.407	0.279
3	4,185,563	737,149	585	0.423	7,154.809	1,260.084	6.558	8.741	49	0.28
4	10,104,074	6,757,005	468	0.481	21,589	14,438	60.118	21.102	41	2.564
5	4,579,596	18,525	600	0.512	7,632.66	30.876	0.165	9.564	36.774	0.007
6	5,137,181	2,635,829	574	0.7	8,949.793	4,592.037	23.451	10.729	20.185	1

Lane 3 - 5uM Ionomycin

#	Vol. (Int.)	Local Bg. Corr. Vol.	Area	Rf	Density	Local Bg. Corr. Den.	% band purity	% lane purity	Mol. Wt.	Rel. Quant. (w/ LB Corr. Vol.)
1	3,013,282	1,052,570	444	0.293	6,786.671	2,370.655	4.442	4.35	71.61	0.399
2	3,939,079	979,025	624	0.332	6,312.627	1,568.951	4.132	5.687	64.831	0.371
3	5,078,309	1,435,698	532	0.423	9,545.694	2,698.681	6.059	7.331	49	0.545
4	15,499,371	11,392,985	444	0.483	34,908	25,659	48.079	22.376	40.667	4.322
5	3,657,879	710,791	396	0.522	9,237.068	1,794.929	3	5.281	35.868	0.27
6	11,704,257	8,125,369	532	0.7	22,000	15,273	34.289	16.897	20.185	3.083

Lane 4 - 2uM Thapsigargin

#	Vol. (Int.)	Local Bg. Corr. Vol.	Area	Rf	Density	Local Bg. Corr. Den.	% band purity	% lane purity	Mol. Wt.	Rel. Quant. (w/ LB Corr. Vol.)
1	2,393,552	745,288	432	0.288	5,540.63	1,725.205	5.161	3.975	72.458	0.283
2	4,323,522	616,444	851	0.329	5,080.519	724.377	4.269	7.181	65.254	0.234
3	5,949,802	1,546,729	756	0.421	7,870.108	2,045.939	10.711	9.882	49.333	0.587
4	10,721,643	7,387,362	396	0.483	27,074	18,654	51.158	17.807	40.667	2.803
5	5,110,311	38,427	592	0.512	8,632.282	64.912	0.266	8.487	36.774	0.015
6	7,167,834	4,105,902	532	0.697	13,473	7,717.862	28.434	11.905	20.37	1.558

Lane 5 - t = 0

#	Vol. (Int.)	Local Bg. Corr. Vol.	Area	Rf	Density	Local Bg. Corr. Den.	% band purity	% lane purity	Mol. Wt.	Rel. Quant. (w/ LB Corr. Vol.)
1	2,265,337	860,799	494	0.279	4,585.702	1,742.51	4.095	3.9	74.153	0.327
2	2,970,578	538,192	800	0.315	3,713.223	672.74	2.56	5.114	67.797	0.204
3	4,970,605	1,127,605	874	0.413	5,687.191	1,290.166	5.364	8.556	50.424	0.428
4	15,925,119	11,346,363	680	0.476	23,419	16,685	53.978	27.414	41.667	4.305

#	Vol. (Int.)	Local Bg. Corr. Vol.	Area	Rf	Density	Local Bg. Corr. Den.	% band purity	% lane purity	Mol. Wt.	Rel. Quant. (w/ LB Corr. Vol.)
5	4,119,236	480,323	559	0.512	7,368.937	859.255	2.285	7.091	36.774	0.182
6	10,132,900	6,667,010	616	0.692	16,449	10,823	31.717	17.443	20.741	2.529

iBright™ Image Analysis Report

Katarina+ Chang

19 November 2022

GAPDH CHEMI_02012022_115716

Date:	1 February 2022 11:57:16AM
Mode:	Chemi Blots
Notes:	
Model:	FL1500
Instrument name:	2462619090234
Serial No:	2462619090234
Firmware version:	1.6.0
iBA version:	5.0
Image size:	615px X 491px
Image area:	112.7mm X 90.16mm
Optical Zoom:	2x
Digital Zoom:	1.1x
Focus level:	455
Resolution:	5 x 5
Exposure time:	15000 ms
Exposure mode:	Normal

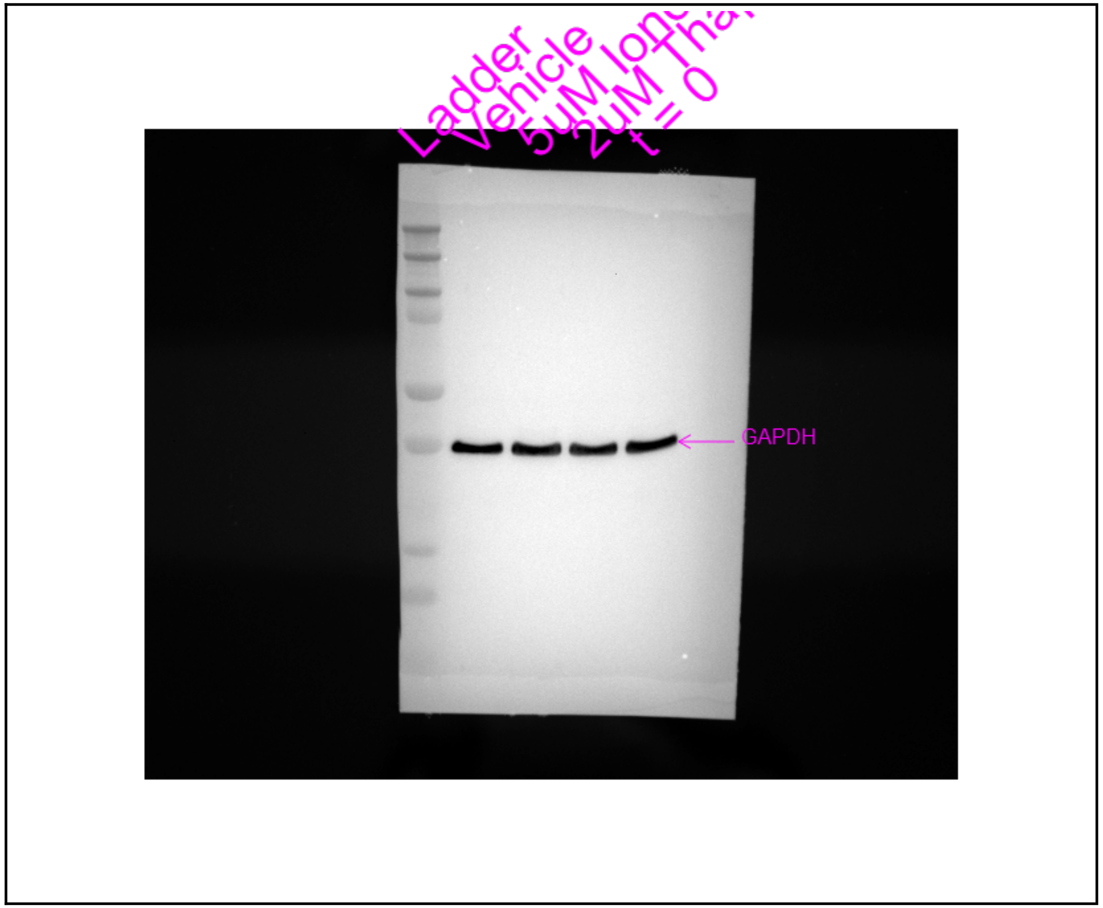

GAPDH CHEMI_02012022_115716

Date:	1 February 2022 11:57:16AM
Mode:	Chemi Blots
Notes:	
Model:	FL1500
Instrument name:	2462619090234
Serial No:	2462619090234
Firmware version:	1.6.0
iBA version:	5.0
Image size:	615px X 491px
Image area:	112.7mm X 90.16mm
Optical Zoom:	2x
Digital Zoom:	1.1x
Focus level:	455
Resolution:	5 x 5
Exposure time:	15000 ms
Exposure mode:	Normal

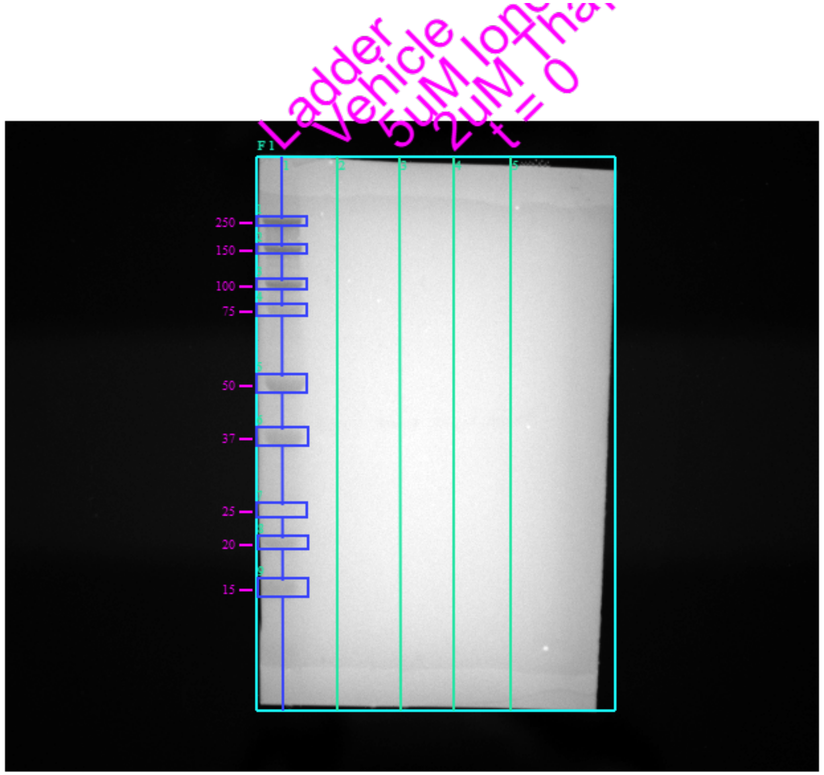

GAPDH CHEMI_02012022_115716

Date:	1 February 2022 11:57:16AM
Mode:	Chemi Blots
Notes:	
Model:	FL1500
Instrument name:	2462619090234
Serial No:	2462619090234
Firmware version:	1.6.0
iBA version:	5.0
Image size:	615px X 491px
Image area:	112.7mm X 90.16mm
Optical Zoom:	2x
Digital Zoom:	1.1x
Focus level:	455
Resolution:	5 x 5
Exposure time:	15000 ms
Exposure mode:	Normal

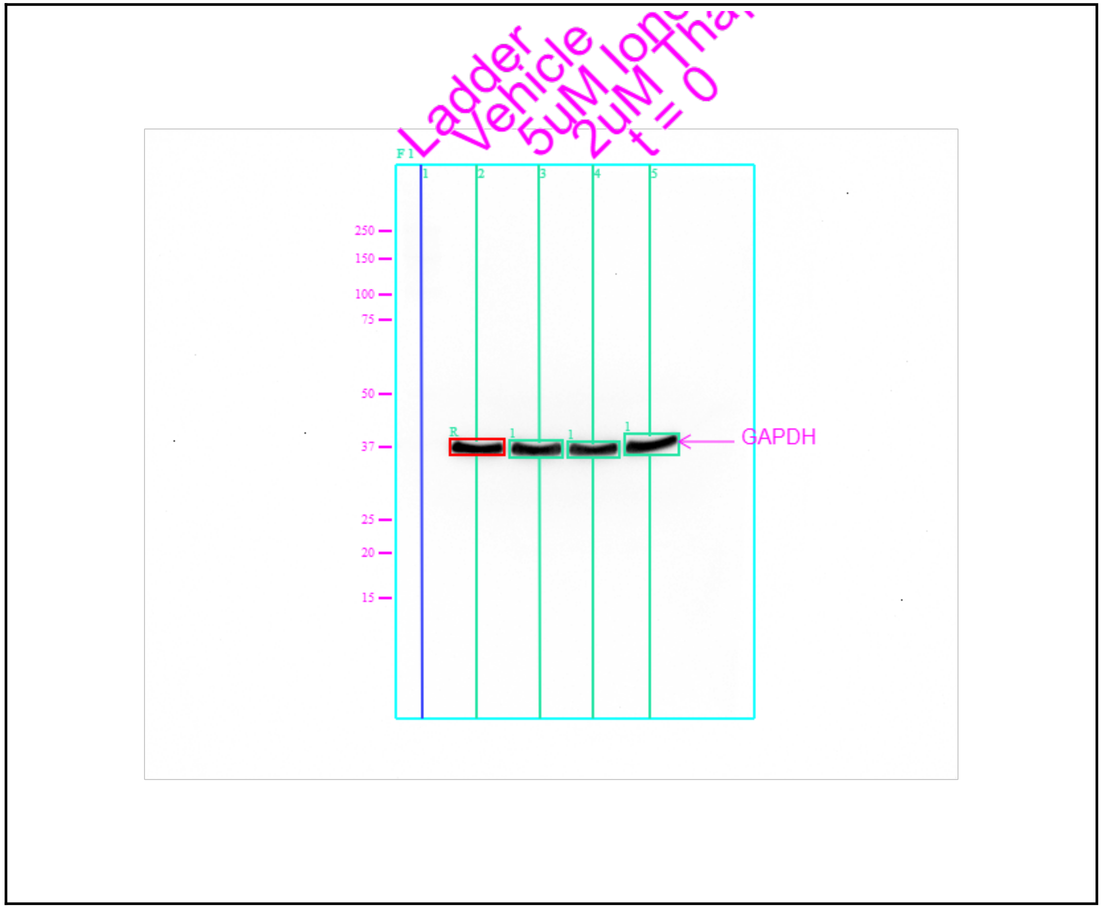

LANE AND BAND ANALYSIS DATA TABLE

GAPDH CHEMI_02012022_115716

Frame: 1
Channel: Membrane
Sensitivity: 100
Molecular Weight Analysis Regression Method : Point to Point

Lane 1 - Ladder

#	Vol. (Int.)	Local Bg. Corr. Vol.	Area	Rf	Density	Local Bg. Corr. Den.	% band purity	% lane purity	Mol. Wt.
1	12,056,418	726,571	312	0.115	38,642	2,328.754	15.199	2.354	250
2	11,632,778	697,511	312	0.165	37,284	2,235.612	14.591	2.271	150
3	12,251,217	485,986	351	0.23	34,903	1,384.576	10.166	2.392	100
4	12,467,863	163,304	390	0.275	31,968	418.729	3.416	2.434	75
5	18,609,280	294,503	585	0.409	31,810	503.425	6.161	3.633	50
6	18,742,668	464,917	600	0.505	31,237	774.862	9.726	3.659	37
7	13,913,780	550,386	468	0.636	29,730	1,176.041	11.514	2.716	25
8	13,627,825	485,666	429	0.696	31,766	1,132.089	10.16	2.66	20
9	19,366,957	911,505	585	0.778	33,105	1,558.129	19.068	3.781	15

Frame: 1
Channel: Chemi
Sensitivity: 100
Molecular Weight Analysis Regression Method : Point to Point

Lane 2 - Vehicle

#	Vol. (Int.)	Local Bg. Corr. Vol.	Area	Rf	Density	Local Bg. Corr. Den.	% band purity	% lane purity	Mol. Wt.	Rel. Quant. (w/ LB Corr. Vol.)
1	15,011,578	13,135,114	546	0.51	27,493	24,056	100	66.245	36.564	1

Lane 3 - 5uM Ionomycin

#	Vol. (Int.)	Local Bg. Corr. Vol.	Area	Rf	Density	Local Bg. Corr. Den.	% band purity	% lane purity	Mol. Wt.	Rel. Quant. (w/ LB Corr. Vol.)
1	14,208,313	11,811,529	574	0.512	24,753	20,577	100	62.807	36.345	0.899

Lane 4 - 2uM Thapsigargin

#	Vol. (Int.)	Local Bg. Corr. Vol.	Area	Rf	Density	Local Bg. Corr. Den.	% band purity	% lane purity	Mol. Wt.	Rel. Quant. (w/ LB Corr. Vol.)

#	Vol. (Int.)	Local Bg. Corr. Vol.	Area	Rf	Density	Local Bg. Corr. Den.	% band purity	% lane purity	Mol. Wt.	Rel. Quant. (w/ LB Corr. Vol.)
1	12,615,392	10,274,648	520	0.514	24,260	19,758	100	58.914	36.127	0.782

Lane 5 - t = 0

#	Vol. (Int.)	Local Bg. Corr. Vol.	Area	Rf	Density	Local Bg. Corr. Den.	% band purity	% lane purity	Mol. Wt.	Rel. Quant. (w/ LB Corr. Vol.)
1	15,379,993	13,742,027	714	0.505	21,540	19,246	100	70.988	37	1.046
