# Supplementary material for: Elevation of Cytoplasmic Calcium Suppresses Microtentacle Formation and Function in Breast Tumor Cells
Source: Cancers (Basel). 2023 Jan 31;15(3):884. doi: 10.3390/cancers15030884 (PMC9913253; doi:10.3390/cancers15030884)
Supplement: Supplementary file 1 [file cancers-15-00884-s001.zip › cancers-2080686-Supplementary/File S1_ Original Blots/Original Immunoblot Images MDAMB436 Biological Replicate 3.pdf]

iBright™ Image Analysis Report

Katarina+ Chang

19 November 2022

GAPDH CHEMI_02242022_153141

Date:	24 February 2022 03:31:41PM
Mode:	Chemi Blots
Notes:	
Model:	FL1500
Instrument name:	2462619090234
Serial No:	2462619090234
Firmware version:	1.6.0
iBA version:	5.0
Image size:	676px X 540px
Image area:	118.63mm X 94.91mm
Optical Zoom:	1.9x
Digital Zoom:	1x
Focus level:	430
Resolution:	5 x 5
Exposure time:	23885 ms
Exposure mode:	Normal

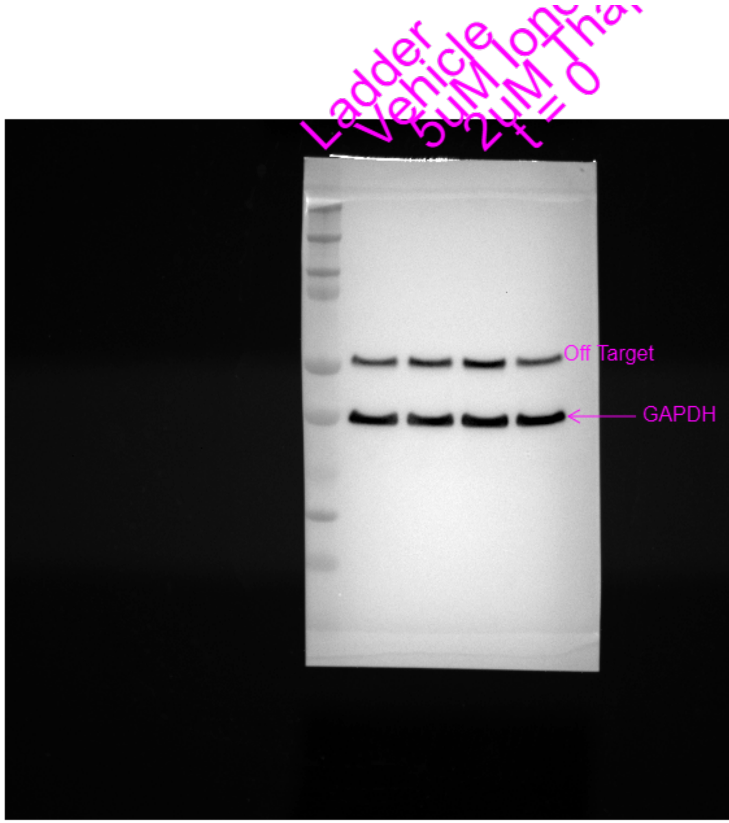

GAPDH CHEMI_02242022_153141

Date:	24 February 2022 03:31:41PM
Mode:	Chemi Blots
Notes:	
Model:	FL1500
Instrument name:	2462619090234
Serial No:	2462619090234
Firmware version:	1.6.0
iBA version:	5.0
Image size:	676px X 540px
Image area:	118.63mm X 94.91mm
Optical Zoom:	1.9x
Digital Zoom:	1x
Focus level:	430
Resolution:	5 x 5
Exposure time:	23885 ms
Exposure mode:	Normal

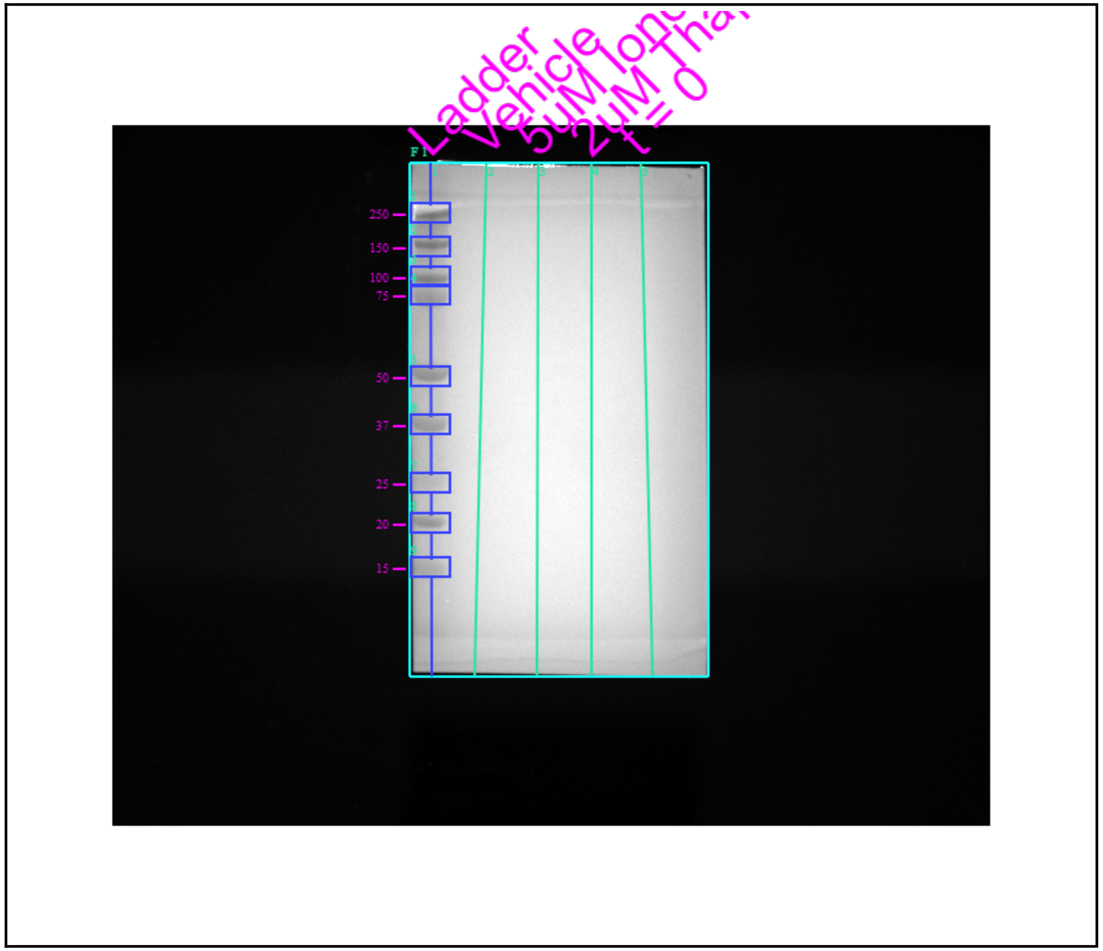

GAPDH CHEMI_02242022_153141

Date:	24 February 2022 03:31:41PM
Mode:	Chemi Blots
Notes:	
Model:	FL1500
Instrument name:	2462619090234
Serial No:	2462619090234
Firmware version:	1.6.0
iBA version:	5.0
Image size:	676px X 540px
Image area:	118.63mm X 94.91mm
Optical Zoom:	1.9x
Digital Zoom:	1x
Focus level:	430
Resolution:	5 x 5
Exposure time:	23885 ms
Exposure mode:	Normal

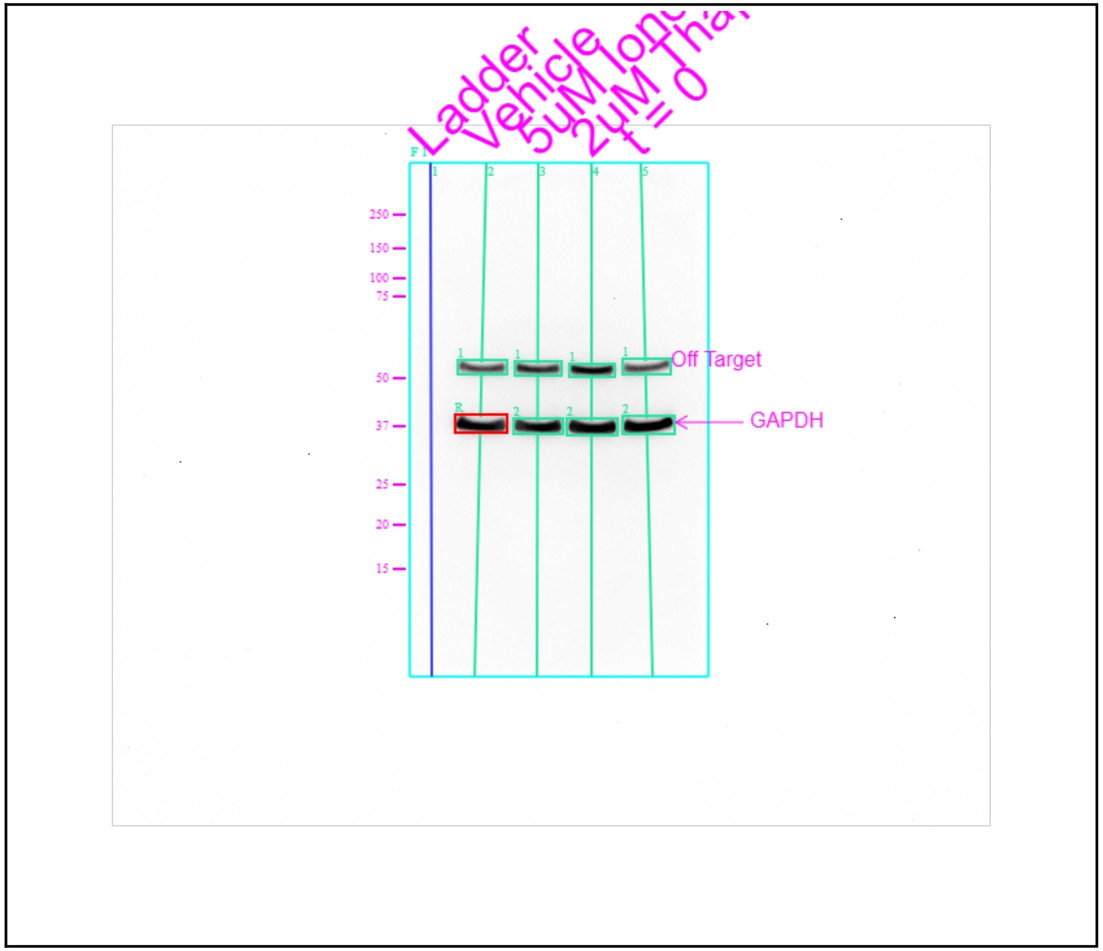

LANE AND BAND ANALYSIS DATA TABLE

GAPDH CHEMI_02242022_153141

Frame: 1
Channel: Membrane
Sensitivity: 100
Molecular Weight Analysis Regression Method : Point to Point

Lane 1 - Ladder

#	Vol. (Int.)	Local Bg. Corr. Vol.	Area	Rf	Density	Local Bg. Corr. Den.	% band purity	% lane purity	Mol. Wt.
1	16,418,318	320,600	496	0.096	33,101	646.371	9.93	3.474	250
2	17,388,210	535,798	496	0.162	35,056	1,080.239	16.596	3.68	150
3	16,883,909	446,122	496	0.22	34,040	899.44	13.818	3.573	100
4	16,459,284	20,927	496	0.255	33,184	42.192	0.648	3.483	75
5	16,150,223	475,818	496	0.414	32,560	959.311	14.738	3.418	50
6	15,836,485	162,788	496	0.508	31,928	328.204	5.042	3.351	37
7	14,789,712	251,424	496	0.621	29,817	506.904	7.787	3.13	25
8	15,879,435	517,395	496	0.699	32,014	1,043.136	16.026	3.36	20
9	15,873,094	497,698	496	0.785	32,002	1,003.425	15.415	3.359	15

Frame: 1
Channel: Chemi
Sensitivity: 100
Molecular Weight Analysis Regression Method : Point to Point

Lane 2 - Vehicle

#	Vol. (Int.)	Local Bg. Corr. Vol.	Area	Rf	Density	Local Bg. Corr. Den.	% band purity	% lane purity	Mol. Wt.	Rel. Quant. (w/ LB Corr. Vol.)
1	6,981,030	5,582,374	468	0.396	14,916	11,928	33.804	20.106	52.778	0.511
2	13,242,597	10,931,711	615	0.508	21,532	17,775	66.196	38.14	37	1

Lane 3 - 5uM Ionomycin

#	Vol. (Int.)	Local Bg. Corr. Vol.	Area	Rf	Density	Local Bg. Corr. Den.	% band purity	% lane purity	Mol. Wt.	Rel. Quant. (w/ LB Corr. Vol.)
1	7,857,057	6,226,993	444	0.399	17,696	14,024	40.106	21.446	52.381	0.57
2	11,899,522	9,299,294	507	0.513	23,470	18,341	59.894	32.48	36.467	0.851

Lane 4 - 2uM Thapsigargin

#	Vol. (Int.)	Local Bg. Corr. Vol.	Area	Rf	Density	Local Bg. Corr. Den.	% band purity	% lane purity	Mol. Wt.	Rel. Quant. (w/ LB Corr. Vol.)
1	8,678,107	7,076,940	396	0.404	21,914	17,871	40.137	21.712	51.587	0.647
2	13,545,547	10,555,208	560	0.513	24,188	18,848	59.863	33.889	36.467	0.966

Lane 5 - t = 0

#	Vol. (Int.)	Local Bg. Corr. Vol.	Area	Rf	Density	Local Bg. Corr. Den.	% band purity	% lane purity	Mol. Wt.	Rel. Quant. (w/ LB Corr. Vol.)
1	6,860,471	5,513,648	494	0.396	13,887	11,161	30.789	18.68	52.778	0.504
2	14,775,373	12,393,995	615	0.51	24,024	20,152	69.211	40.232	36.733	1.134

iBright™ Image Analysis Report

Katarina+ Chang

19 November 2022

MLCK_CHEMI_02222022_122503

Date:	22 February 2022 12:25:03PM
Mode:	Chemi Blots
Notes:	
Model:	FL1500
Instrument name:	2462619090234
Serial No:	2462619090234
Firmware version:	1.6.0
iBA version:	5.0
Image size:	676px X 540px
Image area:	112.7mm X 90.16mm
Optical Zoom:	2x
Digital Zoom:	1x
Focus level:	455
Resolution:	5 x 5
Exposure time:	60000 ms
Exposure mode:	Normal

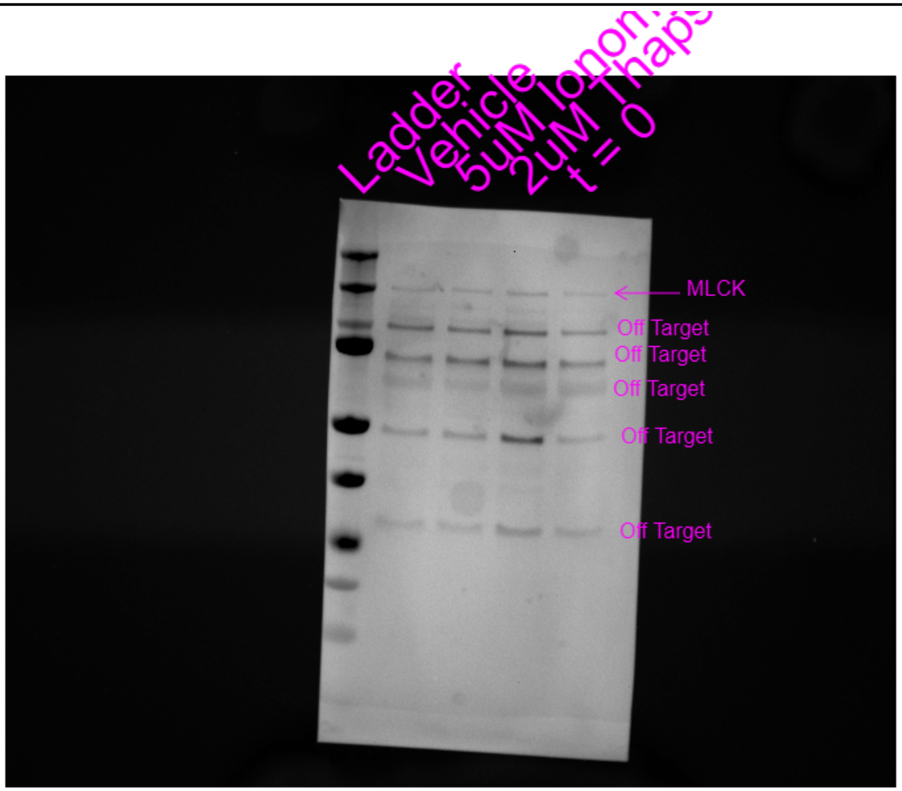

MLCK_CHEMI_02222022_122503

Date:	22 February 2022 12:25:03PM
Mode:	Chemi Blots
Notes:	
Model:	FL1500
Instrument name:	2462619090234
Serial No:	2462619090234
Firmware version:	1.6.0
iBA version:	5.0
Image size:	676px X 540px
Image area:	112.7mm X 90.16mm
Optical Zoom:	2x
Digital Zoom:	1x
Focus level:	455
Resolution:	5 x 5
Exposure time:	60000 ms
Exposure mode:	Normal

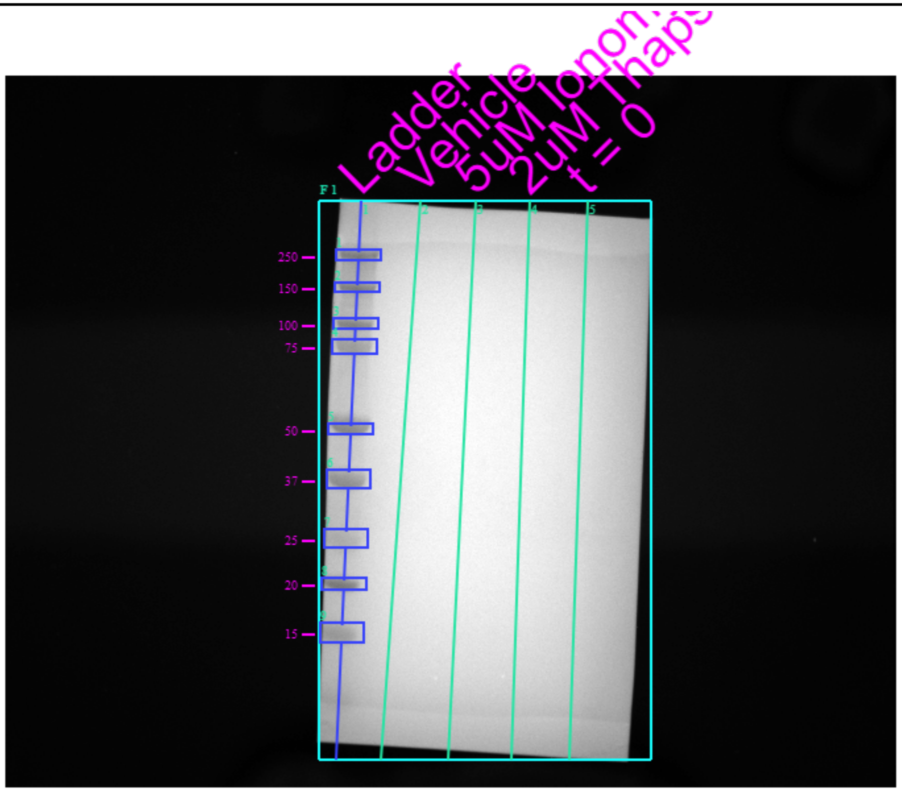

MLCK_CHEMI_02222022_122503

Date:	22 February 2022 12:25:03PM
Mode:	Chemi Blots
Notes:	
Model:	FL1500
Instrument name:	2462619090234
Serial No:	2462619090234
Firmware version:	1.6.0
iBA version:	5.0
Image size:	676px X 540px
Image area:	112.7mm X 90.16mm
Optical Zoom:	2x
Digital Zoom:	1x
Focus level:	455
Resolution:	5 x 5
Exposure time:	60000 ms
Exposure mode:	Normal

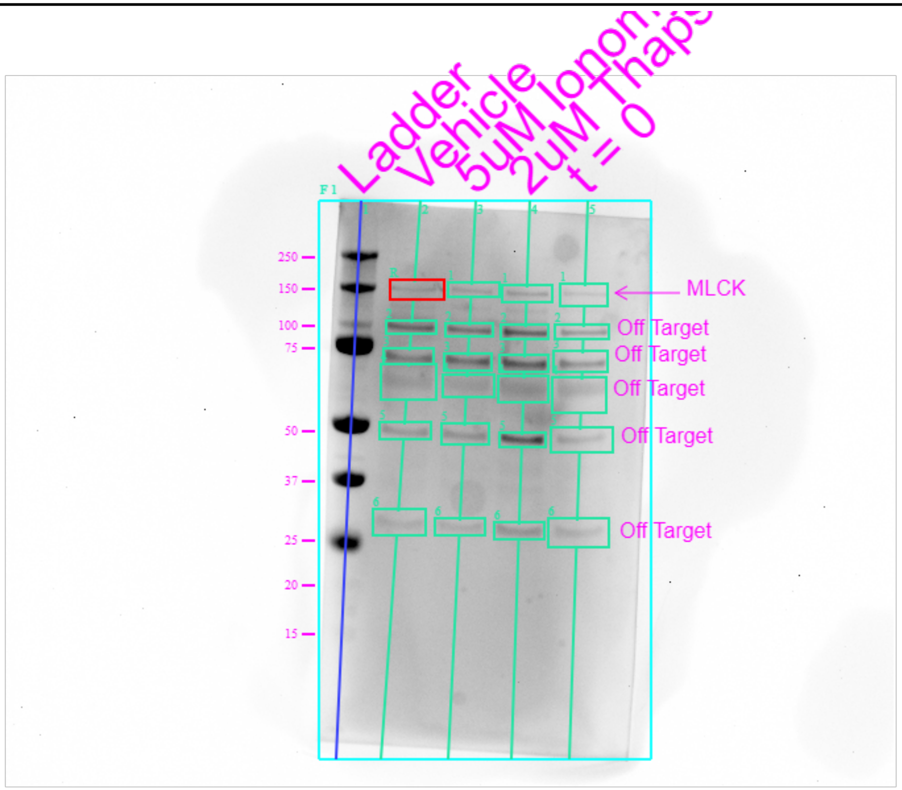

LANE AND BAND ANALYSIS DATA TABLE

MLCK_CHEMI_02222022_122503

Frame: 1
Channel: Membrane
Sensitivity: 100
Molecular Weight Analysis Regression Method : Point to Point

Lane 1 - Ladder

#	Vol. (Int.)	Local Bg. Corr. Vol.	Area	Rf	Density	Local Bg. Corr. Den.	% band purity	% lane purity	Mol. Wt.
1	11,641,272	509,420	315	0.097	36,956	1,617.207	8.597	2.47	250
2	10,012,990	737,678	280	0.153	35,760	2,634.567	12.449	2.124	150
3	10,734,583	860,767	315	0.219	34,078	2,732.596	14.526	2.278	100
4	13,320,836	412,434	420	0.259	31,716	981.988	6.96	2.826	75
5	10,708,652	732,087	315	0.408	33,995	2,324.086	12.354	2.272	50
6	16,717,929	686,628	510	0.498	32,780	1,346.33	11.587	3.547	37
7	15,788,704	292,662	510	0.604	30,958	573.848	4.939	3.35	25
8	12,739,157	1,177,697	350	0.684	36,397	3,364.851	19.874	2.703	20
9	18,319,024	516,328	544	0.771	33,674	949.133	8.713	3.887	15

Frame: 1
Channel: Chemi
Sensitivity: 100
Molecular Weight Analysis Regression Method : Point to Point

Lane 2 - Vehicle

#	Vol. (Int.)	Local Bg. Corr. Vol.	Area	Rf	Density	Local Bg. Corr. Den.	% band purity	% lane purity	Mol. Wt.	Rel. Quant. (w/ LB Corr. Vol.)
1	16,285,486	1,014,709	672	0.158	24,234	1,509.985	9.559	5.993	146.429	1
2	13,708,223	2,559,834	429	0.226	31,953	5,966.981	24.114	5.045	95.588	2.523
3	14,992,717	2,414,788	507	0.278	29,571	4,762.897	22.747	5.518	71.825	2.38
4	29,427,613	1,304,722	1,204	0.323	24,441	1,083.657	12.291	10.83	64.286	1.286
5	12,367,259	1,704,062	560	0.41	22,084	3,042.969	16.052	4.551	49.658	1.679
6	16,252,373	1,617,561	861	0.575	18,876	1,878.7	15.237	5.981	28.2	1.594

Lane 3 - 5uM Ionomycin

#	Vol. (Int.)	Local Bg. Corr. Vol.	Area	Rf	Density	Local Bg. Corr. Den.	% band purity	% lane purity	Mol. Wt.	Rel. Quant. (w/ LB Corr. Vol.)
1	10,543,241	511,767	480	0.158	21,965	1,066.183	5.528	3.893	146.429	0.504
2	11,640,454	1,859,149	407	0.231	28,600	4,567.935	20.081	4.299	92.647	1.832
3	15,573,688	3,133,339	518	0.288	30,065	6,048.918	33.844	5.751	70.238	3.088
4	17,542,677	877,561	720	0.33	24,364	1,218.835	9.479	6.478	63.095	0.865
5	14,469,448	1,883,776	629	0.417	23,003	2,994.876	20.347	5.343	48.632	1.856
6	10,396,745	992,691	546	0.583	19,041	1,818.116	10.722	3.839	27.4	0.978

Lane 4 - 2uM Thapsigargin

#	Vol. (Int.)	Local Bg. Corr. Vol.	Area	Rf	Density	Local Bg. Corr. Den.	% band purity	% lane purity	Mol. Wt.	Rel. Quant. (w/ LB Corr. Vol.)
1	10,480,207	1,116,355	546	0.165	19,194	2,044.607	6.499	3.775	141.071	1.1
2	12,941,140	3,305,089	444	0.233	29,146	7,443.896	19.24	4.662	91.176	3.257
3	16,562,323	4,187,950	532	0.29	31,132	7,872.087	24.379	5.966	69.841	4.127
4	21,755,874	2,155,170	819	0.337	26,563	2,631.465	12.546	7.837	61.905	2.124
5	13,661,756	4,223,132	432	0.427	31,624	9,775.769	24.584	4.921	47.263	4.162
6	12,228,433	2,190,743	546	0.59	22,396	4,012.351	12.753	4.405	26.6	2.159

Lane 5 - t = 0

#	Vol. (Int.)	Local Bg. Corr. Vol.	Area	Rf	Density	Local Bg. Corr. Den.	% band purity	% lane purity	Mol. Wt.	Rel. Quant. (w/ LB Corr. Vol.)
1	8,644,002	469,768	666	0.167	12,978	705.358	4.132	4.298	139.286	0.463
2	8,653,739	1,585,901	504	0.233	17,170	3,146.629	13.95	4.302	91.176	1.563
3	13,265,231	2,742,898	714	0.288	18,578	3,841.595	24.127	6.595	70.238	2.703
4	20,662,205	2,518,198	1,176	0.347	17,569	2,141.325	22.15	10.273	60.317	2.482
5	15,225,598	1,740,467	960	0.427	15,859	1,812.987	15.309	7.57	47.263	1.715
6	16,967,268	2,311,394	1,081	0.594	15,695	2,138.2	20.331	8.436	26.067	2.278

iBright™ Image Analysis Report

Katarina+ Chang

19 November 2022

pCofilin CHEMI_02232022_115805

Date:	23 February 2022 11:58:05AM
Mode:	Chemi Blots
Notes:	
Model:	FL1500
Instrument name:	2462619090234
Serial No:	2462619090234
Firmware version:	1.6.0
iBA version:	5.0
Image size:	676px X 540px
Image area:	118.63mm X 94.91mm
Optical Zoom:	1.9x
Digital Zoom:	1x
Focus level:	430
Resolution:	5 x 5
Exposure time:	13294 ms
Exposure mode:	Normal

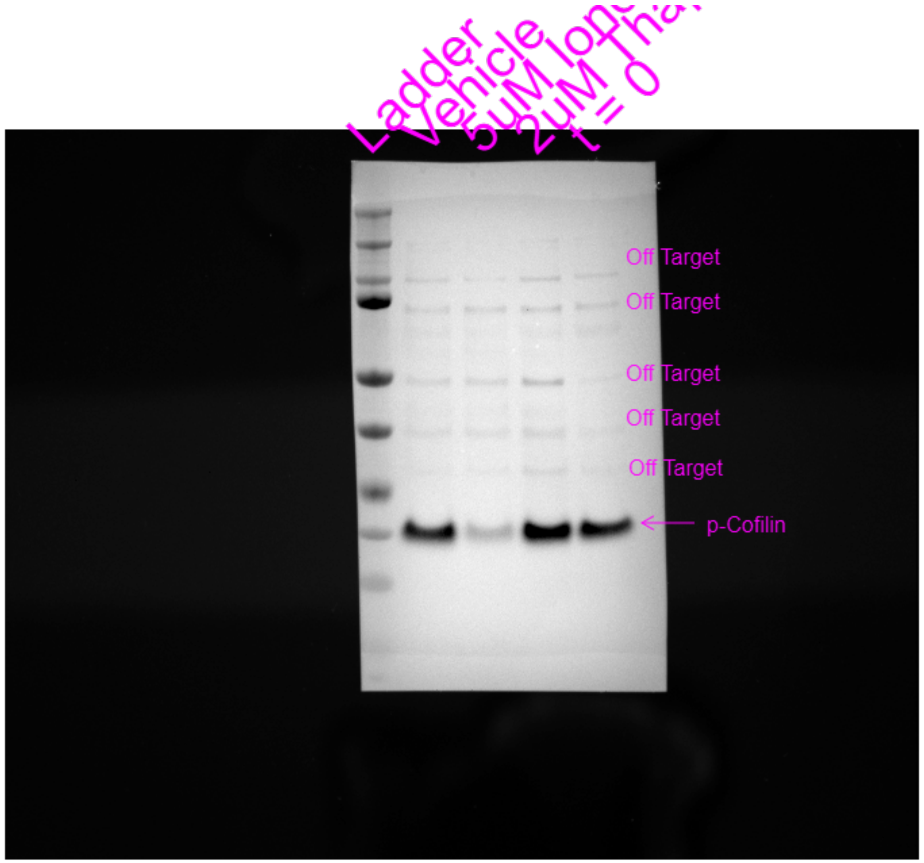

pCofilin CHEMI_02232022_115805

Date:	23 February 2022 11:58:05AM
Mode:	Chemi Blots
Notes:	
Model:	FL1500
Instrument name:	2462619090234
Serial No:	2462619090234
Firmware version:	1.6.0
iBA version:	5.0
Image size:	676px X 540px
Image area:	118.63mm X 94.91mm
Optical Zoom:	1.9x
Digital Zoom:	1x
Focus level:	430
Resolution:	5 x 5
Exposure time:	13294 ms
Exposure mode:	Normal

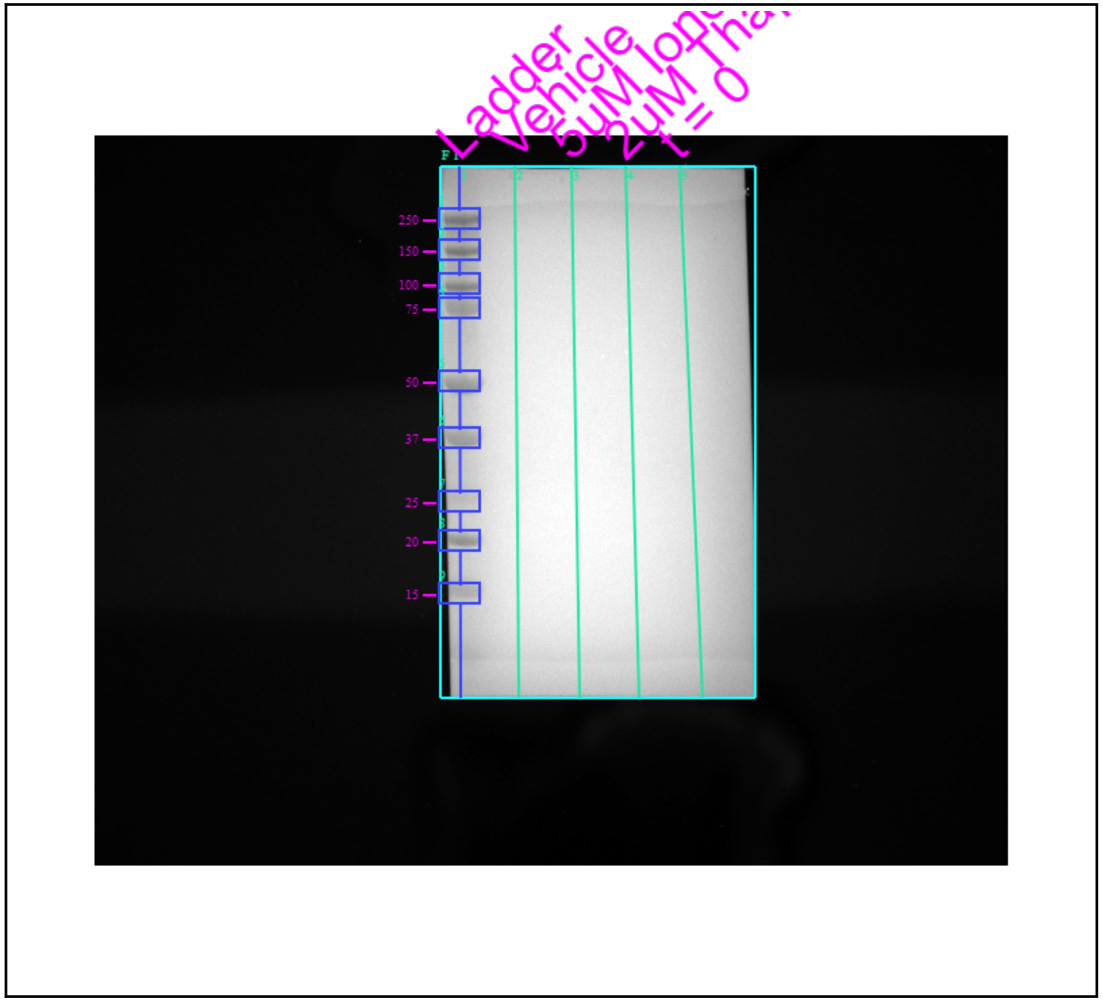

pCofilin CHEMI_02232022_115805

Date:	23 February 2022 11:58:05AM
Mode:	Chemi Blots
Notes:	
Model:	FL1500
Instrument name:	2462619090234
Serial No:	2462619090234
Firmware version:	1.6.0
iBA version:	5.0
Image size:	676px X 540px
Image area:	118.63mm X 94.91mm
Optical Zoom:	1.9x
Digital Zoom:	1x
Focus level:	430
Resolution:	5 x 5
Exposure time:	13294 ms
Exposure mode:	Normal

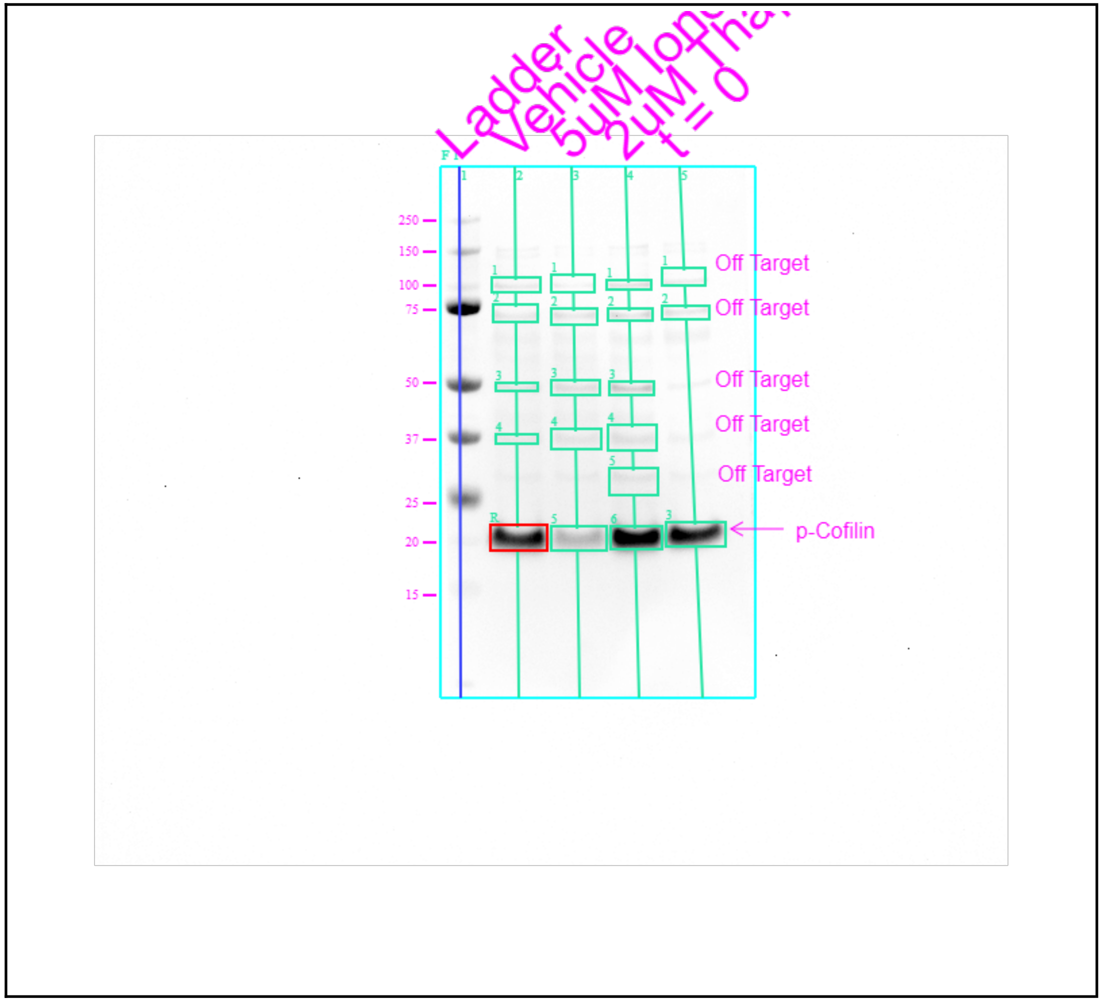

LANE AND BAND ANALYSIS DATA TABLE

pCofilin CHEMI_02232022_115805

Frame: 1
Channel: Membrane
Sensitivity: 100
Molecular Weight Analysis Regression Method : Point to Point

Lane 1 - Ladder

#	Vol. (Int.)	Local Bg. Corr. Vol.	Area	Rf	Density	Local Bg. Corr. Den.	% band purity	% lane purity	Mol. Wt.
1	18,618,572	467,284	496	0.097	37,537	942.106	4.505	4.435	250
2	17,837,792	361,807	496	0.155	35,963	729.45	3.488	4.249	150
3	17,242,615	388,278	496	0.219	34,763	782.819	3.743	4.108	100
4	16,809,106	310,389	496	0.265	33,889	625.786	2.992	4.004	75
5	17,240,926	798,393	496	0.402	34,759	1,609.664	7.697	4.107	50
6	17,661,126	1,363,544	496	0.509	35,607	2,749.082	13.145	4.207	37
7	17,183,438	1,989,437	496	0.628	34,644	4,010.962	19.178	4.094	25
8	18,731,246	2,255,520	496	0.702	37,764	4,547.421	21.743	4.462	20
9	18,742,608	2,438,660	496	0.802	37,787	4,916.654	23.509	4.465	15

Frame: 1
Channel: Chemi
Sensitivity: 100
Molecular Weight Analysis Regression Method : Point to Point

Lane 2 - Vehicle

#	Vol. (Int.)	Local Bg. Corr. Vol.	Area	Rf	Density	Local Bg. Corr. Den.	% band purity	% lane purity	Mol. Wt.	Rel. Quant. (w/ LB Corr. Vol.)
1	746,291	388,690	444	0.221	1,680.836	875.43	2.646	2.429	98.611	0.029
2	972,290	474,625	476	0.275	2,042.626	997.112	3.231	3.164	73.148	0.036
3	758,905	338,586	224	0.415	3,387.969	1,511.548	2.305	2.47	48.452	0.026
4	840,965	266,150	256	0.511	3,285.02	1,039.651	1.812	2.737	36.745	0.02
5	16,641,646	13,221,849	860	0.697	19,350	15,374	90.006	54.154	20.345	1

Lane 3 - 5uM Ionomycin

#	Vol. (Int.)	Local Bg. Corr. Vol.	Area	Rf	Density	Local Bg. Corr. Den.	% band purity	% lane purity	Mol. Wt.	Rel. Quant. (w/ LB Corr. Vol.)

#	Vol. (Int.)	Local Bg. Corr. Vol.	Area	Rf	Density	Local Bg. Corr. Den.	% band purity	% lane purity	Mol. Wt.	Rel. Quant. (w/ LB Corr. Vol.)
1	373,012	174,884	462	0.219	807.385	378.539	4.854	1.712	100	0.013
2	932,057	544,089	455	0.282	2,048.477	1,195.801	15.102	4.277	71.759	0.041
3	1,366,892	614,691	444	0.415	3,078.586	1,384.441	17.062	6.273	48.452	0.046
4	1,733,159	500,802	608	0.511	2,850.59	823.688	13.901	7.953	36.745	0.038
5	5,642,041	1,768,291	798	0.7	7,070.227	2,215.904	49.082	25.891	20.172	0.134

Lane 4 - 2uM Thapsigargin

#	Vol. (Int.)	Local Bg. Corr. Vol.	Area	Rf	Density	Local Bg. Corr. Den.	% band purity	% lane purity	Mol. Wt.	Rel. Quant. (w/ LB Corr. Vol.)
1	577,359	441,313	272	0.221	2,122.643	1,622.475	2.402	1.644	98.611	0.033
2	778,985	595,425	340	0.277	2,291.132	1,751.251	3.24	2.217	72.685	0.045
3	1,468,490	1,043,690	374	0.417	3,926.444	2,790.617	5.68	4.18	48.143	0.079
4	2,071,912	771,842	740	0.509	2,799.881	1,043.031	4.201	5.898	37	0.058
5	2,394,669	658,155	777	0.593	3,081.942	847.047	3.582	6.817	28.574	0.05
6	19,304,246	14,864,231	702	0.697	27,498	21,174	80.895	54.952	20.345	1.124

Lane 5 - t = 0

#	Vol. (Int.)	Local Bg. Corr. Vol.	Area	Rf	Density	Local Bg. Corr. Den.	% band purity	% lane purity	Mol. Wt.	Rel. Quant. (w/ LB Corr. Vol.)
1	514,160	334,669	462	0.206	1,112.9	724.392	2.409	2.318	110	0.025
2	659,448	477,791	396	0.275	1,665.273	1,206.544	3.439	2.973	73.148	0.036
3	16,273,815	13,079,271	855	0.692	19,033	15,297	94.151	73.358	20.69	0.989

iBright™ Image Analysis Report

Katarina+ Chang

19 November 2022

Alpha Tubulin CHEMI_02242022_125609

Date:	24 February 2022 12:56:09PM
Mode:	Chemi Blots
Notes:	
Model:	FL1500
Instrument name:	2462619090234
Serial No:	2462619090234
Firmware version:	1.6.0
iBA version:	5.0
Image size:	615px X 491px
Image area:	112.7mm X 90.16mm
Optical Zoom:	2x
Digital Zoom:	1.1x
Focus level:	455
Resolution:	5 x 5
Exposure time:	1935 ms
Exposure mode:	Normal

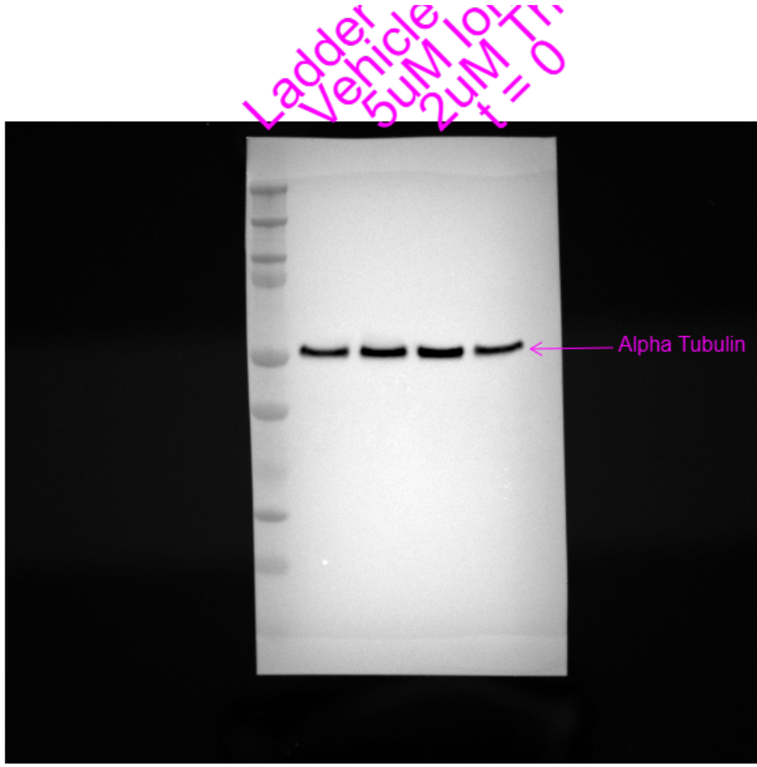

Alpha Tubulin CHEMI_02242022_125609

Date:	24 February 2022 12:56:09PM
Mode:	Chemi Blots
Notes:	
Model:	FL1500
Instrument name:	2462619090234
Serial No:	2462619090234
Firmware version:	1.6.0
iBA version:	5.0
Image size:	615px X 491px
Image area:	112.7mm X 90.16mm
Optical Zoom:	2x
Digital Zoom:	1.1x
Focus level:	455
Resolution:	5 x 5
Exposure time:	1935 ms
Exposure mode:	Normal

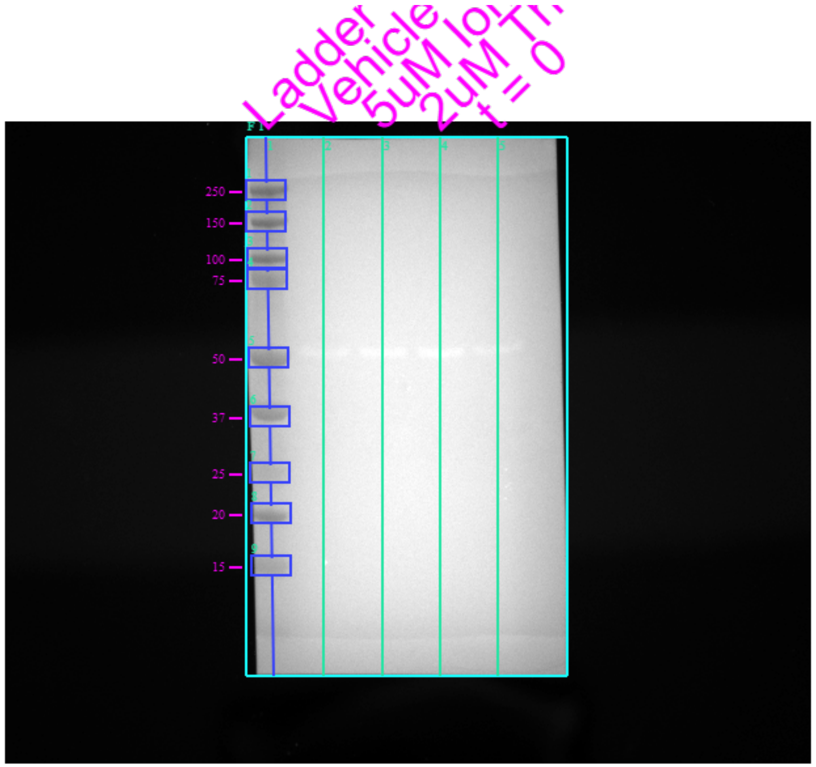

Alpha Tubulin CHEMI_02242022_125609

Date:	24 February 2022 12:56:09PM
Mode:	Chemi Blots
Notes:	
Model:	FL1500
Instrument name:	2462619090234
Serial No:	2462619090234
Firmware version:	1.6.0
iBA version:	5.0
Image size:	615px X 491px
Image area:	112.7mm X 90.16mm
Optical Zoom:	2x
Digital Zoom:	1.1x
Focus level:	455
Resolution:	5 x 5
Exposure time:	1935 ms
Exposure mode:	Normal

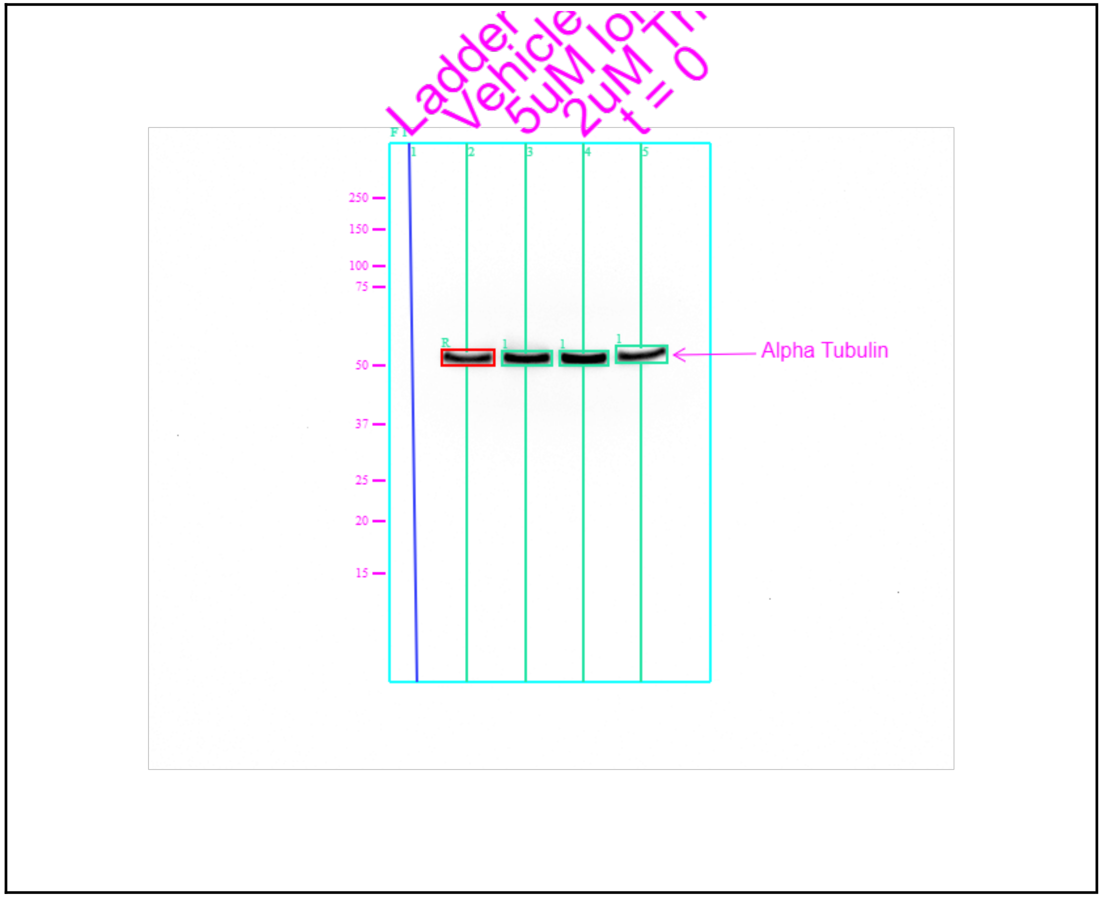

LANE AND BAND ANALYSIS DATA TABLE

Alpha Tubulin CHEMI_02242022_125609

Frame: 1
Channel: Membrane
Sensitivity: 100
Molecular Weight Analysis Regression Method : Point to Point

Lane 1 - Ladder

#	Vol. (Int.)	Local Bg. Corr. Vol.	Area	Rf	Density	Local Bg. Corr. Den.	% band purity	% lane purity	Mol. Wt.
1	18,817,163	340,008	496	0.097	37,937	685.5	8.394	3.678	250
2	18,197,676	350,913	496	0.155	36,688	707.488	8.664	3.557	150
3	17,526,749	481,735	496	0.223	35,336	971.24	11.893	3.426	100
4	17,046,902	112,906	496	0.262	34,368	227.635	2.788	3.332	75
5	16,974,017	586,404	496	0.408	34,221	1,182.267	14.478	3.318	50
6	16,182,073	111,978	496	0.517	32,625	225.763	2.765	3.163	37
7	15,734,739	541,768	496	0.621	31,723	1,092.275	13.376	3.076	25
8	16,662,109	643,297	496	0.697	33,592	1,296.971	15.882	3.257	20
9	17,089,286	881,396	496	0.794	34,454	1,777.009	21.761	3.34	15

Frame: 1
Channel: Chemi
Sensitivity: 100
Molecular Weight Analysis Regression Method : Point to Point

Lane 2 - Vehicle

#	Vol. (Int.)	Local Bg. Corr. Vol.	Area	Rf	Density	Local Bg. Corr. Den.	% band purity	% lane purity	Mol. Wt.	Rel. Quant. (w/ LB Corr. Vol.)
1	9,370,673	8,450,400	533	0.398	17,581	15,854	100	77.876	51.667	1

Lane 3 - 5uM Ionomycin

#	Vol. (Int.)	Local Bg. Corr. Vol.	Area	Rf	Density	Local Bg. Corr. Den.	% band purity	% lane purity	Mol. Wt.	Rel. Quant. (w/ LB Corr. Vol.)
1	11,456,694	9,993,602	468	0.398	24,480	21,353	100	71.624	51.667	1.183

Lane 4 - 2uM Thapsigargin

#	Vol. (Int.)	Local Bg. Corr. Vol.	Area	Rf	Density	Local Bg. Corr. Den.	% band purity	% lane purity	Mol. Wt.	Rel. Quant. (w/ LB Corr. Vol.)

#	Vol. (Int.)	Local Bg. Corr. Vol.	Area	Rf	Density	Local Bg. Corr. Den.	% band purity	% lane purity	Mol. Wt.	Rel. Quant. (w/ LB Corr. Vol.)
1	12,972,711	11,958,026	456	0.398	28,448	26,223	100	77.58	51.667	1.415

Lane 5 - t = 0

#	Vol. (Int.)	Local Bg. Corr. Vol.	Area	Rf	Density	Local Bg. Corr. Den.	% band purity	% lane purity	Mol. Wt.	Rel. Quant. (w/ LB Corr. Vol.)
1	8,651,264	8,085,318	560	0.391	15,448	14,438	100	79.175	52.917	0.957

iBright™ Image Analysis Report

Katarina+ Chang

19 November 2022

GAPDH_CHEMI_02242022_154803

Date:	24 February 2022 03:48:03PM
Mode:	Chemi Blots
Notes:	
Model:	FL1500
Instrument name:	2462619090234
Serial No:	2462619090234
Firmware version:	1.6.0
iBA version:	5.1.0
Image size:	676px X 540px
Image area:	118.63mm X 94.91mm
Optical Zoom:	1.9x
Digital Zoom:	1x
Focus level:	430
Resolution:	5 x 5
Exposure time:	28401 ms
Exposure mode:	Normal

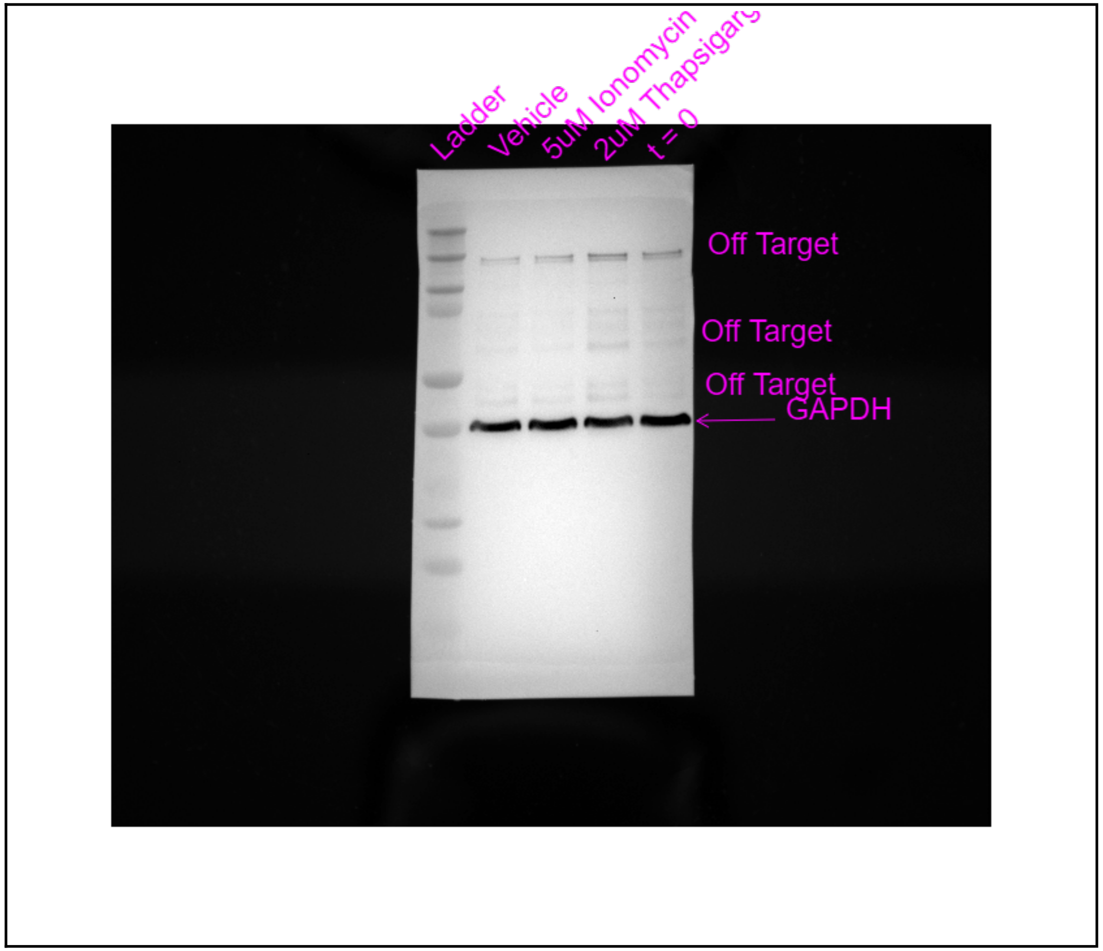

GAPDH_CHEMI_02242022_154803

Date:	24 February 2022 03:48:03PM
Mode:	Chemi Blots
Notes:	
Model:	FL1500
Instrument name:	2462619090234
Serial No:	2462619090234
Firmware version:	1.6.0
iBA version:	5.1.0
Image size:	676px X 540px
Image area:	118.63mm X 94.91mm
Optical Zoom:	1.9x
Digital Zoom:	1x
Focus level:	430
Resolution:	5 x 5
Exposure time:	28401 ms
Exposure mode:	Normal

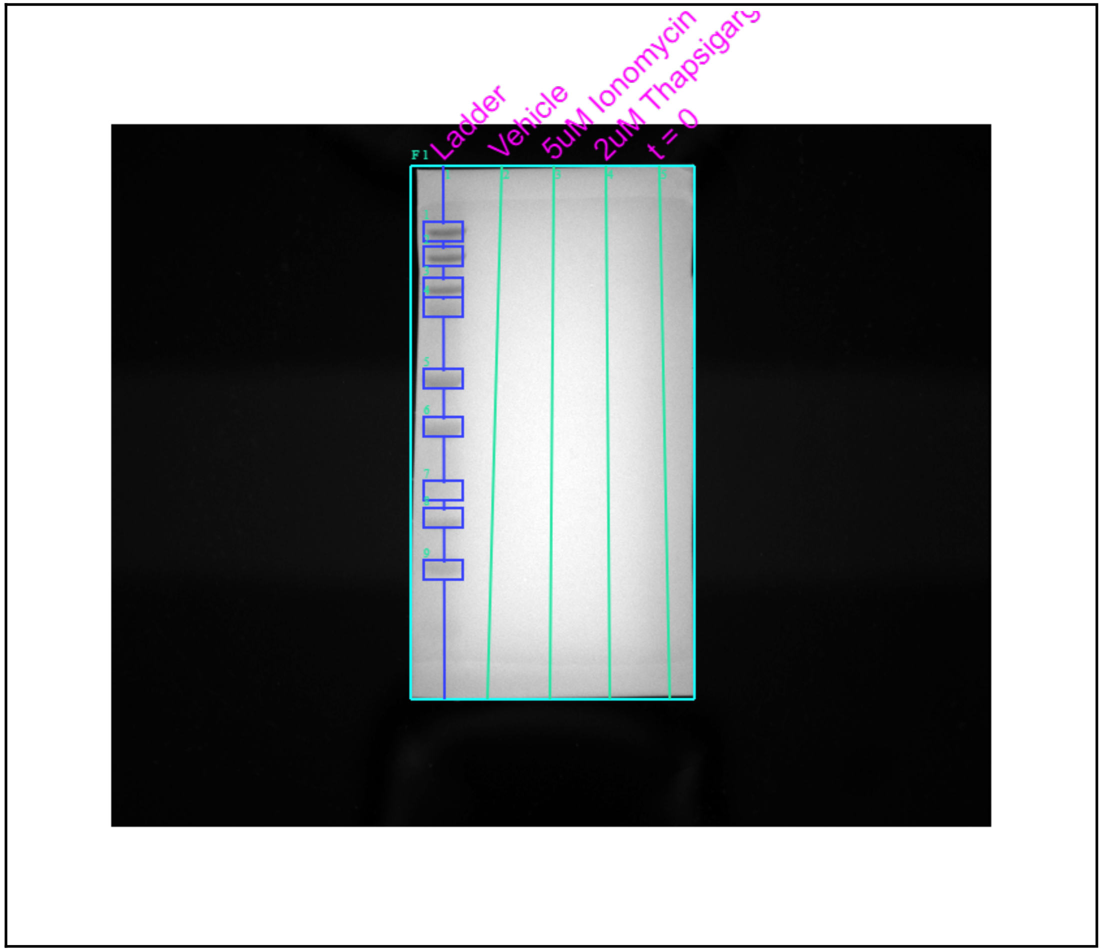

GAPDH_CHEMI_02242022_154803

Date:	24 February 2022 03:48:03PM
Mode:	Chemi Blots
Notes:	
Model:	FL1500
Instrument name:	2462619090234
Serial No:	2462619090234
Firmware version:	1.6.0
iBA version:	5.1.0
Image size:	676px X 540px
Image area:	118.63mm X 94.91mm
Optical Zoom:	1.9x
Digital Zoom:	1x
Focus level:	430
Resolution:	5 x 5
Exposure time:	28401 ms
Exposure mode:	Normal

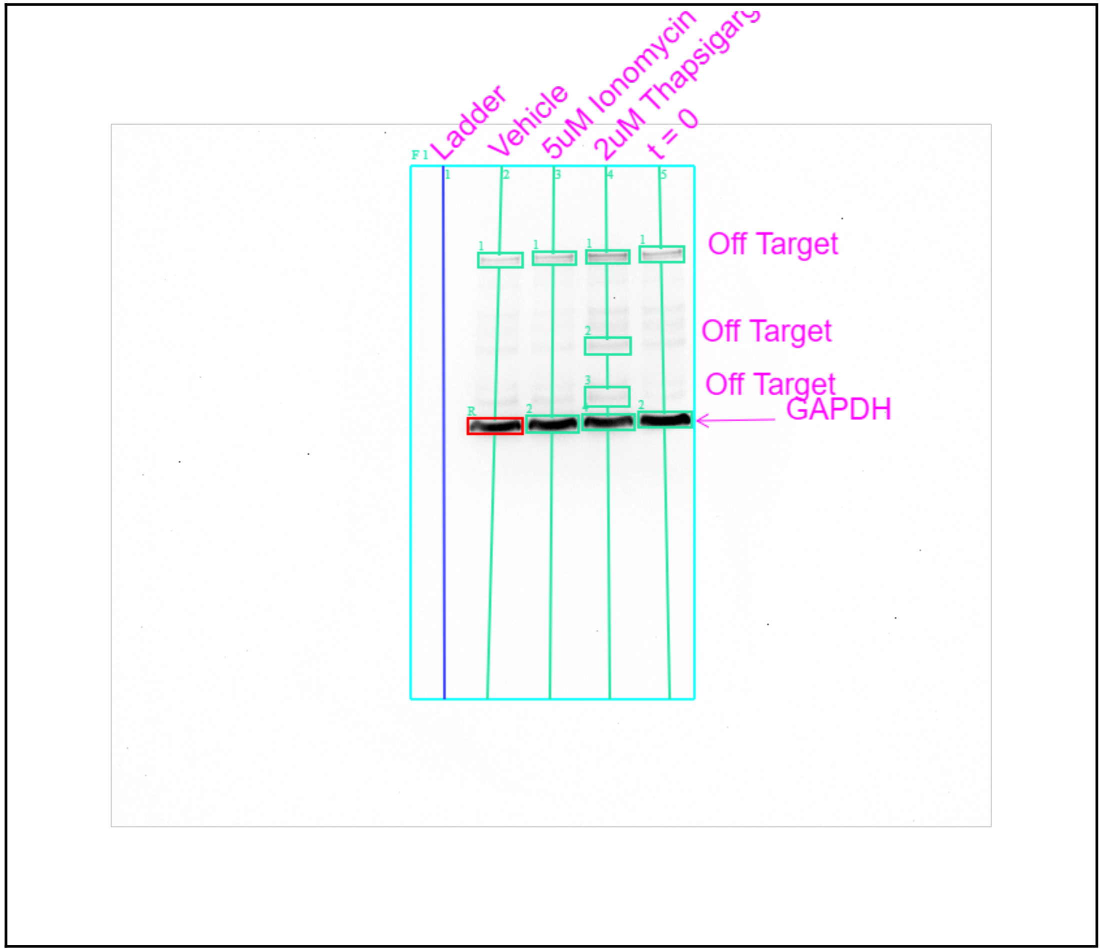

LANE AND BAND ANALYSIS DATA TABLE

GAPDH_CHEMI_02242022_154803

Frame: 1
Channel: Membrane
Sensitivity: 100
Molecular Weight Analysis Regression Method : Point to Point

Lane 1 - Ladder

#	Vol. (Int.)	Local Bg. Corr. Vol.	Area	Rf	Density	Local Bg. Corr. Den.	% band purity	% lane purity	Mol. Wt.
1	18,005,776	1,742,288	496	0.122	36,301	3,512.678	13.767	3.912	250
2	17,555,173	1,325,956	496	0.168	35,393	2,673.299	10.477	3.814	150
3	16,630,089	1,113,658	496	0.227	33,528	2,245.279	8.8	3.613	100
4	15,726,371	492,735	496	0.263	31,706	993.419	3.893	3.417	75
5	15,648,485	2,512,375	496	0.398	31,549	5,065.274	19.852	3.4	50
6	14,763,080	2,041,908	496	0.488	29,764	4,116.75	16.134	3.207	37
7	13,147,289	698,092	496	0.607	26,506	1,407.444	5.516	2.856	25
8	13,910,698	1,330,685	496	0.659	28,045	2,682.834	10.514	3.022	20
9	14,250,458	1,398,063	496	0.756	28,730	2,818.677	11.047	3.096	15

Frame: 1
Channel: Chemi
Sensitivity: 100
Molecular Weight Analysis Regression Method : Point to Point

Lane 2 - Vehicle

#	Vol. (Int.)	Local Bg. Corr. Vol.	Area	Rf	Density	Local Bg. Corr. Den.	% band purity	% lane purity	Mol. Wt.	Rel. Quant. (w/ LB Corr. Vol.)
1	830,448	544,453	420	0.176	1,977.257	1,296.319	7.092	4.376	143.75	0.076
2	8,740,551	7,132,408	559	0.488	15,636	12,759	92.908	46.057	37	1

Lane 3 - 5uM Ionomycin

#	Vol. (Int.)	Local Bg. Corr. Vol.	Area	Rf	Density	Local Bg. Corr. Den.	% band purity	% lane purity	Mol. Wt.	Rel. Quant. (w/ LB Corr. Vol.)
1	1,164,853	805,511	374	0.173	3,114.58	2,153.775	9.643	5.299	145.833	0.113
2	9,733,783	7,547,728	574	0.483	16,957	13,149	90.357	44.282	37.703	1.058

Lane 4 - 2uM Thapsigargin

#	Vol. (Int.)	Local Bg. Corr. Vol.	Area	Rf	Density	Local Bg. Corr. Den.	% band purity	% lane purity	Mol. Wt.	Rel. Quant. (w/ LB Corr. Vol.)
1	1,849,564	1,389,013	374	0.171	4,945.358	3,713.941	15.728	7.6	147.917	0.195
2	1,245,758	536,435	504	0.337	2,471.742	1,064.356	6.074	5.119	61.364	0.075
3	1,536,699	231,032	560	0.432	2,744.105	412.557	2.616	6.315	45.081	0.032
4	8,699,884	6,674,996	533	0.48	16,322	12,523	75.582	35.75	38.054	0.936

Lane 5 - t = 0

#	Vol. (Int.)	Local Bg. Corr. Vol.	Area	Rf	Density	Local Bg. Corr. Den.	% band purity	% lane purity	Mol. Wt.	Rel. Quant. (w/ LB Corr. Vol.)
1	1,415,902	1,070,079	455	0.166	3,111.873	2,351.822	9.837	6.31	155.263	0.15
2	10,857,929	9,807,799	546	0.476	19,886	17,963	90.163	48.388	38.757	1.375

iBright™ Image Analysis Report

Katarina+ Chang

19 November 2022

total MYPT1 CHEMI_02222022_114433

Date:	22 February 2022 11:44:33AM
Mode:	Chemi Blots
Notes:	
Model:	FL1500
Instrument name:	2462619090234
Serial No:	2462619090234
Firmware version:	1.6.0
iBA version:	5.0
Image size:	615px X 491px
Image area:	112.7mm X 90.16mm
Optical Zoom:	2x
Digital Zoom:	1.1x
Focus level:	455
Resolution:	5 x 5
Exposure time:	3343 ms
Exposure mode:	Normal

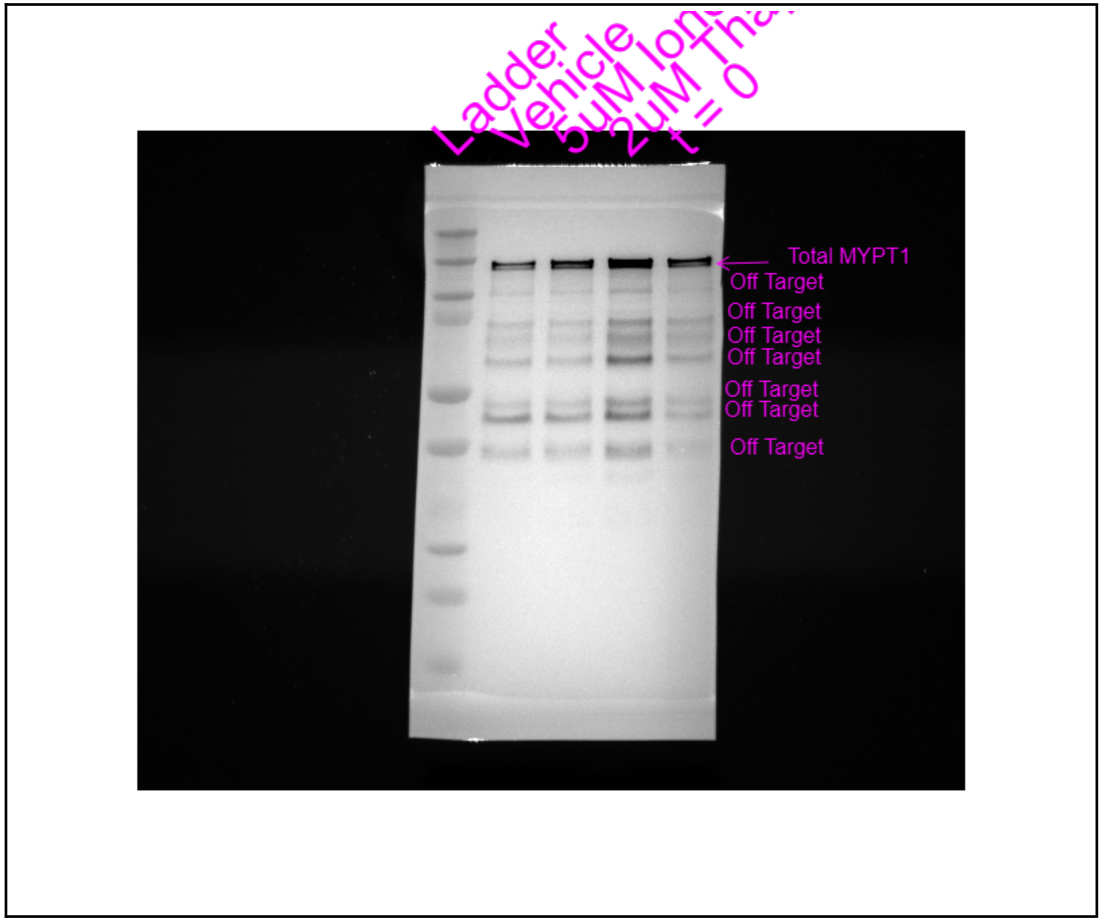

total MYPT1 CHEMI_02222022_114433

Date:	22 February 2022 11:44:33AM
Mode:	Chemi Blots
Notes:	
Model:	FL1500
Instrument name:	2462619090234
Serial No:	2462619090234
Firmware version:	1.6.0
iBA version:	5.0
Image size:	615px X 491px
Image area:	112.7mm X 90.16mm
Optical Zoom:	2x
Digital Zoom:	1.1x
Focus level:	455
Resolution:	5 x 5
Exposure time:	3343 ms
Exposure mode:	Normal

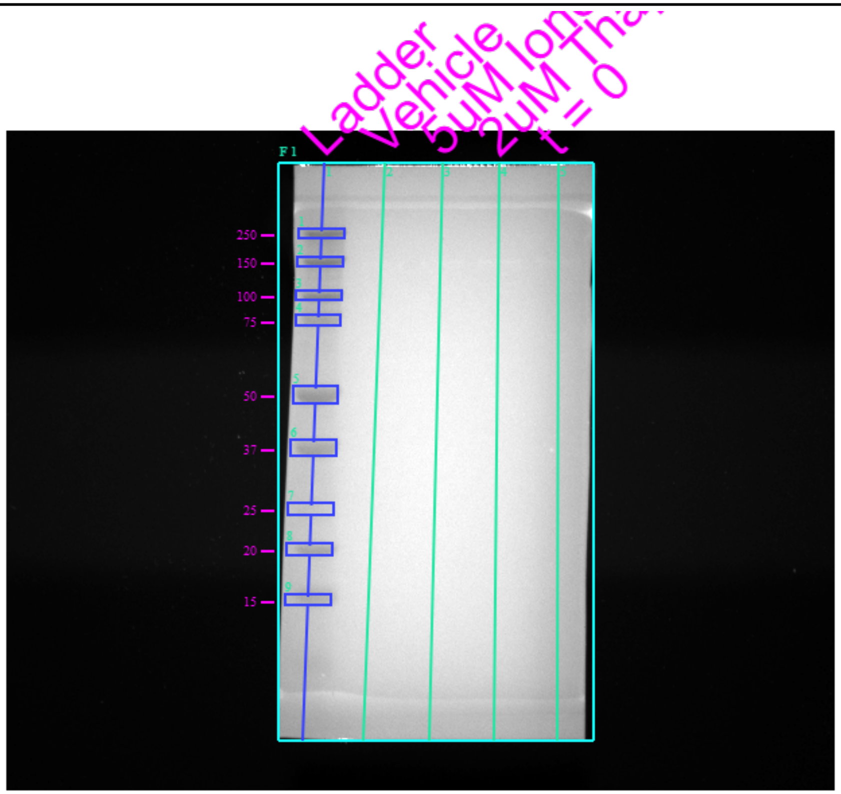

total MYPT1 CHEMI_02222022_114433

Date:	22 February 2022 11:44:33AM
Mode:	Chemi Blots
Notes:	
Model:	FL1500
Instrument name:	2462619090234
Serial No:	2462619090234
Firmware version:	1.6.0
iBA version:	5.0
Image size:	615px X 491px
Image area:	112.7mm X 90.16mm
Optical Zoom:	2x
Digital Zoom:	1.1x
Focus level:	455
Resolution:	5 x 5
Exposure time:	3343 ms
Exposure mode:	Normal

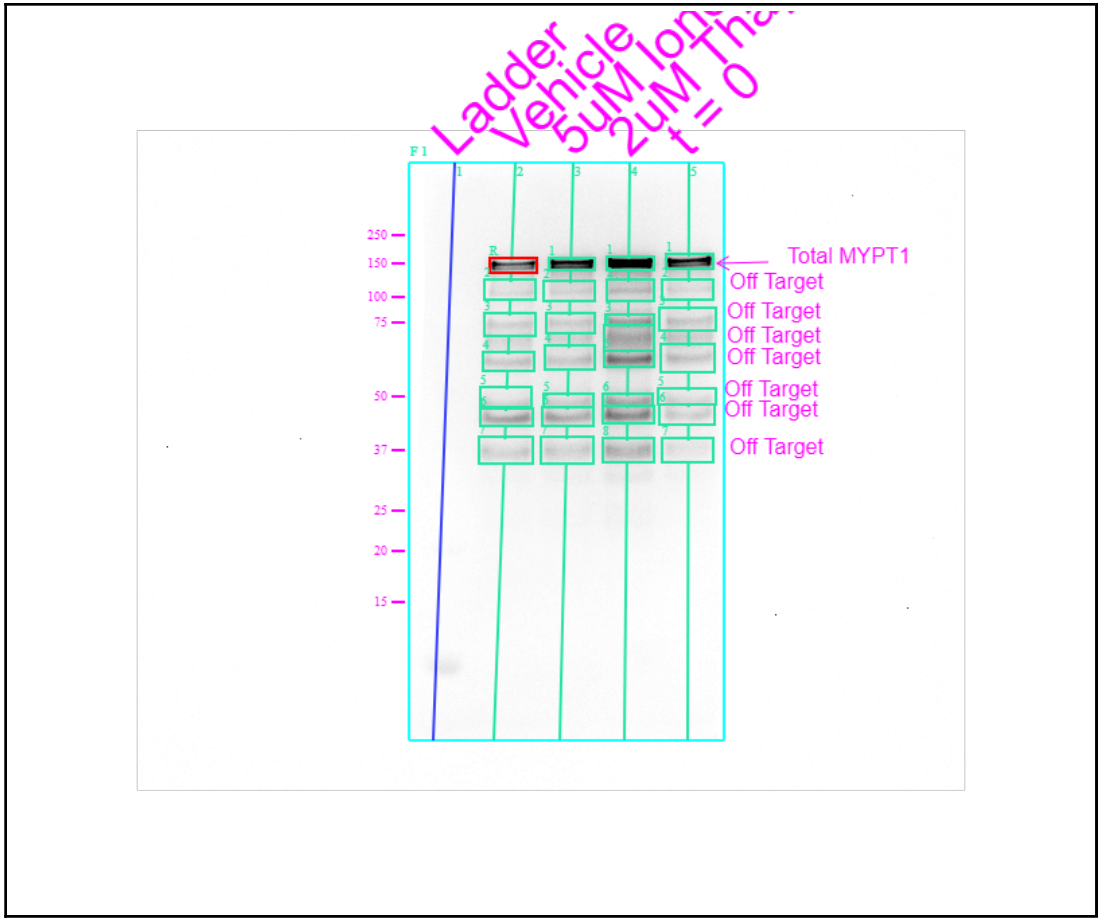

LANE AND BAND ANALYSIS DATA TABLE

total MYPT1 CHEMI_02222022_114433

Frame: 1
Channel: Membrane
Sensitivity: 100
Molecular Weight Analysis Regression Method : Point to Point

Lane 1 - Ladder

#	Vol. (Int.)	Local Bg. Corr. Vol.	Area	Rf	Density	Local Bg. Corr. Den.	% band purity	% lane purity	Mol. Wt.
1	10,387,813	1,161,593	280	0.121	37,099	4,148.549	21.61	2.353	250
2	10,134,932	845,175	280	0.17	36,196	3,018.485	15.724	2.296	150
3	9,730,635	601,123	280	0.228	34,752	2,146.868	11.183	2.204	100
4	9,673,496	193,414	306	0.272	31,612	632.074	3.598	2.191	75
5	15,333,120	1,260,286	476	0.4	32,212	2,647.66	23.446	3.474	50
6	14,081,275	759,886	455	0.493	30,947	1,670.08	14.137	3.19	37
7	9,585,477	1,895.773	350	0.598	27,387	5.416	0.035	2.172	25
8	10,712,041	452,217	350	0.667	30,605	1,292.051	8.413	2.427	20
9	9,656,863	99,577	315	0.756	30,656	316.118	1.853	2.188	15

Frame: 1
Channel: Chemi
Sensitivity: 100
Molecular Weight Analysis Regression Method : Point to Point

Lane 2 - Vehicle

#	Vol. (Int.)	Local Bg. Corr. Vol.	Area	Rf	Density	Local Bg. Corr. Den.	% band purity	% lane purity	Mol. Wt.	Rel. Quant. (w/ LB Corr. Vol.)
1	3,939,525	2,836,772	432	0.177	9,119.271	6,566.602	36.275	15.362	144	1
2	1,723,637	339,924	624	0.219	2,762.239	544.752	4.347	6.721	108	0.12
3	2,245,923	654,105	702	0.279	3,199.321	931.774	8.364	8.758	73.636	0.231
4	2,015,807	833,081	585	0.344	3,445.824	1,424.071	10.653	7.861	60.909	0.294
5	1,811,928	440,303	624	0.405	2,903.731	705.615	5.63	7.066	49.35	0.155
6	2,902,584	1,506,883	560	0.44	5,183.186	2,690.864	19.269	11.319	44.475	0.531
7	2,423,321	1,209,140	861	0.498	2,814.542	1,404.345	15.462	9.45	36.467	0.426

Lane 3 - 5uM Ionomycin

#	Vol. (Int.)	Local Bg. Corr. Vol.	Area	Rf	Density	Local Bg. Corr. Den.	% band purity	% lane purity	Mol. Wt.	Rel. Quant. (w/ LB Corr. Vol.)
1	5,331,851	3,972,709	350	0.174	15,233	11,350	47.084	17.243	146	1.4
2	2,163,518	398,982	624	0.221	3,467.176	639.395	4.729	6.997	106	0.141
3	2,224,771	504,006	592	0.277	3,758.059	851.363	5.973	7.195	74.091	0.178
4	2,678,575	780,399	722	0.337	3,709.938	1,080.886	9.249	8.662	62.273	0.275
5	1,637,418	379,986	418	0.412	3,917.268	909.06	4.504	5.295	48.375	0.134
6	2,897,379	1,272,212	585	0.44	4,952.785	2,174.722	15.078	9.37	44.475	0.448
7	2,547,271	1,129,146	840	0.498	3,032.465	1,344.222	13.383	8.238	36.467	0.398

Lane 4 - 2uM Thapsigargin

#	Vol. (Int.)	Local Bg. Corr. Vol.	Area	Rf	Density	Local Bg. Corr. Den.	% band purity	% lane purity	Mol. Wt.	Rel. Quant. (w/ LB Corr. Vol.)
1	8,318,990	6,464,994	396	0.174	21,007	16,325	40.21	18.894	146	2.279
2	2,961,653	682,290	612	0.221	4,839.302	1,114.853	4.244	6.726	106	0.241
3	2,277,256	863,444	333	0.272	6,838.607	2,592.927	5.37	5.172	75	0.304
4	5,251,539	1,115,694	760	0.302	6,909.92	1,468.019	6.939	11.927	69.091	0.393
5	4,114,733	2,188,637	494	0.34	8,329.419	4,430.44	13.613	9.345	61.818	0.772
6	2,416,554	827,407	380	0.409	6,359.353	2,177.387	5.146	5.488	48.7	0.292
7	4,033,871	1,943,085	546	0.435	7,388.042	3,558.765	12.085	9.162	45.125	0.685
8	3,431,301	1,992,563	741	0.498	4,630.636	2,689.02	12.393	7.793	36.467	0.702

Lane 5 - t = 0

#	Vol. (Int.)	Local Bg. Corr. Vol.	Area	Rf	Density	Local Bg. Corr. Den.	% band purity	% lane purity	Mol. Wt.	Rel. Quant. (w/ LB Corr. Vol.)
1	5,489,244	4,246,190	432	0.17	12,706	9,829.144	52.733	21.766	150	1.497
2	1,838,505	330,493	624	0.219	2,946.322	529.638	4.104	7.29	108	0.117
3	2,698,250	712,813	774	0.27	3,486.111	920.948	8.852	10.699	76.316	0.251
4	3,002,429	1,014,303	902	0.337	3,328.635	1,124.505	12.596	11.905	62.273	0.358
5	1,577,339	446,528	572	0.405	2,757.586	780.645	5.545	6.254	49.35	0.157
6	1,992,902	675,242	672	0.435	2,965.628	1,004.825	8.386	7.902	45.125	0.238
7	1,503,092	626,696	741	0.498	2,028.464	845.744	7.783	5.96	36.467	0.221

iBright™ Image Analysis Report

Katarina+ Chang

19 November 2022

GAPDH CHEMI_02242022_154136

Date:	24 February 2022 03:41:36PM
Mode:	Chemi Blots
Notes:	
Model:	FL1500
Instrument name:	2462619090234
Serial No:	2462619090234
Firmware version:	1.6.0
iBA version:	5.1.0
Image size:	676px X 540px
Image area:	118.63mm X 94.91mm
Optical Zoom:	1.9x
Digital Zoom:	1x
Focus level:	430
Resolution:	5 x 5
Exposure time:	21164 ms
Exposure mode:	Normal

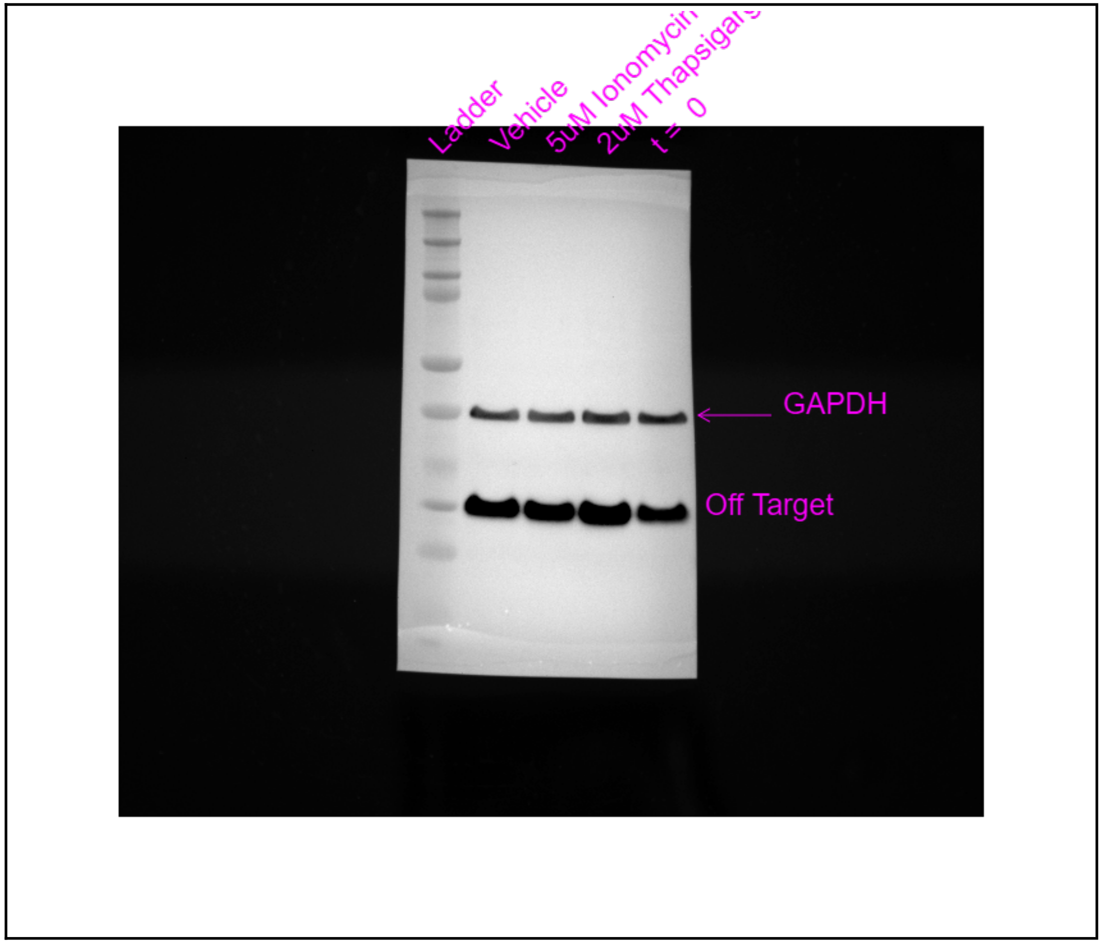

GAPDH CHEMI_02242022_154136

Date:	24 February 2022 03:41:36PM
Mode:	Chemi Blots
Notes:	
Model:	FL1500
Instrument name:	2462619090234
Serial No:	2462619090234
Firmware version:	1.6.0
iBA version:	5.1.0
Image size:	676px X 540px
Image area:	118.63mm X 94.91mm
Optical Zoom:	1.9x
Digital Zoom:	1x
Focus level:	430
Resolution:	5 x 5
Exposure time:	21164 ms
Exposure mode:	Normal

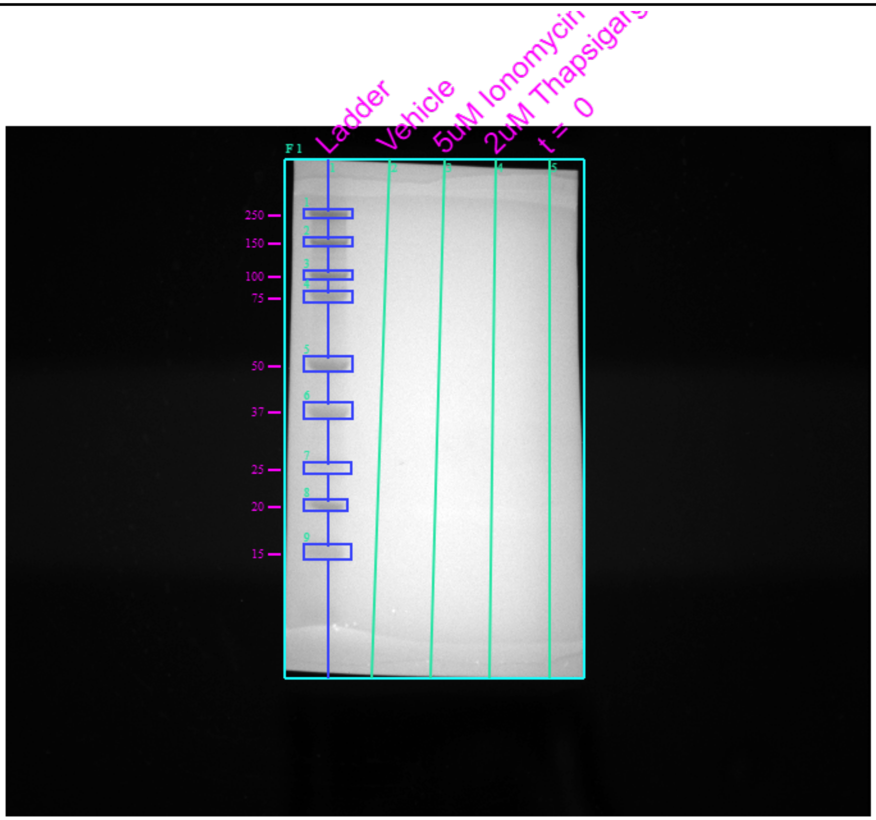

GAPDH CHEMI_02242022_154136

Date:	24 February 2022 03:41:36PM
Mode:	Chemi Blots
Notes:	
Model:	FL1500
Instrument name:	2462619090234
Serial No:	2462619090234
Firmware version:	1.6.0
iBA version:	5.1.0
Image size:	676px X 540px
Image area:	118.63mm X 94.91mm
Optical Zoom:	1.9x
Digital Zoom:	1x
Focus level:	430
Resolution:	5 x 5
Exposure time:	21164 ms
Exposure mode:	Normal

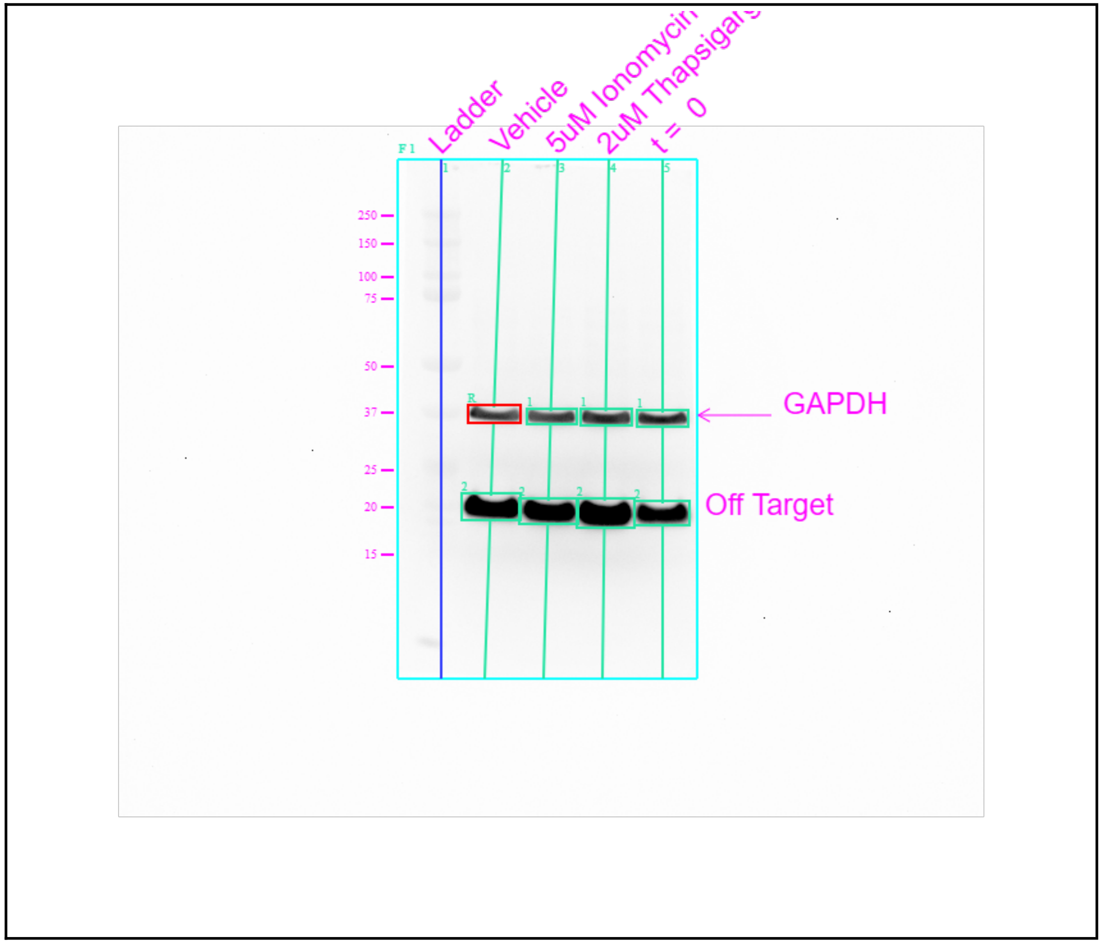

LANE AND BAND ANALYSIS DATA TABLE

GAPDH CHEMI_02242022_154136

Frame: 1
Channel: Membrane
Sensitivity: 100
Molecular Weight Analysis Regression Method : Point to Point

Lane 1 - Ladder

#	Vol. (Int.)	Local Bg. Corr. Vol.	Area	Rf	Density	Local Bg. Corr. Den.	% band purity	% lane purity	Rolling Bg. Corr. Vol.	Rolling Bg. Corr. Den.	Mol. Wt.
1	11,220,453	1,704,260	312	0.103	35,962	5,462.375	12.061	3.84	1,963,264	6,292.513	250
2	10,740,357	1,746,843	312	0.158	34,424	5,598.857	10.13	3.225	1,648,896	5,284.923	150
3	10,305,998	1,741,574	312	0.222	33,032	5,581.97	9.848	3.135	1,603,072	5,138.051	100
4	11,860,756	1,497,727	390	0.264	30,412	3,840.327	8.787	2.797	1,430,272	3,667.364	75
5	15,190,406	2,795,947	507	0.394	29,961	5,514.689	17.127	5.453	2,787,840	5,498.698	50
6	15,289,503	2,453,726	546	0.483	28,002	4,494.005	14.999	4.775	2,441,472	4,471.56	37
7	9,577,484	595,687	380	0.594	25,203	1,567.598	4.382	1.395	713,216	1,876.884	25
8	10,234,435	1,788,047	350	0.665	29,241	5,108.708	12.069	3.842	1,964,544	5,612.983	20
9	13,377,619	1,515,725	494	0.756	27,080	3,068.27	10.598	3.374	1,725,184	3,492.275	15

Frame: 1
Channel: Chemi
Sensitivity: 100
Molecular Weight Analysis Regression Method : Point to Point

Lane 2 - Vehicle

#	Vol. (Int.)	Local Bg. Corr. Vol.	Area	Rf	Density	Local Bg. Corr. Den.
1	16,467,880	13,685,575	630	0.49	26,139	21,723
2	44,916,102	35,232,724	1,034	0.667	43,439	34,074

#	% band purity	% lane purity	Rolling Bg. Corr. Vol.	Rolling Bg. Corr. Den.	Mol. Wt.	Rel. Quant. (w/ LB Corr. Vol.)
1	26.585	26.544	14,546,432	23,089	36.2	1
2	73.415	73.3	40,169,728	38,848	19.865	2.574

Lane 3 - 5uM Ionomycin

#	Vol. (Int.)	Local Bg. Corr. Vol.	Area	Rf	Density	Local Bg. Corr. Den.

#	Vol. (Int.)	Local Bg. Corr. Vol.	Area	Rf	Density	Local Bg. Corr. Den.
1	15,678,471	12,096,629	520	0.495	30,150	23,262
2	45,306,965	26,773,290	987	0.677	45,903	27,125

#	% band purity	% lane purity	Rolling Bg. Corr. Vol.	Rolling Bg. Corr. Den.	Mol. Wt.	Rel. Quant. (w/ LB Corr. Vol.)
1	26.239	25.479	13,948,416	26,823	35.667	0.884
2	73.761	71.622	39,209,984	39,726	19.324	1.956

Lane 4 - 2uM Thapsigargin

#	Vol. (Int.)	Local Bg. Corr. Vol.	Area	Rf	Density	Local Bg. Corr. Den.
1	18,830,943	15,018,772	574	0.495	32,806	26,165
2	51,389,599	34,822,892	1,104	0.68	46,548	31,542

#	% band purity	% lane purity	Rolling Bg. Corr. Vol.	Rolling Bg. Corr. Den.	Mol. Wt.	Rel. Quant. (w/ LB Corr. Vol.)
1	26.885	25.895	16,626,688	28,966	35.667	1.097
2	73.115	70.422	45,216,512	40,956	19.189	2.544

Lane 5 - t = 0

#	Vol. (Int.)	Local Bg. Corr. Vol.	Area	Rf	Density	Local Bg. Corr. Den.
1	17,698,628	15,305,986	574	0.498	30,833	26,665
2	35,907,385	28,363,904	880	0.68	40,803	32,231

#	% band purity	% lane purity	Rolling Bg. Corr. Vol.	Rolling Bg. Corr. Den.	Mol. Wt.	Rel. Quant. (w/ LB Corr. Vol.)
1	33.031	31.492	15,933,696	27,759	35.4	1.118
2	66.969	63.85	32,304,896	36,710	19.189	2.073

iBright™ Image Analysis Report

Katarina+ Chang

19 November 2022

total Cofilin CHEMI_02242022_125217

Date:	24 February 2022 12:52:17PM
Mode:	Chemi Blots
Notes:	
Model:	FL1500
Instrument name:	2462619090234
Serial No:	2462619090234
Firmware version:	1.6.0
iBA version:	5.1.0
Image size:	676px X 540px
Image area:	125.22mm X 100.18mm
Optical Zoom:	1.8x
Digital Zoom:	1x
Focus level:	405
Resolution:	5 x 5
Exposure time:	262 ms
Exposure mode:	Normal

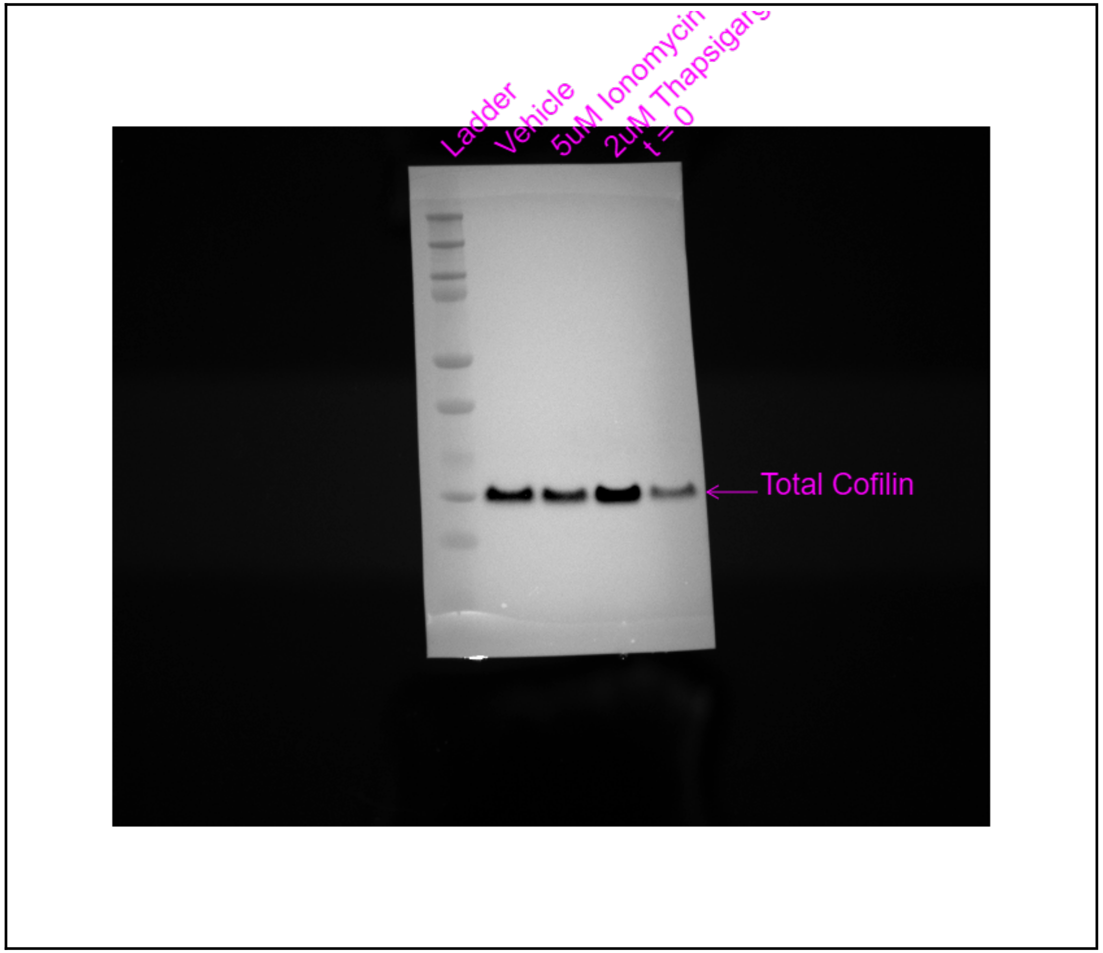

total Cofilin CHEMI_02242022_125217

Date:	24 February 2022 12:52:17PM
Mode:	Chemi Blots
Notes:	
Model:	FL1500
Instrument name:	2462619090234
Serial No:	2462619090234
Firmware version:	1.6.0
iBA version:	5.1.0
Image size:	676px X 540px
Image area:	125.22mm X 100.18mm
Optical Zoom:	1.8x
Digital Zoom:	1x
Focus level:	405
Resolution:	5 x 5
Exposure time:	262 ms
Exposure mode:	Normal

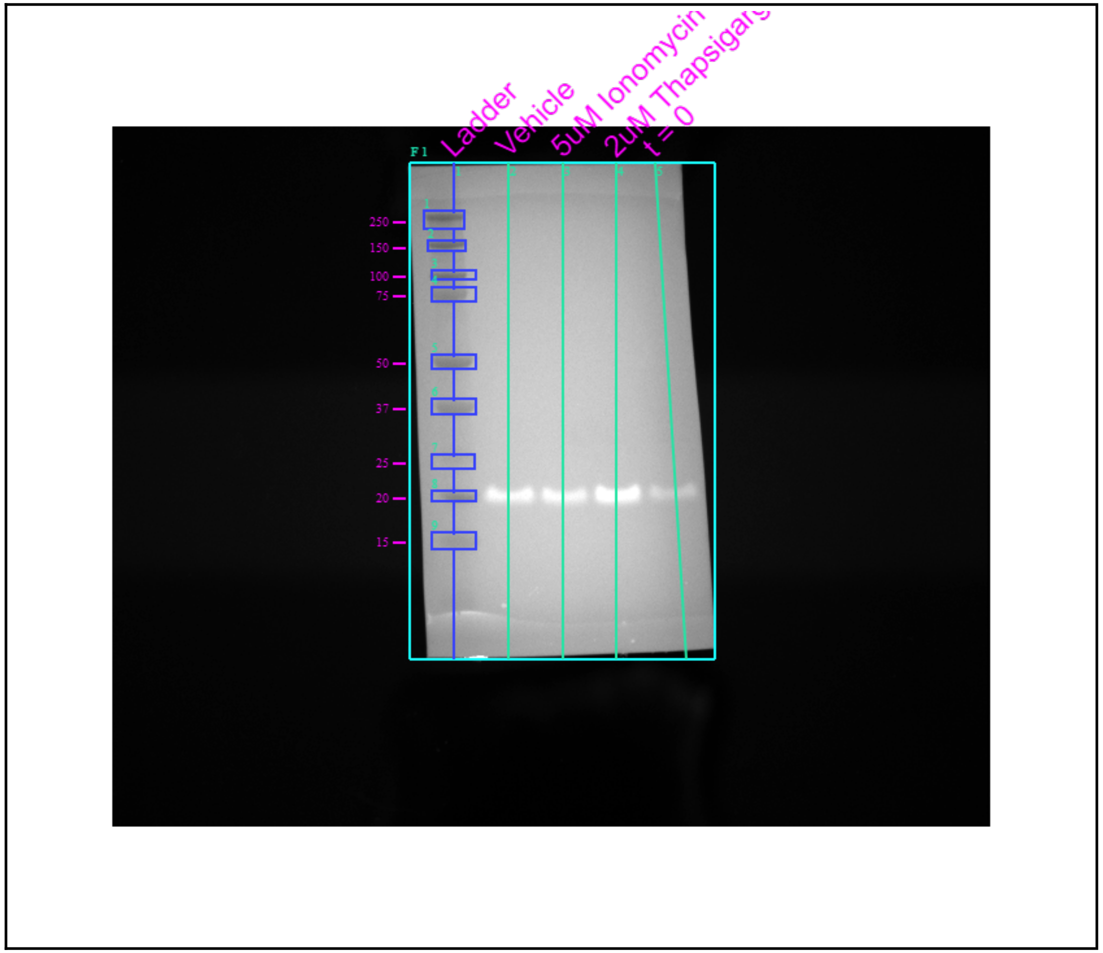

total Cofilin CHEMI_02242022_125217

Date:	24 February 2022 12:52:17PM
Mode:	Chemi Blots
Notes:	
Model:	FL1500
Instrument name:	2462619090234
Serial No:	2462619090234
Firmware version:	1.6.0
iBA version:	5.1.0
Image size:	676px X 540px
Image area:	125.22mm X 100.18mm
Optical Zoom:	1.8x
Digital Zoom:	1x
Focus level:	405
Resolution:	5 x 5
Exposure time:	262 ms
Exposure mode:	Normal

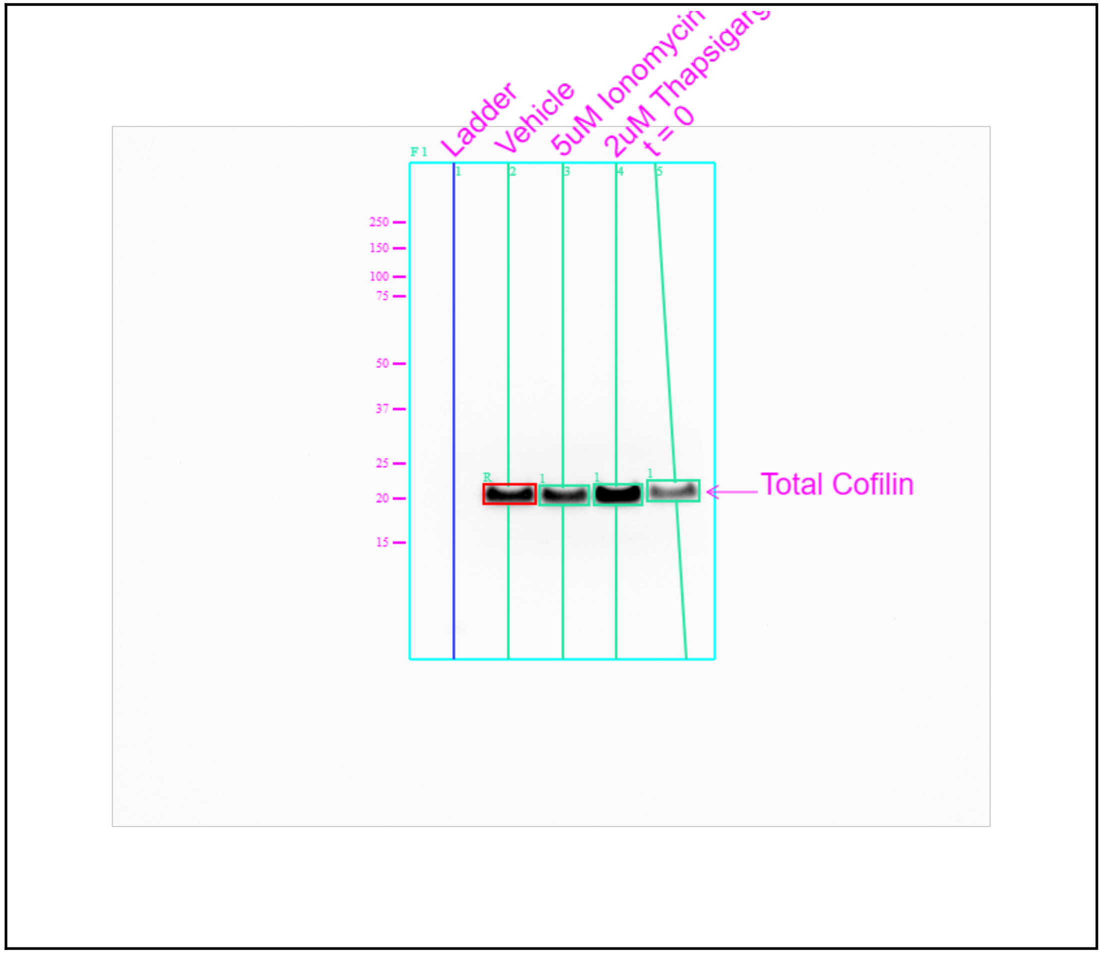

LANE AND BAND ANALYSIS DATA TABLE

total Cofilin CHEMI_02242022_125217

Frame: 1
Channel: Membrane
Sensitivity: 100
Molecular Weight Analysis Regression Method : Point to Point

Lane 1 - Ladder

#	Vol. (Int.)	Local Bg. Corr. Vol.	Area	Rf	Density	Local Bg. Corr. Den.	% band purity	% lane purity	Rolling Bg. Corr. Vol.	Rolling Bg. Corr. Den.	Mol. Wt.
1	17,108,090	2,150,171	480	0.115	35,641	4,479.524	15.685	6.349	2,396,672	4,993.067	250
2	9,663,358	1,550,944	270	0.167	35,790	5,744.239	11.131	4.506	1,700,864	6,299.496	150
3	9,265,794	1,557,348	280	0.225	33,092	5,561.958	9.797	3.966	1,497,088	5,346.743	100
4	12,962,665	1,755,805	420	0.264	30,863	4,180.488	10.774	4.361	1,646,336	3,919.848	75
5	12,989,755	2,341,337	420	0.399	30,927	5,574.613	15.269	6.181	2,333,184	5,555.2	50
6	13,268,061	2,037,369	455	0.491	29,160	4,477.734	13.497	5.463	2,062,336	4,532.607	37
7	10,769,602	601,931	408	0.601	26,396	1,475.322	4.711	1.907	719,872	1,764.392	25
8	9,415,894	1,463,391	315	0.671	29,891	4,645.686	10.004	4.049	1,528,576	4,852.622	20
9	13,869,946	1,243,990	490	0.76	28,306	2,538.756	9.132	3.697	1,395,456	2,847.869	15

Frame: 1
Channel: Chemi
Sensitivity: 100
Molecular Weight Analysis Regression Method : Point to Point

Lane 2 - Vehicle

#	Vol. (Int.)	Local Bg. Corr. Vol.	Area	Rf	Density	Local Bg. Corr. Den.
1	12,348,467	10,376,353	656	0.666	18,823	15,817

#	% band purity	% lane purity	Rolling Bg. Corr. Vol.	Rolling Bg. Corr. Den.	Mol. Wt.	Rel. Quant. (w/ LB Corr. Vol.)
1	100	97.477	11,511,040	17,547	20.37	1

Lane 3 - 5uM Ionomycin

#	Vol. (Int.)	Local Bg. Corr. Vol.	Area	Rf	Density	Local Bg. Corr. Den.
1	10,199,657	7,951,115	624	0.668	16,345	12,742

#	% band purity	% lane purity	Rolling Bg. Corr. Vol.	Rolling Bg. Corr. Den.	Mol. Wt.	Rel. Quant. (w/ LB Corr. Vol.)

#	% band purity	% lane purity	Rolling Bg. Corr. Vol.	Rolling Bg. Corr. Den.	Mol. Wt.	Rel. Quant. (w/ LB Corr. Vol.)
1	100	94.203	9,500,928	15,225	20.185	0.766

Lane 4 - 2uM Thapsigargin

#	Vol. (Int.)	Local Bg. Corr. Vol.	Area	Rf	Density	Local Bg. Corr. Den.
1	16,979,096	15,168,375	646	0.668	26,283	23,480

#	% band purity	% lane purity	Rolling Bg. Corr. Vol.	Rolling Bg. Corr. Den.	Mol. Wt.	Rel. Quant. (w/ LB Corr. Vol.)
1	100	96.225	16,464,384	25,486	20.185	1.462

Lane 5 - t = 0

#	Vol. (Int.)	Local Bg. Corr. Vol.	Area	Rf	Density	Local Bg. Corr. Den.
1	6,722,713	5,759,864	697	0.661	9,645.212	8,263.794

#	% band purity	% lane purity	Rolling Bg. Corr. Vol.	Rolling Bg. Corr. Den.	Mol. Wt.	Rel. Quant. (w/ LB Corr. Vol.)
1	100	98.718	6,189,312	8,879.931	20.741	0.555

iBright™ Image Analysis Report

Katarina+ Chang
19 November 2022

detyrosinated tubulin CHEMI_0223202 2_115125

Date:	23 February 2022 11:51:25AM
Mode:	Chemi Blots
Notes:	
Model:	FL1500
Instrument name:	2462619090234
Serial No:	2462619090234
Firmware version:	1.6.0
iBA version:	5.1.0
Image size:	676px X 540px
Image area:	118.63mm X 94.91mm
Optical Zoom:	1.9x
Digital Zoom:	1x
Focus level:	430
Resolution:	5 x 5
Exposure time:	37400 ms
Exposure mode:	Normal

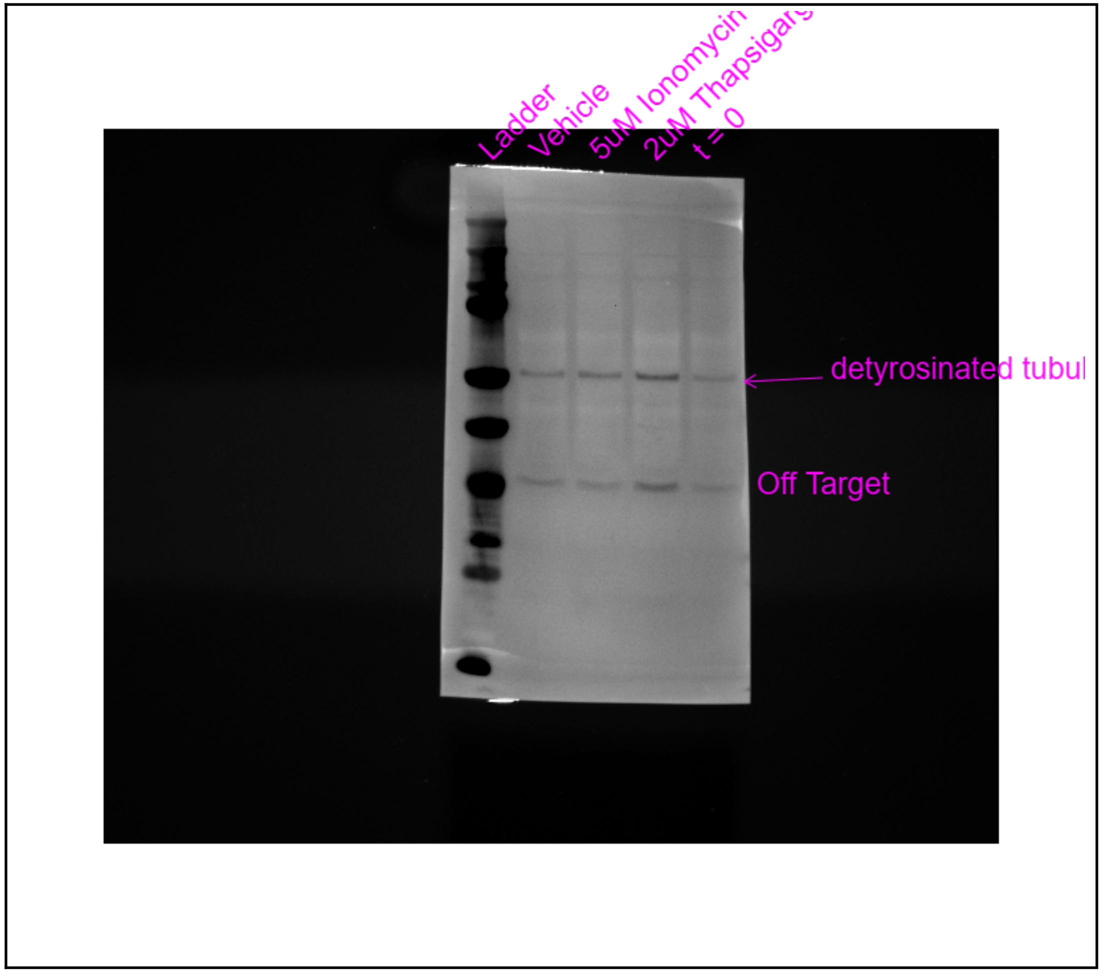

detyrosinated tubulin CHEMI_0223202 2_115125

Date:	23 February 2022 11:51:25AM
Mode:	Chemi Blots
Notes:	
Model:	FL1500
Instrument name:	2462619090234
Serial No:	2462619090234
Firmware version:	1.6.0
iBA version:	5.1.0
Image size:	676px X 540px
Image area:	118.63mm X 94.91mm
Optical Zoom:	1.9x
Digital Zoom:	1x
Focus level:	430
Resolution:	5 x 5
Exposure time:	37400 ms
Exposure mode:	Normal

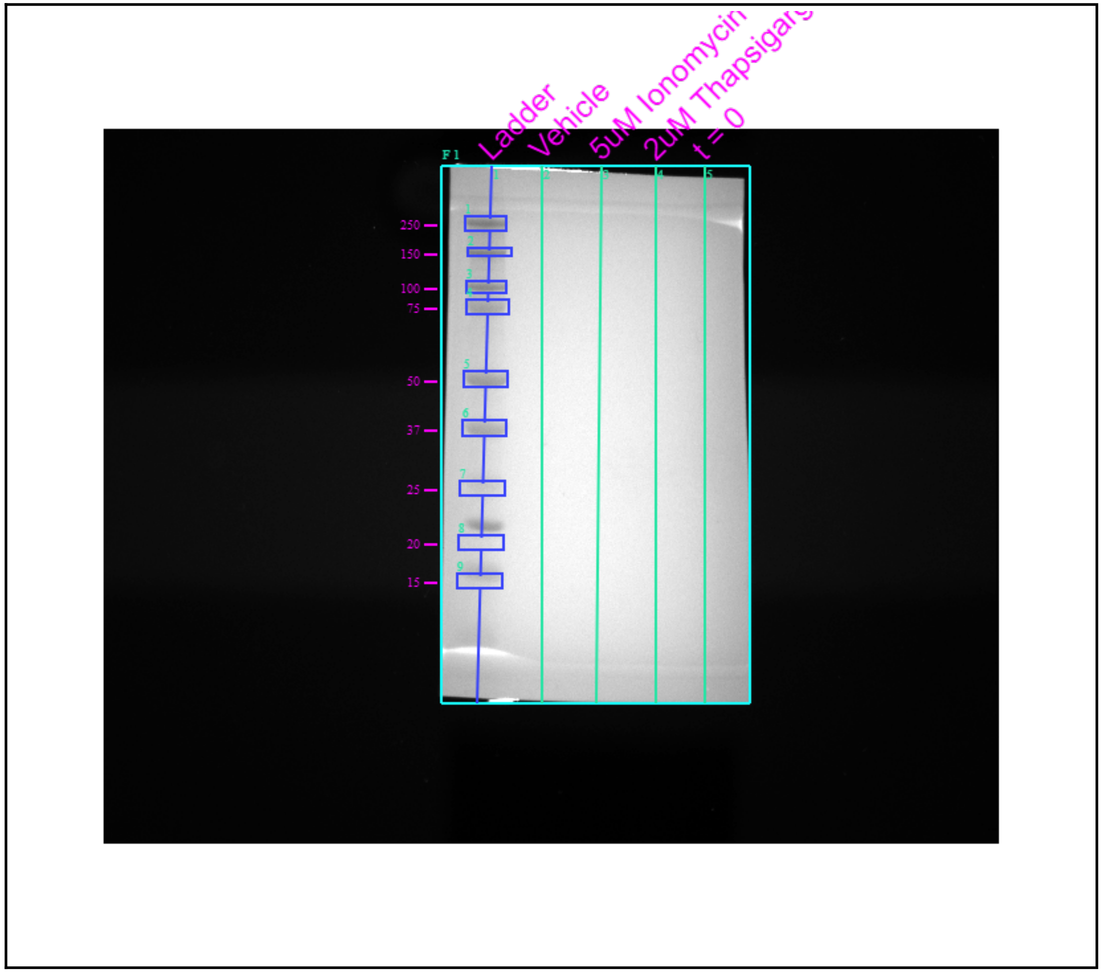

detyrosinated tubulin CHEMI_02232022_115125

Date:	23 February 2022 11:51:25AM
Mode:	Chemi Blots
Notes:	
Model:	FL1500
Instrument name:	2462619090234
Serial No:	2462619090234
Firmware version:	1.6.0
iBA version:	5.1.0
Image size:	676px X 540px
Image area:	118.63mm X 94.91mm
Optical Zoom:	1.9x
Digital Zoom:	1x
Focus level:	430
Resolution:	5 x 5
Exposure time:	37400 ms
Exposure mode:	Normal

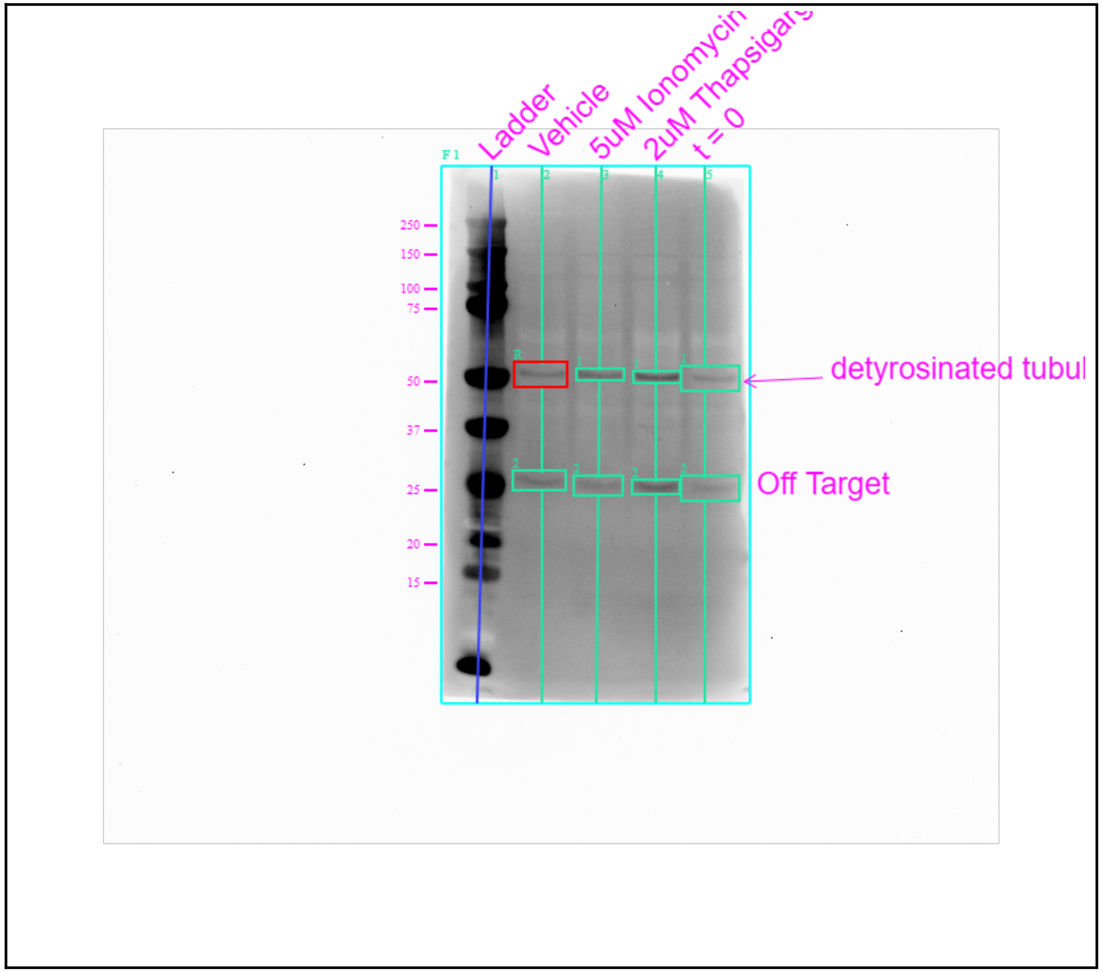

LANE AND BAND ANALYSIS DATA TABLE

detyrosinated tubulin CHEMI_02232022_115125

Frame: 1
Channel: Membrane
Sensitivity: 100
Molecular Weight Analysis Regression Method : Point to Point

Lane 1 - Ladder

#	Vol. (Int.)	Local Bg. Corr. Vol.	Area	Rf	Density	Local Bg. Corr. Den.	% band purity	% lane purity	Rolling Bg. Corr. Vol.	Rolling Bg. Corr. Den.	Mol. Wt.
1	13,706,070	2,077,858	384	0.106	35,692	5,411.091	16.139	2.779	2,403,840	6,260	250
2	8,375,700	1,549,853	238	0.16	35,192	6,511.992	9.857	1.697	1,468,160	6,168.739	150
3	10,383,626	1,748,282	310	0.224	33,495	5,639.62	12.327	2.122	1,836,032	5,922.684	100
4	12,265,929	1,789,357	396	0.261	30,974	4,518.581	11.853	2.041	1,765,376	4,458.02	75
5	13,639,886	2,760,500	442	0.397	30,859	6,245.476	18.735	3.226	2,790,400	6,313.122	50
6	12,935,002	2,323,600	442	0.488	29,264	5,257.015	16.356	2.816	2,436,096	5,511.529	37
7	10,784,635	733,856	420	0.599	25,677	1,747.276	5.875	1.011	875,008	2,083.352	25
8	10,058,751	122,467	420	0.7	23,949	291.59	2.19	0.377	326,144	776.533	20
9	11,137,067	632,266	420	0.771	26,516	1,505.398	6.669	1.148	993,280	2,364.952	15

Frame: 1
Channel: Chemi
Sensitivity: 100
Molecular Weight Analysis Regression Method : Point to Point

Lane 2 - Vehicle

#	Vol. (Int.)	Local Bg. Corr. Vol.	Area	Rf	Density	Local Bg. Corr. Den.
1	26,328,177	770,051	820	0.387	32,107	939.087
2	21,672,421	1,140,071	656	0.584	33,037	1,737.914

#	% band purity	% lane purity	Rolling Bg. Corr. Vol.	Rolling Bg. Corr. Den.	Mol. Wt.	Rel. Quant. (w/ LB Corr. Vol.)
1	66.482	12.97	3,252,224	3,966.127	51.818	1
2	33.518	6.539	1,639,680	2,499.512	26.6	1.481

Lane 3 - 5uM Ionomycin

#	Vol. (Int.)	Local Bg. Corr. Vol.	Area	Rf	Density	Local Bg. Corr. Den.

#	Vol. (Int.)	Local Bg. Corr. Vol.	Area	Rf	Density	Local Bg. Corr. Den.
1	12,103,914	1,309,129	370	0.387	32,713	3,538.189
2	18,642,475	1,142,780	608	0.594	30,661	1,879.574

#	% band purity	% lane purity	Rolling Bg. Corr. Vol.	Rolling Bg. Corr. Den.	Mol. Wt.	Rel. Quant. (w/ LB Corr. Vol.)
1	51.954	7.377	2,127,104	5,748.93	51.818	1.7
2	48.046	6.822	1,967,104	3,235.368	25.533	1.484

Lane 4 - 2uM Thapsigargin

#	Vol. (Int.)	Local Bg. Corr. Vol.	Area	Rf	Density	Local Bg. Corr. Den.
1	12,621,633	2,327,782	370	0.392	34,112	6,291.304
2	15,073,615	2,123,601	444	0.596	33,949	4,782.886

#	% band purity	% lane purity	Rolling Bg. Corr. Vol.	Rolling Bg. Corr. Den.	Mol. Wt.	Rel. Quant. (w/ LB Corr. Vol.)
1	54.699	7.474	3,115,520	8,420.324	50.909	3.023
2	45.301	6.19	2,580,224	5,811.315	25.267	2.758

Lane 5 - t = 0

#	Vol. (Int.)	Local Bg. Corr. Vol.	Area	Rf	Density	Local Bg. Corr. Den.
1	22,466,851	3,930,856	900	0.394	24,963	4,367.618
2	23,273,142	3,475,224	900	0.599	25,859	3,861.361

#	% band purity	% lane purity	Rolling Bg. Corr. Vol.	Rolling Bg. Corr. Den.	Mol. Wt.	Rel. Quant. (w/ LB Corr. Vol.)
1	55.145	6.024	1,705,216	1,894.684	50.455	5.105
2	44.855	4.9	1,387,008	1,541.12	25	4.513

iBright™ Image Analysis Report

Katarina+ Chang

19 November 2022

GAPDH_CHEMI_02252022_151419

Date:	25 February 2022 03:14:19PM
Mode:	Chemi Blots
Notes:	
Model:	FL1500
Instrument name:	2462619090234
Serial No:	2462619090234
Firmware version:	1.6.0
iBA version:	5.1.0
Image size:	676px X 540px
Image area:	118.63mm X 94.91mm
Optical Zoom:	1.9x
Digital Zoom:	1x
Focus level:	430
Resolution:	5 x 5
Exposure time:	20820 ms
Exposure mode:	Normal

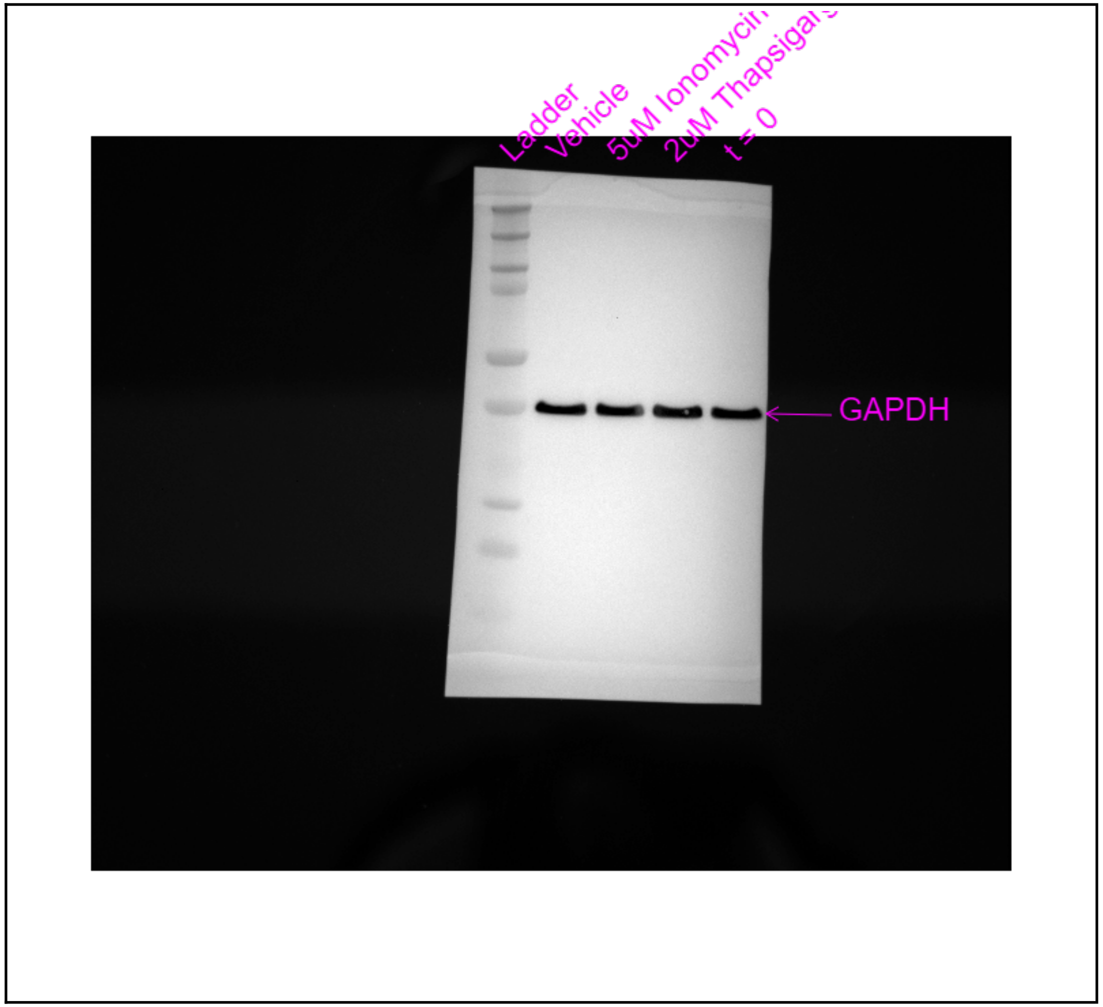

GAPDH_CHEMI_02252022_151419

Date:	25 February 2022 03:14:19PM
Mode:	Chemi Blots
Notes:	
Model:	FL1500
Instrument name:	2462619090234
Serial No:	2462619090234
Firmware version:	1.6.0
iBA version:	5.1.0
Image size:	676px X 540px
Image area:	118.63mm X 94.91mm
Optical Zoom:	1.9x
Digital Zoom:	1x
Focus level:	430
Resolution:	5 x 5
Exposure time:	20820 ms
Exposure mode:	Normal

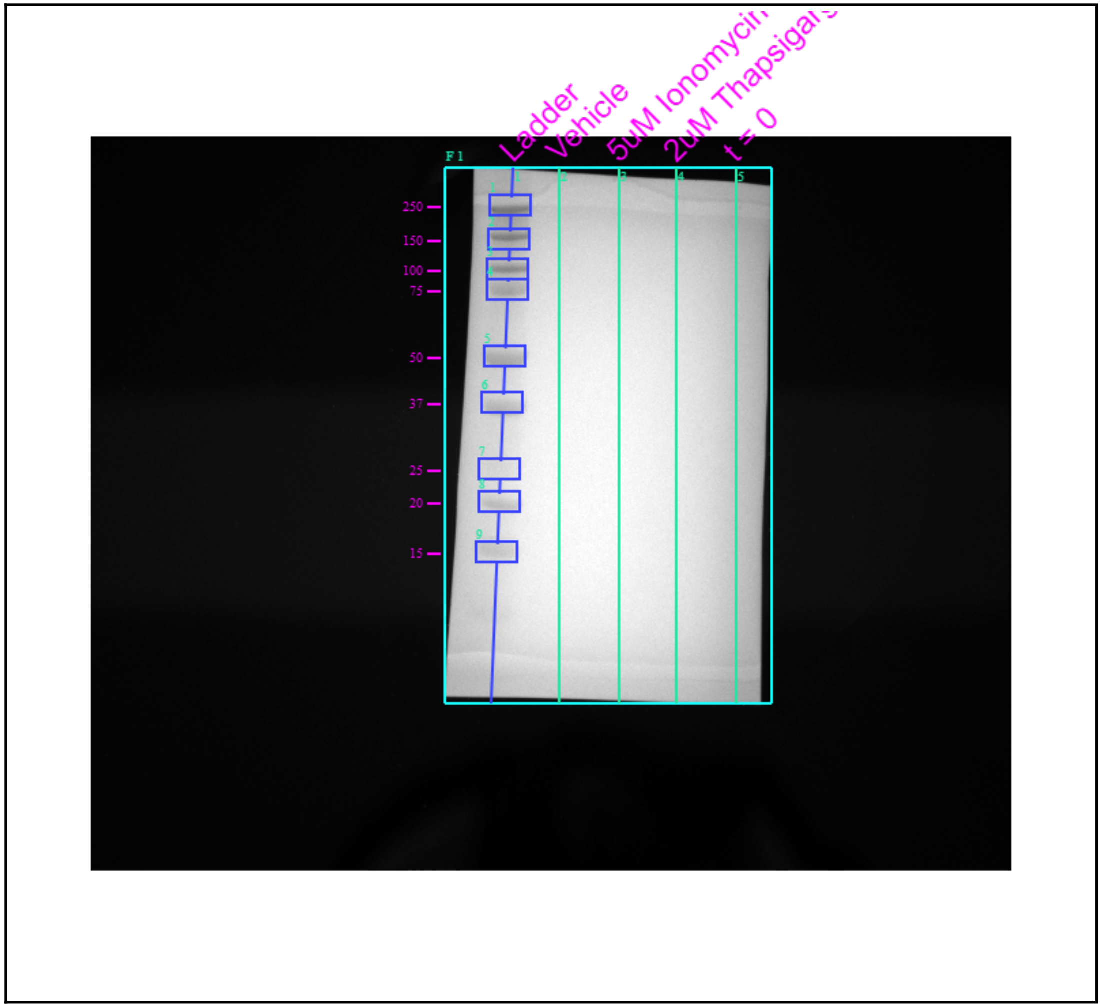

GAPDH_CHEMI_02252022_151419

Date:	25 February 2022 03:14:19PM
Mode:	Chemi Blots
Notes:	
Model:	FL1500
Instrument name:	2462619090234
Serial No:	2462619090234
Firmware version:	1.6.0
iBA version:	5.1.0
Image size:	676px X 540px
Image area:	118.63mm X 94.91mm
Optical Zoom:	1.9x
Digital Zoom:	1x
Focus level:	430
Resolution:	5 x 5
Exposure time:	20820 ms
Exposure mode:	Normal

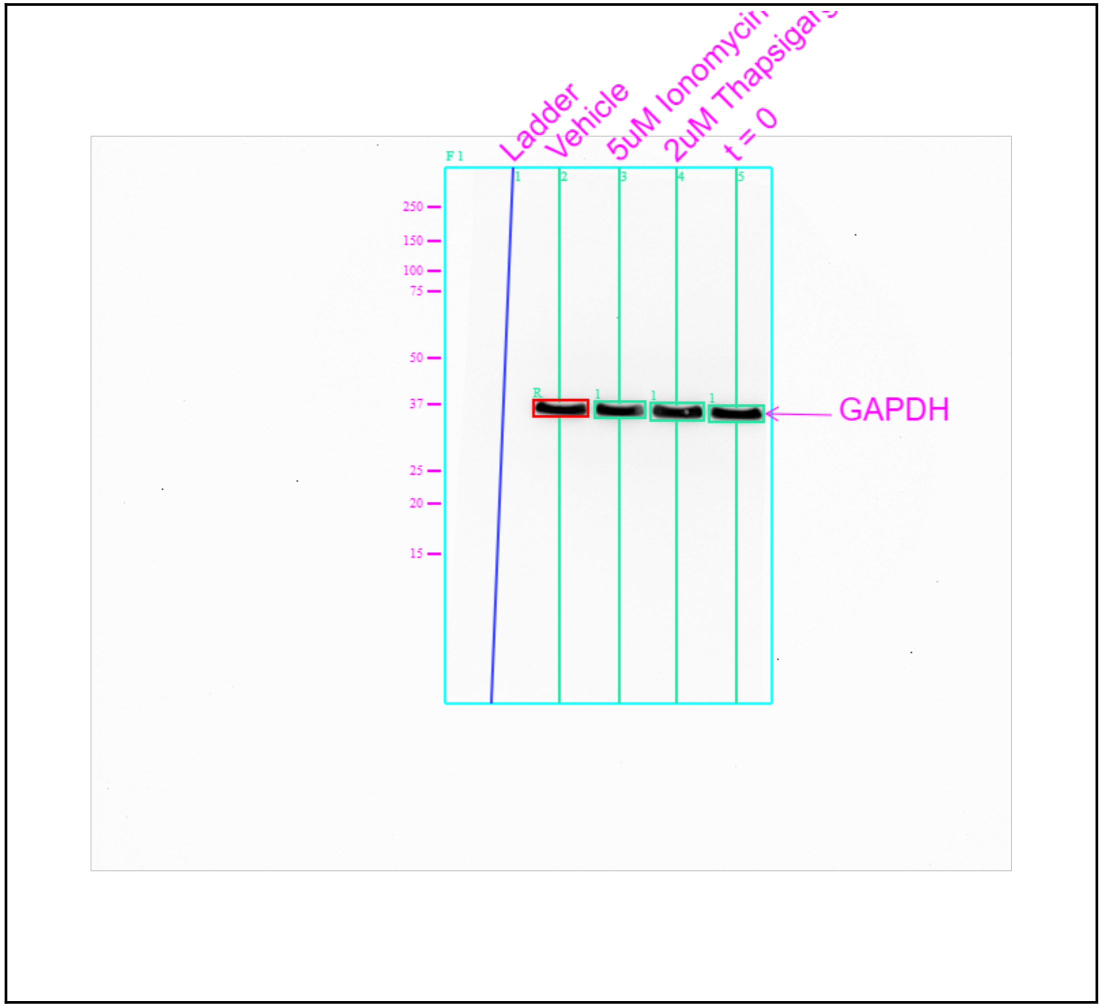

LANE AND BAND ANALYSIS DATA TABLE

GAPDH_CHEMI_02252022_151419

Frame: 1
Channel: Membrane
Sensitivity: 100
Molecular Weight Analysis Regression Method : Point to Point

Lane 1 - Ladder

#	Vol. (Int.)	Local Bg. Corr. Vol.	Area	Rf	Density	Local Bg. Corr. Den.	% band purity	% lane purity	Rolling Bg. Corr. Vol.	Rolling Bg. Corr. Den.	Mol. Wt.
1	17,576,462	1,792,522	496	0.069	35,436	3,613.958	15.724	5.208	2,467,840	4,975.484	250
2	17,155,219	2,036,534	496	0.132	34,587	4,105.915	15.024	4.976	2,358,016	4,754.065	150
3	16,227,124	1,722,596	496	0.188	32,715	3,472.977	10.943	3.624	1,717,504	3,462.71	100
4	15,306,037	1,491,040	496	0.226	30,858	3,006.131	8.299	2.749	1,302,528	2,626.065	75
5	14,858,559	2,291,243	496	0.35	29,956	4,619.443	15.341	5.081	2,407,680	4,854.194	50
6	13,731,536	1,513,388	496	0.437	27,684	3,051.186	11.442	3.79	1,795,840	3,620.645	37
7	12,034,476	516,797	496	0.561	24,263	1,041.931	4.04	1.338	634,112	1,278.452	25
8	12,831,561	1,376,180	496	0.622	25,870	2,774.558	9.363	3.101	1,469,440	2,962.581	20
9	12,987,752	1,285,910	496	0.716	26,184	2,592.561	9.824	3.254	1,541,888	3,108.645	15

Frame: 1
Channel: Chemi
Sensitivity: 100
Molecular Weight Analysis Regression Method : Point to Point

Lane 2 - Vehicle

#	Vol. (Int.)	Local Bg. Corr. Vol.	Area	Rf	Density	Local Bg. Corr. Den.
1	13,647,247	11,659,969	533	0.449	25,604	21,876

#	% band purity	% lane purity	Rolling Bg. Corr. Vol.	Rolling Bg. Corr. Den.	Mol. Wt.	Rel. Quant. (w/ LB Corr. Vol.)
1	100	89.21	12,616,448	23,670	35.776	1

Lane 3 - 5uM Ionomycin

#	Vol. (Int.)	Local Bg. Corr. Vol.	Area	Rf	Density	Local Bg. Corr. Den.
1	12,519,992	10,389,709	494	0.452	25,344	21,031

#	% band purity	% lane purity	Rolling Bg. Corr. Vol.	Rolling Bg. Corr. Den.	Mol. Wt.	Rel. Quant. (w/ LB Corr. Vol.)

#	% band purity	% lane purity	Rolling Bg. Corr. Vol.	Rolling Bg. Corr. Den.	Mol. Wt.	Rel. Quant. (w/ LB Corr. Vol.)
1	100	84.914	11,446,528	23,171	35.531	0.891

Lane 4 - 2uM Thapsigargin

#	Vol. (Int.)	Local Bg. Corr. Vol.	Area	Rf	Density	Local Bg. Corr. Den.
1	13,657,379	11,371,938	560	0.454	24,388	20,307

#	% band purity	% lane purity	Rolling Bg. Corr. Vol.	Rolling Bg. Corr. Den.	Mol. Wt.	Rel. Quant. (w/ LB Corr. Vol.)
1	100	89.334	12,509,440	22,338	35.286	0.975

Lane 5 - t = 0

#	Vol. (Int.)	Local Bg. Corr. Vol.	Area	Rf	Density	Local Bg. Corr. Den.
1	14,102,982	12,502,694	533	0.459	26,459	23,457

#	% band purity	% lane purity	Rolling Bg. Corr. Vol.	Rolling Bg. Corr. Den.	Mol. Wt.	Rel. Quant. (w/ LB Corr. Vol.)
1	100	90.788	13,286,144	24,927	34.796	1.072

iBright™ Image Analysis Report

Katarina+ Chang

19 November 2022

pMYPT1 CHEMI_02242022_124307

Date:	24 February 2022 12:43:07PM
Mode:	Chemi Blots
Notes:	
Model:	FL1500
Instrument name:	2462619090234
Serial No:	2462619090234
Firmware version:	1.6.0
iBA version:	5.1.0
Image size:	615px X 491px
Image area:	112.7mm X 90.16mm
Optical Zoom:	2x
Digital Zoom:	1.1x
Focus level:	455
Resolution:	5 x 5
Exposure time:	40000 ms
Exposure mode:	Normal

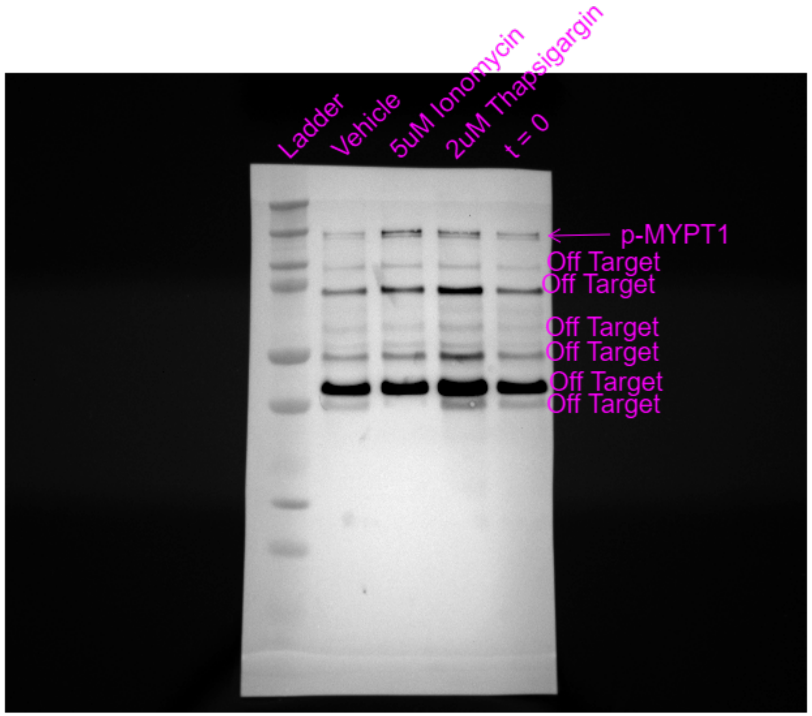

pMYPT1 CHEMI_02242022_124307

Date:	24 February 2022 12:43:07PM
Mode:	Chemi Blots
Notes:	
Model:	FL1500
Instrument name:	2462619090234
Serial No:	2462619090234
Firmware version:	1.6.0
iBA version:	5.1.0
Image size:	615px X 491px
Image area:	112.7mm X 90.16mm
Optical Zoom:	2x
Digital Zoom:	1.1x
Focus level:	455
Resolution:	5 x 5
Exposure time:	40000 ms
Exposure mode:	Normal

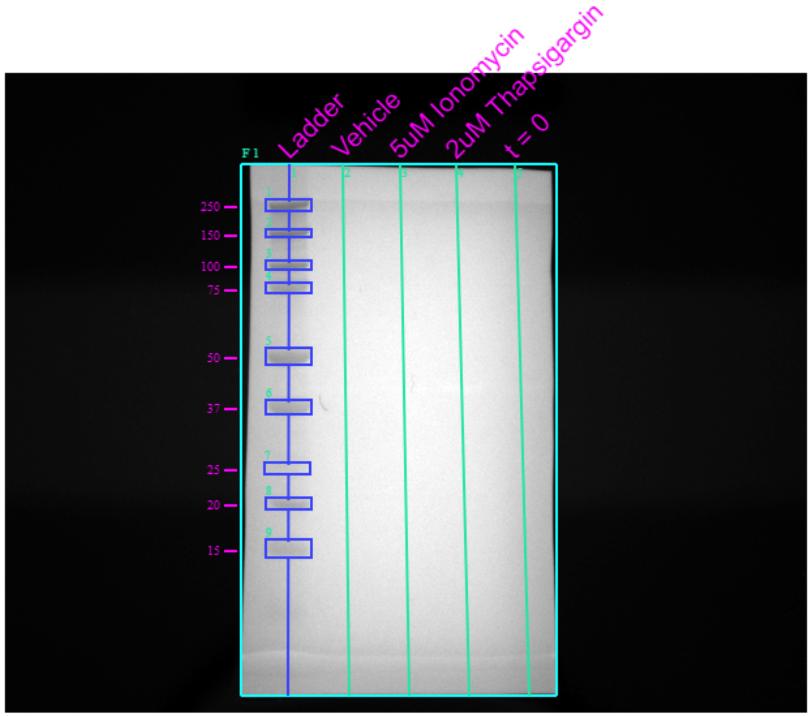

pMYPT1 CHEMI_02242022_124307

Date:	24 February 2022 12:43:07PM
Mode:	Chemi Blots
Notes:	
Model:	FL1500
Instrument name:	2462619090234
Serial No:	2462619090234
Firmware version:	1.6.0
iBA version:	5.1.0
Image size:	615px X 491px
Image area:	112.7mm X 90.16mm
Optical Zoom:	2x
Digital Zoom:	1.1x
Focus level:	455
Resolution:	5 x 5
Exposure time:	40000 ms
Exposure mode:	Normal

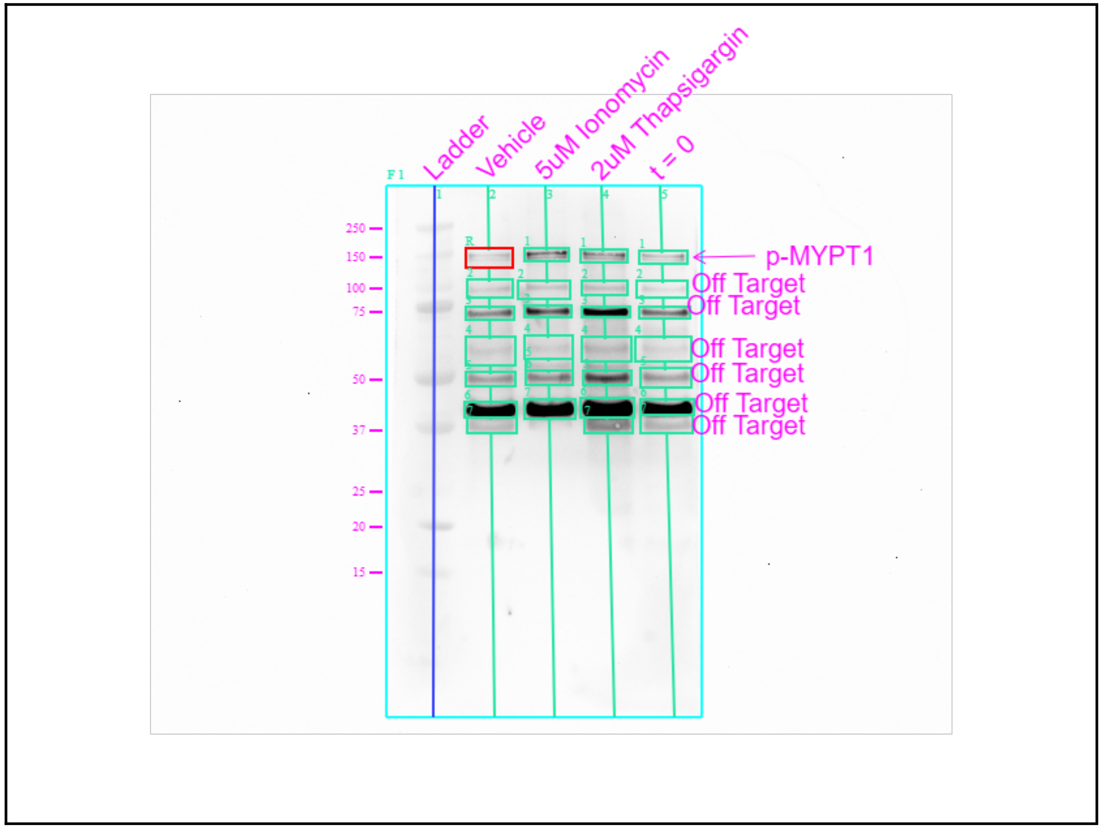

LANE AND BAND ANALYSIS DATA TABLE

pMYPT1 CHEMI_02242022_124307

Frame: 1
Channel: Membrane
Sensitivity: 100
Molecular Weight Analysis Regression Method : Point to Point

Lane 1 - Ladder

#	Vol. (Int.)	Local Bg. Corr. Vol.	Area	Rf	Density	Local Bg. Corr. Den.	% band purity	% lane purity	Rolling Bg. Corr. Vol.	Rolling Bg. Corr. Den.	Mol. Wt.
1	13,252,415	2,136,997	360	0.076	36,812	5,936.105	15.321	3.368	2,448,384	6,801.067	250
2	9,158,275	1,626,473	252	0.13	36,342	6,454.26	11.584	2.547	1,851,136	7,345.778	150
3	9,786,181	1,781,340	288	0.189	33,979	6,185.212	10.309	2.266	1,647,360	5,720	100
4	10,085,255	1,381,534	324	0.233	31,127	4,263.996	8.308	1.827	1,327,616	4,097.58	75
5	15,526,727	2,882,281	504	0.36	30,806	5,718.813	18.496	4.066	2,955,776	5,864.635	50
6	12,927,679	2,031,579	432	0.456	29,925	4,702.73	13.508	2.97	2,158,592	4,996.741	37
7	9,478,288	430,520	360	0.571	26,328	1,195.89	3.161	0.695	505,088	1,403.022	25
8	10,619,203	1,423,162	360	0.637	29,497	3,953.229	9.205	2.024	1,470,976	4,086.044	20
9	15,649,993	1,595,249	540	0.723	28,981	2,954.166	10.108	2.222	1,615,360	2,991.407	15

Frame: 1
Channel: Chemi
Sensitivity: 100
Molecular Weight Analysis Regression Method : Point to Point

Lane 2 - Vehicle

#	Vol. (Int.)	Local Bg. Corr. Vol.	Area	Rf	Density	Local Bg. Corr. Den.
1	4,315,570	1,973,289	592	0.135	7,289.814	3,333.259
2	4,804,766	1,372,782	540	0.194	8,897.715	2,542.189
3	7,833,504	4,662,522	418	0.24	18,740	11,154
4	9,243,590	1,828,848	897	0.311	10,305	2,038.85
5	7,855,221	3,710,178	468	0.363	16,784	7,927.733
6	25,530,871	19,486,950	533	0.422	47,900	36,560
7	7,335,194	156,058	507	0.451	14,467	307.807

#	% band purity	% lane purity	Rolling Bg. Corr. Vol.	Rolling Bg. Corr. Den.	Mol. Wt.	Rel. Quant. (w/ LB Corr. Vol.)

#	% band purity	% lane purity	Rolling Bg. Corr. Vol.	Rolling Bg. Corr. Den.	Mol. Wt.	Rel. Quant. (w/ LB Corr. Vol.)
1	4.473	4.253	1,676,288	2,831.568	145.833	1
2	2.662	2.531	997,632	1,847.467	97.222	0.696
3	11.392	10.831	4,269,056	10,213	73.558	2.363
4	2.931	2.786	1,098,240	1,224.348	59.615	0.927
5	9.618	9.145	3,604,224	7,701.333	49.667	1.88
6	57.655	54.819	21,606,144	40,536	41.667	9.875
7	11.27	10.716	4,223,488	8,330.351	37.667	0.079

Lane 3 - 5uM Ionomycin

#	Vol. (Int.)	Local Bg. Corr. Vol.	Area	Rf	Density	Local Bg. Corr. Den.
1	7,376,875	5,080,486	385	0.13	19,160	13,196
2	6,137,838	1,614,004	615	0.196	9,980.224	2,624.398
3	10,119,092	6,794,814	370	0.235	27,348	18,364
4	7,497,535	1,243,861	722	0.304	10,384	1,722.8
5	4,723,954	246,796	370	0.336	12,767	667.017
6	9,029,973	4,402,955	444	0.36	20,337	9,916.566
7	28,872,413	21,425,279	640	0.419	45,113	33,476

#	% band purity	% lane purity	Rolling Bg. Corr. Vol.	Rolling Bg. Corr. Den.	Mol. Wt.	Rel. Quant. (w/ LB Corr. Vol.)
1	11.96	11.106	5,289,984	13,740	150	2.575
2	3.583	3.327	1,584,896	2,577.067	95.833	0.818
3	15.808	14.679	6,991,872	18,896	74.519	3.443
4	2.043	1.897	903,680	1,251.634	61.058	0.63
5	2.408	2.236	1,065,216	2,878.962	54.808	0.125
6	10.421	9.677	4,609,024	10,380	50	2.231
7	53.776	49.937	23,785,216	37,164	42	10.858

Lane 4 - 2uM Thapsigargin

#	Vol. (Int.)	Local Bg. Corr. Vol.	Area	Rf	Density	Local Bg. Corr. Den.
1	6,606,323	4,312,334	407	0.132	16,231	10,595
2	4,674,605	1,772,732	432	0.191	10,820	4,103.547
3	14,568,844	11,184,098	342	0.238	42,598	32,702
4	10,760,047	2,171,198	780	0.306	13,794	2,783.587

#	Vol. (Int.)	Local Bg. Corr. Vol.	Area	Rf	Density	Local Bg. Corr. Den.	[redacted]
5	13,867,049	7,674,428	456	0.36	30,410	16,829	[redacted]
6	35,235,058	23,417,647	672	0.417	52,433	34,847	[redacted]
7	12,794,247	1,598,949	494	0.451	25,899	3,236.74	[redacted]

#	% band purity	% lane purity	Rolling Bg. Corr. Vol.	Rolling Bg. Corr. Den.	Mol. Wt.	Rel. Quant. (w/ LB Corr. Vol.)
1	7.138	6.77	4,311,296	10,592	147.917	2.185
2	2.084	1.976	1,258,496	2,913.185	98.611	0.898
3	18.643	17.682	11,259,648	32,922	74.038	5.668
4	2.438	2.312	1,472,512	1,887.836	60.577	1.1
5	12.636	11.985	7,631,616	16,736	50	3.889
6	44.471	42.179	26,859,264	39,969	42.333	11.867
7	12.591	11.942	7,604,480	15,393	37.667	0.81

Lane 5 - t = 0

#	Vol. (Int.)	Local Bg. Corr. Vol.	Area	Rf	Density	Local Bg. Corr. Den.	[redacted]
1	3,619,618	2,356,824	418	0.135	8,659.373	5,638.335	[redacted]
2	2,942,115	900,349	560	0.194	5,253.777	1,607.768	[redacted]
3	7,543,364	5,634,225	400	0.238	18,858	14,085	[redacted]
4	6,902,984	1,020,066	880	0.306	7,844.3	1,159.166	[redacted]
5	8,931,693	4,189,823	640	0.36	13,955	6,546.6	[redacted]
6	26,550,626	20,808,170	546	0.417	48,627	38,110	[redacted]
7	9,436,738	762,559	615	0.449	15,344	1,239.934	[redacted]

#	% band purity	% lane purity	Rolling Bg. Corr. Vol.	Rolling Bg. Corr. Den.	Mol. Wt.	Rel. Quant. (w/ LB Corr. Vol.)
1	5.775	5.706	2,454,016	5,870.852	145.833	1.194
2	1.993	1.969	846,848	1,512.229	97.222	0.456
3	13.052	12.898	5,546,496	13,866	74.038	2.855
4	2.527	2.497	1,073,664	1,220.073	60.577	0.517
5	9.978	9.86	4,240,384	6,625.6	50	2.123
6	53.579	52.946	22,768,896	41,701	42.333	10.545
7	13.097	12.942	5,565,696	9,049.912	38	0.386

iBright™ Image Analysis Report

Katarina+ Chang

19 November 2022

GAPDH CHEMI_02252022_125908

Date:	25 February 2022 12:59:08PM
Mode:	Chemi Blots
Notes:	
Model:	FL1500
Instrument name:	2462619090234
Serial No:	2462619090234
Firmware version:	1.6.0
iBA version:	5.1.0
Image size:	676px X 540px
Image area:	125.22mm X 100.18mm
Optical Zoom:	1.8x
Digital Zoom:	1x
Focus level:	405
Resolution:	5 x 5
Exposure time:	370 ms
Exposure mode:	Normal

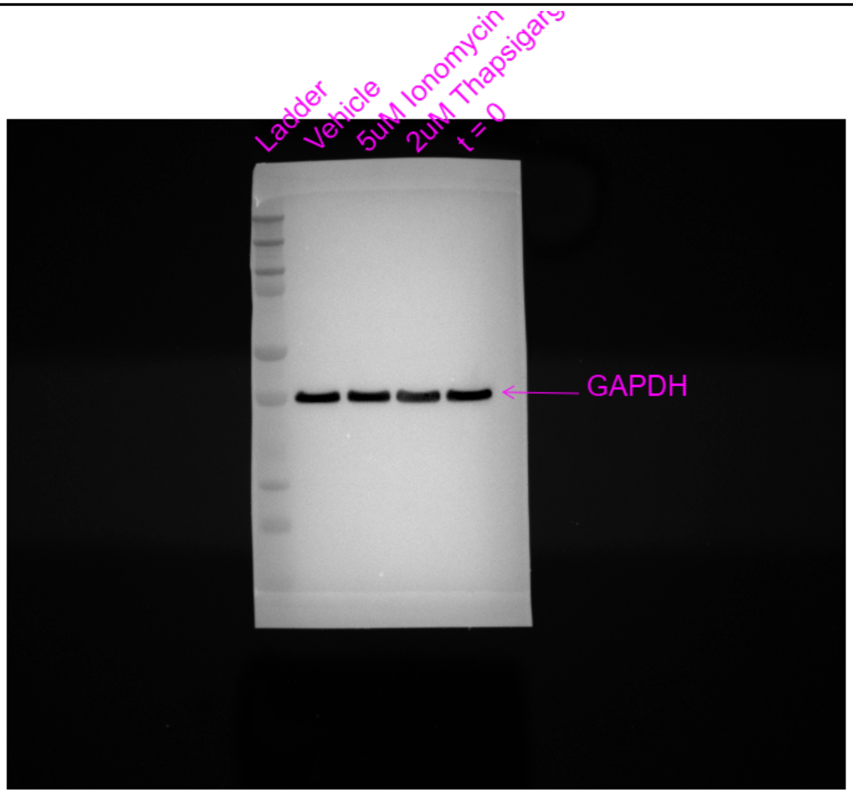

GAPDH CHEMI_02252022_125908

Date:	25 February 2022 12:59:08PM
Mode:	Chemi Blots
Notes:	
Model:	FL1500
Instrument name:	2462619090234
Serial No:	2462619090234
Firmware version:	1.6.0
iBA version:	5.1.0
Image size:	676px X 540px
Image area:	125.22mm X 100.18mm
Optical Zoom:	1.8x
Digital Zoom:	1x
Focus level:	405
Resolution:	5 x 5
Exposure time:	370 ms
Exposure mode:	Normal

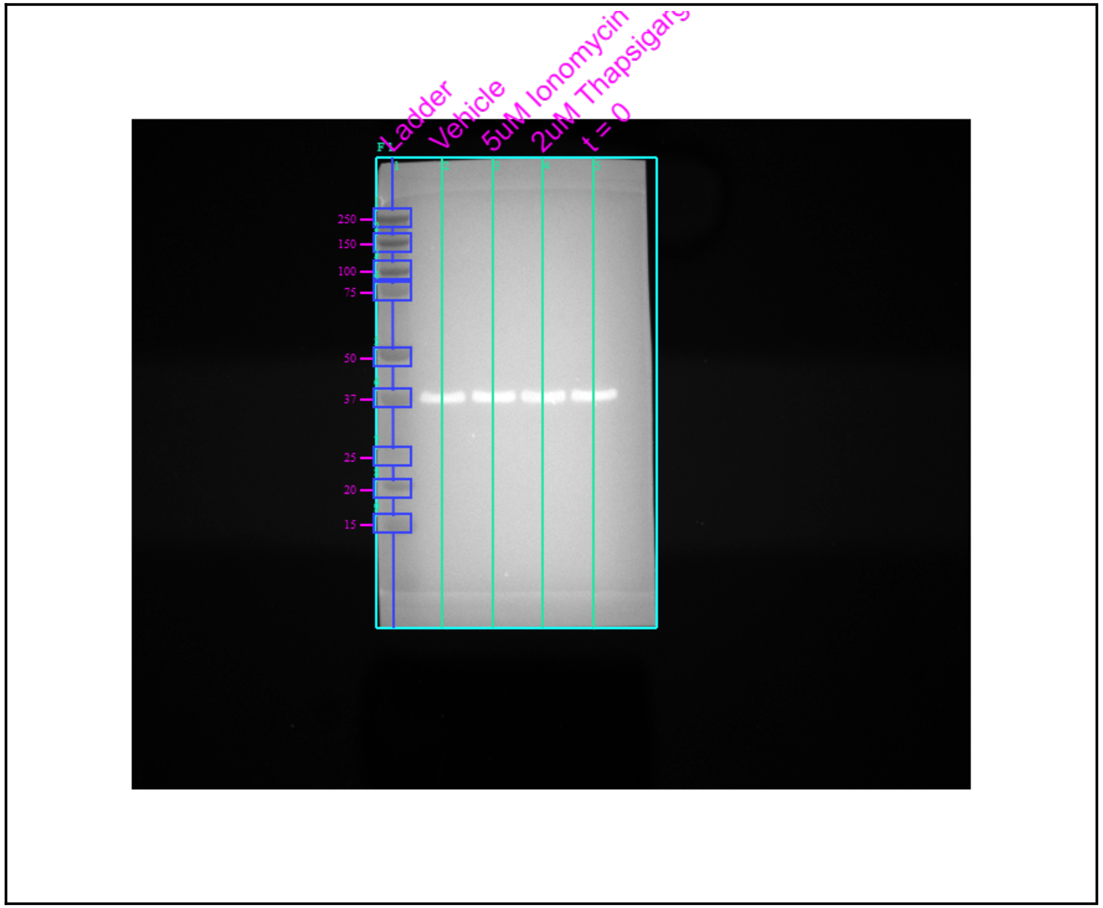

GAPDH CHEMI_02252022_125908

Date:	25 February 2022 12:59:08PM
Mode:	Chemi Blots
Notes:	
Model:	FL1500
Instrument name:	2462619090234
Serial No:	2462619090234
Firmware version:	1.6.0
iBA version:	5.1.0
Image size:	676px X 540px
Image area:	125.22mm X 100.18mm
Optical Zoom:	1.8x
Digital Zoom:	1x
Focus level:	405
Resolution:	5 x 5
Exposure time:	370 ms
Exposure mode:	Normal

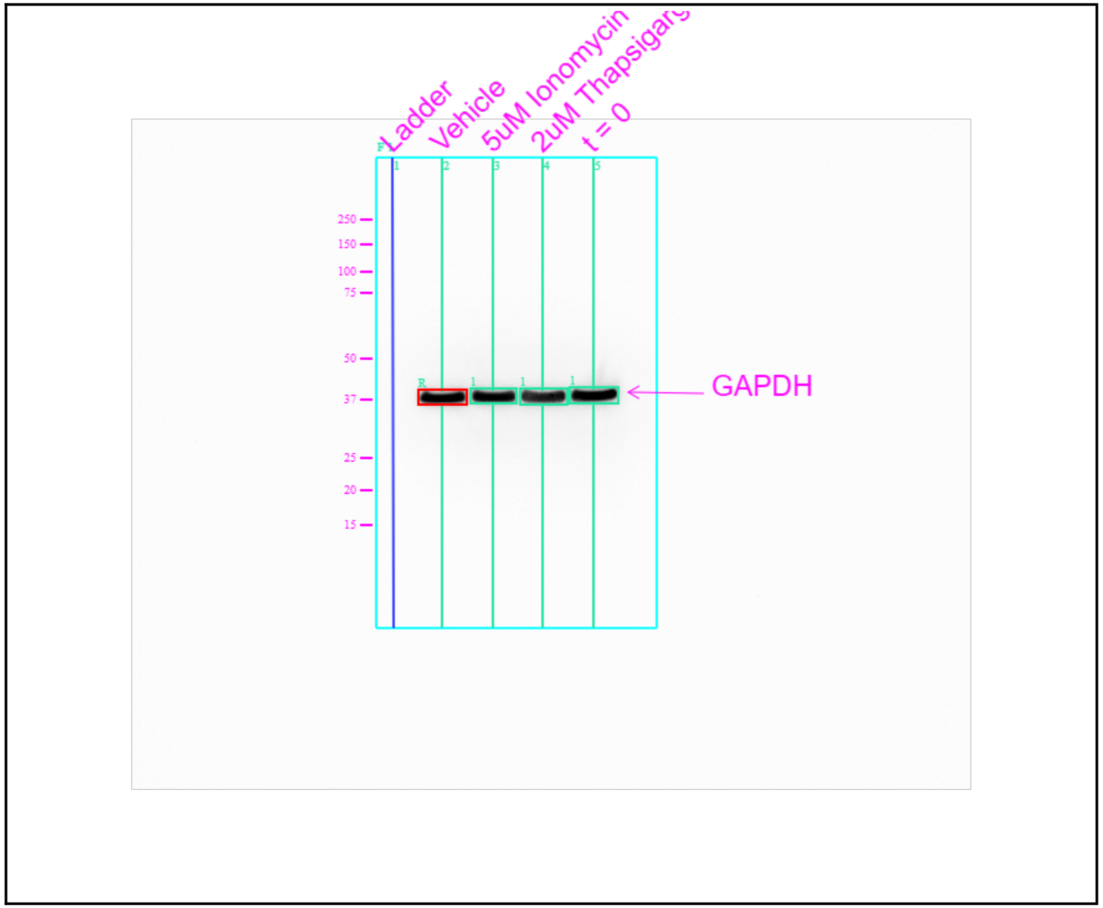

LANE AND BAND ANALYSIS DATA TABLE

GAPDH CHEMI_02252022_125908

Frame: 1
Channel: Membrane
Sensitivity: 100
Molecular Weight Analysis Regression Method : Point to Point

Lane 1 - Ladder

#	Vol. (Int.)	Local Bg. Corr. Vol.	Area	Rf	Density	Local Bg. Corr. Den.	% band purity	% lane purity	Rolling Bg. Corr. Vol.	Rolling Bg. Corr. Den.	Mol. Wt.
1	19,668,929	859,833	496	0.127	39,655	1,733.536	16.506	2.382	2,386,688	4,811.871	250
2	19,155,875	702,090	496	0.179	38,620	1,415.504	13.882	2.004	2,007,296	4,046.968	150
3	18,376,330	629,009	496	0.237	37,049	1,268.165	10.016	1.446	1,448,192	2,919.742	100
4	17,838,814	860,369	496	0.282	35,965	1,734.616	8.748	1.263	1,264,896	2,550.194	75
5	17,383,210	780,669	496	0.422	35,046	1,573.931	12.508	1.805	1,808,640	3,646.452	50
6	16,970,172	864,729	496	0.509	34,214	1,743.407	11.374	1.642	1,644,544	3,315.613	37
7	15,894,058	781,027	496	0.633	32,044	1,574.653	7.67	1.107	1,108,992	2,235.871	25
8	16,390,781	694,997	496	0.702	33,045	1,401.204	9.608	1.387	1,389,312	2,801.032	20
9	16,848,331	851,221	496	0.776	33,968	1,716.173	9.688	1.398	1,400,832	2,824.258	15

Frame: 1
Channel: Chemi
Sensitivity: 100
Molecular Weight Analysis Regression Method : Point to Point

Lane 2 - Vehicle

#	Vol. (Int.)	Local Bg. Corr. Vol.	Area	Rf	Density	Local Bg. Corr. Den.
1	13,999,350	11,831,736	520	0.509	26,921	22,753

#	% band purity	% lane purity	Rolling Bg. Corr. Vol.	Rolling Bg. Corr. Den.	Mol. Wt.	Rel. Quant. (w/ LB Corr. Vol.)
1	100	90.53	13,304,064	25,584	37	1

Lane 3 - 5uM Ionomycin

#	Vol. (Int.)	Local Bg. Corr. Vol.	Area	Rf	Density	Local Bg. Corr. Den.
1	13,071,382	10,469,625	494	0.507	26,460	21,193

#	% band purity	% lane purity	Rolling Bg. Corr. Vol.	Rolling Bg. Corr. Den.	Mol. Wt.	Rel. Quant. (w/ LB Corr. Vol.)

#	% band purity	% lane purity	Rolling Bg. Corr. Vol.	Rolling Bg. Corr. Den.	Mol. Wt.	Rel. Quant. (w/ LB Corr. Vol.)
1	100	89.277	12,059,136	24,411	37.394	0.885

Lane 4 - 2uM Thapsigargin

#	Vol. (Int.)	Local Bg. Corr. Vol.	Area	Rf	Density	Local Bg. Corr. Den.	
1	12,742,150	9,660,214	546	0.507	23,337	17,692	

#	% band purity	% lane purity	Rolling Bg. Corr. Vol.	Rolling Bg. Corr. Den.	Mol. Wt.	Rel. Quant. (w/ LB Corr. Vol.)
1	100	91.281	11,621,632	21,285	37.394	0.816

Lane 5 - t = 0

#	Vol. (Int.)	Local Bg. Corr. Vol.	Area	Rf	Density	Local Bg. Corr. Den.	
1	15,399,467	12,886,816	560	0.504	27,499	23,012	

#	% band purity	% lane purity	Rolling Bg. Corr. Vol.	Rolling Bg. Corr. Den.	Mol. Wt.	Rel. Quant. (w/ LB Corr. Vol.)
1	100	90.392	14,407,424	25,727	37.788	1.089

iBright™ Image Analysis Report

Katarina+ Chang

19 November 2022

pMLC CHEMI_02232022_113821

Date:	23 February 2022 11:38:21AM
Mode:	Chemi Blots
Notes:	
Model:	FL1500
Instrument name:	2462619090234
Serial No:	2462619090234
Firmware version:	1.6.0
iBA version:	5.1.0
Image size:	676px X 540px
Image area:	112.7mm X 90.16mm
Optical Zoom:	2x
Digital Zoom:	1x
Focus level:	455
Resolution:	5 x 5
Exposure time:	55210 ms
Exposure mode:	Normal

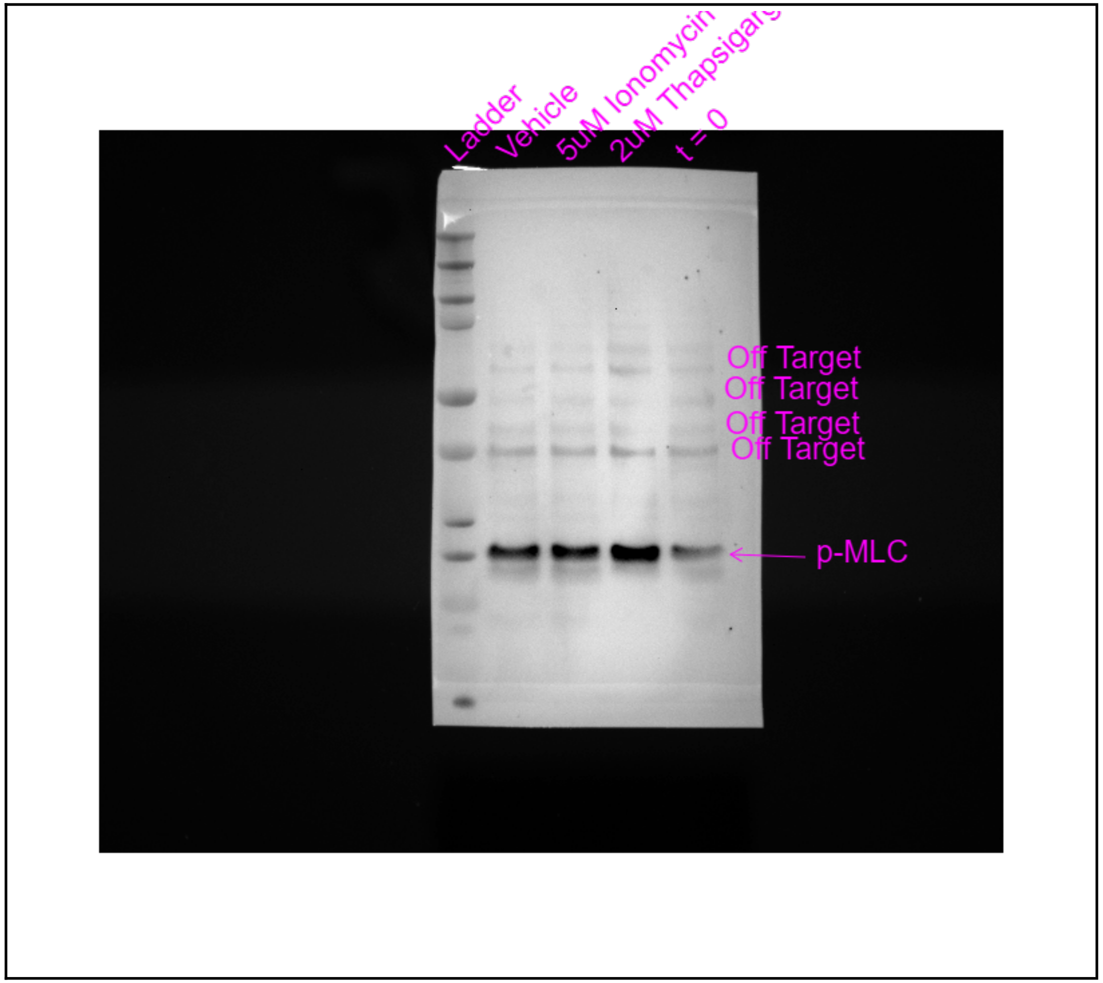

pMLC CHEMI_02232022_113821

Date:	23 February 2022 11:38:21AM
Mode:	Chemi Blots
Notes:	
Model:	FL1500
Instrument name:	2462619090234
Serial No:	2462619090234
Firmware version:	1.6.0
iBA version:	5.1.0
Image size:	676px X 540px
Image area:	112.7mm X 90.16mm
Optical Zoom:	2x
Digital Zoom:	1x
Focus level:	455
Resolution:	5 x 5
Exposure time:	55210 ms
Exposure mode:	Normal

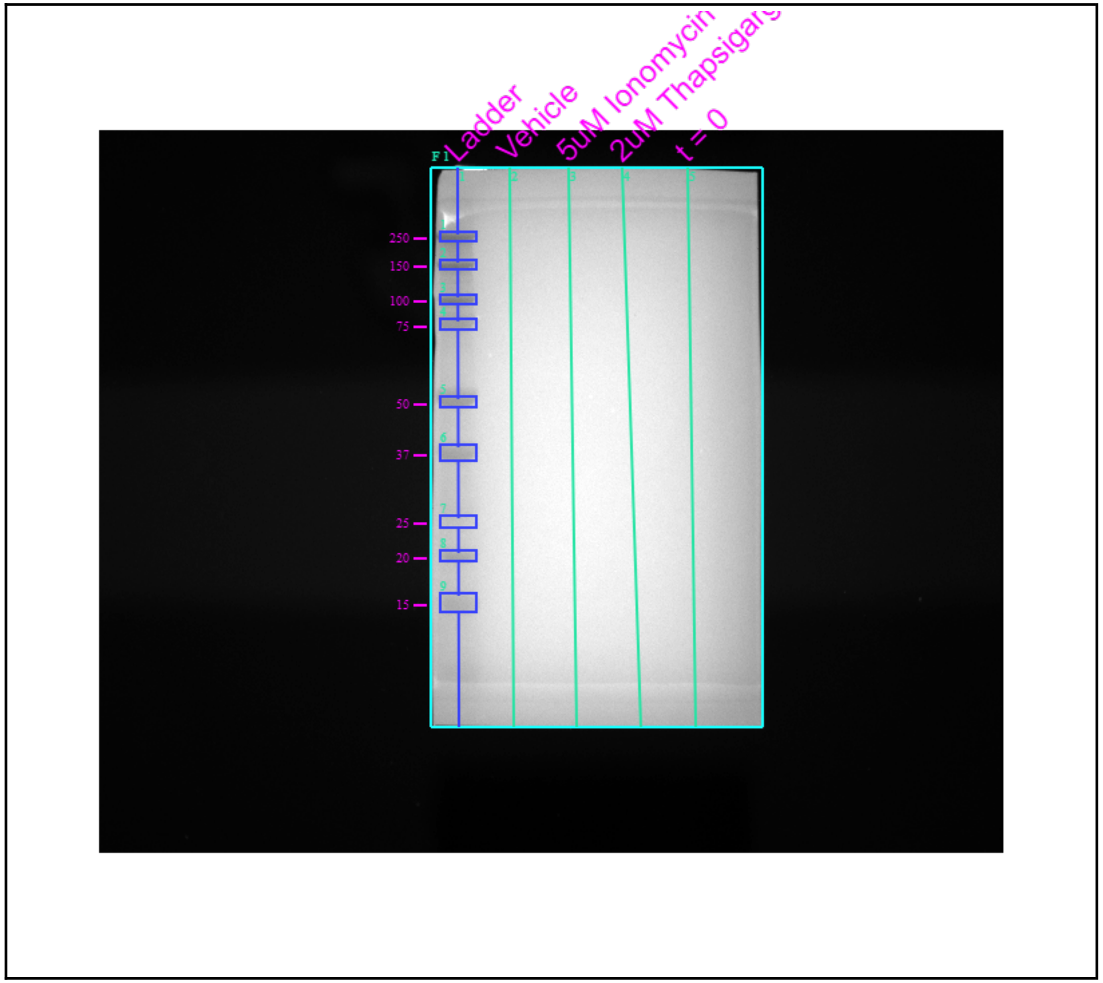

pMLC CHEMI_02232022_113821

Date:	23 February 2022 11:38:21AM
Mode:	Chemi Blots
Notes:	
Model:	FL1500
Instrument name:	2462619090234
Serial No:	2462619090234
Firmware version:	1.6.0
iBA version:	5.1.0
Image size:	676px X 540px
Image area:	112.7mm X 90.16mm
Optical Zoom:	2x
Digital Zoom:	1x
Focus level:	455
Resolution:	5 x 5
Exposure time:	55210 ms
Exposure mode:	Normal

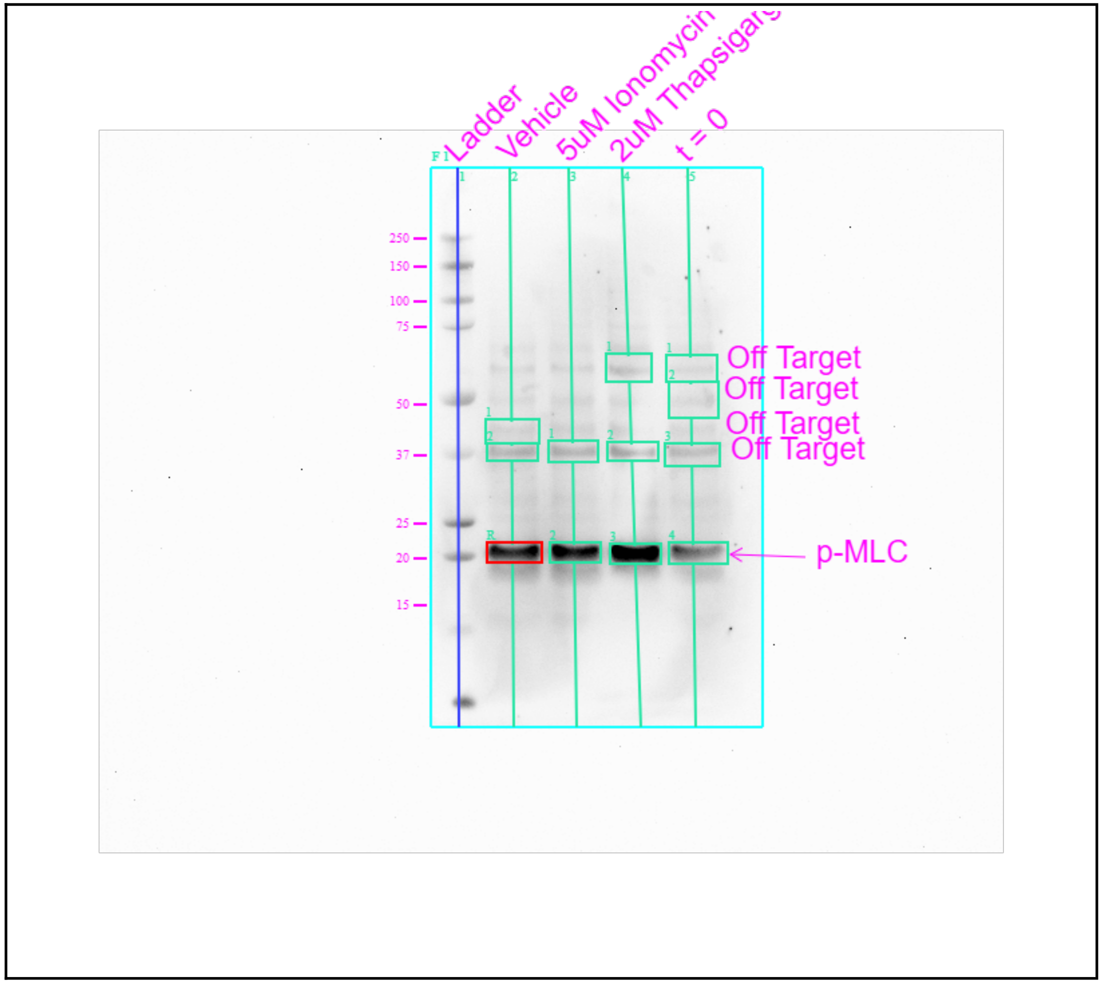

LANE AND BAND ANALYSIS DATA TABLE

pMLC CHEMI_02232022_113821

Frame: 1
Channel: Membrane
Sensitivity: 100
Molecular Weight Analysis Regression Method : Point to Point

Lane 1 - Ladder

#	Vol. (Int.)	Local Bg. Corr. Vol.	Area	Rf	Density	Local Bg. Corr. Den.	% band purity	% lane purity	Rolling Bg. Corr. Vol.	Rolling Bg. Corr. Den.	Mol. Wt.
1	8,744,047	1,015,673	224	0.122	39,035	4,534.255	16.557	2.937	1,898,752	8,476.571	250
2	8,484,604	1,168,969	224	0.172	37,877	5,218.615	10.588	1.878	1,214,208	5,420.571	150
3	8,215,819	1,327,050	224	0.234	36,677	5,924.333	11.275	2	1,293,056	5,772.571	100
4	8,285,595	700,915	252	0.28	32,879	2,781.411	7.304	1.296	837,632	3,323.937	75
5	8,186,460	1,191,979	252	0.419	32,485	4,730.077	13.369	2.372	1,533,184	6,084.063	50
6	11,323,587	1,725,638	364	0.51	31,108	4,740.765	16.135	2.863	1,850,368	5,083.429	37
7	7,666,025	387,679	280	0.632	27,378	1,384.571	3.206	0.569	367,616	1,312.914	25
8	7,754,309	1,081,616	252	0.694	30,771	4,292.131	9.713	1.723	1,113,856	4,420.063	20
9	12,590,605	1,337,869	420	0.778	29,977	3,185.404	11.853	2.103	1,359,360	3,236.571	15

Frame: 1
Channel: Chemi
Sensitivity: 100
Molecular Weight Analysis Regression Method : Point to Point

Lane 2 - Vehicle

#	Vol. (Int.)	Local Bg. Corr. Vol.	Area	Rf	Density	Local Bg. Corr. Den.
1	4,096,200	422,789	779	0.471	5,258.28	542.734
2	4,405,728	1,628,316	546	0.507	8,069.099	2,982.265
3	15,092,859	9,167,138	672	0.687	22,459	13,641

#	% band purity	% lane purity	Rolling Bg. Corr. Vol.	Rolling Bg. Corr. Den.	Mol. Wt.	Rel. Quant. (w/ LB Corr. Vol.)
1	4.229	3.848	554,752	712.134	42.474	0.046
2	12.121	11.029	1,590,016	2,912.117	37.342	0.178
3	83.65	76.116	10,973,184	16,329	20.577	1

Lane 3 - 5uM Ionomycin

#	Vol. (Int.)	Local Bg. Corr. Vol.	Area	Rf	Density	Local Bg. Corr. Den.	[redacted]
1	4,768,574	1,820,388	646	0.507	7,381.693	2,817.939	
2	15,584,899	9,729,086	624	0.687	24,975	15,591	

#	% band purity	% lane purity	Rolling Bg. Corr. Vol.	Rolling Bg. Corr. Den.	Mol. Wt.	Rel. Quant. (w/ LB Corr. Vol.)
1	11.892	9.345	1,571,072	2,432	37.342	0.199
2	88.108	69.234	11,640,064	18,653	20.577	1.061

Lane 4 - 2uM Thapsigargin

#	Vol. (Int.)	Local Bg. Corr. Vol.	Area	Rf	Density	Local Bg. Corr. Den.	[redacted]
1	3,723,426	1,334,044	770	0.356	4,835.618	1,732.525	
2	3,609,723	1,736,051	585	0.507	6,170.467	2,967.609	
3	20,616,432	15,031,633	624	0.689	33,039	24,089	

#	% band purity	% lane purity	Rolling Bg. Corr. Vol.	Rolling Bg. Corr. Den.	Mol. Wt.	Rel. Quant. (w/ LB Corr. Vol.)
1	4.651	4.384	951,296	1,235.449	61.207	0.146
2	8.725	8.224	1,784,320	3,050.12	37.342	0.189
3	86.624	81.65	17,715,968	28,390	20.385	1.64

Lane 5 - t = 0

#	Vol. (Int.)	Local Bg. Corr. Vol.	Area	Rf	Density	Local Bg. Corr. Den.	[redacted]
1	3,128,077	855,726	819	0.359	3,819.386	1,044.843	
2	4,230,732	1,072,210	1,064	0.414	3,976.252	1,007.717	
3	4,902,180	1,856,918	756	0.512	6,484.365	2,456.242	
4	10,076,668	5,548,646	765	0.689	13,172	7,253.133	

#	% band purity	% lane purity	Rolling Bg. Corr. Vol.	Rolling Bg. Corr. Den.	Mol. Wt.	Rel. Quant. (w/ LB Corr. Vol.)
1	4.018	3.277	365,568	446.359	60.776	0.093
2	6.79	5.537	617,728	580.571	50.862	0.117
3	18.547	15.124	1,687,296	2,231.873	36.765	0.203
4	70.644	57.606	6,426,624	8,400.816	20.385	0.605

iBright™ Image Analysis Report

Katarina+ Chang
19 November 2022

Acetyl Tubulin CHEMI_03012022_11482 1

Date:	1 March 2022 11:48:21AM
Mode:	Chemi Blots
Notes:	
Model:	FL1500
Instrument name:	2462619090234
Serial No:	2462619090234
Firmware version:	1.6.0
iBA version:	5.1.0
Image size:	676px X 540px
Image area:	118.63mm X 94.91mm
Optical Zoom:	1.9x
Digital Zoom:	1x
Focus level:	430
Resolution:	5 x 5
Exposure time:	2302 ms
Exposure mode:	Normal

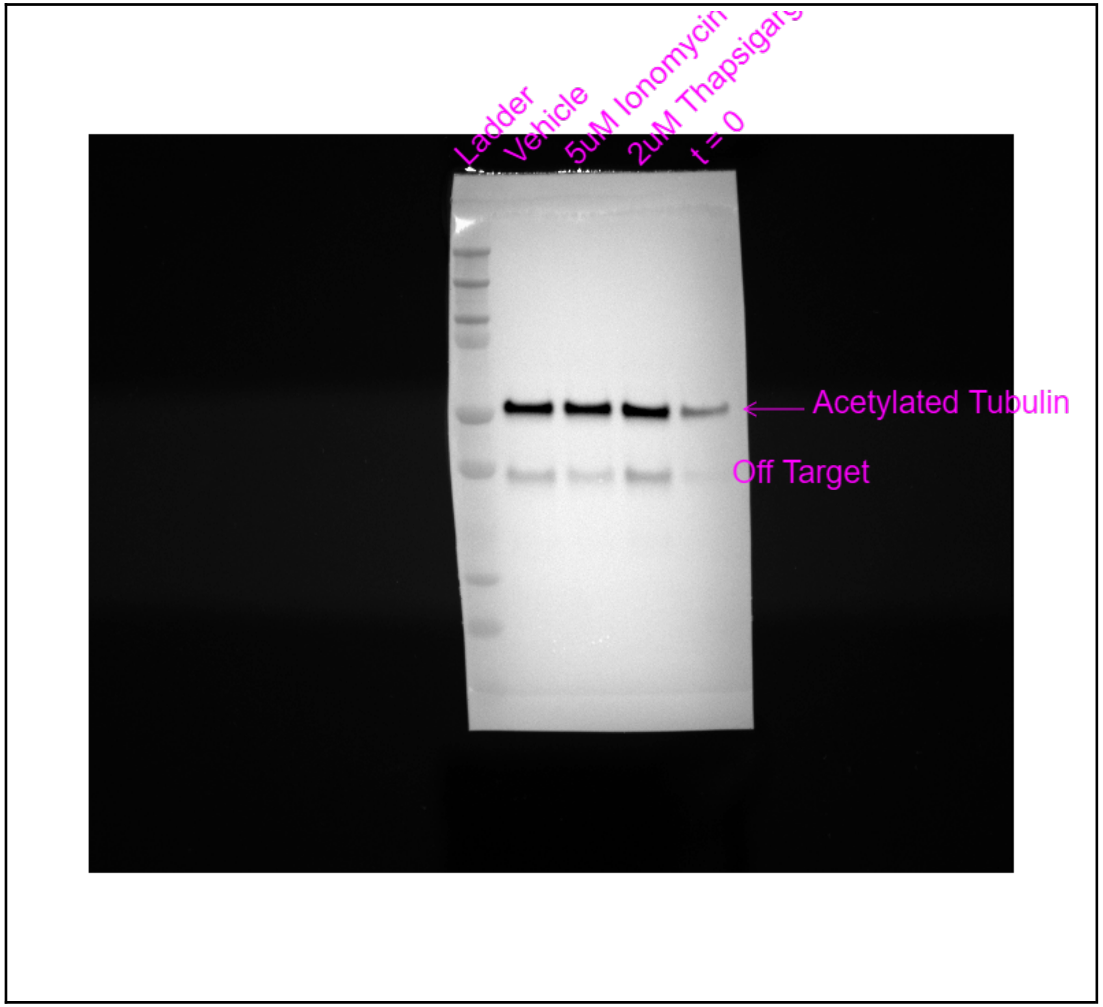

Acetyl Tubulin CHEMI_03012022_11482 1

Date:	1 March 2022 11:48:21AM
Mode:	Chemi Blots
Notes:	
Model:	FL1500
Instrument name:	2462619090234
Serial No:	2462619090234
Firmware version:	1.6.0
iBA version:	5.1.0
Image size:	676px X 540px
Image area:	118.63mm X 94.91mm
Optical Zoom:	1.9x
Digital Zoom:	1x
Focus level:	430
Resolution:	5 x 5
Exposure time:	2302 ms
Exposure mode:	Normal

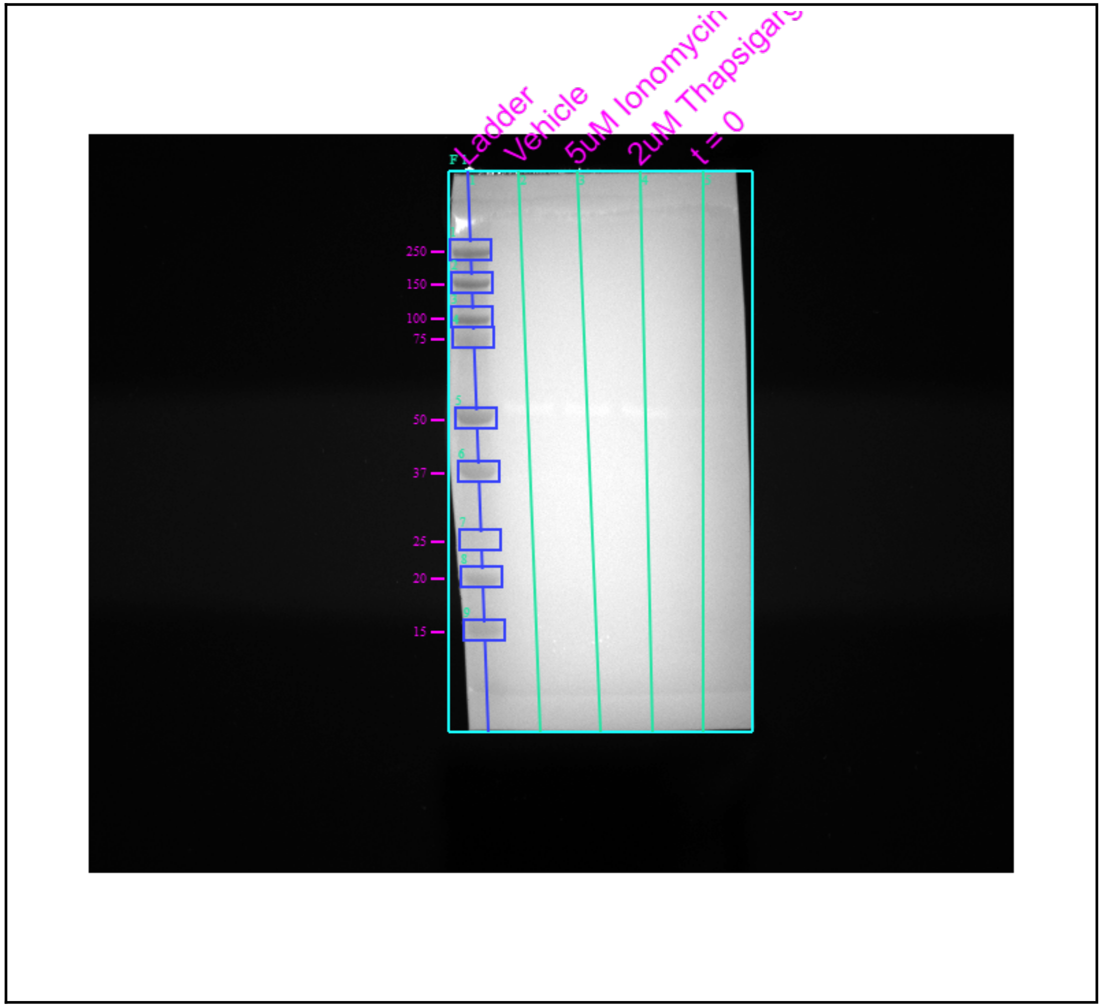

Acetyl Tubulin CHEMI_03012022_11482 1

Date:	1 March 2022 11:48:21AM
Mode:	Chemi Blots
Notes:	
Model:	FL1500
Instrument name:	2462619090234
Serial No:	2462619090234
Firmware version:	1.6.0
iBA version:	5.1.0
Image size:	676px X 540px
Image area:	118.63mm X 94.91mm
Optical Zoom:	1.9x
Digital Zoom:	1x
Focus level:	430
Resolution:	5 x 5
Exposure time:	2302 ms
Exposure mode:	Normal

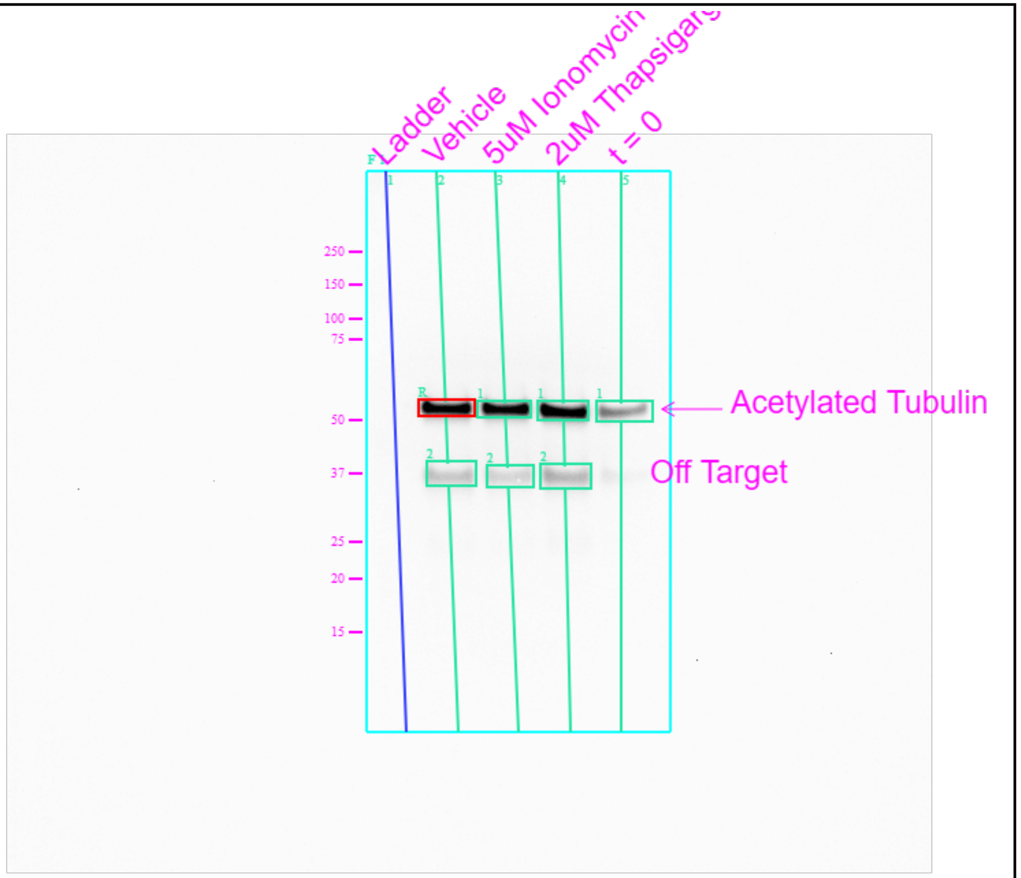

LANE AND BAND ANALYSIS DATA TABLE

Acetyl Tubulin CHEMI_03012022_114821

Frame: 1
Channel: Membrane
Sensitivity: 100
Molecular Weight Analysis Regression Method : Point to Point

Lane 1 - Ladder

#	Vol. (Int.)	Local Bg. Corr. Vol.	Area	Rf	Density	Local Bg. Corr. Den.	% band purity	% lane purity	Rolling Bg. Corr. Vol.	Rolling Bg. Corr. Den.	Mol. Wt.
1	17,308,187	262,116	496	0.139	34,895	528.461	18.983	5.219	3,006,464	6,061.419	250
2	16,934,073	627,770	496	0.198	34,141	1,265.666	11.633	3.198	1,842,432	3,714.581	150
3	16,379,111	629,408	496	0.259	33,022	1,268.968	11.417	3.139	1,808,128	3,645.419	100
4	15,443,832	208,505	496	0.295	31,136	420.374	8.286	2.278	1,312,256	2,645.677	75
5	15,446,802	2,497,651	496	0.439	31,142	5,035.587	15.948	4.384	2,525,696	5,092.129	50
6	14,651,844	2,092,797	496	0.534	29,540	4,219.35	13.284	3.652	2,103,808	4,241.548	37
7	13,467,879	164.951	496	0.656	27,152	0.333	3.624	0.996	573,952	1,157.161	25
8	14,604,632	78,996	496	0.722	29,444	159.268	8.084	2.222	1,280,256	2,581.161	20
9	15,577,700	63,518	496	0.817	31,406	128.061	8.742	2.403	1,384,448	2,791.226	15

Frame: 1
Channel: Chemi
Sensitivity: 100
Molecular Weight Analysis Regression Method : Point to Point

Lane 2 - Vehicle

#	Vol. (Int.)	Local Bg. Corr. Vol.	Area	Rf	Density	Local Bg. Corr. Den.	[redacted]
1	11,058,926	9,468,078	546	0.422	20,254	17,340	[redacted]
2	2,774,709	1,785,041	703	0.539	3,946.954	2,539.177	[redacted]

#	% band purity	% lane purity	Rolling Bg. Corr. Vol.	Rolling Bg. Corr. Den.	Mol. Wt.	Rel. Quant. (w/ LB Corr. Vol.)
1	83.464	76.84	10,360,320	18,974	52.966	1
2	16.536	15.224	2,052,608	2,919.784	36.52	0.189

Lane 3 - 5uM Ionomycin

#	Vol. (Int.)	Local Bg. Corr. Vol.	Area	Rf	Density	Local Bg. Corr. Den.	[redacted]

#	Vol. (Int.)	Local Bg. Corr. Vol.	Area	Rf	Density	Local Bg. Corr. Den.
1	10,416,024	8,792,839	520	0.424	20,030	16,909
2	1,926,451	1,160,000	595	0.544	3,237.733	1,949.581

#	% band purity	% lane purity	Rolling Bg. Corr. Vol.	Rolling Bg. Corr. Den.	Mol. Wt.	Rel. Quant. (w/ LB Corr. Vol.)
1	89.275	80.361	9,802,496	18,850	52.542	0.929
2	10.725	9.654	1,177,600	1,979.16	36.04	0.123

Lane 4 - 2uM Thapsigargin

#	Vol. (Int.)	Local Bg. Corr. Vol.	Area	Rf	Density	Local Bg. Corr. Den.
1	11,700,859	10,299,155	570	0.427	20,527	18,068
2	3,554,086	2,650,645	722	0.544	4,922.557	3,671.254

#	% band purity	% lane purity	Rolling Bg. Corr. Vol.	Rolling Bg. Corr. Den.	Mol. Wt.	Rel. Quant. (w/ LB Corr. Vol.)
1	80.86	74.816	11,115,776	19,501	52.119	1.088
2	19.14	17.709	2,631,168	3,644.277	36.04	0.28

Lane 5 - t = 0

#	Vol. (Int.)	Local Bg. Corr. Vol.	Area	Rf	Density	Local Bg. Corr. Den.
1	4,005,692	3,472,821	672	0.427	5,960.851	5,167.889

#	% band purity	% lane purity	Rolling Bg. Corr. Vol.	Rolling Bg. Corr. Den.	Mol. Wt.	Rel. Quant. (w/ LB Corr. Vol.)
1	100	80.358	3,665,664	5,454.857	52.119	0.367

iBright™ Image Analysis Report

Katarina+ Chang

19 November 2022

GAPDH CHEMI_03022022_105936

Date:	2 March 2022 10:59:36AM
Mode:	Chemi Blots
Notes:	
Model:	FL1500
Instrument name:	2462619090234
Serial No:	2462619090234
Firmware version:	1.6.0
iBA version:	5.1.0
Image size:	615px X 491px
Image area:	112.7mm X 90.16mm
Optical Zoom:	2x
Digital Zoom:	1.1x
Focus level:	455
Resolution:	5 x 5
Exposure time:	7951 ms
Exposure mode:	Normal

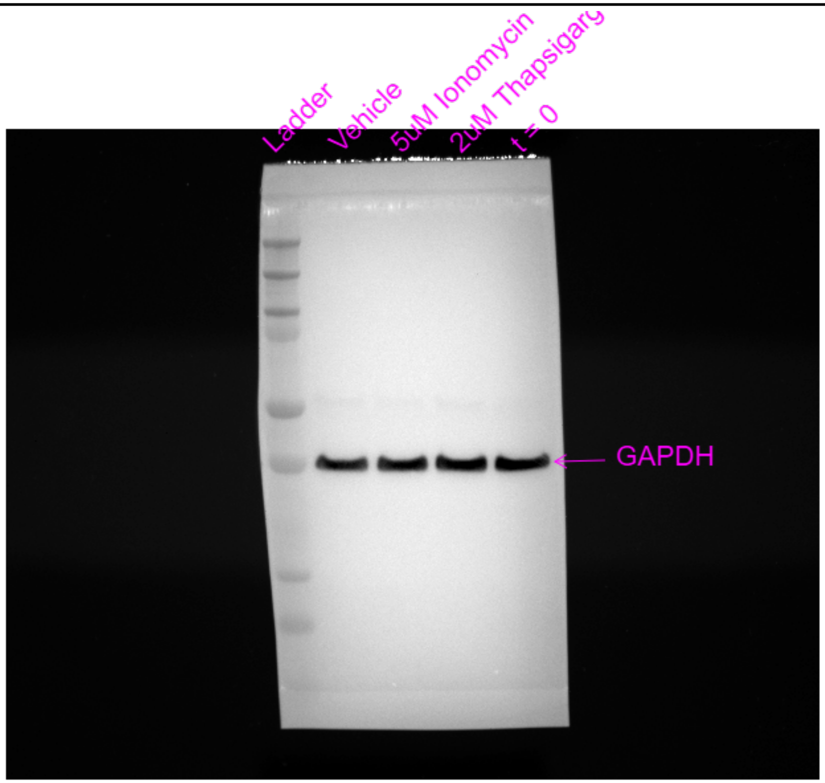

GAPDH CHEMI_03022022_105936

Date:	2 March 2022 10:59:36AM
Mode:	Chemi Blots
Notes:	
Model:	FL1500
Instrument name:	2462619090234
Serial No:	2462619090234
Firmware version:	1.6.0
iBA version:	5.1.0
Image size:	615px X 491px
Image area:	112.7mm X 90.16mm
Optical Zoom:	2x
Digital Zoom:	1.1x
Focus level:	455
Resolution:	5 x 5
Exposure time:	7951 ms
Exposure mode:	Normal

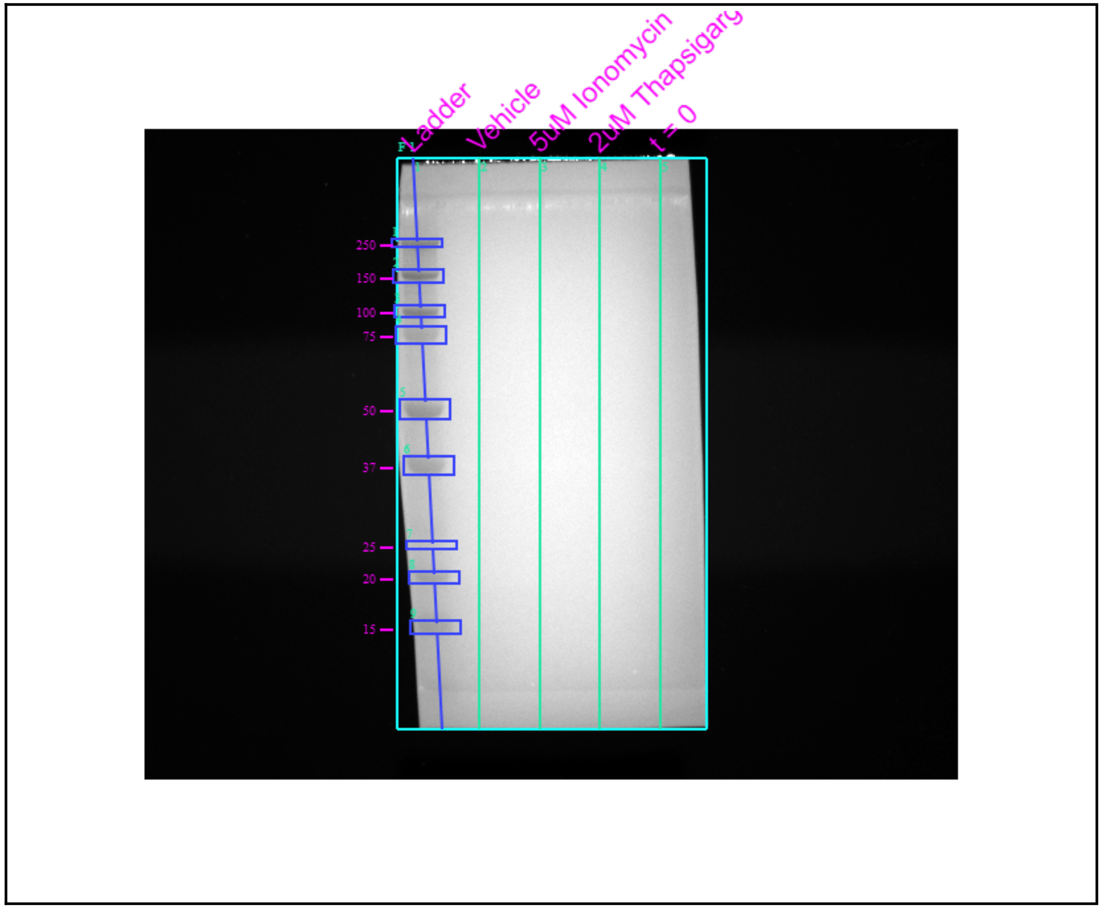

GAPDH CHEMI_03022022_105936

Date:	2 March 2022 10:59:36AM
Mode:	Chemi Blots
Notes:	
Model:	FL1500
Instrument name:	2462619090234
Serial No:	2462619090234
Firmware version:	1.6.0
iBA version:	5.1.0
Image size:	615px X 491px
Image area:	112.7mm X 90.16mm
Optical Zoom:	2x
Digital Zoom:	1.1x
Focus level:	455
Resolution:	5 x 5
Exposure time:	7951 ms
Exposure mode:	Normal

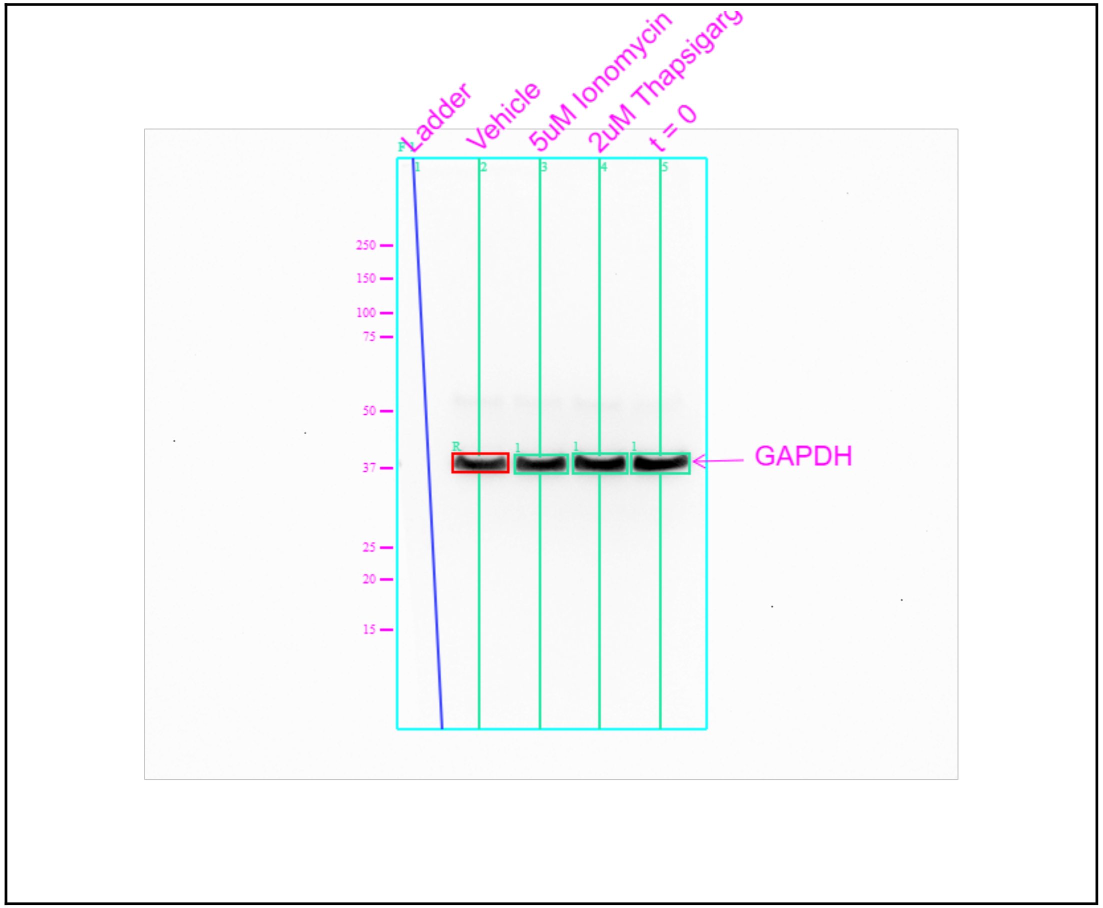

LANE AND BAND ANALYSIS DATA TABLE

GAPDH CHEMI_03022022_105936

Frame: 1
Channel: Membrane
Sensitivity: 100
Molecular Weight Analysis Regression Method : Point to Point

Lane 1 - Ladder

#	Vol. (Int.)	Local Bg. Corr. Vol.	Area	Rf	Density	Local Bg. Corr. Den.	% band purity	% lane purity	Rolling Bg. Corr. Vol.	Rolling Bg. Corr. Den.	Mol. Wt.
1	10,976,734	851,614	273	0.148	40,207	3,119.468	10.463	3.179	1,741,824	6,380.308	250
2	16,009,612	1,153,106	429	0.206	37,318	2,687.893	14.219	4.319	2,366,976	5,517.427	150
3	13,800,750	860,494	390	0.267	35,386	2,206.396	13.716	4.167	2,283,264	5,854.523	100
4	17,100,685	376,436	546	0.309	31,319	689.445	10.157	3.086	1,690,880	3,096.85	75
5	19,515,341	1,388,986	624	0.439	31,274	2,225.941	17.294	5.254	2,878,976	4,613.744	50
6	17,515,879	1,356,550	585	0.538	29,941	2,318.891	13.145	3.993	2,188,288	3,740.663	37
7	7,668,850	16,990	273	0.677	28,091	62.236	1.888	0.574	314,368	1,151.531	25
8	12,066,147	75,141	390	0.733	30,938	192.672	6.928	2.105	1,153,280	2,957.128	20
9	14,777,304	659,784	429	0.821	34,445	1,537.959	12.189	3.703	2,029,056	4,729.734	15

Frame: 1
Channel: Chemi
Sensitivity: 100
Molecular Weight Analysis Regression Method : Point to Point

Lane 2 - Vehicle

#	Vol. (Int.)	Local Bg. Corr. Vol.	Area	Rf	Density	Local Bg. Corr. Den.
1	14,045,964	11,734,890	645	0.534	21,776	18,193

#	% band purity	% lane purity	Rolling Bg. Corr. Vol.	Rolling Bg. Corr. Den.	Mol. Wt.	Rel. Quant. (w/ LB Corr. Vol.)
1	100	89.58	12,696,576	19,684	37.605	1

Lane 3 - 5uM Ionomycin

#	Vol. (Int.)	Local Bg. Corr. Vol.	Area	Rf	Density	Local Bg. Corr. Den.
1	15,051,292	12,113,525	615	0.536	24,473	19,696

#	% band purity	% lane purity	Rolling Bg. Corr. Vol.	Rolling Bg. Corr. Den.	Mol. Wt.	Rel. Quant. (w/ LB Corr. Vol.)

#	% band purity	% lane purity	Rolling Bg. Corr. Vol.	Rolling Bg. Corr. Den.	Mol. Wt.	Rel. Quant. (w/ LB Corr. Vol.)
1	100	87.324	13,748,992	22,356	37.302	1.032

Lane 4 - 2uM Thapsigargin

#	Vol. (Int.)	Local Bg. Corr. Vol.	Area	Rf	Density	Local Bg. Corr. Den.	
1	17,331,197	13,791,429	672	0.534	25,790	20,522	

#	% band purity	% lane purity	Rolling Bg. Corr. Vol.	Rolling Bg. Corr. Den.	Mol. Wt.	Rel. Quant. (w/ LB Corr. Vol.)
1	100	88.909	15,844,864	23,578	37.605	1.175

Lane 5 - t = 0

#	Vol. (Int.)	Local Bg. Corr. Vol.	Area	Rf	Density	Local Bg. Corr. Den.	
1	18,375,381	15,605,447	720	0.534	25,521	21,674	

#	% band purity	% lane purity	Rolling Bg. Corr. Vol.	Rolling Bg. Corr. Den.	Mol. Wt.	Rel. Quant. (w/ LB Corr. Vol.)
1	100	94.472	16,904,192	23,478	37.605	1.33

iBright™ Image Analysis Report

Katarina+ Chang

19 November 2022

GAPDH CHEMI_03022022_110703

Date:	2 March 2022 11:07:03AM
Mode:	Chemi Blots
Notes:	
Model:	FL1500
Instrument name:	2462619090234
Serial No:	2462619090234
Firmware version:	1.6.0
iBA version:	5.1.0
Image size:	615px X 491px
Image area:	112.7mm X 90.16mm
Optical Zoom:	2x
Digital Zoom:	1.1x
Focus level:	455
Resolution:	5 x 5
Exposure time:	10000 ms
Exposure mode:	Normal

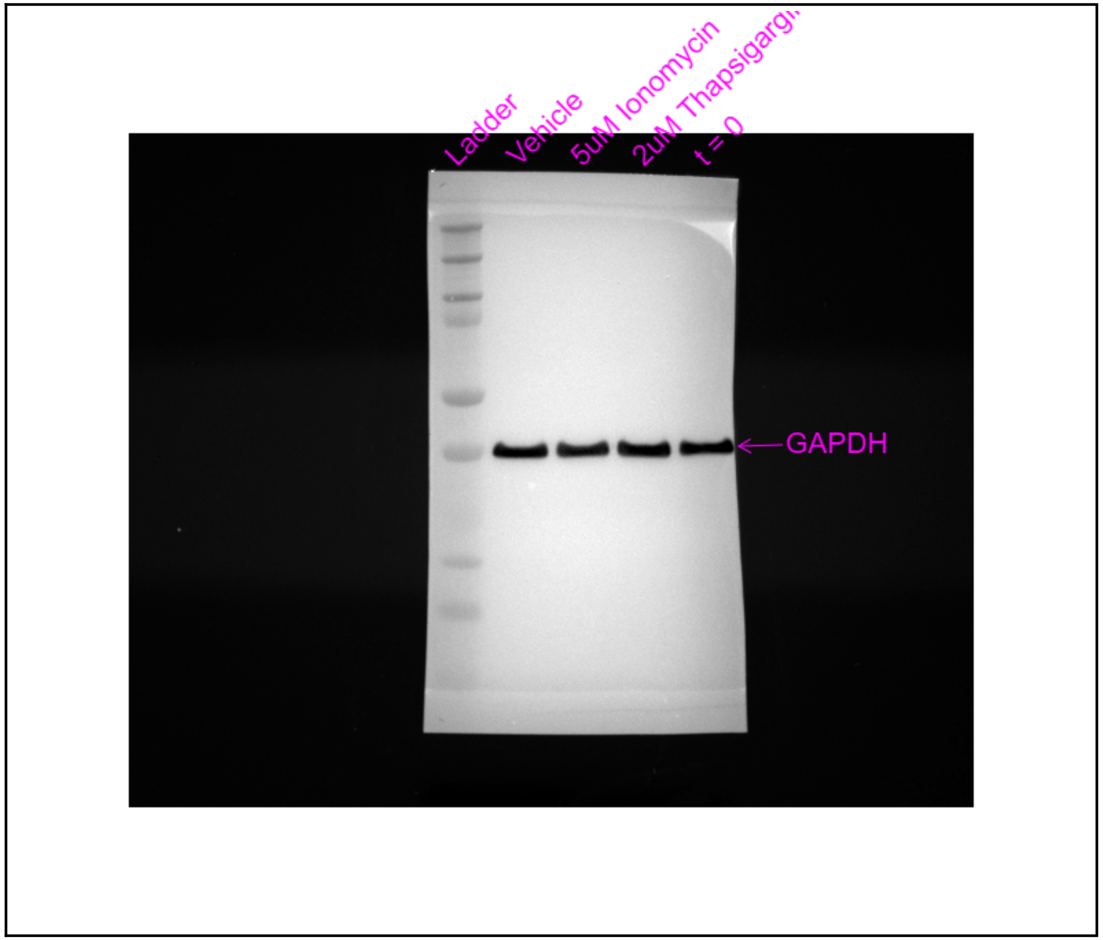

GAPDH CHEMI_03022022_110703

Date:	2 March 2022 11:07:03AM
Mode:	Chemi Blots
Notes:	
Model:	FL1500
Instrument name:	2462619090234
Serial No:	2462619090234
Firmware version:	1.6.0
iBA version:	5.1.0
Image size:	615px X 491px
Image area:	112.7mm X 90.16mm
Optical Zoom:	2x
Digital Zoom:	1.1x
Focus level:	455
Resolution:	5 x 5
Exposure time:	10000 ms
Exposure mode:	Normal

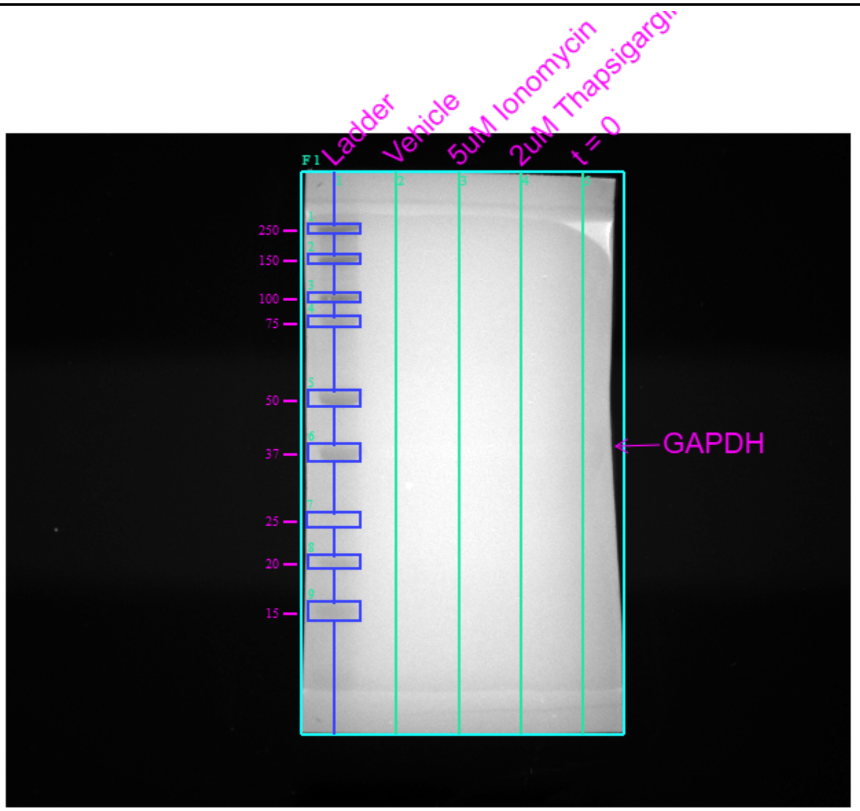

GAPDH CHEMI_03022022_110703

Date:	2 March 2022 11:07:03AM
Mode:	Chemi Blots
Notes:	
Model:	FL1500
Instrument name:	2462619090234
Serial No:	2462619090234
Firmware version:	1.6.0
iBA version:	5.1.0
Image size:	615px X 491px
Image area:	112.7mm X 90.16mm
Optical Zoom:	2x
Digital Zoom:	1.1x
Focus level:	455
Resolution:	5 x 5
Exposure time:	10000 ms
Exposure mode:	Normal

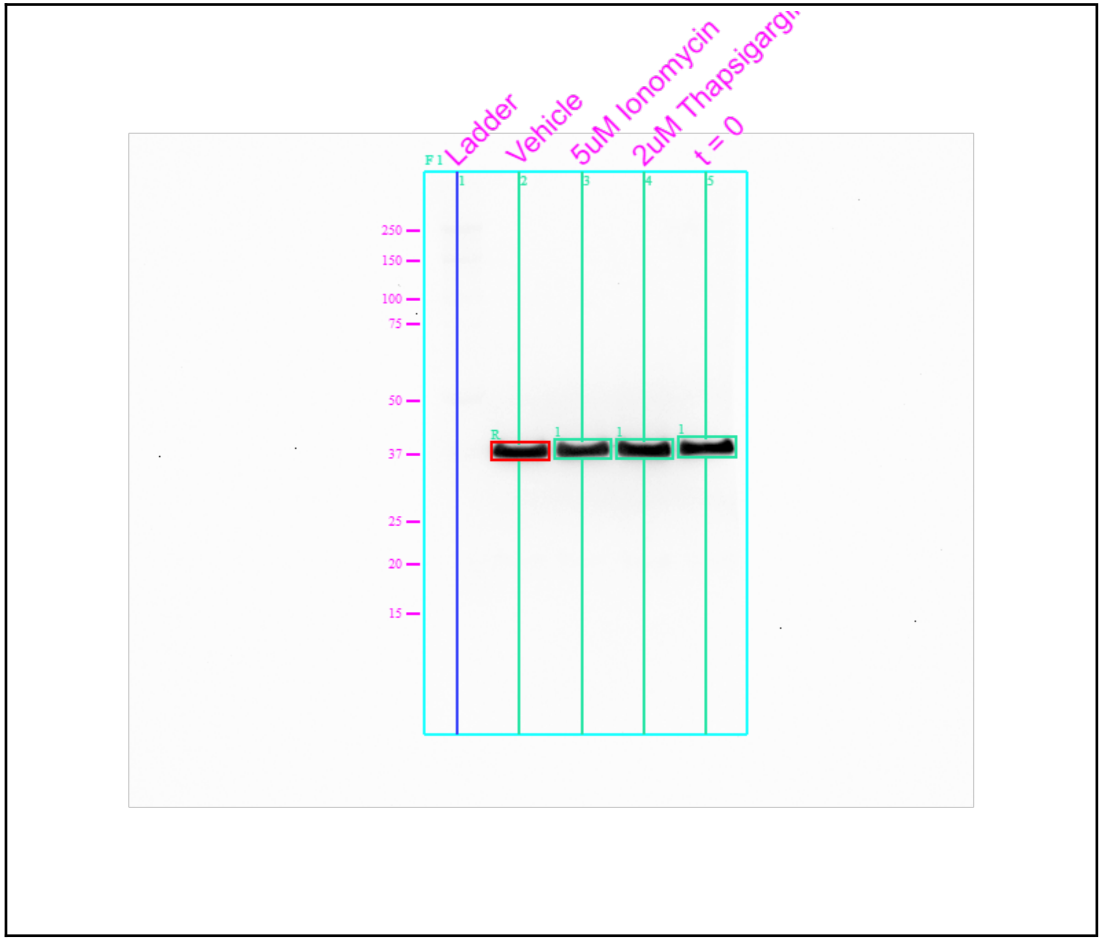

LANE AND BAND ANALYSIS DATA TABLE

GAPDH CHEMI_03022022_110703

Frame: 1
Channel: Membrane
Sensitivity: 100
Molecular Weight Analysis Regression Method : Point to Point

Lane 1 - Ladder

#	Vol. (Int.)	Local Bg. Corr. Vol.	Area	Rf	Density	Local Bg. Corr. Den.	% band purity	% lane purity	Rolling Bg. Corr. Vol.	Rolling Bg. Corr. Den.	Mol. Wt.
1	11,236,070	974,964	312	0.1	36,013	3,124.888	14.244	4.429	1,869,568	5,992.205	250
2	10,826,796	1,006,582	312	0.154	34,701	3,226.224	10.862	3.377	1,425,664	4,569.436	150
3	10,538,890	850,425	312	0.222	33,778	2,725.724	10.833	3.368	1,421,824	4,557.128	100
4	10,861,266	176,127	351	0.266	30,943	501.789	7.102	2.208	932,096	2,655.544	75
5	15,634,864	728,647	507	0.402	30,837	1,437.175	17.67	5.494	2,319,104	4,574.17	50
6	16,154,671	376,981	546	0.498	29,587	690.442	15.822	4.92	2,076,672	3,803.429	37
7	12,859,695	NA	480	0.617	26,791	NA	2.684	0.835	352,256	733.867	25
8	12,458,646	113,072	429	0.693	29,041	263.573	8.627	2.682	1,132,288	2,639.366	20
9	17,684,451	222,304	585	0.78	30,229	380.008	12.155	3.78	1,595,392	2,727.166	15

Frame: 1
Channel: Chemi
Sensitivity: 100
Molecular Weight Analysis Regression Method : Point to Point

Lane 2 - Vehicle

#	Vol. (Int.)	Local Bg. Corr. Vol.	Area	Rf	Density	Local Bg. Corr. Den.
1	19,356,415	16,395,455	602	0.495	32,153	27,234

#	% band purity	% lane purity	Rolling Bg. Corr. Vol.	Rolling Bg. Corr. Den.	Mol. Wt.	Rel. Quant. (w/ LB Corr. Vol.)
1	100	83.345	6,869,248	11,410	37.333	1

Lane 3 - 5uM Ionomycin

#	Vol. (Int.)	Local Bg. Corr. Vol.	Area	Rf	Density	Local Bg. Corr. Den.
1	18,010,765	14,159,637	630	0.493	28,588	22,475

#	% band purity	% lane purity	Rolling Bg. Corr. Vol.	Rolling Bg. Corr. Den.	Mol. Wt.	Rel. Quant. (w/ LB Corr. Vol.)

#	% band purity	% lane purity	Rolling Bg. Corr. Vol.	Rolling Bg. Corr. Den.	Mol. Wt.	Rel. Quant. (w/ LB Corr. Vol.)
1	100	90.204	16,239,360	25,776	37.667	0.864

Lane 4 - 2uM Thapsigargin

#	Vol. (Int.)	Local Bg. Corr. Vol.	Area	Rf	Density	Local Bg. Corr. Den.
1	20,710,474	16,591,412	630	0.493	32,873	26,335

#	% band purity	% lane purity	Rolling Bg. Corr. Vol.	Rolling Bg. Corr. Den.	Mol. Wt.	Rel. Quant. (w/ LB Corr. Vol.)
1	100	89.756	18,883,328	29,973	37.667	1.012

Lane 5 - t = 0

#	Vol. (Int.)	Local Bg. Corr. Vol.	Area	Rf	Density	Local Bg. Corr. Den.
1	20,132,432	18,174,623	688	0.488	29,262	26,416

#	% band purity	% lane purity	Rolling Bg. Corr. Vol.	Rolling Bg. Corr. Den.	Mol. Wt.	Rel. Quant. (w/ LB Corr. Vol.)
1	100	92.904	19,088,640	27,745	38.333	1.109

iBright™ Image Analysis Report

Katarina+ Chang

19 November 2022

Total MLC CHEMI_03012022_114513

Date:	1 March 2022 11:45:13AM
Mode:	Chemi Blots
Notes:	
Model:	FL1500
Instrument name:	2462619090234
Serial No:	2462619090234
Firmware version:	1.6.0
iBA version:	5.1.0
Image size:	676px X 540px
Image area:	112.7mm X 90.16mm
Optical Zoom:	2x
Digital Zoom:	1x
Focus level:	455
Resolution:	5 x 5
Exposure time:	13548 ms
Exposure mode:	Normal

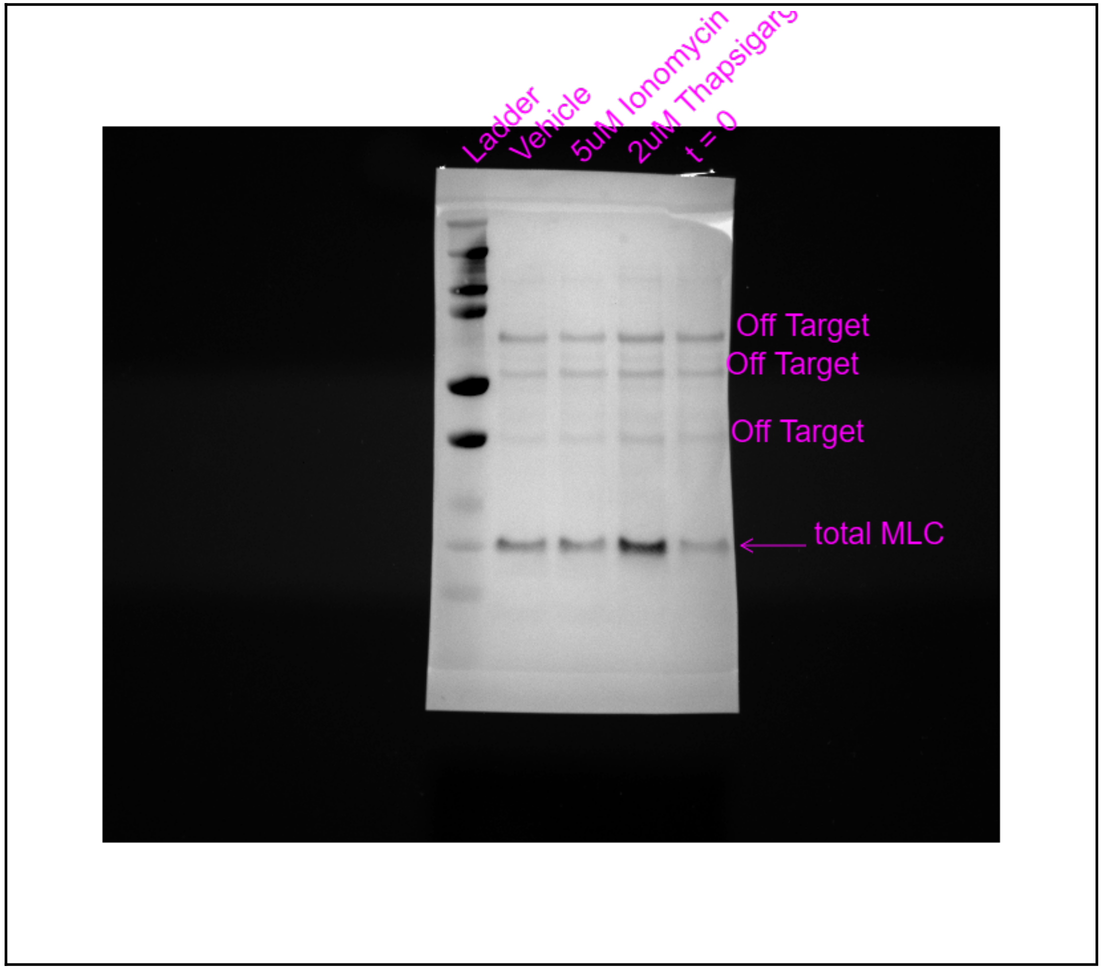

Total MLC CHEMI_03012022_114513

Date:	1 March 2022 11:45:13AM
Mode:	Chemi Blots
Notes:	
Model:	FL1500
Instrument name:	2462619090234
Serial No:	2462619090234
Firmware version:	1.6.0
iBA version:	5.1.0
Image size:	676px X 540px
Image area:	112.7mm X 90.16mm
Optical Zoom:	2x
Digital Zoom:	1x
Focus level:	455
Resolution:	5 x 5
Exposure time:	13548 ms
Exposure mode:	Normal

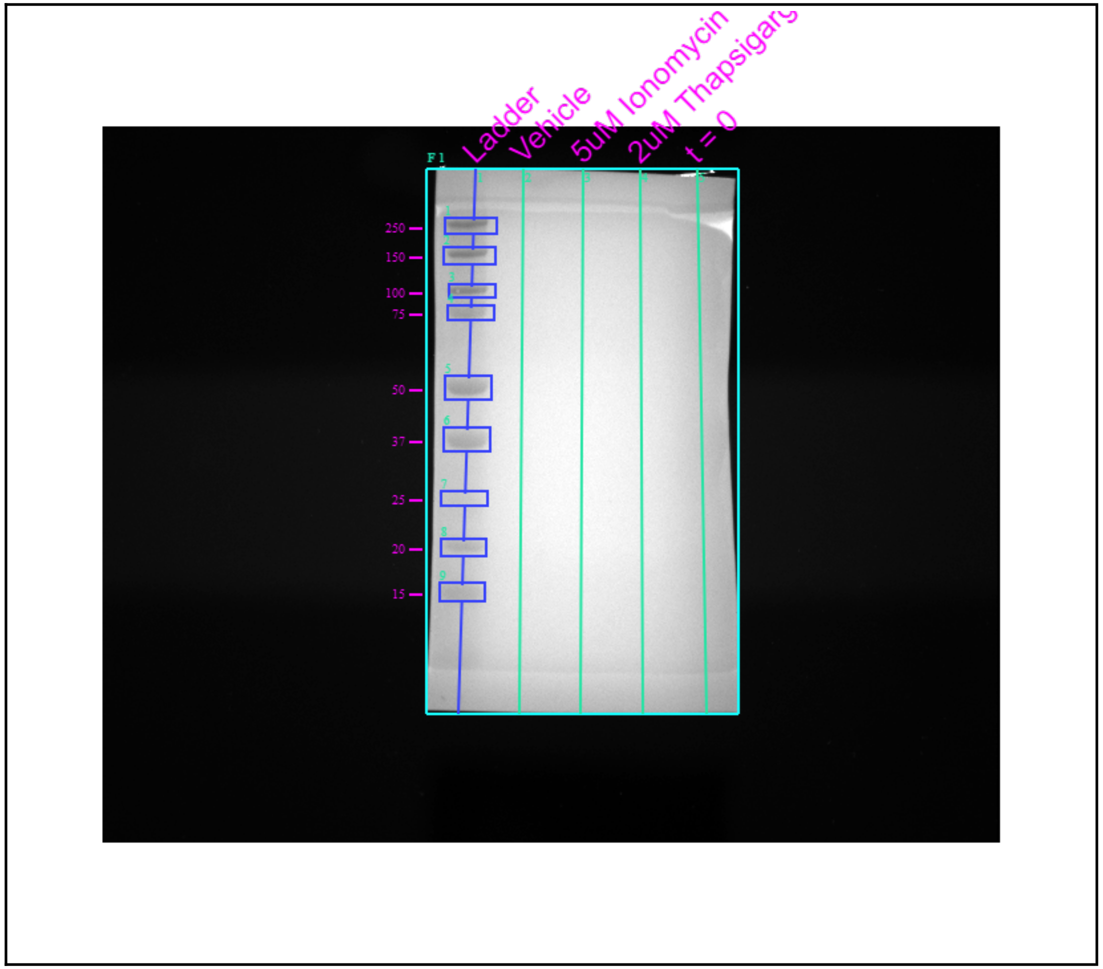

Total MLC CHEMI_03012022_114513

Date:	1 March 2022 11:45:13AM
Mode:	Chemi Blots
Notes:	
Model:	FL1500
Instrument name:	2462619090234
Serial No:	2462619090234
Firmware version:	1.6.0
iBA version:	5.1.0
Image size:	676px X 540px
Image area:	112.7mm X 90.16mm
Optical Zoom:	2x
Digital Zoom:	1x
Focus level:	455
Resolution:	5 x 5
Exposure time:	13548 ms
Exposure mode:	Normal

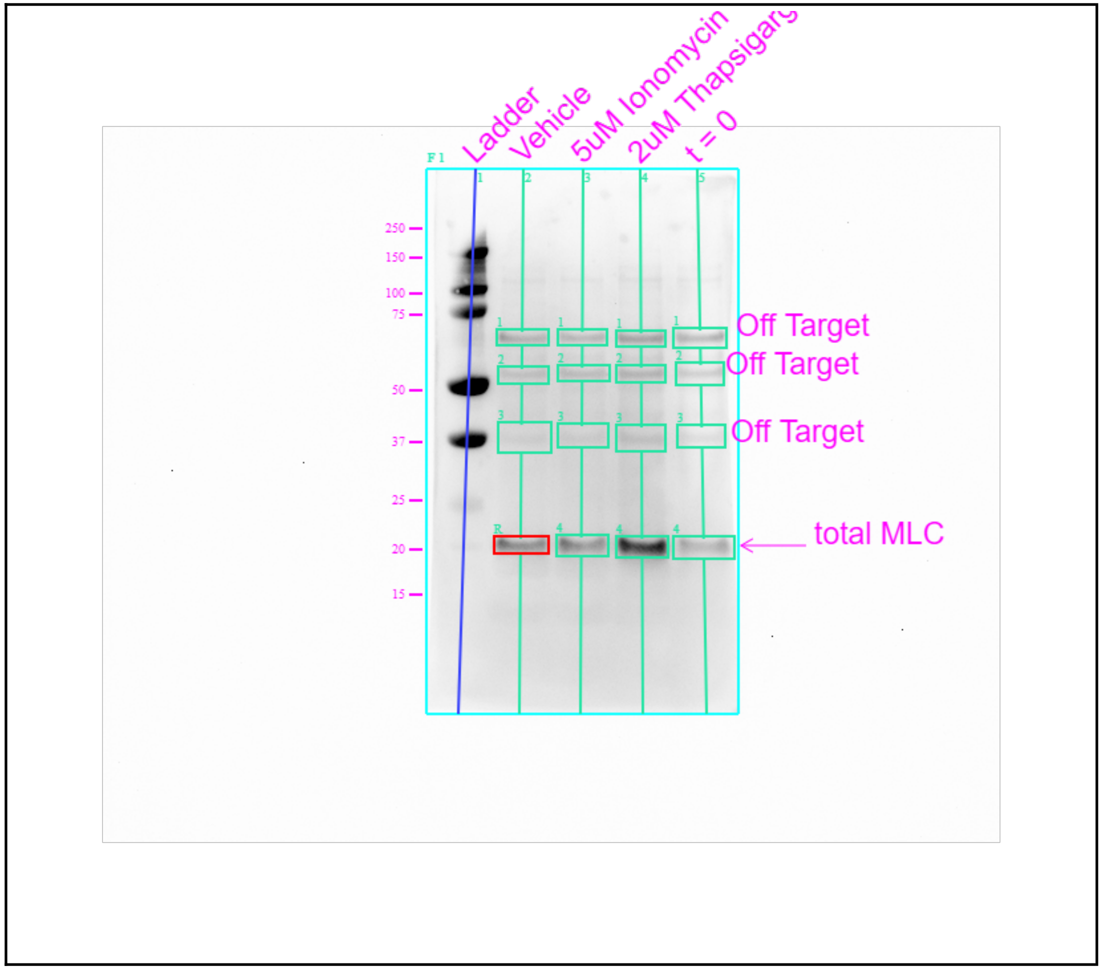

LANE AND BAND ANALYSIS DATA TABLE

Total MLC CHEMI_03012022_114513

Frame: 1
Channel: Membrane
Sensitivity: 100
Molecular Weight Analysis Regression Method : Point to Point

Lane 1 - Ladder

#	Vol. (Int.)	Local Bg. Corr. Vol.	Area	Rf	Density	Local Bg. Corr. Den.	% band purity	% lane purity	Rolling Bg. Corr. Vol.	Rolling Bg. Corr. Den.	Mol. Wt.
1	18,026,875	2,422,277	520	0.105	34,667	4,658.227	16.623	3.823	2,686,720	5,166.769	250
2	18,217,554	2,325,612	560	0.158	32,531	4,152.88	12.299	2.828	1,987,840	3,549.714	150
3	12,784,832	1,893,283	396	0.224	32,284	4,781.019	10.417	2.396	1,683,712	4,251.798	100
4	13,151,997	1,505,062	432	0.263	30,444	3,483.942	8.086	1.859	1,306,880	3,025.185	75
5	19,909,527	2,899,959	684	0.401	29,107	4,239.706	16.634	3.825	2,688,512	3,930.573	50
6	19,272,673	2,569,917	684	0.496	28,176	3,757.189	14.949	3.438	2,416,128	3,532.351	37
7	11,329,242	554,639	432	0.603	26,225	1,283.888	3.923	0.902	634,112	1,467.852	25
8	13,873,804	1,310,690	490	0.693	28,313	2,674.878	7.801	1.794	1,260,800	2,573.061	20
9	15,618,023	1,509,576	525	0.776	29,748	2,875.384	9.269	2.131	1,498,112	2,853.547	15

Frame: 1
Channel: Chemi
Sensitivity: 100
Molecular Weight Analysis Regression Method : Point to Point

Lane 2 - Vehicle

#	Vol. (Int.)	Local Bg. Corr. Vol.	Area	Rf	Density	Local Bg. Corr. Den.
1	7,482,736	2,700,961	520	0.309	14,389	5,194.157
2	7,028,694	1,301,721	546	0.377	12,873	2,384.106
3	10,420,283	907,882	984	0.491	10,589	922.645
4	12,098,981	6,141,829	588	0.689	20,576	10,445

#	% band purity	% lane purity	Rolling Bg. Corr. Vol.	Rolling Bg. Corr. Den.	Mol. Wt.	Rel. Quant. (w/ LB Corr. Vol.)
1	23.409	14.045	2,716,672	5,224.369	66.667	0.44
2	12.459	7.475	1,445,888	2,648.147	54.386	0.212
3	6.181	3.708	717,312	728.976	37.667	0.148

#	% band purity	% lane purity	Rolling Bg. Corr. Vol.	Rolling Bg. Corr. Den.	Mol. Wt.	Rel. Quant. (w/ LB Corr. Vol.)
4	57.951	34.77	6,725,376	11,437	20.27	1

Lane 3 - 5uM Ionomycin

#	Vol. (Int.)	Local Bg. Corr. Vol.	Area	Rf	Density	Local Bg. Corr. Den.
1	6,149,745	2,029,281	494	0.309	12,448	4,107.858
2	6,814,318	1,483,695	520	0.375	13,104	2,853.26
3	8,663,529	776,272	741	0.489	11,691	1,047.602
4	13,216,890	5,292,179	680	0.691	19,436	7,782.617

#	% band purity	% lane purity	Rolling Bg. Corr. Vol.	Rolling Bg. Corr. Den.	Mol. Wt.	Rel. Quant. (w/ LB Corr. Vol.)
1	19.043	11.243	2,113,024	4,277.377	66.667	0.33
2	15.7	9.269	1,742,080	3,350.154	54.825	0.242
3	6.608	3.901	733,184	989.452	38	0.126
4	58.648	34.626	6,507,520	9,569.882	20.135	0.862

Lane 4 - 2uM Thapsigargin

#	Vol. (Int.)	Local Bg. Corr. Vol.	Area	Rf	Density	Local Bg. Corr. Den.
1	7,640,352	3,513,944	494	0.311	15,466	7,113.248
2	7,496,771	2,126,871	532	0.375	14,091	3,997.879
3	10,336,156	1,940,278	798	0.494	12,952	2,431.427
4	22,456,628	13,059,473	680	0.693	33,024	19,205

#	% band purity	% lane purity	Rolling Bg. Corr. Vol.	Rolling Bg. Corr. Den.	Mol. Wt.	Rel. Quant. (w/ LB Corr. Vol.)
1	16.254	11.034	3,404,288	6,891.271	66.228	0.572
2	10.744	7.294	2,250,240	4,229.774	54.825	0.346
3	6.113	4.15	1,280,256	1,604.331	37.333	0.316
4	66.89	45.409	14,009,600	20,602	20	2.126

Lane 5 - t = 0

#	Vol. (Int.)	Local Bg. Corr. Vol.	Area	Rf	Density	Local Bg. Corr. Den.
1	6,089,253	3,195,011	656	0.309	9,282.398	4,870.444
2	5,169,817	1,708,684	666	0.375	7,762.488	2,565.593
3	4,484,152	978,670	666	0.489	6,732.961	1,469.476
4	10,541,805	4,329,960	846	0.693	12,460	5,118.156

#	% band purity	% lane purity	Rolling Bg. Corr. Vol.	Rolling Bg. Corr. Den.	Mol. Wt.	Rel. Quant. (w/ LB Corr. Vol.)
1	29.632	15.502	3,239,424	4,938.146	66.667	0.52
2	18.523	9.69	2,024,960	3,040.48	54.825	0.278
3	9.28	4.855	1,014,528	1,523.315	38	0.159
4	42.565	22.268	4,653,312	5,500.369	20	0.705
