# Supplementary material for: Elevation of Cytoplasmic Calcium Suppresses Microtentacle Formation and Function in Breast Tumor Cells
Source: Cancers (Basel). 2023 Jan 31;15(3):884. doi: 10.3390/cancers15030884 (PMC9913253; doi:10.3390/cancers15030884)
Supplement: Supplementary file 1 [file cancers-15-00884-s001.zip › cancers-2080686-Supplementary/Supplemental Method.pdf]

1. Supplementary Methods:

1.1. Chemiluminescence and Molecular Weights: Using the iBright Software (ThermoFisher), the molecular weights of bands of interest can be determined. For each blot, the Dual Color Precision Plus Protein™ Standards (Bio-Rad, Cat#: 1610374) was used. The software allows for each band of the marker to be identified. Using these as the standard, the molecular weight of a band of interest is calculated by the software.
